# Development of Covalent Inhibitors of Chikungunya Virus nsP2 Cysteine Protease Enabled by Direct-to-Biology Synthesis and Screening

**DOI:** 10.1021/acs.jmedchem.5c03672

**Published:** 2026-08-20

**Authors:** Zhengjun Cai, Kan Li, Sainetra Sridhar, Haozhou Tan, Hiwot Demssie, Gaungjin Fan, Wenyi Zhang, Bobby Brooke Herrera, Jun Wang

**Affiliations:** † Department of Medicinal Chemistry, Ernest Mario School of Pharmacy, Rutgers, The State University of New Jersey, Piscataway, New Jersey 08854, United States; ‡ Rutgers Global Health Institute, New Brunswick, New Jersey 08901, United States; ∥ Department of Medicine, Division of Allergy, Immunology, and Infectious Diseases, and Child Health Institute of New Jersey, 12287 Rutgers Robert Wood Johnson Medical School, New Brunswick, New Jersey 08901, United States

## Abstract

Chikungunya virus (CHIKV), an arthropod-borne alphavirus, has emerged as a global health threat due to its rapid transmission and the lack of effective antiviral therapies. The cysteine protease activity of the virus-encoded nonstructural protein 2 (nsP2) is critical for CHIKV replication, as it processes viral polyproteins and counteracts host antiviral defenses, establishing it as a highly attractive target for therapeutic intervention. In this study, we present a rapid drug development platform that integrates covalent docking with direct-to-biology (D2B) synthesis and screening to identify nsP2 inhibitors. Candidates prioritized by in silico docking were synthesized and directly tested in FRET enzymatic assays without purification. This approach led to the identification of several nsP2 inhibitors with diverse chemical scaffolds, potent enzymatic inhibition, and antiviral activity. Together, these findings establish a streamlined strategy for covalent inhibitor development and provide promising leads for CHIKV antiviral development.

## Graphical abstract

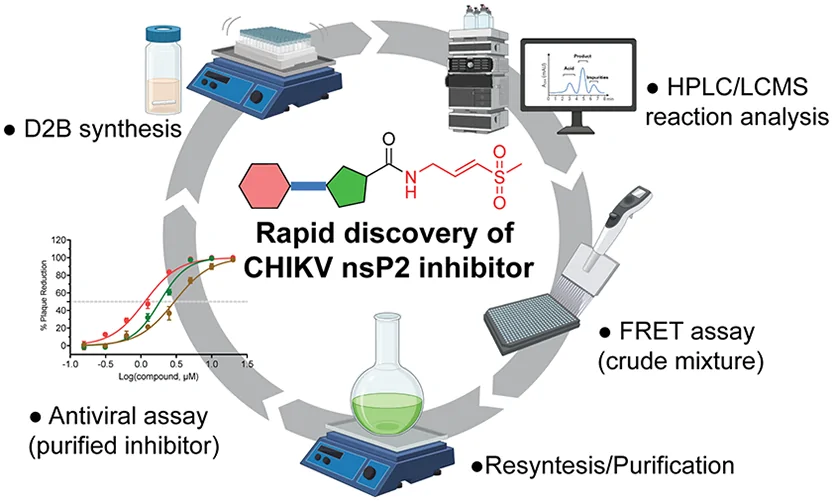

## Introduction

Chikungunya virus (CHIKV) is an arthropod-borne alphavirus of the *Togaviridae* family that causes Chikungunya fever, an acute and often debilitating illness characterized by high fever, rash, myalgia, and severe joint pain.^1,2^ Rarer manifestations, including neurological complications, severe febrile illness, and even death, can occur in vulnerable populations such as young children, the elderly, and individuals with underlying health conditions.^3^ The virus was first identified in Tanzania in 1952 and has since become one of the most widely distributed arboviruses globally.^4,5^ Over the past two decades, CHIKV has spread to more than 100 countries across Africa, Asia, the Pacific, and the Americas, causing an estimated 10 million reported infections.^6^

Transmission occurs primarily through the bites of infected *Aedes aegypti* and *Aedes albopictus* mosquitoes—species that have adapted to urban environments and expanded their geographic range due to global warming, rapid urbanization, and increased international travel.^7,8^ These environmental and societal factors have prolonged mosquito breeding seasons and facilitated viral transmission in regions previously considered nonendemic, including southern Europe, the United States, and East Asia.^9^ CHIKV first appeared in the Americas in 2013 on the island of St. Martin, spreading rapidly across the Caribbean and South America, resulting in nearly one million reported cases by 2016.^10^ More recent resurgences underscore the virus’s re-emergent potential: between October 2022 and April 2023, Paraguay experienced an epidemic with approximately 120,000 confirmed cases,^11^ and in 2025, Guangdong Province in southern China reported over 7000 laboratory-confirmed infections within months,^12^ marking one of the largest CHIKV outbreaks in East Asia to date.

While the acute febrile phase of infection typically lasts one to two weeks, many patients experience lingering musculoskeletal symptoms, including chronic arthralgia, swelling, and stiffness of the wrists, ankles, and small joints.^13^ These long-term complications can persist for months to years, causing fatigue, reduced mobility, and significant socioeconomic impact due to loss of productivity and increased healthcare costs. The high attack rate during outbreaks—often exceeding 30% in affected populations—combined with the potential for chronic sequelae, underscores the substantial global disease burden imposed by CHIKV.

Despite its clinical and public health significance, there are currently no FDA-approved antiviral drugs for CHIKV.^14–16^ Although two CHIKV vaccines have been licensed in the United States, their protection is limited: the live-attenuated vaccine IXCHIQ was suspended by the FDA due to safety concerns, while the virus-like particle vaccine VIMKUNYA is currently intended only for adolescents and adult travelers. Reflecting its growing global impact and potential for re-emergence, CHIKV is classified by the National Institute of Allergy and Infectious Diseases (NIAID) as a Category C priority pathogen, underscoring the critical importance of continued research into CHIKV biology, transmission, and antiviral drug development.

The CHIKV genome consists of a positive-sense, single-stranded RNA approximately 11.8 kb in length.^17^ It encodes four nonstructural proteins (nsP1–nsP4) involved in viral RNA replication and structural proteins responsible for virion assembly. Among these, nonstructural protein 2 (nsP2) is central to the viral life cycle.^16,18^ NsP2 is a multifunctional protein that contains an N-terminal RNA-specific helicase domain, a papain-like cysteine protease domain, and a C-terminal methyltransferase domain. The protease activity of nsP2 plays an essential role in the post-translational processing of the viral nonstructural polyprotein by cleaving the nsp1/2, nsp2/3, and nsp3/4 junctions (Figure 1A).^19^ Proteolytic cleavage by nsP2 generates the individual nsP1, nsP2, nsP3, and nsP4 proteins, which assemble to form the viral RNA replicase. In addition to its enzymatic functions, CHIKV nsP2 has been shown to modulate host antiviral responses.^20^ It can translocate into the nucleus of infected cells and interfere with host gene expression, including the suppression of type I interferon responses, thereby promoting immune evasion.^21^ Given its essential role in viral replication and pathogenesis, the nsP2 protease is considered an attractive target for antiviral drug development.^15^ The CHIKV nsP2 cysteine protease is a papain-like cysteine protease, featuring a catalytic dyad composed of a cysteine (Cys) and a histidine (His) residue. The nsP1/2, nsP2/3, and nsP3/4 junctions are highly conserved. The nsP2-mediated cleavage occurs specifically between the P1 A/C/G and P1′ G/A/Y residues of each site (Figure 1B).

**Figure 1:**
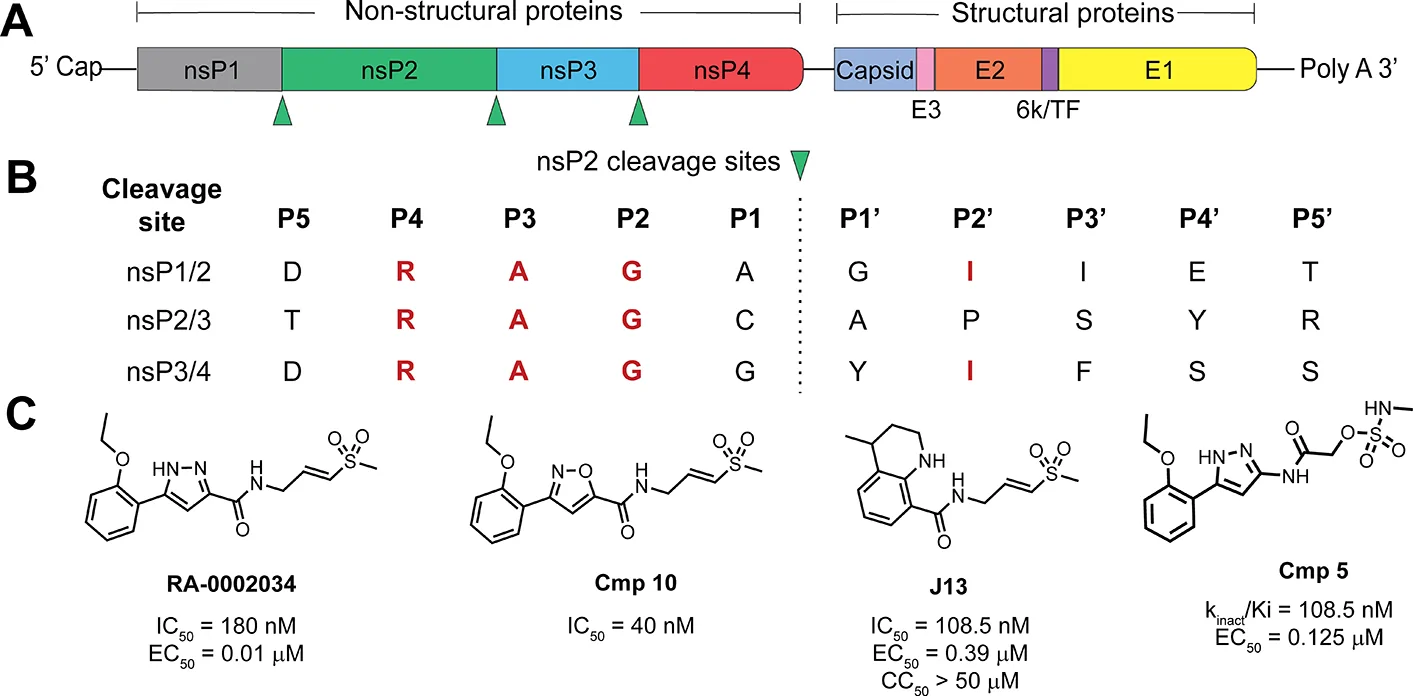
Genome organization of CHIKV, nsP2 substrate sequences, and reported nsP2 inhibitors. (A) CHIKV polyprotein schematic showing the nonstructural ORF (nsP1, Mtase; nsP2, helicase/protease; nsP3, AUD; nsP4, RdRp) and the structural ORF (capsid, E3, E2, E1, 6k/TF). (B) nsP junction cleavage sites processed by the viral protease nsP2, with conserved P and P′ residues highlighted. (C) Chemical structures and activities of β-amidomethyl vinyl sulfone-containing and N-alkyl sulfamate-containing nsP2 inhibitors.

Several nsP2 inhibitors have recently been reported to have potent enzymatic inhibitory and antiviral activities in cell culture (Figure 1C).^15,22–30^ Ogungbe et al. first reported a series of covalent inhibitors containing β-aminomethyl vinyl sulfone warhead as nsP2 inhibitors. Follow-up work led to the development of leads with favorable pharmacokinetic (PK) properties and promising *in vivo* antiviral efficacy.^15,22–25^ Recently, Pearce et al. and Wilson et al. further advanced the field by identifying several nsP2 inhibitors bearing β-aminomethyl vinyl sulfone and N-alkyl sulfamate warheads.^26–30^ Their studies began with fragment-based screening, followed by structure-guided hit-to-lead development and lead optimization, resulting in a series of potent nsP2 inhibitors. Some of the most potent CHIKV nsP2 inhibitors are covalent inhibitors featuring an internal β-amidomethyl vinyl sulfone reactive warhead, including **RA-0002034**,^27^ **compound 10** (**Cmp 10**),^28^ and **J13**.^24^ A recent study explored N-alkyl sulfamates as a new class of warheads with a representative example of **compound 5** ^30^ (Figure 1C). Significantly, the nsP2 protease domain is conserved among alphaviruses, and nsP2 inhibitors have shown broad-spectrum antiviral activity against other alphaviruses, including Western Equine Encephalitis virus (VEEV), Mayaro virus (MAYV), and Ross River virus (RRV).^27,30^

As part of our drug discovery projects targeting viral cysteine proteases, including SARS-CoV-2 main protease and papain-like protease, and the enterovirus D68 2A protease,^31–39^ we are interested in extending our expertise to other viral cysteine proteases such as CHIKV nsP2. In this study, we report the development of a direct-to-biology (D2B) platform that integrates rapid synthesis, crude-mixture enzymatic screening, covalent docking, and structure-guided optimization to accelerate the discovery of covalent nsP2 inhibitors against CHIKV and other alphaviruses. A major advantage of this platform is that it addresses a key rate-limiting step in the design-make-test-analyze (DMTA) cycle: compound synthesis and purification. Conventional medicinal chemistry workflows require sequential synthesis, isolation, purification, and characterization of each analog before biological testing, which is time-consuming and resource-intensive, particularly when broad exploration of substrate-recognition elements and covalent warheads is needed. Related rapid chemistry-to-biology strategies have proven valuable in antiviral drug discovery, including the COVID Moonshot effort and structure-enabled SARS-CoV-2 M^pro^ inhibitor campaigns,^40,41^ where rapid design, synthesis, structural characterization, and biochemical testing accelerated inhibitor discovery. These precedents highlight the importance of shortening the design-to-testing interval for emerging viral targets. Similarly, our D2B workflow enables immediate evaluation of designed nsP2 inhibitor analogs directly from crude reaction mixtures, reducing the synthesis-to-testing cycle from weeks to days. Validation experiments showed a strong correlation between enzymatic inhibition results obtained from crude and purified samples (*R* ^2^ = 0.71), supporting the reliability of this purification-free screening strategy. By combining covalent docking-guided design, rapid D2B synthesis, and direct FRET-based enzymatic screening, this platform enables rapid analog profiling and efficient SAR exploration, leading to the identification of potent nsP2 inhibitors with diverse scaffolds. Thus, the primary innovation of this work is methodological: D2B provides a faster and more resource-efficient framework for advancing viral cysteine protease inhibitor discovery beyond conventional sequential analog synthesis.

## Results and Discussion

### CHIKV nsP2 FRET Assay Development and Optimization

Full-length CHIKV nsP2 was expressed, purified, and utilized to develop a FRET-based enzymatic assay for inhibitor evaluation (Figure S1). The protein’s identity was confirmed by trypsin digestion followed by LC–MS/MS analysis, which provided 92.7% sequence coverage. The FRET enzymatic assay was performed in 384-well plates with a 50 μL reaction volume, yielding stable, reproducible fluorescence signals. The optimized reaction buffer contained 25 mM HEPES (pH 7.2), 0.003% Tween-20, and 1 mM DTT.

To identify the optimal enzyme concentration, nsP2 was titrated from 0.0625 to 4 μM, and fluorescence signals were continuously monitored for over 1 h (Figure 2A). A concentration of 1 μM nsP2 yielded a fully linear signal increase over 1 h with a slope of 2.76 RFU/s and was therefore selected for compound testing. Higher enzyme concentrations (2–4 μM) produced larger signal windows but decreased assay sensitivity due to substrate depletion and diminished inhibitor response. In contrast, lower concentrations (0.0625–0.5 μM) generated narrow or negligible signal windows, limiting the ability to detect inhibitory effects.

**Figure 2:**
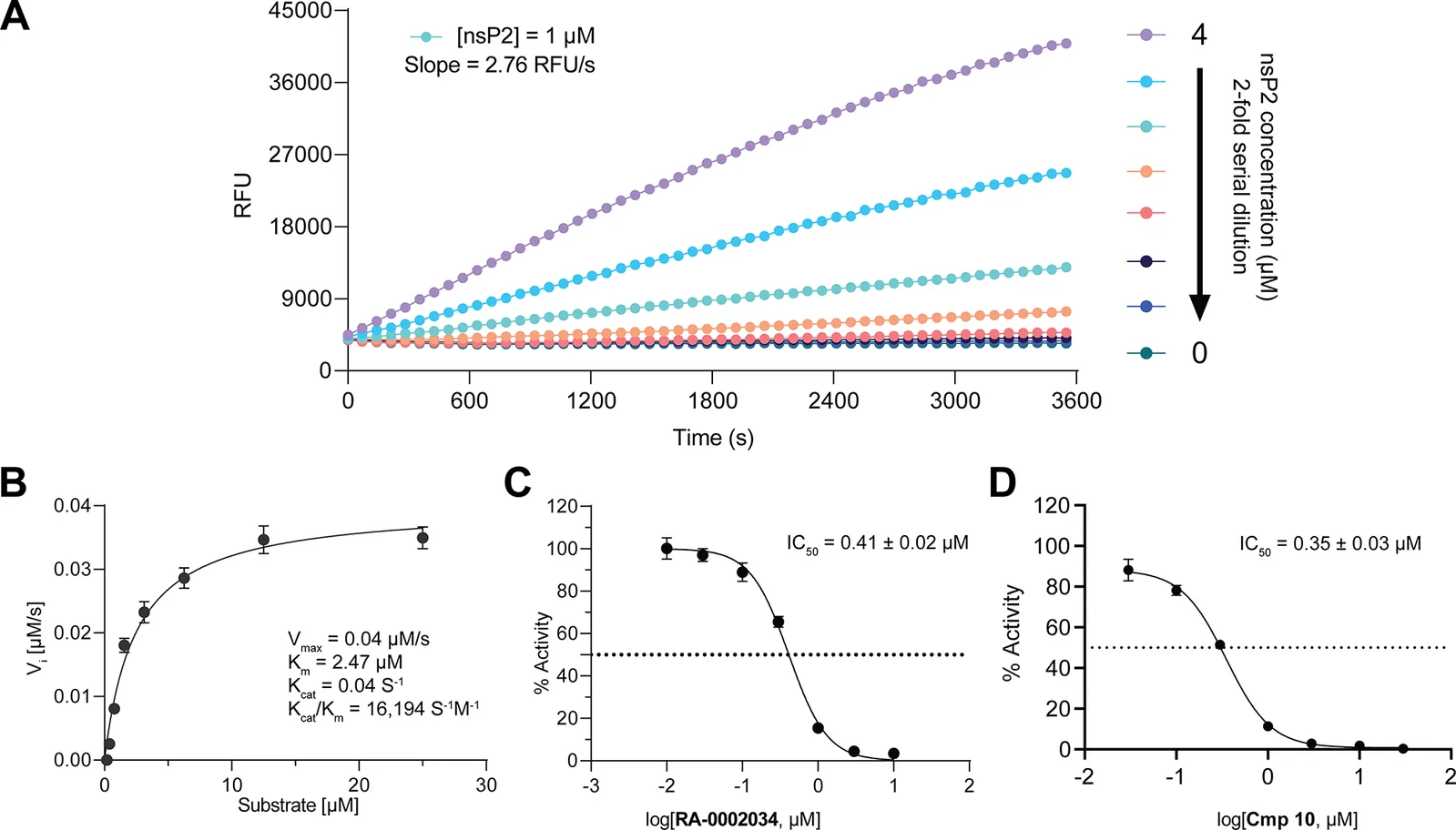
Optimization of the CHIKV nsP2 FRET enzymatic assay. (A) Enzyme titration of CHIKV nsP2. Data shown are representative of two independent experiments. (B) Michaelis–Menten kinetics of CHIKV nsP2. Enzymatic parameters represent the mean of two replicates; error bars indicate the 95% confidence interval. (C) Inhibition of CHIKV nsP2 by **RA-0002034**. (D) Inhibition of CHIKV nsP2 by **Cmp 10**. IC_50_ values were derived from FRET assays and are expressed as mean ± SD (*n* = 2).

Enzyme kinetics were further characterized by varying substrate concentrations (0.2–25 μM). Initial velocities (first 1000 s) were fitted to the Michaelis–Menten equation, yielding a catalytic efficiency (k_cat_/K_m_) of 16,194 M^–1^s^–1^ (Figure 2B). Assay performance was validated using **RA-0002034** and **Cmp 10**, two previously reported covalent nsP2 inhibitors, under the optimized conditions (1 μM nsP2, 20 μM FRET substrate, 50 μL reaction volume, 384-well format).^27,28^ **RA-0002034** and **Cmp 10** exhibited IC_50_ values of 0.41 and 0.35 μM, respectively (Figure 2C).

The reported IC_50_ value for **Cmp 10** was 40 nM.^28^ The discrepancy may arise from differences in the experimental conditions, including the protein construct used (full-length nsp2 in the present study versus the isolated nsp2 protease domain in the original study); the FRET substrate employed (Dabcyl/EDANS in the present study versus QSY-21/Cy5 in the original study); and the enzyme concentration (0.50 μM in the present study versus 0.15 μM in the original study). Therefore, direct comparison of IC_50_ values across different studies is not meaningful unless the assay conditions are closely matched.

### Structure-Based Design of Covalent nsP2 Inhibitors

Previous mechanistic studies suggested that **RA-0002034** forms a covalent complex with CHIKV nsp2.^27^ We therefore performed a covalent docking using the Molecular Operating Environment (MOE). The docking model showed that **RA-0002034** binds at the catalytic site with the distal 2-ethoxy phenyl substituent facing the interface between the N-terminal protease subdomain and the C-terminal MTase domain, in a similar pose as previously reported.^27^ The docking model of nsP2 reveals a groove between the N-terminal protease domain (gray) and the C-terminal MTase domain (cyan) (Figure 3). Our design hypothesis was to extend the distal phenyl substituent in **RA-0002034** to engage additional interactions with residues in this groove (white cycle) (Figure 3). As β-aminomethyl vinyl sulfone has been demonstrated by Ogungbe, Pearce, and Wilson et al. to be a preferred cysteine-reactive warhead for nsP2, we retained this warhead in our inhibitor design and focused primarily on optimizing the noncovalent interactions of the scaffold to improve inhibitor potency and binding. For this, we explored two strategies: one is to extend the substituents on the phenyl ring with **15** as a representative example; another is to insert a flexible benzyl ether linker in between the 5-membered heterocycle and the distal aromatic substituent with **34** as a representative example (Table 1).

**Figure 3:**
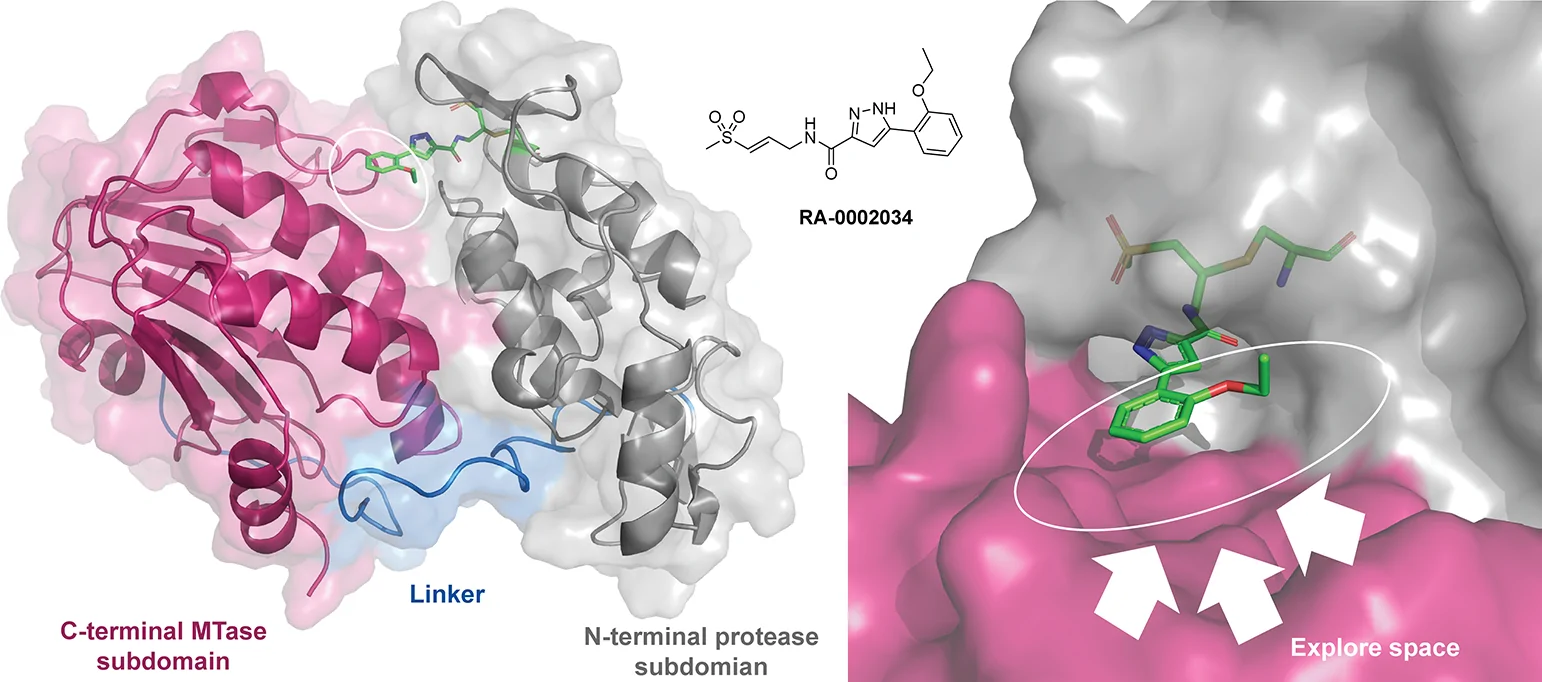
Design principle of CHIKV nsp2 inhibitor. The docking model was generated using Molecular Operating Environment (MOE) covalent docking with the X-ray crystal structure of CHIKV nsP2 (PDB: 3TRK). The N-terminal protease domain, linker, and the C-terminal MTase domain are colored in gray, blue, and magenta, respectively.

**Table 1:**
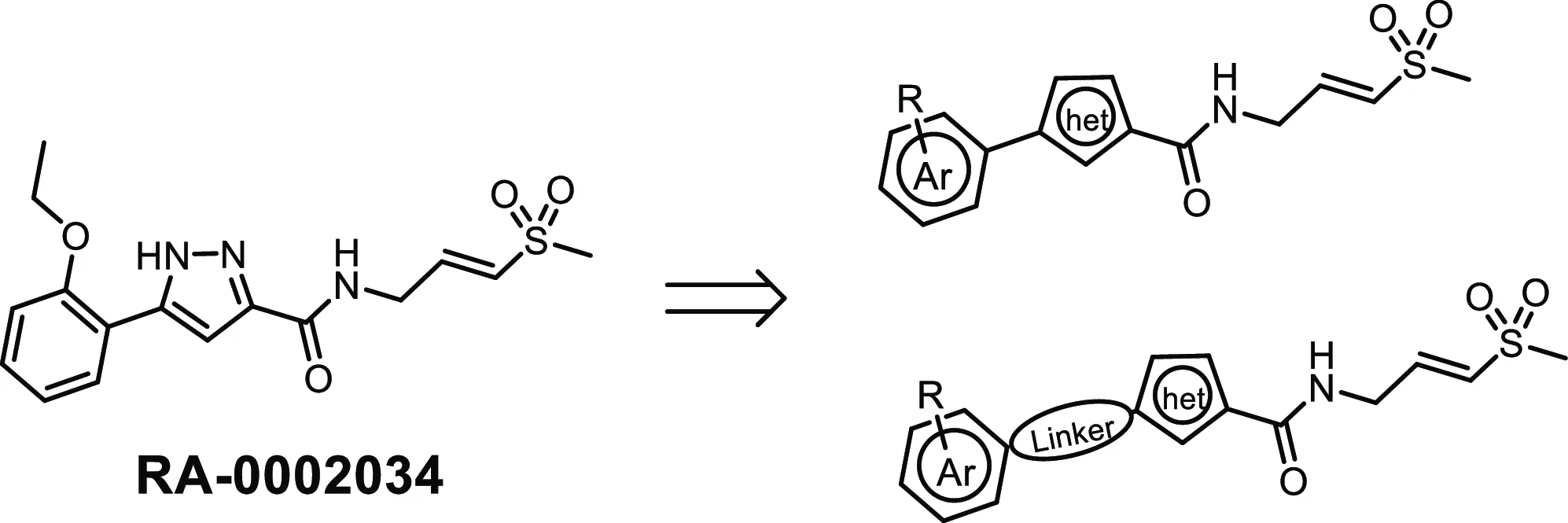

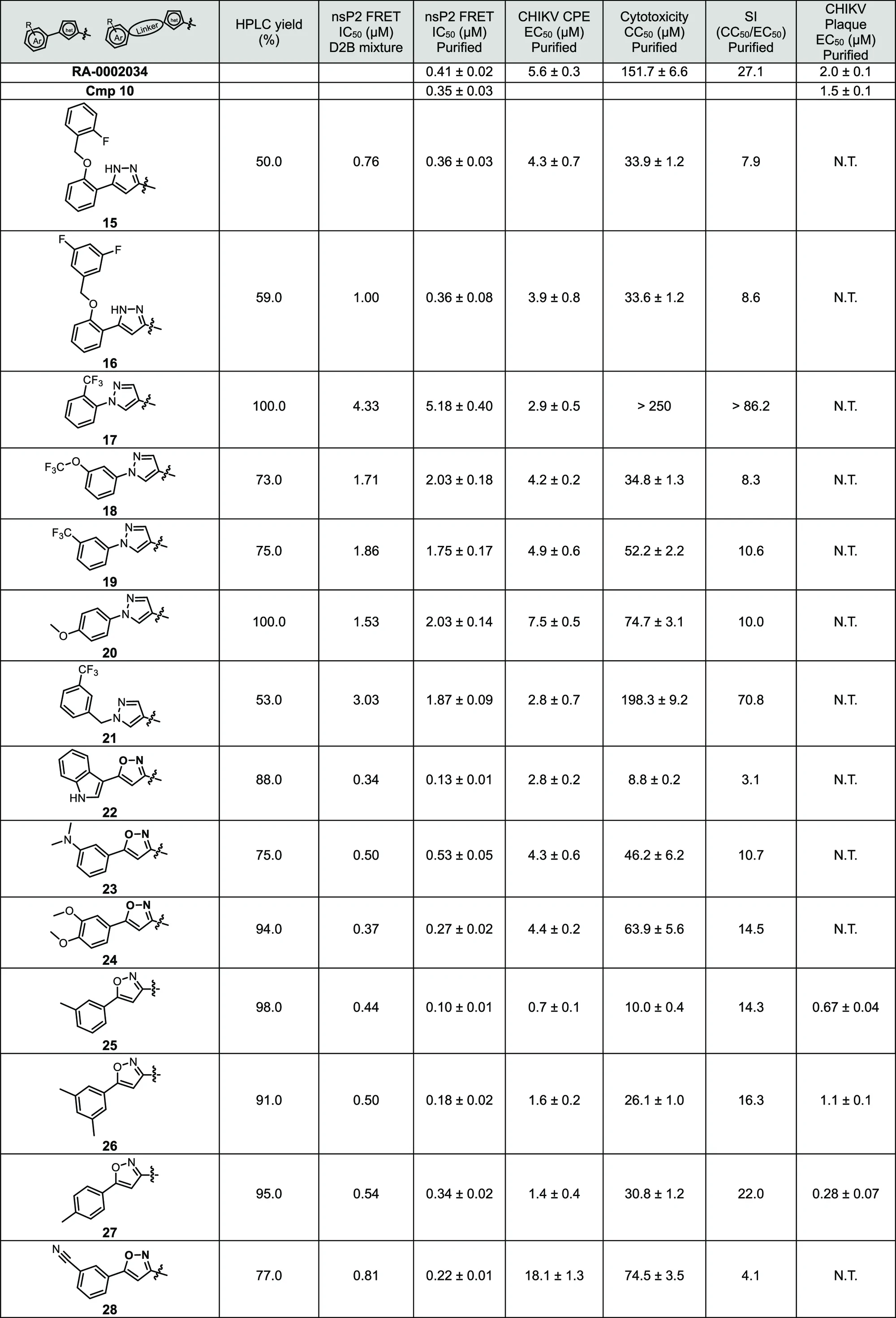

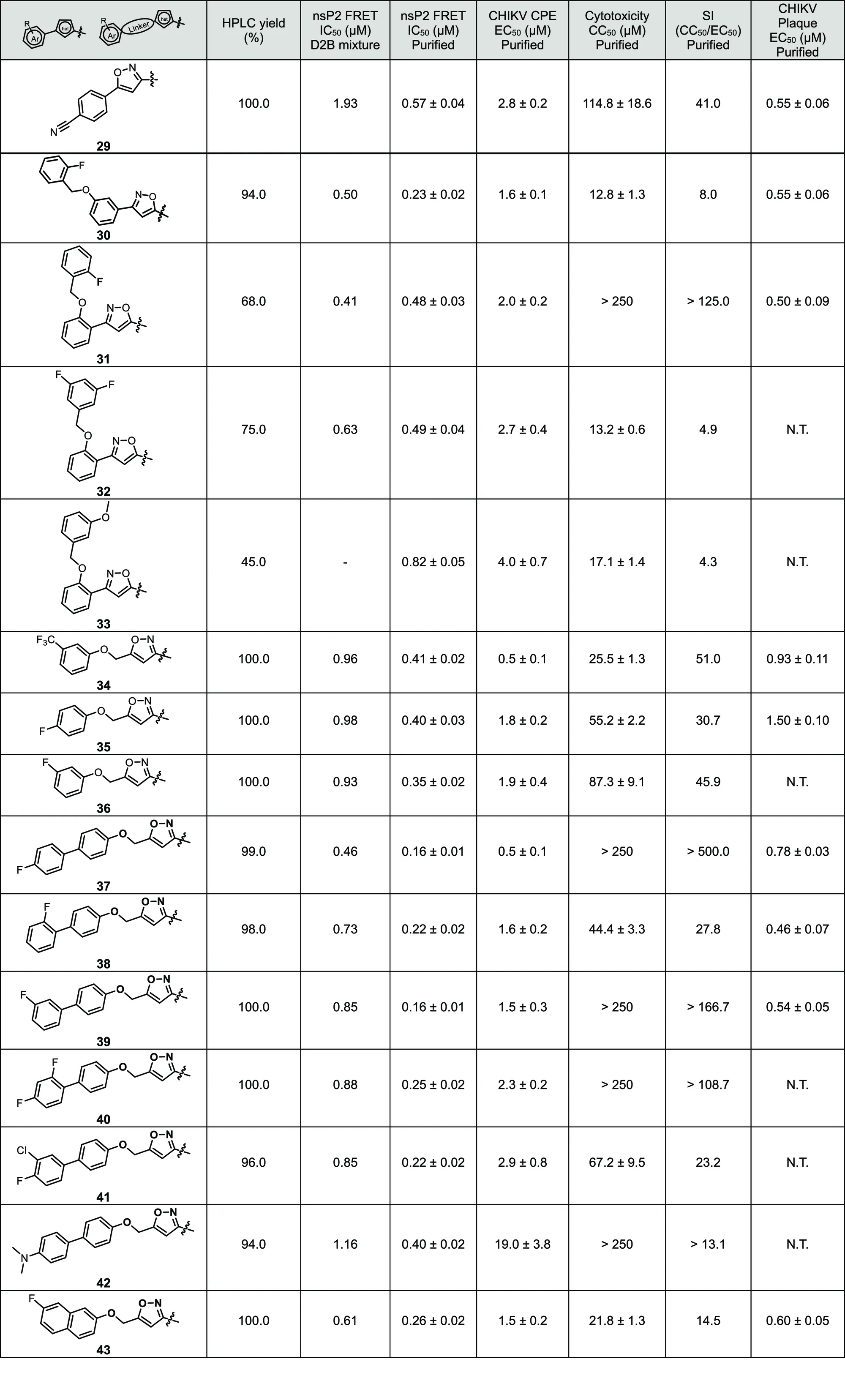

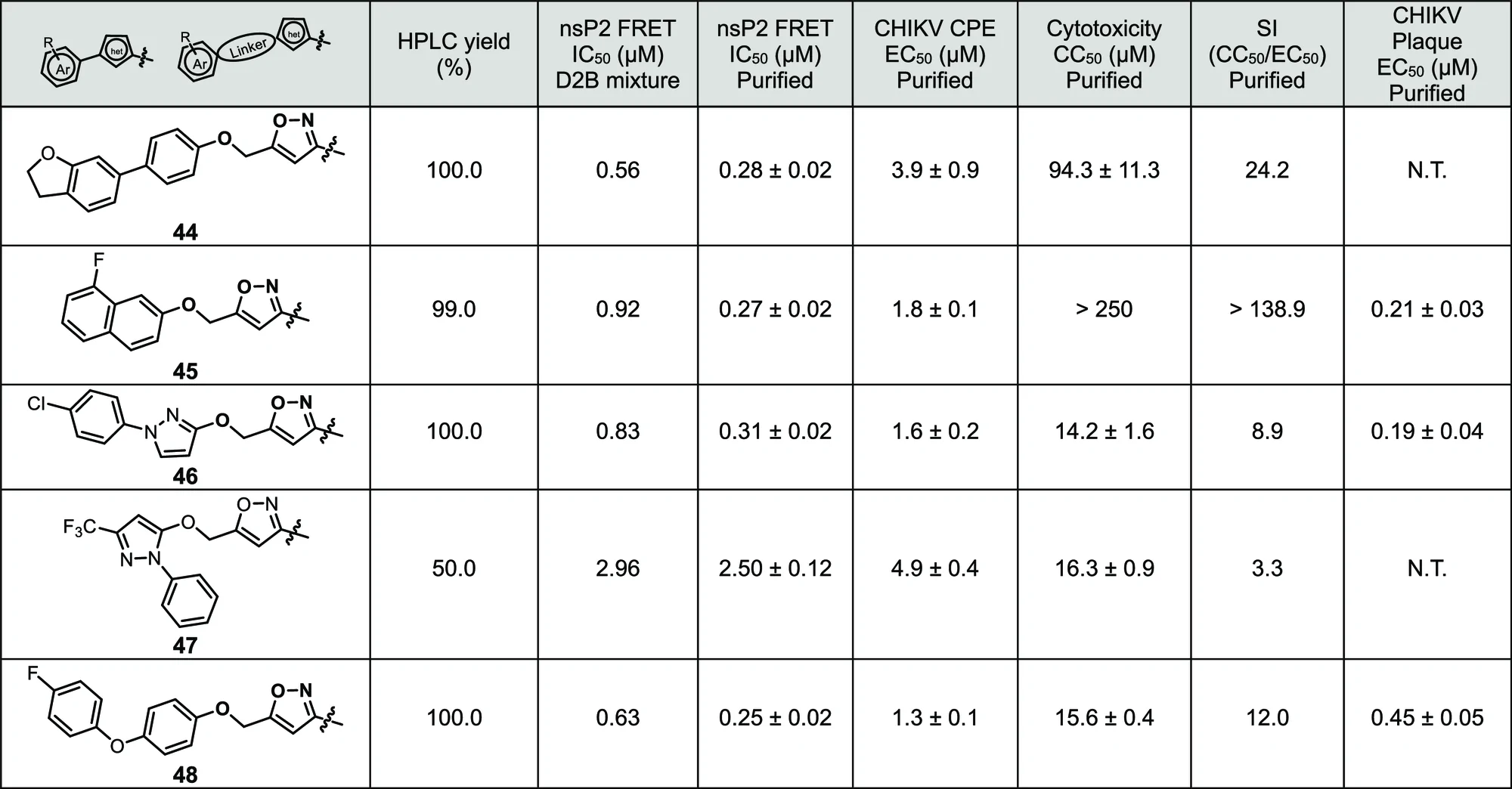
SAR Study of β-Amidomethyl Vinyl Sulfone-Containing CHIKV nsp2 Inhibitors^a^

a For the IC_50_ obtained from FRET assays, the plotted values are the mean ± SD (*n* = 2). For EC_50_ obtained from CPE assays and CC_50_ from cytotoxicity assays, the plotted values are the mean ± SD (*n* = 3). For EC_50_ obtained from plaque the plotted values are the mean ± SD (*n* = 2). N.T. = not tested.

### Synthesis of nsP2 Inhibitors and Structure–Activity Relationship Studies

The synthetic routes and general structures of the target compounds are shown in Scheme 1. Except for the listed ones, other starting carboxylic acids were purchased from commercial suppliers and used without further purification. Intermediates **3** were synthesized from 1-(2-hydroxyphenyl)­ethan-1-one or 1-(3-hydroxyphenyl)­ethan-1-one (**1**) and substituted (bromomethyl)­benzene (**2**). Compounds **3** underwent Claisen condensation with diethyl oxalate to afford the intermediate **4**. Subsequent cyclization of **4** with hydroxylamine hydrochloride or hydrazine hydrate yielded compounds **5** and **5′**, respectively. Hydrolysis of the ester groups in **5** and **5′** with 1 M LiOH, followed by acidification, provided the corresponding carboxylic acids **6** and **6′**. In a parallel route, carboxylic acids **10** were obtained via a Mitsunobu reaction between phenols **7** and **8**, followed by hydrolysis of esters **9** (Scheme 1). The key vinyl sulfone intermediates were synthesized as follows: tert-butyl (2-oxoethyl)­carbamate (**11**) was reacted with diethyl ((methylsulfonyl)­methyl)­phosphonate (**12**) under NaH to afford **13**. Boc deprotection of **13** with 4 M HCl in dioxane gave the amino hydrochloride **14**, which was then coupled with a series of R′–COOH acids to furnish the final vinyl sulfone products (Scheme 1).

**Scheme 1:**
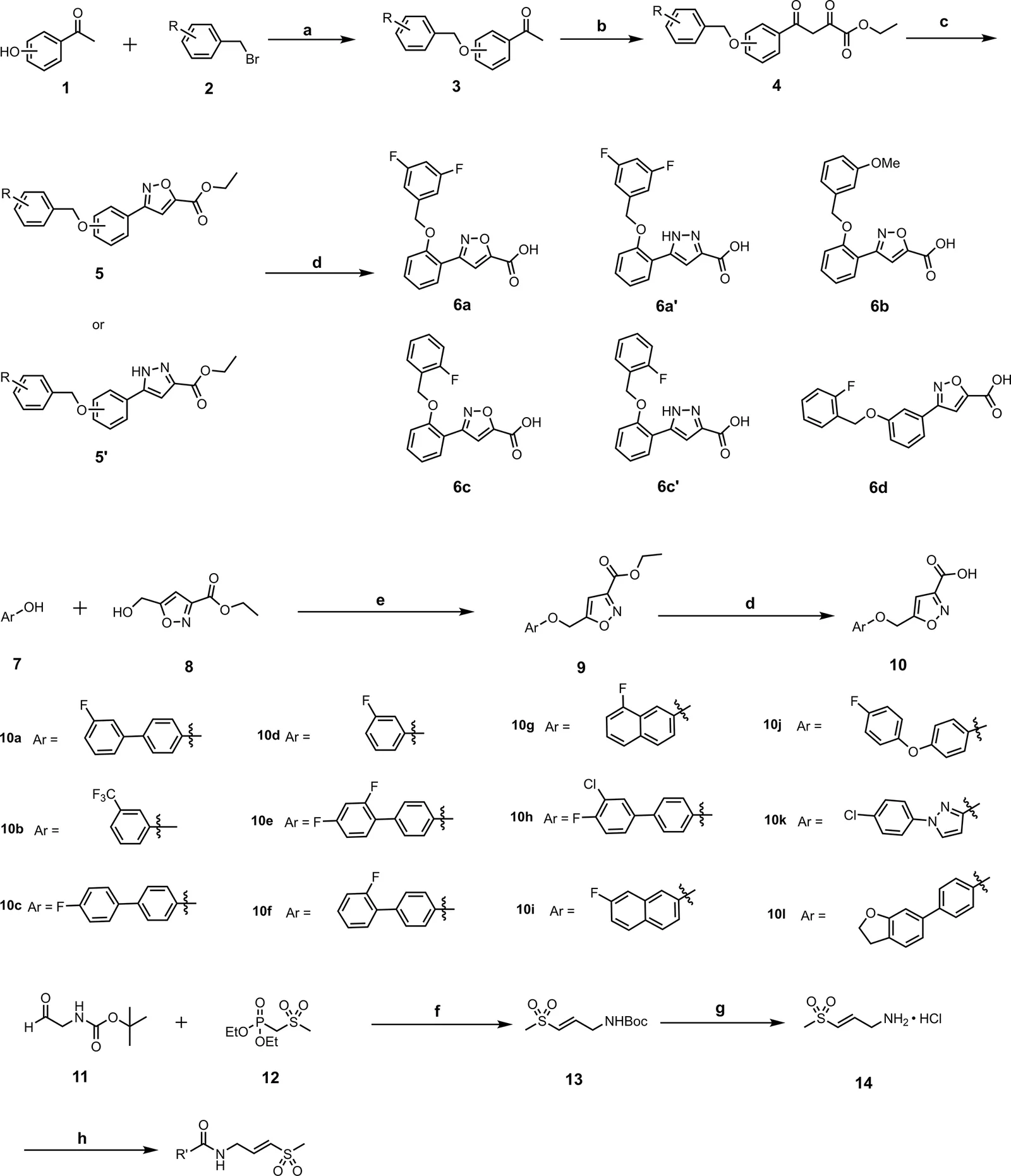
Synthesis of Vinyl Sulfone nsP2 Protease Inhibitors^a^

a Reaction conditions: (a) K_2_CO_3_, DMF, 80 °C, overnight; (b) Diethyl oxalate, EtONa, EtOH, 0 to 25 °C, overnight; (c) NH_2_OH^•^HCl or NH_2_NH_2_^•^H_2_O, EtOH, reflux, 2 h; (d) LiOH, THF/H_2_O, 25 °C, 2 to 3 h; (e) CMBP, Toluene, N_2_, 110 °C, overnight; (f) NaH, THF, 0 °C, 30 min, then 25 °C, 2 h; (g) 4N HCl, DCM, 25 °C, 1 h; (h) R′-COOH, T_3_P, DIPEA, DMF, 0–25 °C, overnight.

In this stage, all the compounds were synthesized, purified, and titrated to determine the inhibitory activity against nsP2 in the FRET assay, the antiviral activity against the CHIKV 181/25 strain in the cytopathic effect (CPE) assay, and the cytotoxicity in the neutral red uptake cell viability assay (Table 1). Compared to the control compounds **RA-0002034** (IC_50_ = 0.41 μM in the FRET assay, EC_50_ = 5.6 μM in the CPE assay) and **Cmp 10** (IC_50_ = 0.35 μM in the FRET assay), several designed analogs showed improved enzymatic inhibition or antiviral activity, including **30** (IC_50_ = 0.23 μM, EC_50_ = 1.6 μM), **34** (IC_50_ = 0.41 μM, EC_50_ = 0.5 μM), **37** (IC_50_ = 0.16 μM, EC_50_ = 0.5 μM), and **45** (IC_50_ = 0.27 μM, EC_50_ = 1.8 μM). These compounds all contain bulkier substitutions designed to fit into the groove between the MTase and protease domains. The SAR analysis indicates that the isoxazole linker is preferred over alternative heterocycles, resulting in improved IC_50_, EC_50_, and selectivity index values (Figure 4). For Type 1 analogs, substitution on the phenyl ring was important, with 3,5-dimethyl, 3-cyano, and 3-methyl groups improving activity, whereas 2-CF_3_, 3-OCF_3_, 3-CF_3_, and 4-OMe substitutions were less favorable. Benzyl ether substitutions were generally tolerated and showed activity comparable to direct phenyl substitution. For Type 2 analogs, substituted bicyclic aromatic groups were favored over monocyclic aromatics, and 2F or 3F substitution further improved activity while reducing cytotoxicity. Collectively, the structure–activity relationship studies validated our design hypothesis (Figure 3).

**Figure 4:**
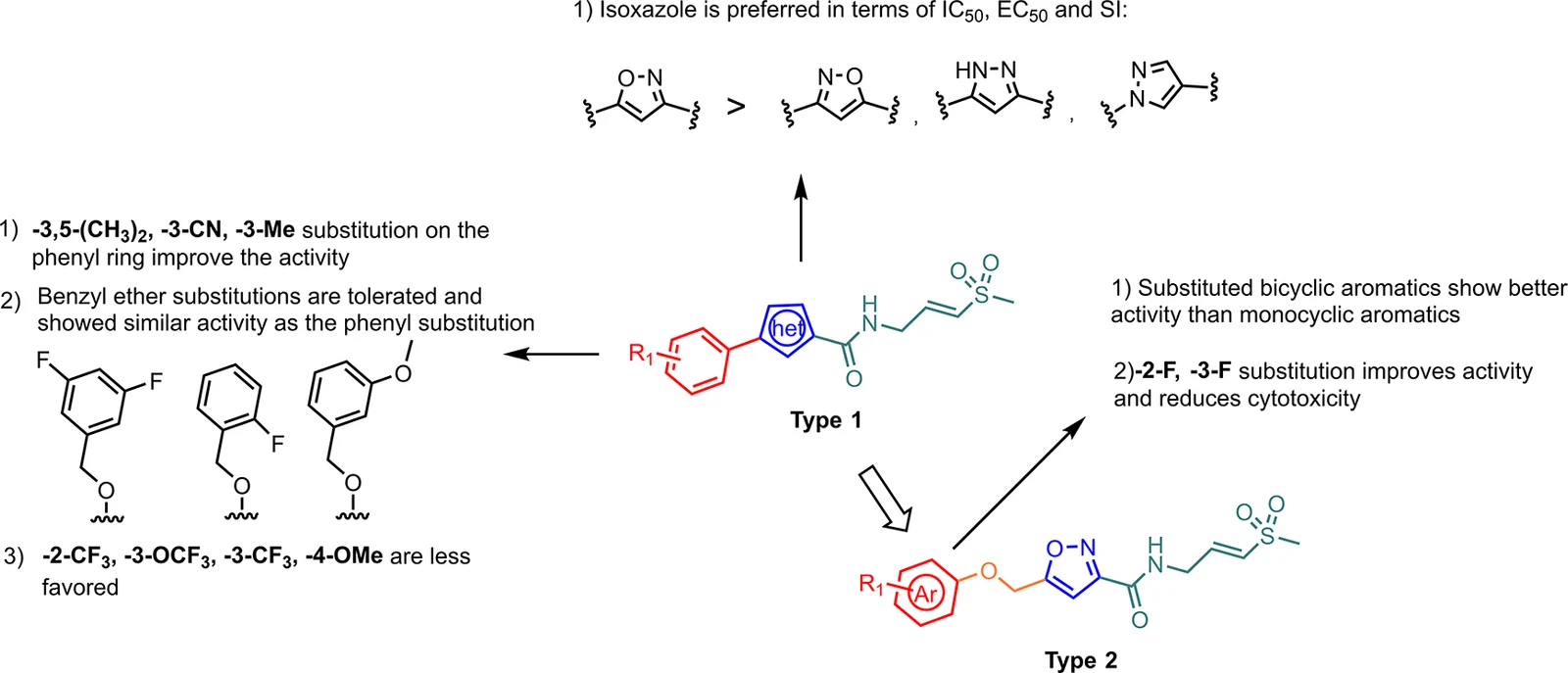
Summary of the structure–activity relationship of nsP2 inhibitors.

### Establishment of Direct-to-Biology (D2B) Platform for the Development of nsP2 Inhibitors

During the development of nsP2 inhibitors, we found that the extensive purification required after routine amide coupling reaction consumed considerable time and resources. Inspired by direct-to-biology high-throughput chemistry approaches,^40,42,43^ we sought to establish a D2B platform for the rapid synthesis and enzymatic evaluation of β-amino vinyl sulfone–containing covalent nsP2 inhibitors using crude reaction mixtures without purification (Figure 5A). Although T3P-mediated amide coupling performed well in our prior large-scale syntheses (millimole) (Scheme 1), the sticky and viscous liquid formed during the reaction made it disadvantageous for small-scale parallel D2B synthesis (micromole). We therefore switched to HATU as the coupling reagent and performed the reaction at the micromole scale in DMF, with a final theoretical concentration of 50 mM (100% yield) (Scheme 2).

**Figure 5:**
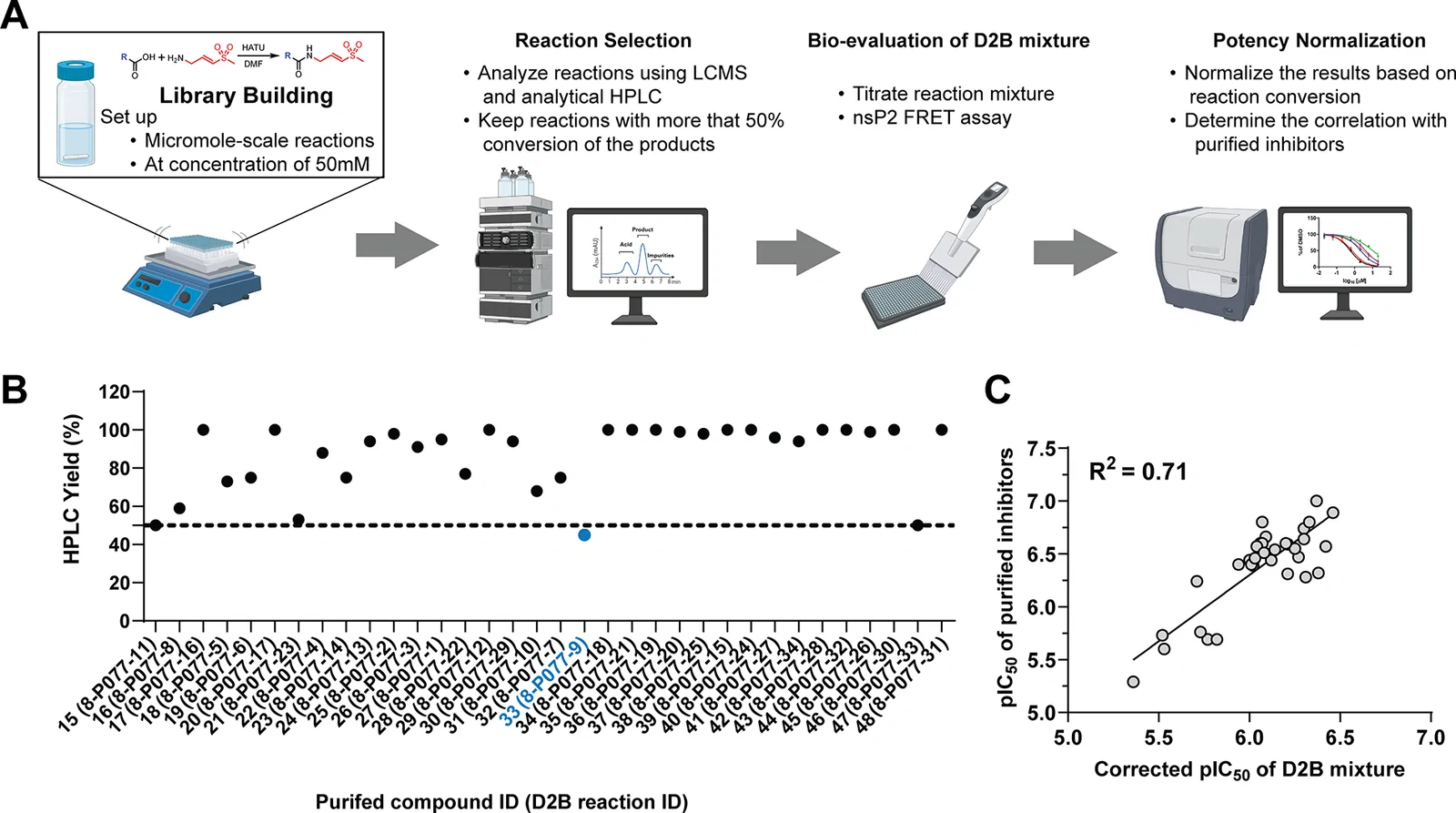
Establishment of D2B platform with β-amidomethyl vinyl sulfone-containing nsP2 inhibitors. (A) Schematic workflow for D2B synthesis, reaction analysis, bioevaluation, and normalization of results (The graph was created with Biorender.com). (B) The HPLC yields D2B reaction mixtures and corresponding purified inhibitors. The reaction with a yield of less than 50% yield was highlighted in blue and excluded from the correlation. (C) The correlation between pIC_50_ values for the D2B reaction mixtures and purified compounds for 33 lead compounds.

**Scheme 2:**
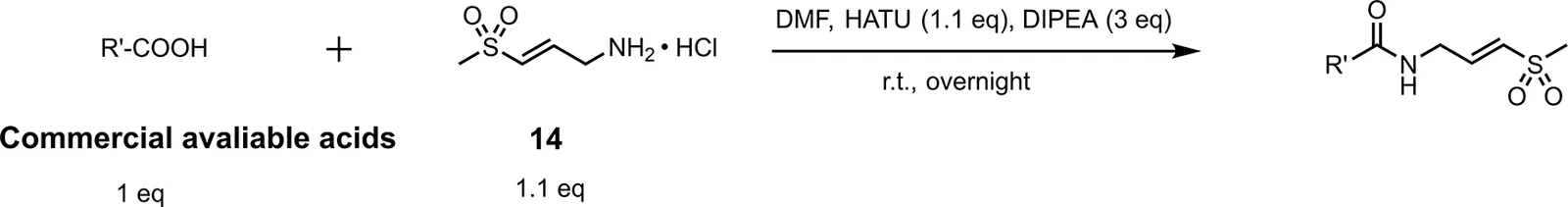
Directly-to-Biology Reaction^a^

a Reaction conditions: HATU, DIPEA, DMF, 25 °C, overnight.

Product formation was confirmed by LC-MS. In several cases, the starting acids and the amide products could not be separated in the LC-MS column with a short elution time (5 min), so we turned to analytical HPLC with a longer column (250 mm) and extended elution time (45 min). Gratifyingly, all amide products exhibited distinct retention times compared to their corresponding acid starting materials. Relative conversion was calculated from the % area of the reaction mixtures relative to the starting materials in HPLC UV traces. Reactions with more than 50% conversion were retained and tested in the nsP2 FRET assay as crude reaction mixtures. For the 34 reported analogs (Table 1), 33 reactions were retained, and the reaction mixture for compound **33** (8-P077–9) was excluded because the % conversion was below 50% (Figure 5B). The relevant low yields for the pyrazole-containing compounds **15** (8-P077–11) (50.0%) and **16** (8-P077–8) (59.0%) might be due to the formation of their respective cyclic byproducts, as reported.^44^ All inhibitory activities (IC_50_ values) obtained in the D2B workflow are normalized to % conversion from HPLC analysis. Pearson correlation analysis was performed using GraphPad Prism 10 (GraphPad Software, San Diego, CA, USA), revealing a strong positive correlation between the two data sets of the D2B reaction mixtures and those of the corresponding purified inhibitors (*r* = 0.8422, 95% CI = 0.7018–0.9196, *P* < 0.0001, *n* = 33), corresponding to an *R* ^2^ value of 0.71. These results highlight the robustness of this D2B platform in the discovery of nsP2 inhibitors (Figure 5C). The corresponding values for pIC_50_ values for the purified inhibitors and D2B reaction mixtures are provided in Table S1.

### Rapid Development of Diverse nsP2 Inhibitors through Virtual Screening and D2B Synthesis

The robust performance of the D2B platform in evaluating nsP2 inhibitors sparked interest in designing nsP2 inhibitors with diverse scaffolds. For this, a reaction-based virtual library was generated and subjected to structure-based covalent virtual screening (SBVS), followed by D2B synthesis and hit evaluation (Figure 6A).

**Figure 6:**
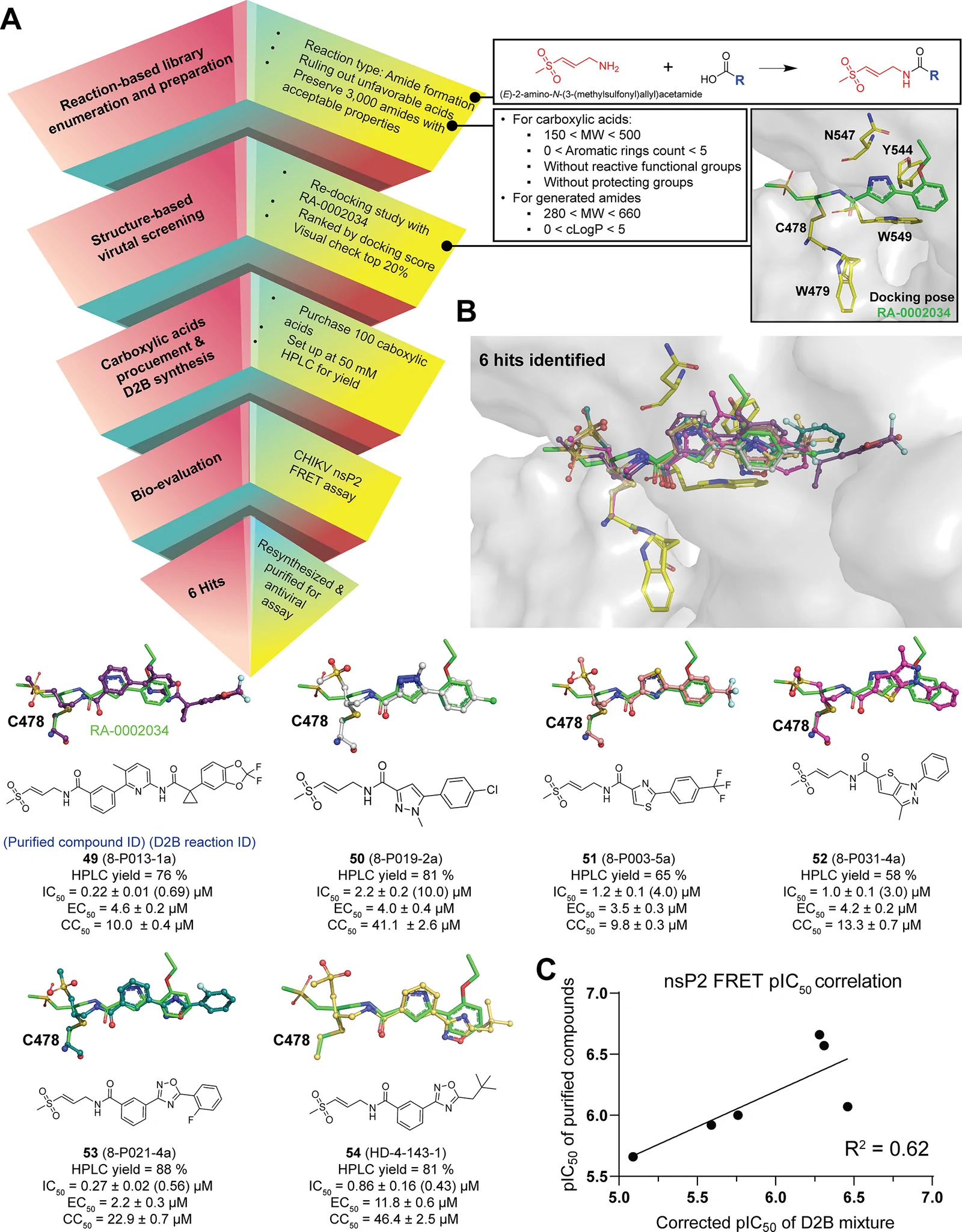
Rapid development of diverse nsP2 inhibitors through SBVS and D2B. (A) Workflow for reaction-based virtual library generation, covalent virtual screening, D2B synthesis, and hit evaluation against CHIKV nsP2. (B) Docking poses of compounds superimposed with **RA-0002034**, along with %HPLC conversion, enzymatic activities of purified compounds and D2B reaction mixtures, antiviral activities against CHIKV 181/25, and cytotoxicity profiles. IC_50_ values from FRET assays are shown as mean ± SD (*n* = 2); EC_50_ from CPE assays and CC_50_ from cytotoxicity assays are mean ± SD (*n* = 3). (C) The correlation of pIC_50_ between D2B reaction mixtures and purified compounds.

To construct the virtual combinatorial library, reactants and the reaction type were defined using the Enumerate Combinatorial Library module in DataWarrior (v06.05.01, OpenMolecules). (E)-2-amino-N-(3-(methylsulfonyl)­allyl)­acetamide, previously used in D2B synthesis, was defined as the amine component, and amide bond formation was selected as the reaction type (Figure 6A). For the acid component, >20,000 commercially available aromatic carboxylic acids were chosen. Molecular properties, including molecular weight (MW) and aromatic ring count, were calculated, and acids were filtered to retain those with MW 150–500 Da and 1–4 aromatic rings. R-group decomposition further excluded acids with reactive or protected functional groups (e.g., aldehydes, Esters, Michael acceptors, Boc-protecting groups, etc.), resulting in ∼3000 carboxylic acids for amide formation. The corresponding virtual amides with MW 280–660 Da and cLogP 0–5 were subjected to covalent virtual screening (Figure 6A). Covalent docking of the known nsP2 inhibitor **RA-0002034** using the DOCK module in MOE confirmed the docking protocol, as the predicted binding pose was consistent with the reported one (Figure 6A).^27^ The same parameters were then applied to the virtual library to predict binding affinities. The docking poses of top-scoring candidates were inspected and filtered. We finally narrowed the list to 100 carboxylic acids and subjected them to D2B amide synthesis using the established reaction condition (Figure 5A). The D2B mixtures were tested in the FRET enzymatic assay, and the IC_50_ values were normalized based on the HPLC conversion.

Six compounds demonstrated single-digit to submicromolar IC_50_ values in the FRET assay, comparable to **RA-0002034** and **Cmp 10**, namely, **8-P013–1a** (D2B reaction mixture ID) or **49** (purified compound ID), **8-P019–2a** (**50**), **8-P003–5a** (**51**), **8-P031–4a** (**52**), **8-P021–4a** (**53**), and **HD-4–143–1** (**54**) (Figure 6B). Unlike **RA-0002034**, which features a pyrazole linker, these SBVS-derived compounds incorporate diverse aromatic scaffolds, including 1-methylpyrazole, thiazole, thieno­[2,3-*c*]­pyrazole, benzene, and 1,2,4-oxadiazole. Analytical HPLC confirmed >50% conversion for all reaction mixtures. Meanwhile, the corresponding compounds were resynthesized and purified to validate the D2B results. A positive correlation was observed between the corrected pIC_50_ values of the D2B reaction mixtures and the pIC_50_ values of the corresponding purified compounds. Pearson correlation analysis performed using GraphPad Prism 10 (GraphPad Software, San Diego, CA, USA) yielded a correlation coefficient of *r* = 0.7878 (95% CI = – 0.066 to 0.976, *n* = 6), corresponding to an *R* ^2^ value of 0.62. Although the correlation did not reach statistical significance (*P* = 0.0628), likely due to the limited sample size, the observed trend supports the ability of the D2B platform to reasonably predict the enzymatic activity of purified compounds (Figure 6C). The corresponding pIC_50_ values for the purified compounds and D2B reaction mixtures are provided in Table S2.

Among these, the most potent compounds, **49** and **53**, exhibited IC_50_ values of 0.22 μM and 0.27 μM, respectively, surpassing **RA-0002034** (0.41 μM) and **Cmp 10** (0.35 μM). Purified compounds were also evaluated in a CPE assay for antiviral activity and in a neutral red uptake assay for cytotoxicity. The most potent hit, **53**, showed improved antiviral potency (EC_50_ = 2.2 μM) compared to **RA-0002034** (EC_50_ = 5.6 μM).

### Determination of k_inact_/K_i_ of Representative nsP2 Inhibitors

Because these internal vinyl sulfone-containing compounds are covalent inhibitors of nsP2, we further characterized their enzymatic potency by comparing their covalent inhibition efficiencies, expressed as k_inact_/K_i_ values. Time-dependent inhibition was measured using a FRET-based enzymatic assay. Briefly, nsP2 protease was preincubated with inhibitors at multiple concentrations for defined time intervals before the addition of the fluorogenic peptide substrate.^45^ Residual enzymatic activity was determined from the initial rate of fluorescence increase and normalized to DMSO-treated controls. The time-dependent decrease in activity was fitted to obtain k_obs_ values at each inhibitor concentration, which were then used to determine k_inact_/K_i_.

Using this approach, **22, 25, 37, 39,** and **53** showed covalent inhibition efficiencies comparable to or modestly improved over the positive control **Cmp 10**. Specifically, **22, 25, 37, 39,** and **53** had k_inact_/K_i_ values of 2,699, 4,786, 3,850, 3,388, and 2,514 M^–1^ s^–1^, respectively, compared with 2,215 M^–1^ s^–1^ for **Cmp 10** (Figure 7). In contrast, **45** showed reduced activity, with a k_inact_/K_i_ value of 1,621 M^–1^ s^–1^. These results indicate that optimizing the substrate-recognition component can yield nsP2 covalent inhibitors with kinetic potency comparable to or greater than that of the current control compound.

**Figure 7:**
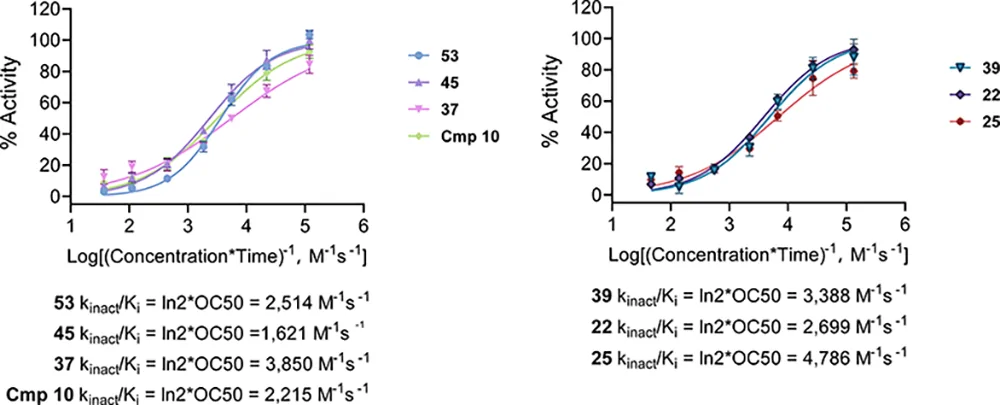
Diagonal dose–response time–course curves (DRTC) with global fitting. Each data point is the mean of four technical replicates.

#### Plaque and Cytopathic Effect Assays Validate the Broad-Spectrum Antiviral Activity of nsP2 Inhibitors against the Semliki Forest Serocomplex Alphaviruses

Plaque assay was performed as a secondary assay to validate the antiviral activity of the lead compounds from our SAR studies and the two top compounds identified through the SBVS–D2B workflow. For the lead compounds derived from **RA-0002034** and **Cmp 10**, 15 representative analogs were selected based on FRET IC_50_values, CPE EC_50_ values, and selectivity indices (SI = CC_50_/EC_50_). Results are summarized in Table 1. Overall, compounds **35**, **26**, and **34** exhibited modest improvements in EC_50_ (less than 2-fold) compared to **RA-0002034** (Figure S2). Notably, **27**, **48**, and **46** demonstrated potent antiviral activity, with 6- to 10-fold improvements relative to **RA-0002034** (Figure S2). The antiviral activities of the two top compounds, **49** and **53**, were also evaluated in plaque assays and compared with those of **RA-0002034** and **Cmp10**. For **49**, despite modest improvements in EC_50_ (less than 2-fold) compared to **RA-0002034**, complete plaque reduction was achieved at 5 μM. It exhibited slightly increased potency compared to **Cmp10** (Figure 8A). Notably, **53** displayed enhanced inhibition (about 4-fold to **RA-0002034** and about 3-fold to **Cmp10**) of plaque formation, highlighting its potential as a promising starting point for the development of nsP2 protease inhibitors against CHIKV (Figure 8A).

**Figure 8:**
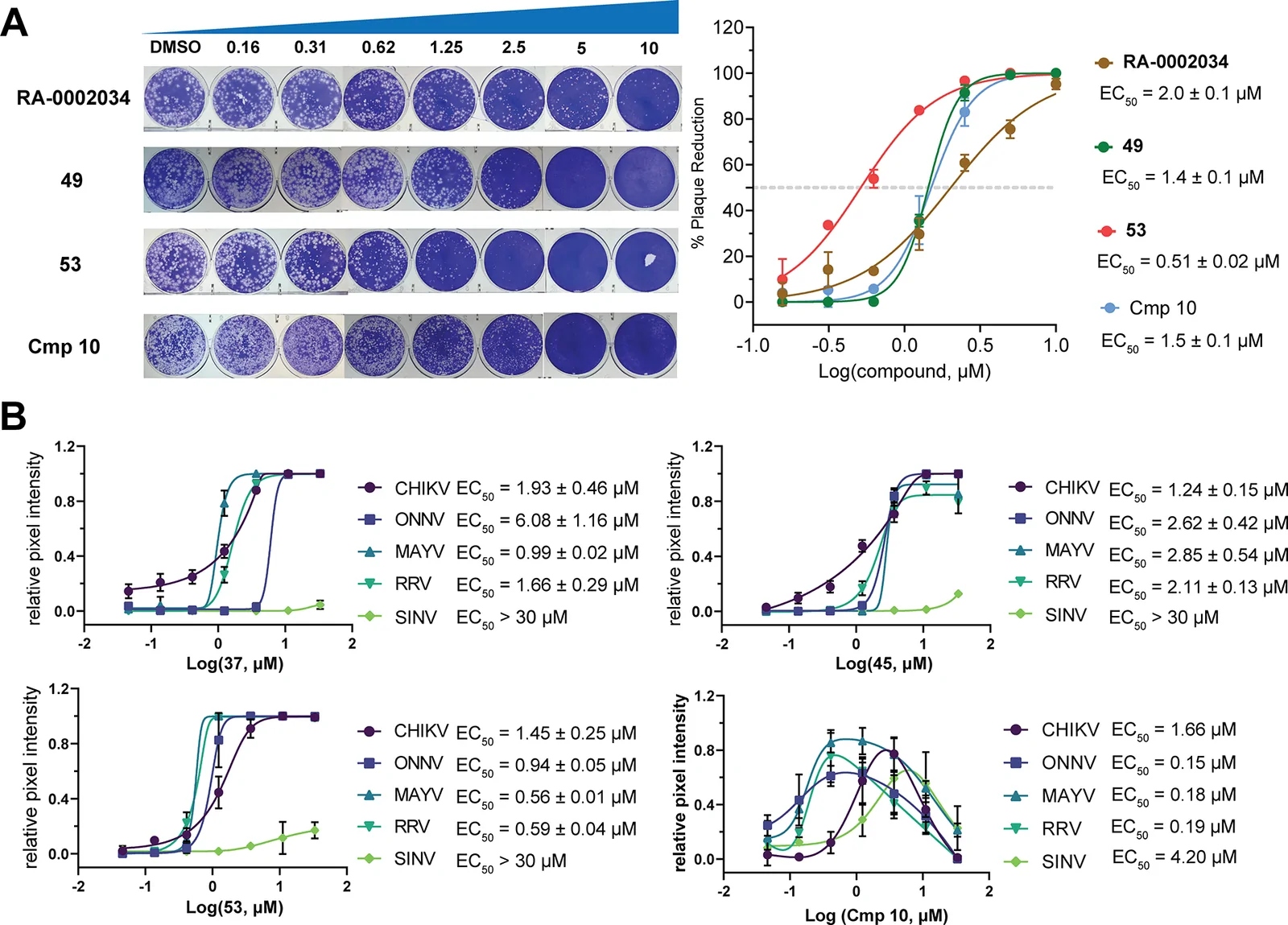
Antiviral activities of potent inhibitors against alphaviruses. (A) Plaque assay results of two representative compounds in inhibiting the CHIKV 181/25 strain. Representative images from two independent experiments are shown. Quantified activities are reported as mean ± SD (*n* = 2). (B) Cytopathic effect assay results demonstrate the broad-spectrum anti-Semliki Forest serocomplex alphavirus activity of potent compounds. Quantified activities are reported as mean ± SD (*n* = 3). The belt curves for the control **Cmp 10** are due to cytotoxicity at higher drug concentrations.

The broad-spectrum antiviral activities of the two lead compounds with favorable SI, **37** and **45**, along with the most potent hit, **53**, and control compound, **Cmp10**, were further evaluated against multiple alphaviruses within the Semliki Forest serocomplex, including o’nyong–nyong virus (ONNV), Mayaro virus (MAYV), and Ross River virus (RRV), as well as Sindbis virus (SINV), which belongs to the genetically distinct Western Equine Encephalitis serocomplex.^7,14^ **37, 45**, and **53** exhibited single-digit micromolar EC_50_ values against CHIKV, none of our inhibitors showed measurable activity against SINV at up to 30 μM (Figure 8B). Sequence alignment of the nsP2 protease domains from CHIKV, ONNV, MAYV, RRV, and SINV revealed a seven–amino acid insertion (DSARPVA) within the flexible loop (residues 543–548, ADNHW) at the drug-binding site of SINV (Figure S3). This insertion likely perturbs inhibitor binding, consistent with the observed phenotypic drug resistance. **37** displayed enhanced antiviral potency against MAYV (EC_50_ = 0.99 μM) and RRV (EC_50_ = 1.66 μM) but moderate activity against ONNV (EC_50_ = 6.08 μM). **45** showed consistent inhibition of CHIKV, ONNV, MAYV, and RRV, with EC_50_ values ranging from 1.24 to 2.85 μM. Notably, **53** demonstrated more potent inhibition against ONNV (EC_50_ = 0.94 μM), MAYV (EC_50_ = 0.56 μM), and RRV (EC_50_ = 0.59 μM), suggesting it is a promising hit for further optimization. **Cmp10**, used as a control, exhibited significant cytotoxicity at higher concentrations, resulting in a bell-shaped dose–response curve that compromised reliable quantification of the EC_50_ values (Figure 8B).

### Molecular Dynamics Simulations and GSH Stability of nsP2 Inhibitor 53 (Jun16229)

To elucidate the mechanism of action of **53**, a comprehensive evaluation integrating molecular dynamics (MD) simulations was conducted. MD analysis of covalent inhibitors in their noncovalent preassociation complexes has been reported as an effective approach for modeling the transient intermediate state preceding covalent bond formation between electrophilic warheads and nucleophilic residues.^46^ In this study, the stability of the **53**–CHIKV nsP2 noncovalent complex was assessed through 200 ns MD simulations.

The results revealed that **53** forms a stable complex with nsP2 over the 200 ns simulation, maintained by specific intermolecular interactions (Figure 9A). The amide NH forms a hydrogen bond with Asn547, while the carbonyl oxygen interacts with Trp549—in contrast to the Trp479 interaction observed in the **RA-0002034**/nsP2 complex. Two Pi–Pi stacking interactions were also observed during the simulation: one between the phenyl core and Tyr544, and the other between Trp549 and the oxadiazole ring. Other residues, including Ala511and Tyr544, contribute favorable hydrophobic interactions stabilizing the complex (Figure 9A).

**Figure 9:**
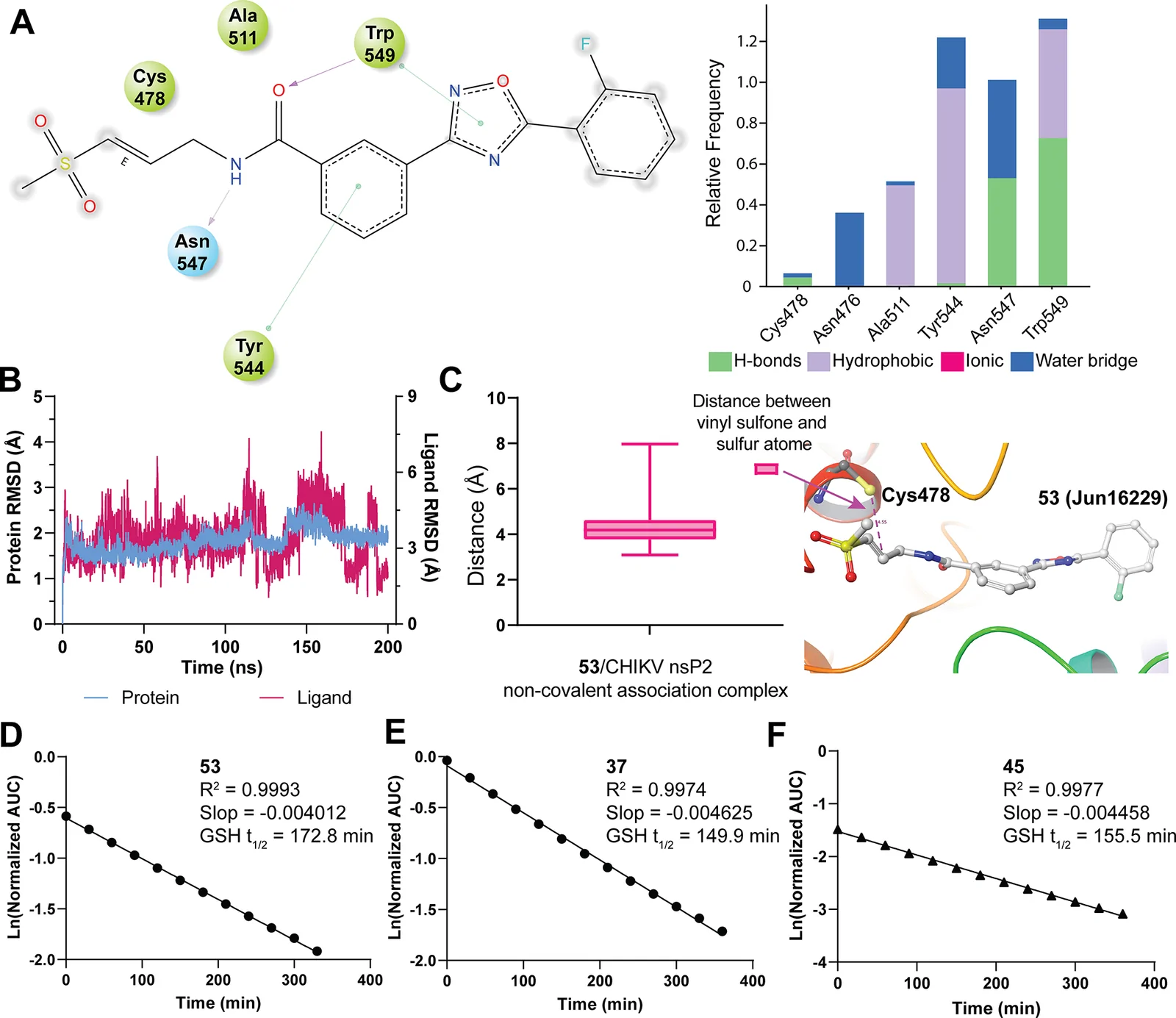
MD-Based assessment of **53** in noncovalent association complex with nsP2 and the LC-MS-based GSH reactivity assay of covalent engagement. (A) Protein–ligand contact and stabilizing interactions inside the binding area of nsP2 with **53**; hydrogen bonding interactions bar is depicted in green, ion hydrogen bonding interactions in magenta, water bridges in blue, and lipophilic interactions in gray. (B) RMSD plots of Cα carbons of nsP2 (blue diagram) and of the heavy atoms of the ligand **53** (red diagram). (C) Boxplots of the distance between the electrophilic carbon atom of the ligand and the sulfur atom of Cys478 measured in the MD simulation. LC-MS-based GSH reactivity assay results for **53** (D), **37** (E), and **45** (F). For each compound. the standard deviation of the single linear regression (R^2^), the slop of the fitted line, and the calculated GSH half-life (GSH t_1/2_) are reported.

Throughout the 200 ns simulation, the RMSD values for the nsP2 backbone remained below 1.5 Å, and for the ligand, it is about 3 Å, indicating conformational stability of the noncovalent complex (Figure 9B). Another critical parameter, the distance between the γ-sulfur atom of Cys478 and the electrophilic carbon of the vinyl sulfone moiety, was monitored (Figure 9C). The average distance of approximately 4 Å over the 200 ns trajectory, comparable to the sum of the generalized Born radii of carbon and sulfur, suggests that the geometric conditions required for covalent bond formation are stably maintained within the **53**–nsP2 complex (Figure 9C).

To assess the GSH stability of **53**, we conducted an LC-MS-based GSH reactivity assay to determine the compound’s GSH t_1/2_. Both depletion of the parent compound and formation of the corresponding GSH adduct were observed. From the natural log plot of **53** AUC values normalized to the internal standard, **53** exhibited a moderate reactivity (GSH t_1/2_ = 172.8 min) comparable to our two lead compounds, **37** (GSH t_1/2_ = 149.9 min) and **45** (GSH t_1/2_ = 155.5 min) (Figure 9D,F), as well as reported inhibitor, **RA-0002034** (GSH t_1/2_ = 130 min).^27^

## Conclusion

Chikungunya, a mosquito-borne viral disease caused by the chikungunya virus (CHIKV), remains a major global public health concern. The infection affects individuals of all ages, including those without underlying health conditions, and has been reported across tropical and subtropical regions worldwide. Despite its high morbidity and expanding geographic distribution, no licensed antiviral therapy is currently available, underscoring the urgent need for novel therapeutic strategies targeting essential viral enzymes.

In this study, we focused on developing covalent inhibitors targeting nsP2, a multifunctional cysteine protease that plays a central role in CHIKV polyprotein processing and viral replication. Building upon the previously reported β-amino vinyl sulfone inhibitor **RA-0002034**,^27^ we employed a structure-based drug design (SBDD) strategy to explore additional binding pockets within the nsP2 active site. This iterative optimization yielded a series of lead compounds exhibiting enhanced enzymatic potency and improved antiviral activity in cell-based assays.

To accelerate lead identification, we established a D2B platform incorporating a FRET-based enzymatic assay, which enabled rapid evaluation of unpurified reaction mixtures. A strong correlation (*R* ^2^ = 0.71) between the enzymatic inhibitory activities of 33 lead compounds identified in our structure–activity relationship (SAR) study and their corresponding D2B mixtures validated the reliability and predictive power of this approach for nsP2 inhibitor development.

Finally, through the integration of reaction-based virtual library enumeration, structure-guided covalent virtual screening, D2B synthesis and testing, and cell-based antiviral assays, we identified several promising covalent nsP2 inhibitors featuring diverse scaffolds. These compounds demonstrated broad-spectrum antiviral activity against multiple alphaviruses, except SINV. Sequence alignment of the protease domain of nsP2 from CHIKV, ONNV, MAYV, RRV, and SINV revealed a 7-amino acid insertion in the flexible loop of SINV, which is located at the drug-binding site.

In summary, this work establishes a rapid, purification-free platform for the development of covalent nsP2 inhibitors with activity against CHIKV and related alphaviruses. By uniting covalent virtual screening with direct-to-biology synthesis and enzymatic testing, we demonstrate the feasibility of compressing the antiviral lead identification process from months to weeks. The most potent hit, **53**, showed submicromolar enzymatic inhibition and broad-spectrum antiviral activity across the Semliki Forest serocomplex, identifying a promising scaffold for further optimization. Going forward, efforts to define the *in vivo* pharmacokinetics, resistance profile, and warhead selectivity of these molecules will advance their translational potential. More broadly, this workflow highlights how covalent inhibitor design, when paired with D2B evaluation, can accelerate antiviral development against genetically diverse and emerging viral targets.

## Experimental Section

### General Information

Solvents and commercially available building blocks were purchased from suppliers and used without purification. All reactions were monitored by thin-layer chromatography (TLC), visualizing under ultraviolet light (254 nm), or liquid chromatography–mass spectrometry (LC-MS). Column chromatography purification was performed by using the CombiFlash^@^ NextGen 300+ system. High-performance liquid chromatography purification was performed by using the ACCQPrep HP150 system. The ESI-MS readings were recorded on an Agilent MSD iQ G6160A mass spectrometer. ^1^H NMR and ^13^C NMR spectra were recorded on a Bruker AV-400 spectrometer at 400 and 100 MHz, respectively. Coupling constants (J) are expressed in hertz (Hz). NMR data is analyzed with MestReNova (14.1.0). Chemical shifts (δ) of NMR are reported in parts per million (ppm) units. High-resolution mass spectra were recorded on a Q-TOF Premier mass spectrometer. The purity of compounds was determined to be over 95% by reverse-phase HPLC analysis. All compounds were characterized by proton and carbon NMR and MS. **Cmp 10** (SGCNSP2PRO-1) was provided by the Structural Genomics Consortium (https://www.thesgc.org/chemical-probes/sgc-nsp2pro-1).

### General Procedure of Intermediate Acids 6 and 6’

1-(2-hydroxyphenyl)­ethan-1-one or 1-(3-hydroxyphenyl)­ethan-1-one (**1**) (1 eq), substituted (bromomethyl)­benzene **2** (2 eq), and K2CO3 (2 eq) were dissolved in anhydrous DMF, and the reaction mixture was stirred at 80 °C for overnight. After finishing, the mixture was added H_2_O and EtOAc; the organic layer was separated, washed with brine, dried over Na_2_SO_4_, and concentrated. The residue was primarily purified by column chromatography to get the intermediate 3. Three (1 eq) and diethyl oxalate (2 eq) were dissolved in anhydrous EtOH, the reaction mixture was then degassed and filled with N_2_. Sodium ethoxide (2 eq) was slowly added to the mixture, and the reaction mixture was stirred at ambient temperature overnight. After completion, the solid was filtered and washed with ethanol for 2 times, then the solution was removed under vacuum to get the crude product **4**. **4** (1 eq) was dissolved in EtOH, then NH_2_OH^•^HCl (8 eq) or NH_2_NH_2_^•^H_2_O (8 eq) was added, and the reaction mixture was refluxed for 2 h. When the reaction finished, the solution was removed under vacuum to get the residue. Next, the residue was dissolved in THF/H_2_O (3:1), and LiOH (3 eq) was added to the mixture, which was stirred for 3 h. After the aster was fully hydrolyzed, THF was removed under vacuum, then water was added to dissolve the residue, and 1 N HCl was added to acidify. The precipitate was filtered, washed with water, and dried in vacuum to get the acids **6** or **6’**.

#### 3,(5-((Difluorobenzyl)­oxy)­phenyl)­isoxazole-5-carboxylic Acid (**6a**)

Off-white solid (26% yield for 4 steps). ^1^H NMR (400 MHz, DMSO-*d*_6_): δ 14.03 (s, 1H), 8.03–7.88 (m, 1H), 7.59–7.50 (m, 1H), 7.30 (d, *J* = 8.5 Hz, 1H), 7.28–7.22 (m, 3H), 7.17 (t, *J* = 7.6 Hz, 1H), 7.09 (s, 1H), 5.38 (s, 2H). C_17_H_11_F_2_NO_4_, MS calcd for *m*/*z* [M + H]^+^: 332.1 (calculated), 332.2 (found).

#### 3-(2-((3-Methoxybenzyl)­oxy)­phenyl)­isoxazole-5-carboxylic Acid (**6b**)

Yellow white solid (19% yield for 4 steps). ^1^H NMR (400 MHz, DMSO-*d*_6_): δ 14.04 (s, 1H), 7.95 (d, *J* = 7.5 Hz, 1H), 7.54 (t, *J* = 8.0 Hz, 1H), 7.35 (d, *J* = 8.1 Hz, 2H), 7.26–7.02 (m, 4H), 6.94 (d, *J* = 8.1 Hz, 1H), 5.33 (s, 2H), 3.78 (s, 3H). C_18_H_15_NO_5_, MS calcd for *m*/*z* [M + H]^+^: 326.1 (calculated), 326.2 (found).

#### 3-(2-((2-Fluorobenzyl)­oxy)­phenyl)­isoxazole-5-carboxylic Acid (**6c**)

Yellow white solid (25% yield for 4 steps). ^1^H NMR (400 MHz, DMSO-*d*_6_): δ 13.95 (s, 1H), 7.95 (d, *J* = 7.8 Hz, 1H), 7.59 (dt, *J* = 25.1, 7.6 Hz, 2H), 7.51–7.39 (m, 2H), 7.30 (dt, *J* = 14.6, 8.5 Hz, 2H), 7.18 (t, *J* = 7.6 Hz, 1H), 6.62 (s, 1H), 5.40 (s, 2H). C_17_H_12_FNO_4_, MS calcd for *m*/*z* [M + H]^+^: 314.1 (calculated), 314.1 (found).

#### 3-(3-((2-Fluorobenzyl)­oxy)­phenyl)­isoxazole-5-carboxylic Acid (**6d**)

Yellow white solid (21% yield for 4 steps). ^1^H NMR (400 MHz, DMSO-*d*_6_): δ 7.67–7.58 (m, 2H), 7.56 (d, *J* = 7.7 Hz, 1H), 7.52–7.40 (m, 3H), 7.27 (q, *J* = 8.0, 7.5 Hz, 2H), 7.21 (dd, *J* = 8.1, 2.5 Hz, 1H), 5.25 (s, 2H). C_17_H_12_FNO_4_, MS calcd for *m*/*z* [M + H]^+^: 314.1 (calculated), 314.2 (found).

#### 5-(2-((3,5-Difluorobenzyl)­oxy)­phenyl)-1H-pyrazole-3-carboxylic Acid (**6a′**)

Yellow white solid (19% yield for 4 steps). ^1^H NMR (400 MHz, DMSO-*d*_6_): δ 13.32 (s, 1H), 7.83 (d, *J* = 7.7 Hz, 1H), 7.36 (t, *J* = 7.8 Hz, 1H), 7.25–7.16 (m, 4H), 7.15 (s, 1H), 7.08 (t, *J* = 7.5 Hz, 1H), 5.31 (s, 2H). C_17_H_12_F_2_N_2_O_3_, MS calcd for *m*/*z* [M + H]^+^: 331.1 (calculated), 331.2 (found).

#### 5-(2-((2-Fluorobenzyl)­oxy)­phenyl)-1H-pyrazole-3-carboxylic Acid (**6c′**)

Off white solid (16% yield for 4 steps).^1^H NMR (400 MHz, DMSO-*d*_6_): δ 13.27 (s, 1H), 7.85 (d, *J* = 7.7 Hz, 1H), 7.58 (t, *J* = 7.7 Hz, 1H), 7.44 (d, *J* = 7.0 Hz, 1H), 7.35 (d, *J* = 7.7 Hz, 1H), 7.27 (q, *J* = 8.9, 7.4 Hz, 3H), 7.08 (d, *J* = 11.0 Hz, 2H), 5.32 (s, 2H). C_17_H_13_FN_2_O_3_, MS calcd for *m*/*z* [M + H]^+^: 313.1 (calculated), 313.2 (found).

### General Procedure of Intermediate Acids 10

Substituted aromatic phenol **7** (1.2 eq), ethyl 5-(hydroxymethyl)­isoxazole-3-carboxylate **8** (1.0 eq), and CMBP (1.8 eq) were dissolved in anhydrous toluene. The reaction was degassed and filled with N_2_, then the reaction mixture was stirred at 110 °C for overnight. After completion, the solvent was removed under vacuum to yield crude residue **9**. Residue **9** was dissolved in the THF/H_2_O, then 3 eq LiOH was added, and the mixture was stirred at rt for 2 h. Once the ester was hydrolyzed completely, the organic solvent was removed, and water was added to dissolve the residue. One N HCl was added to acidify, the precipitate was filtered, washed with water, and dried in a vacuum to get the acids **10**.

#### 5-(((3′-Fluoro-[1,1′-biphenyl]-4 yl)­oxy)­methyl)­isoxazole-3-carboxylic Acid (**10a**)

Yellow white solid (58% yield for 2 steps). ^1^H NMR (400 MHz, DMSO-*d*_6_): δ 7.69 (d, *J* = 8.5 Hz, 2H), 7.53–7.45 (m, 3H), 7.17 (dd, *J* = 7.5, 5.1 Hz, 3H), 6.97 (s, 1H), 5.42 (s, 2H). C_17_H_12_FNO_4_, MS calcd for *m*/*z* [M + H]^+^: 314.1 (calculated), 314.2 (found).

#### 5-((3-(Trifluoromethyl)­phenoxy)­methyl)­isoxazole-3-carboxylic Acid (**10b**)

Yellow white solid (45% yield for 2 steps). ^1^H NMR (400 MHz, DMSO-*d*_6_): δ 7.58 (t, *J* = 7.9 Hz, 1H), 7.45–7.33 (m, 3H), 7.00 (s, 1H), 5.48 (s, 2H). C_12_H_8_F_3_NO_4_, MS calcd for *m*/*z* [M + H]^+^: 288.0 (calculated), 288.1 (found).

#### 5-(((4′-Fluoro-[1,1′-biphenyl]-4 yl)­oxy)­methyl)­isoxazole-3-carboxylic Acid (**10c**)

Off white solid (48% yield for 2 steps). ^1^H NMR (400 MHz, DMSO-*d*_6_): δ 7.70–7.64 (m, 2H), 7.64–7.59 (m, 2H), 7.26 (t, *J* = 8.9 Hz, 2H), 7.19–7.12 (m, 2H), 6.99 (s, 1H), 5.41 (s, 2H). C_17_H_12_FNO_4_, MS calcd for *m*/*z* [M + H]^+^: 314.1 (calculated), 314.1 (found).

#### 5-((3-Fluorophenoxy)­methyl)­isoxazole-3-carboxylic Acid (**10d**)

Yellow white solid (62% yield for 2 steps). ^1^H NMR (400 MHz, DMSO-*d*_6_): δ 7.36 (q, *J* = 8.0 Hz, 1H), 7.00 (d, *J* = 9.4 Hz, 2H), 6.92 (dd, *J* = 8.3, 2.4 Hz, 1H), 6.84 (td, *J* = 8.5, 2.5 Hz, 1H), 5.39 (s, 2H). C_11_H_8_FNO_4_, MS calcd for *m*/*z* [M + H]^+^: 238.0 (calculated), 238.1 (found).

#### 5-(((2′,4′-Difluoro-[1,1′-biphenyl]-4 yl)­oxy)­methyl)­isoxazole-3-carboxylic Acid (**10e**)

Yellow white solid (65% yield for 2 steps). ^1^H NMR (400 MHz, DMSO-*d*_6_): δ 7.56 (td, *J* = 9.0, 6.6 Hz, 1H), 7.50 (d, *J* = 8.2 Hz, 2H), 7.34 (ddd, *J* = 11.5, 9.2, 2.6 Hz, 1H), 7.23–7.14 (m, 3H), 6.99 (s, 1H), 5.42 (s, 2H). C_17_H_11_F_2_NO_4_, MS calcd for *m*/*z* [M + H]^+^: 332.0 (calculated), 332.1 (found).

#### 5-(((2′-Fluoro-[1,1′-biphenyl]-4 yl)­oxy)­methyl)­isoxazole-3-carboxylic Acid (**10f**)

Yellow white solid (58% yield for 2 steps). ^1^H NMR (400 MHz, DMSO-*d*_6_): δ 7.57–7.48 (m, 3H), 7.44–7.35 (m, 1H), 7.33–7.25 (m, 2H), 7.18 (d, *J* = 8.5 Hz, 2H), 7.00 (s, 1H), 5.42 (s, 2H). C_17_H_12_FNO_4_, MS calcd for *m*/*z* [M + H]^+^: 314.1 (calculated), 314.1 (found).

#### 5-(((8-Fluoronaphthalen-2-yl)­oxy)­methyl)­isoxazole-3-carboxylic Acid (**10g**)

Yellow white solid (53% yield for 2 steps). ^1^H NMR (400 MHz, DMSO-*d*_6_): δ 7.99 (dd, *J* = 9.1, 1.8 Hz, 1H), 7.74 (d, *J* = 7.9 Hz, 1H), 7.53 (d, *J* = 2.6 Hz, 1H), 7.36 (ddd, *J* = 16.2, 11.3, 7.0 Hz, 3H), 7.01 (s, 1H), 5.55 (s, 2H). C_15_H_10_FNO_4_, MS calcd for *m*/*z* [M + H]^+^: 288.1 (calculated), 288.2 (found).

#### 5-(((3′-Chloro-4′-fluoro-[1,1′-biphenyl]-4 yl)­oxy)­methyl)­isoxazole-3-carboxylic Acid (**10h**)

Yellow white solid (72% yield for 2 steps). ^1^H NMR (400 MHz, DMSO-*d*_6_): δ 7.85 (dd, *J* = 7.1, 2.4 Hz, 1H), 7.72–7.62 (m, 3H), 7.47 (t, *J* = 9.0 Hz, 1H), 7.16 (d, *J* = 8.5 Hz, 2H), 6.99 (s, 1H), 5.42 (s, 2H). C_17_H_11_ClFNO_4_, MS calcd for *m*/*z* [M + H]^+^: 348.0 (calculated), 348.1 (found).

#### 5-(((7-Fluoronaphthalen-2-yl)­oxy)­methyl)­isoxazole-3-carboxylic Acid (**10i**)

Yellow white solid (56% yield for 2 steps). ^1^H NMR (400 MHz, DMSO-*d*_6_): δ 7.98–7.85 (m, 2H), 7.60 (dd, *J* = 10.6, 2.6 Hz, 1H), 7.51 (d, *J* = 2.6 Hz, 1H), 7.27 (ddd, *J* = 21.8, 8.9, 2.6 Hz, 2H), 7.04 (s, 1H), 5.48 (s, 2H). C_15_H_10_FNO_4_, MS calcd for *m*/*z* [M + H]^+^: 288.1 (calculated), 288.2 (found).

#### 5-((4-(4-Fluorophenoxy)­phenoxy)­methyl)­isoxazole-3-carboxylic Acid (**10j**)

Yellow white solid (46% yield for 2 steps). ^1^H NMR (400 MHz, DMSO-*d*_6_): δ 7.20 (t, *J* = 8.7 Hz, 2H), 7.09 (d, *J* = 8.8 Hz, 2H), 7.03–6.94 (m, 5H), 5.34 (s, 2H). C_17_H_12_FNO_5_, MS calcd for *m*/*z* [M + H]^+^: 330.1 (calculated), 330.2 (found).

#### 5-(((1-(4-Chlorophenyl)-1H-pyrazol-3-yl)­oxy)­methyl)­isoxazole-3-carboxylic Acid (**10k**)

Yellow white solid (65% yield for 2 steps). ^1^H NMR (400 MHz, DMSO-*d*_6_): δ 8.42 (d, *J* = 2.7 Hz, 1H), 7.78 (d, *J* = 8.7 Hz, 2H), 7.53 (d, *J* = 8.8 Hz, 2H), 6.97 (s, 1H), 6.17 (d, *J* = 2.7 Hz, 1H), 5.49 (s, 2H). C_14_H_10_ClN_3_O_4_, MS calcd for *m*/*z* [M + H]^+^: 320.0 (calculated), 320.1 (found).

#### 5-((4-(2,3-Dihydrobenzofuran-6-yl)­phenoxy)­methyl)­isoxazole-3-carboxylic Acid (**10L**)

Yellow white solid (44% yield for 2 steps). ^1^H NMR (400 MHz, DMSO-*d*_6_): δ 7.58–7.52 (m, 2H), 7.50–7.44 (m, 1H), 7.34 (dd, *J* = 8.3, 2.0 Hz, 1H), 7.11 (d, *J* = 8.6 Hz, 2H), 6.98 (s, 1H), 6.81 (d, *J* = 8.3 Hz, 1H), 5.39 (s, 2H), 4.56 (t, *J* = 8.7 Hz, 2H), 3.22 (t, *J* = 8.7 Hz, 2H). C_19_H_15_NO_5_, MS calcd for *m*/*z* [M + H]^+^: 338.1 (calculated), 338.0 (found).

### General Procedure of Final Vinyl Sulfones

Sodium hydride (60% dispersion in mineral oil) (1.2 eq) in tetrahydrofuran was cooled to 0 °C with stirring under the protection of N_2_, followed by the dropwise addition of diethyl­(methylsulfonylmethyl)­phosphonate **12** (1.2 eq) in THF. The mixture was vigorously stirred at 0 °C for 30 min, then tert-butyl (2-oxoethyl)­carbamate **11** (1 eq) in THF was added dropwise. The reaction was stirred at rt for 2 h. After finished, water was added to quench the reaction and ethyl acetate was added to extract, and the aqueous layer was extracted with EtOAc for 2 times. Combined organic layer was then dried over Na_2_SO_4_, filtered, and evaporated. The residue was purified by flash column chromatography to afford tert-butyl (E)-(3-(methylsulfonyl)­allyl)­carbamate (**13**) with 52% yield. ^1^H NMR (400 MHz, CDCl_3_): δ 6.84 (dt, *J* = 15.2, 4.3 Hz, 1H), 6.45 (d, *J* = 15.2 Hz, 1H), 5.09 (s, 1H), 3.92 (d, *J* = 5.8 Hz, 2H), 2.88 (s, 3H), 1.39 (s, 9H).

**13** (1 eq) was dissolved in DCM, 4N HCl in dioxane (8 eq) was slow added to the mixture at 0 °C and the reaction was stirred at rt for 1 h. After the starting material was consumed totally (checked by TLC), the participate was filtered and washed with DCM for three times to get (E)-3-(methylsulfonyl) prop-2-en-1-amine hydrochloride (**14**) as a powder solid (60%). ^1^H NMR (400 MHz, DMSO-*d*_6_): δ 8.55 (s, 2H), 7.04 (dt, *J* = 15.5, 1.6 Hz, 1H), 6.78 (dt, *J* = 15.3, 5.5 Hz, 1H), 3.72 (s, 2H), 3.05 (s, 3H).

Acids (1.0 eq), **14** (1.1 eq) and DIPEA (3 eq) were dissolved in dimethylformamide and the mixture was stirred at 0 °C for 5 min. Then propanephosphonic acid anhydride (2.5 eq) was added and reaction was stirred at rt for overnight. After reaction done. The reaction was quenched with H_2_O and extracted with ethyl acetate for 3 times. The combined organic layers were washed with saturated aqueous NaHCO_3_ and aqueous NaCl. The organic layer was dried over Na_2_SO_4_, filtered, and evaporated. The residues were purified by flash column chromatography to yield the final product vinyl sulfonyl.

#### (E)-5-(2-Ethoxyphenyl)-N-(3-(methylsulfonyl)­allyl)-1H-pyrazole-3-carboxamide (Control Compound **RA-0002034**)

White solid (72% yield). ^1^H NMR (400 MHz, DMSO-*d*_6_): δ 8.55 (t, *J* = 5.8 Hz, 1H), 7.67 (dd, *J* = 7.6, 1.7 Hz, 1H), 7.28 (ddd, *J* = 8.7, 7.4, 1.7 Hz, 1H), 7.11–7.02 (m, 2H), 7.01–6.93 (m, 1H), 6.75 (dt, *J* = 15.3, 4.3 Hz, 1H), 6.62 (dt, *J* = 15.3, 1.8 Hz, 1H), 4.13–4.08 (m, 2H), 4.05 (td, *J* = 6.4, 6.0, 3.5 Hz, 2H), 2.94 (s, 3H), 1.34 (t, *J* = 6.9 Hz, 3H). ^13^C NMR (101 MHz, DMSO-*d*_6_): δ 155.5, 143.9, 130.5, 130.1, 128.2, 121.0, 113.2, 106.0, 64.1, 42.6, 39.1, 15.0. C_16_H_19_N_3_O_4_S, HRMS calcd for *m*/*z* [M + H]^+^: 350.1174 (calculated), 350.1165 (found).

#### (E)-5-(2-((2-Fluorobenzyl)­oxy)­phenyl)-N-(3-(methylsulfonyl)­allyl)-1H-pyrazole-3-carboxamide **15 (Jun14482)**

Yellow White solid (43% yield). ^1^H NMR (400 MHz, DMSO-*d*_6_): δ 8.59 (t, *J* = 5.9 Hz, 1H), 7.75 (dd, *J* = 7.8, 1.7 Hz, 1H), 7.55 (td, *J* = 7.7, 1.8 Hz, 1H), 7.44–7.40 (m, 1H), 7.39–7.34 (m, 1H), 7.24 (dt, *J* = 15.1, 8.0 Hz, 4H), 7.10–7.05 (m, 2H), 6.81 (dt, *J* = 15.2, 4.3 Hz, 1H), 6.68 (d, *J* = 15.3 Hz, 1H), 5.33 (s, 2H), 4.09 (t, *J* = 5.5 Hz, 2H), 3.01 (s, 3H). ^13^C NMR (101 MHz, DMSO-*d*_6_): δ 161.9, 159.4, 154.9, 143.9, 130.8, 130.8, 130.7, 130.5, 130.1, 128.4, 128.3, 125.0, 125.0, 124.2, 124.0, 121.6, 115.9, 115.7, 113.7, 106.1, 64.3, 64.2, 42.6, 39.1. C_21_H_20_FN_3_O_4_S, HRMS calcd for *m*/*z* [M + H]^+^: 430.1237 (calculated), 430.1247 (found).

#### (E)-5-(2-((3,5-Difluorobenzyl)­oxy)­phenyl)-N-(3-(methylsulfonyl)­allyl)-1H-pyrazole-3-carboxamide **16 (Jun14364)**

Yellow white solid (52% yield). ^1^H NMR (400 MHz, DMSO-*d*_6_): δ 8.56 (t, *J* = 6.0 Hz, 1H), 7.67 (d, *J* = 7.6 Hz, 1H), 7.31–7.25 (m, 1H), 7.16–7.10 (m, 4H), 7.09 (s, 1H), 7.05 (s, 1H), 7.00 (t, *J* = 7.5 Hz, 1H), 6.75 (dt, *J* = 15.4, 4.3 Hz, 1H), 6.66–6.59 (m, 1H), 5.25 (s, 2H), 4.04 (t, *J* = 5.2 Hz, 2H), 2.94 (s, 3H). 13C NMR (101 MHz, DMSO-*d*_6_): δ 164.1, 164.0, 161.7, 161.6, 154.7, 143.9, 142.2, 142.2, 142.1, 130.5, 130.1, 128.8, 121.7, 113.8, 113.7, 110.9, 110.8, 110.7, 110.6, 106.0, 103.9, 103.6, 103.3, 68.8, 42.6, 39.1. C_21_H_19_F_2_N_3_O_4_S, HRMS calcd for *m*/*z* [M + H]+: 448.1143 (calculated), 448.1147 (found).

#### (E)-N-(3-(Methylsulfonyl)­allyl)-1-(2-(trifluoromethyl)­phenyl)-1H-pyrazole-4-carboxamide **17 (Jun14852)**

White solid (79% yield). ^1^H NMR (400 MHz, DMSO-*d*_6_): δ 8.65 (t, *J* = 5.7 Hz, 1H), 8.52 (s, 1H), 8.18 (s, 1H), 7.97 (d, *J* = 7.9 Hz, 1H), 7.88 (t, *J* = 7.7 Hz, 1H), 7.78 (t, *J* = 7.7 Hz, 1H), 7.67 (d, *J* = 7.9 Hz, 1H), 6.87–6.72 (m, 2H), 4.13 (t, *J* = 4.7 Hz, 2H), 3.03 (s, 3H). ^13^C NMR (101 MHz, DMSO-*d*_6_): δ 161.8, 143.8, 140.6, 137.9, 134.3, 134.1, 130.6, 130.5, 129.5, 127.9, 127.8, 125.2, 124.9, 124.8, 122.0, 119.9, 42.6, 39.1. C_15_H_14_F_3_N_3_O_3_S, HRMS calcd for *m*/*z* [M + H]+: 374.0786 (calculated), 374.0790 (found).

#### (E)-N-(3-(Methylsulfonyl)­allyl)-1-(3-(trifluoromethoxy)­phenyl)-1H-Pyrazole-4-Carboxamide **18 (Jun14736)**

White solid (62% yield). ^1^H NMR (400 MHz, DMSO-*d*_6_): δ 9.07 (d, *J* = 2.1 Hz, 1H), 8.62 (t, *J* = 5.4 Hz, 1H), 8.23 (d, *J* = 1.9 Hz, 1H), 7.94 (d, *J* = 8.3 Hz, 1H), 7.89 (s, 1H), 7.75–7.61 (m, 1H), 7.37 (d, *J* = 8.2 Hz, 1H), 6.88–6.73 (m, 2H), 4.14 (t, *J* = 4.6 Hz, 2H), 3.01 (s, 3H). ^13^C NMR (101 MHz, DMSO-*d*_6_): δ 161.7, 149.4, 143.7, 141.2, 140.9, 132.1, 130.7, 130.3, 121.0, 119.6, 118.0, 112.0, 42.6, 39.1. C_15_H_14_F_3_N_3_O_4_S, HRMS calcd for *m*/*z* [M + H]+: 390.0735 (calculated), 390.0734 (found).

#### (E)-N-(3-(Methylsulfonyl)­allyl)-1-(3-(trifluoromethyl)­phenyl)-1H-pyrazole-4-carboxamide **19 (Jun14741)**

White solid (54% yield). ^1^H NMR (400 MHz, DMSO-*d*_6_): δ 9.16 (s, 1H), 8.66 (t, *J* = 5.8 Hz, 1H), 8.25 (s, 1H), 8.21 (d, *J* = 7.7 Hz, 2H), 7.79 (t, *J* = 7.9 Hz, 1H), 7.74 (d, *J* = 7.8 Hz, 1H), 6.88–6.72 (m, 2H), 4.15 (t, *J* = 4.6 Hz, 2H), 3.02 (s, 3H). ^13^C NMR (101 MHz, DMSO-*d*_6_): δ 161.7, 143.7, 141.3, 140.0, 131.5, 131.0, 130.7, 130.6, 130.4, 125.5, 123.9, 123.9, 123.0, 122.8, 121.1, 115.7, 115.7, 42.6, 39.1. C_15_H_14_F_3_N_3_O_3_S, HRMS calcd for *m*/*z* [M + H]+: 374.0786 (calculated), 374.0793 (found).

#### (E)-1-(4-Methoxyphenyl)-N-(3-(methylsulfonyl)­allyl)-1H-pyrazole-4-carboxamide **20 (Jun14742)**

Yellow white solid (75% yield). ^1^H NMR (400 MHz, DMSO-*d*_6_): δ 8.77 (s, 1H), 8.51 (t, *J* = 5.7 Hz, 1H), 8.07 (s, 1H), 7.72–7.65 (m, 2H), 7.08–6.96 (m, 2H), 6.84–6.63 (m, 2H), 4.06 (t, *J* = 4.8 Hz, 2H), 3.74 (s, 3H), 2.95 (s, 3H). ^13^C NMR (101 MHz, DMSO-*d*_6_): δ 162.0, 158.6, 143.8, 140.3, 133.2, 130.6, 129.3, 120.8, 120.1, 115.1, 55.9, 42.6, 39.1. C_15_H_17_N_3_O_4_S, HRMS calcd for *m*/*z* [M + H]+: 336.1018 (calculated), 336.1026 (found).

#### (E)-N-(3-(Methylsulfonyl)­allyl)-1-(3-(trifluoromethyl)­benzyl)-1H-pyrazole-4-carboxamide **21 (Jun14853)**

White solid (38% yield). ^1^H NMR (400 MHz, DMSO-*d*_6_): δ 8.49 (t, *J* = 5.7 Hz, 1H), 8.36 (s, 1H), 7.95 (s, 1H), 7.69 (d, *J* = 9.0 Hz, 2H), 7.61 (t, *J* = 7.6 Hz, 1H), 7.56 (d, *J* = 7.7 Hz, 1H), 6.84–6.65 (m, 2H), 5.48 (s, 2H), 4.07 (t, *J* = 4.3 Hz, 2H), 3.00 (s, 3H). 13C NMR (101 MHz, DMSO-*d*_6_): δ 162.1, 143.9, 139.6, 138.7, 132.5, 132.4, 130.5, 130.2, 129.8, 129.5, 125.9, 125.1, 125.0, 124.9, 124.8, 123.1, 118.8, 54.7, 42.6, 39.0. C_16_H_16_F_3_N_3_O_3_S, HRMS calcd for *m*/*z* [M + H]+: 388.0943 (calculated), 388.0962 (found).

#### (E)-5-(1H-Indol-3-yl)-N-(3-(methylsulfonyl)­allyl)­isoxazole-3-carboxamide **22 (Jun14617)**

Yellow solid (75% yield). ^1^H NMR (400 MHz, DMSO-*d*_6_): δ 11.88 (s, 1H), 9.07 (d, *J* = 6.2 Hz, 1H), 8.10 (d, *J* = 2.7 Hz, 1H), 7.92 (d, *J* = 7.7 Hz, 1H), 7.47 (d, *J* = 7.8 Hz, 1H), 7.19 (dd, *J* = 13.8, 7.1 Hz, 2H), 7.03 (d, *J* = 4.9 Hz, 1H), 6.89–6.66 (m, 2H), 4.10 (d, *J* = 4.8 Hz, 2H), 2.97 (s, 3H). ^13^C NMR (101 MHz, DMSO-*d*_6_): δ 168.7, 159.5, 159.2, 142.8, 136.8, 130.9, 127.3, 124.0, 123.0, 121.4, 119.8, 112.8, 103.3, 96.7, 42.5, 39.3. C_16_H_15_N_3_O_4_S, HRMS calcd for *m*/*z* [M + Na]+: 368.0681 (calculated), 368.0672 (found).

#### (E)-5-(3-(Dimethylamino)­phenyl)-N-(3-(methylsulfonyl)­allyl)­isoxazole-3-carboxamide **23 (Jun14778)**

White solid (60% yield). ^1^H NMR (400 MHz, DMSO-*d*_6_): δ 9.16 (d, *J* = 6.3 Hz, 1H), 7.43–7.30 (m, 2H), 7.20 (d, *J* = 5.9 Hz, 2H), 6.93–6.83 (m, 1H), 6.77 (d, *J* = 12.6 Hz, 2H), 4.17–4.11 (m, 2H), 3.02 (s, 3H), 2.98 (s, 6H). ^13^C NMR (101 MHz, DMSO-*d*_6_): δ 171.8, 159.6, 159.2, 151.1, 142.8, 130.9, 130.3, 116.6, 115.1, 113.8, 109.4, 100.2, 42.6, 40.5. C_16_H_19_N_3_O_4_S, HRMS calcd for *m*/*z* [M + H]+: 350.1174 (calculated), 350.1181 (found).

#### (E)-5-(3,4-Dimethoxyphenyl)-N-(3-(methylsulfonyl)­allyl)­isoxazole-3-carboxamide **24 (Jun14694)**

White solid (79% yield). ^1^H NMR (400 MHz, DMSO-*d*_6_): δ 9.07 (t, *J* = 5.8 Hz, 1H), 7.47–7.39 (m, 2H), 7.27 (d, *J* = 1.2 Hz, 1H), 7.05 (d, *J* = 8.4 Hz, 1H), 6.72 (dd, *J* = 5.7, 2.6 Hz, 2H), 4.07 (dd, *J* = 6.1, 3.2 Hz, 2H), 3.79 (s, 3H), 3.77 (s, 3H), 2.95 (s, 3H). ^13^C NMR (101 MHz, DMSO-*d*_6_): δ 169.0, 157.6, 157.1, 149.3, 147.5, 140.7, 128.8, 117.3, 117.2, 110.4, 107.5, 97.1, 54.1, 54.0, 40.5, 37.3. C_16_H_18_N_2_O_6_S, HRMS calcd for *m*/*z* [M + H]+: 367.0964 (calculated), 367.0959 (found).

#### (E)-N-(3-(Methylsulfonyl)­allyl)-5-(*m*-tolyl)­isoxazole-3-carboxamide **25 (Jun14502)**

White solid (80% yield). ^1^H NMR (400 MHz, DMSO-*d*_6_): δ 9.12 (t, *J* = 5.7 Hz, 1H), 7.70 (s, 1H), 7.67 (d, *J* = 7.7 Hz, 1H), 7.38 (t, *J* = 7.6 Hz, 1H), 7.29 (d, *J* = 8.5 Hz, 2H), 6.80–6.66 (m, 2H), 4.07 (dd, *J* = 5.9, 2.8 Hz, 2H), 2.95 (s, 3H), 2.33 (s, 3H). ^13^C NMR (101 MHz, DMSO-*d*_6_): δ 171.1, 159.7, 159.1, 142.7, 139.2, 131.9, 131.0, 129.6, 126.7, 126.6, 123.4, 100.2, 42.6, 39.4, 21.3. C_15_H_16_N_2_O_4_S, HRMS calcd for *m*/*z* [M + H]+: 321.0909 (calculated), 321.0915 (found).

#### (E)-5-(2,4-Dimethylphenyl)-N-(3-(methylsulfonyl)­allyl)­isoxazole-3-carboxamide **26 (Jun14528)**

White solid (76% yield). ^1^H NMR (400 MHz, DMSO-*d*_6_): δ 9.13 (t, *J* = 5.8 Hz, 1H), 7.59 (d, *J* = 7.9 Hz, 1H), 7.17 (s, 1H), 7.13 (d, *J* = 8.0 Hz, 1H), 7.03 (s, 1H), 6.73 (d, *J* = 2.5 Hz, 2H), 4.08 (dd, *J* = 5.9, 2.7 Hz, 2H), 2.95 (s, 3H), 2.39 (s, 3H), 2.27 (s, 3H). ^13^C NMR (101 MHz, DMSO-*d*_6_): δ 171.0, 159.3, 159.2, 142.8, 140.9, 136.3, 132.5, 130.9, 128.6, 127.6, 123.6, 102.4, 42.6, 39.4, 21.3, 21.2. C_16_H_18_N_2_O_4_S, HRMS calcd for *m*/*z* [M + H]+: 335.1066 (calculated), 335.1068 (found).

#### (E)-N-(3-(Methylsulfonyl)­allyl)-5-(*p*-tolyl)­isoxazole-3-carboxamide **27 (Jun14529)**

White solid (79% yield). ^1^H NMR (400 MHz, DMSO-*d*_6_): δ 9.10 (t, *J* = 5.8 Hz, 1H), 7.76 (d, *J* = 8.0 Hz, 2H), 7.30 (d, *J* = 8.0 Hz, 2H), 7.27 (s, 1H), 6.79–6.65 (m, 2H), 4.07 (dd, *J* = 5.9, 3.0 Hz, 2H), 2.95 (s, 3H), 2.31 (s, 3H). ^13^C NMR (101 MHz, DMSO-*d*_6_): δ 171.1, 159.7, 159.1, 142.8, 141.3, 130.9, 130.3, 126.2, 124.1, 99.7, 42.6, 39.4, 21.4. C_15_H_16_N_2_O_4_S, HRMS calcd for *m*/*z* [M + H]+: 321.0909 (calculated), 321.0899 (found).

#### (E)-5-(3-Cyanophenyl)-N-(3-(methylsulfonyl)­allyl)­isoxazole-3-carboxamide **28 (Jun14744)**

White solid (61% yield). ^1^H NMR (400 MHz, DMSO-*d*_6_): δ 9.17 (t, *J* = 5.8 Hz, 1H), 8.43–8.37 (m, 1H), 8.20 (dd, *J* = 8.2, 1.8 Hz, 1H), 7.94 (d, *J* = 7.8 Hz, 1H), 7.71 (t, *J* = 7.9 Hz, 1H), 7.53 (d, *J* = 3.2 Hz, 1H), 6.80–6.67 (m, 2H), 4.08 (dd, *J* = 5.9, 2.7 Hz, 2H), 2.95 (s, 3H). ^13^C NMR (101 MHz, DMSO-*d*_6_): δ 168.9, 159.9, 158.8, 142.7, 134.6, 131.1, 131.0, 130.6, 129.9, 127.8, 118.4, 113.1, 102.0, 42.6, 39.5. C_15_H_13_N_3_O_4_S, HRMS calcd for *m*/*z* [M + H]+: 332.0705 (calculated), 332.0696 (found).

#### (E)-5-(4-Cyanophenyl)-N-(3-(methylsulfonyl)­allyl)­isoxazole-3-carboxamide **29 (Jun14693)**

White solid (80% yield). ^1^H NMR (400 MHz, DMSO-*d*_6_): δ 9.18 (t, *J* = 5.8 Hz, 1H), 8.08 (d, *J* = 8.1 Hz, 2H), 7.98 (d, *J* = 8.1 Hz, 2H), 7.58 (s, 1H), 6.73 (d, *J* = 2.5 Hz, 2H), 4.08 (dd, *J* = 5.9, 2.6 Hz, 2H), 2.95 (s, 3H). ^13^C NMR (101 MHz, DMSO-*d*_6_): δ 169.0, 159.9, 158.8, 142.7, 133.7, 131.0, 130.5, 126.9, 118.7, 113.5, 102.8, 42.6, 39.5. C_15_H_13_N_3_O_4_S, HRMS calcd for *m*/*z* [M + H]+: 332.0705 (calculated), 332.0696 (found).

#### (E)-3-(3-((2-Fluorobenzyl)­oxy)­phenyl)-N-(3-(methylsulfonyl)­allyl)­isoxazole-5-carboxamide **30 (Jun14969)**

Yellow white solid (66% yield). ^1^H NMR (400 MHz, DMSO-*d*_6_): δ 9.21 (t, *J* = 5.8 Hz, 1H), 7.66–7.59 (m, 2H), 7.58–7.48 (m, 3H), 7.45 (dd, *J* = 8.4, 6.1 Hz, 1H), 7.28 (q, *J* = 8.1, 7.3 Hz, 2H), 7.22 (dd, *J* = 8.1, 2.4 Hz, 1H), 6.80 (d, *J* = 2.6 Hz, 2H), 5.26 (s, 2H), 4.15 (dd, *J* = 6.0, 2.8 Hz, 2H), 3.03 (s, 3H). ^13^C NMR (101 MHz, DMSO-*d*_6_): δ 170.7, 16.16, 159.7, 159.7, 159.1, 159.1, 142.7, 131.3, 131.2, 131.1, 131.0, 130.9, 128.0, 125.0, 125.0, 124.0, 123.9, 118.8, 118.0, 116.0, 115.8, 112.2, 100.9, 64.3, 64.3, 42.6, 39.4. C_21_H_19_FN_2_O_5_S, HRMS calcd for *m*/*z* [M + H]+: 431.1077 (calculated), 431.1080 (found).

#### (E)-3-(2-((2-Fluorobenzyl)­oxy)­phenyl)-N-(3-(methylsulfonyl)­allyl)­isoxazole-5-carboxamide **31 (Jun14454)**

White solid (62% yield). ^1^H NMR (400 MHz, DMSO-*d*_6_): δ 9.15 (t, *J* = 5.8 Hz, 1H), 7.93 (dd, *J* = 7.8, 1.7 Hz, 1H), 7.60 (t, *J* = 7.6 Hz, 1H), 7.55 (t, *J* = 7.8 Hz, 1H), 7.46 (q, *J* = 7.1 Hz, 1H), 7.40 (d, *J* = 8.4 Hz, 1H), 7.33–7.23 (m, 2H), 7.17 (t, *J* = 7.5 Hz, 1H), 7.00 (s, 1H), 6.83–6.71 (m, 2H), 5.39 (s, 2H), 4.11 (dd, *J* = 5.9, 3.2 Hz, 2H), 3.01 (s, 3H). ^13^C NMR (101 MHz, DMSO-*d*_6_): δ 167.2, 162.2, 159.7, 159.5, 159.1, 155.3, 142.8, 132.7, 131.3, 131.3, 131.2, 130.9, 127.8, 125.1, 125.1, 123.6, 123.5, 121.8, 116.1, 115.9, 115.6, 113.9, 103.0, 64.8, 64.8, 42.6, 39.4. C_21_H_19_FN_2_O_5_S, HRMS calcd for *m*/*z* [M + H]^+^: 431.1077 (calculated), 431.1092 (found).

#### (E)-3-(2-((3,5-Difluorobenzyl)­oxy)­phenyl)-N-(3-(methylsulfonyl)­allyl)­isoxazole-5-carboxamide **32 (Jun14363)**

White solid (68% yield). ^1^H NMR (400 MHz, DMSO-*d*_6_): δ 9.11 (t, *J* = 5.8 Hz, 1H), 7.87 (dd, *J* = 7.8, 1.7 Hz, 1H), 7.49–7.42 (m, 1H), 7.22 (d, *J* = 8.5 Hz, 1H), 7.17 (dd, *J* = 7.2, 3.2 Hz, 3H), 7.13–7.06 (m, 2H), 6.78–6.67 (m, 2H), 5.32 (s, 2H), 4.06 (dd, *J* = 6.0, 2.9 Hz, 2H), 2.94 (s, 3H). ^13^C NMR (101 MHz, DMSO-*d*_6_): δ 167.3, 164.2, 164.1, 161.7, 161.6, 159.5, 159.1, 155.1, 142.8, 141.6, 141.5, 141.4, 132.7, 130.9, 128.0, 121.9, 115.6, 114.0, 111.2, 111.2, 111.0, 111.0, 104.2, 103.9, 103.7, 103.2, 69.0, 42.6, 39.4. C_21_H_18_F_2_N_2_O_5_S, HRMS calcd for *m*/*z* [M + H]+: 449.0983 (calculated), 449.0984 (found).

#### (E)-3-(2-((3-Methoxybenzyl)­oxy)­phenyl)-N-(3-(methylsulfonyl)­allyl)­isoxazole-5-carboxamide **33 (Jun14435)**

White solid (39% yield). ^1^H NMR (400 MHz, DMSO-*d*_6_): δ 9.22 (t, *J* = 5.8 Hz, 1H), 7.98 (dd, *J* = 7.7, 1.6 Hz, 1H), 7.62–7.53 (m, 1H), 7.37 (t, *J* = 8.4 Hz, 2H), 7.24–7.16 (m, 2H), 7.16–7.13 (m, 1H), 7.10 (d, *J* = 7.5 Hz, 1H), 6.96 (dd, *J* = 8.4, 2.6 Hz, 1H), 6.89–6.77 (m, 2H), 5.37 (s, 2H), 4.17 (dd, *J* = 6.1, 3.0 Hz, 2H), 3.81 (s, 3H), 3.06 (s, 3H). ^13^C NMR (101 MHz, DMSO-*d*_6_): δ 167.3, 159.9, 159.5, 159.1, 155.5, 142.7, 138.4, 132.7, 130.9, 130.1, 127.8, 121.6, 120.1, 115.5, 114.3, 114.0, 113.3, 103.1, 70.3, 55.5, 42.6, 39.4. C_22_H_22_N_2_O_6_S, HRMS calcd for *m*/*z* [M + Na]^+^: 465.1096 (calculated), 465.1085 (found).

#### (E)-N-(3-(Methylsulfonyl)­allyl)-5-((3-(trifluoromethyl)­phenoxy)­methyl)­isoxazole-3-carboxamide **34 (Jun14855)**

Yellow white solid (74% yield). ^1^H NMR (400 MHz, DMSO-*d*_6_): δ 9.12 (t, *J* = 5.8 Hz, 1H), 7.50 (t, *J* = 7.9 Hz, 1H), 7.37–7.27 (m, 3H), 6.93 (s, 1H), 6.76–6.64 (m, 2H), 5.41 (s, 2H), 4.04 (dd, *J* = 5.9, 2.6 Hz, 2H), 2.93 (s, 3H). ^13^C NMR (101 MHz, DMSO-*d*_6_): δ 169.3, 159.0, 158.8, 158.1, 142.7, 131.3, 131.0, 130.9, 130.7, 125.7, 123.0, 119.6, 118.6, 118.5, 112.0, 111.9, 104.4, 60.8, 40.6, 39.4.

#### (E)-5-((4-Fluorophenoxy)­methyl)-N-(3-(methylsulfonyl)­allyl)­isoxazole-3-carboxamide **35 (Jun14458)**

White solid (85% yield). ^1^H NMR (400 MHz, DMSO-*d*_6_): δ 9.17 (t, *J* = 5.8 Hz, 1H), 7.16 (t, *J* = 8.7 Hz, 2H), 7.09 (dd, *J* = 9.3, 4.4 Hz, 2H), 6.96 (s, 1H), 6.77 (d, *J* = 2.4 Hz, 2H), 5.35 (s, 2H), 4.11 (dd, *J* = 5.9, 2.7 Hz, 2H), 3.01 (s, 3H). ^13^C NMR (101 MHz, DMSO-*d*_6_): δ 169.7, 158.9, 158.8, 158.6, 156.3, 154.1, 154.1, 142.6, 130.9, 116.8, 116.7, 116.5, 116.3, 104.2, 61.1, 42.5, 39.4. C_15_H_15_FN_2_O_5_S, HRMS calcd for *m*/*z* [M + H]+: 355.0764 (calculated), 355.0756 (found).

#### (E)-5-((3-Fluorophenoxy)­methyl)-N-(3-(methylsulfonyl)­allyl)­isoxazole-3-carboxamide **36 (Jun14861)**

White solid (72% yield). ^1^H NMR (400 MHz, DMSO-*d*_6_): δ 9.11 (t, *J* = 5.8 Hz, 1H), 7.29 (q, *J* = 8.1 Hz, 1H), 6.95–6.89 (m, 2H), 6.85 (dd, *J* = 8.4, 2.3 Hz, 1H), 6.77 (td, *J* = 8.5, 2.2 Hz, 1H), 6.70 (d, *J* = 1.8 Hz, 2H), 5.32 (s, 2H), 4.04 (dd, *J* = 6.0, 2.5 Hz, 2H), 2.93 (s, 3H). ^13^C NMR (101 MHz, DMSO-*d*_6_): δ 169.4, 164.6, 162.2, 159.3, 159.2, 159.0, 158.8, 142.6, 131.4, 131.3, 131.0, 111.6, 111.6, 108.8, 108.6, 104.3, 103.1, 102.8, 60.8, 42.6. C_15_H_15_FN_2_O_5_S, HRMS calcd for *m*/*z* [M + H]+: 355.0764 (calculated), 355.0758 (found).

#### (E)-5-(((4′-Fluoro-[1,1′-biphenyl]-4 yl)­oxy)­methyl)-N-(3-(methylsulfonyl)­allyl) isoxazole-3-carboxamide **37 (Jun14856)**

White solid (78% yield). ^1^H NMR (400 MHz, DMSO-*d*_6_): δ 9.12 (t, *J* = 5.9 Hz, 1H), 7.62–7.57 (m, 2H), 7.56–7.51 (m, 2H), 7.23–7.13 (m, 2H), 7.11–7.05 (m, 2H), 6.92 (s, 1H), 6.74–6.65 (d, *J* = 2.4 Hz, 2H), 5.35 (s, 2H), 4.04 (dd, *J* = 5.9, 2.7 Hz, 2H), 2.93 (s, 2H). ^13^C NMR (101 MHz, DMSO-*d*_6_): δ 169.8, 163.2, 160.8, 159.0, 158.8, 157.4, 142.7, 136.5, 136.5, 133.0, 130.9, 128.7, 128.6, 128.3, 116.1, 115.9, 115.7, 104.2, 60.6, 42.5, 39.4. C_21_H_19_FN_2_O_5_S, HRMS calcd for *m*/*z* [M + H]+: 431.1077 (calculated), 431.1070 (found).

#### (E)-5-(((2′-Fluoro-[1,1′-biphenyl]-4 yl)­oxy)­methyl)-N-(3-(methylsulfonyl)­allyl) isoxazole-3-carboxamide **38 (Jun14924)**

White solid (71% yield). ^1^H NMR (400 MHz, DMSO-*d*_6_): δ 9.19 (t, *J* = 5.9 Hz, 1H), 7.57–7.46 (m, 3H), 7.39 (dddd, *J* = 8.6, 7.0, 5.2, 1.7 Hz, 1H), 7.29 (ddt, *J* = 12.6, 7.5, 1.4 Hz, 2H), 7.22–7.16 (m, 2H), 7.01 (s, 1H), 6.78 (d, *J* = 1.9 Hz, 2H), 5.44 (s, 2H), 4.12 (dd, *J* = 6.0, 2.5 Hz, 2H), 3.01 (s, 3H). ^13^C NMR (101 MHz, DMSO-*d*_6_): δ 169.8, 160.7, 159.0, 158.8, 158.3, 157.6, 142.6, 131.0, 130.9, 130.5, 130.5, 129.6, 129.5, 128.8, 128.2, 128.1, 125.3, 125.3, 116.6, 116.40,115.4, 104.2, 60.7, 42.6. C_21_H_19_FN_2_O_5_S, HRMS calcd for *m*/*z* [M + H]+: 431.1077 (calculated), 431.1076 (found).

#### (E)-5-(((3′-Fluoro-[1,1′-biphenyl]-4 yl)­oxy)­methyl)-N-(3-(methylsulfonyl)­allyl)-isoxazole-3-carboxamide **39 (Jun14782)**

Yellow white solid (76% yield). ^1^H NMR (400 MHz, DMSO-*d*_6_): δ 9.12 (t, *J* = 5.8 Hz, 1H), 7.62 (d, *J* = 8.6 Hz, 2H), 7.46–7.35 (m, 3H), 7.14–7.04 (m, 3H), 6.93 (s, 1H), 6.70 (d, *J* = 2.5 Hz, 2H), 5.36 (s, 2H), 4.04 (dd, *J* = 5.9, 2.7 Hz, 2H), 2.93 (s, 3H). ^13^C NMR (101 MHz, DMSO-*d*_6_): δ 169.7, 164.4, 161.9, 159.0, 158.8, 157.9, 142.7, 142.5, 142.4, 132.6, 131.27, 131.1, 130.9, 128.5, 122.7, 122.7, 115.7, 114.1, 113.9, 113.4, 113.2, 104.2, 60.6, 42.5, 39.4. C_21_H_19_FN_2_O_5_S, HRMS calcd for *m*/*z* [M + H]+: 431.1077 (calculated), 431.1073 (found).

C_16_H_15_F_3_N_2_O_5_S, HRMS calcd for *m*/*z* [M + H]+: 405.0732 (calculated), 405.0739 (found).

#### (E)-5-(((2′,4′-Difluoro-[1,1′-biphenyl]-4 yl)­oxy)­methyl)-N-(3-(methylsulfonyl)­allyl) isoxazole-3-carboxamide **40 (Jun14897)**

White solid (67% yield). ^1^H NMR (400 MHz, DMSO-*d*_6_): δ 9.11 (t, *J* = 5.9 Hz, 1H), 7.48 (td, *J* = 8.9, 6.6 Hz, 1H), 7.42 (dd, *J* = 8.7, 1.7 Hz, 2H), 7.26 (ddd, *J* = 11.5, 9.2, 2.6 Hz, 1H), 7.10 (dq, *J* = 8.4, 3.0 Hz, 3H), 6.93 (s, 1H), 6.75–6.65 (m, 2H), 5.36 (s, 2H), 4.04 (dd, *J* = 6.0, 2.6 Hz, 2H), 2.93 (s, 3H). ^13^C NMR (101 MHz, DMSO-*d*_6_): δ 169.7, 159.0, 158.8, 157.6, 142.6, 132.1, 131.1, 130.55,130.5, 128.0, 124.8, 115.5, 112.4, 112.3, 105.1, 104.8, 104.2, 60.7, 42.6. C_21_H_18_F_2_N_2_O_5_S, HRMS calcd for *m*/*z* [M + H]+: 449.0983 (calculated), 449.0984 (found).

#### (E)-5-(((3′-Chloro-4′-fluoro-[1,1′-biphenyl]-4 yl)­oxy)­methyl)-N-(3-(methylsulfonyl) allyl)­isoxazole-3-carboxamide **41 (Jun14927)**

White solid (63% yield). ^1^H NMR (400 MHz, DMSO-*d*_6_): δ 9.12 (q, *J* = 8.8, 5.8 Hz, 1H), 7.85–7.75 (m, 1H), 7.61 (t, *J* = 9.4 Hz, 3H), 7.40 (q, *J* = 8.9 Hz, 1H), 7.10 (t, *J* = 9.1 Hz, 2H), 7.00–6.89 (m, 1H), 6.83–6.62 (m, 2H), 5.36 (s, 2H), 4.14–3.98 (m, 2H), 2.93 (s, 3H). ^13^C NMR (101 MHz, DMSO-*d*_6_): δ 169.7, 159.0, 158.8, 157.8, 142.6, 138.0, 131.7, 131.0, 128.6, 128.5, 127.3, 120.5, 117.7, 117.5, 115.8, 104.2, 60.7, 42.6. C_21_H_18_ClFN_2_O_5_S, HRMS calcd for *m*/*z* [M + H]+: 465.0687 (calculated), 465.0688 (found).

#### (E)-5-(((4’-(Dimethylamino)-[1,1′-biphenyl]-4 yl)­oxy)­methyl)-N-(3-(methylsulfonyl)­allyl)­isoxazole-3-carboxamide **42 (Jun15176)**

Yellow white solid (75% yield). ^1^H NMR (400 MHz, DMSO-*d*_6_): δ 9.19 (t, *J* = 5.9 Hz, 1H), 7.53 (d, *J* = 8.2 Hz, 2H), 7.46 (d, *J* = 8.4 Hz, 2H), 7.09 (d, *J* = 8.3 Hz, 2H), 6.98 (s, 1H), 6.82–6.72 (m, 4H), 5.38 (s, 2H), 4.11 (dd, *J* = 5.9, 2.6 Hz, 2H), 3.00 (s, 3H), 2.92 (s, 6H). ^13^C NMR (101 MHz, DMSO-*d*_6_): δ 170.0, 159.0, 158.8, 156.4, 150.0, 142.7, 134.4, 130.9, 127.8, 127.1, 127.1, 115.6, 113.2, 104.1, 60.6, 42.6, 40.6, 39.4. C_23_H_25_N_3_O_5_S, HRMS calcd for *m*/*z* [M + H]+: 456.1593 (calculated), 456.1606 (found).

#### (E)-5-(((7-Fluoronaphthalen-2-yl)­oxy)­methyl)-N-(3-(methylsulfonyl)­allyl)­isoxazole-3-carboxamide **43 (Jun14966)**

Yellow white solid (78% yield). ^1^H NMR (400 MHz, DMSO-*d*_6_): δ 9.19 (t, *J* = 5.9 Hz, 1H), 7.98–7.88 (m, 2H), 7.59 (dd, *J* = 10.6, 2.6 Hz, 1H), 7.51 (d, *J* = 2.6 Hz, 1H), 7.33–7.20 (m, 2H), 7.05 (s, 1H), 6.77 (d, *J* = 2.3 Hz, 2H), 5.48 (s, 2H), 4.11 (dd, *J* = 6.1, 2.6 Hz, 2H), 3.00 (s, 3H). ^13^C NMR (101 MHz, DMSO-*d*_6_): δ 169.5, 162.3, 159.9, 159.0, 158.8, 156.6, 142.6, 135.5, 131.0, 130.9, 130.9, 130.2, 126.4, 118.1, 114.5, 114.2, 110.4, 110.2, 107.8, 104.3, 60.6, 42.6. C_19_H_17_FN_2_O_5_S, HRMS calcd for *m*/*z* [M + Na]+: 427.0740 (calculated), 427.0735 (found).

#### (E)-5-((4-(2,3-Dihydrobenzofuran-6-yl)­phenoxy)­methyl)-N-(3-(methylsulfonyl)­allyl)­isoxazole-3-carboxamide **44 (Jun15172)**

Yellow white solid (68% yield). ^1^H NMR (400 MHz, DMSO-*d*_6_): δ 9.19 (t, *J* = 5.8 Hz, 1H), 7.53 (d, *J* = 8.8 Hz, 2H), 7.48 (d, *J* = 2.0 Hz, 1H), 7.33 (dd, *J* = 8.2, 2.0 Hz, 1H), 7.10 (d, *J* = 8.4 Hz, 2H), 6.98 (s, 1H), 6.80 (d, *J* = 8.2 Hz, 1H), 6.77 (d, *J* = 2.4 Hz, 2H), 5.39 (s, 2H), 4.55 (t, *J* = 8.7 Hz, 2H), 4.11 (dd, *J* = 6.0, 2.6 Hz, 2H), 3.21 (t, *J* = 8.7 Hz, 2H), 3.00 (s, 3H). ^13^C NMR (101 MHz, DMSO-*d*_6_): δ 169.9, 159.4, 158.9, 158.8, 156.7, 142.7, 134.4, 132.6, 130.9, 128.5, 127.8, 126.4, 123.6, 115.6, 109.5, 104.1, 71.5, 60.6, 42.5, 39.4, 29.5. C_23_H_22_N_2_O_6_S, HRMS calcd for *m*/*z* [M + H]+: 455.1277 (calculated), 455.1283 (found).

#### (E)-5-(((8-Fluoronaphthalen-2-yl)­oxy)­methyl)-N-(3-(methylsulfonyl)­allyl)­isoxazol-e-3-carboxamide **45 (Jun14926)**

Yellow solid (75% yield). ^1^H NMR (400 MHz, DMSO-*d*_6_): δ 9.36–9.11 (m, 1H), 7.97 (d, *J* = 9.2 Hz, 1H), 7.73 (d, *J* = 8.0 Hz, 1H), 7.53 (d, *J* = 2.6 Hz, 1H), 7.45–7.25 (m, 3H), 7.02 (d, *J* = 6.3 Hz, 1H), 6.91–6.75 (m, 2H), 5.55 (s, 2H), 4.25–4.00 (m, 2H), 3.00 (s, 3H). ^13^C NMR (101 MHz, DMSO-*d*_6_): δ 169.6, 159.0, 158.8, 156.3, 142.6, 131.0, 130.9, 130.3, 124.4, 124.3, 124.0, 120.1, 110.9, 110.8, 104.31,100.3, 60.8, 42.6. C_19_H_17_FN_2_O_5_S. HRMS calcd for *m*/*z* [M + Na]+: 427.0740 (calculated), 427.0750 (found).

#### (E)-5-(((1-(4-Chlorophenyl)-1H-pyrazol-3-yl)­oxy)­methyl)-N-(3-(methylsulfonyl) allyl)­isoxazole-3-Carboxamide **46 (Jun15111)**

White solid (68% yield). ^1^H NMR (400 MHz, DMSO-*d*_6_): δ 9.19 (t, *J* = 5.6 Hz, 1H), 8.47–8.39 (m, 1H), 7.78 (dd, *J* = 7.8, 5.2 Hz, 2H), 7.53 (h, *J* = 3.1 Hz, 2H), 6.99 (q, *J* = 3.0, 2.4 Hz, 1H), 6.83–6.69 (m, 2H), 6.20–6.14 (m, 1H), 5.50 (s, 2H), 4.10 (dd, *J* = 6.1, 3.0 Hz, 2H), 3.00 (s, 3H). ^13^C NMR (101 MHz, DMSO-*d*_6_): δ 171.9, 165.7, 161.3, 161.1, 144.9, 141.1, 133.3, 132.7, 132.1, 121.5, 106.6, 97.2, 63.5, 44.9, C_18_H_17_ClN_4_O_5_S, HRMS calcd for *m*/*z* [M + H]+: 437.0686 (calculated), 437.0693 (found).

#### (E)-N-(3-(Methylsulfonyl)­allyl)-5-(((1-phenyl-3-(trifluoromethyl)-1H-pyrazol-5-yl)­oxy)­methyl)­isoxazole-3-carboxamide **47 (Jun15175)**

White solid (41% yield). ^1^H NMR (400 MHz, DMSO-*d*_6_): δ 9.22 (t, *J* = 5.9 Hz, 1H), 7.65 (d, *J* = 7.6 Hz, 2H), 7.55 (t, *J* = 7.7 Hz, 2H), 7.46 (t, *J* = 7.5 Hz, 1H), 7.10 (s, 1H), 6.78 (s, 2H), 6.72 (s, 1H), 5.63 (s, 2H), 4.16–4.08 (m, 2H), 3.01 (s, 3H). ^13^C NMR (101 MHz, DMSO-*d*_6_): δ 167.7, 159.1, 158.6, 154.1, 142.6, 137.3, 130.9, 129.8, 128.6, 123.4, 105.3, 87.1, 64.5, 42.6, 39.4. C_19_H_17_F_3_N_4_O_5_S, HRMS calcd for *m*/*z* [M + H]+: 471.0950 (calculated), 471.0959 (found).

#### (E)-5-((4-(4-Fluorophenoxy)­phenoxy)­methyl)-N-(3-(methylsulfonyl)­allyl)­isoxazole-3-carboxamide **48 (Jun1551)**

White solid (71% yield). ^1^H NMR (400 MHz, DMSO-*d*_6_): δ 9.10 (d, *J* = 6.0 Hz, 1H), 7.16–7.08 (m, 2H), 7.01 (dd, *J* = 8.2, 4.9 Hz, 2H), 6.91 (q, *J* = 6.7, 6.3 Hz, 5H), 6.73–6.66 (m, 2H), 5.30–5.24 (m, 2H), 4.04 (dq, *J* = 6.5, 3.0 Hz, 2H), 2.99–2.89 (s, 3H). ^13^C NMR (101 MHz, DMSO-*d*_6_): δ 169.8, 159.0, 158.8, 154.1, 154.0, 151.4, 142.6, 131.0, 120.5, 120.0, 119.9, 116.9, 116.7, 104.1, 61.1, 42.6. C_21_H_19_FN_2_O_6_S, HRMS calcd for *m*/*z* [M + H]+: 447.1026 (calculated), 447.1017 (found).

#### (E)-3-(6-(1-(2,2-Difluorobenzo­[d]­[1,3]­dioxol-5-yl)­cyclopropane-1-carboxamido)-3-methylpyridin-2-yl)-N-(3-(methylsulfonyl)­allyl)­benzamide **49 (Jun16225)**

White solid (66% yield). ^1^H NMR (400 MHz, DMSO-*d*_6_): δ 8.94 (s, 1H), 8.91 (t, *J* = 5.7 Hz, 1H), 7.99–7.91 (m, 3H), 7.75 (d, *J* = 8.4 Hz, 1H), 7.63 (d, *J* = 7.5 Hz, 1H), 7.57 (d, *J* = 7.8 Hz, 2H), 7.43–7.32 (m, 2H), 6.89–6.79 (m, 1H), 6.79–6.72 (m, 1H), 4.16 (t, *J* = 4.9 Hz, 2H), 3.02 (s, 3H), 2.24 (s, 3H), 1.52 (q, *J* = 4.2 Hz, 2H), 1.18 (q, *J* = 4.3 Hz, 2H). ^13^C NMR (101 MHz, DMSO-*d*_6_): δ 171.6, 166.4, 155.8, 149.5, 143.6, 143.3, 142.5, 141.1, 140.1, 136.7, 134.1, 132.1, 131.6, 130.6, 128.5, 128.1, 127.3, 127.0, 126.8, 113.6, 112.6, 110.5, 42.6, 31.8, 19.1, 16.1. C_28_H_25_F_2_N_3_O_6_S. HRMS calcd for *m*/*z* [M + H]^+^: 570.1510 (calculated), 570.1518 (found).

#### (E)-5-(4-Chlorophenyl)-1-methyl-N-(3-(methylsulfonyl)­allyl)-1H-pyrazole-3-carboxamide **50 (Jun16226)**

Yellow white solid (73% yield). ^1^H NMR (400 MHz, DMSO-*d*_6_): δ 8.62 (t, *J* = 6.0 Hz, 1H), 7.67–7.57 (m, 4H), 6.86 (s, 1H), 6.86–6.76 (m, 1H), 6.67 (d, *J* = 15.3 Hz, 1H), 4.10 (t, *J* = 5.1 Hz, 2H), 3.93 (s, 3H), 3.02 (s, 3H). ^13^C NMR (101 MHz, DMSO-*d*_6_): δ 161.6, 145.3, 143.8, 143.7, 134.2, 130.8, 130.5, 129.3, 128.6, 107.0, 42.6, 39.1, 38.5. C_15_H_16_ClN_3_O_3_S. HRMS calcd for *m*/*z* [M + H]^+^: 354.0679 (calculated), 354.0681 (found).

#### (E)-N-(3-(Methylsulfonyl)­allyl)-2-(4-(trifluoromethyl)­phenyl)­thiazole-4-carboxamide **51 (Jun16227)**

White solid (70% yield). ^1^H NMR (400 MHz, DMSO-*d*_6_): δ 9.06 (t, *J* = 6.1 Hz, 1H), 8.48 (s, 1H), 8.31 (d, *J* = 8.1 Hz, 2H), 7.94 (d, *J* = 8.1 Hz, 2H), 6.93–6.72 (m, 2H), 4.19 (t, *J* = 5.0 Hz, 2H), 3.03 (s, 3H). ^13^C NMR (101 MHz, DMSO-*d*_6_): δ 165.8, 160.8, 151.0, 143.4, 136.4, 130.7, 127.6, 126.7, 126.7, 126.6, 126.6, 126.2, 42.6, 39.5. C_15_H_13_F_3_N_2_O_3_S_2_. HRMS calcd for *m*/*z* [M + H]^+^: 391.0398 (calculated), 391.0407 (found).

#### (E)-3-Methyl-N-(3-(methylsulfonyl)­allyl)-1-phenyl-1H-thieno­[2,3-*c*]­pyrazole-5-carboxamide **52 (Jun16228)**

White solid (65% yield). ^1^H NMR (400 MHz, DMSO-*d*_6_): δ 9.04 (t, *J* = 5.8 Hz, 1H), 8.00 (s, 1H), 7.75 (d, *J* = 8.0 Hz, 2H), 7.60 (t, *J* = 7.7 Hz, 2H), 7.34 (t, *J* = 7.4 Hz, 1H), 6.84 (m, 2H), 4.18 (dd, *J* = 6.0, 2.8 Hz, 2H), 3.04 (s, 3H). ^13^C NMR (101 MHz, DMSO-*d*_6_): δ 162.3, 144.3, 143.3, 142.2, 139.1, 137.0, 131.2, 130.8, 130.4, 126.1, 118.3, 117.4, 42.6, 39.7, 13.1. C_17_H_17_N_3_O_3_S_2_. HRMS calcd for *m*/*z* [M + H]^+^: 376.0789 (calculated), 376.0795 (found).

#### (E)-3-(5-(2-Fluorophenyl)-1,2,4-oxadiazol-3-yl)-N-(3-(methylsulfonyl)­allyl)­benza-mide **53 (Jun16229)**

White solid (76% yield). ^1^H NMR (400 MHz, DMSO-*d*_6_): δ 9.15 (t, *J* = 5.7 Hz, 1H), 8.63 (d, *J* = 2.2 Hz, 1H), 8.31–8.23 (m, 2H), 8.16 (d, *J* = 7.8 Hz, 1H), 7.82 (q, *J* = 7.1 Hz, 1H), 7.75 (t, *J* = 7.8 Hz, 1H), 7.61–7.48 (m, 2H), 6.92–6.76 (m, 2H), 4.20 (t, *J* = 4.7 Hz, 2H), 3.03 (s, 3H). ^13^C NMR (101 MHz, DMSO-*d*_6_): δ 173.2, 168.1, 165.9, 161.7, 159.1, 143.4, 136.3, 136.2, 135.3, 131.4, 130.9, 130.7, 130.4, 130.0, 126.6, 126.6, 126.0, 125.9, 117.9, 117.7, 112.2, 112.1, 42.6. C_19_H_16_FN_3_O_4_S. HRMS calcd for *m*/*z* [M + H]^+^: 402.0924 (calculated), 402.0932 (found).

#### (E)-N-(3-(Methylsulfonyl)­allyl)-3-(5-neopentyl-1,2,4-oxadiazol-3-yl)­benzamide **54 (Jun16387)**

Yellow white solid (65% yield). ^1^H NMR (400 MHz, CDCl_3_) δ 8.45 (s, 1H), 8.28 (d, *J* = 7.8 Hz, 1H), 8.02 (d, *J* = 7.8 Hz, 1H), 7.62 (t, *J* = 7.8 Hz, 1H), 7.02 (dt, *J* = 15.1, 4.5 Hz, 1H), 6.69 (d, *J* = 6.4 Hz, 1H), 6.56 (dd, *J* = 15.2, 2.5 Hz, 1H), 4.40–4.33 (m, 2H), 2.97 (s, 3H), 2.88 (s, 2H), 1.10 (s, 9H). ^13^C NMR (101 MHz, DMSO) δ 179.6, 167.6, 165.9, 143.4, 135.3, 130.7, 130.7, 130.2, 129.9, 127.0, 126.5, 55.3, 42.6, 40.6, 40.4, 40.2, 40.0, 39.8, 39.6, 39.6, 39.4, 31.9, 29.5. C_18_H_23_N_3_O_4_S, HRMS calcd for *m*/*z* [M + H]+: 378.1409 (calculated), 378.1631 (found).

### Procedure of Directly to-Biology Reaction

Before setting up the Directly to-biology reaction, each commercially available acid was prepared at 0.2 mmol/mL in DMF, and intermediates 14 and HATU were separately prepared at 0.22 mmol/mL in DMF. In a standard D2B reaction, the final concentration is 50 mmol/L, and the procedure is showing below: 100 μL stock solution of the acid (1 eq) and 100 μL stock solution of amine **14** (1.1 eq) were added to the HPLC vial, then 11 μL DIPEA (3 eq) was added by micropipette to the mixture at 0 °C and kept stirred for 5 min. Then, 100 μL of the HATU stock solution and the remaining 89 μL of DMF were added, and the reaction mixture was stirred at room temperature overnight. Once the reaction was completed, the vials were stored at −20 °C for further processing.

### Analysis of D2B Reaction Mixtures using HPLC and LCMS

Ten μL of the reaction mixture and 10 μL of the starting material solution were each dissolved separately in 1 mL of acetonitrile. LCMS was employed to determine the starting product, products, and byproducts. An analytical HPLC analysis was applied in the conversion calculation. In the HPLC trace of the starting material, the retention times of the reactant acids were indicated. In the HPLC trace of the reaction mixture, the corresponding peaks of the starting acid (t_1_) and the product (t_2_), along with their areas, were recorded. The relative yield is determined by % area in the HPLC trace, and the calculation formula is as follows: Peak area in t_2_/(Peak area in t_1_ + Peak area in t_2_) × 100%.

### Reagents

Chikungunya virus strain 181/25 was purchased from ATCC (VR-3360). Unless otherwise noted, all biological reagents, control compounds, and consumables were purchased from commercial vendors.

### CHIKV nsP2 Protein Expression

The full-length CHIKV nsP2 protein sequence (UniProt ID: F2YI08) was codon optimized for *Escherichia coli*. Expression (Figure S1) and inserted into pET-28a (+) vector with N-terminal noncleavable Hexa-His tag. The full-length Hexa-His nsP2 protein was expressed in *E. coli* BL21-CodonPlus (DE3)-RIL (Agilent, Part Number: 230245). 0.5 mM of isopropyl β-D-I-thiogalactopyranoside (IPTG, Invitrogen) was added once the OD reached 0.5 to initiate induction at 17 °C for 16 h. In the purification, the cell pellets were resuspended in lysis buffer (Na_2_HPO_4_ 50 mM, NaCl 300 mM, lysozyme 0.5 mg/ml, DNase I 0.02 mg/ml, PMSF 0.5 mM, glycerol 5%, pH 8.0). Cells were lysed by sonication on ice with 30% amplitude, 1 s on and 1 s off. Cell debris was removed by centrifugation. The supernatant was loaded onto the HisTrap HP column (Cytiva). The column was washed with buffer A (Na_2_HPO_4_ 50 mM, NaCl 300 mM, imidazole 30 mM, pH 8.0) until the baseline was reached. The protein was eluted using buffer B (Na_2_HPO_4_ 50 mM, NaCl 300 mM, imidazole 500 mM, pH 8.0) with a linear gradient for 4 CV. Eluted fractions were subjected to SDS-PAGE, and the fraction with more than 95% homogeneity was collected. The purified fractions were dialyzed in dialysis buffer (Na_2_HPO_4_ 50 mM, NaCl 300 mM, DTT 2 mM, glycerol 5%, pH 8.0) to remove the imidazole. The final product was fast-frozen by liquid nitrogen and stored in a −80 °C freezer.

### CHIKV nsP2 FRET Assay

The FRET assay of full-length Hexa-His nsP2 was carried out in 384-well plate format. The nsP2 protein was diluted in reaction buffer (HEPES 25 mM, Tween-20 0.003%, DTT 1 mM, pH 7.2) to a final concentration of 0.5 μM. 48 μL of diluted nsP2 was loaded into each well of the 384-well plate, followed by the addition of 1 μL of the testing compound, and the plate was incubated at room temperature for 30 min. The reaction was initiated by the addition of 1 μL of 1 mM FRET substrate (Dabcyl-RAGGYIFS-E­(Edans)-amide). The FRET substrate was synthesized by ABclonal with a >95% purity (Theoretical MW: 1497.61; Observed: 749.80 [M+2H]^2+^). The fluorescent signal at the excitation of 340 nm and the emission of 460 was monitored every 71 s for 1 h in Cytation5 (BioTeck) plate reader. The initial velocity of each reaction was obtained by plotting the first 50 min of the fluorescent signal against time and fitting with linear regression. The half-maximal inhibitory concentration (IC_50_) was determined by plotting the initial velocity against log concentration, and fitting the data with nonlinear regression (Log­[inhibitor] vs normalized response – variable slope) in GraphPad Prism 8.0. The reported values are the means of two independent replicates, and the error bar represents 95% CI.

### Covalent Docking

The crystal structure of CHIKV nsP2 protease (PDB ID: 3TRK) was retrieved from the Protein Data Bank and processed using *QuickPrep* in MOE. All docking ligands were imported as a database in MOE. Each molecule was washed and processed, with protonation set to dominant and the coordinate set to Rebuilt 3D. Then, molecular docking was conducted using the *Covalent* panel in the *Dock* module. The sulfur atom on the side chain of Cys478 was selected as the reactive site, and the reaction type was selected as vinyl sulfone to beta-mercapto sulfonyl through 1,4-addition. Other parameters were set as default.

### Virtual Library Enumeration, Preparation, and Virtual Screening

The reaction-based combinatorial virtual library was generated using the Enumerate Combinatorial Library module in DataWarrior (v06.04.02, openmolecules), with amide bond formation selected as the reaction type. The number of commercially available carboxylic acid reactants was set to 20,000 from different vendors. The properties of all the carboxylic acids, including molecular weight (MW) and aromatic ring count (AR number), were computed. The ones with favorable properties, as determined by the SAR studies (150 < MW < 500 and 1 < AR Number <5), were preserved. Approximately 6200 carboxylic acids are preserved. R-group decomposition was also performed to further enable substructure analysis. Carboxylic acids containing reactive functional groups and unfavorable moieties, such as aldehyde, Michael acceptors, protecting groups, and so on, were excluded. About 3000 carboxylic acids remained and were set for amide formation. (*E*)-2-amino-*N*-(3-(methylsulfonyl)­allyl)­acetamide was used as an amine reactant to generate the reaction-based virtual library. For all enumerated compounds, key physicochemical properties, including molecular weight (MW) and calculated logP (cLogP), were computed. Compounds with 280 < MW < 660 and 0 < cLogP <5 were kept. Virtual screening was then conducted using the Covalent panel in the Dock module, following the same procedure as the covalent docking method. To retain the poses, the placement number was set to 20 and the refinement number to 1. The docking poses were ranked by the docking score “*S*”. The top 20% compounds (about 500) for each library, along with their corresponding docking poses, were visually checked and compared with the docking pose of the control compound, **RA-0002034**. The carboxylic acids of promising compounds were purchased for D2B synthesis and following bioevaluations. In addition to the docking score, compounds were prioritized based on favorable warhead orientation toward Cys478, conservation of key protein–ligand interactions, occupancy of the substrate-binding pocket, absence of severe steric clashes, structural diversity, and synthetic accessibility. Based on these criteria, approximately 100 carboxylic acid building blocks were selected for purchase, followed by D2B synthesis and biological evaluation.

### Cytopathic Effect Assay

The CPE assay was performed with Vero cells using the neutral red uptake method.^47,48^ Vero cells (about 84,000 cells/mL) grown in DMEM with 10% FBS and 1% PS were dispensed into 96-well cell culture plates at 100 μL/well. The plates were incubated at 37 °C in a 5% CO_2_ atmosphere for 24 h. Then, the growth DMEM medium was aspirated, and the cells were washed with 100 μL PBS buffer. After aspiration, the cells were infected with the virus diluted in 100 μL DMEM with 2% FBS and 1% PS antibiotic. The plates were then incubated for 1 h at 37 °C in a 5% CO_2_ atmosphere, followed by adding 1 μL of the testing compounds and 100 μL of fresh DMEM medium containing 2% FBS and 1% PS antibiotic. After 72 h of incubation at 37 °C in a 5% CO_2_ atmosphere, the medium was aspirated and replaced with 100 μL serum-free DMEM medium containing 66 μg/mL neutral red and incubated at 37 °C in a 5% CO_2_ atmosphere for 2 h^47^. After aspiration and washing with 100 μL PBS buffer, 100 μL neutral red destain solution was added to extract the neutral red from the cells.^47^ The plate was put on an orbital shaker until a homogeneous solution formed in each well, and the amount of neutral red taken up was determined by measuring the absorbance at 540 nm using a BioTek Microplate Epoch 2 Reader (Agilent). The EC_50_ values were calculated from best-fit dose–response curves with the variable slope using GraphPad Prism 8.

### Cytotoxicity Assay

The cytotoxicity of the compounds was determined using a procedure similar to the CPE assay, but without virus infection. Vero cells (about 84,000 cells/mL) grown in DMEM with 10% FBS and 1% PS were dispensed into 96-well cell culture plates at 100 μL/well. The plates were incubated at 37 °C in a 5% CO_2_ atmosphere for 24 h. After washing, PBS was aspirated, and 100 μL of fresh DMEM medium containing 2% FBS and 1% PS was added, followed by the addition of 1 μL of serially diluted compounds. Another 100 μL fresh DMEM medium with 2% FBS and 1% PS was then added. After 72 h of incubation at 33 °C in a 5% CO_2_ atmosphere, the cells were stained with 66 μg/mL neutral red in serum-free DMEM medium. The same procedure as in the CPE assay was followed to measure absorbance at 540 nm. The CC_50_ values were calculated from best-fit dose–response curves with the variable slope using GraphPad Prism 8.

### Plaque Assay

Vero cells (about 420,000 cells/mL) grown in DMEM with 10% FBS and 1% PS were dispensed into 6-well cell culture plates at 3 mL/well and incubated at 37 °C in a 5% CO_2_ atmosphere. After 24 h, the growth DMEM medium was aspirated and washed with 2 mL PBS buffer. After aspiration, cells were infected with 500 μL of diluted virus in DMEM containing 2% FBS and 1% PS antibiotic. The plates were then incubated for 1 h at 37 °C in a 5% CO_2_ atmosphere. During the incubation, a 0.6% Avicel microcrystalline cellulose (FMC BioPolymer, Philadelphia, PA) overlay in DMEM media with 2% FBS and different concentrations of compounds was prepared. After aspiration, 4 mL Avicel overlay solution was added to each well. After 72 h of incubation at 37 °C in a 5% CO2 atm, the overlay was aspirated from each well, and the wells were washed with PBS. Then, the cells were stained with crystal violet dye solution (0.2% crystal violet, 20% methanol). Plaque areas were quantified using ImageJ.^49^

### Broad-Spectrum Antiviral Assay

The *in vitro* potency of the nsP2 inhibitors was tested against a set of alphaviruses that included: Chikungunya virus 181/25 (BEI Resources, catalog number: NR-56523), O’nyong nyong virus UgMP30 (BEI Resources, catalog number: NR-51661), Mayaro virus Guyane (BEI Resources, catalog number: NR-49911), Ross River virus Rarotonga (BEI Resources, catalog number: NR-51647), and Sindbis virus 80–2449 (BEI Resources, catalog number: NR-51641). Viral stocks were propagated in Vero cells CCL-81 (ATCC) grown in EMEM (Eagel’s Minimal Essential Medium) plus 10% heat-killed Fetal Bovine Serum plus 1% penicillin–streptomycin and were tittered using a TCID_50_ assay as described previously.^50^

To test the compounds, Vero cells were seeded in a 96-well plate and incubated at 37 °C with 5% CO_2_ until they reached 90% confluence in each well. These cells were simultaneously infected with 100 plaque-forming units (PFU) of each virus and treated with 3-fold serial dilutions (100 μM-0.046 μM) of each nsP2 inhibitor, in triplicate. Uninfected and/or untreated wells served as controls. 48 h later, the cell culture media was removed, and a 0.2% Crystal violet (Sigma-Aldrich, catalog number: C6158) solution was used to visualize cytopathic effects. Two hours later, plates were destained by washing in tap water and imaged. The pixel intensity of each well was measured in ImageJ 1.54g (NIH). For each compound/virus, the pixel intensity relative to untreated wells was plotted against concentration, and GraphPad Prism (version 10.4.2) was used to calculate the EC_50_ using the asymmetrical curve-fitting function.

### Determination of k_inact_/K_i_

The k_inact_/K_i_ assay of the nsP2 protease was performed in a 384-well plate. The experiment was designed based on an occupancy-based covalent kinetics approach,^45^ in which Activity % was used as a surrogate for Occupancy %. The nsP2 cysteine protease was diluted in reaction buffer (HEPES 25 mM, Tween-20 0.003%, DTT 1 mM, pH 7.2) to a final concentration of 1 μM. Subsequently, 48 μL of diluted protein solution was loaded into each well of the 384-well plate, followed by the addition of 1 μL of testing compound. The compounds were prepared in a 2-fold serial dilution starting from 5 μM. A diagonal design was applied to pair inhibitor concentrations with corresponding preincubation times, where 5, 2.5, 1.25, 0.625, 0.3125, 0.15625, 0.078, and 0 μM corresponded to preincubation times of 90, 60, 30, 15, 10, 5, 2, and 0 min, respectively. The reaction was initiated by adding 1 μL of 1 mM FRET substrate. The fluorescent signal at 340 nm excitation and 460 nm emission was monitored every 71 s for 1 h using a Cytation 5. The initial velocity of each reaction was determined by plotting the first 30 min of the fluorescent signal against time and fitting the data with linear regression. OC_50_ values were determined by plotting the normalized initial velocity against the logarithm of the reciprocal product of inhibitor concentration and preincubation time, followed by nonlinear regression fitting (Log­[inhibitor] vs normalized response – variable slope) in GraphPad Prism to evaluate the OC_50_ of the compounds. The k_inact_/K_I_ values were subsequently calculated using the equation k_inact_/K_i_ = ln2 * OC_50_.

### MD Simulation

MD simulations were carried to the noncovalent complex of **53 (Jun16229)** and CHIKV nsP2. In the covalent docking pose of **53**/nsP2 complex, the covalent bond between vinyl sulfone and cysteine 478 was converted back to the prereaction states, followed by energy minimization under the OPLS3 force field with Protein Prepare Wizard in Schrödinger suite (Schrödinger, LLC, New York, NY, 2023). The resulting noncovalent complexes were subjected to 200 ns MD simulations with Desmond in the Schrödinger suite (D.E. Shaw Research, New York, NY, 2023; Maestro-Desmond Interoperability Tools, Schrödinger, New York, NY, 2023). The complex was solvated using the SPC water model and embedded in an orthorhombic water box extending beyond the solute by 10 Å in the x, y, and *z* directions. Sodium and chloride ions were added randomly in the water phase to neutralize the system. For the MD workflow, the simulation time was set to 200 ns, with recording intervals of 20 ps, yielding 10,000 frames for further analysis. Other parameters were set as default. The visualization of the trajectories was performed using the graphical user interface (GUI) of Maestro, and the protein–ligand interaction analysis was done with the simulation interaction diagram (SID) tool, available with the Desmond module as previously described.^51^

### Glutathione Reactivity Assay

The reaction buffer (100 mM KH_2_PO_4_, pH 7.4) was prepared and degassed by purging with argon before using. Each 500 μL reaction contained 335 μL of buffer, 150 μL of acetonitrile (30%, v/v), 5 μL of 5 mM fluorescein or TAMRA in DMSO as an internal standard (final concentration 50 μM), and 5 μL of a 5 mM test compound in DMSO (final concentration 50 μM). The mixture was vortexed, centrifuged, and the supernatant was transferred to an LC-MS sample vial. The reaction was initiated by the addition of 5 μL of 250 mM GSH in water (final concentration 2.5 mM). Samples were analyzed every 30 min until >50% depletion of the parent compound was observed. The area under the curve (AUC) of the parent compound was quantified and normalized to the internal standard, and the natural log of the normalized AUC was fit by linear regression to derive the depletion rate constant (-k). Because [GSH] ≫ [compound], the second-order reaction was treated as pseudo-first order, and the GSH reactivity half-life was calculated as GSH t_1/2_ = ln(2)/k.

## Supplementary Material









## Data Availability

The data underlying this study are available in the published article and its online Supporting Information

## Notes

The Supporting Information is free of charge at Molecular formula strings Tables S1 and S2; Figures S1–S3; NMR spectra and HPLC traces; PDB files for the docking models of nsp2 with RA-0002034 (Figure 3) and compound **53** (Figure 9A) (PDF)
(PDB)
(PDB)
(CSV)

## Abbreviations USED

CHIKV: chikungunya virus
CPE: cytopathic effect
D2B: direct to biology
DMTA: design-make-test-analyze
MAYV: Mayaro virus
MOE: Molecular Operating Environment
MW: molecular weight
nsP2: nonstructural protein 2
RRV: Ross River virus
SI: selectivity index
VEEV: Western Equine Encephalitis virus.
