# Supplementary material for: Development of Covalent Inhibitors of Chikungunya Virus nsP2 Cysteine Protease Enabled by Direct-to-Biology Synthesis and Screening
Source: J Med Chem. 2026 Aug 20;69(17):20138–59. doi: 10.1021/acs.jmedchem.5c03672 (PMC13576448; doi:10.1021/acs.jmedchem.5c03672)

Supplementary Information

Development of covalent inhibitors of Chikungunya virus nsP2 cysteine protease enabled by direct-to-biology synthesis and screening

Zhengjun Cai,[1,#] Kan Li,[1,#] Sainetra Sridhar,[2] Haozhou Tan,[1] Hiwot Demssie,[1] Gaungjin Fan,[1] Wenyi Zhang,[1] Bobby Brooke Herrera,[2] Jun Wang[1,*]

[1]Department of Medicinal Chemistry, Ernest Mario School of Pharmacy, Rutgers, the State University of New Jersey, Piscataway, NJ, 08854, USA

[2]Rutgers Global Health Institute, New Brunswick, NJ, 08901, USA; Department of Medicine, Division of Allergy, Immunology, and Infectious Diseases, and Child Health Institute of New Jersey, Rutgers Robert Wood Johnson Medical School, New Brunswick, NJ, 08901, USA

[#]These authors contributed equally to this work

*Corresponding authors. Email: junwang@pharmacy.rutgers.edu (J.W.)

Table of Contents

Table S1. %conversion from HPLC, IC50, and pIC50 values of nsP2 inhibitors and corresponding reaction mixtures. S2

Table S2. %conversion from HPLC, IC_{50}, and pIC_{50} values of hits and corresponding reaction mixtures. S3

Figure S1. Sequences of CHIKV nsP2 constructs used in this study, full-length Hexa-His nsP2. S4

Figure S2. Plaque assay results of lead compounds in inhibiting CHIKV 181/25 strain. S5

Figure S3. Sequence alignment of the nsP2 protease domain from alphaviruses CHIKV (181/25), ONNV (UgMP30), MAYV (Guyane), RRV (Rarotonga), and SINV (80-2449). S6

NMR spectra S7-S58

HPLC traces S59-S73

HPLC traces for D2Bs S74-S107

Table S1. %conversion from HPLC, IC_{50}, and pIC_{50} values of nsP2 inhibitors and corresponding reaction mixtures.

IDs	HPLC %	D2B IC_{50} (μM)	Corrected D2B pIC_{50}	pure IC_{50}	pure pIC_{50} (μM)
**15 Jun14482 (8-P077-11)**	50	1.52	6.12	0.36 ± 0.03	6.44
**16 Jun14364 (8-P077-8)**	59	1.71	6.00	0.36 ± 0.08	6.44
**17 Jun14852 (8-P077-16)**	100	4.33	5.36	5.18 ± 0.40	5.29
**18 Jun14736 (8-P077-5)**	73	2.35	5.77	2.03 ± 0.18	5.69
**19 Jun14741 (8-P077-6)**	75	2.48	5.73	1.75 ± 0.17	5.76
**20 Jun14742 (8-P077-17)**	100	1.53	5.82	2.03 ± 0.14	5.69
**21 Jun14853 (8-P077-23)**	53	5.73	5.52	1.87 ± 0.09	5.73
**22 Jun14617 (8-P077-4)**	88	0.39	6.46	0.13 ± 0.01	6.89
**23 Jun14778 (8-P077-14)**	75	0.66	6.31	0.53 ± 0.05	6.28
**24 Jun14694 (8-P077-13)**	94	0.4	6.42	0.27 ± 0.02	6.57
**25 Jun14502 (8-P077-2)**	98	0.44	6.37	0.10 ± 0.01	7.00
**26 Jun14528 (8-P077-3)**	91	0.55	6.30	0.18 ± 0.02	6.74
**27 Jun14529 (8-P077-1)**	95	0.57	6.27	0.34 ± 0.02	6.47
**28 Jun14744 (8-P077-22)**	77	1.05	6.09	0.22 ± 0.01	6.66
**29 Jun14693 (8-P077-12)**	100	1.93	5.71	0.57 ± 0.04	6.24
**30 Jun14969 (8-P077-29)**	94	0.53	6.30	0.23 ± 0.01	6.64
**31 Jun14454 (8-P077-10)**	68	0.61	6.38	0.48 ± 0.03	6.32
**32 Jun14363 (8-P077-7)**	75	0.83	6.21	0.49 ± 0.04	6.31
**33 Jun14435 (8-P077-9)**	45			0.82 ± 0.05	6.09
**34 Jun14855 (8-P077-18)**	100	0.96	6.02	0.41 ± 0.02	6.39
**35 Jun14458 (8-P077-21)**	100	0.98	6.01	0.40 ± 0.03	6.40
**36 Jun14861 (8-P077-19)**	100	0.93	6.03	0.35 ± 0.02	6.46
**37 Jun14856 (8-P077-20)**	99	0.47	6.33	0.16 ± 0.01	6.80
**38 Jun14924 (8-P077-25)**	98	0.74	6.14	0.29 ± 0.02	6.54
**39 Jun14782 (8-P077-15)**	100	0.85	6.07	0.16 ± 0.01	6.80
**40 Jun14897 (8-P077-24)**	100	0.88	6.06	0.25 ± 0.02	6.60
**41 Jun14927 (8-P077-27)**	96	0.89	6.07	0.25 ± 0.02	6.60
**42 Jun15176 (8-P077-34)**	94	1.23	5.94	0.4 ± 0.03	6.40
**43 Jun14966 (8-P077-28)**	100	0.61	6.21	0.26 ± 0.01	6.59
**44 Jun15172 (8-P077-32)**	100	0.56	6.25	0.28 ± 0.02	6.55
**45 Jun14926 (8-P077-26)**	99	0.93	6.04	0.27 ± 0.01	6.57
**46 Jun15111 (8-P077-30)**	100	0.83	6.08	0.31 ± 0.01	6.51
**47 Jun15175 (8-P077-33)**	50	5.92	5.53	2.5 ± 0.13	5.60
**48 Jun1551 (8-P077-31)**	100	0.63	6.20	0.25 ± 0.01	6.60

For the IC_{50} obtained from FRET assays, the plotted values are the mean ± SD (*n* = 2).

Table S2. %conversion from HPLC, IC_{50}, and pIC_{50} values of hits and corresponding reaction mixtures**.**

IDs	HPLC %	D2B IC_{50} (μM)	Corrected D2B pIC_{50}	pure IC_{50}	pure pIC_{50} (μM)
**49 Jun16225 (8-P013-1a)**	76	0.69	6.28	0.22 ± 0.01	6.66
**50 Jun16226 (8-P019-2a)**	81	10	5.09	2.2 ± 0.2	5.66
**51 Jun16227 (8-P003-5a)**	65	4	5.59	1.2 ± 0.1	5.92
**52 Jun16228 (8-P031-4a)**	58	3	5.76	1.0 ± 0.1	6.00
**53 Jun16229 (8-P021-4a)**	88	0.56	6.31	0.27 ± 0.02	6.57
**54 Jun16387 (HD-4-143-1)**	81	0.43	6.46	0.86 ± 0.16	6.07

For the IC_{50} obtained from FRET assays, the plotted values are the mean ± SD (n = 2).

MGSSHHHHHHGIIETPRGAIKVTAQPTDHVVGEYLVLSPQTVLRSQKLSLIHALAEQVK
TCTHNGRAGRYAVEAYDGRVLVPSGYAISPEDFQSLSESATMVYNEREFVNRKLHHIA
MHGPALNTDEESYELVRAERTEHEYVYDVDQRRCCKKEEAAGLVLVGDLTNPPYHEFA
YEGLKIRPACPYKIAVIGVFGVPGSGKSAIIKNLVTRQDLVTSGKKENCQEITTDVMRQR
GLEISARTVDSLLLNGCNRPVDVLYVDEAFACHSGTLLALIALVRPRQKVVLCGDPKQC
GFFNMMQMKVNYNHNICTQVYHKSISRRCTLPVTAIVSSLHYEGKMRTTNEYNKPIVV
DTTGSTKPDPGDLVLTCFRGWVKQLQIDYRGYEVMTAAASQGLTRKGVYAVRQKVNE
NPLYASTSEHVNVLLTRTEGKLVWKTLSGDPWIKTLQNPPKGNFKATIKEWEVEHASIM
AGICSHQMTFDTFQNKANVCWAKSLVPILETAGIKLNDRQWSQIIQAFKEDKAYSPEVA
LNEICTRMYGVDLDSGLFSKPLVSVYYADNHWDNRPGGKMFGFNPEAASILERKYPFT
KGKWNINKQICVTTRRIEDFNPTTNIIPANRRLPHSLVAEHRPVKGERMEWLVNKINGHH
VLLVSGYNLALPTKRVTWVAPLGVRGADYTYNLELGLPATLGRYDLVVINIHTPFRIHHY
QQCVDHAMKLQMLGGDSLRLLKPGGSLLIRAYGYADRTSERVICVLGRKFRSSRALKP
PCVTSNTEMFFLFSNFDNGRRNFTTHVMNNQLNAAFVGQVTRAGC*

6X-His tag   Helicase Domain   Protease Domain

Figure S1. Sequences of CHIKV nsP2 constructs used in this study, full-length Hexa-His nsP2. The amino acid sequence of the 6xHis tag was colored in teal, the helicase domain was colored in yellow, and the protease domain was colored in red.

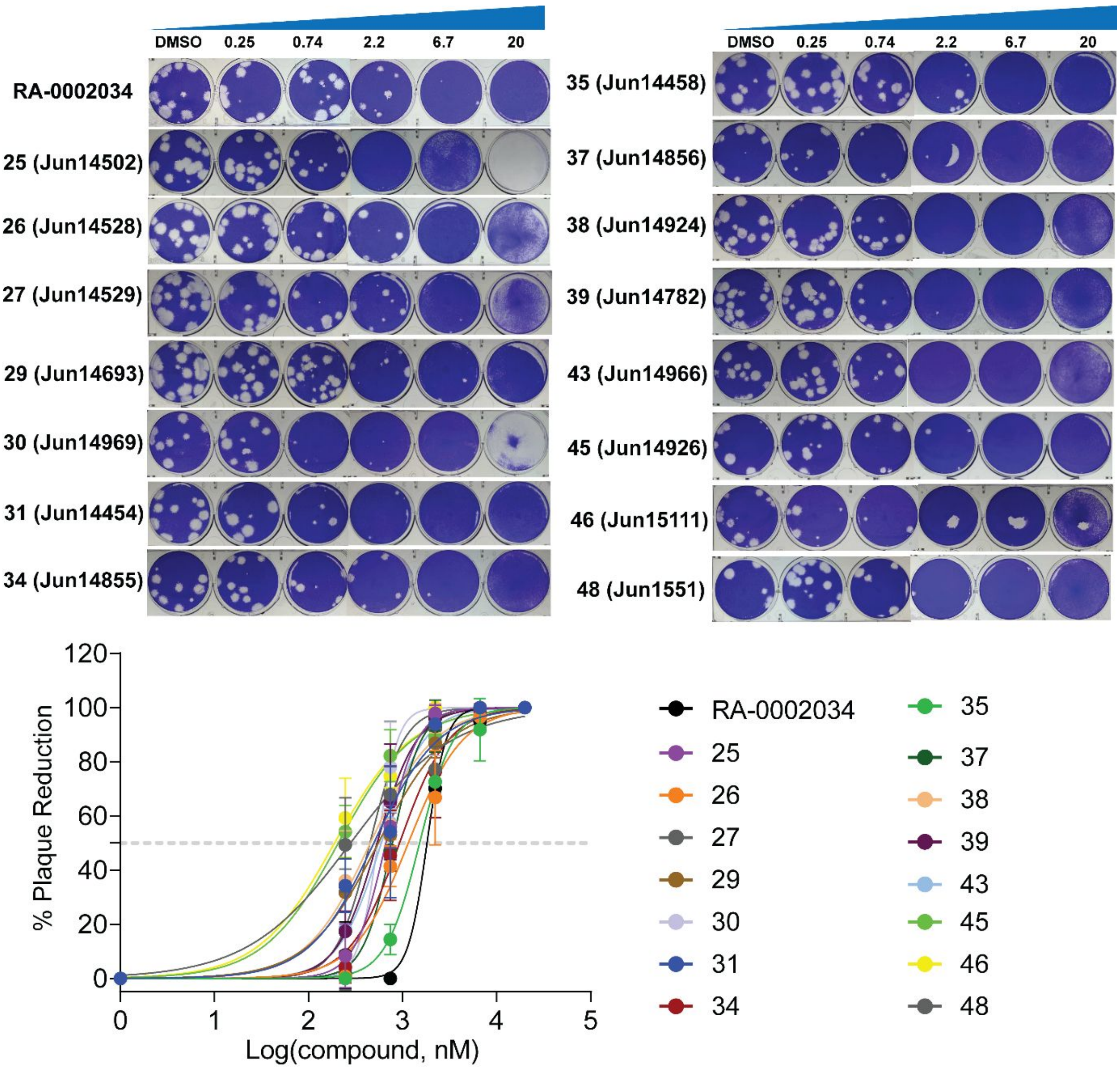

Figure S2. Plaque assay results of lead compounds in inhibiting CHIKV 181/25 strain. The images are representatives of two repeats. The dot with error bar represents the mean ± standard deviation of two repeats and the results are summarized in Table 1.

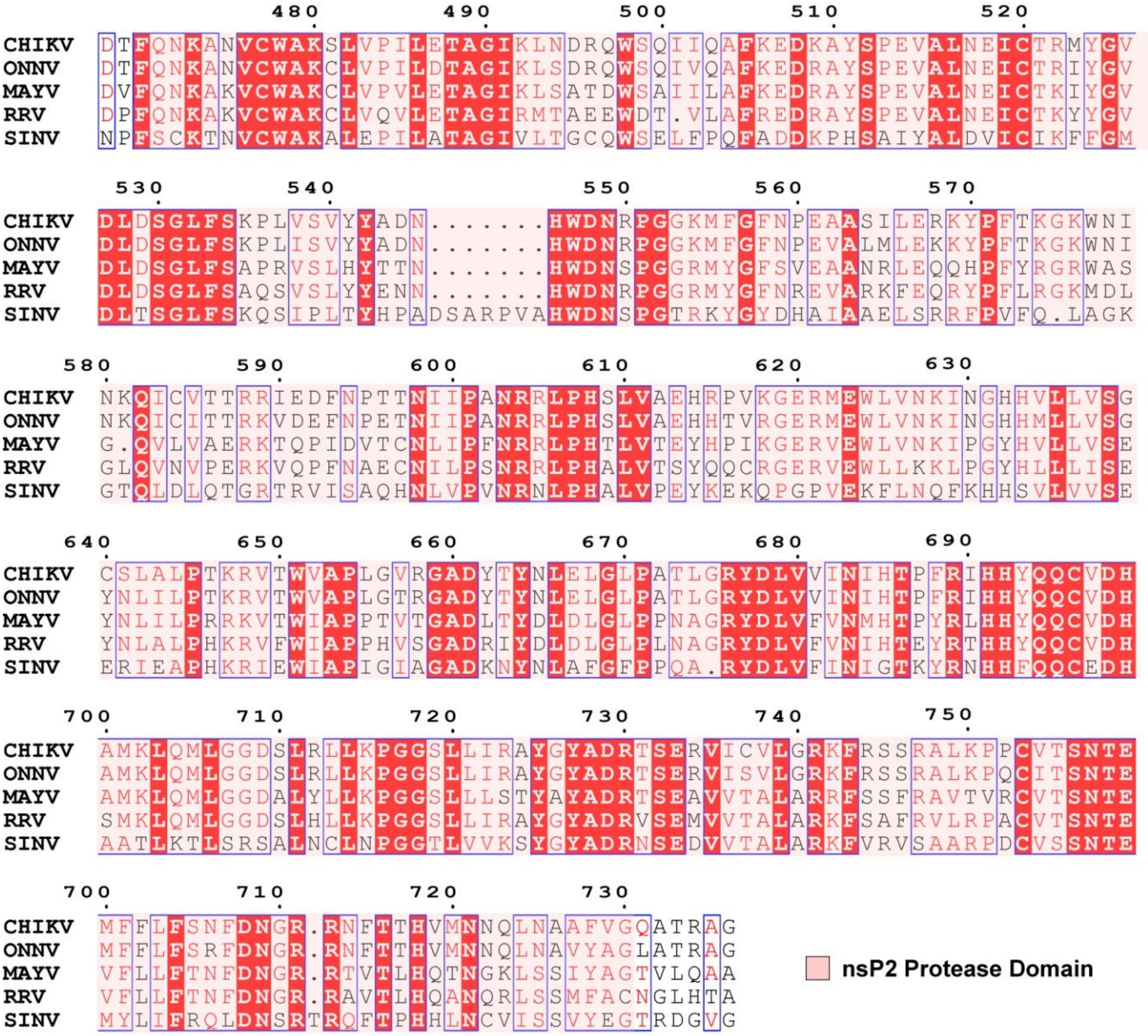

Figure S3. Sequence alignment of the nsP2 protease domain from alphaviruses CHIKV (181/25), ONNV (UgMP30), MAYV (Guyane), RRV (Rarotonga), and SINV (80-2449).

Spectral Data

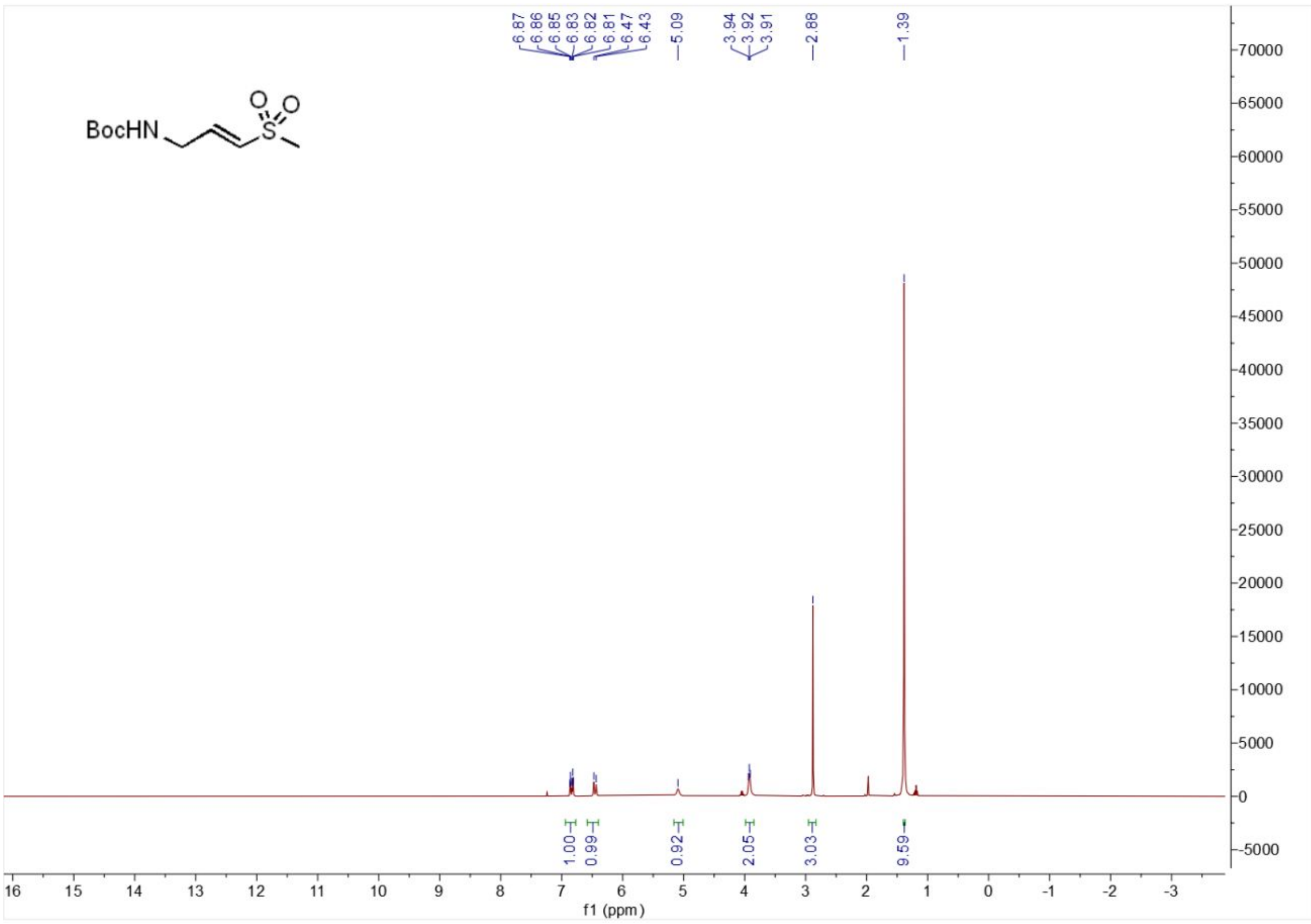

^{1}H NMR spectra of **13**

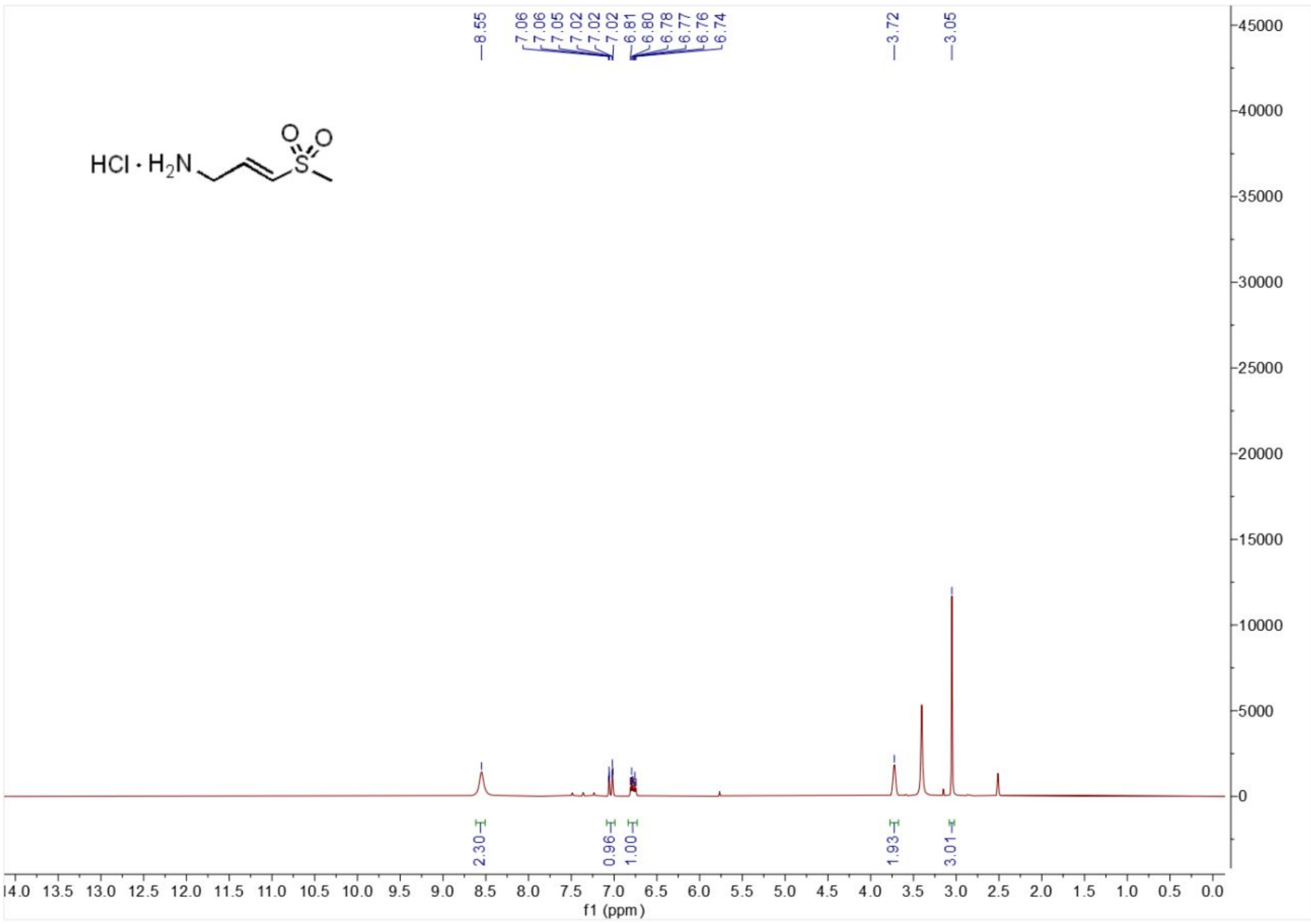

^{1}H NMR spectra of **14**

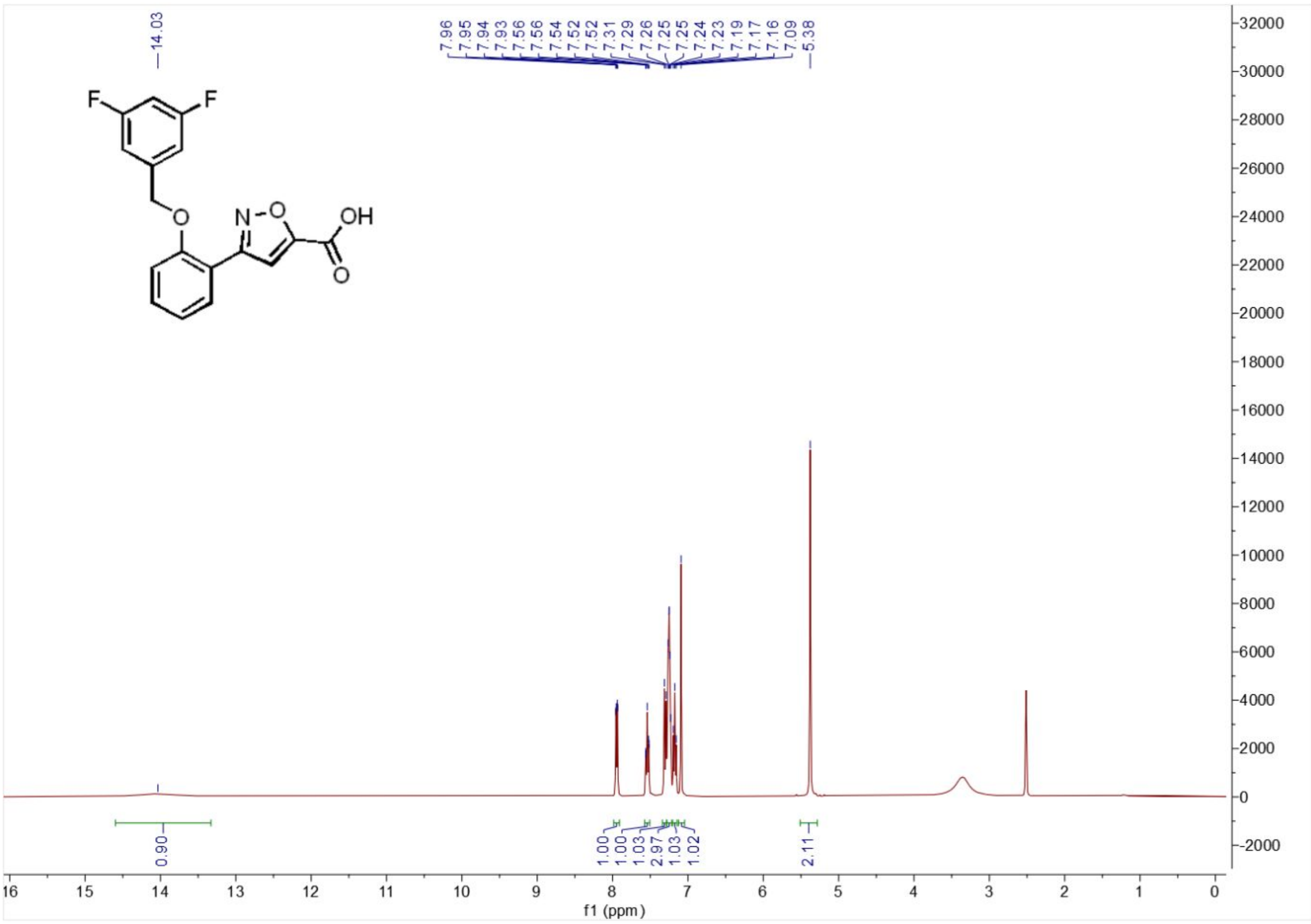

^{1}H NMR spectra of **6a**

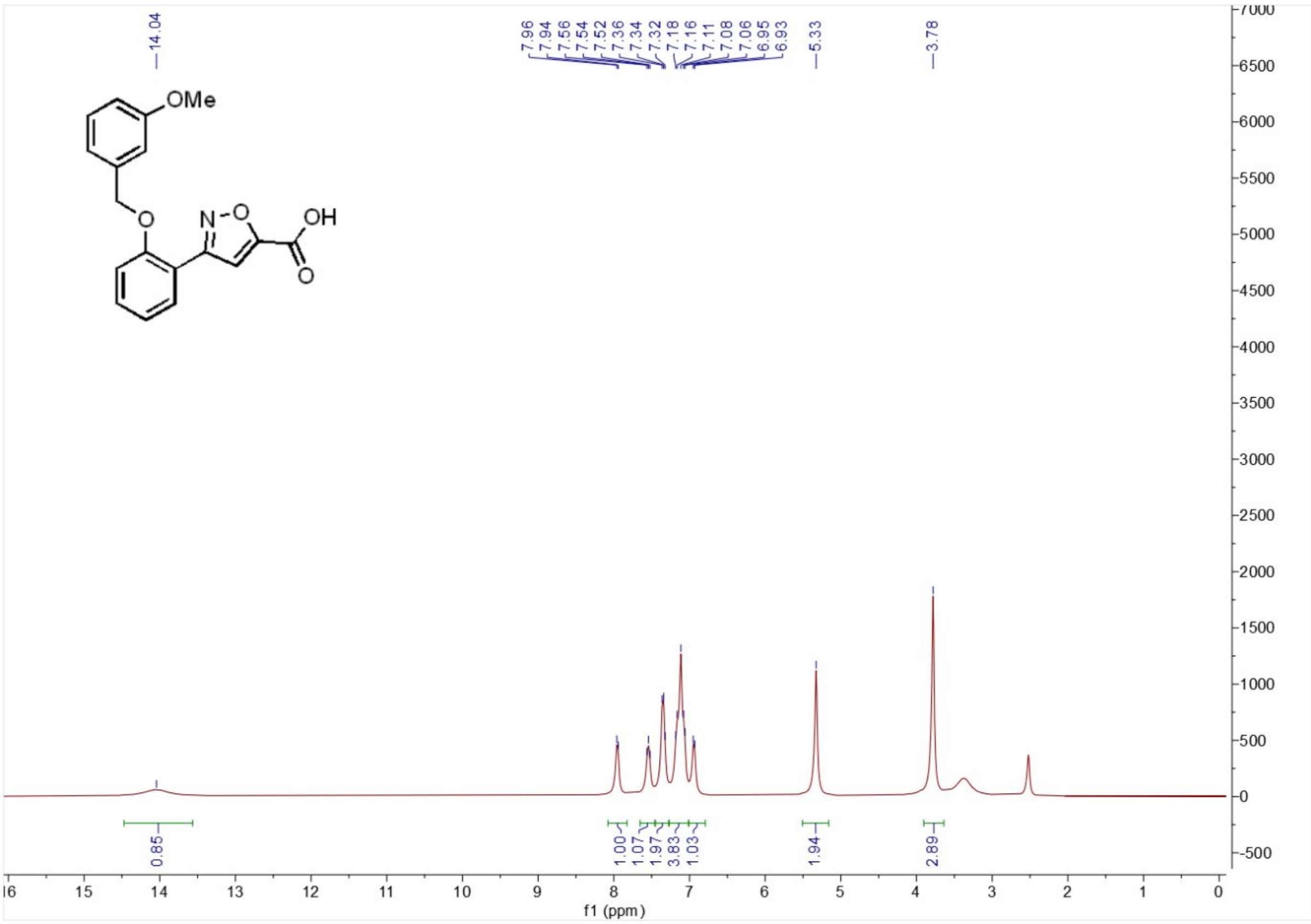

^{1}H NMR spectra of **6b**

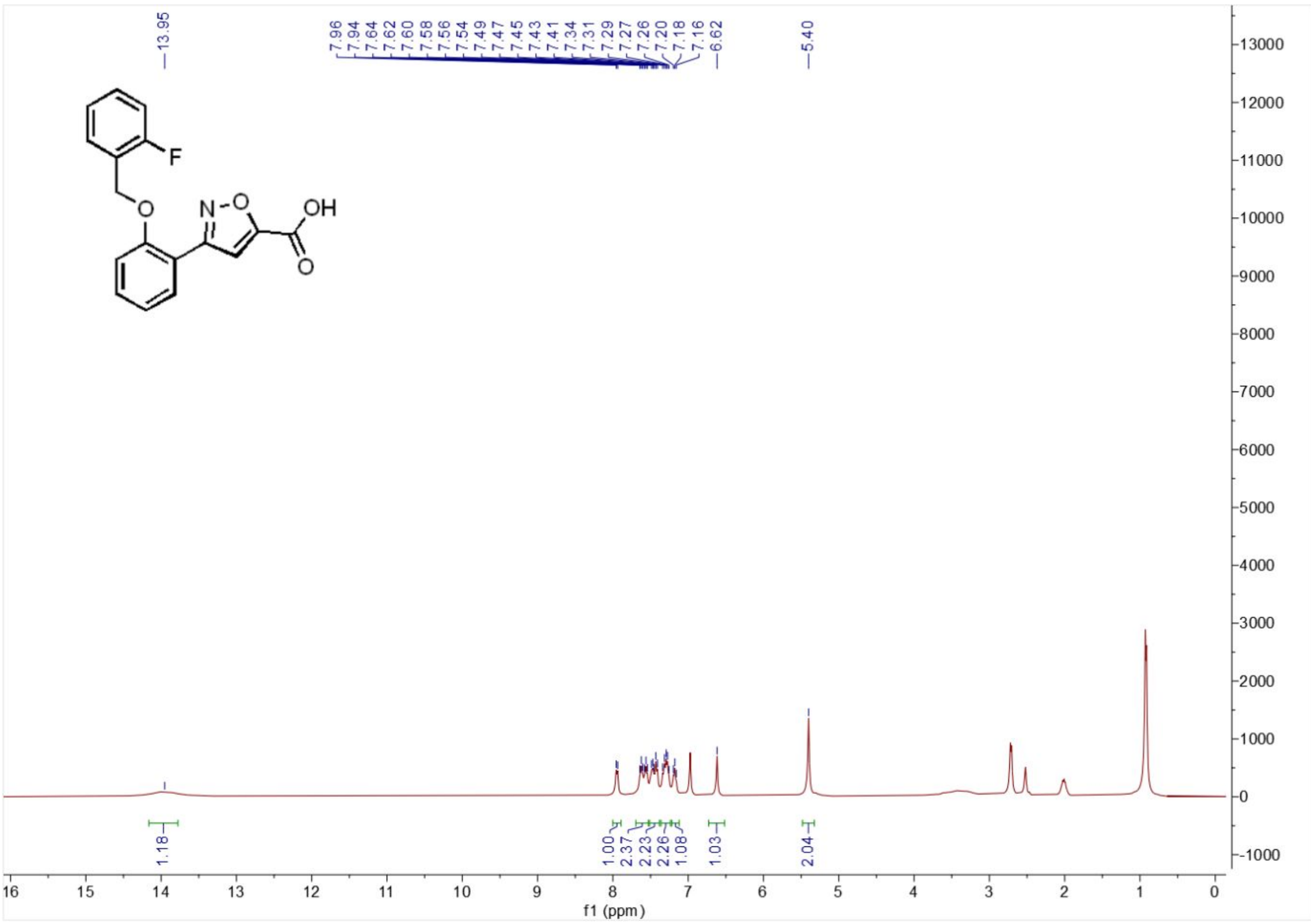

^{1}H NMR spectra of **6c**

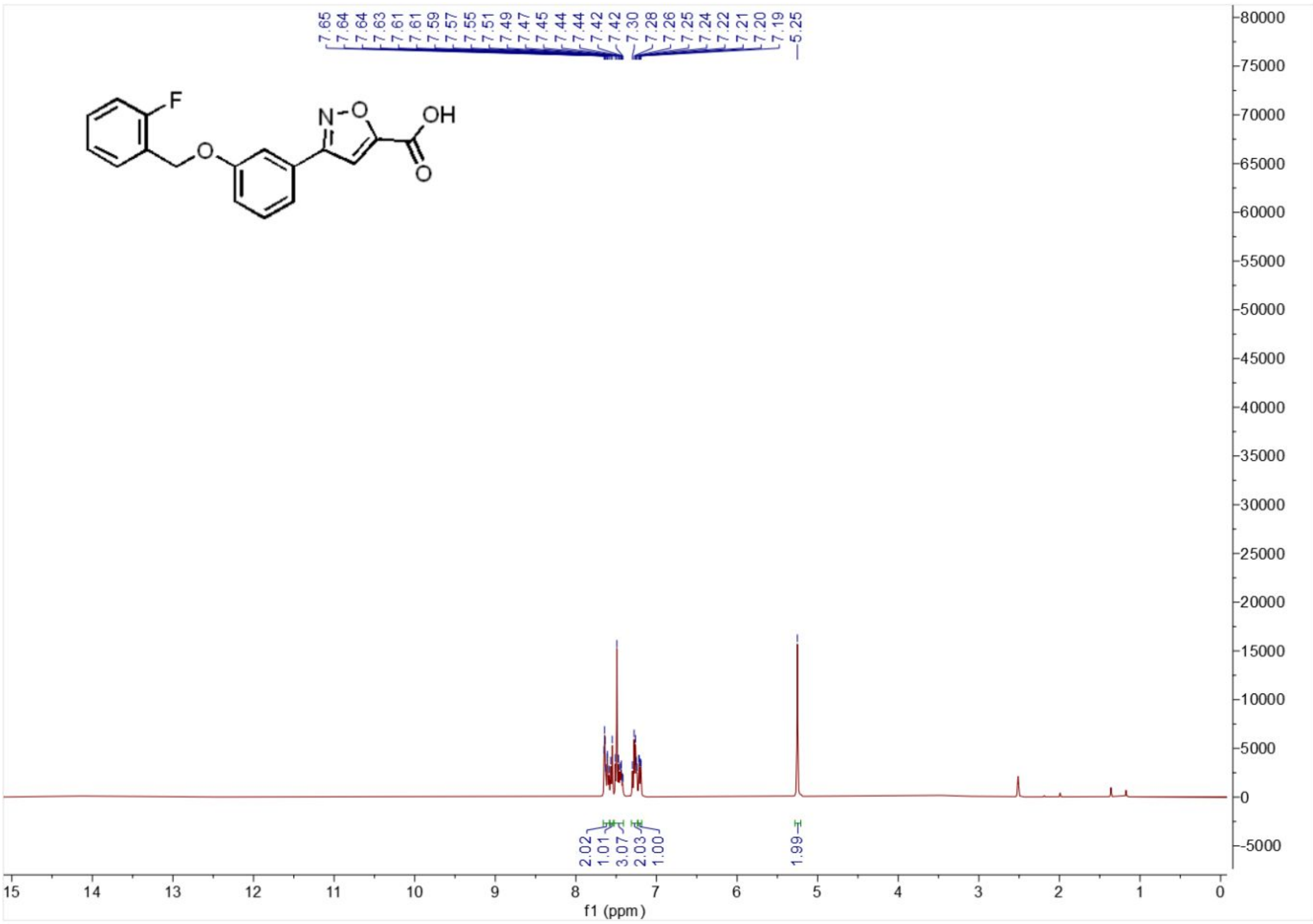

^{1}H NMR spectra of **6d**

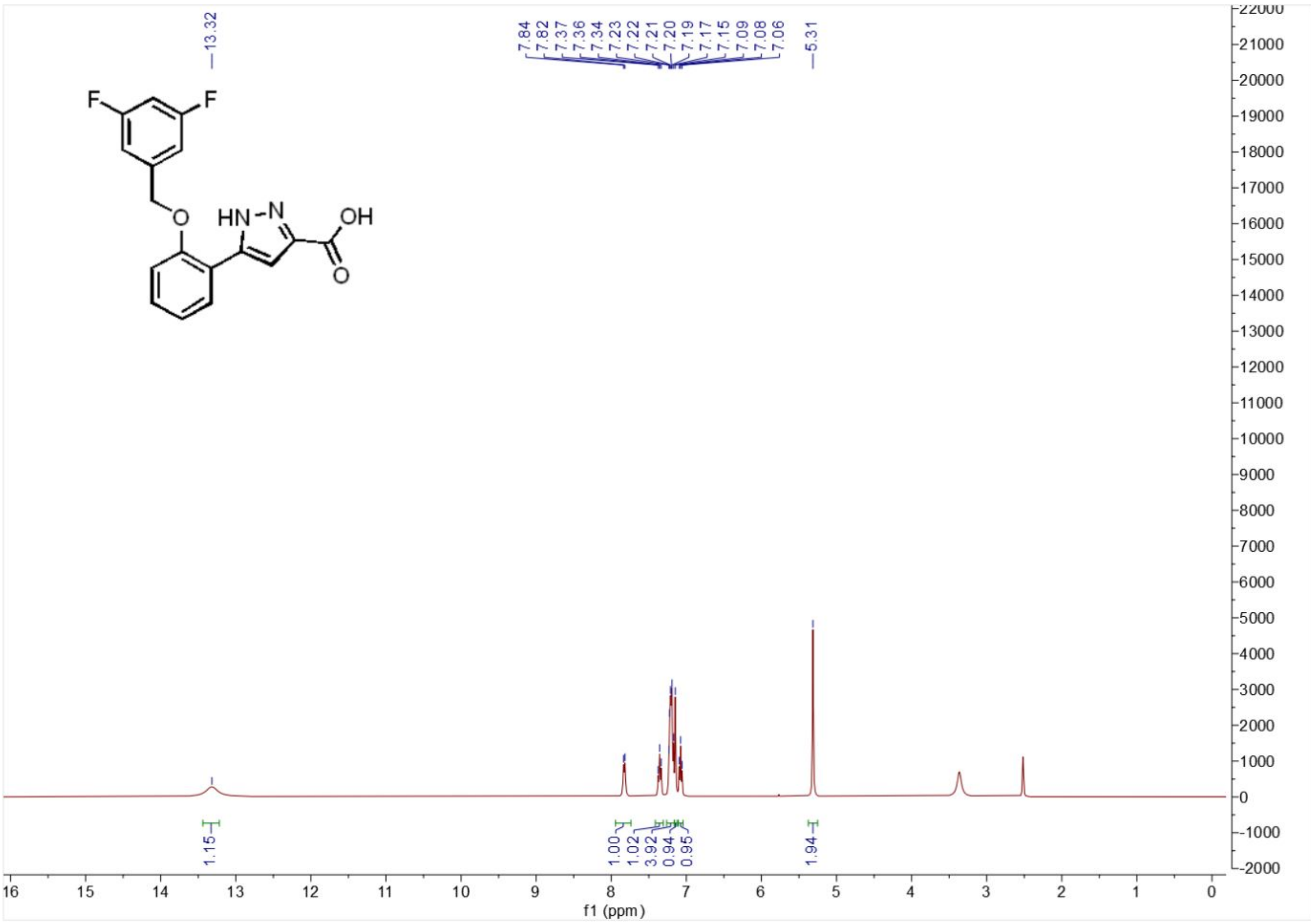

^{1}H NMR spectra of **6a’**

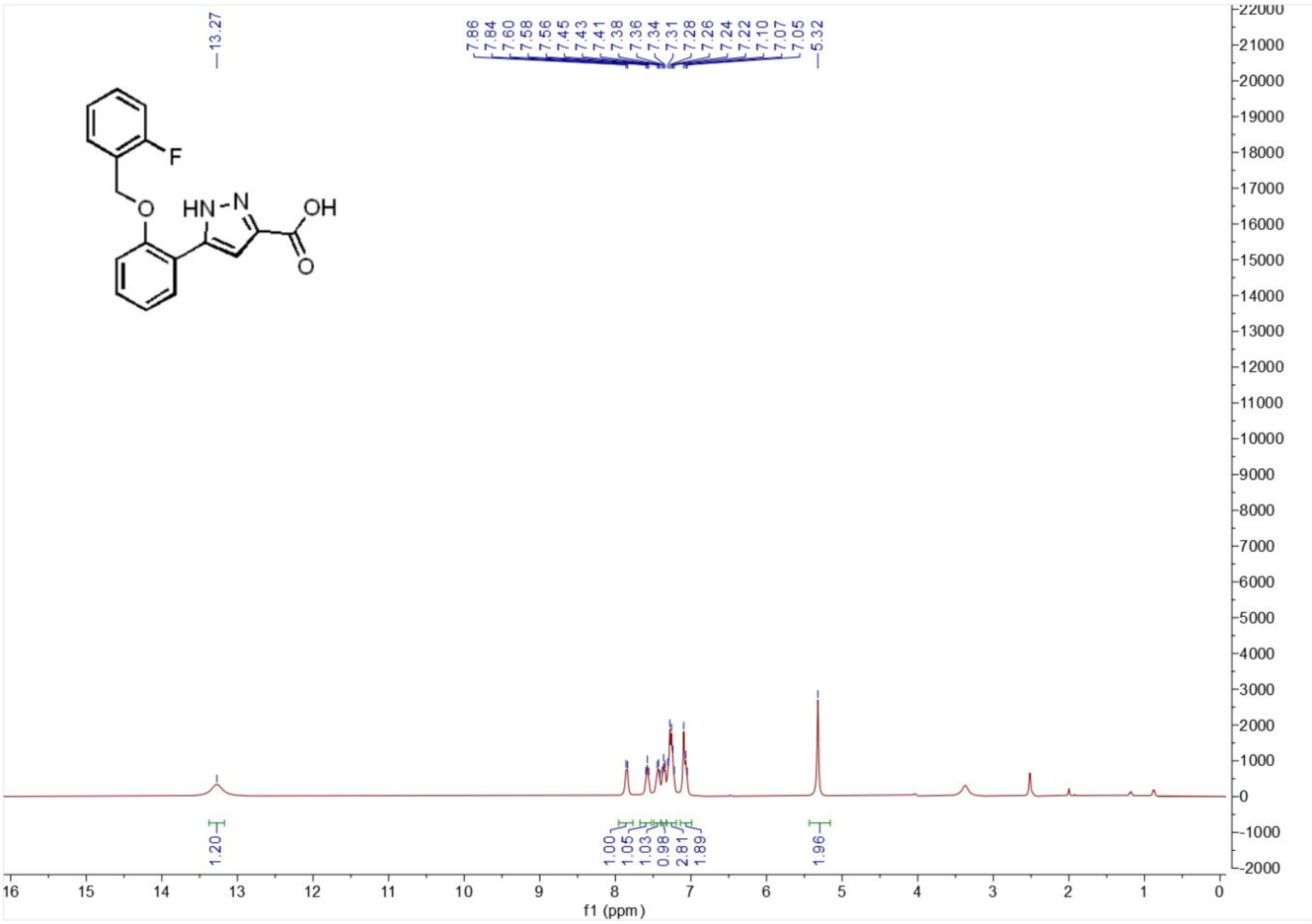

^{1}H NMR spectra of **6b’**

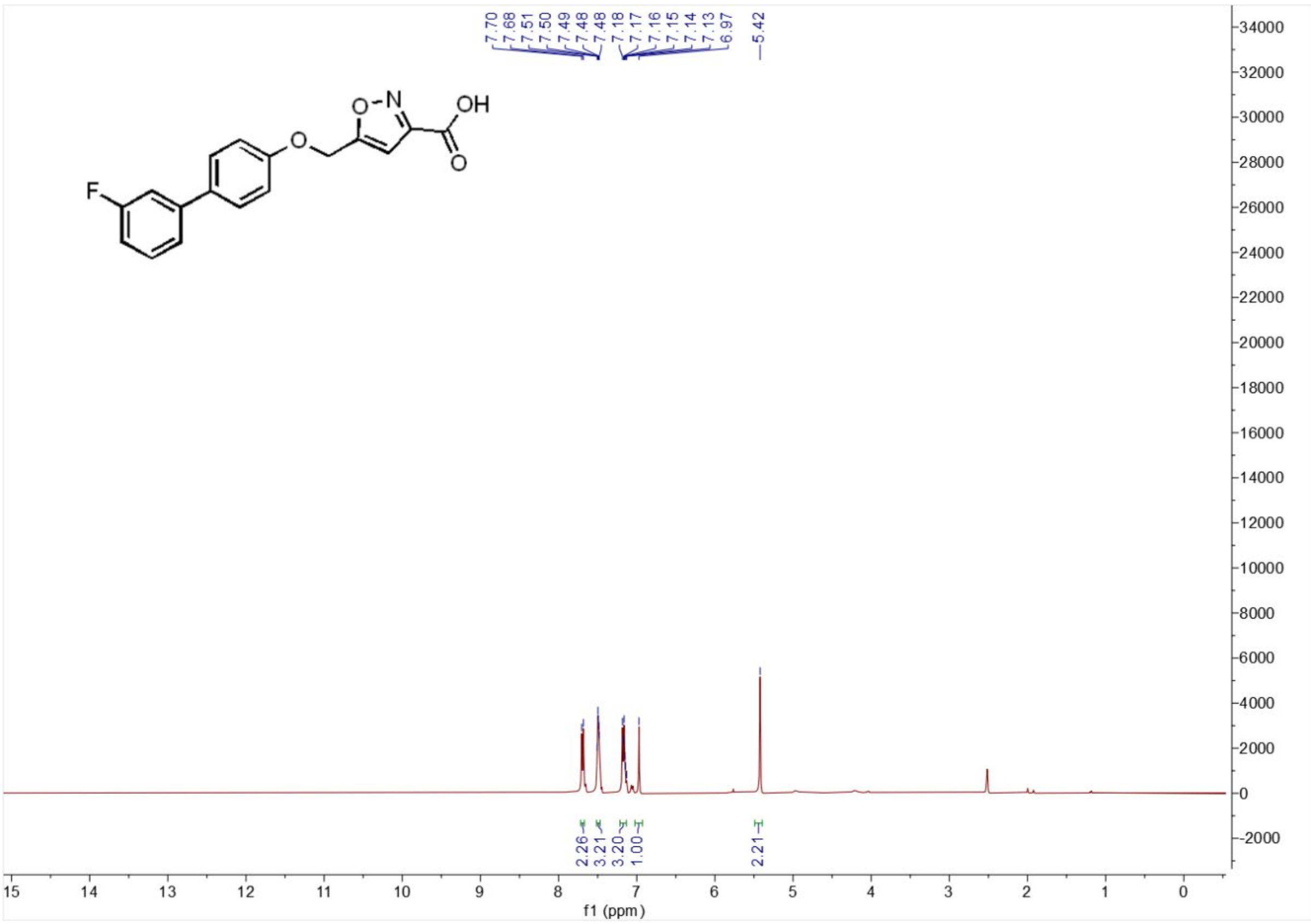

^{1}H NMR spectra of **10a**

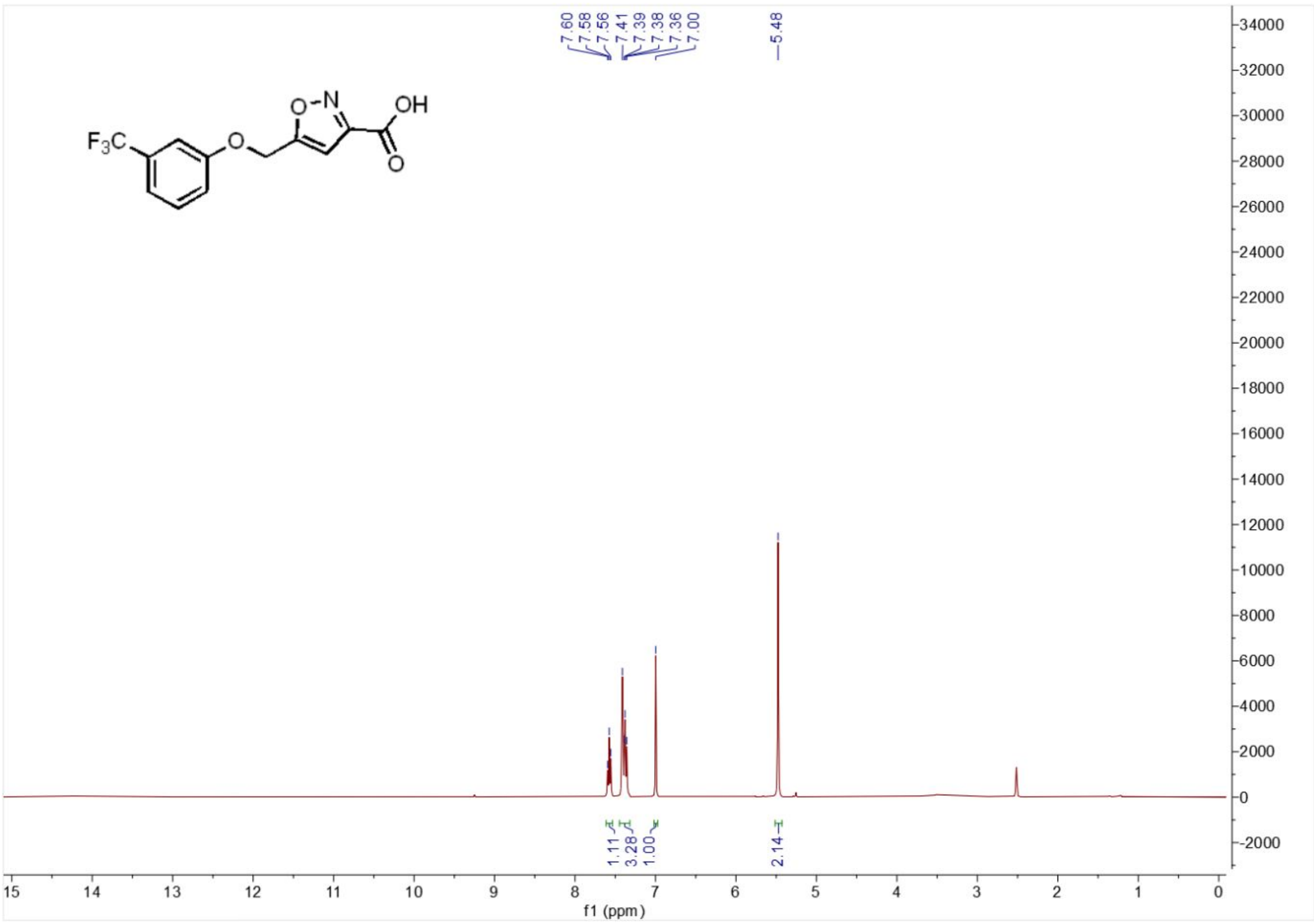

^{1}H NMR spectra of **10b**

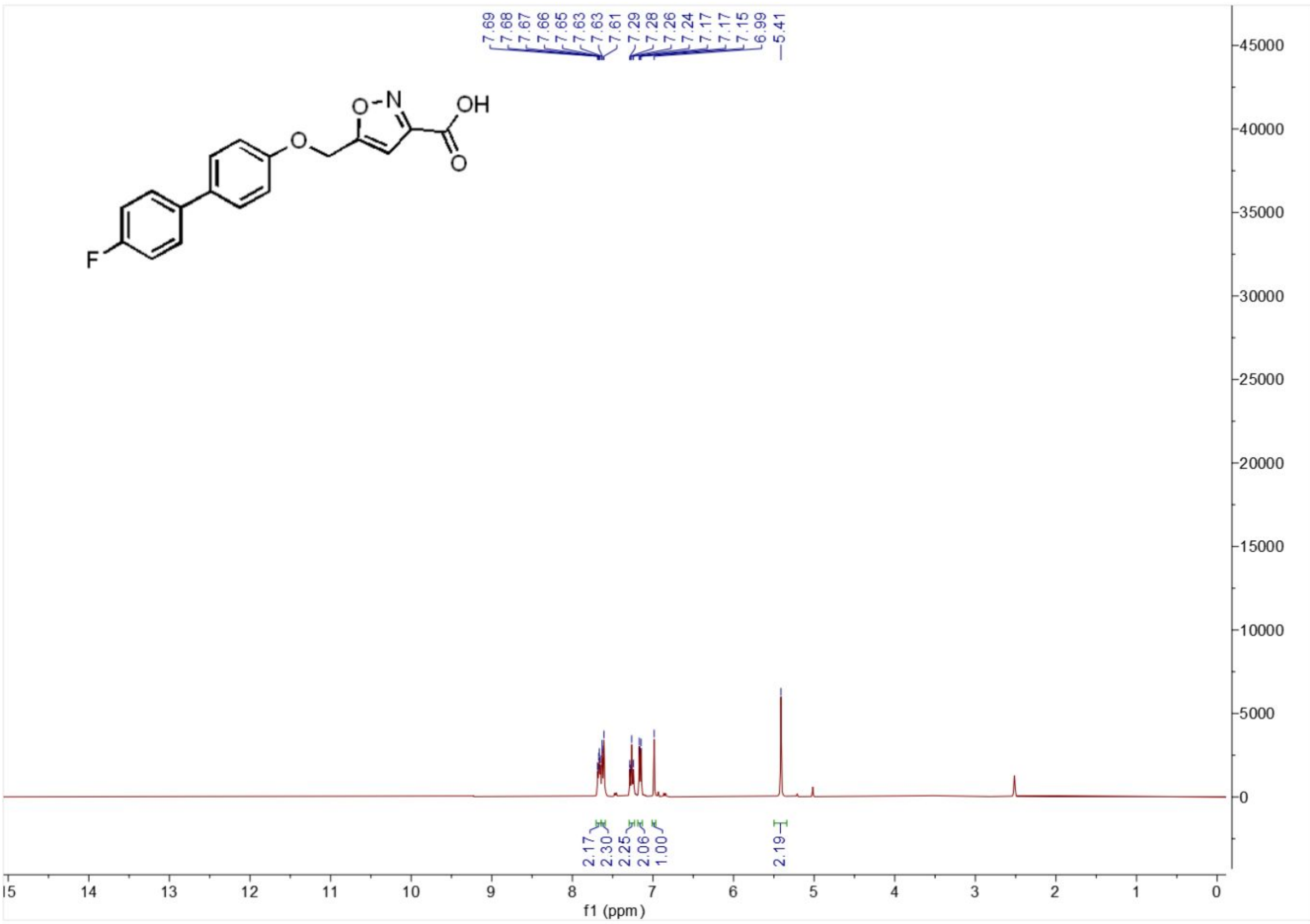

^{1}H NMR spectra of **10c**

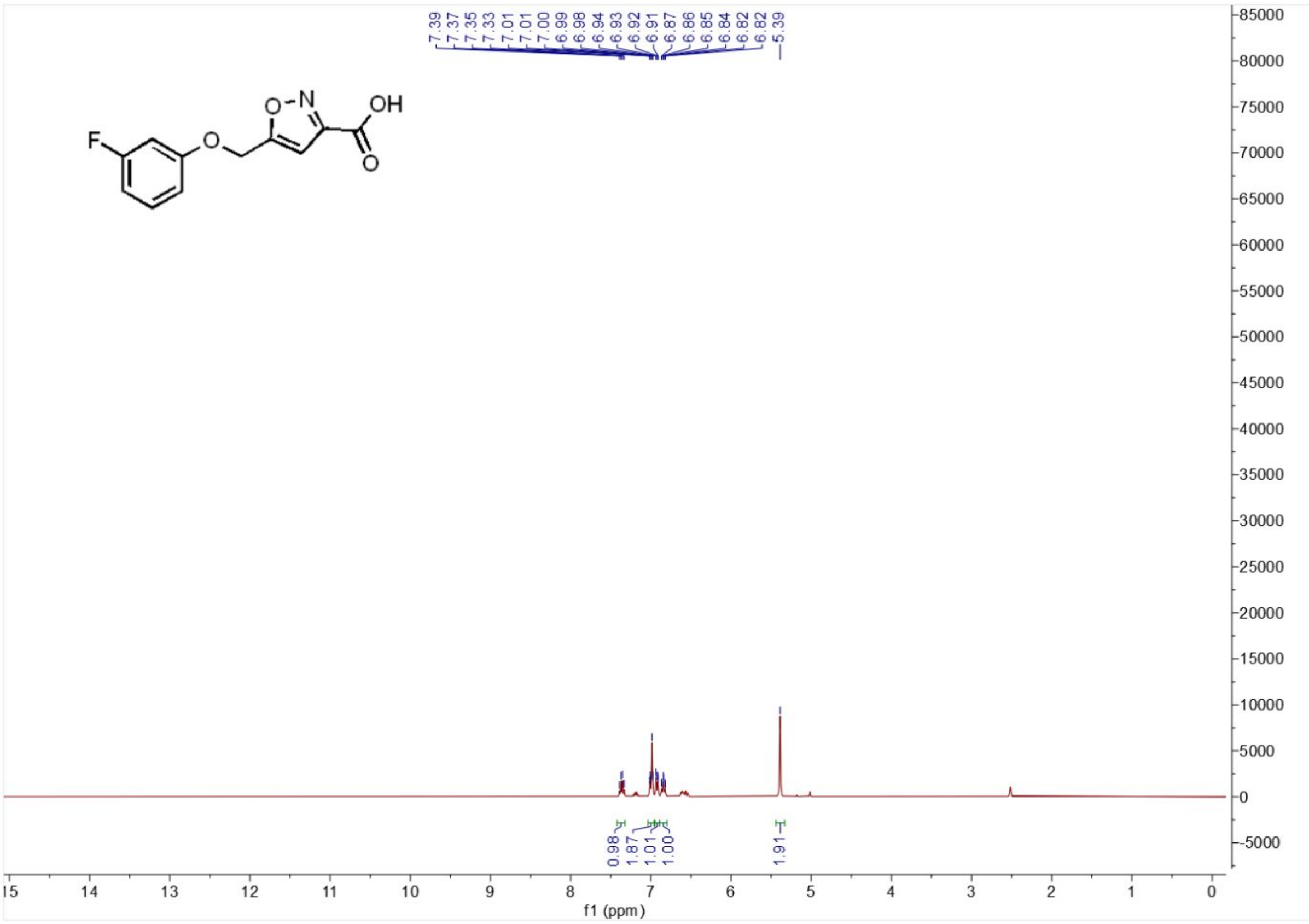

^{1}H NMR spectra of **10d**

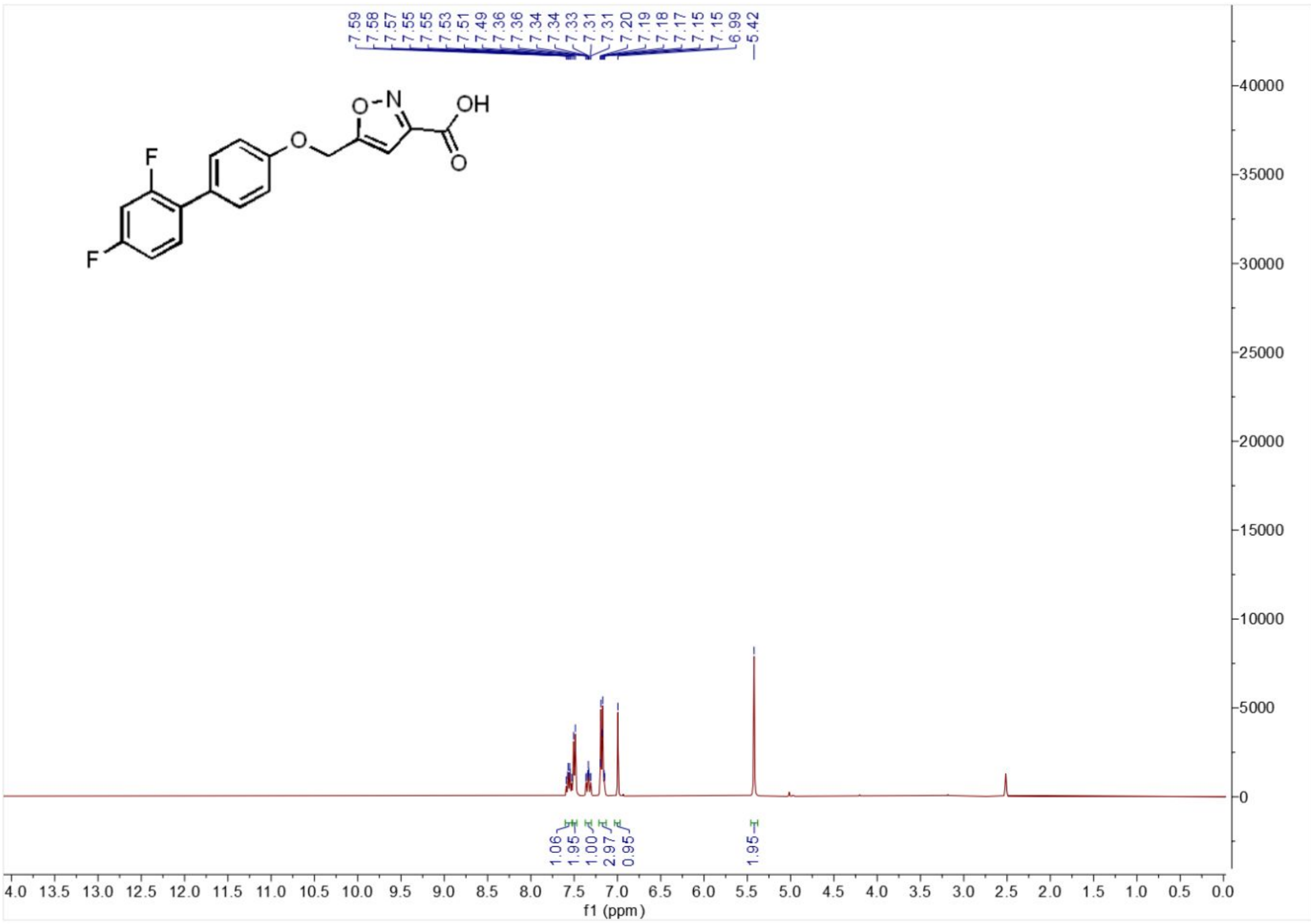

^{1}H NMR spectra of **10e**

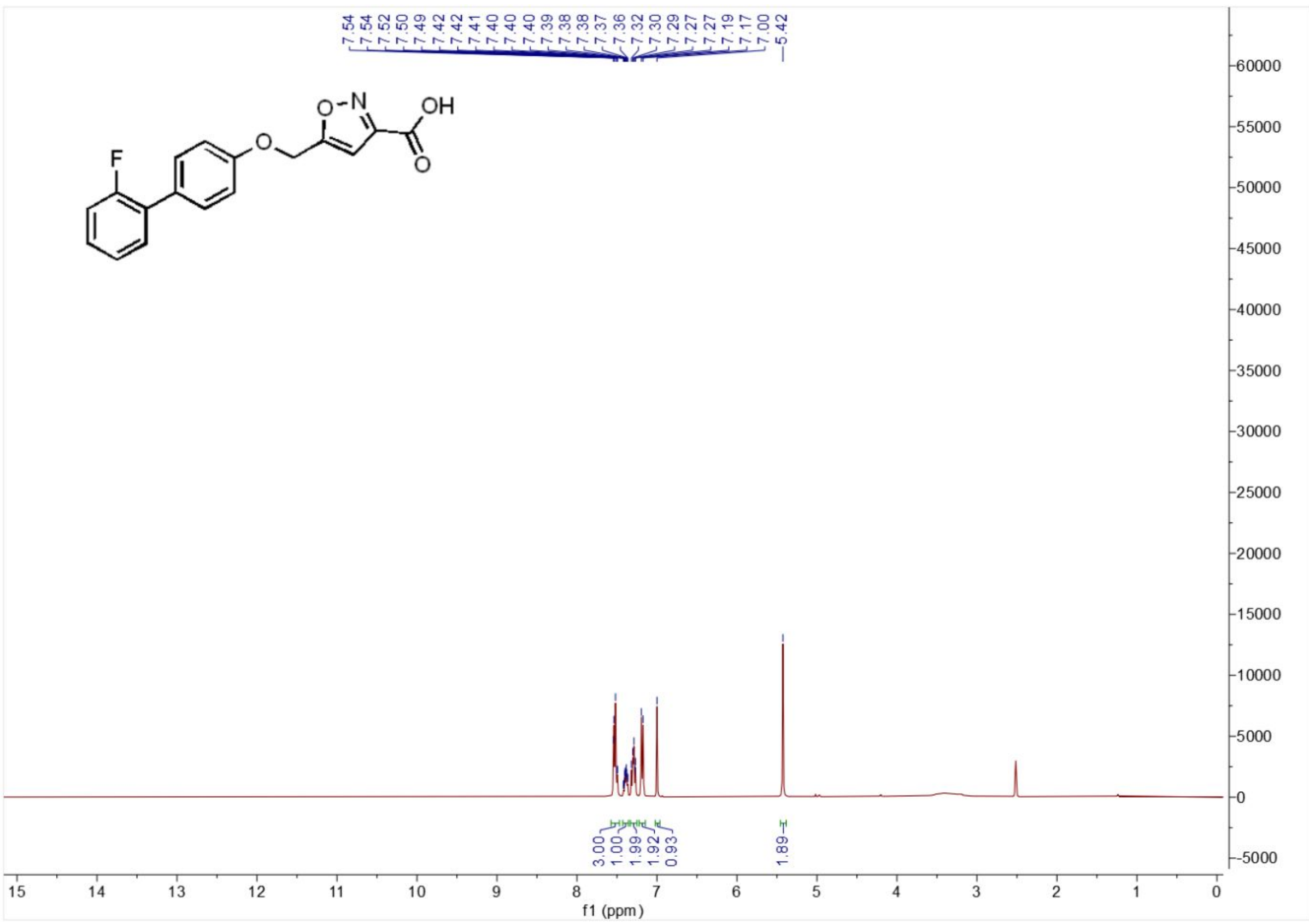

^{1}H NMR spectra of **10f**

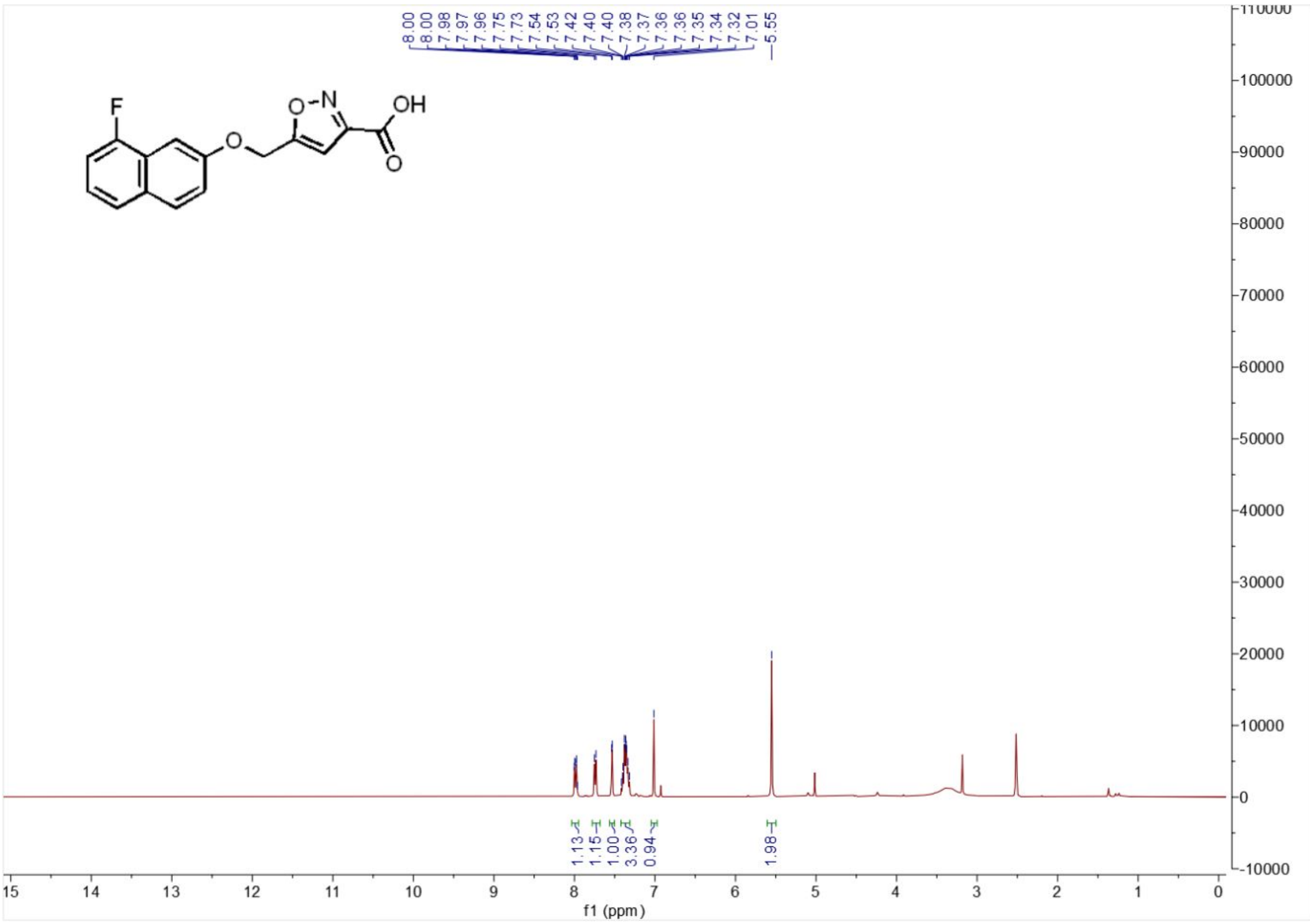

^{1}H NMR spectra of **10g**

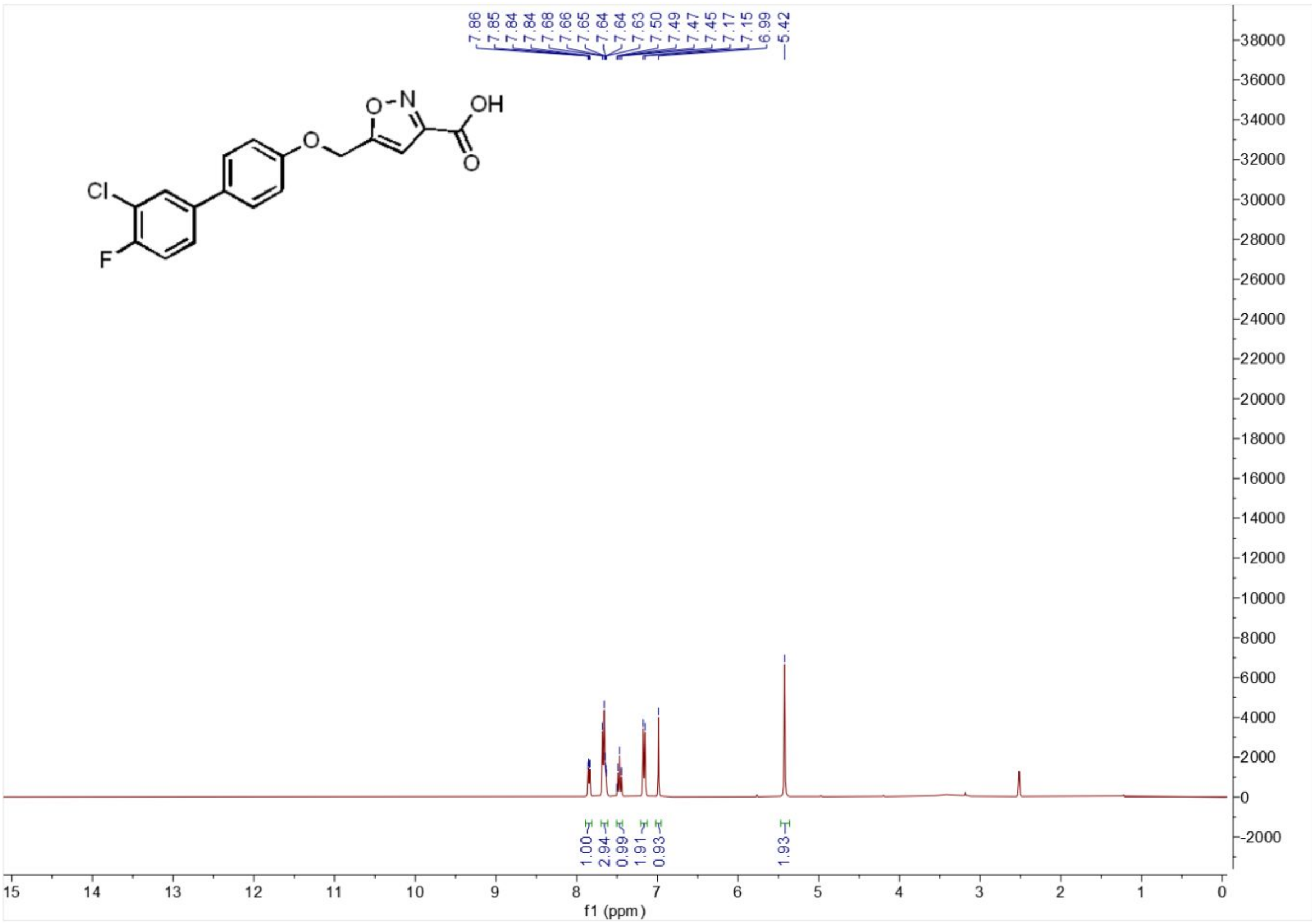

^{1}H NMR spectra of **10h**

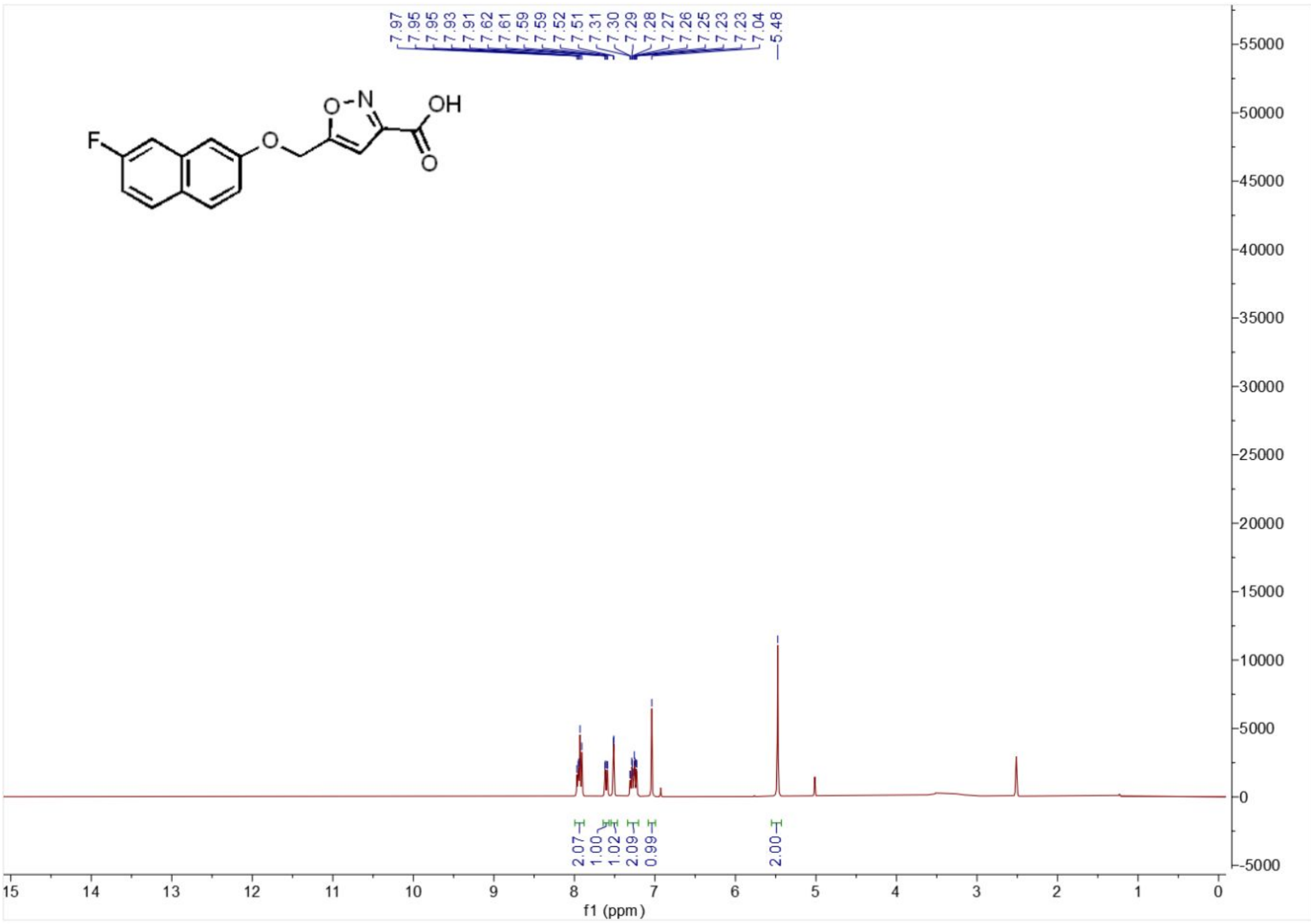

^{1}H NMR spectra of **10i**

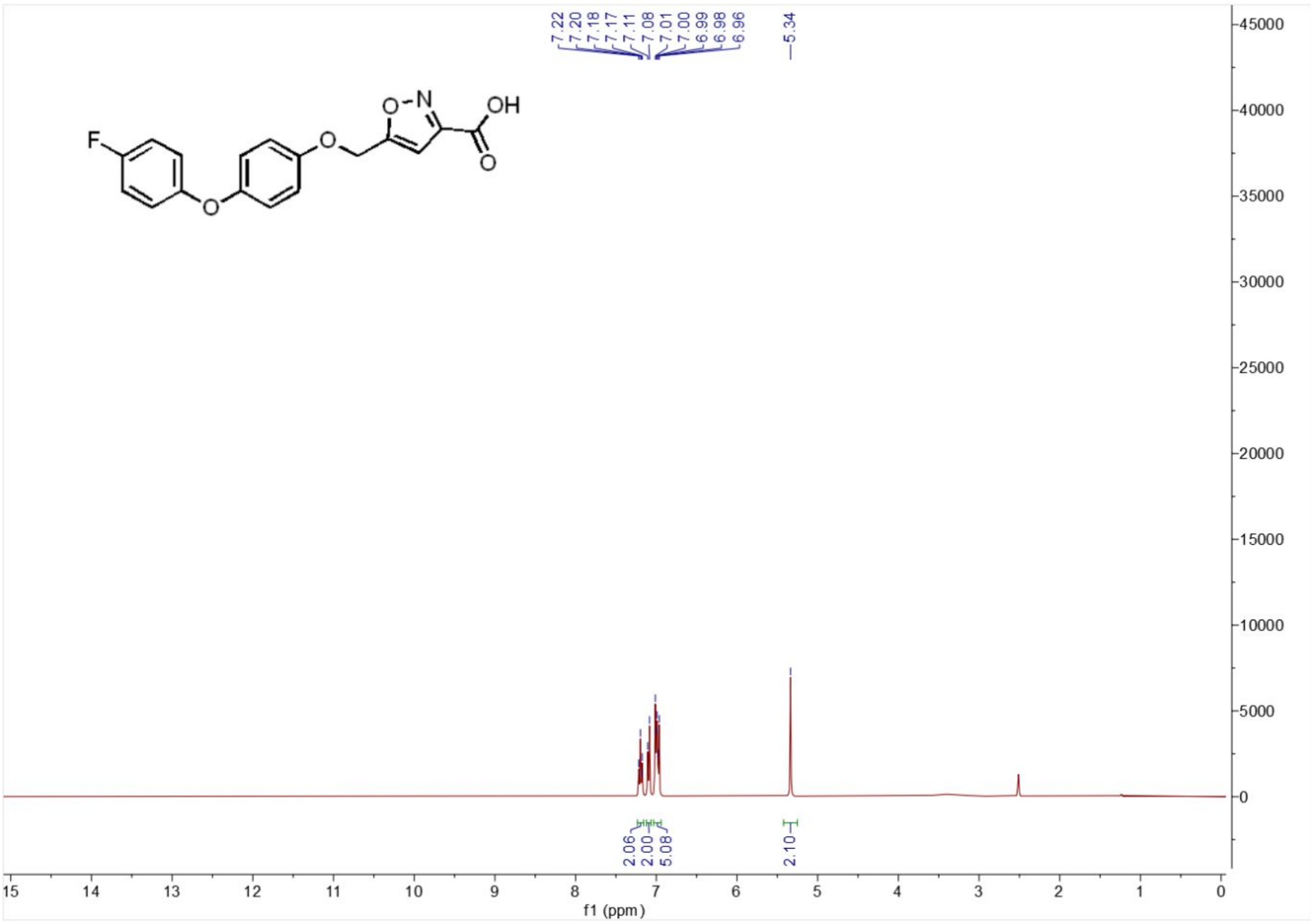

^{1}H NMR spectra of **10j**

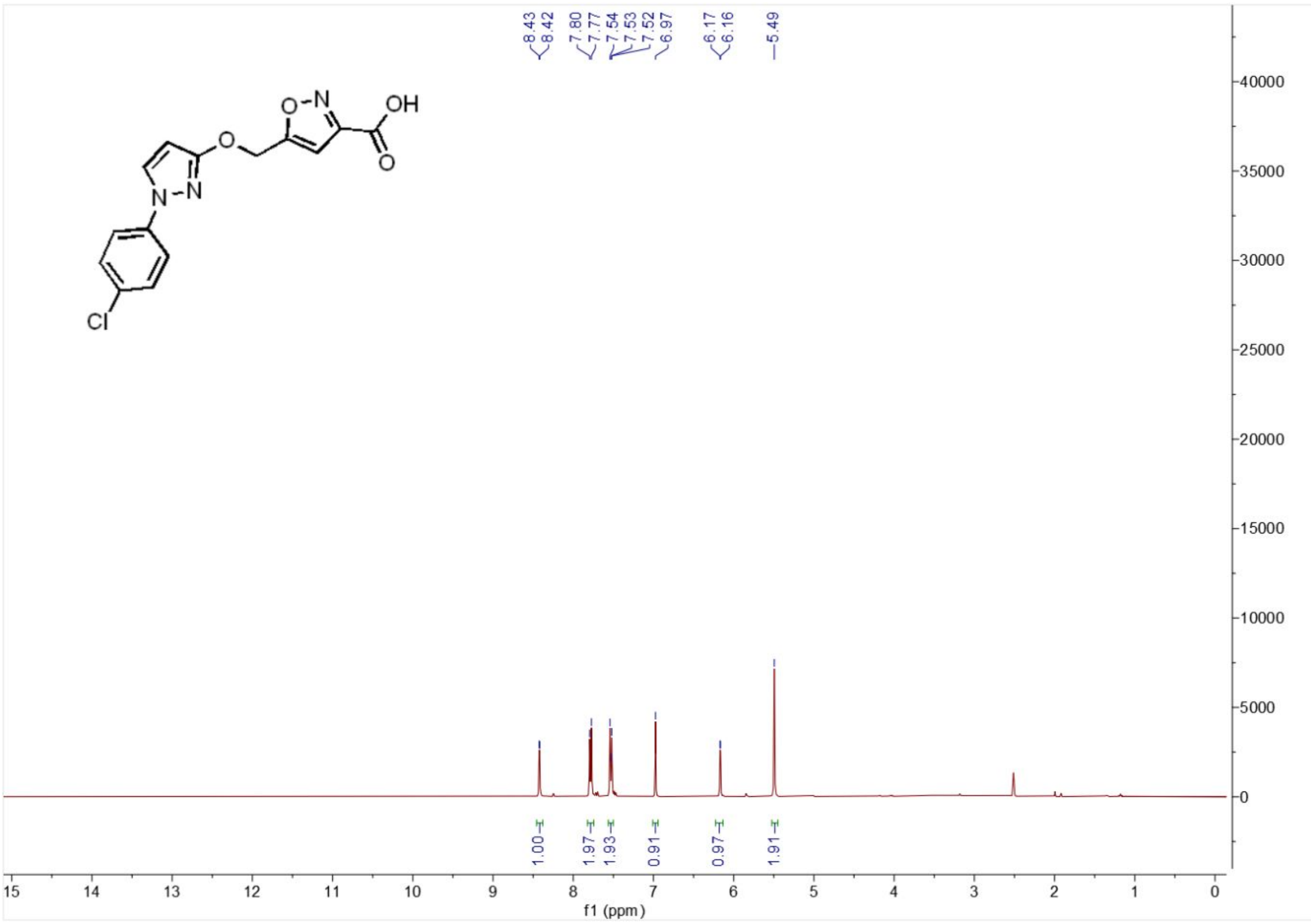

^{1}H NMR spectra of **10k**

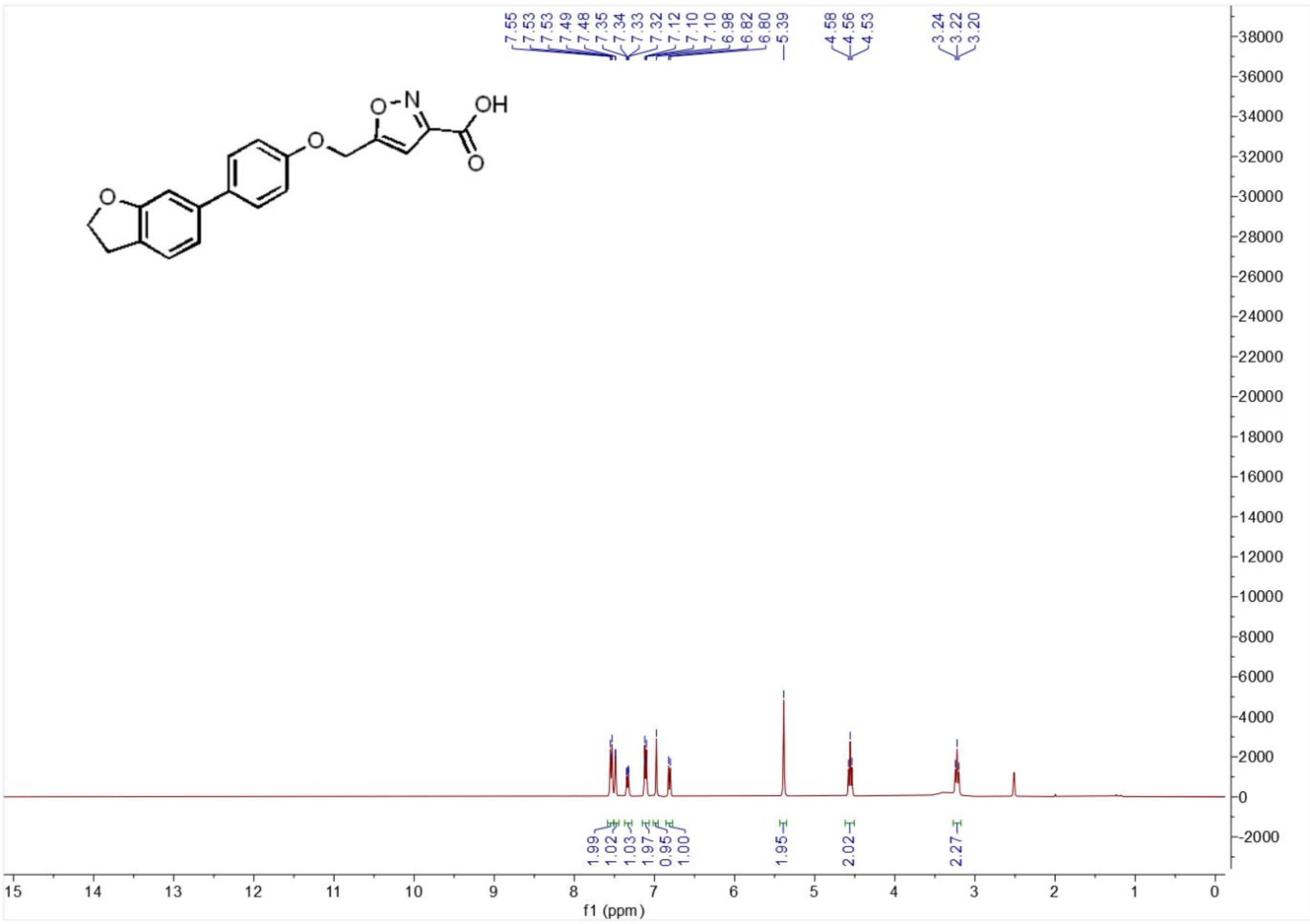

^{1}H NMR spectra of **10l**

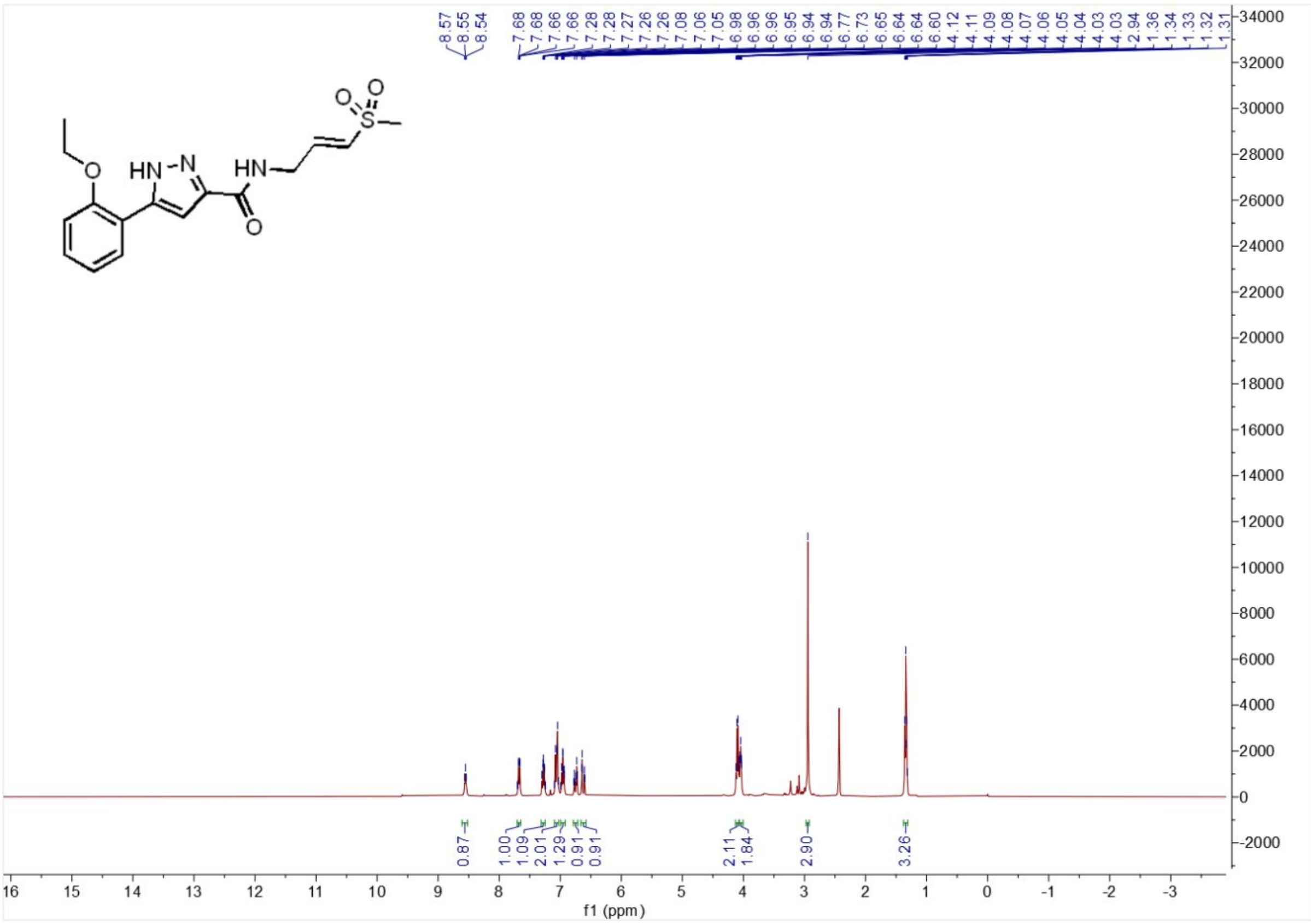

^{1}H NMR spectra of **Jun1423**

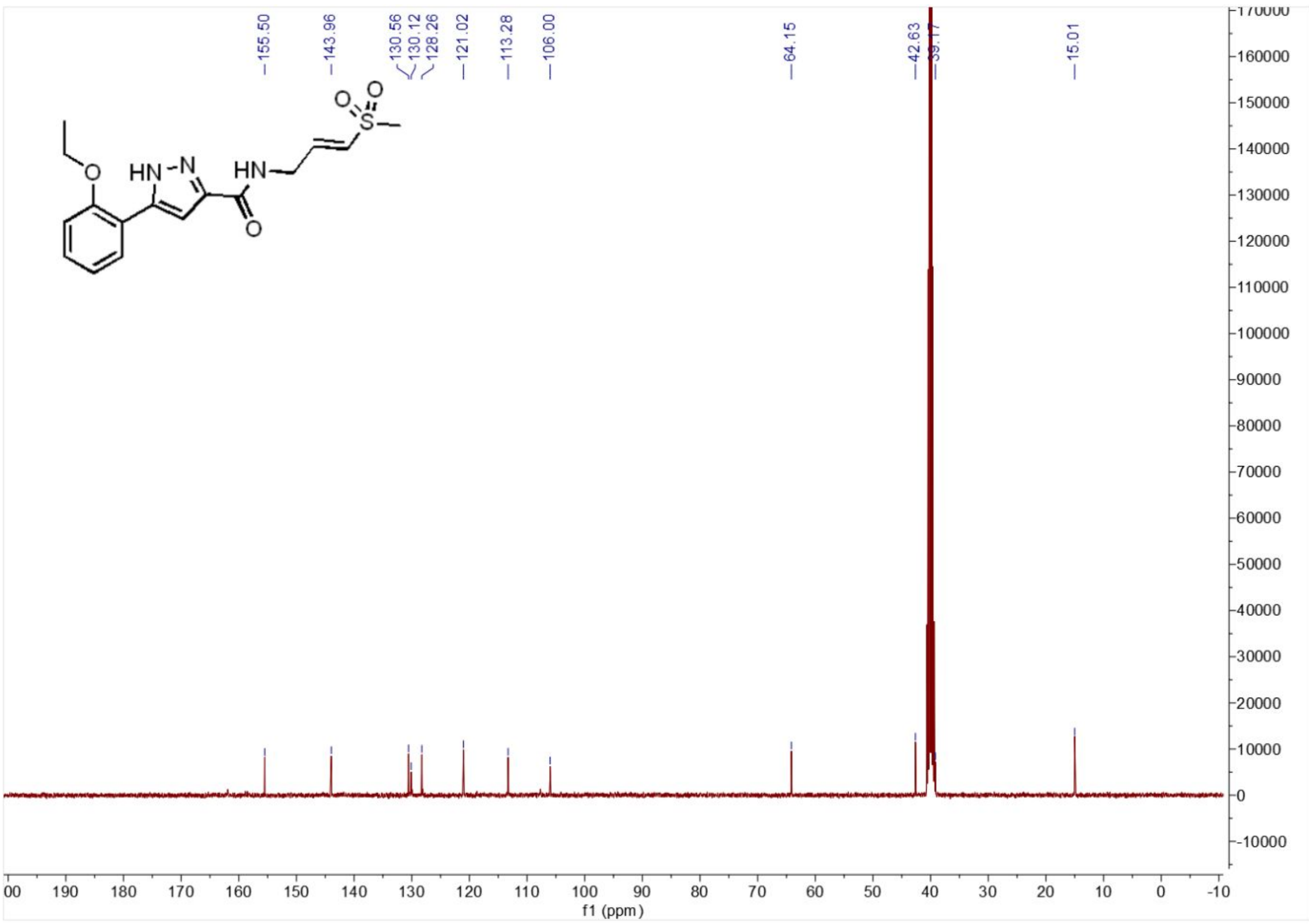

^{13}C NMR spectra of **Jun1423**

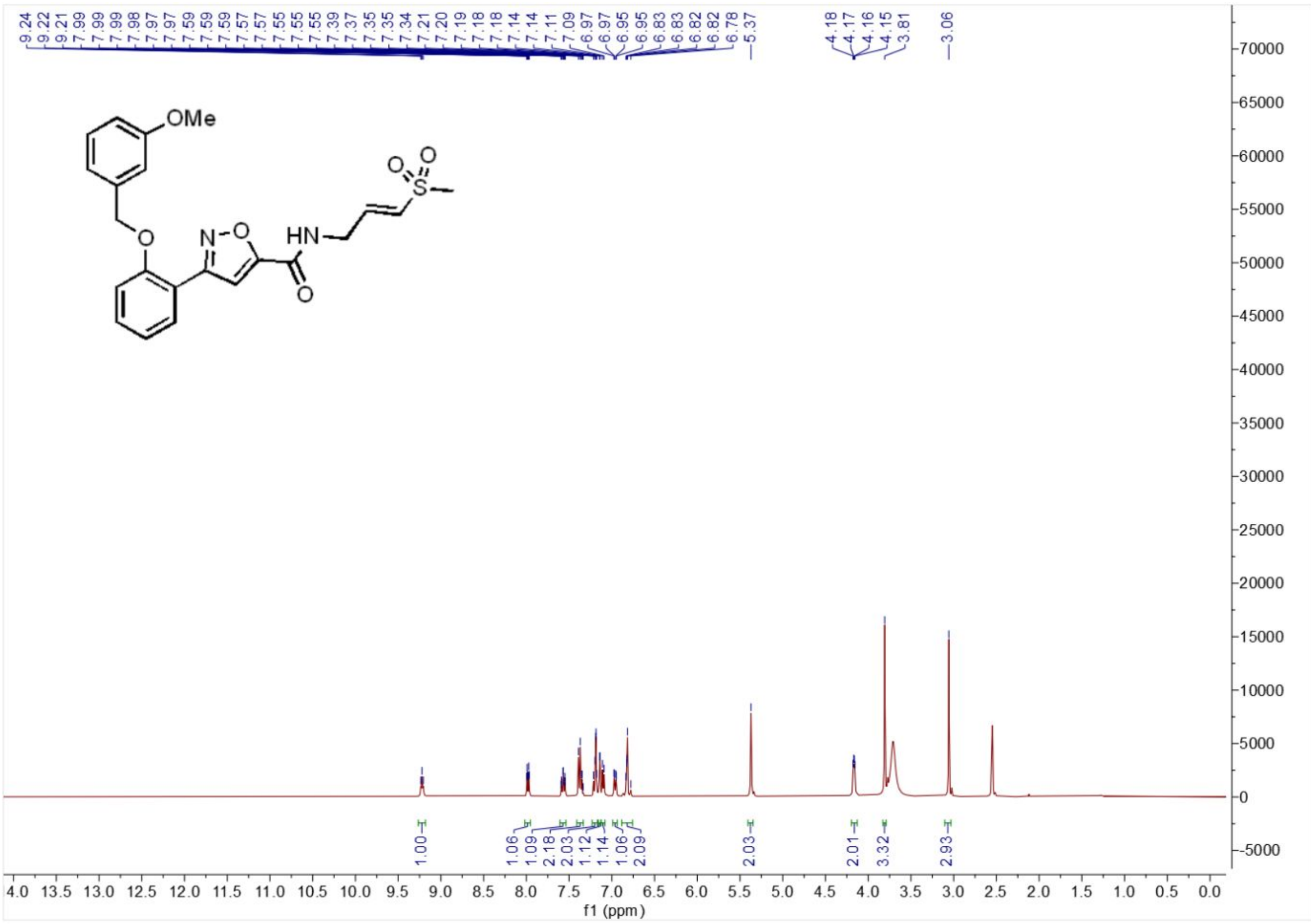

^{1}H NMR spectra of **Jun14435**

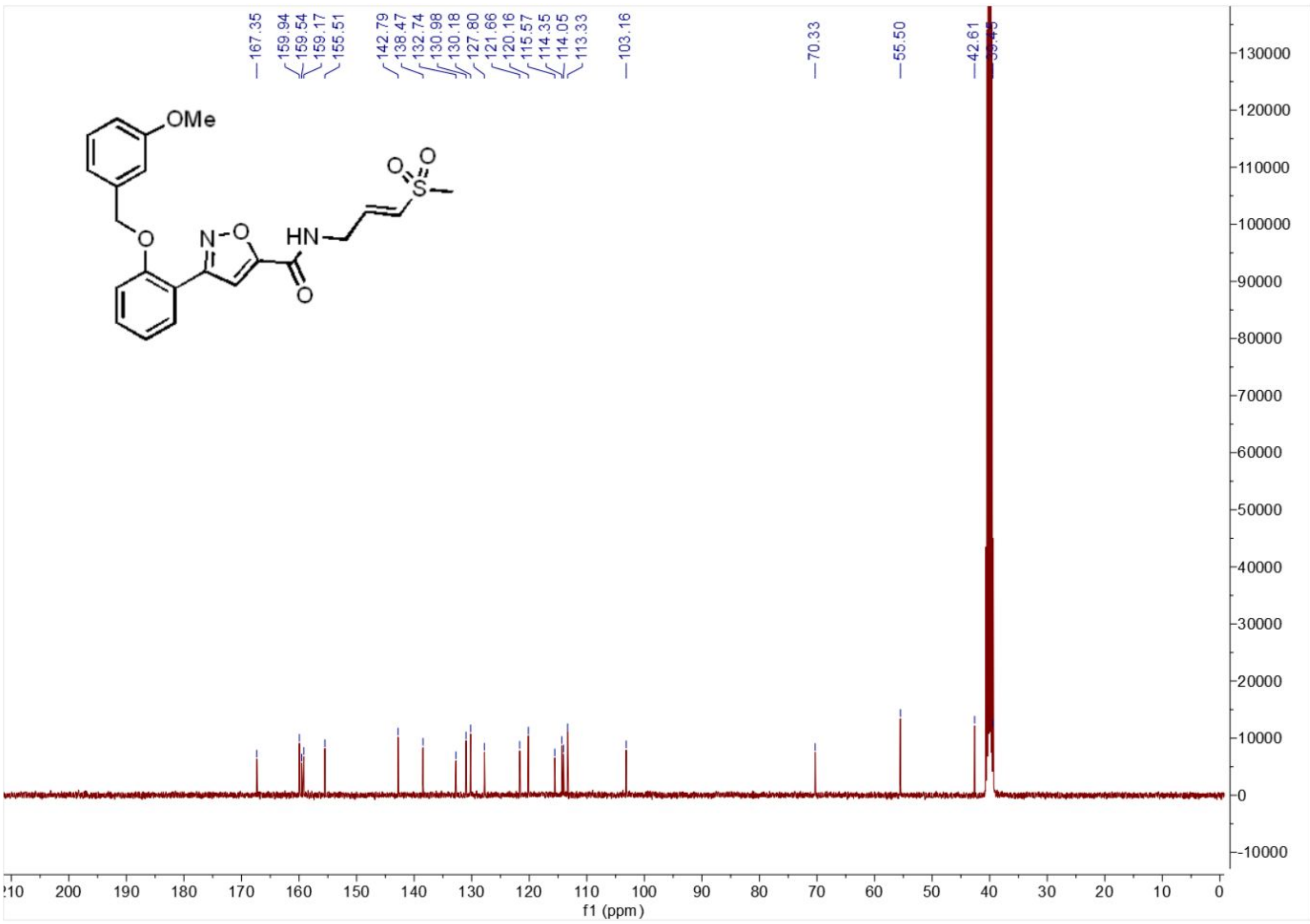

^{13}C NMR spectra of **Jun14435**

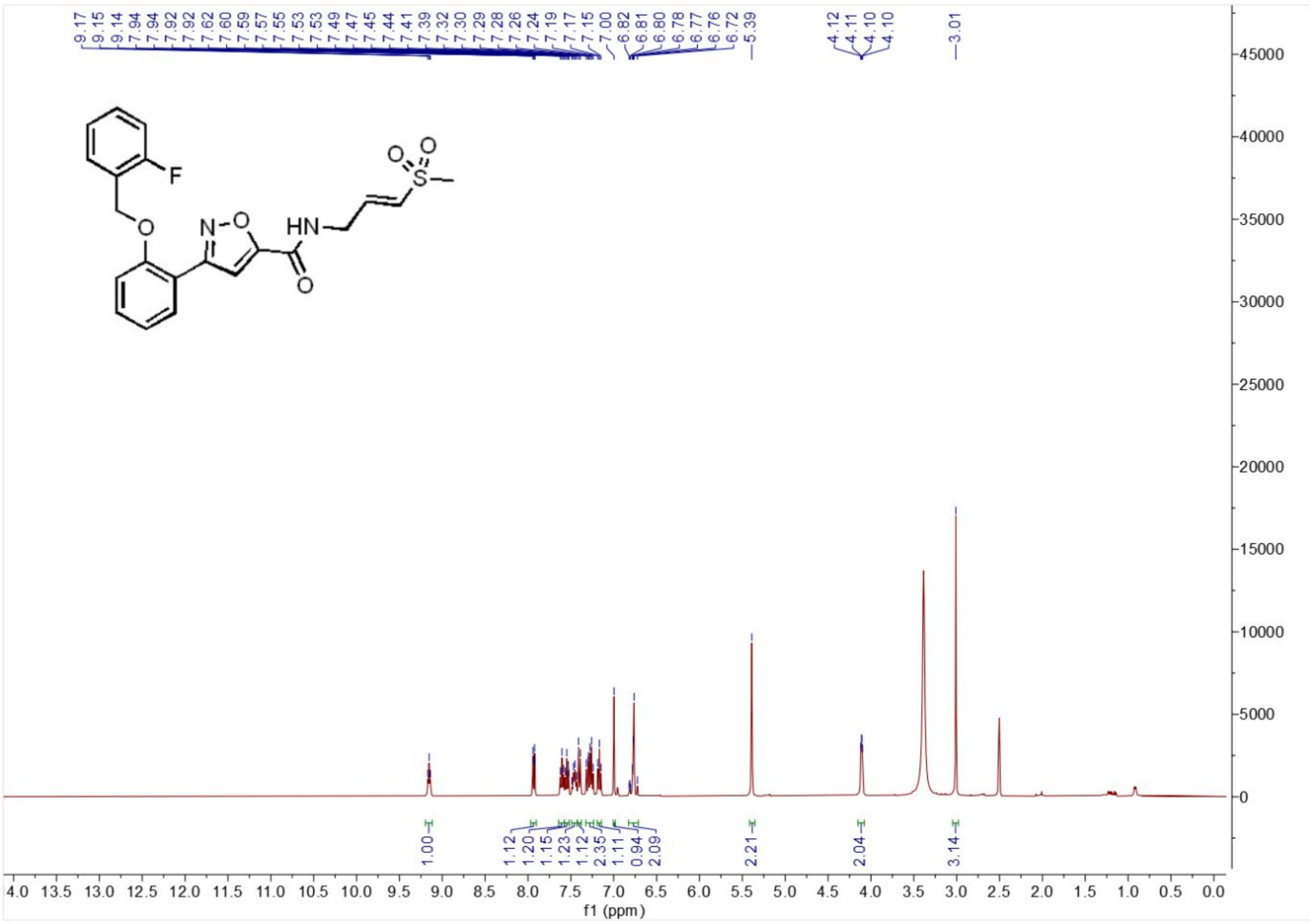

^{1}H NMR spectra of **Jun14454**

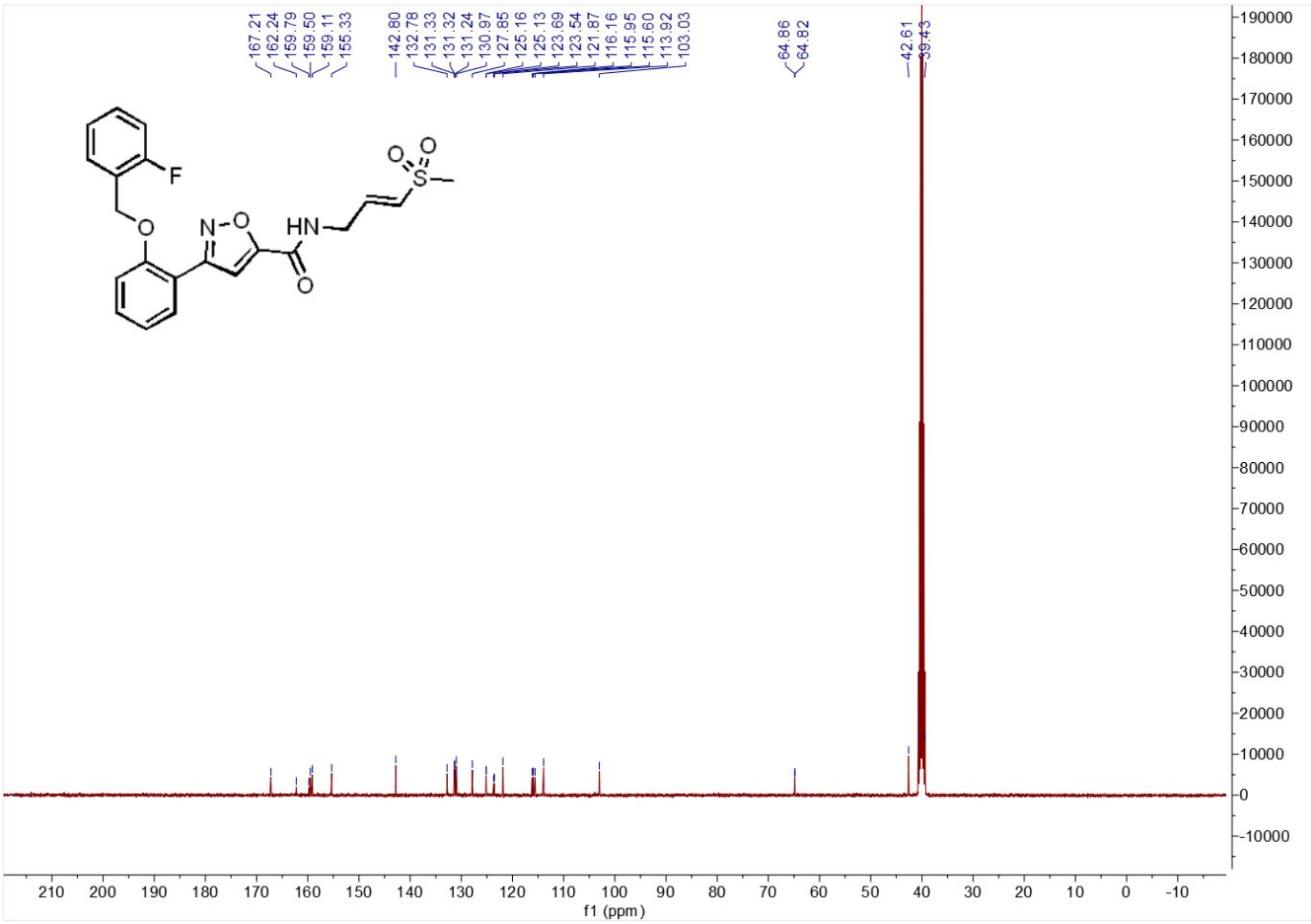

^{13}C NMR spectra of **Jun14454**

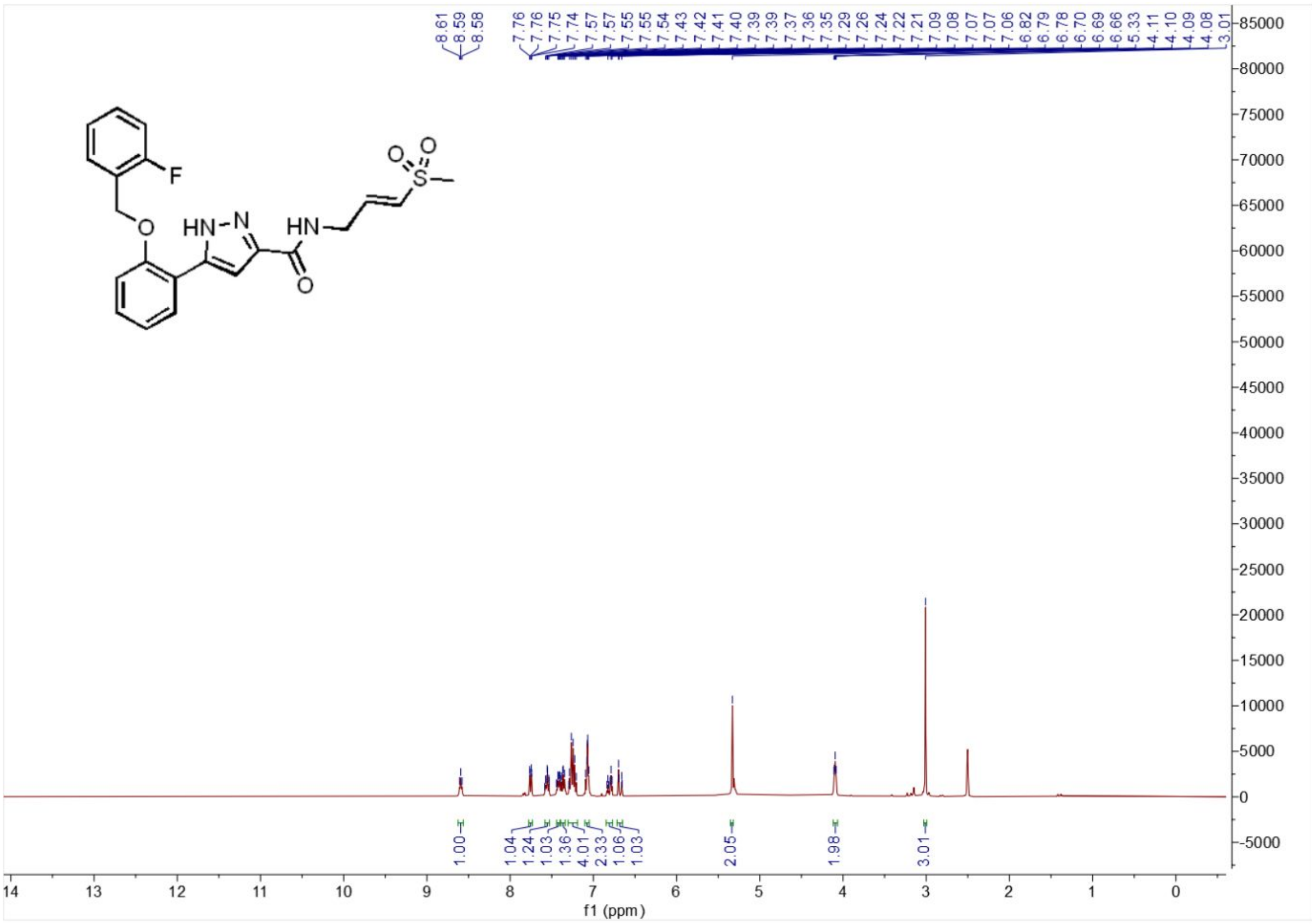

^{1}H NMR spectra of **Jun14482**

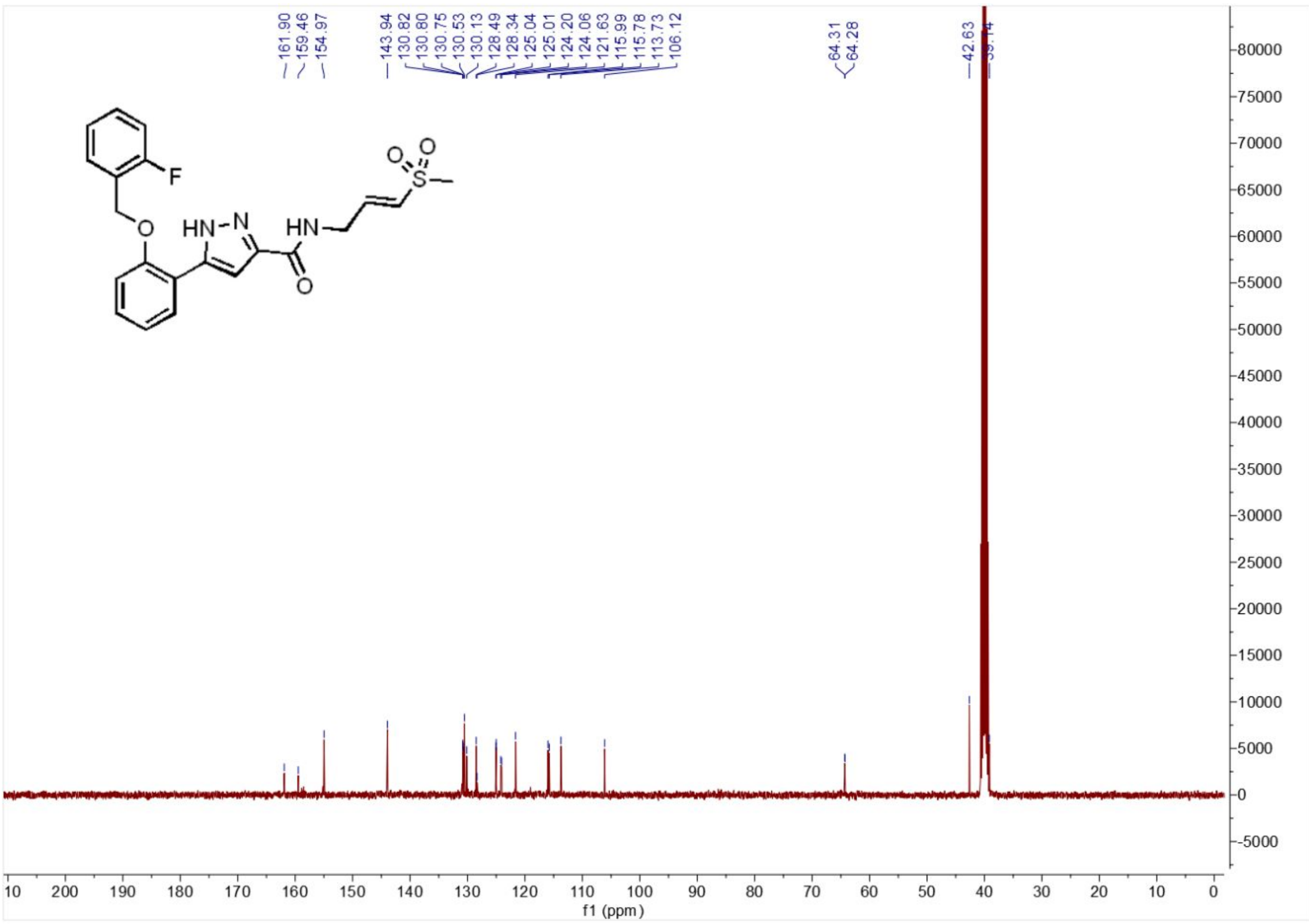

^{13}C NMR spectra of **Jun14482**

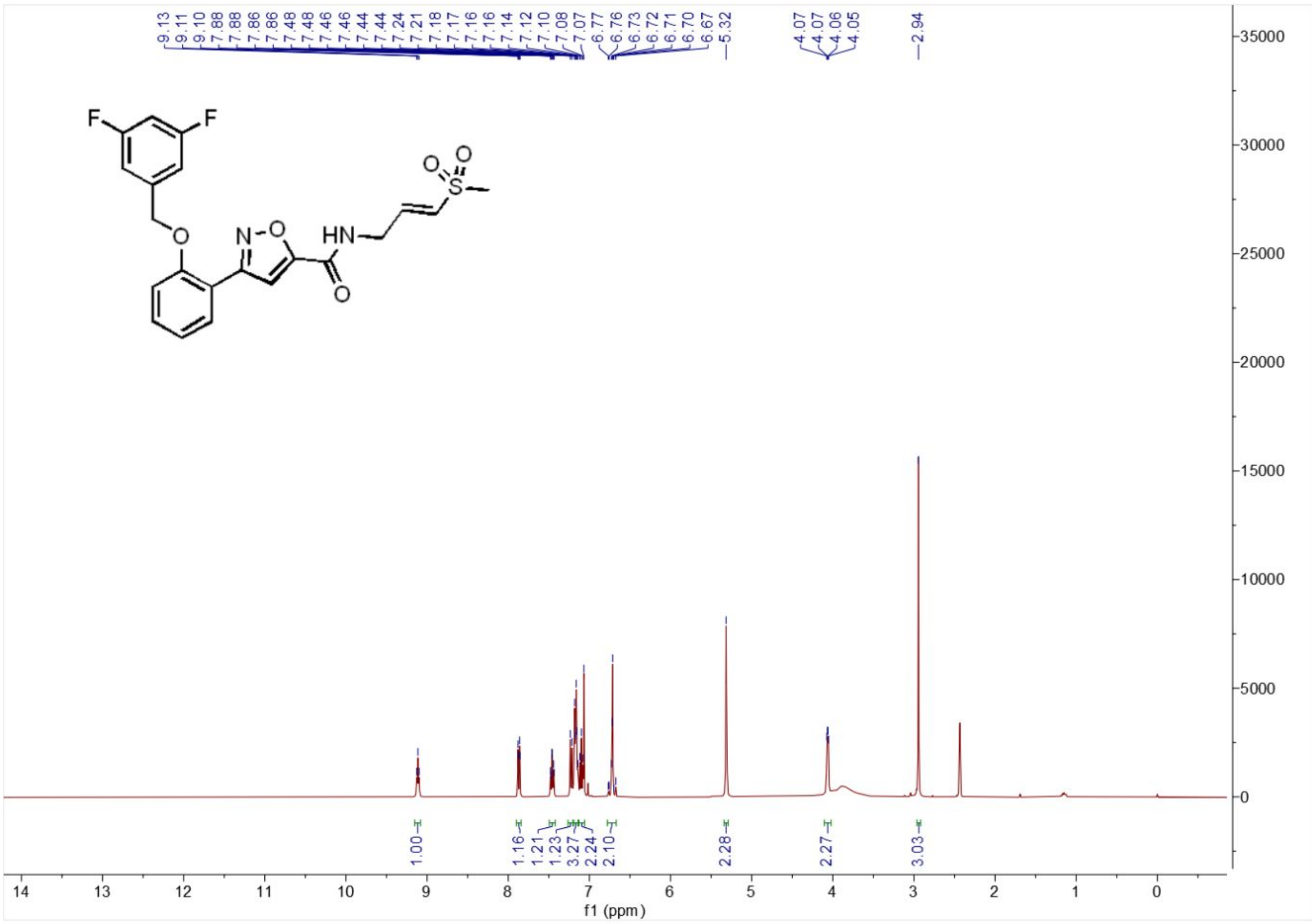

^{1}H NMR spectra of **Jun14363**

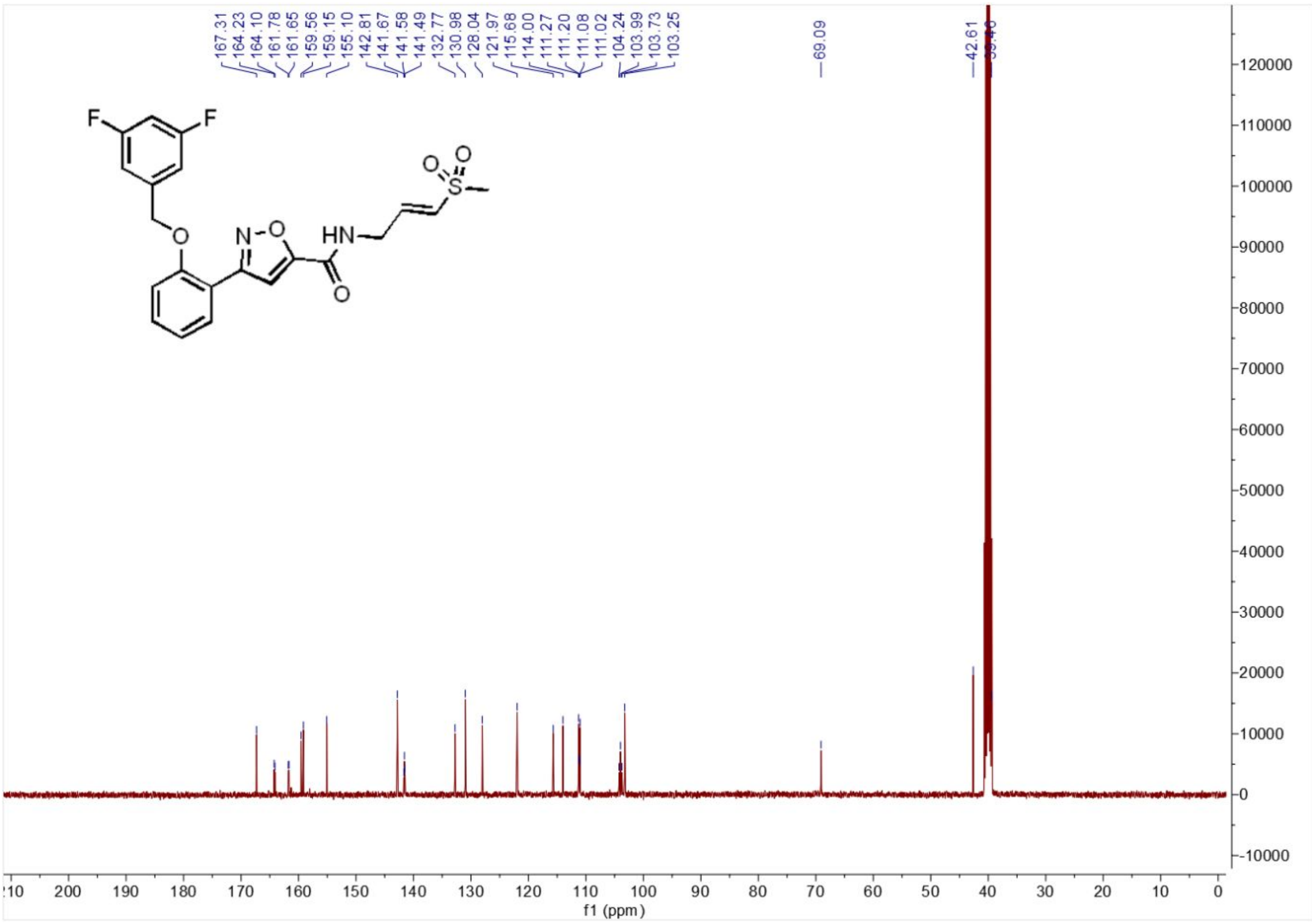

^{13}C NMR spectra of **Jun14363**

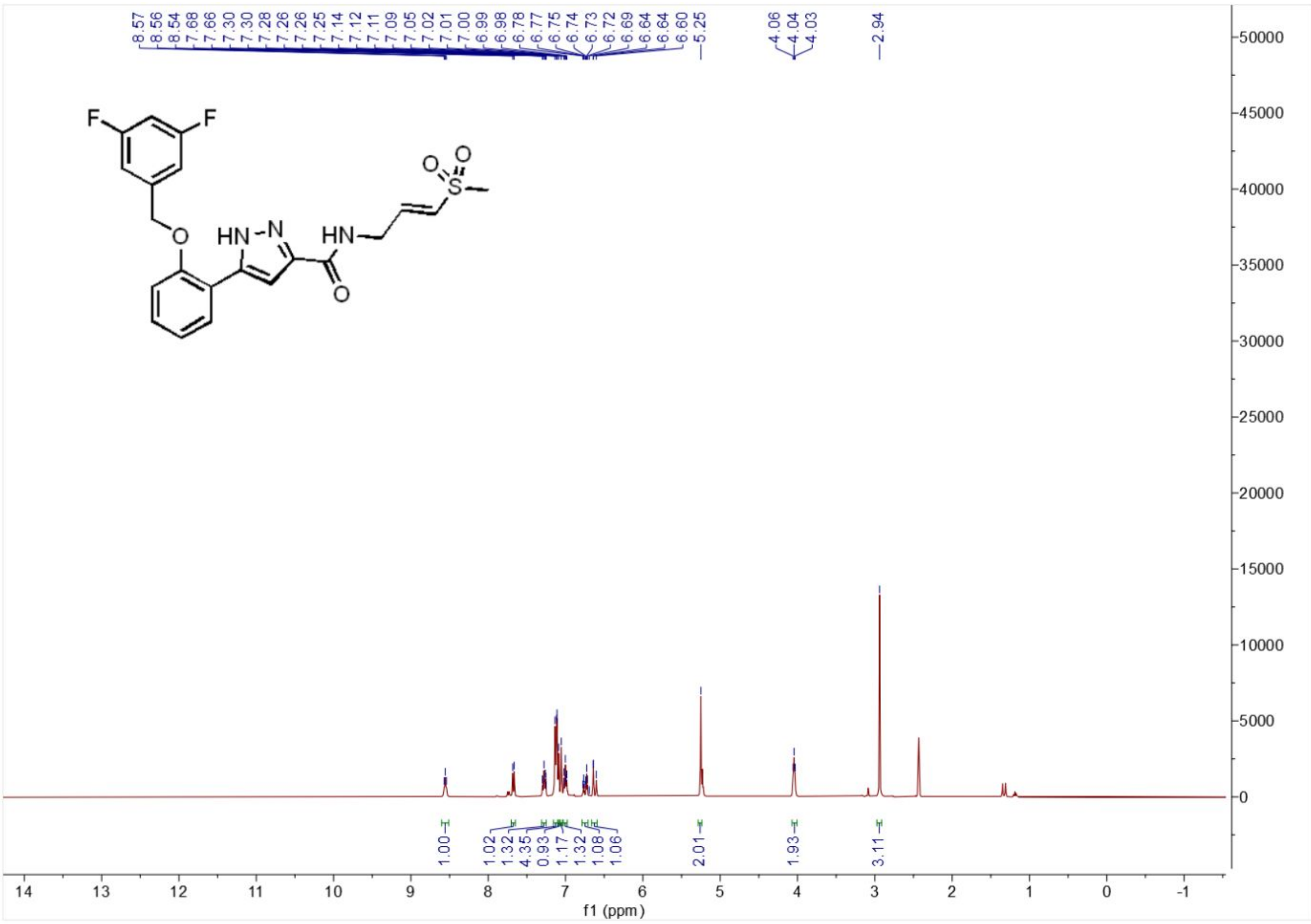

^{1}H NMR spectra of **Jun14364**

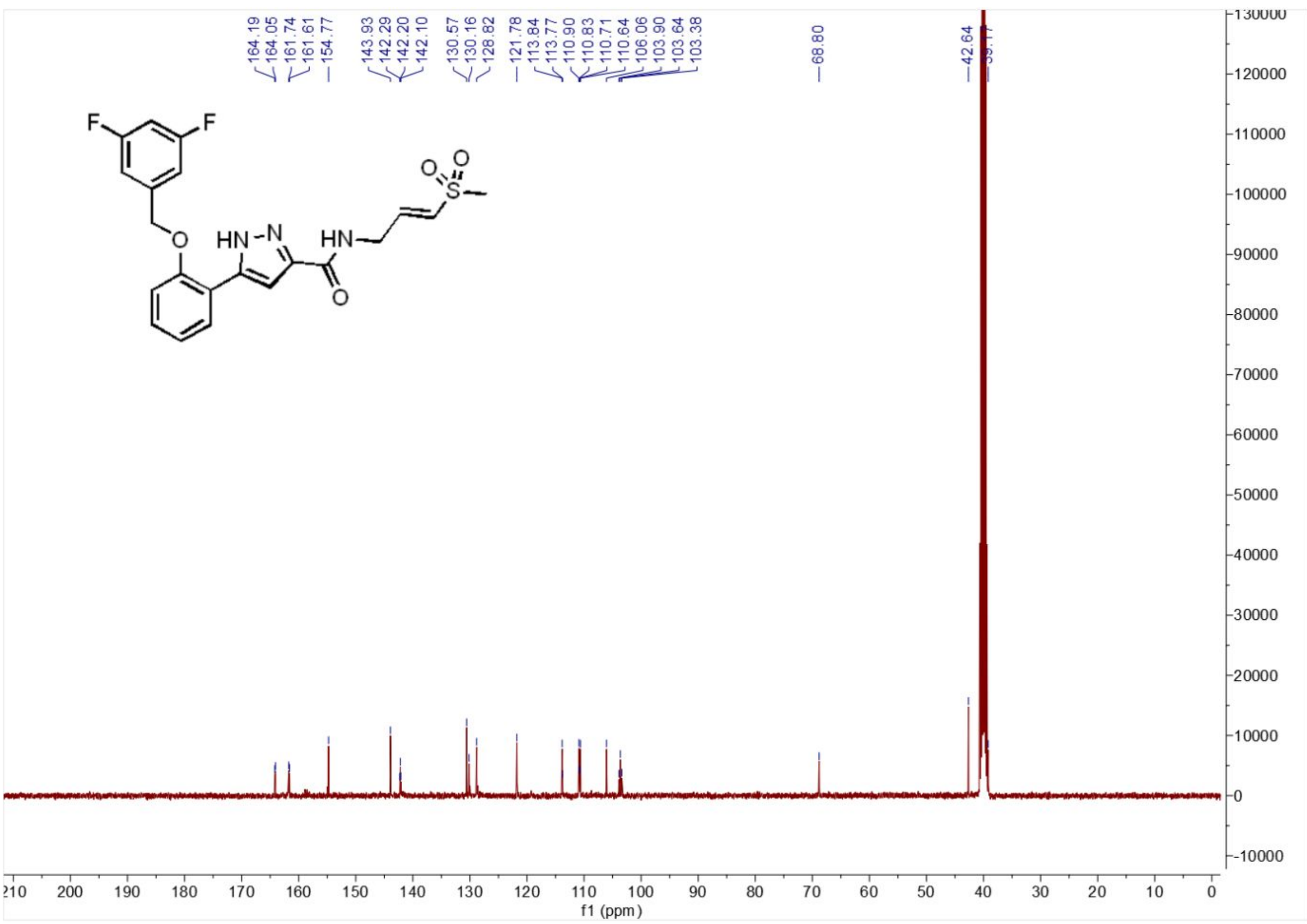

^{13}C NMR spectra of **Jun14364**

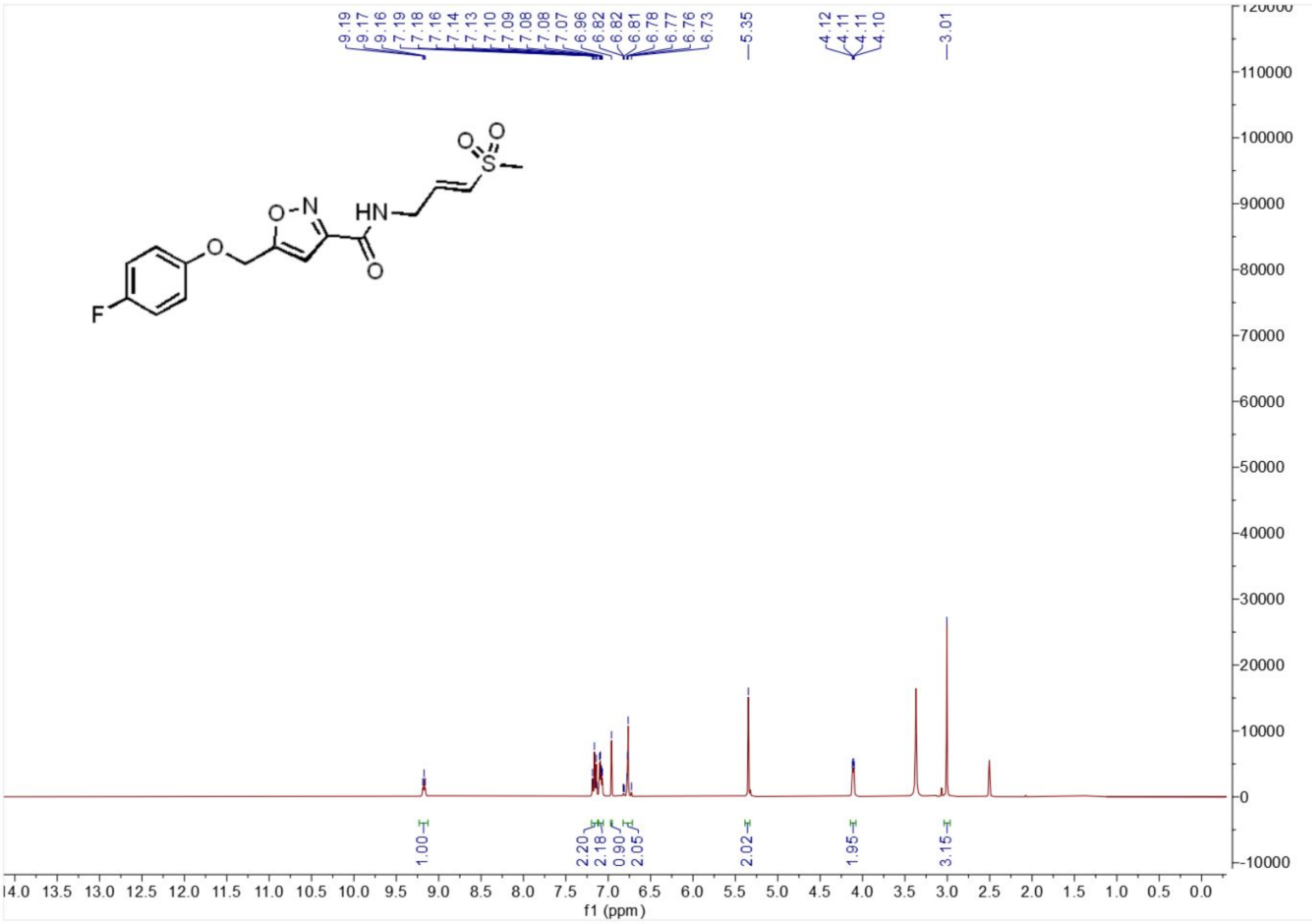

^{1}H NMR spectra of **Jun14458**

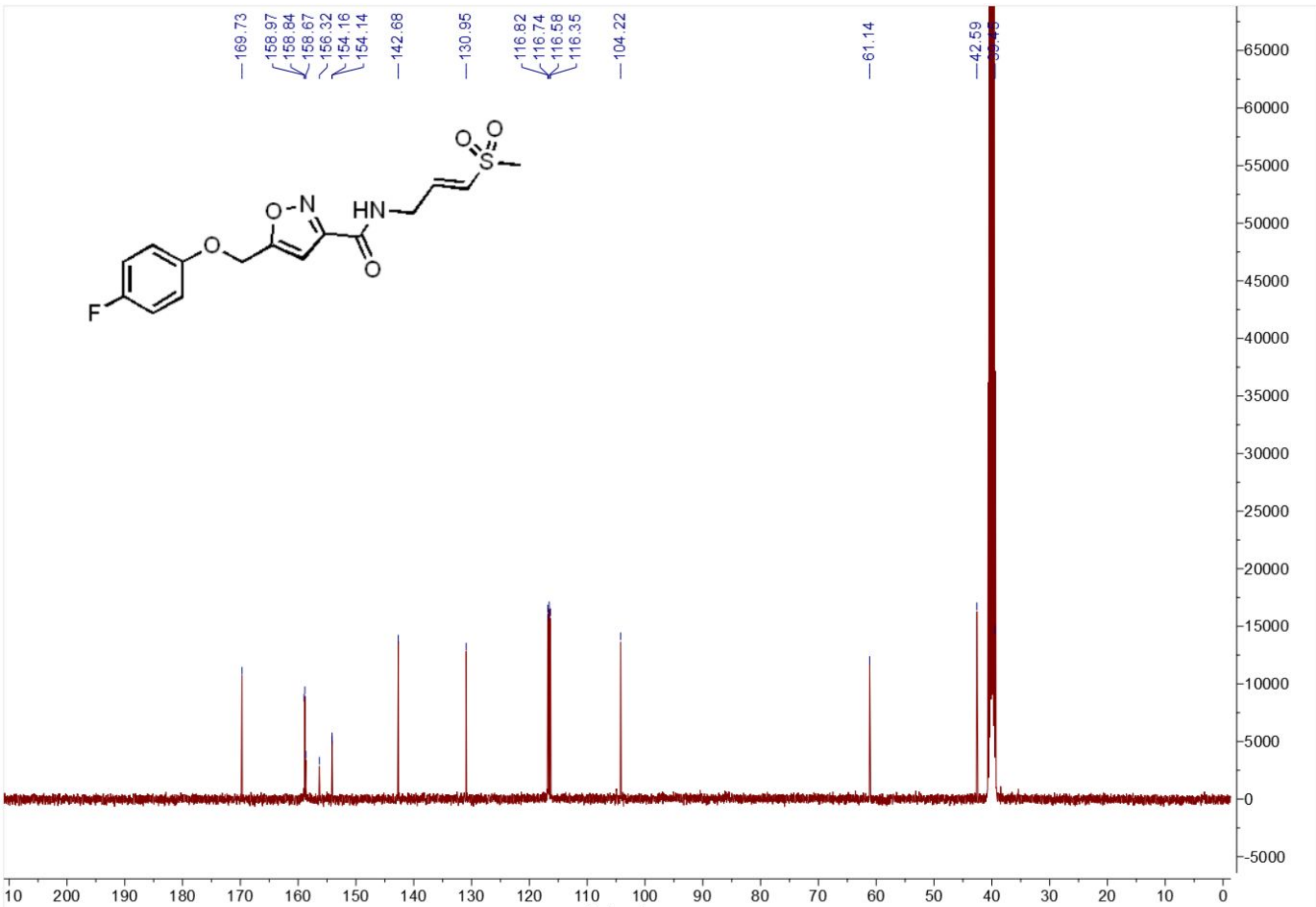

^{13}C NMR spectra of **Jun14458**

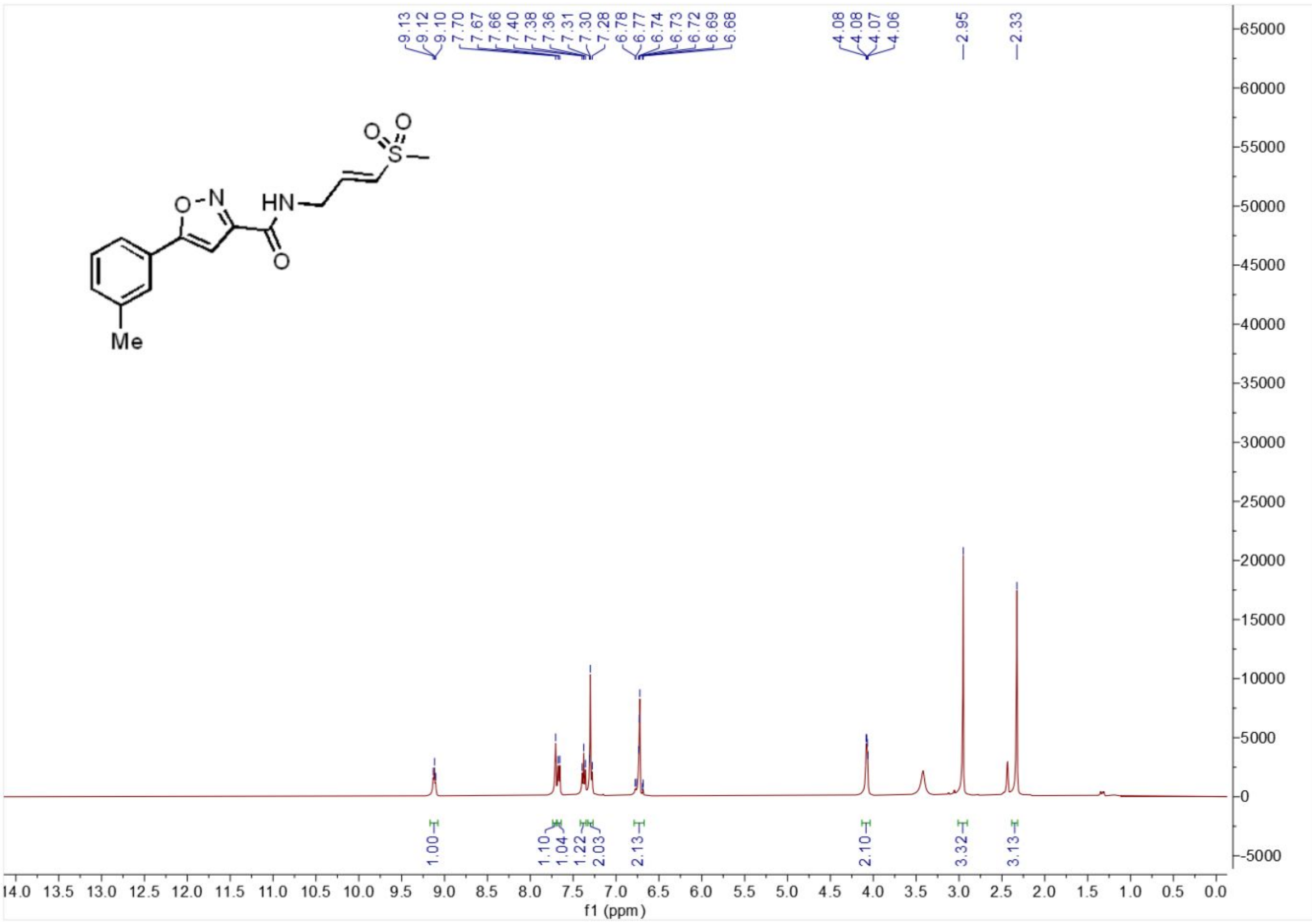

^{1}H NMR spectra of **Jun14502**

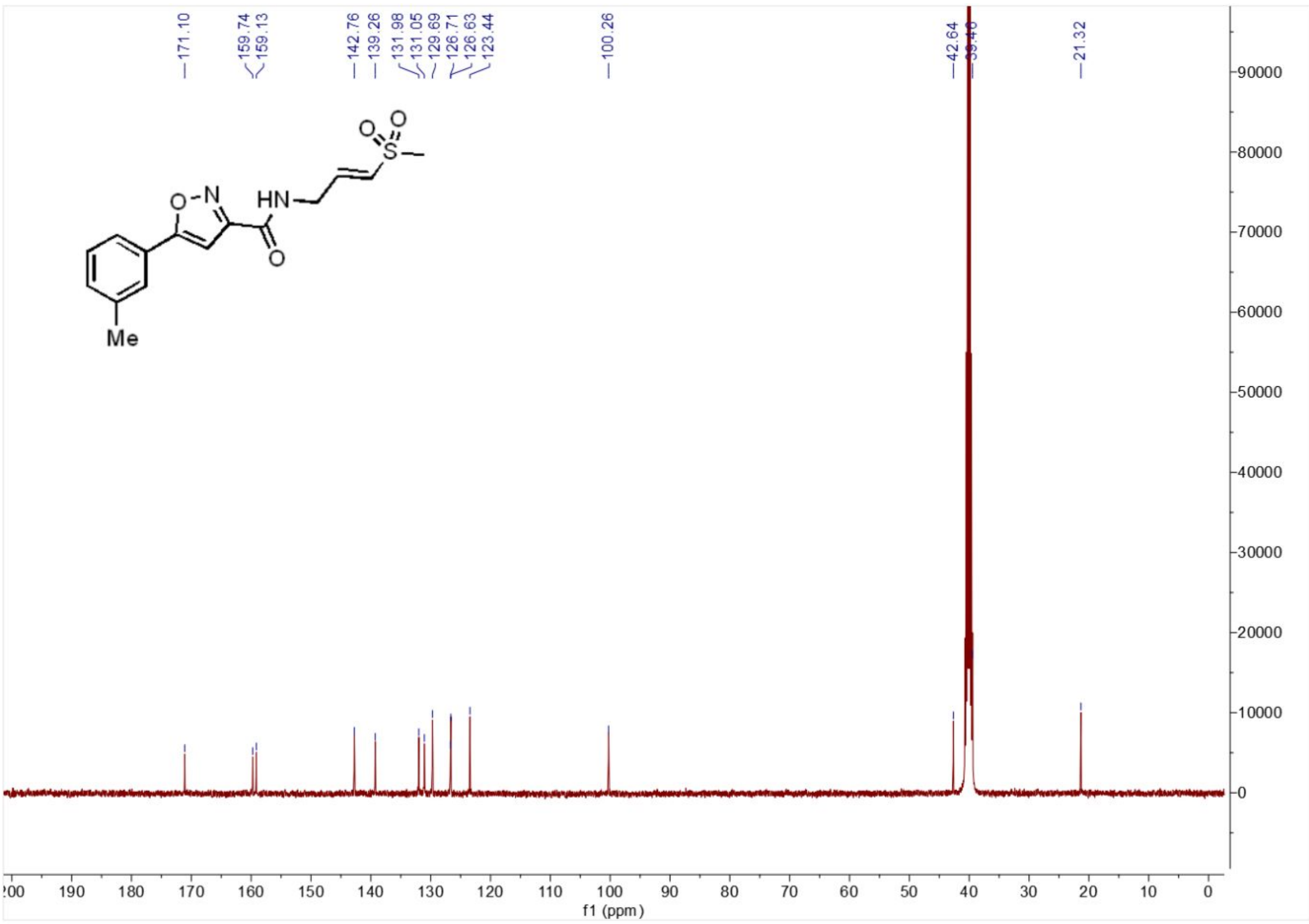

^{13}C NMR spectra of **Jun14502**

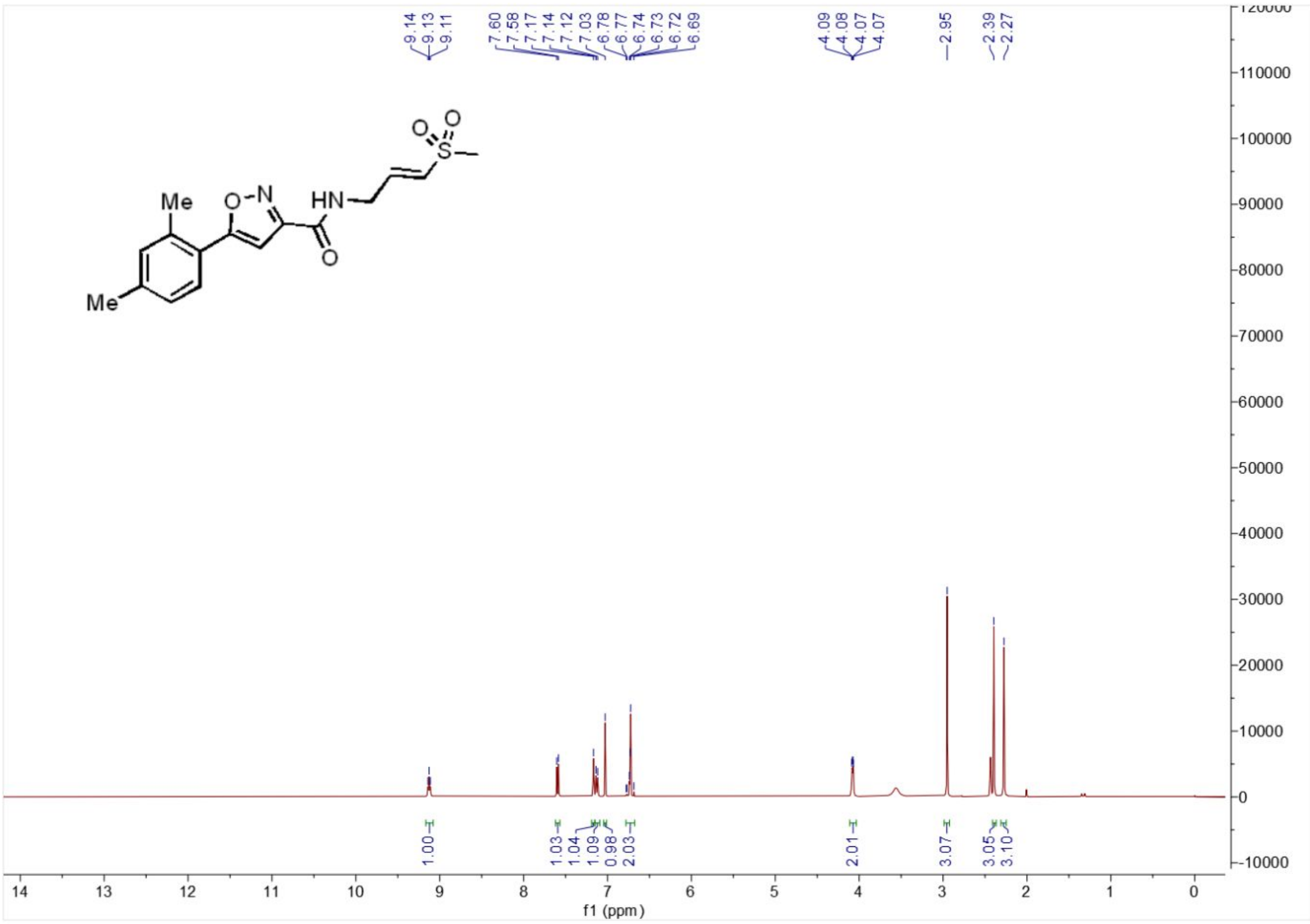

^{1}H NMR spectra of **Jun14528**

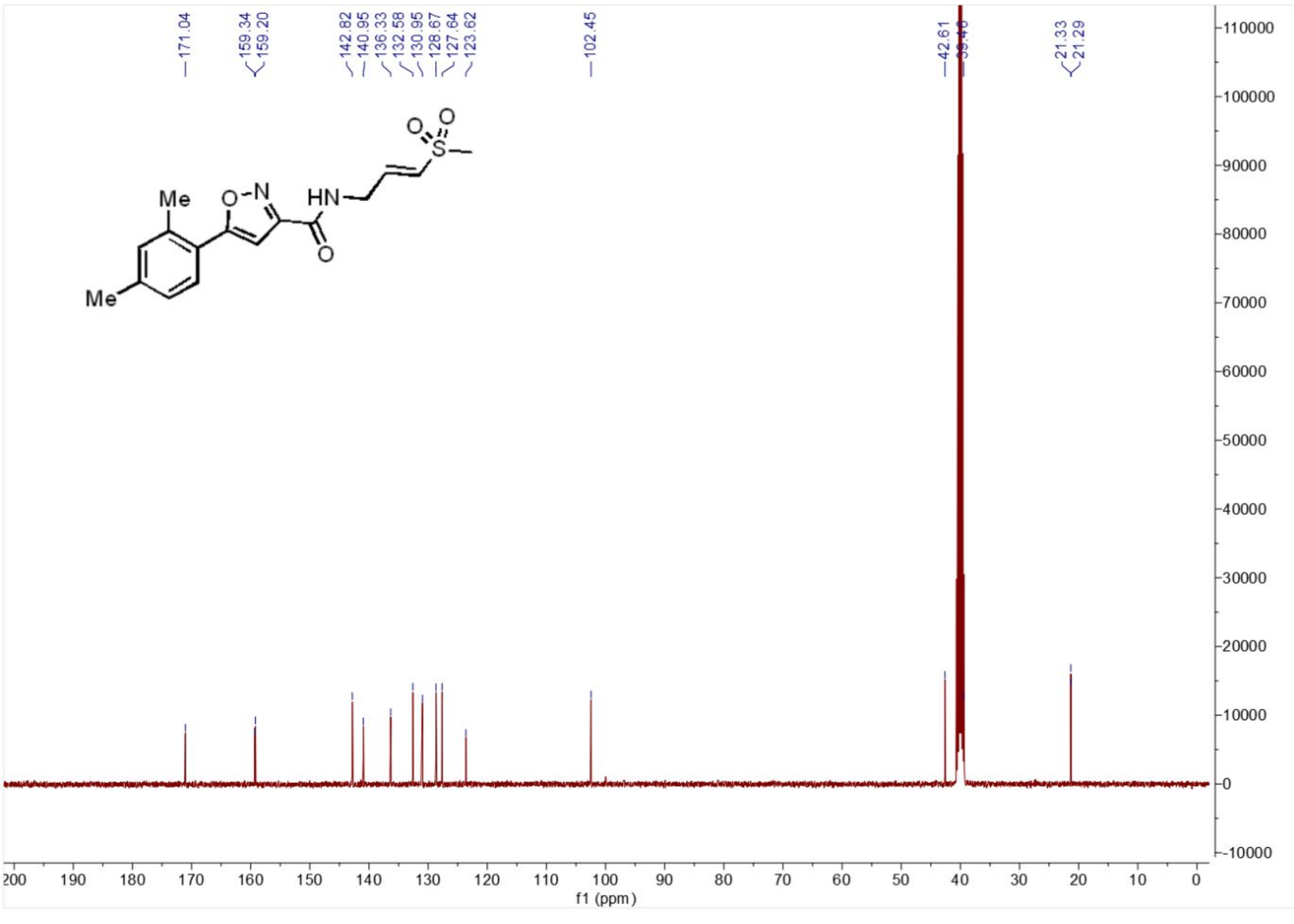

^{13}C NMR spectra of **Jun14528**

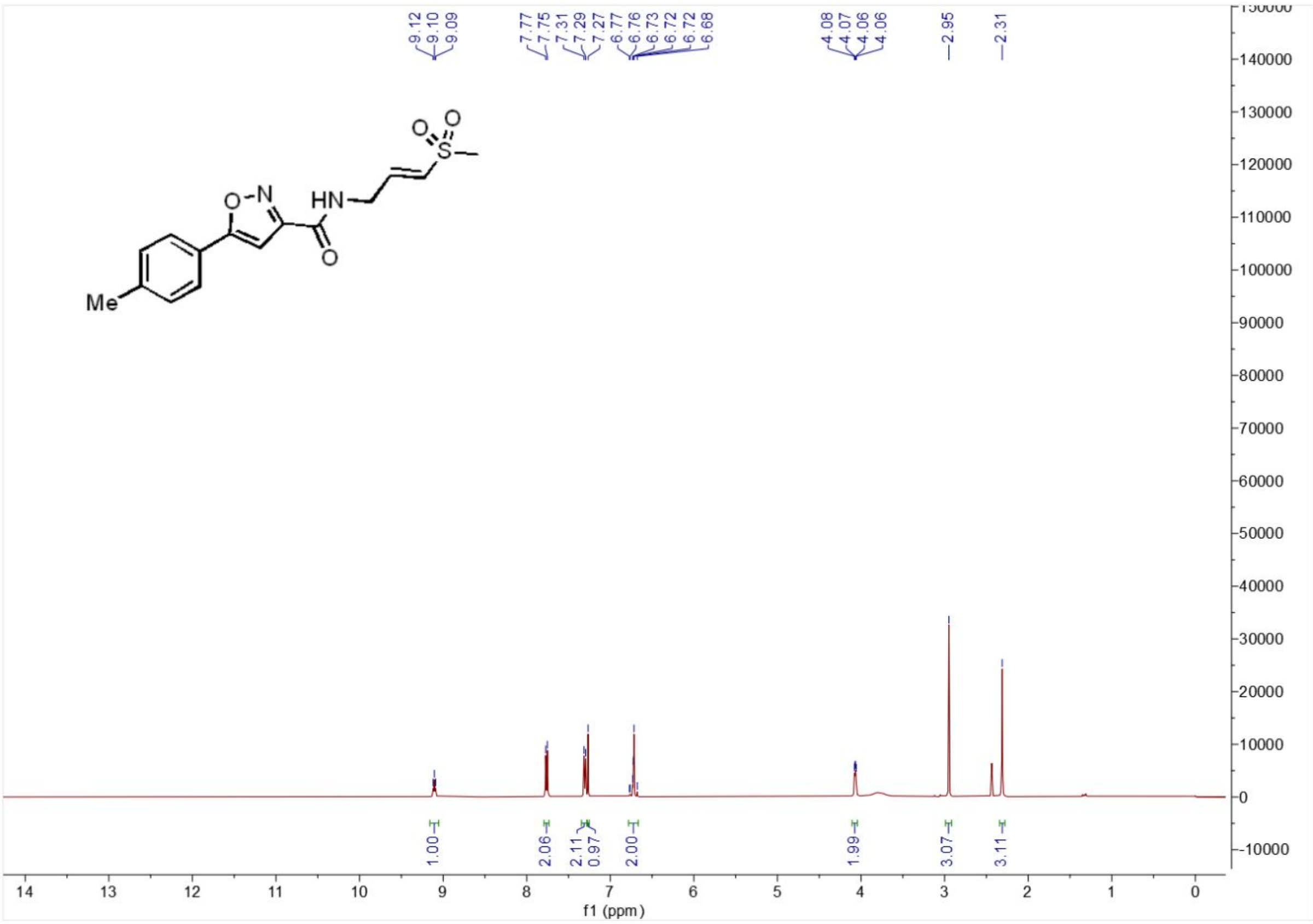

^{1}H NMR spectra of **Jun14529**

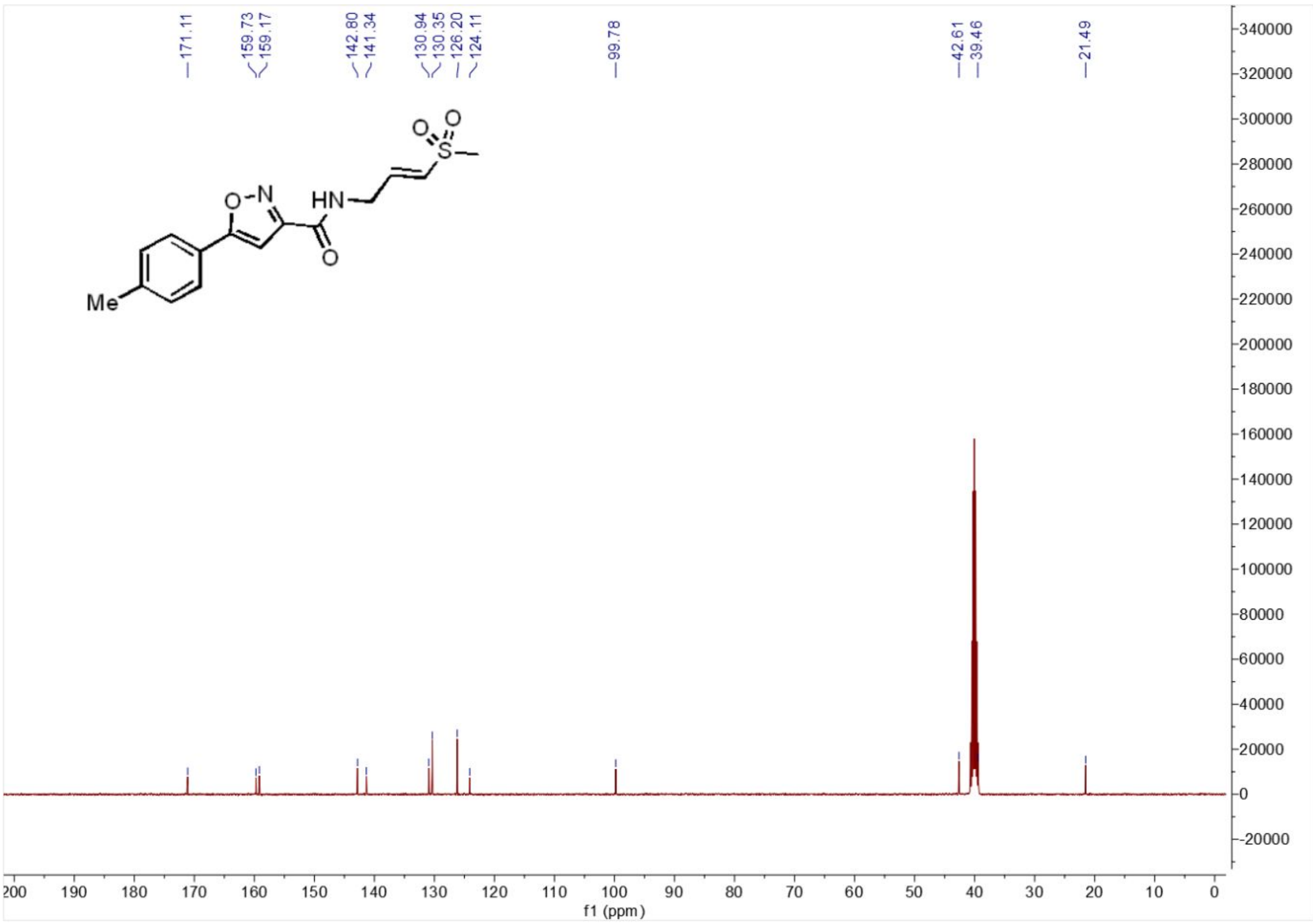

^{13}C NMR spectra of **Jun14529**

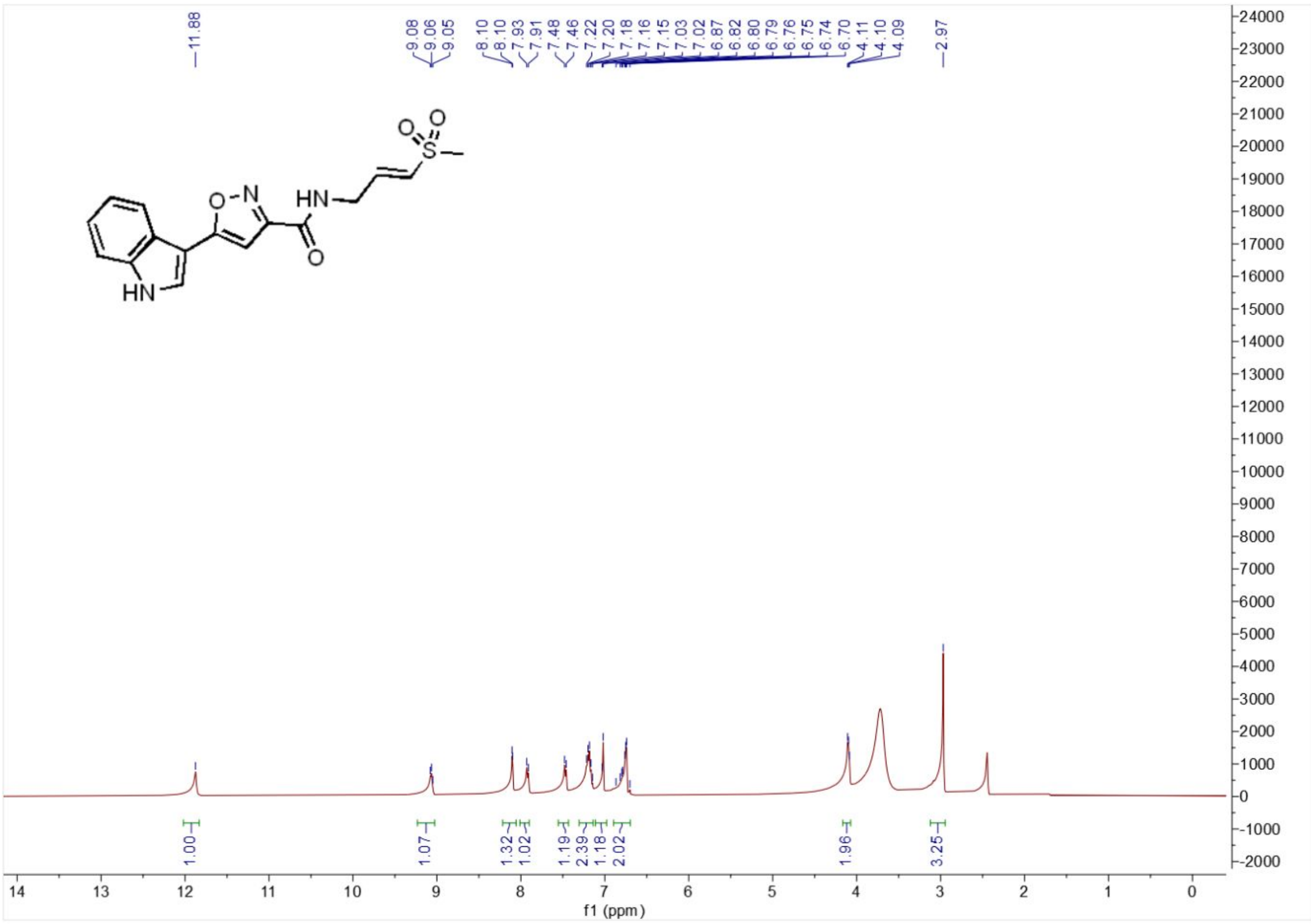

^{1}H NMR spectra of **Jun14617**

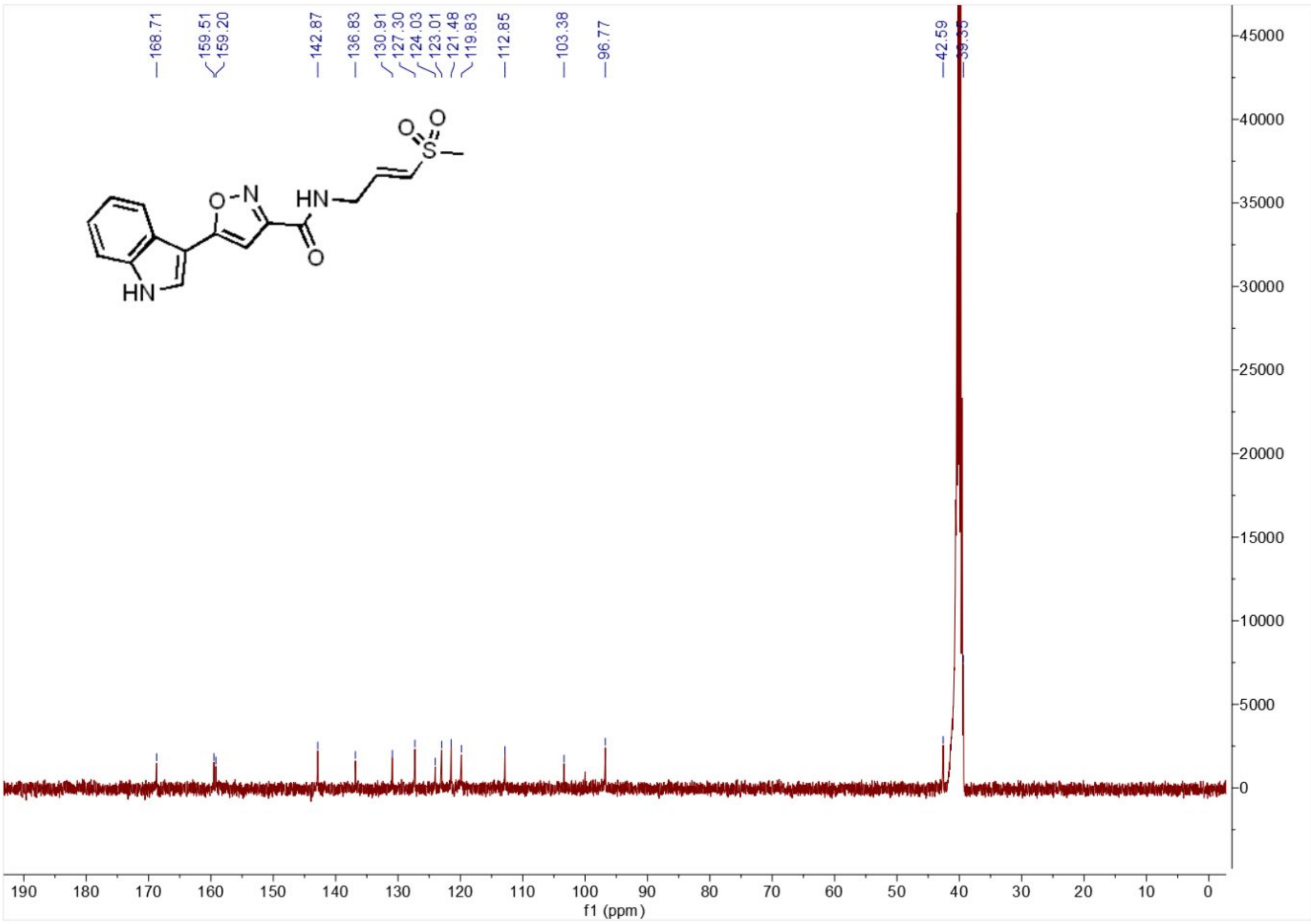

^{13}C NMR spectra of **Jun14617**

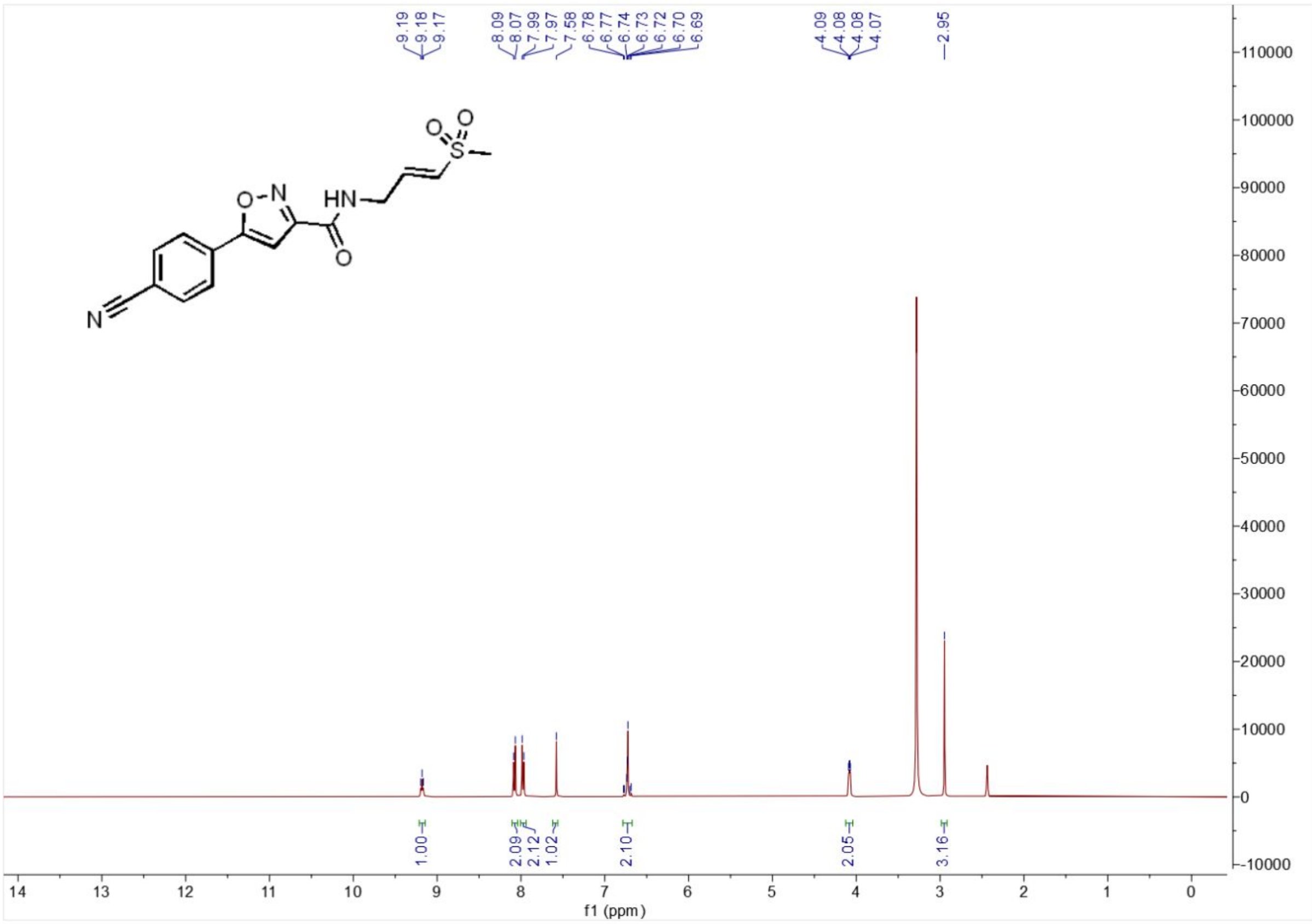

^{1}H NMR spectra of **Jun14693**

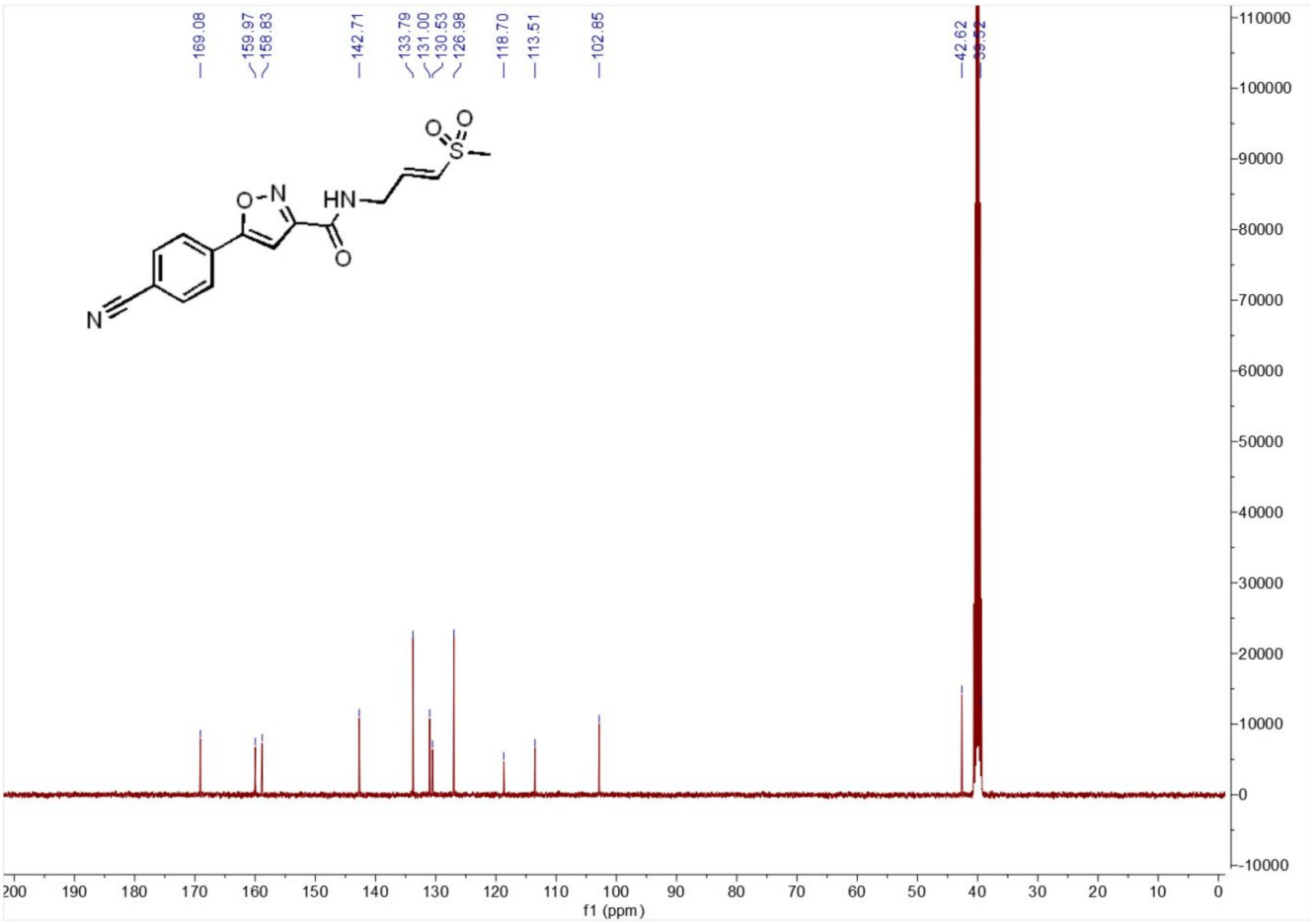

^{13}C NMR spectra of **Jun14693**

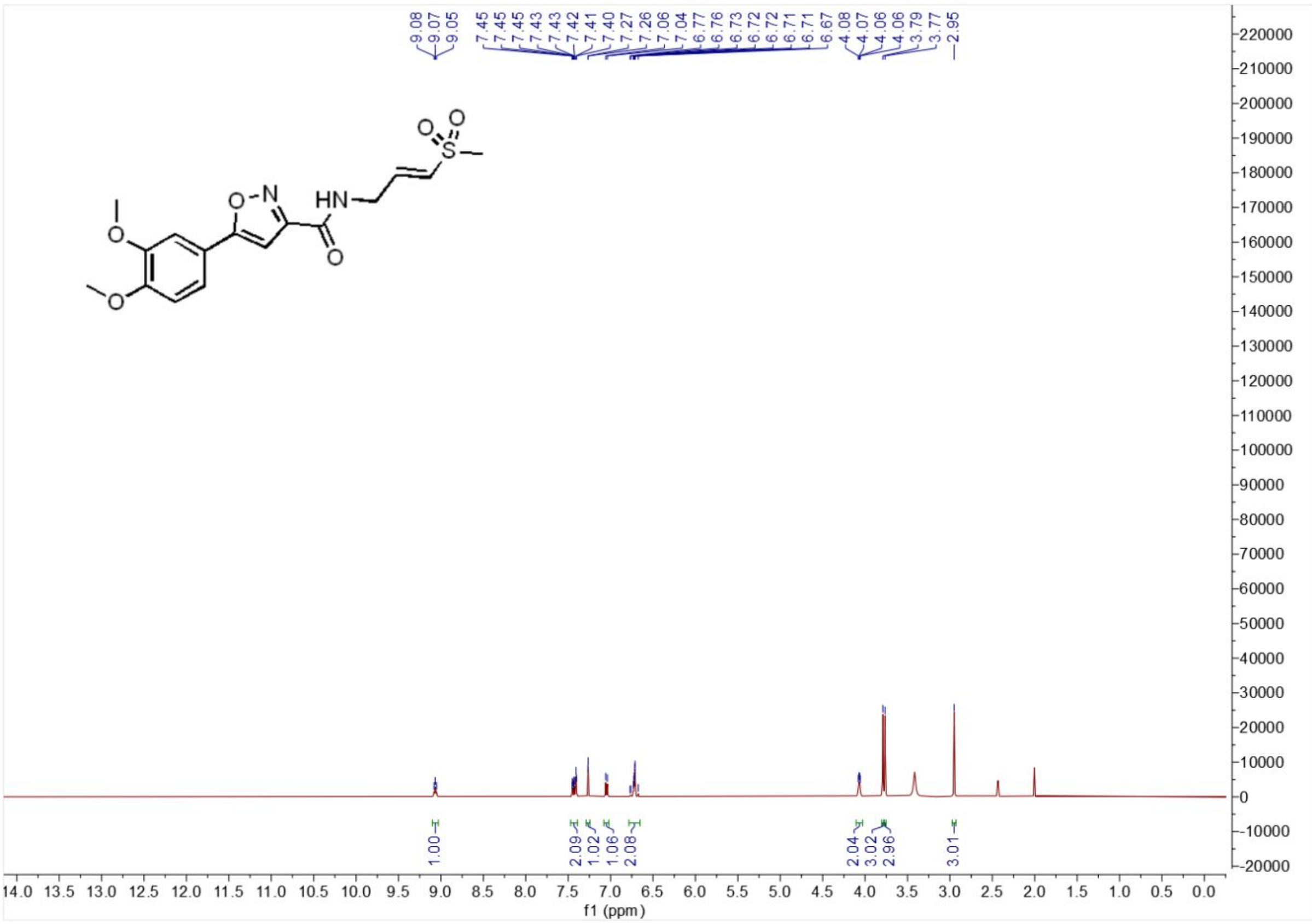

^{1}H NMR spectra of **Jun14694**

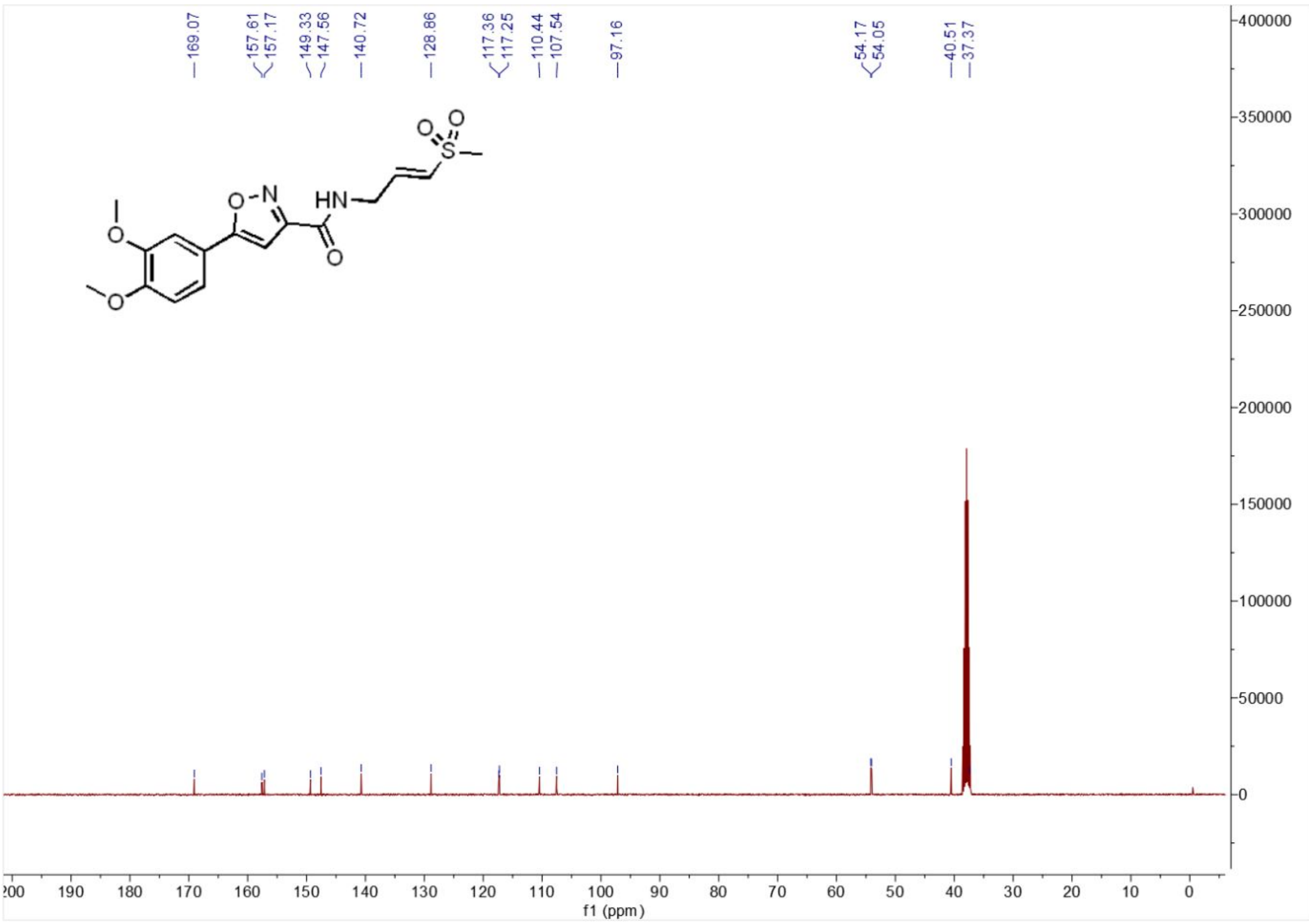

^{13}C NMR spectra of **Jun14694**

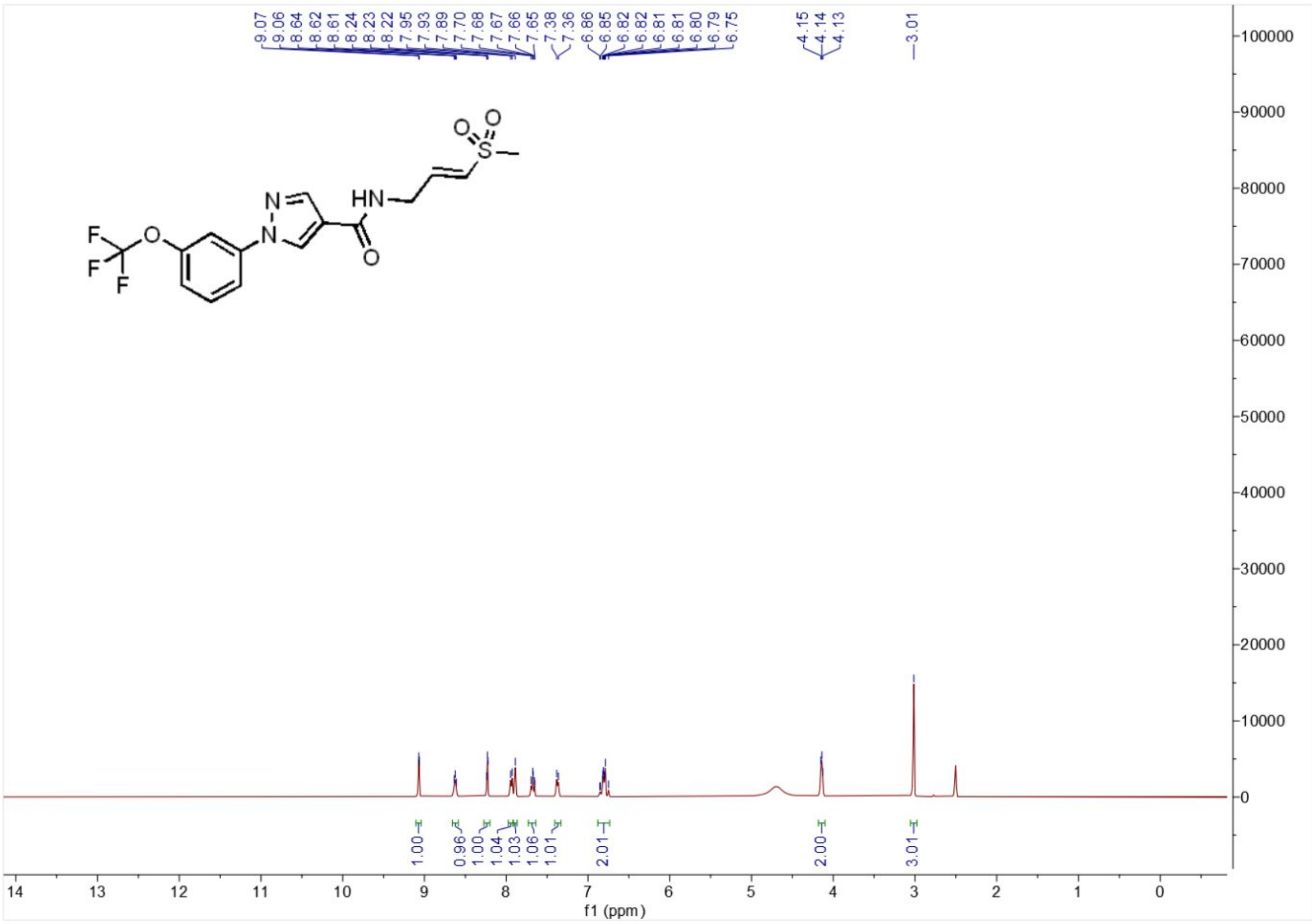

^{1}H NMR spectra of **Jun14736**

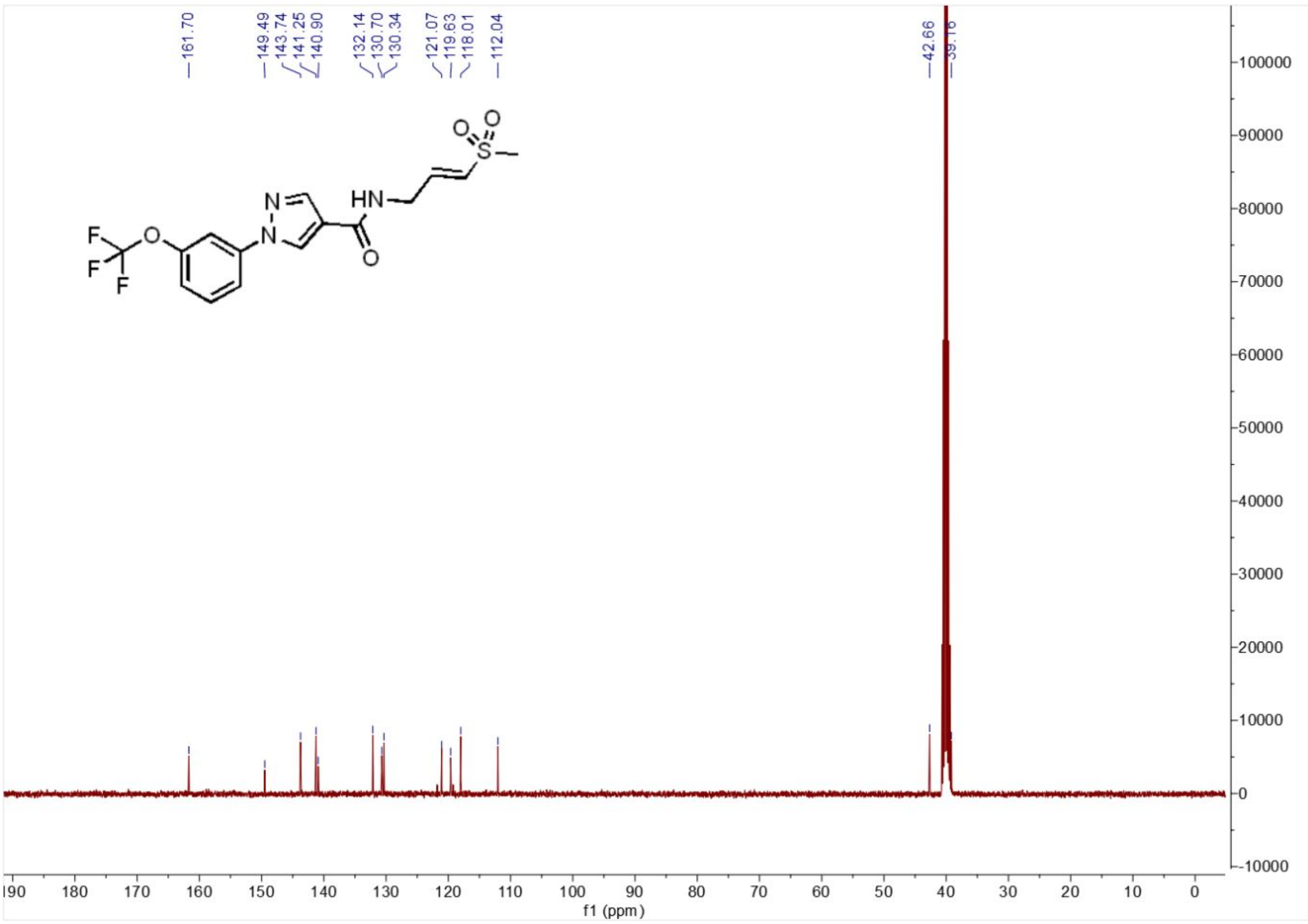

^{13}C NMR spectra of **Jun14736**

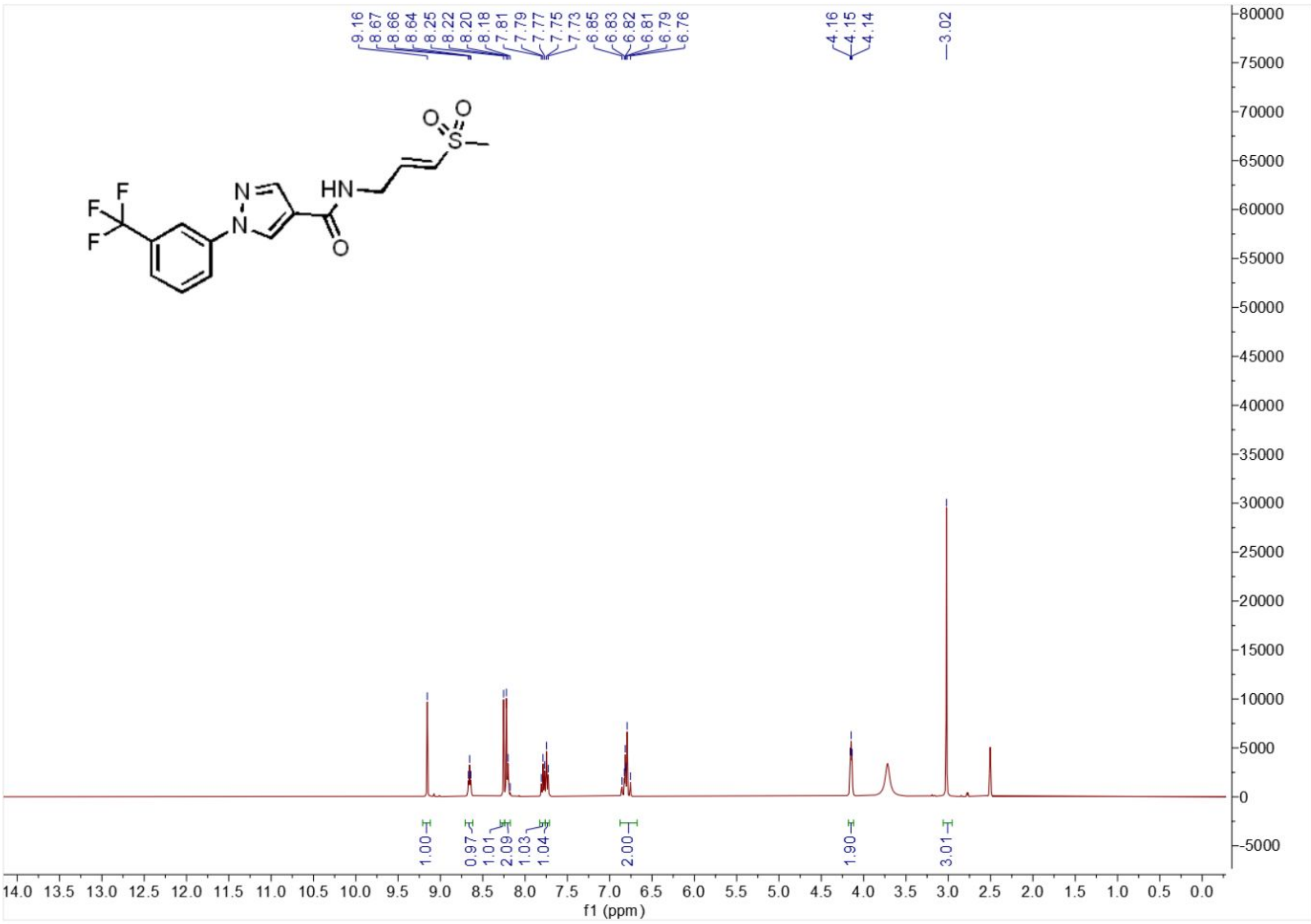

^{1}H NMR spectra of **Jun14741**

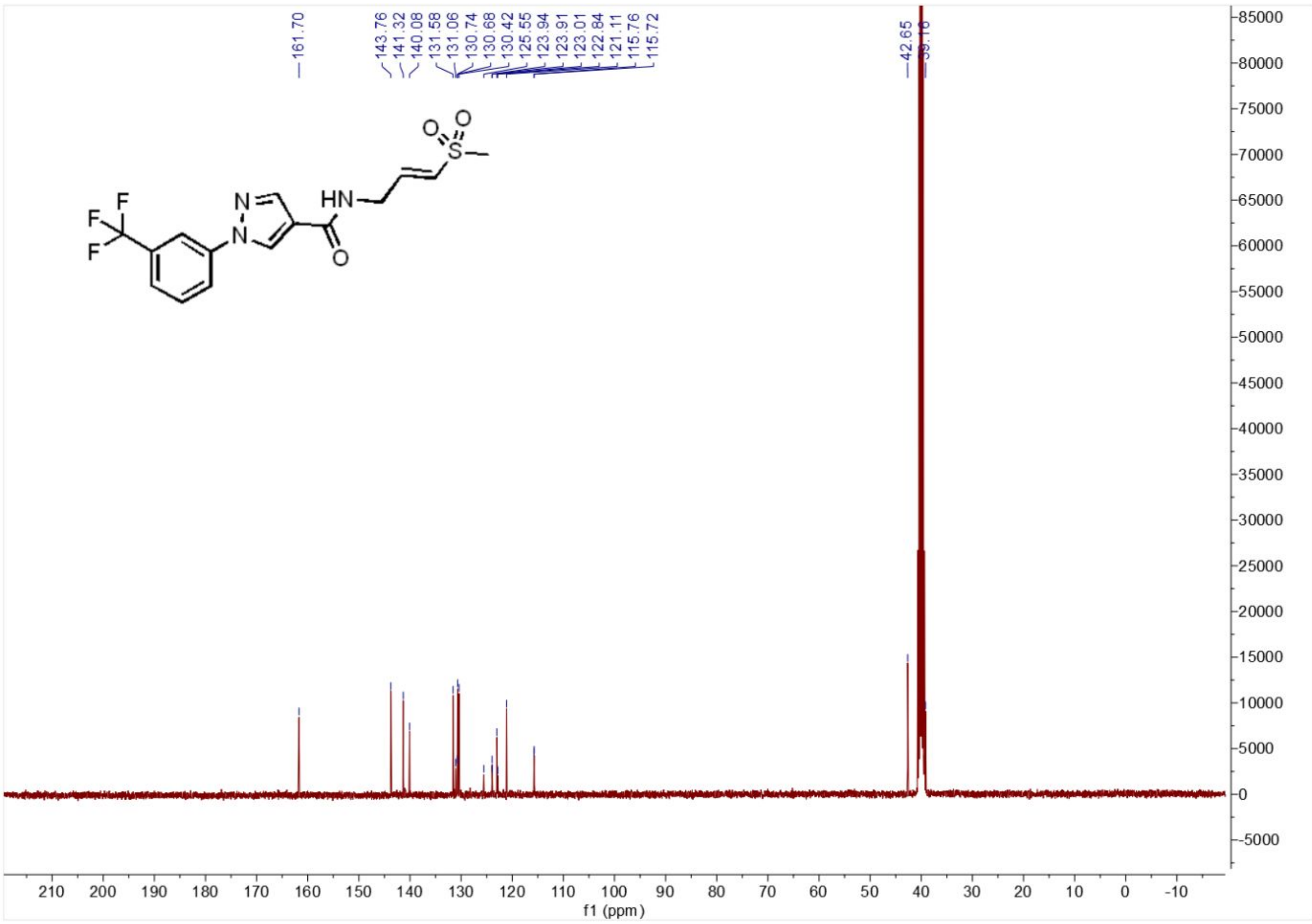

^{13}C NMR spectra of **Jun14741**

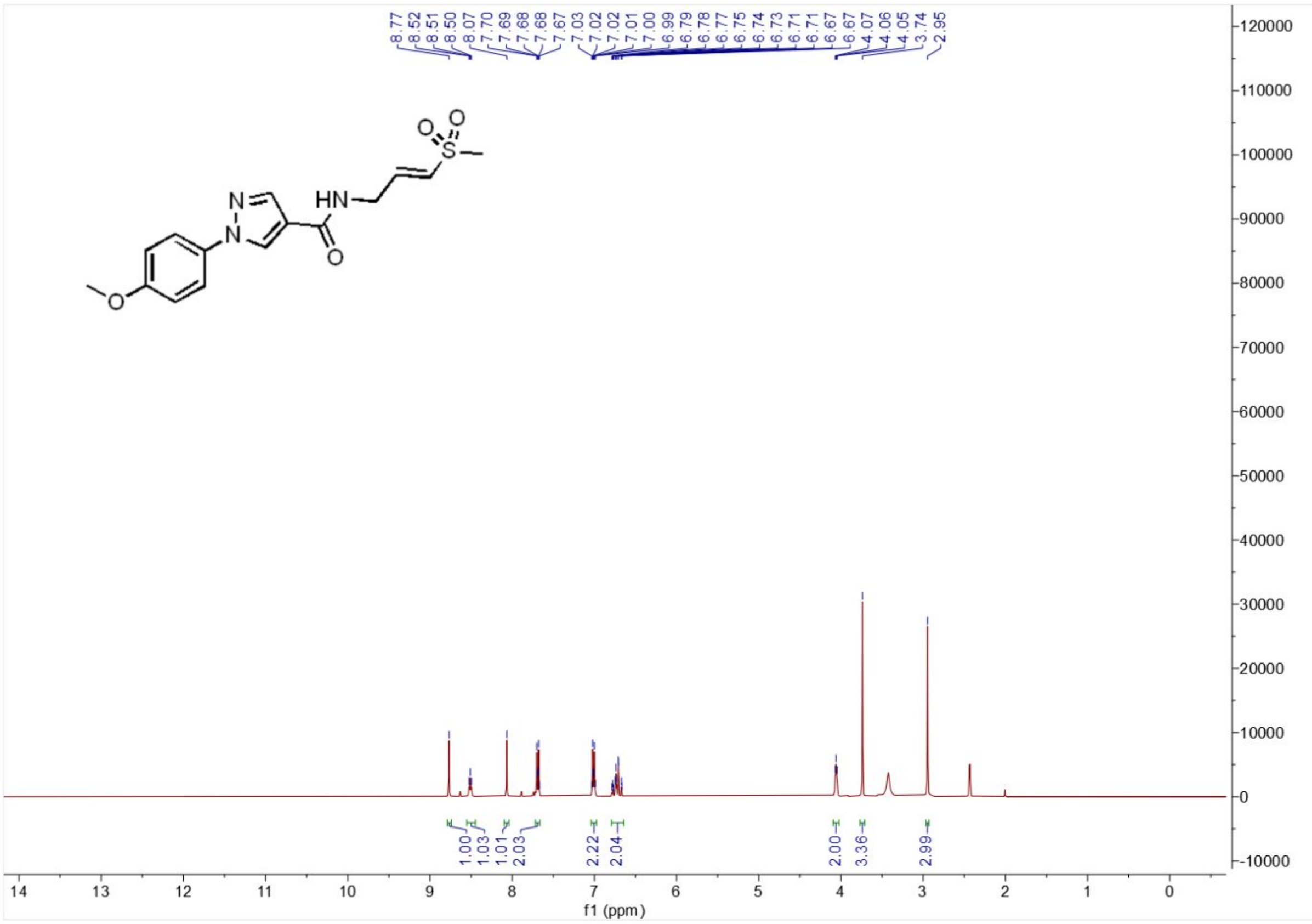

^{1}H NMR spectra of **Jun14742**

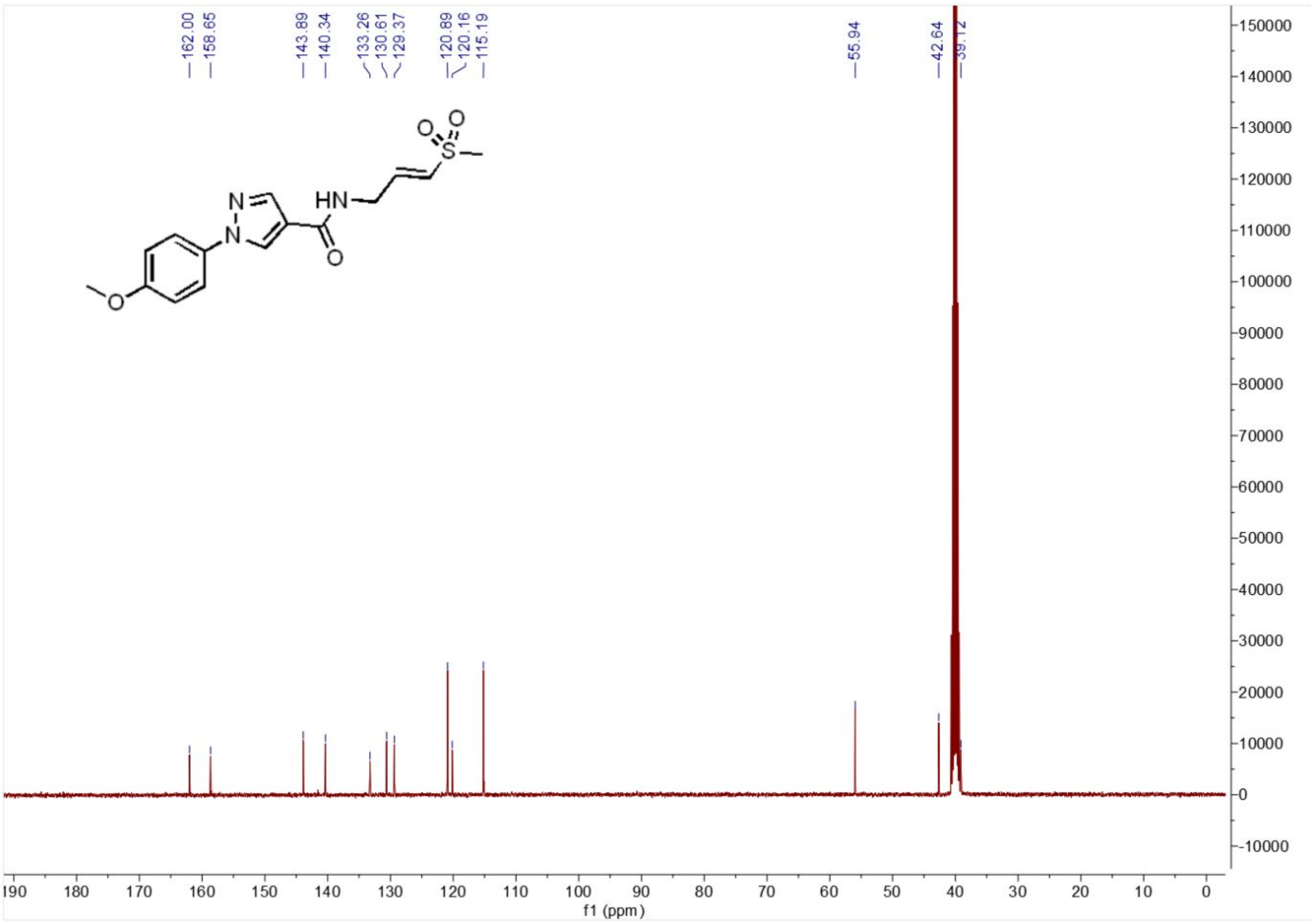

^{13}C NMR spectra of **Jun14742**

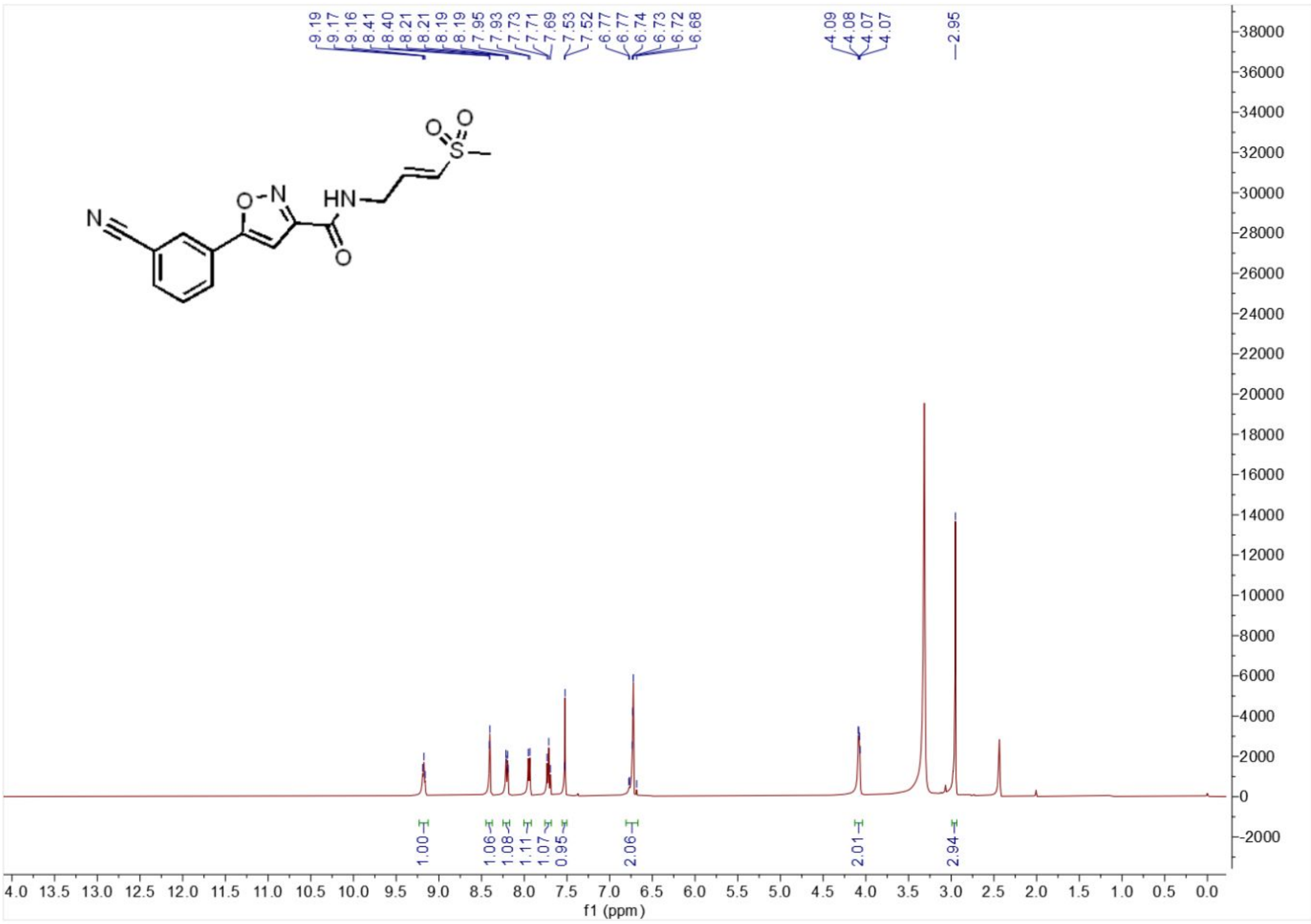

^{1}H NMR spectra of **Jun14744**

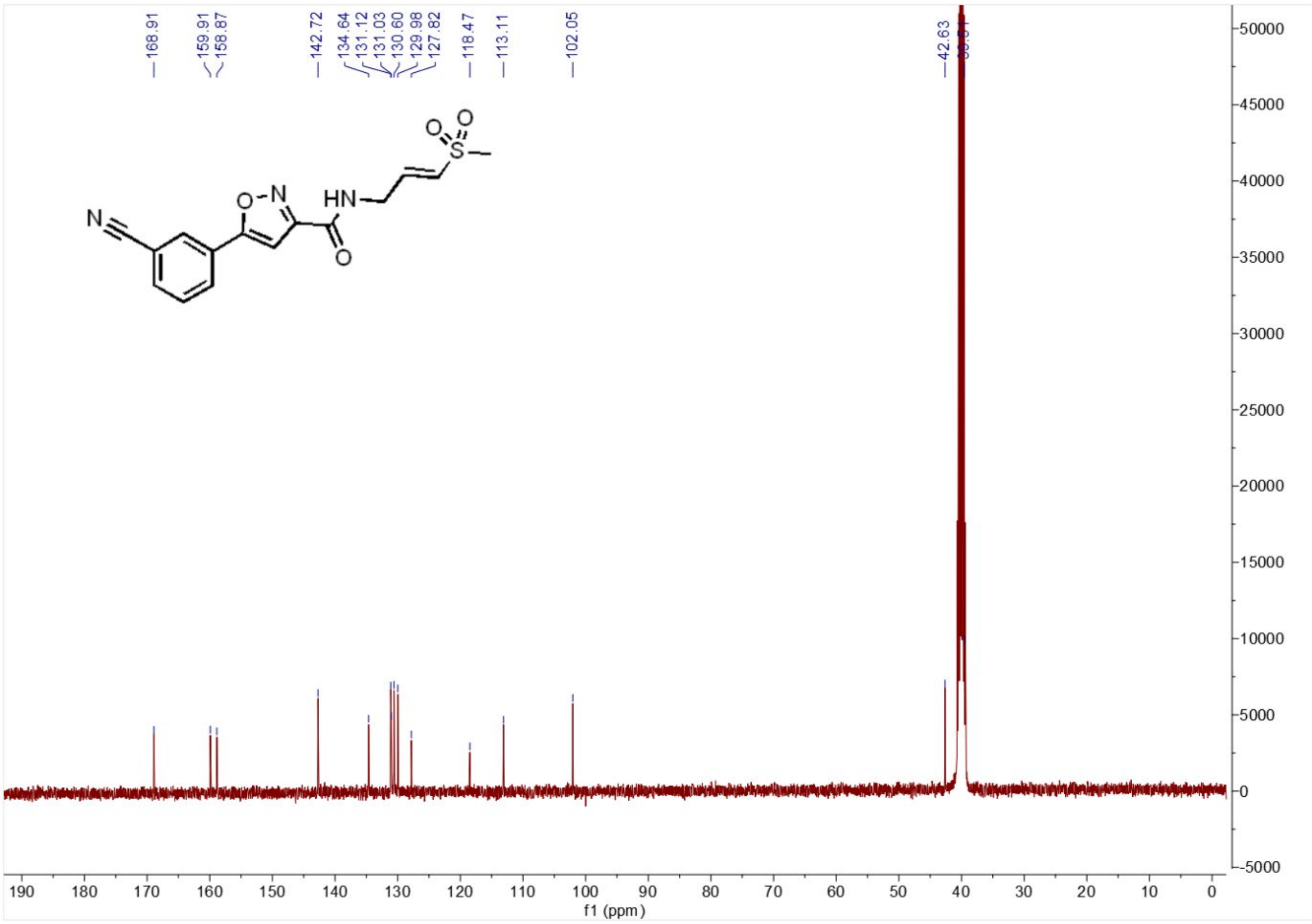

^{13}C NMR spectra of **Jun14744**

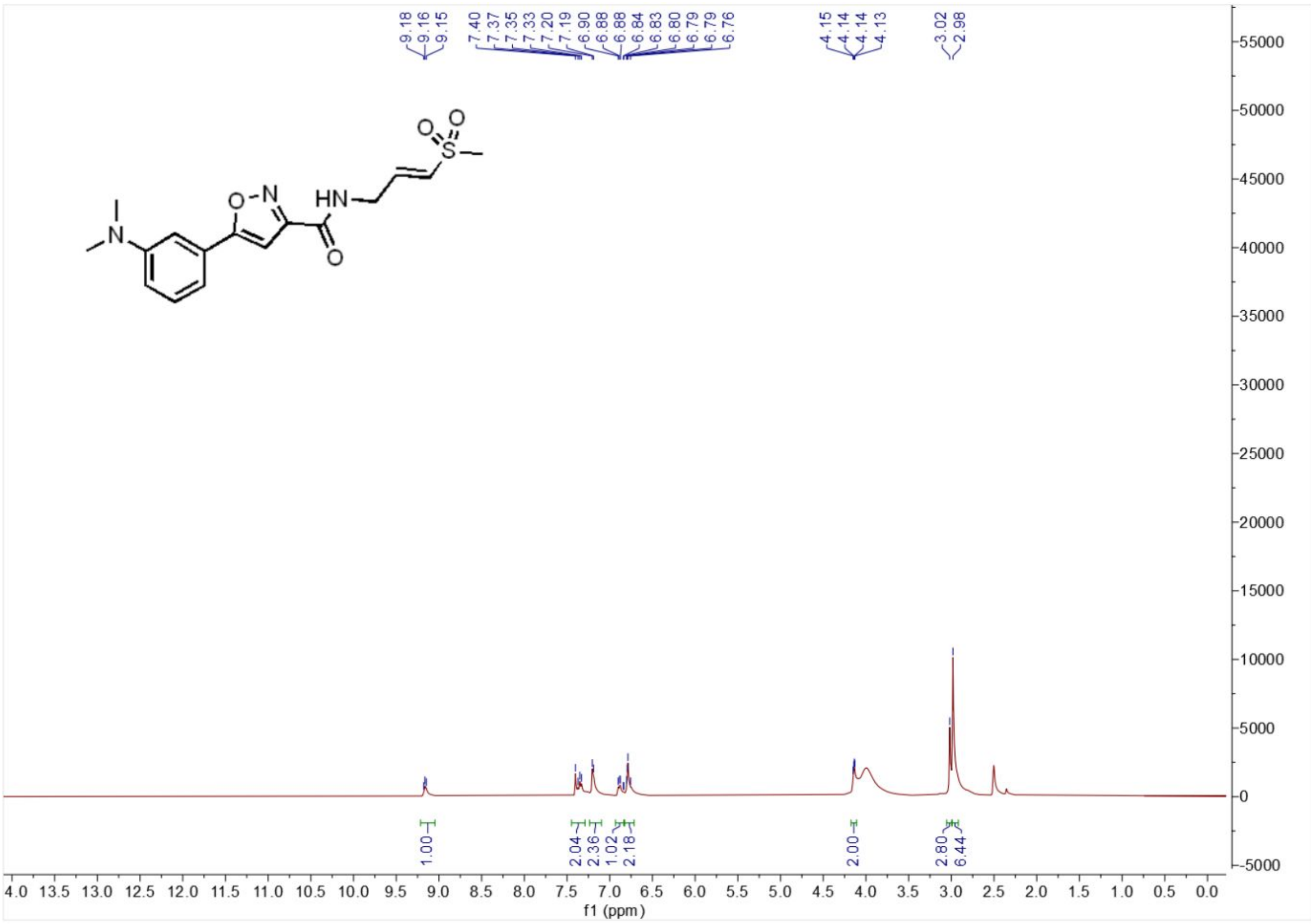

^{1}H NMR spectra of **Jun14778**

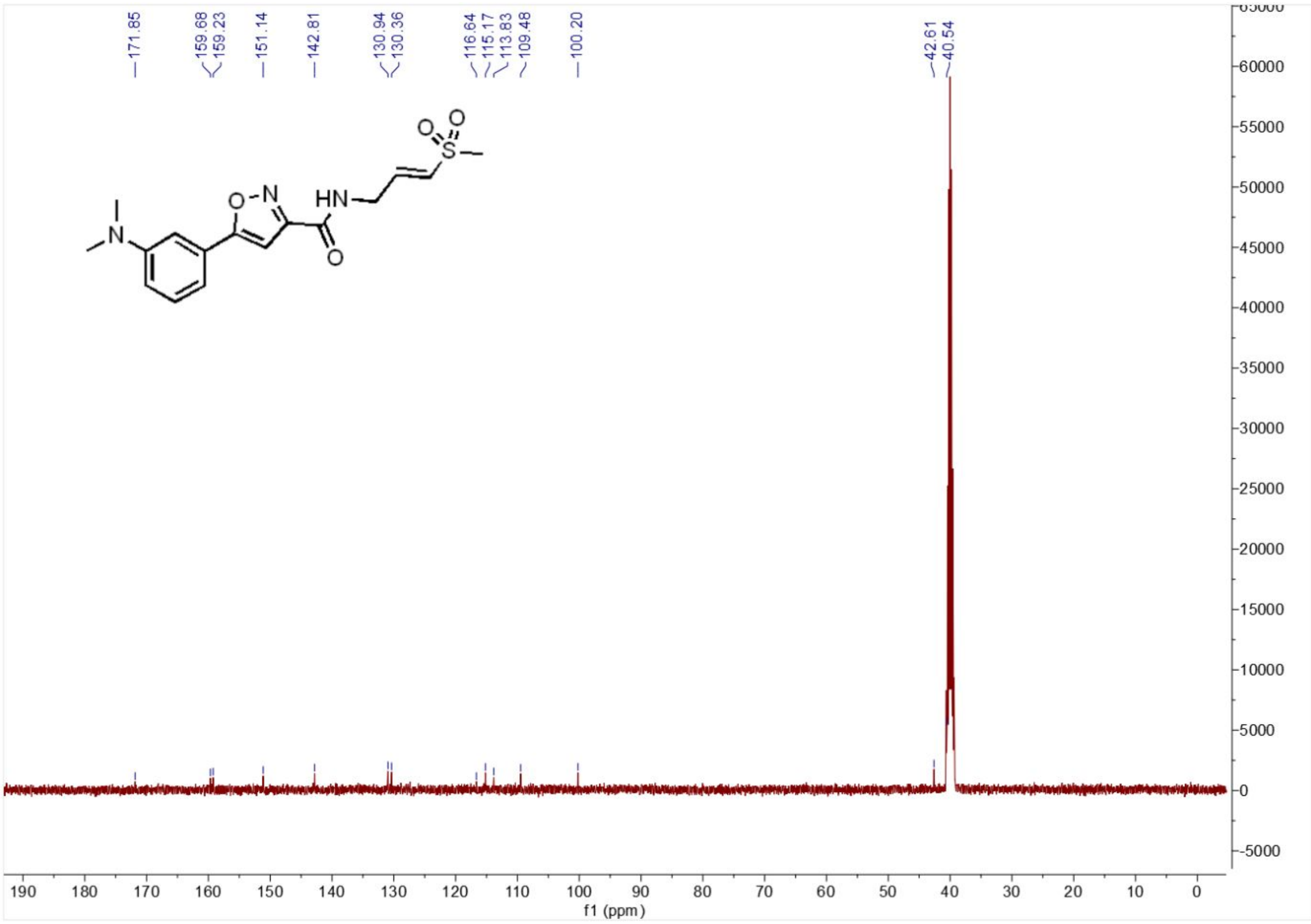

^{13}C NMR spectra of **Jun14778**

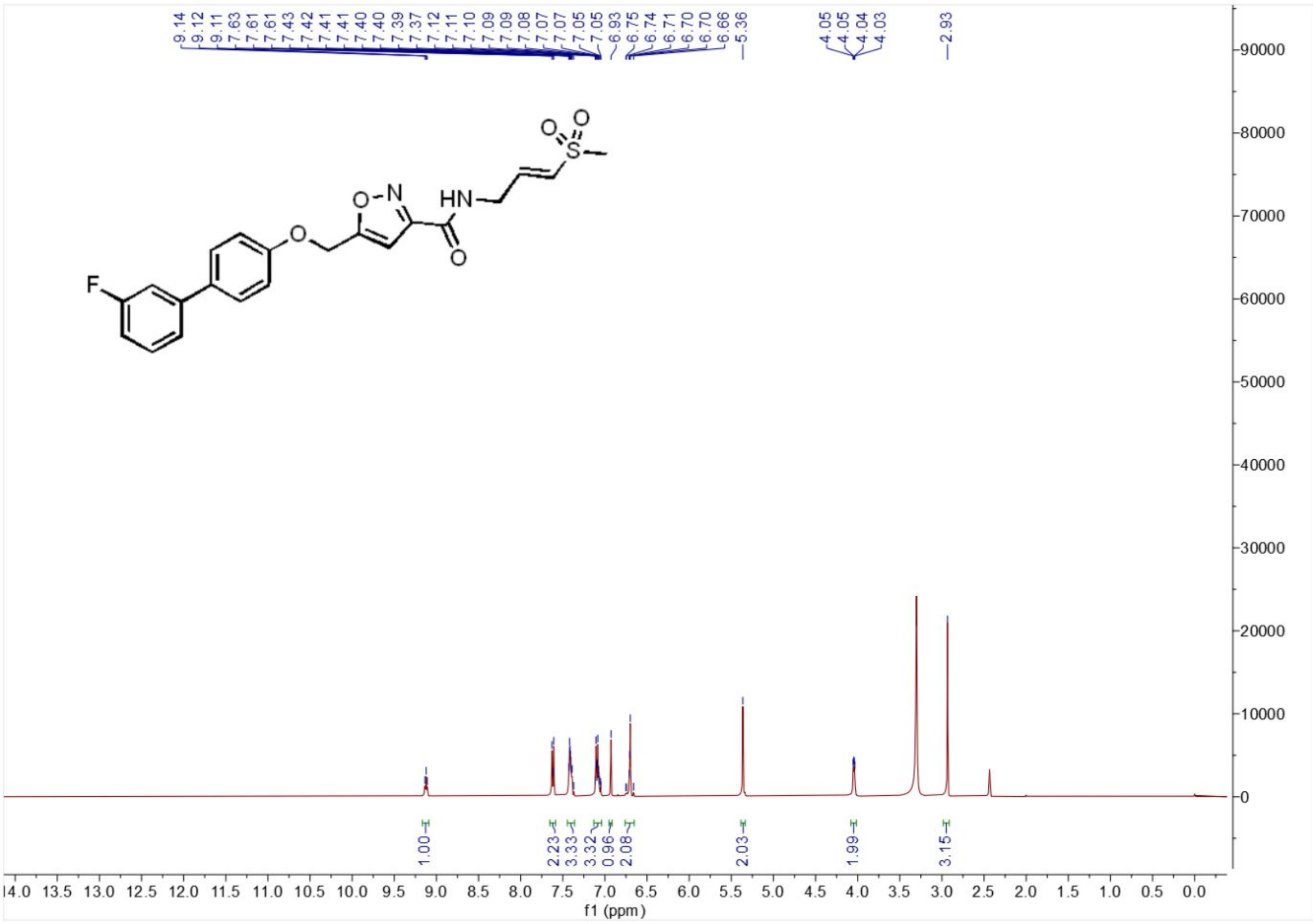

^{1}H NMR spectra of **Jun14782**

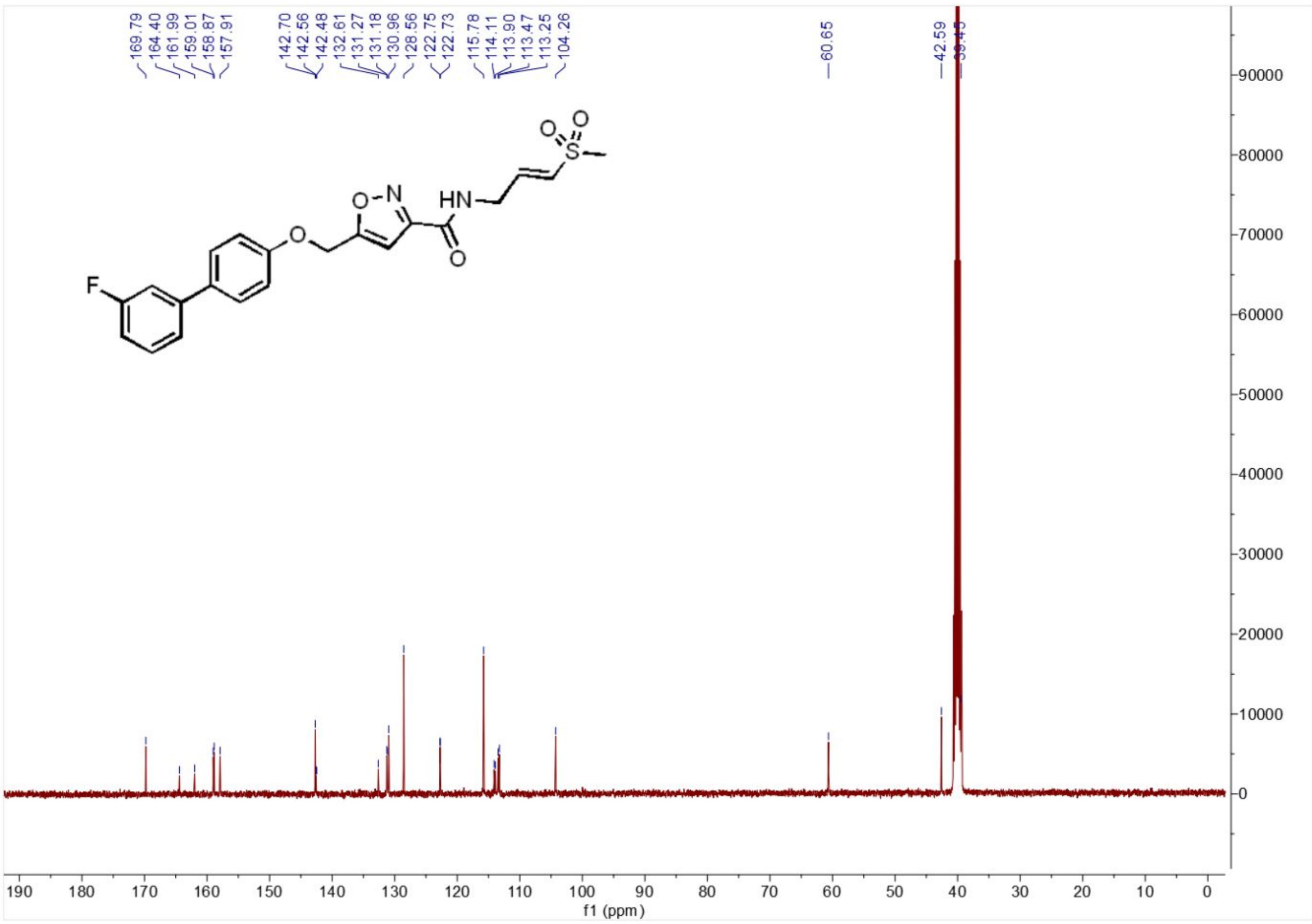

^{13}C NMR spectra of **Jun14782**

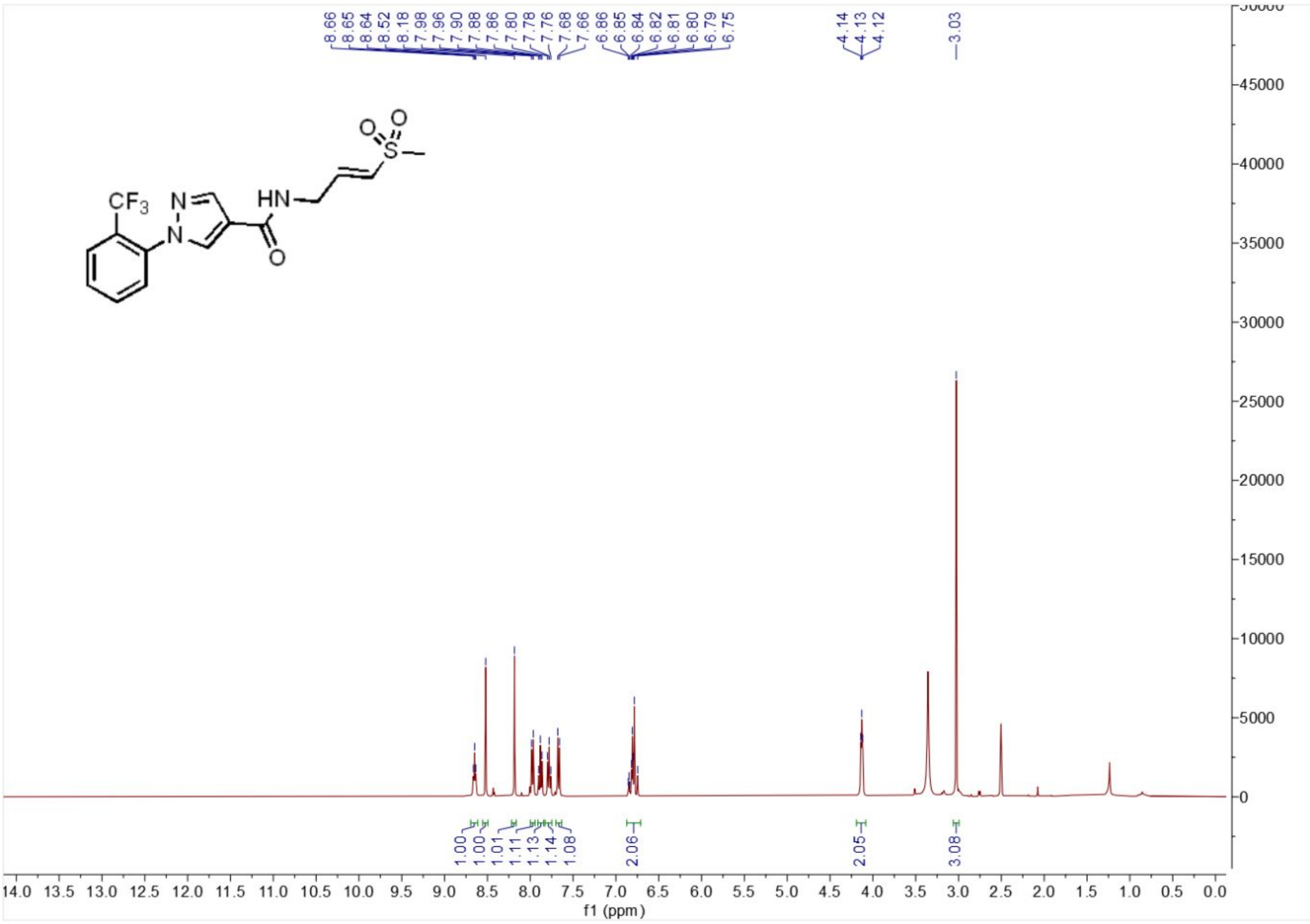

^{1}H NMR spectra of **Jun14852**

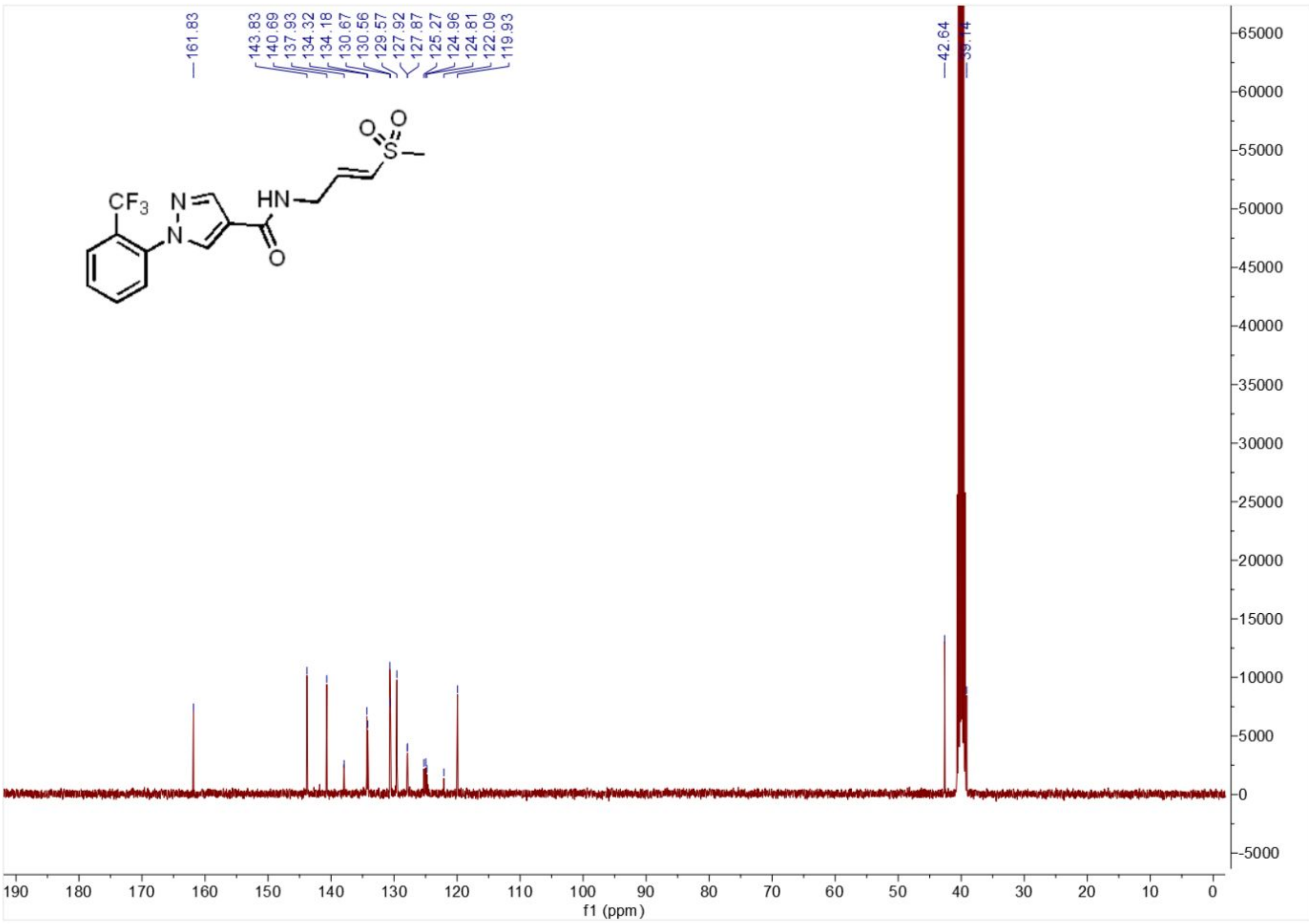

^{13}C NMR spectra of **Jun14852**

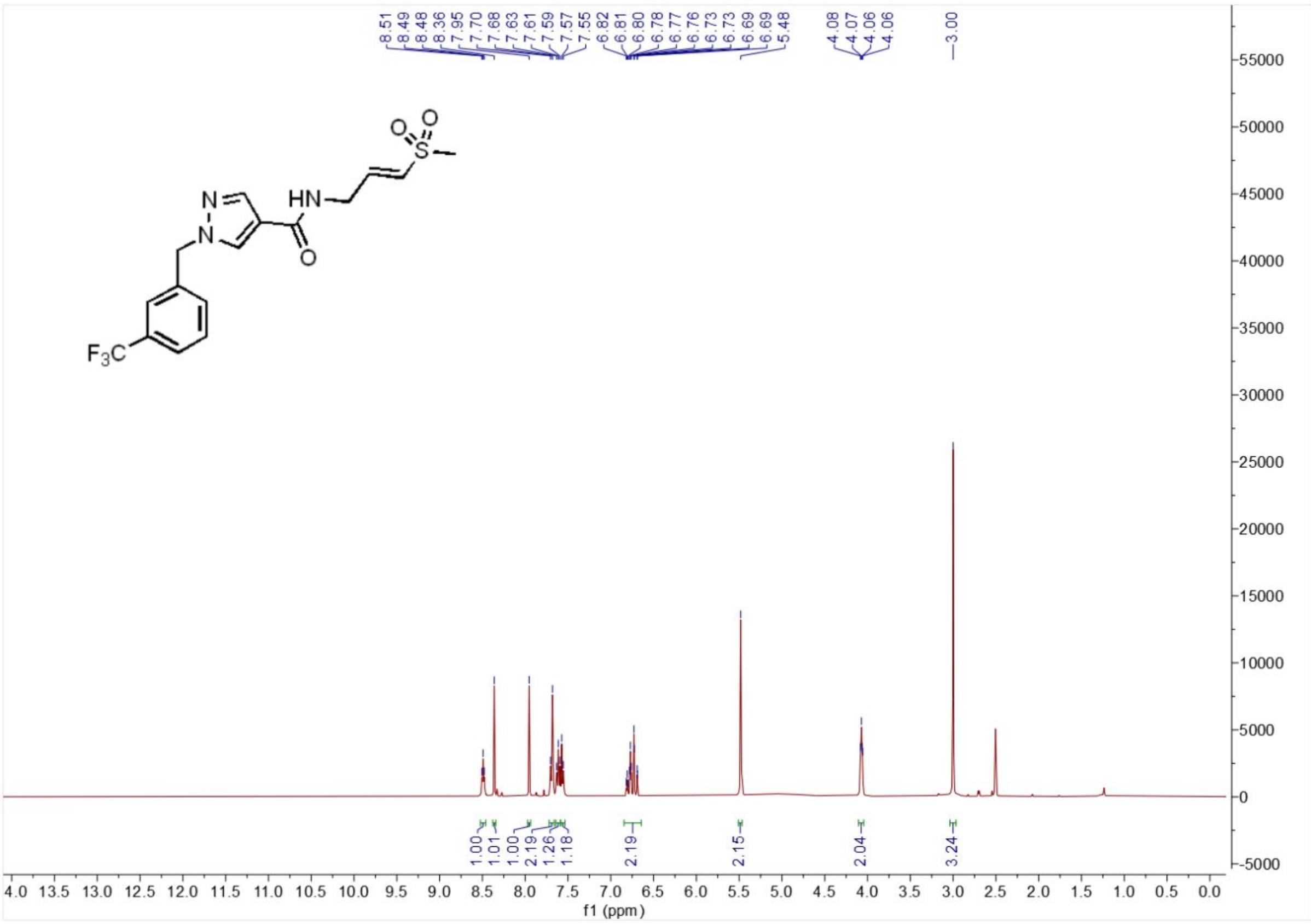

^{1}H NMR spectra of **Jun14853**

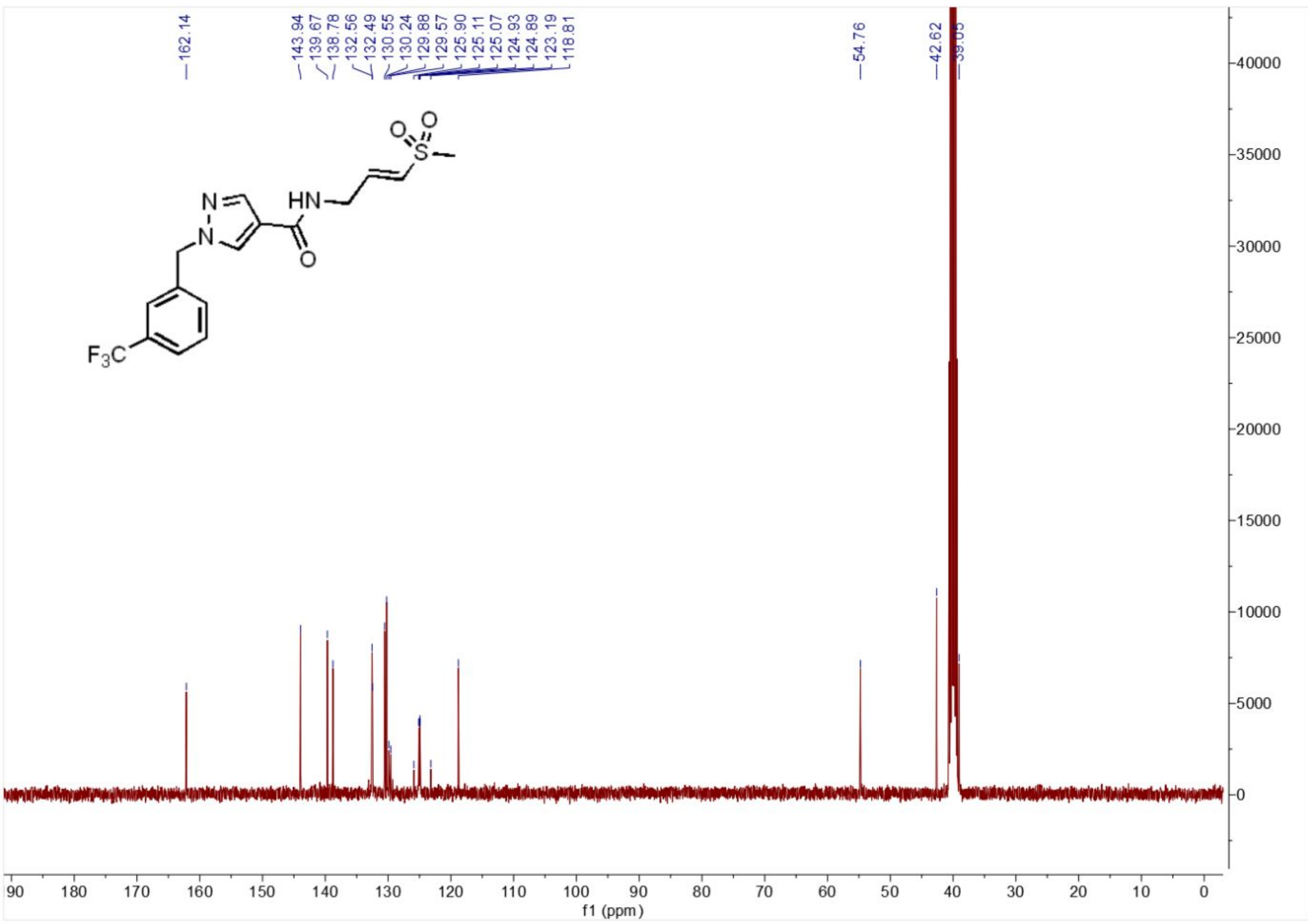

^{13}C NMR spectra of **Jun14853**

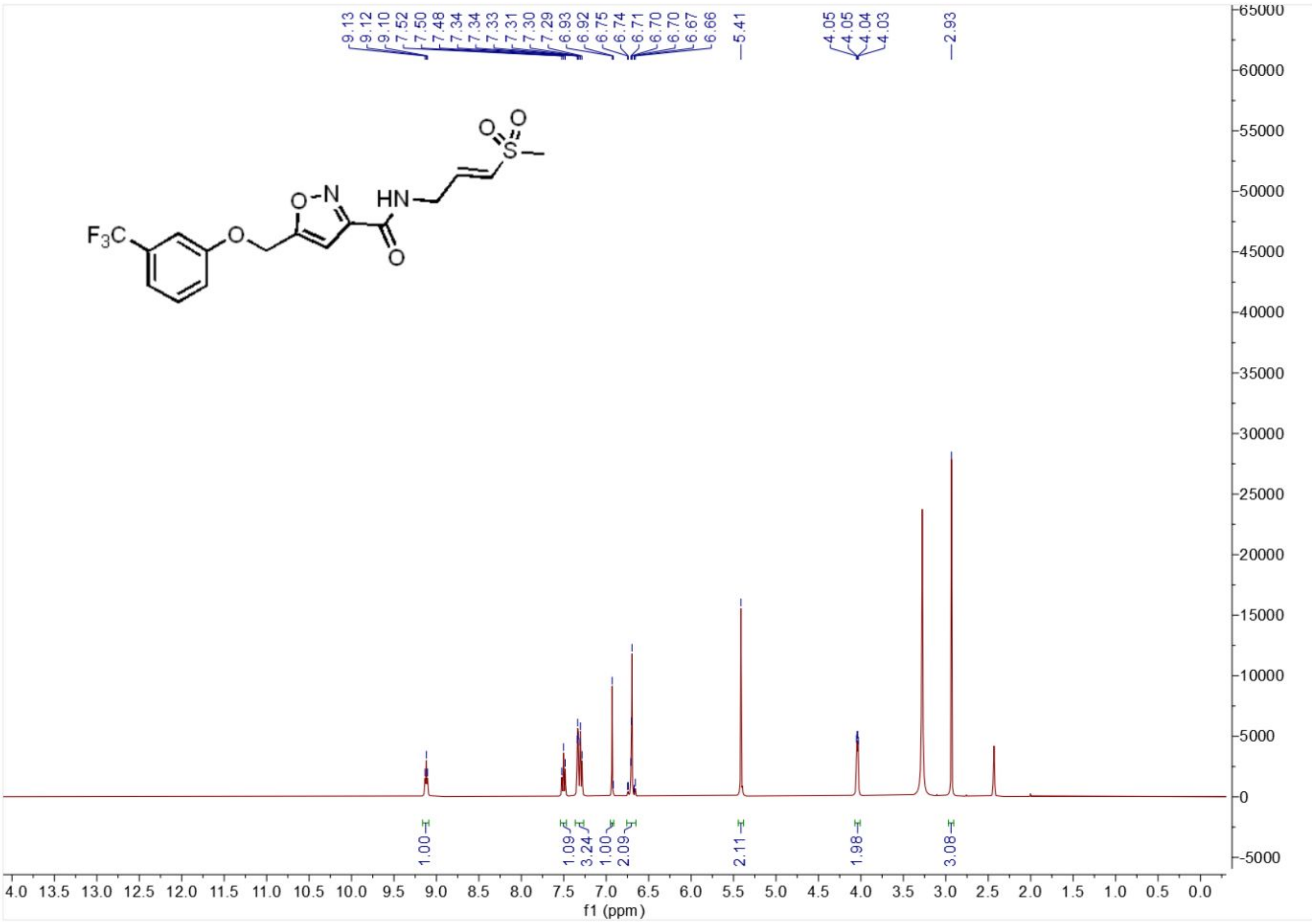

^{1}H NMR spectra of **Jun14855**

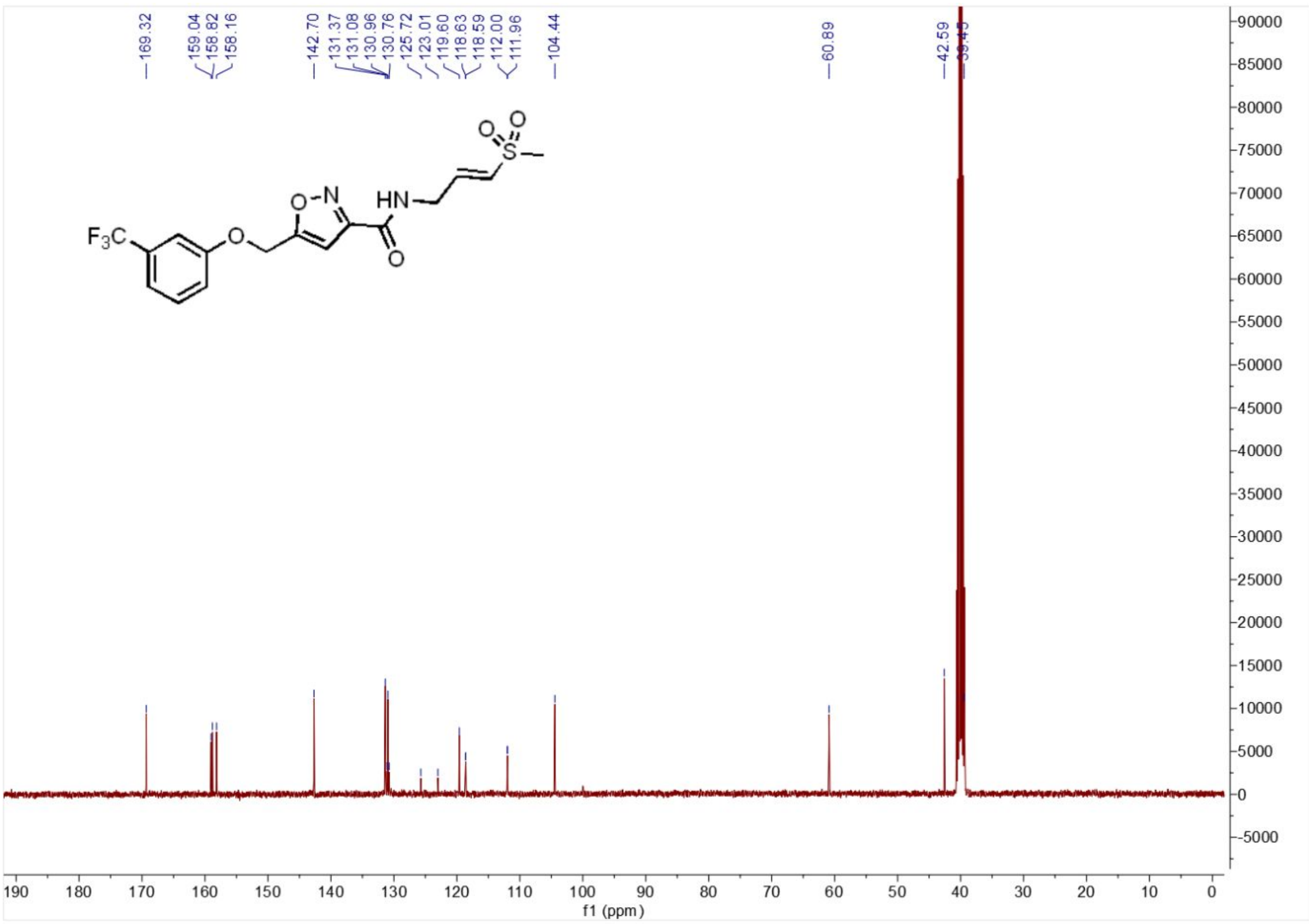

^{13}C NMR spectra of **Jun14855**

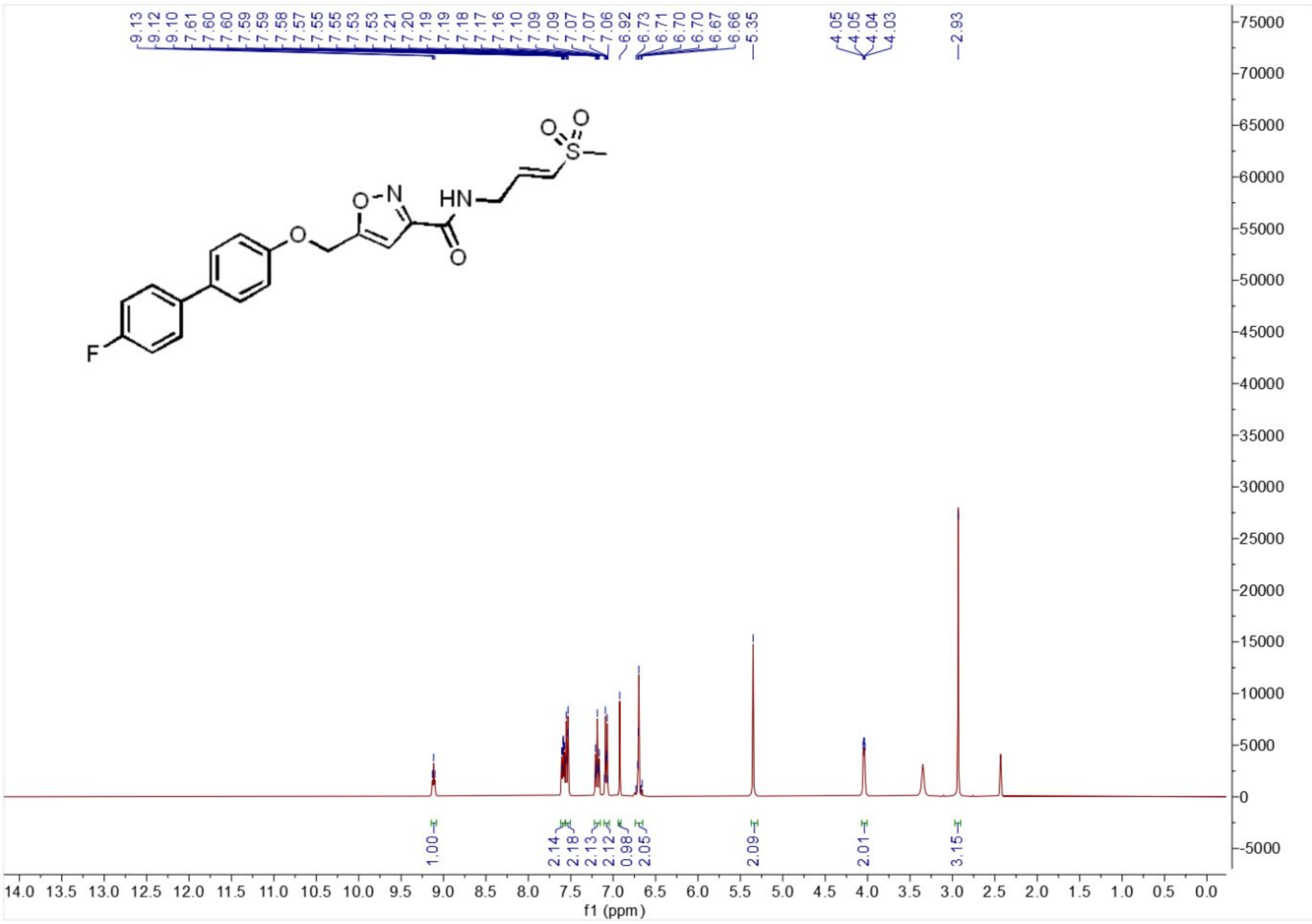

^{1}H NMR spectra of **Jun14856**

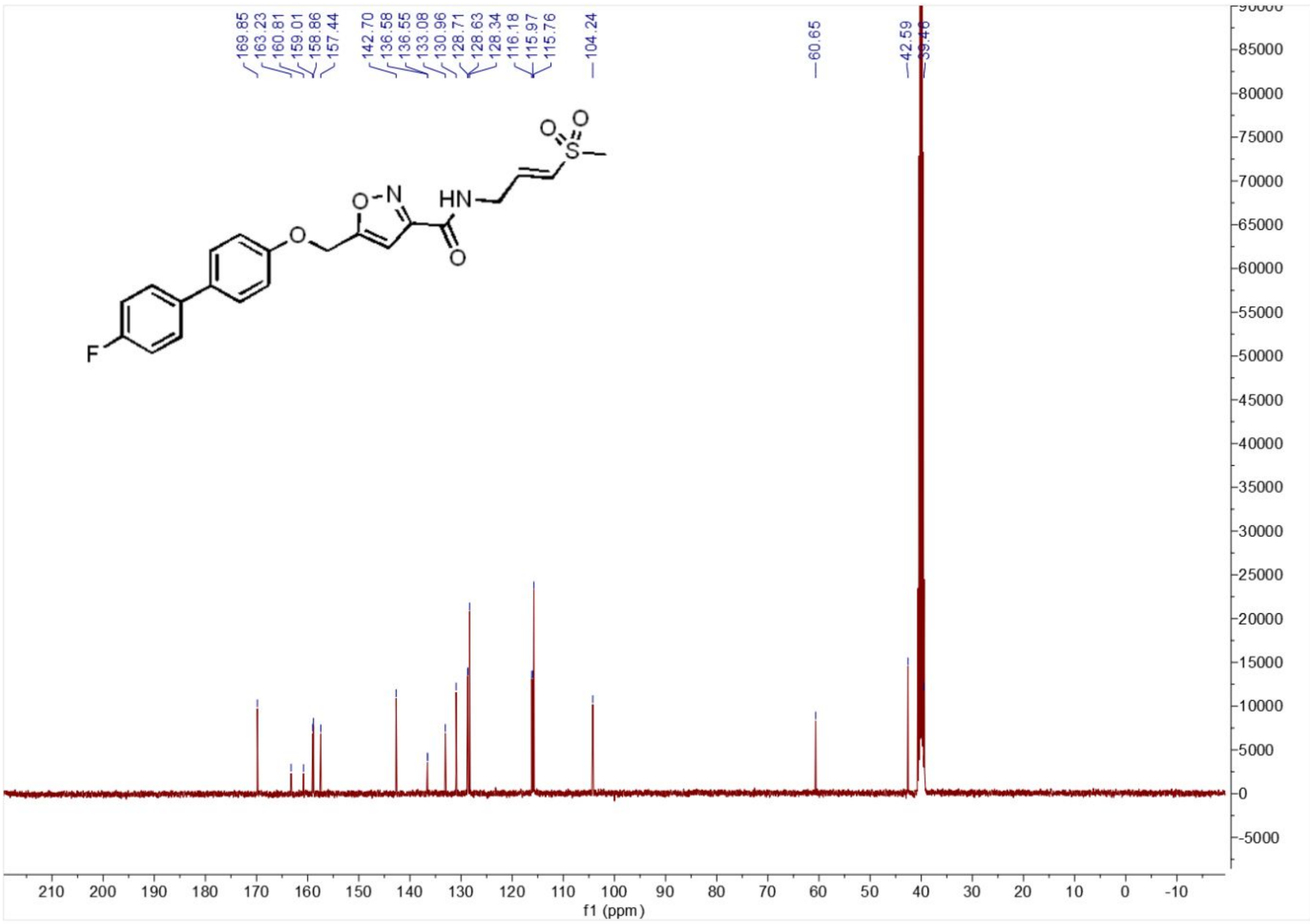

^{13}C NMR spectra of **Jun14856**

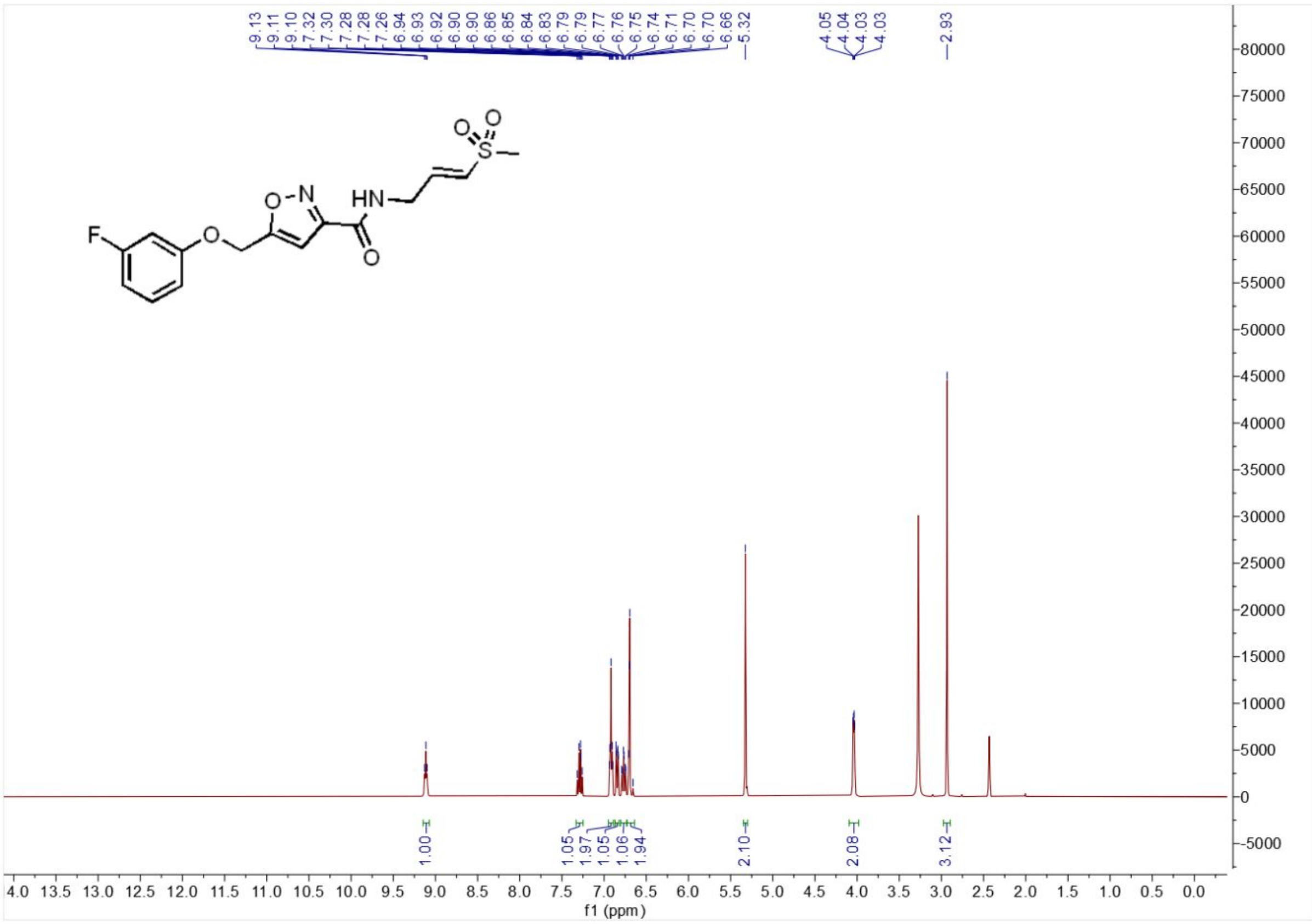

^{1}H NMR spectra of **Jun14861**

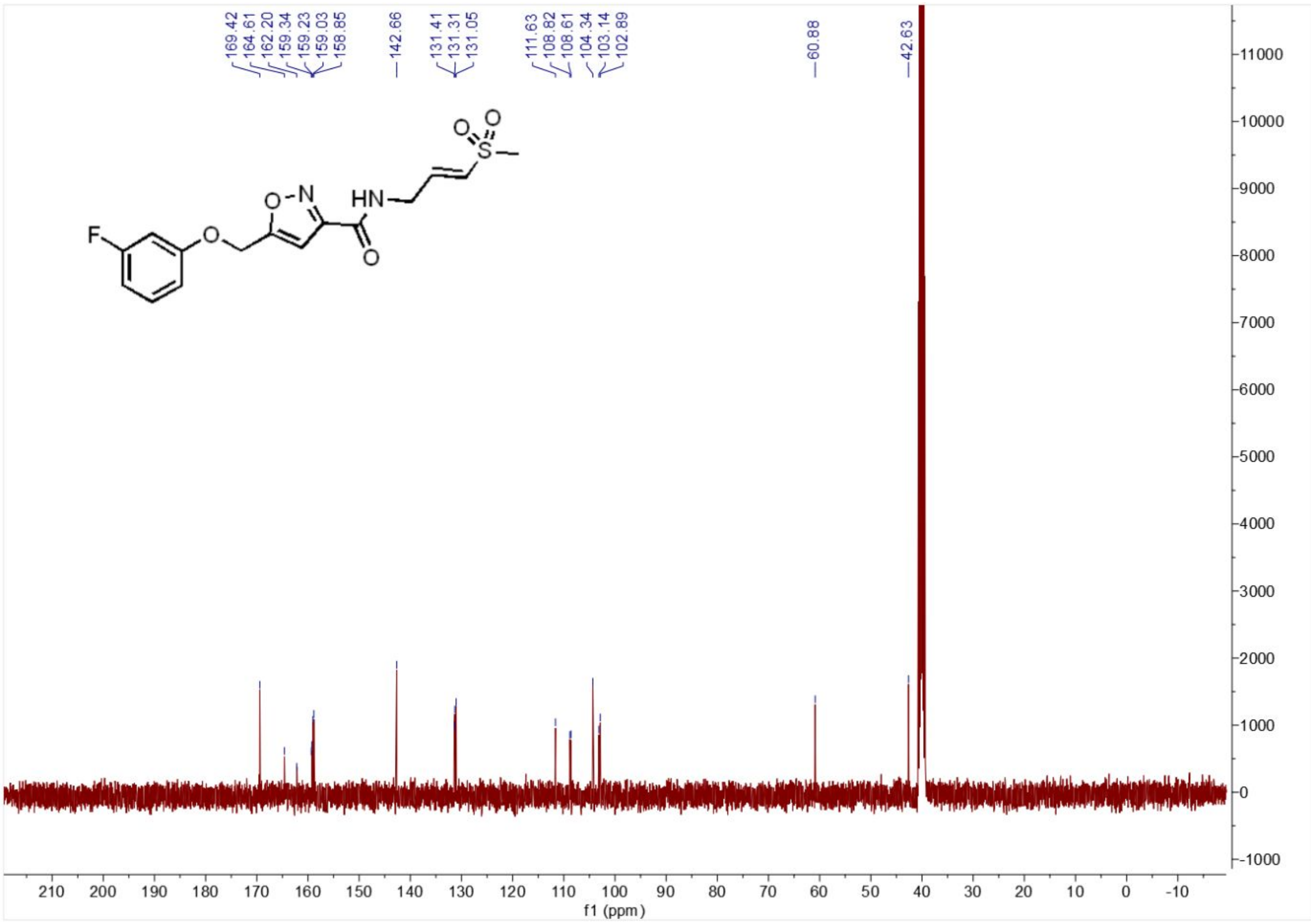

^{13}C NMR spectra of **Jun14861**

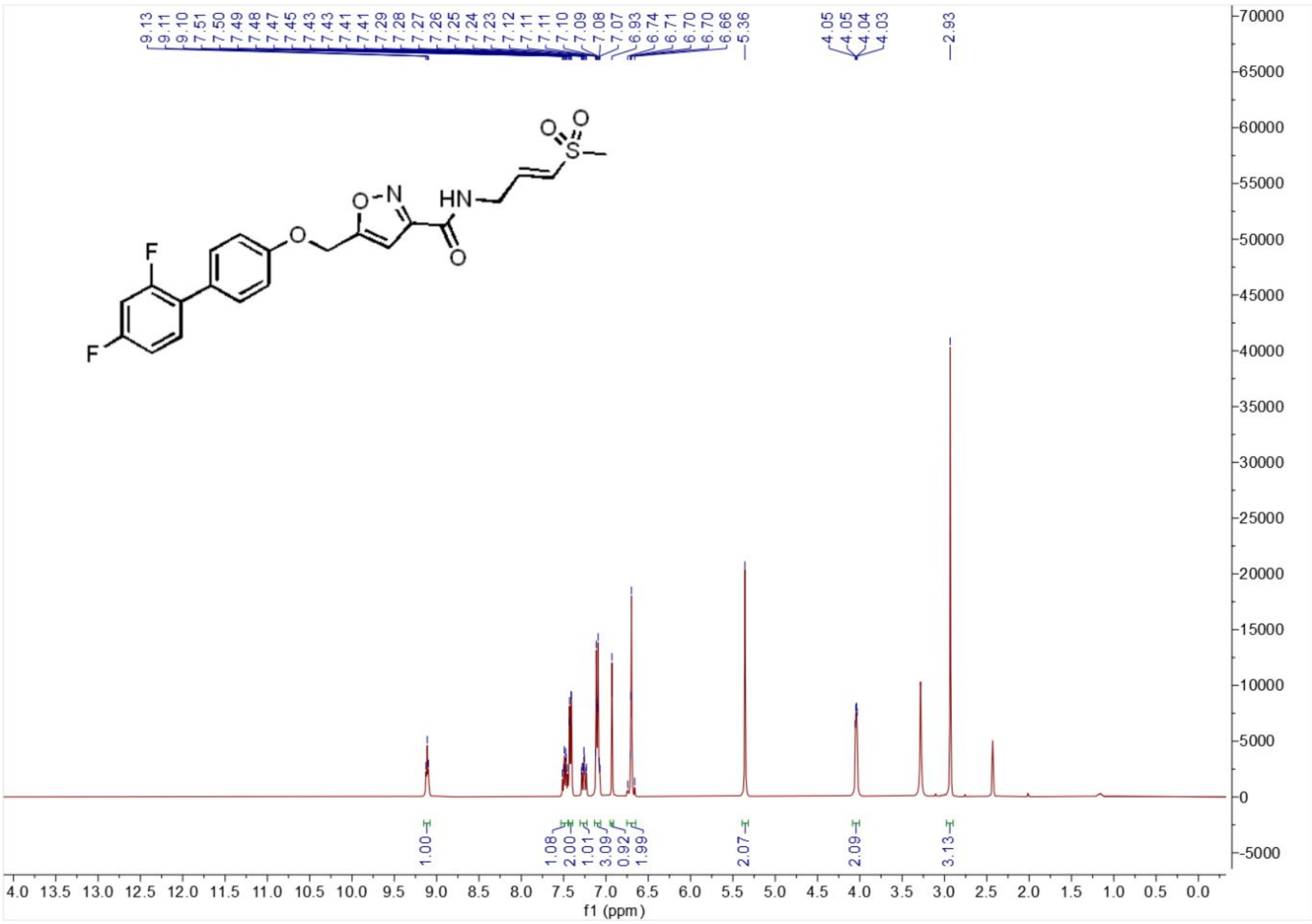

^{1}H NMR spectra of **Jun14897**

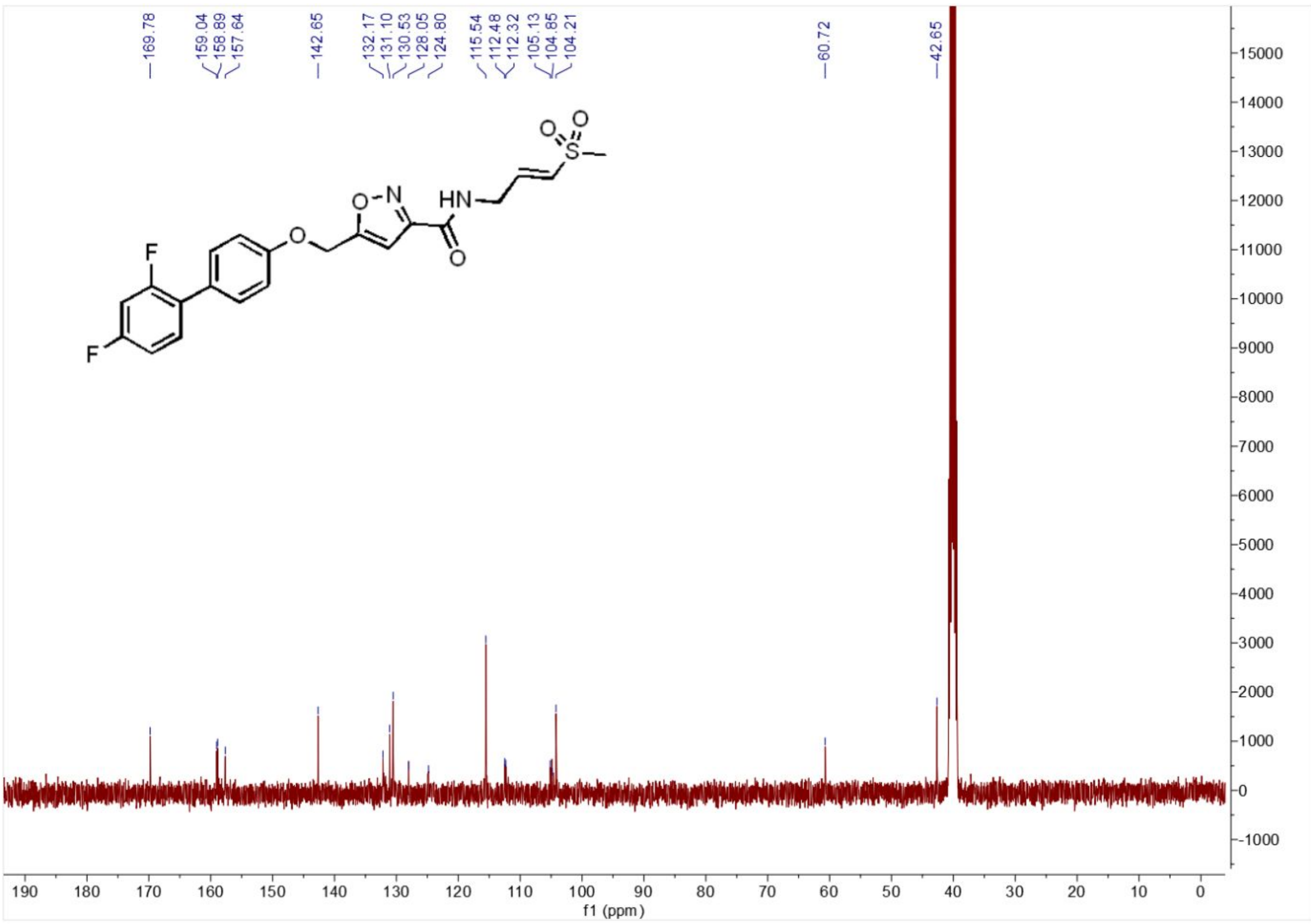

^{13}C NMR spectra of **Jun14897**

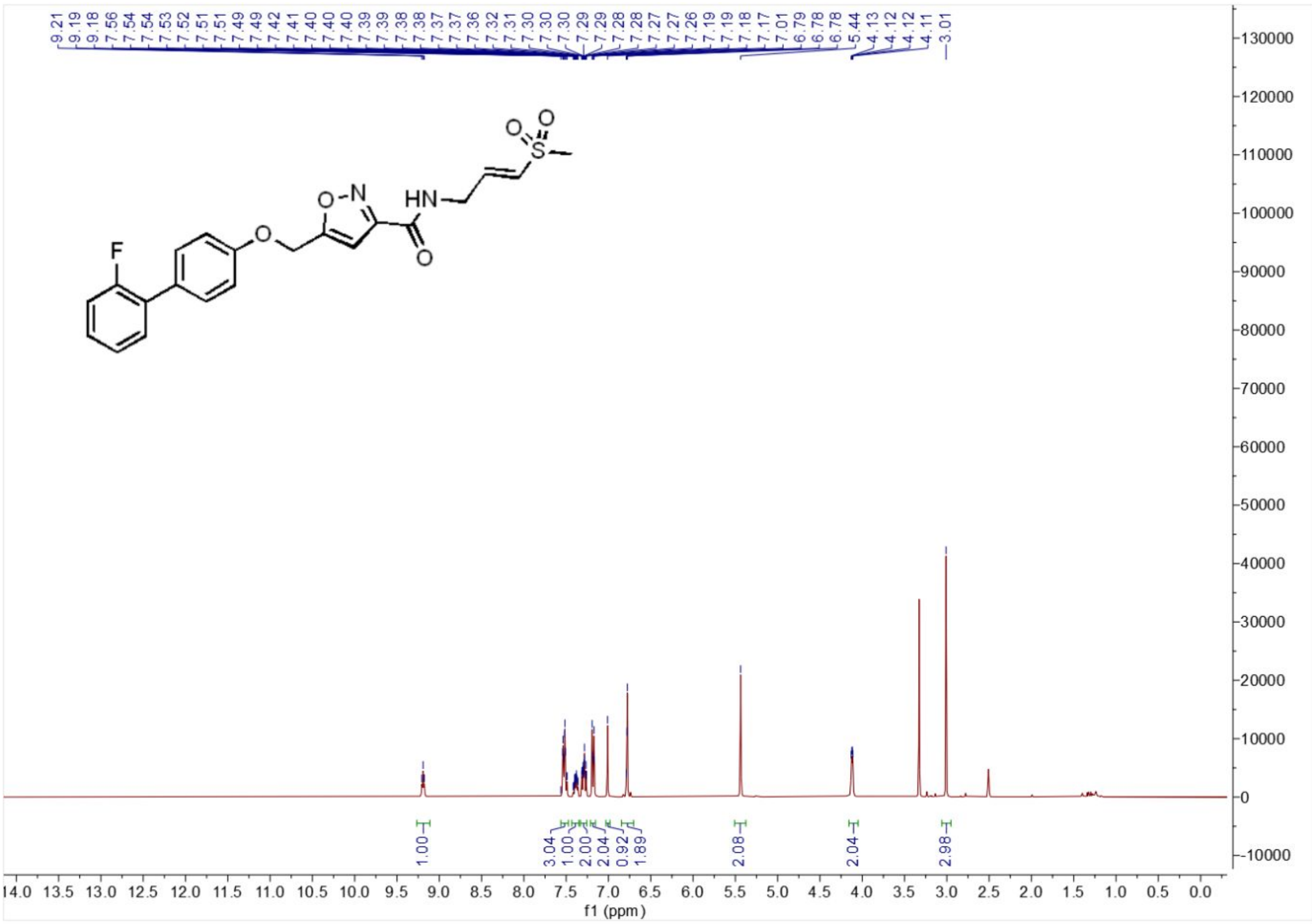

^{1}H NMR spectra of **Jun14924**

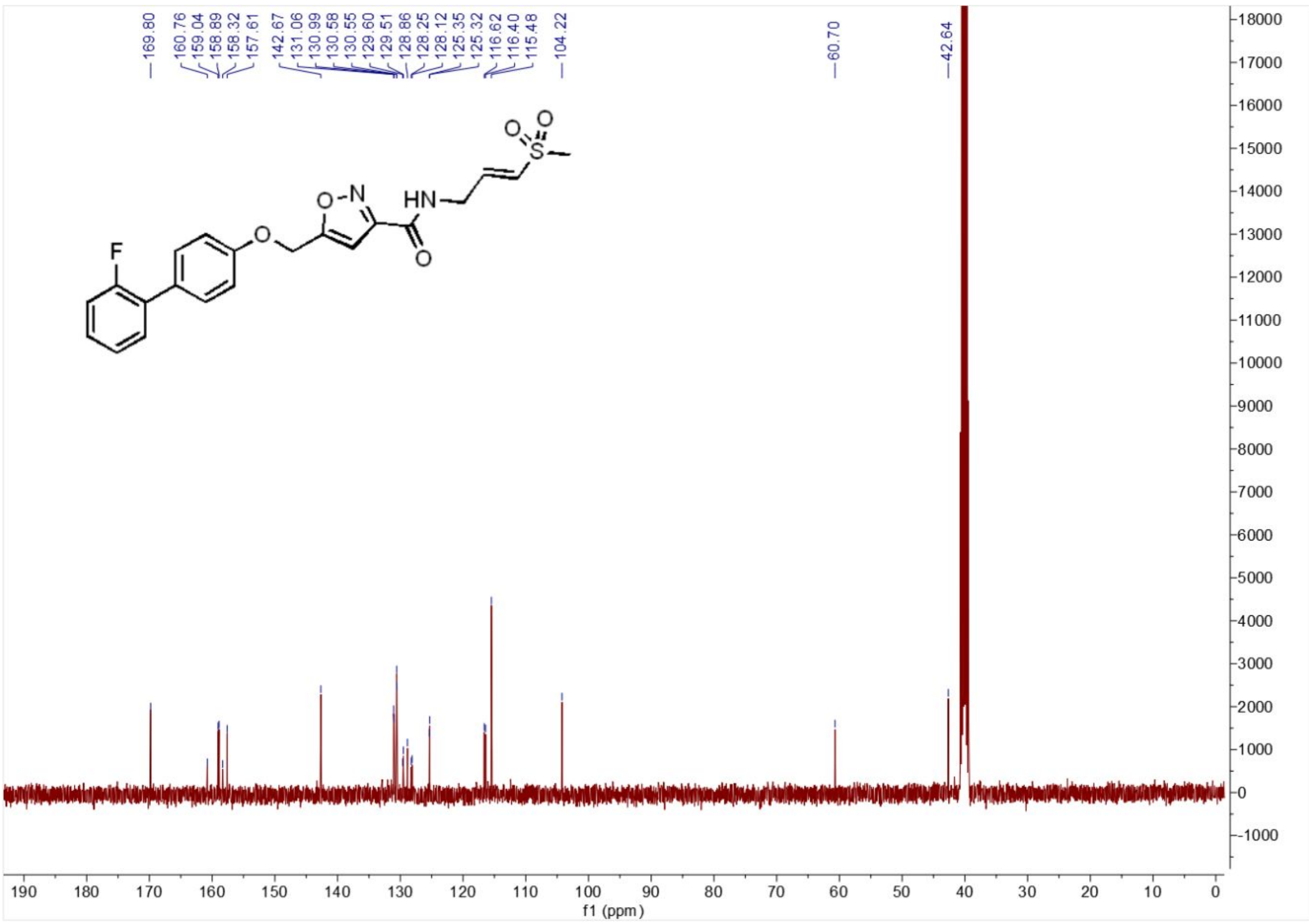

^{13}C NMR spectra of **Jun14924**

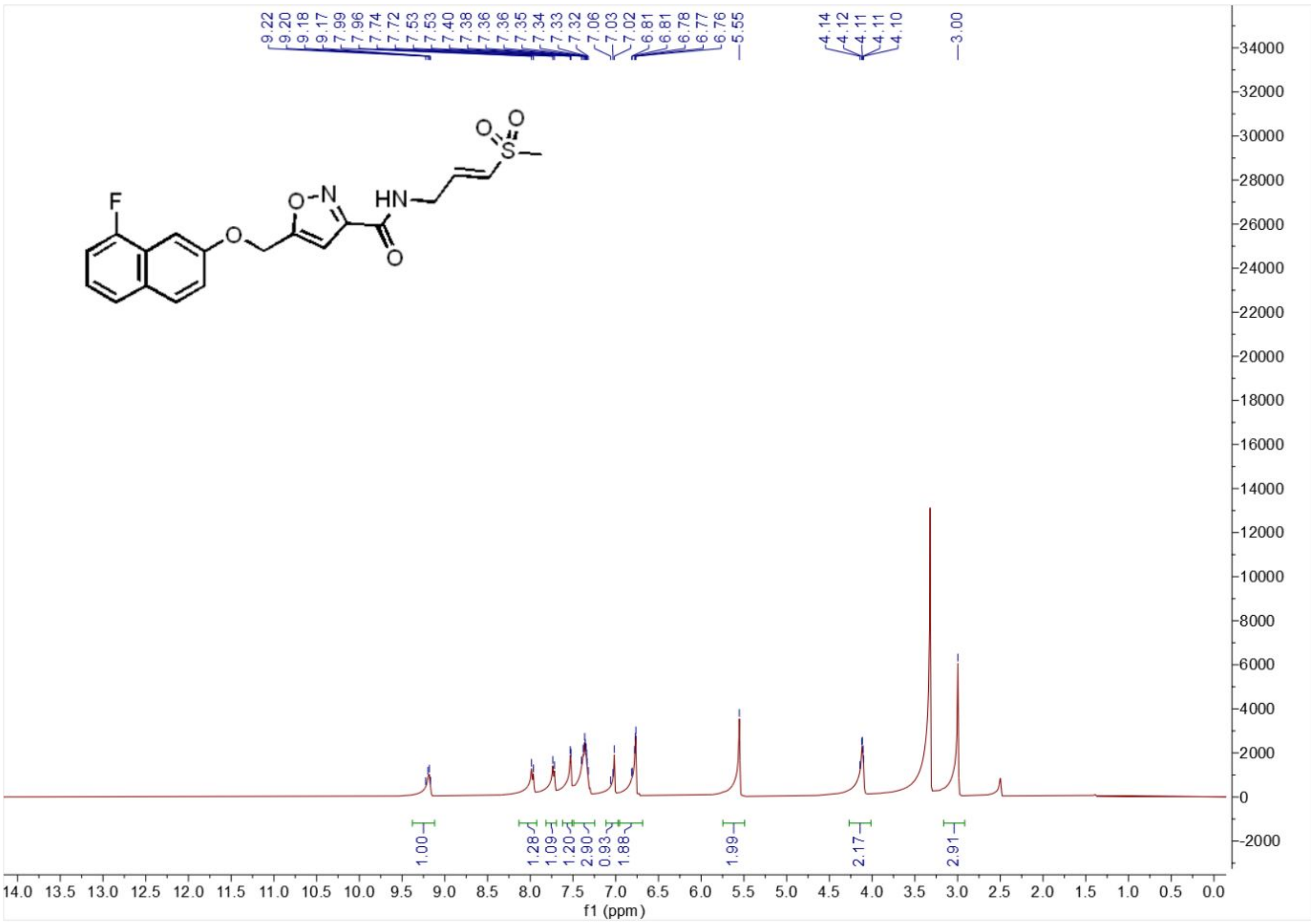

^{1}H NMR spectra of **Jun14926**

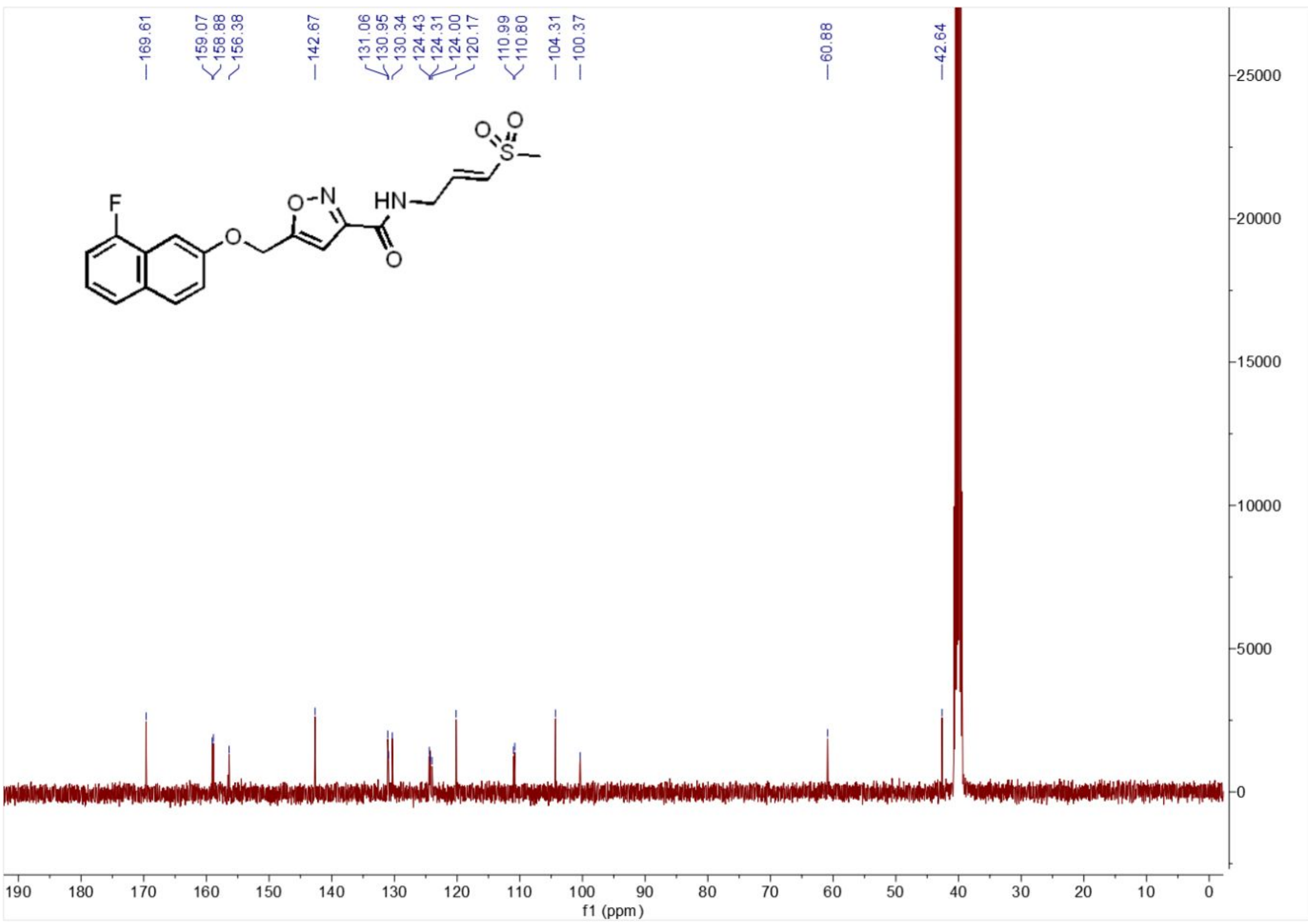

^{13}C NMR spectra of **Jun14926**

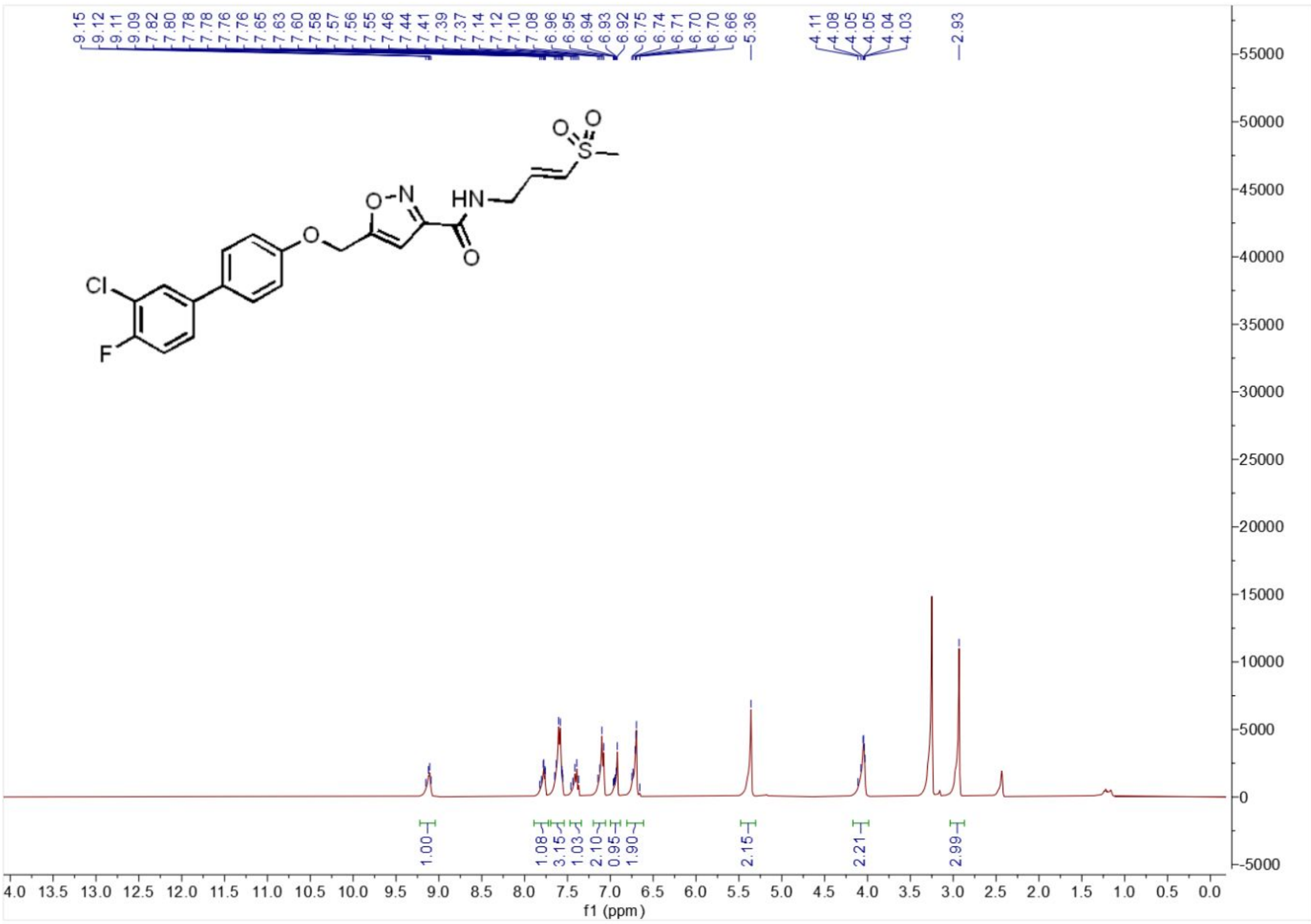

^{1}H NMR spectra of **Jun14927**

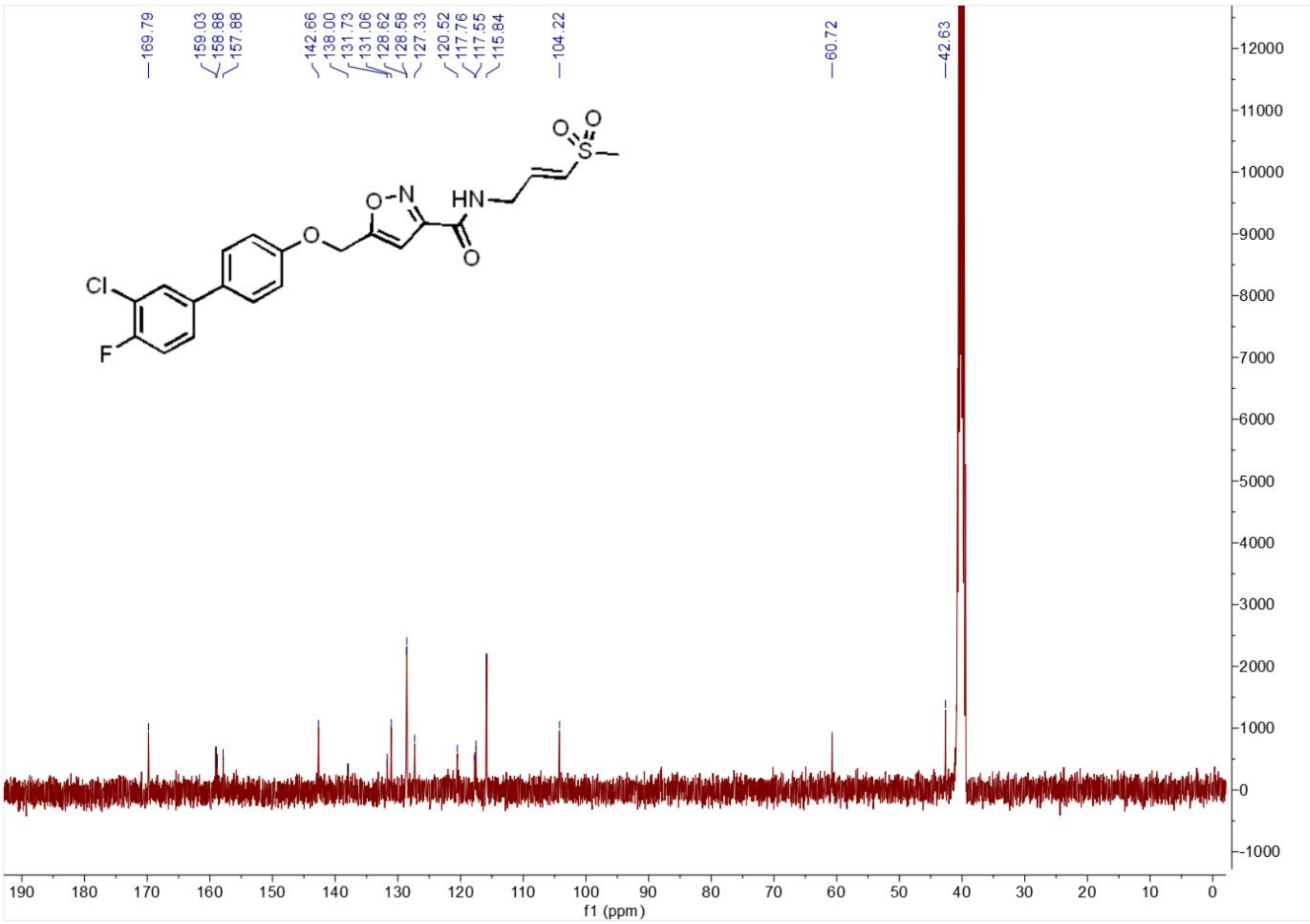

^{13}C NMR spectra of **Jun14927**

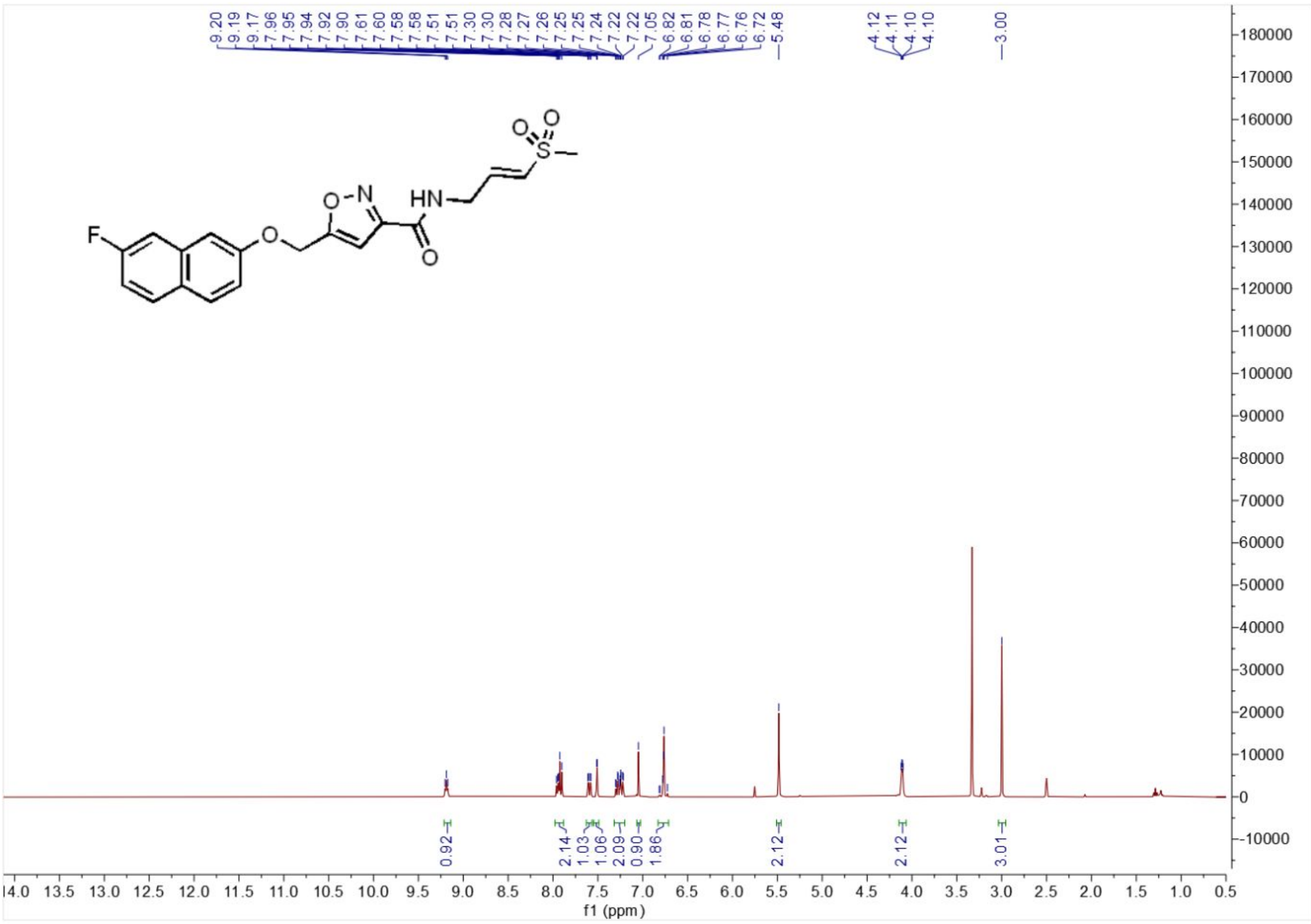

^1H NMR spectra of **Jun14966**

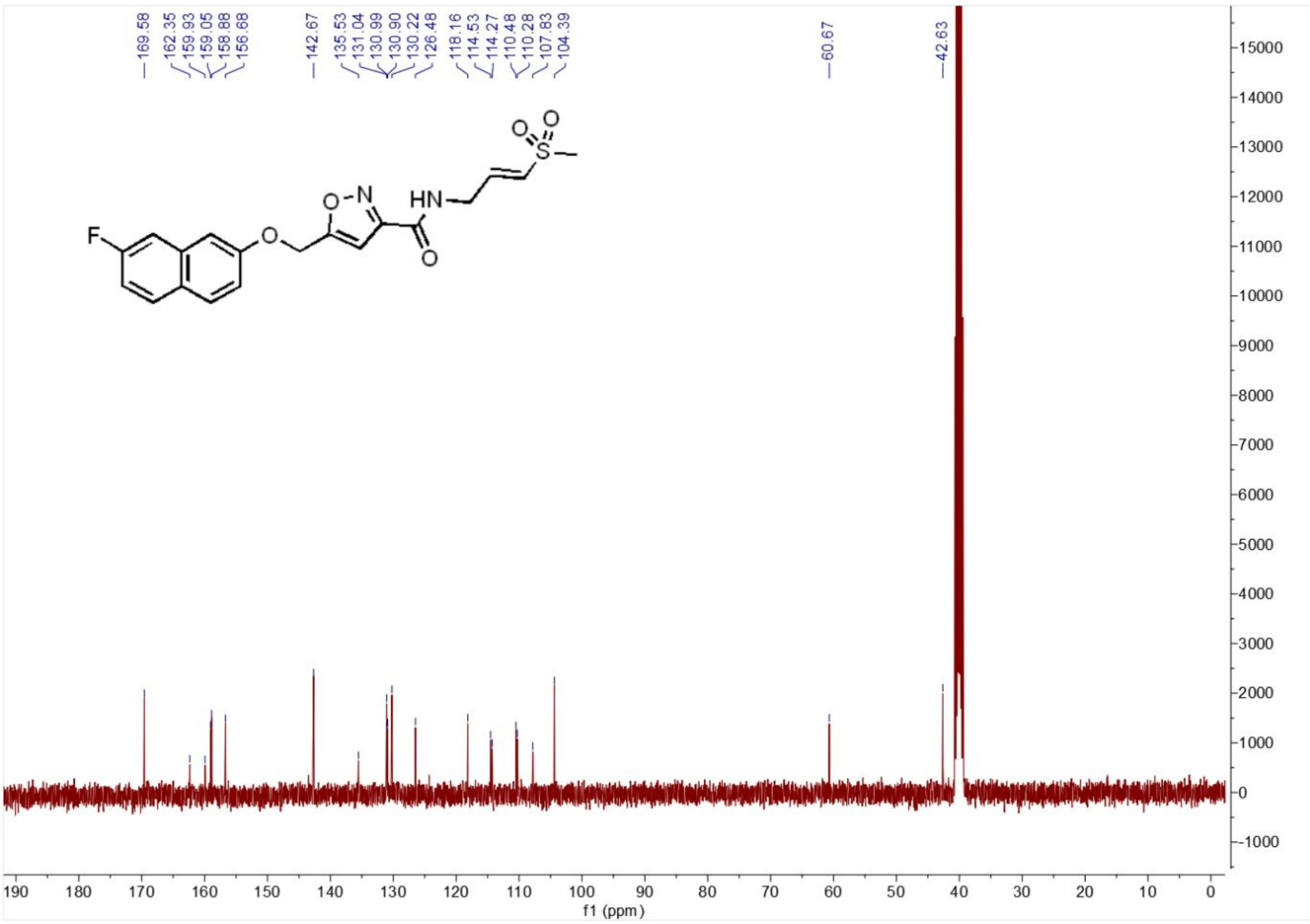

^{13}C NMR spectra of **Jun14966**

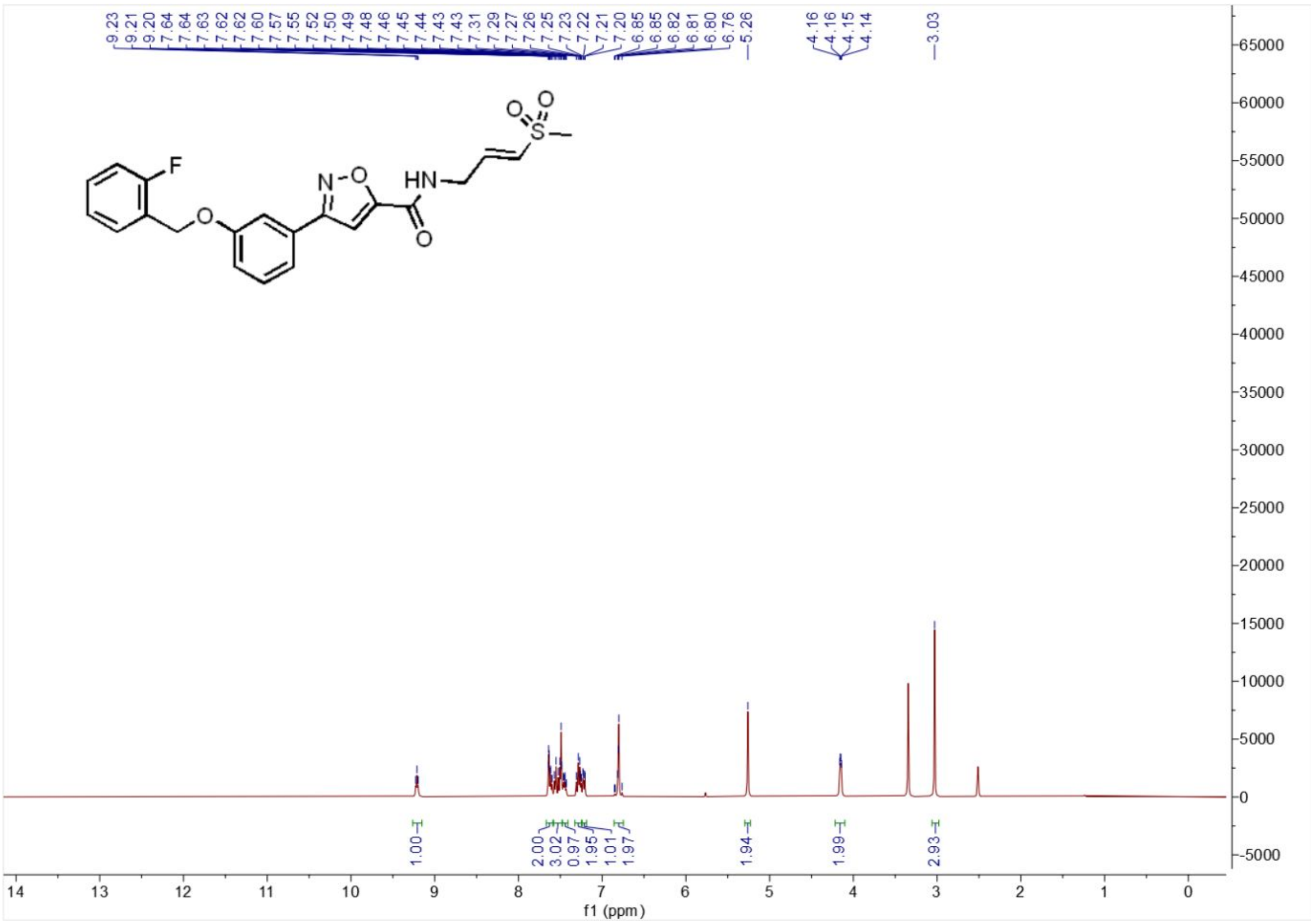

^{1}H NMR spectra of **Jun14969**

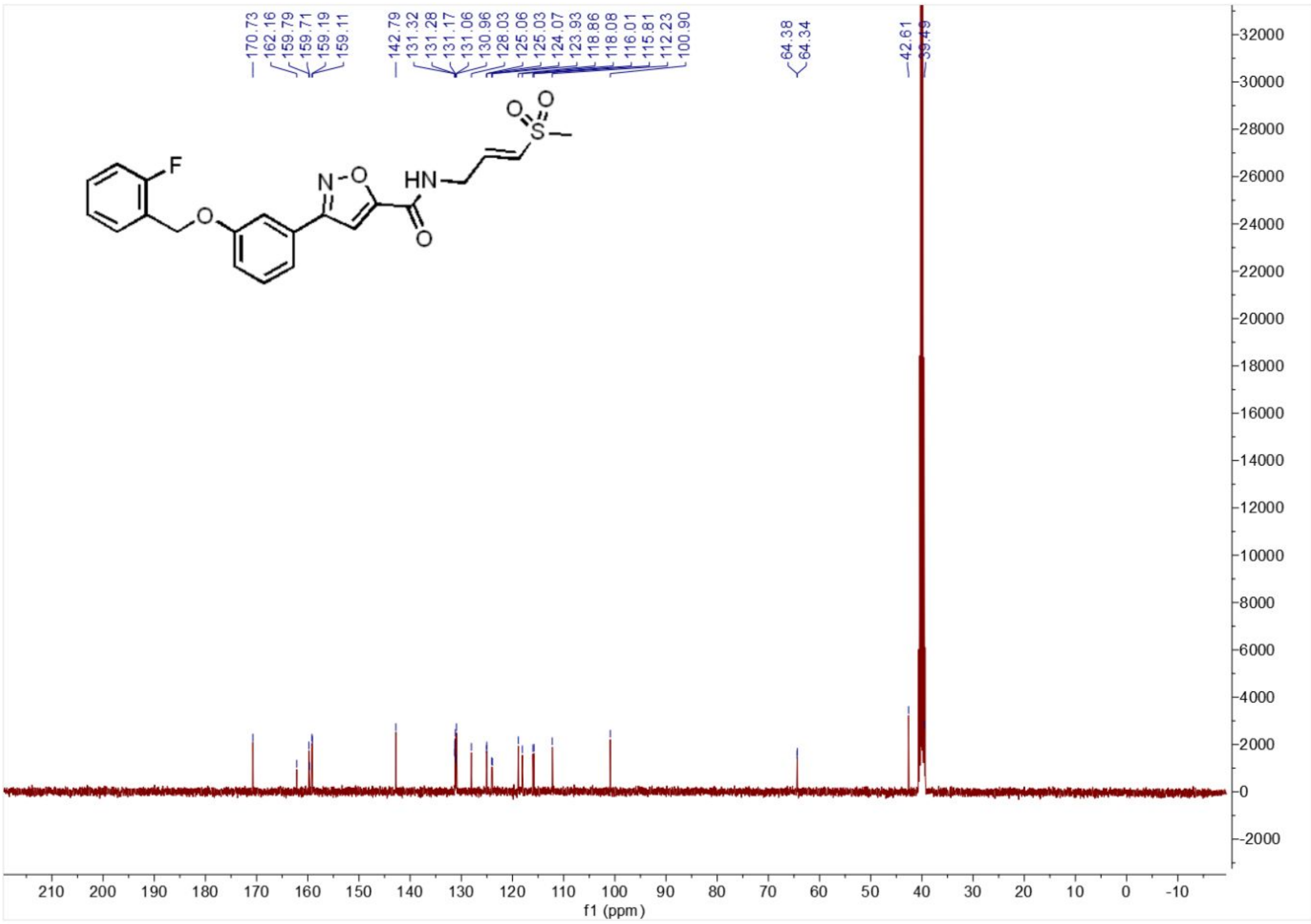

^{13}C NMR spectra of **Jun14969**

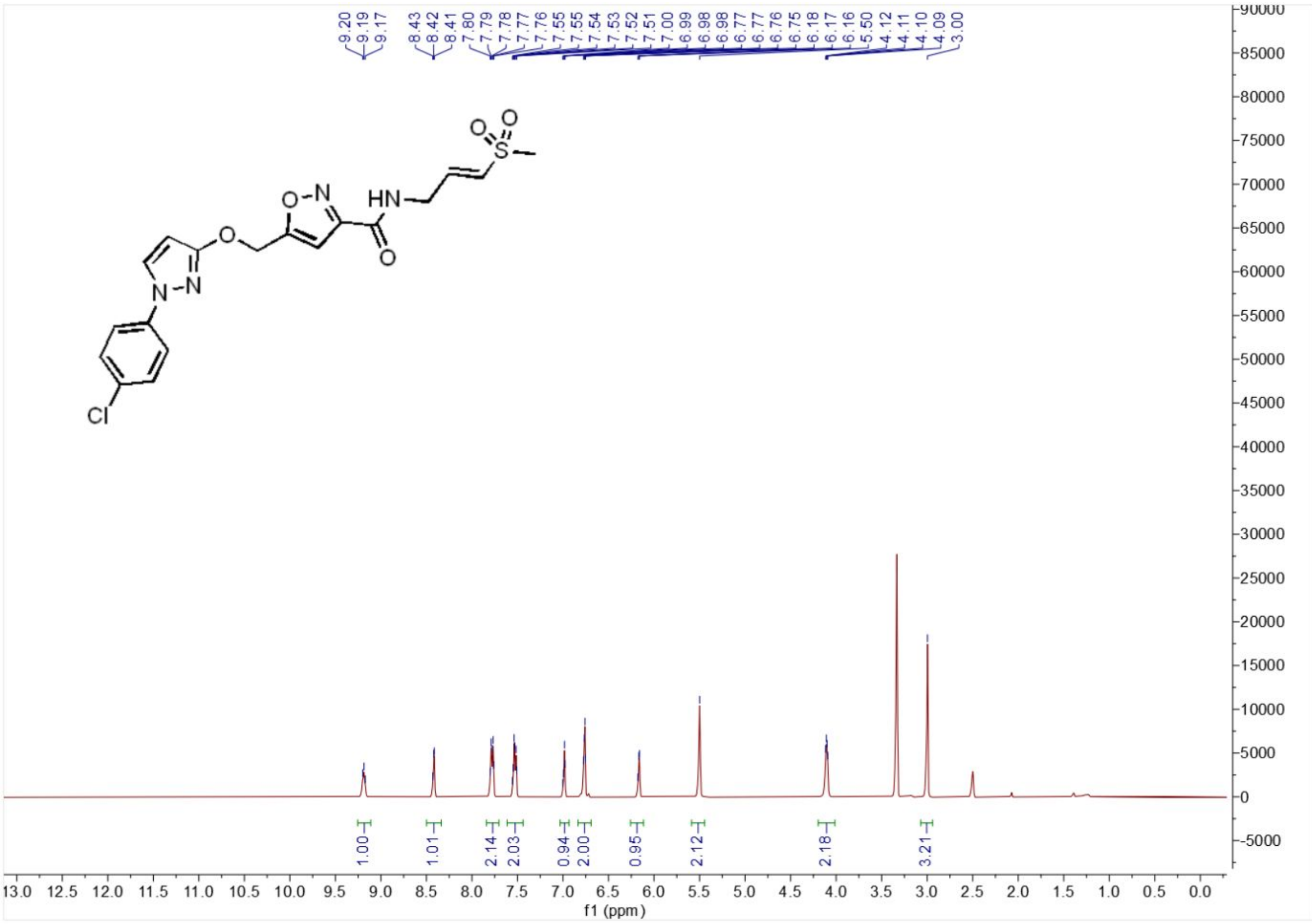

^{1}H NMR spectra of **Jun15111**

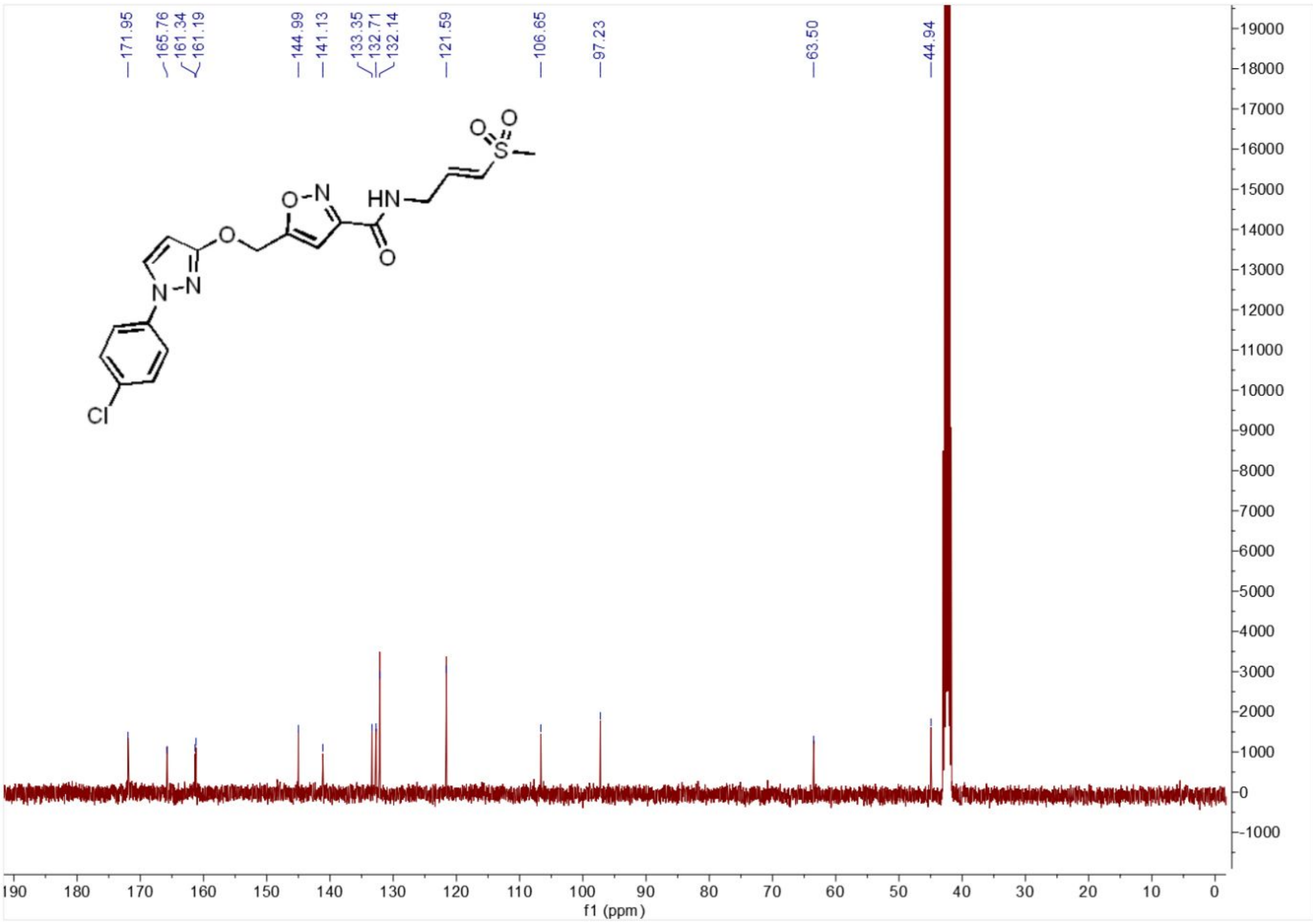

^{13}C NMR spectra of **Jun15111**

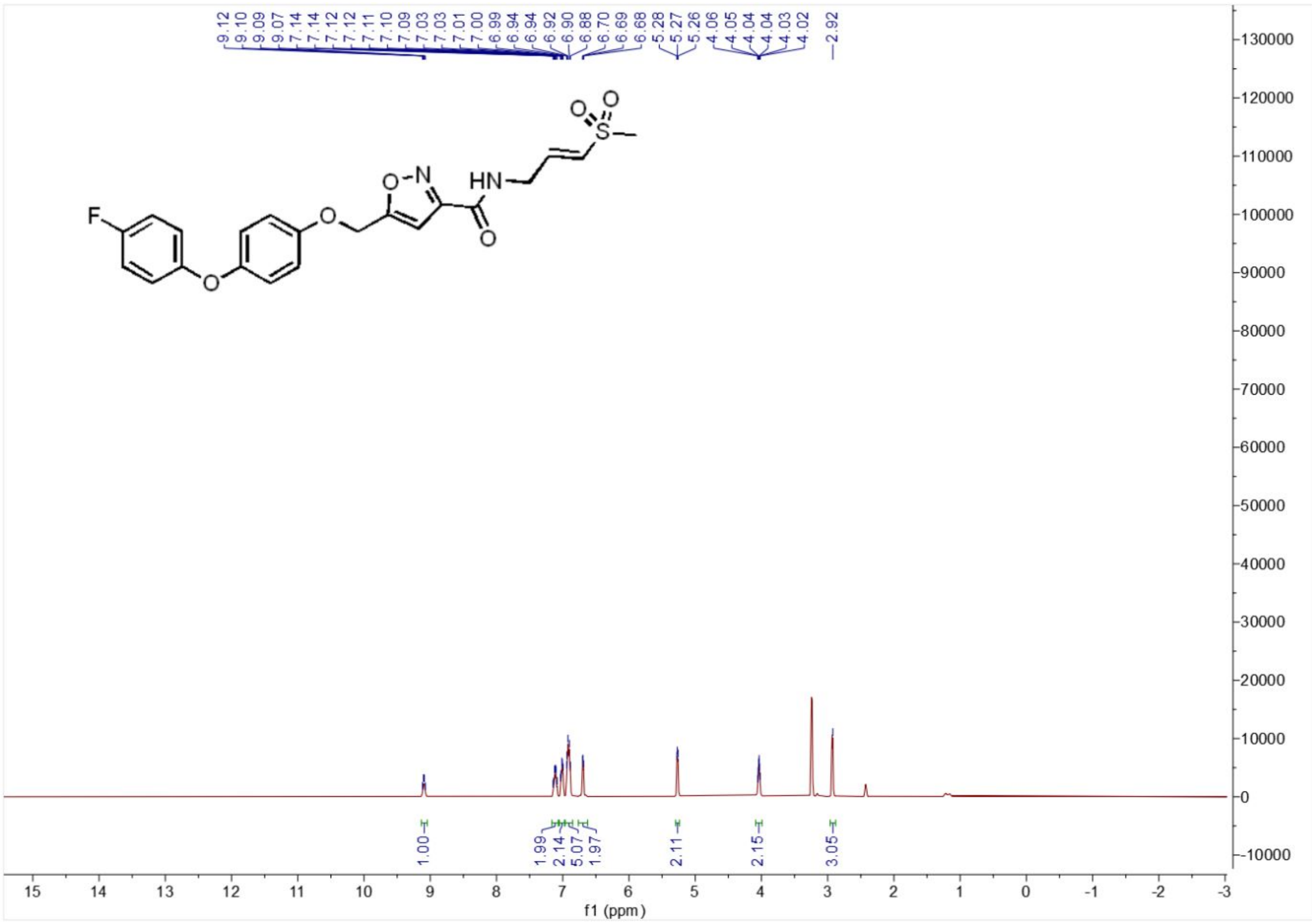

^{1}H NMR spectra of **Jun1551**

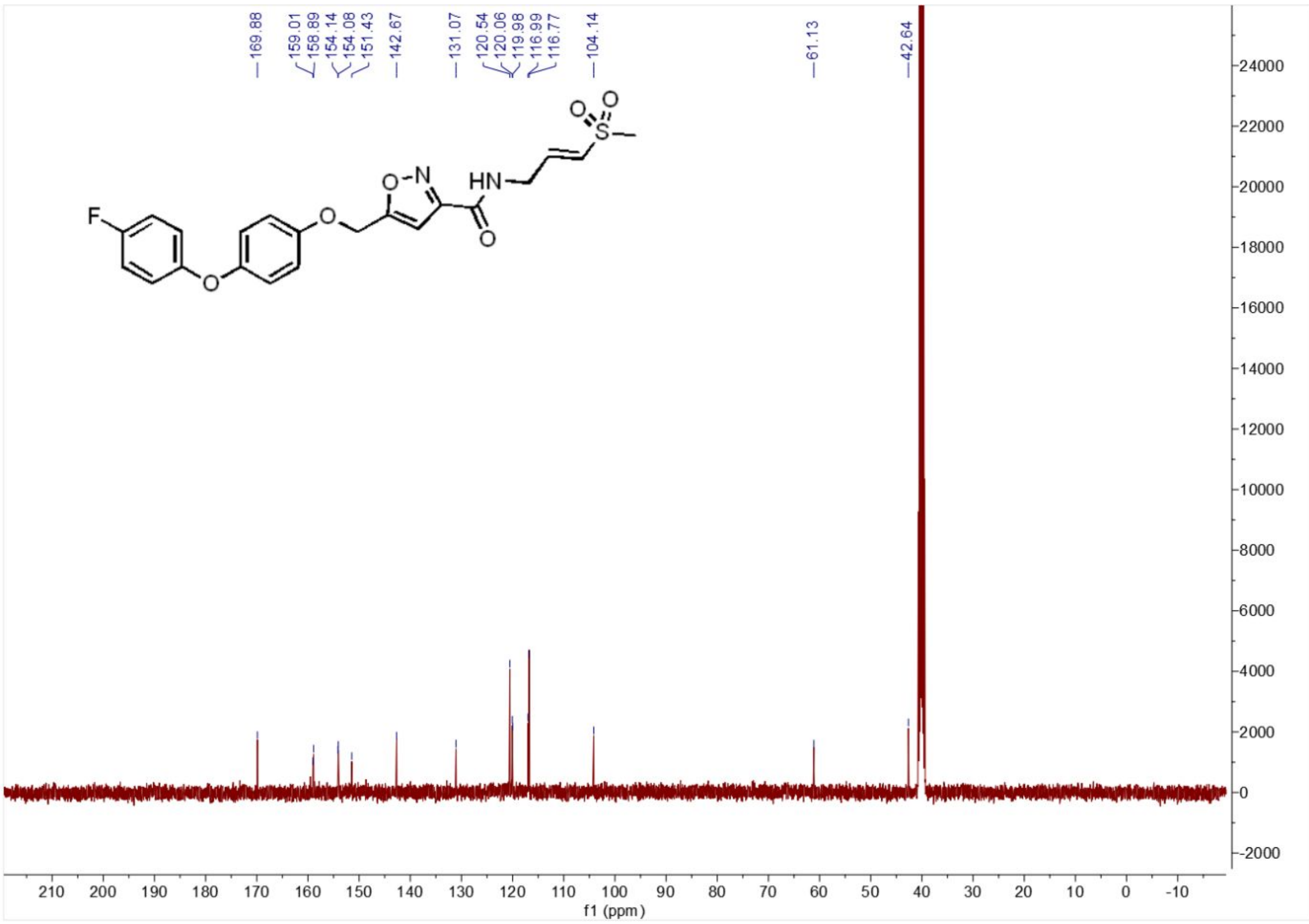

^{13}C NMR spectra of **Jun1551**

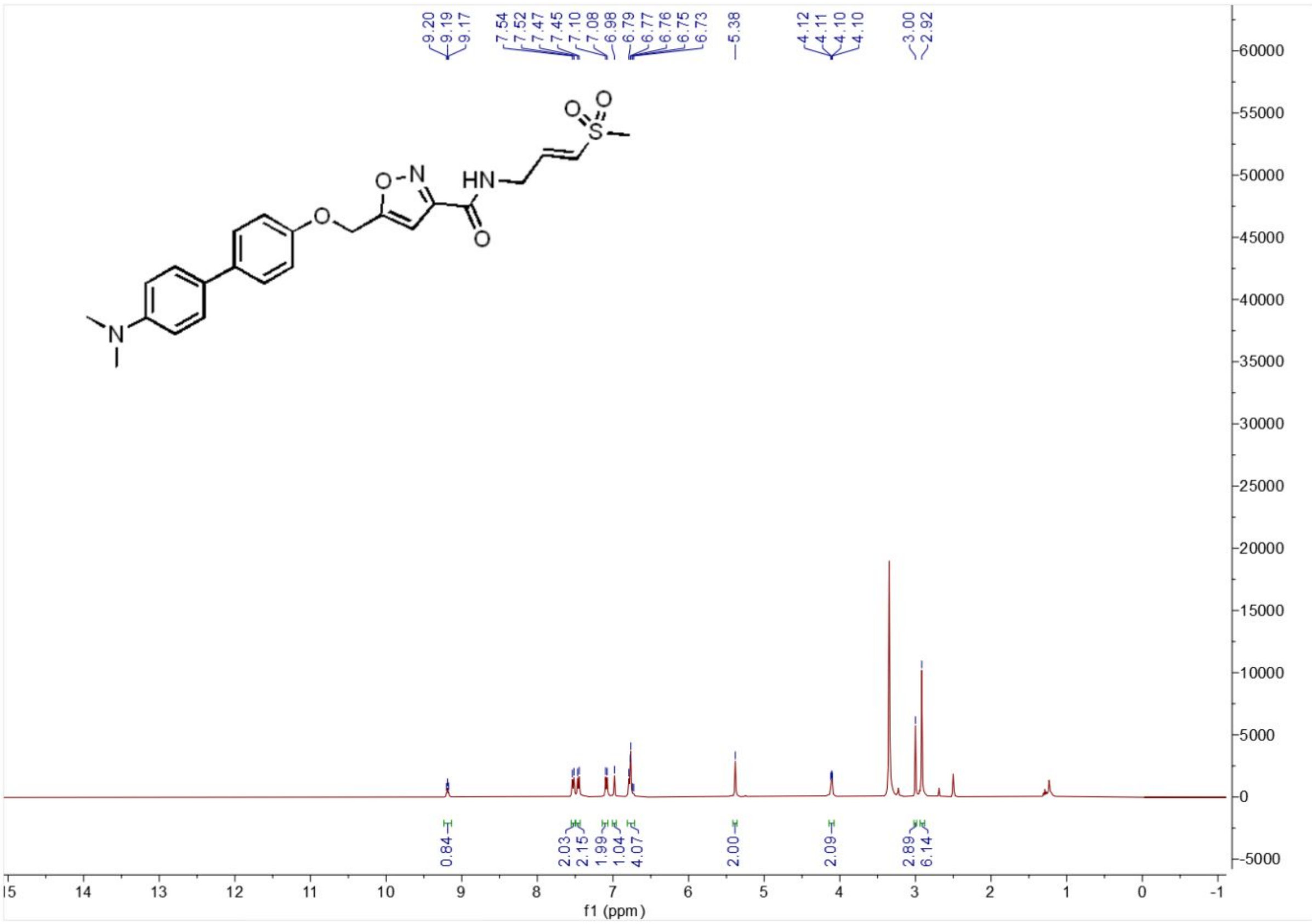

^{1}H NMR spectra of **Jun15176**

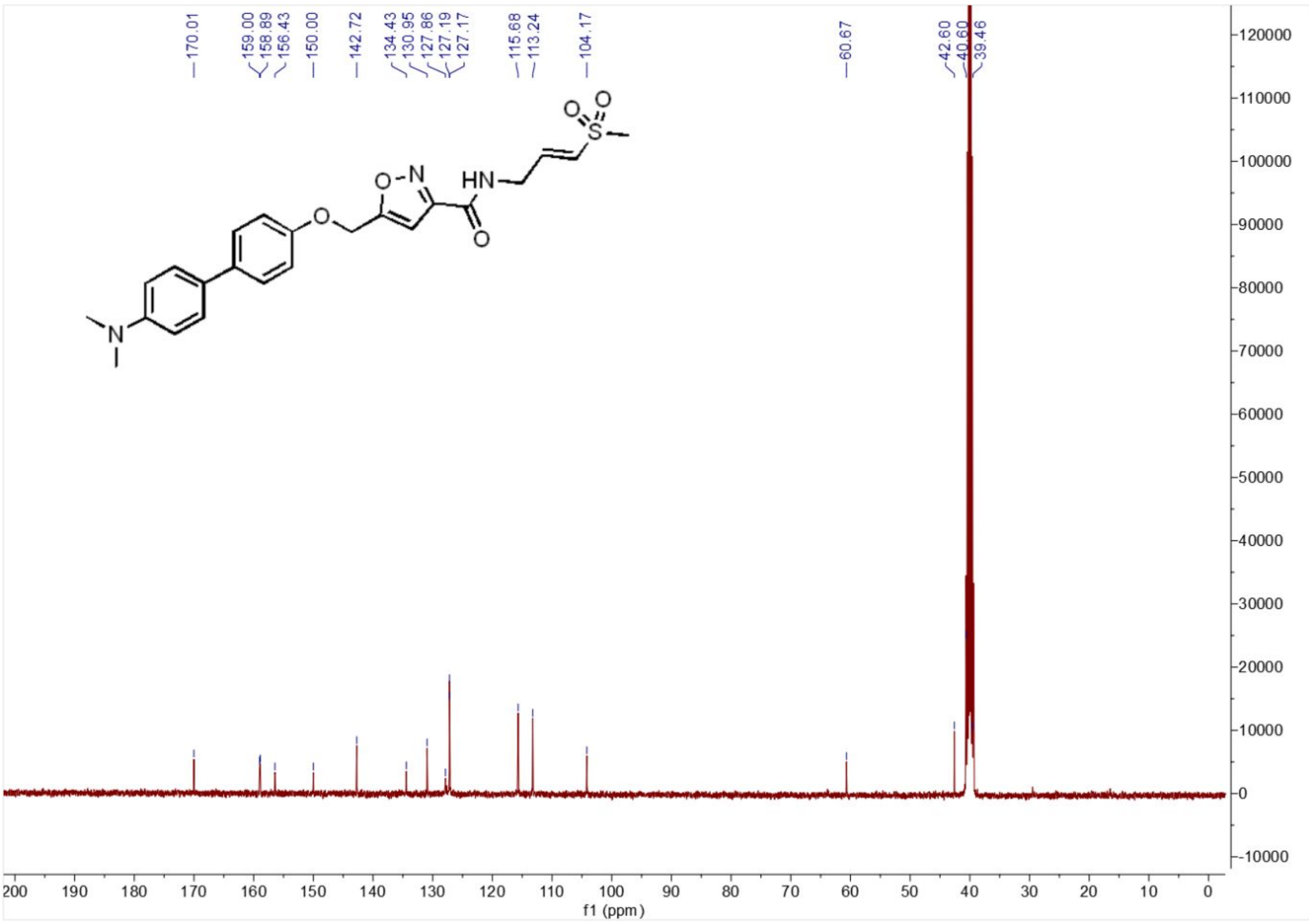

^{13}C NMR spectra of **Jun15176**

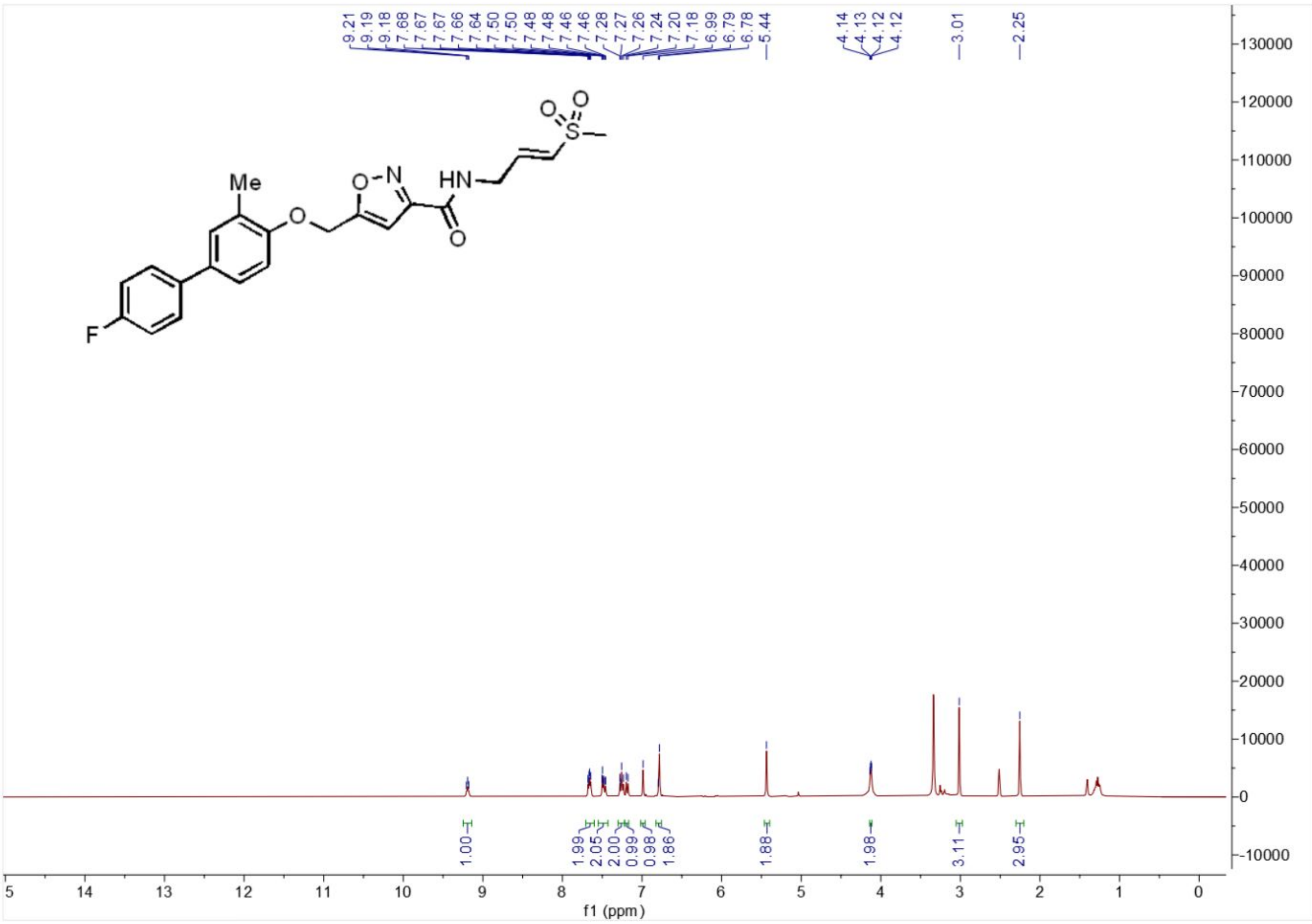

^{1}H NMR spectra of **Jun15112**

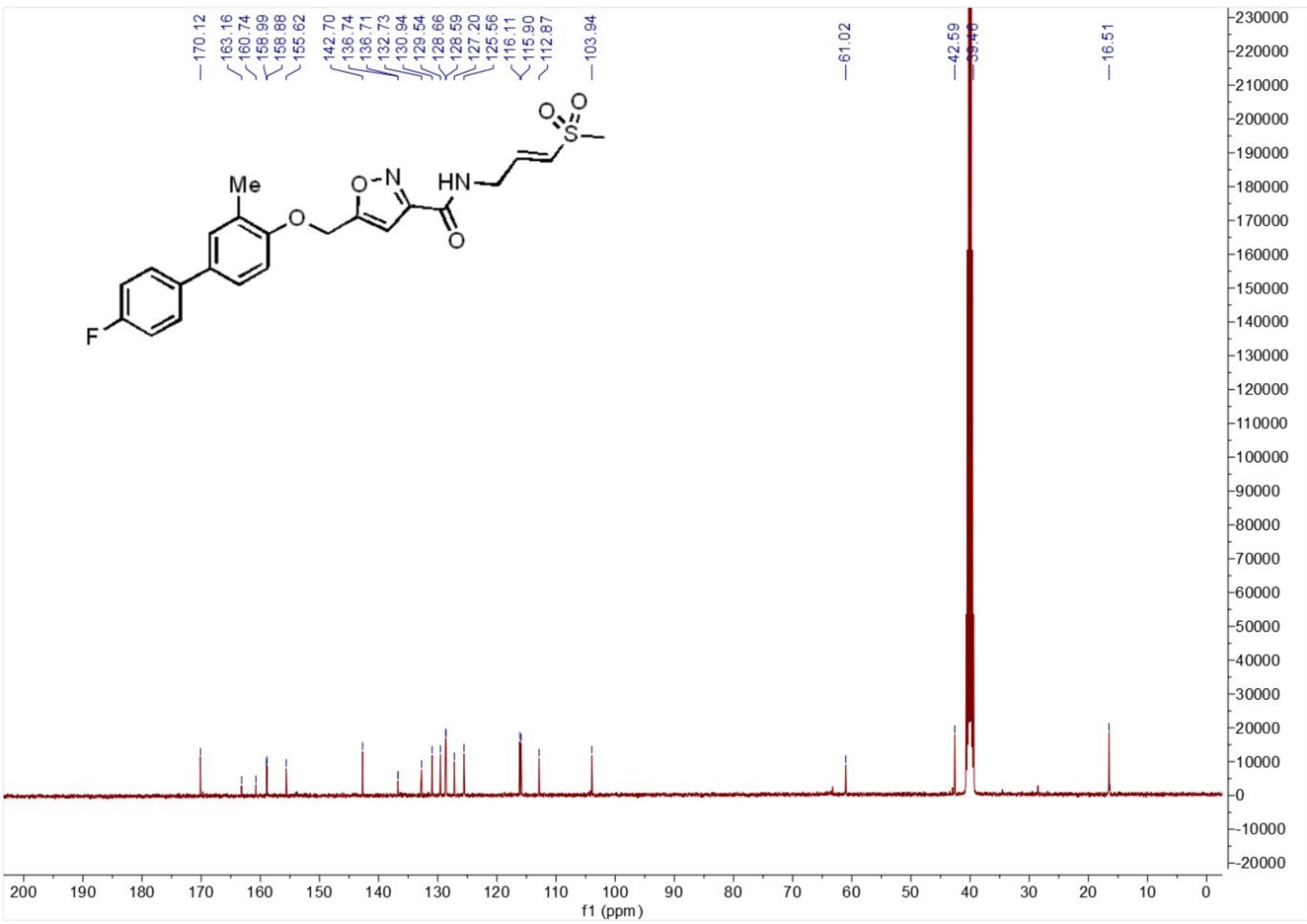

^{13}C NMR spectra of **Jun15112**

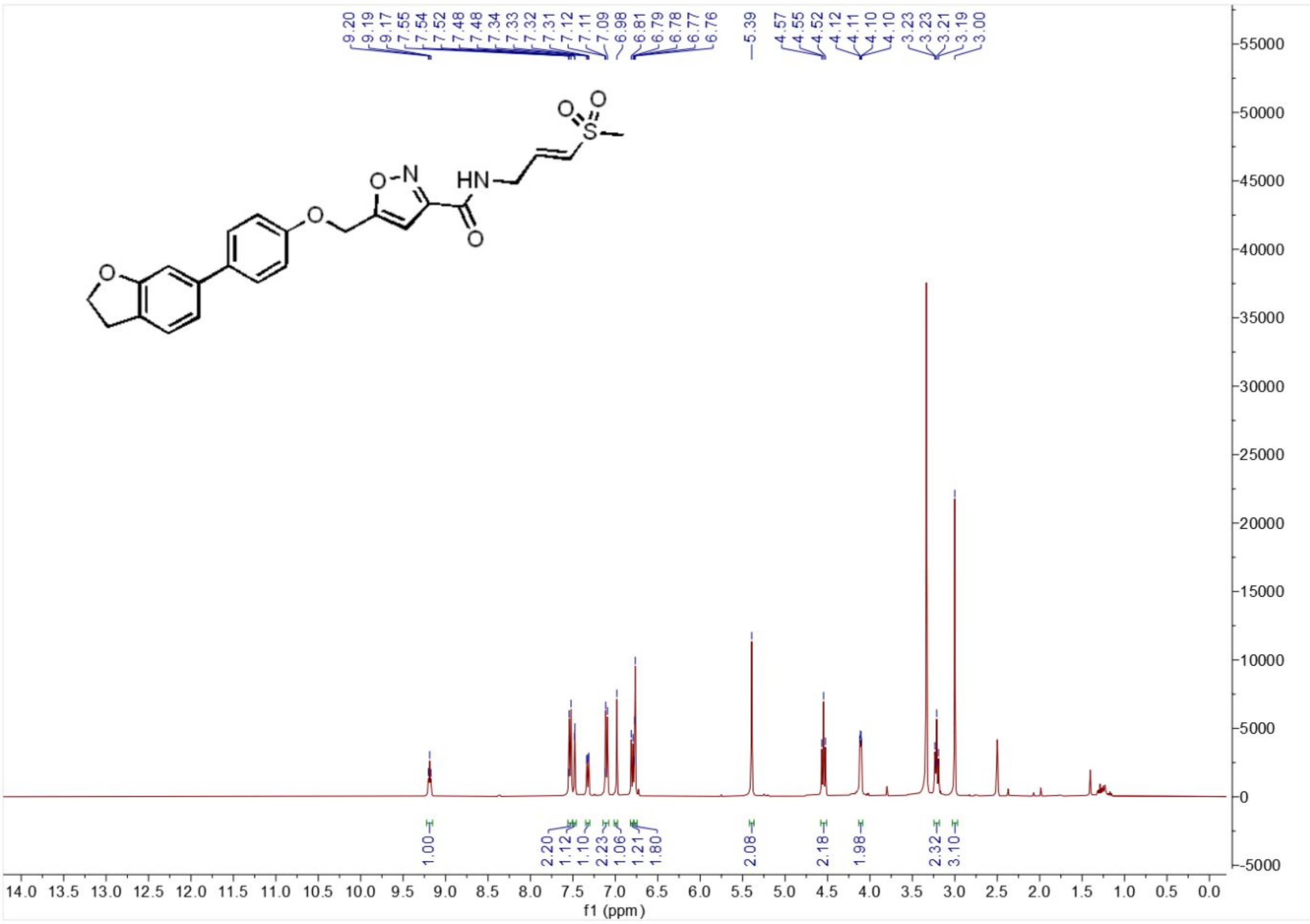

^{1}H NMR spectra of **Jun15172**

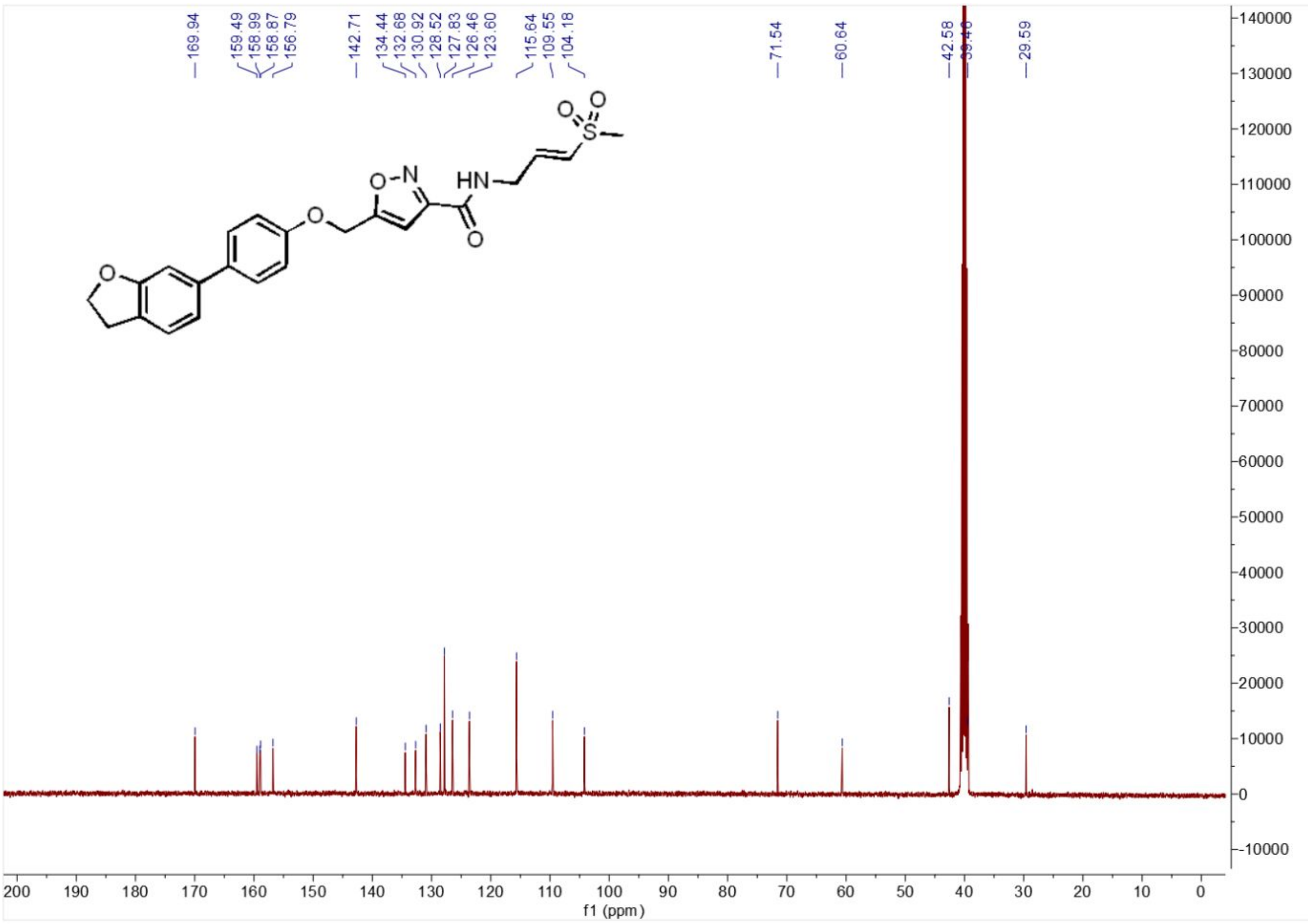

^{13}C NMR spectra of **Jun15172**

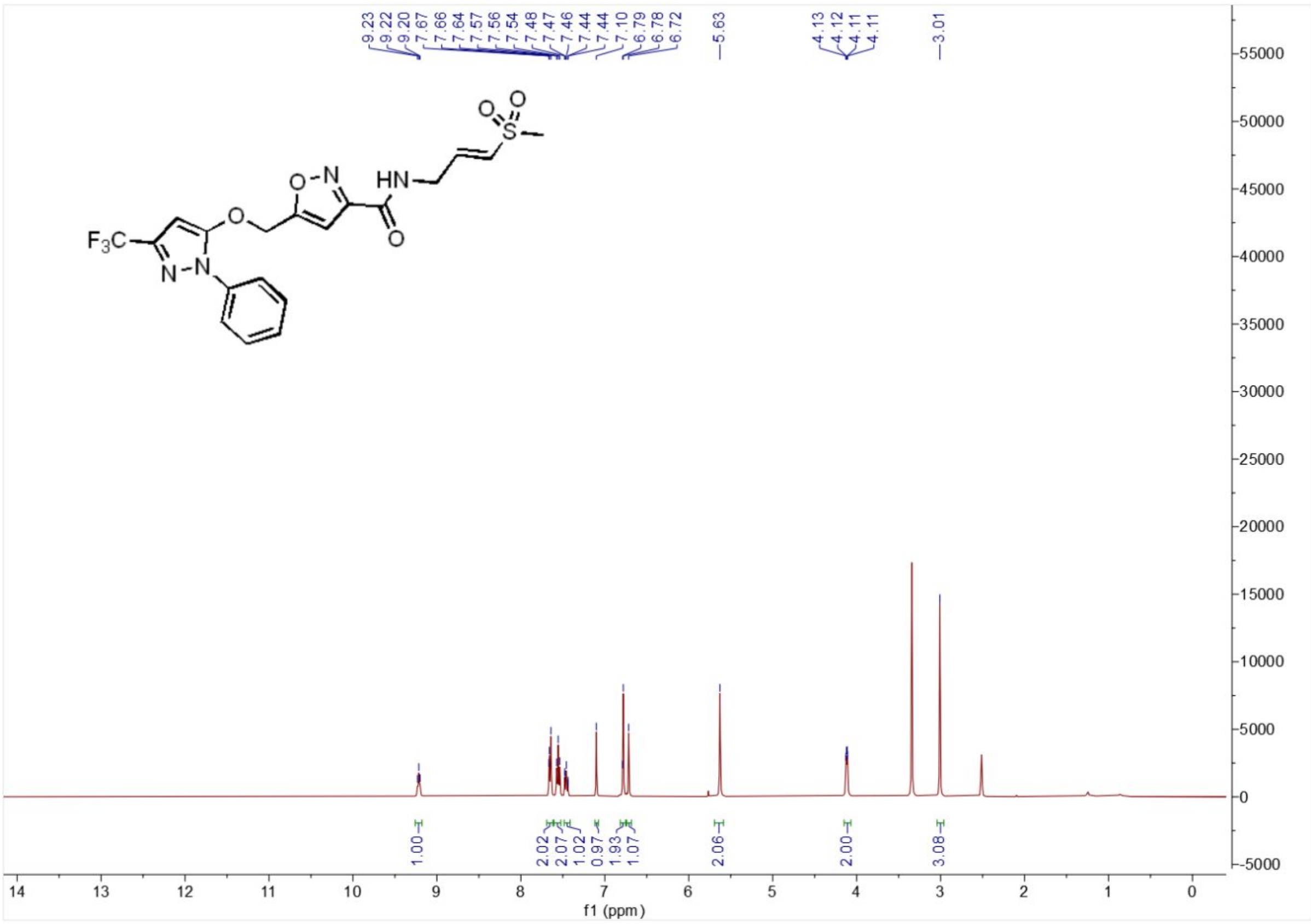

^{1}H NMR spectra of **Jun15175**

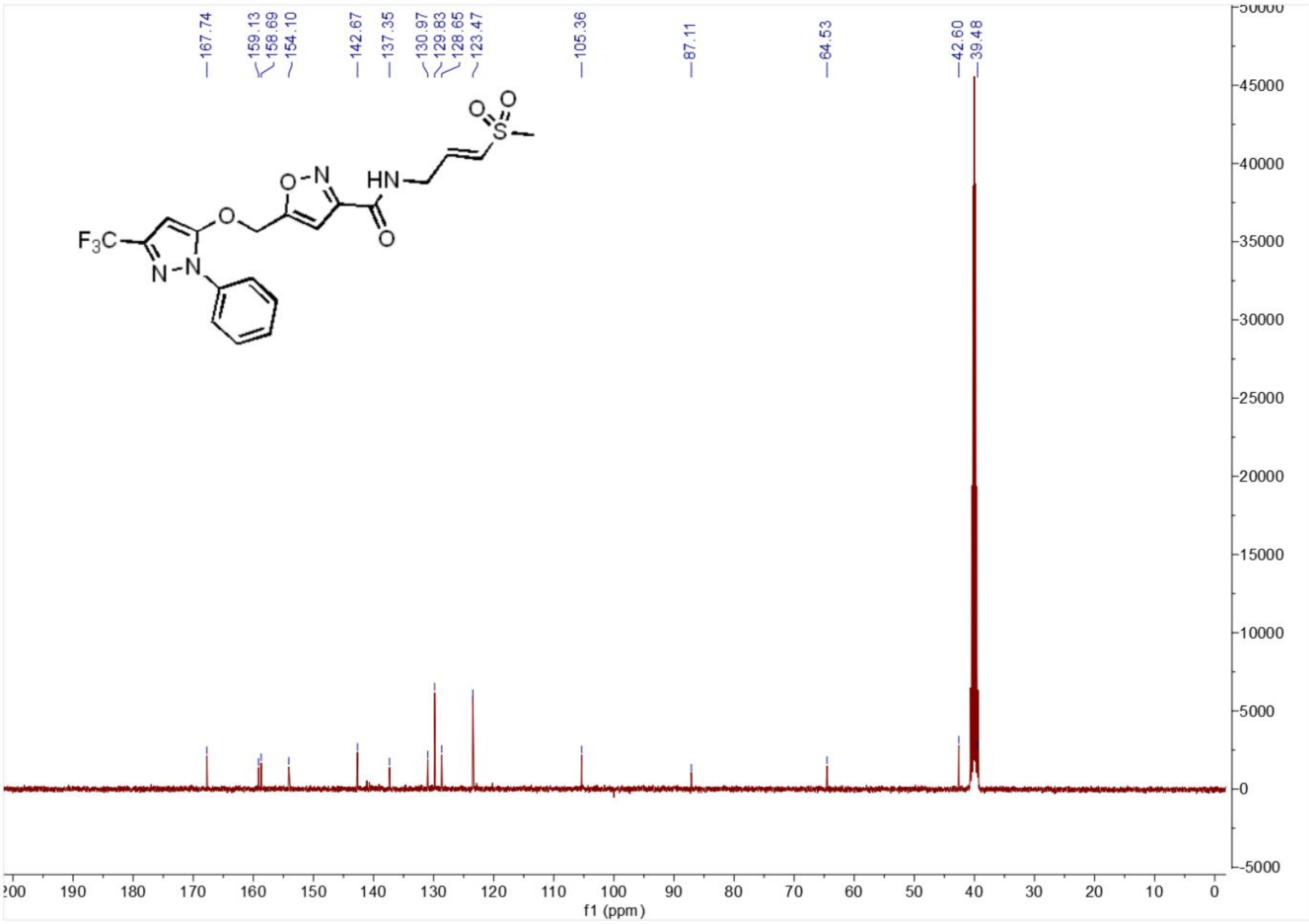

^{13}C NMR spectra of **Jun15175**

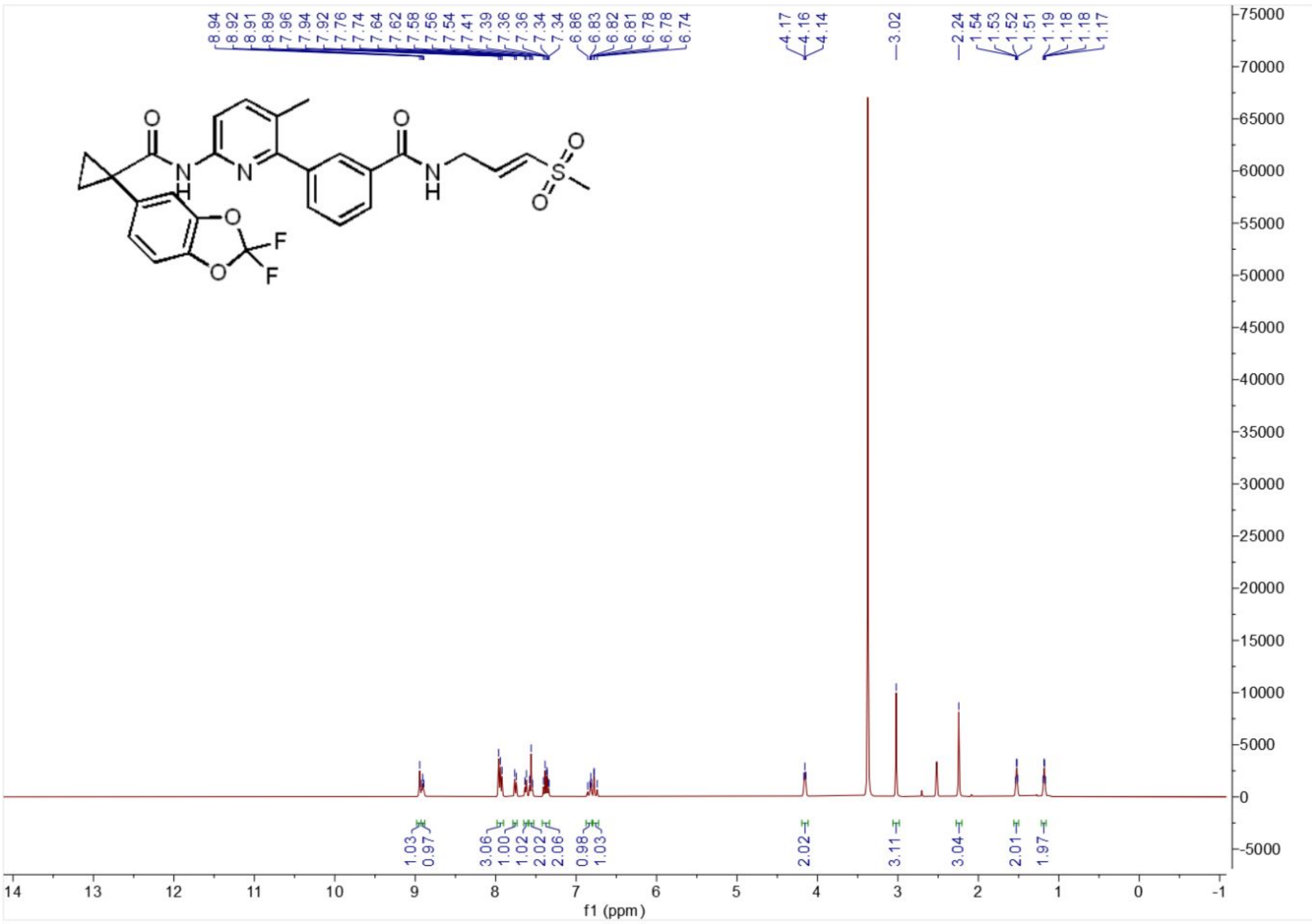

^{1}H NMR spectra of **Jun16225**

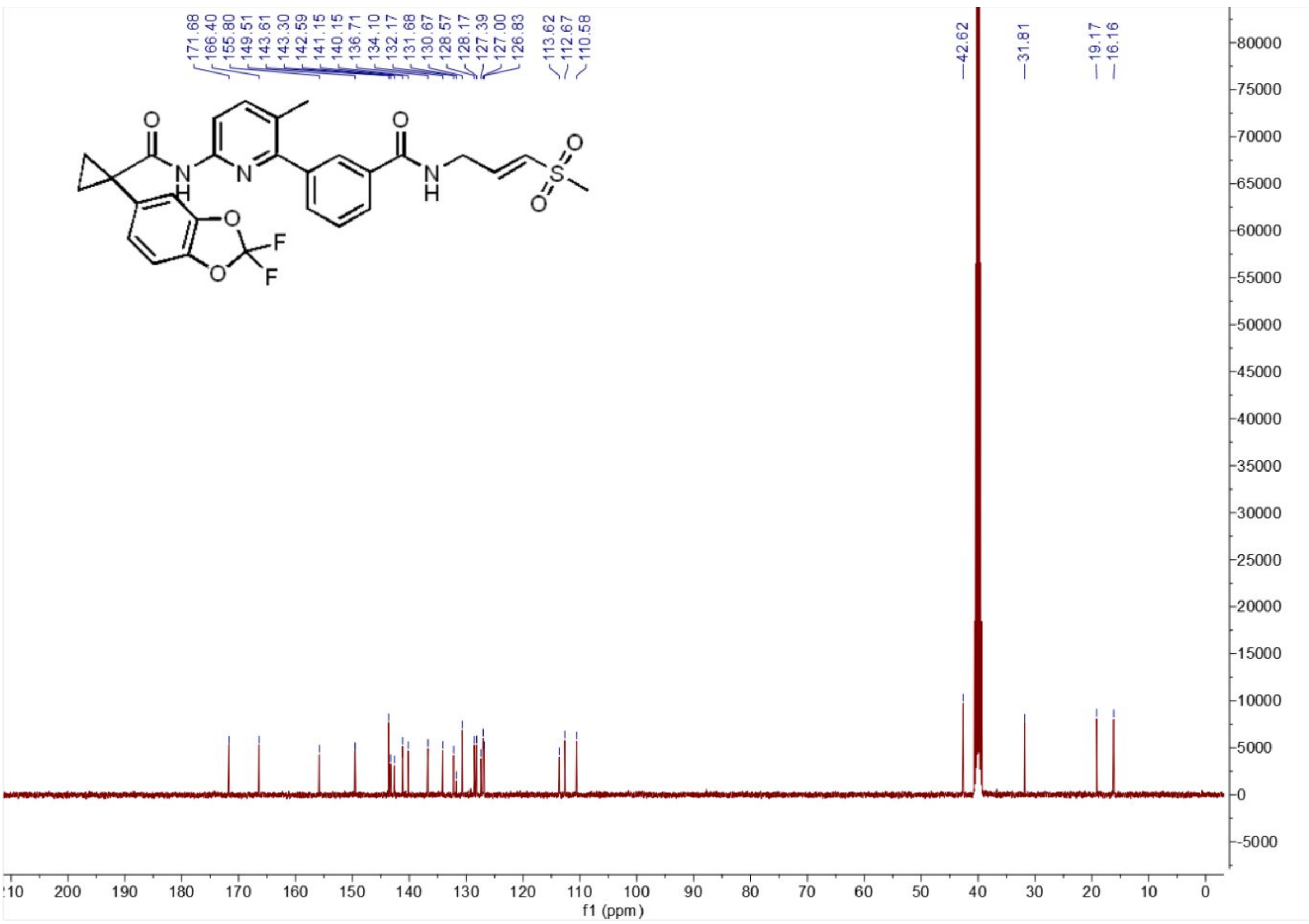

^{13}C NMR spectra of **Jun16225**

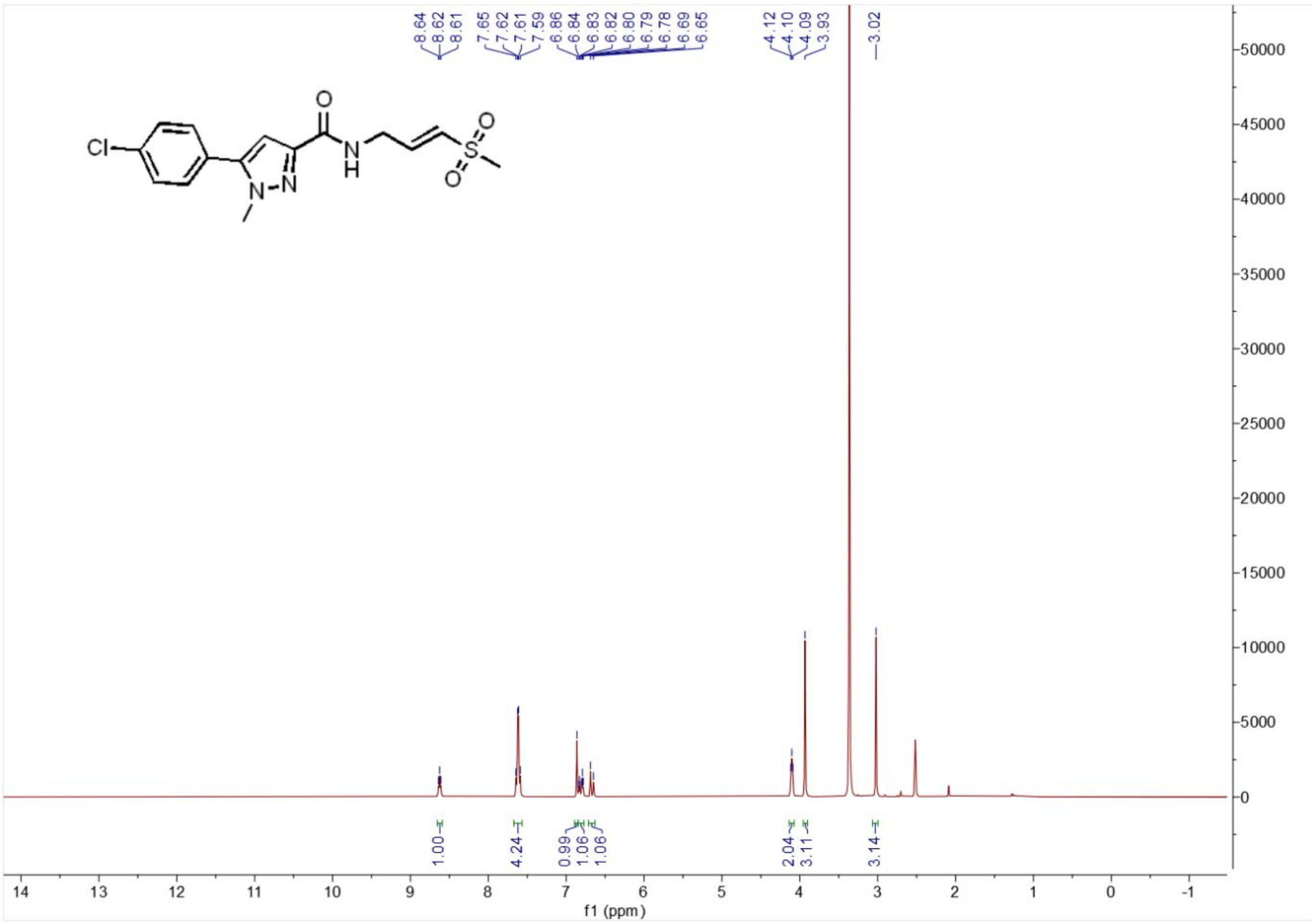

^{1}H NMR spectra of **Jun16226**

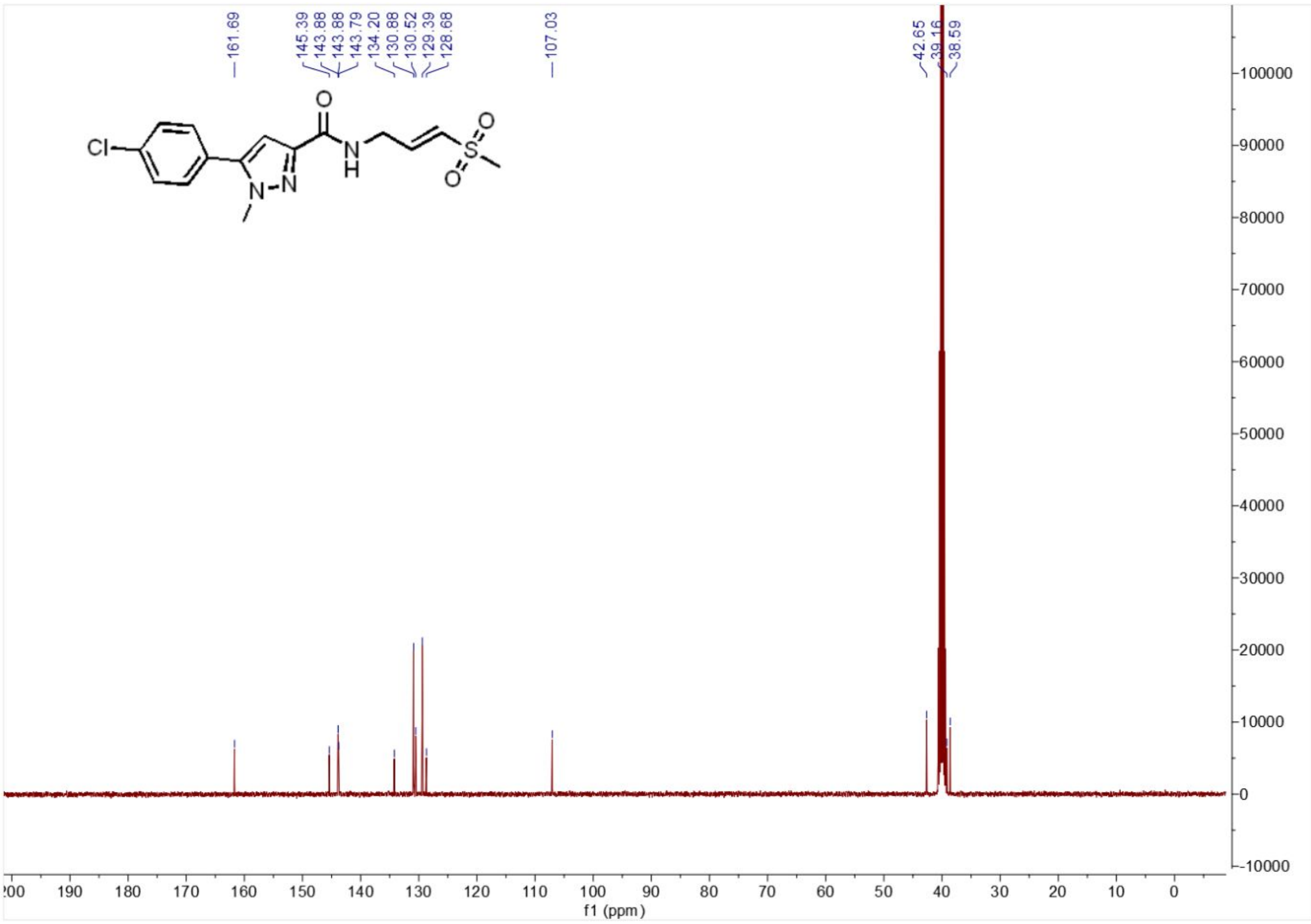

^{13}C NMR spectra of **Jun16226**

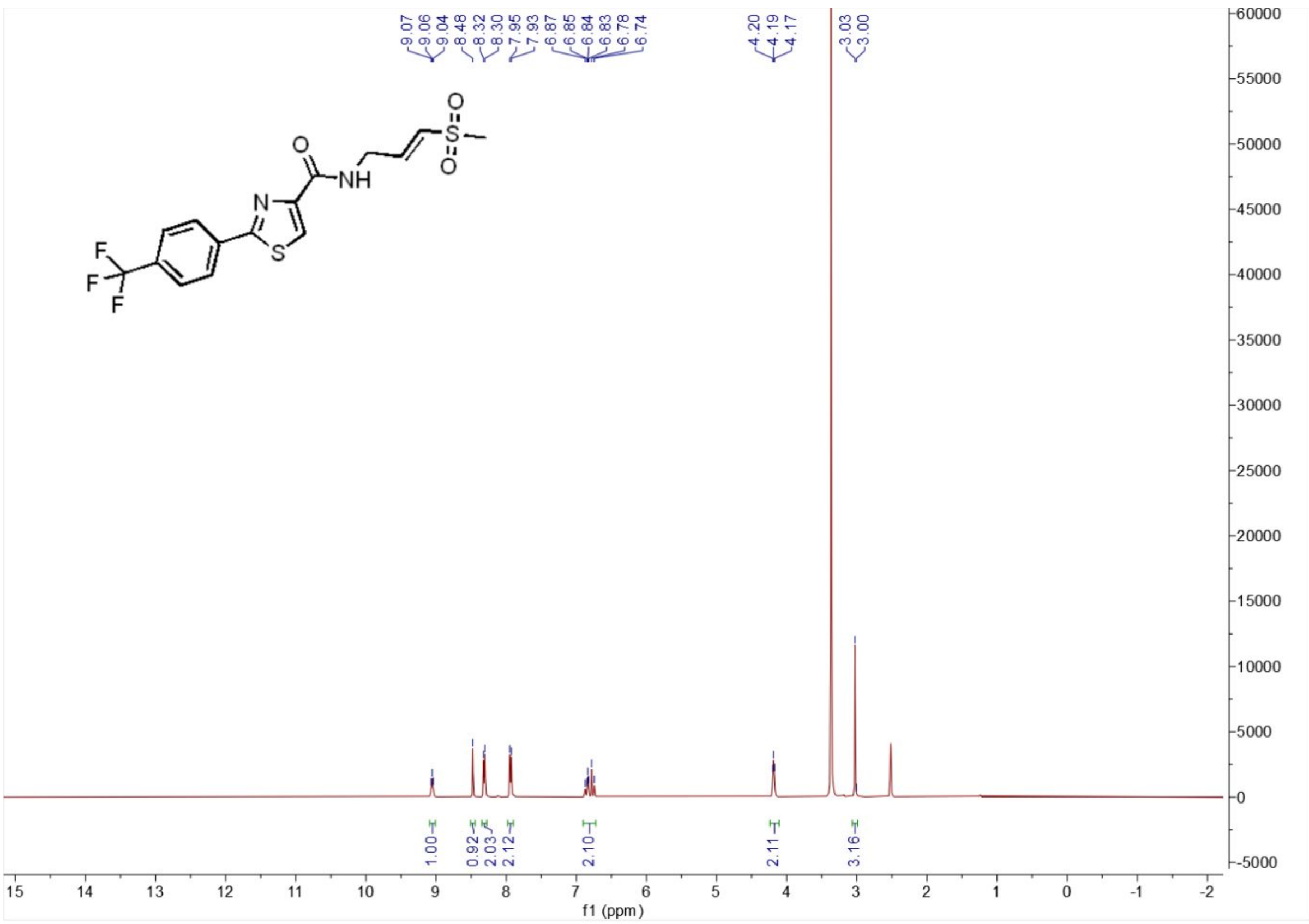

^{1}H NMR spectra of **Jun16227**

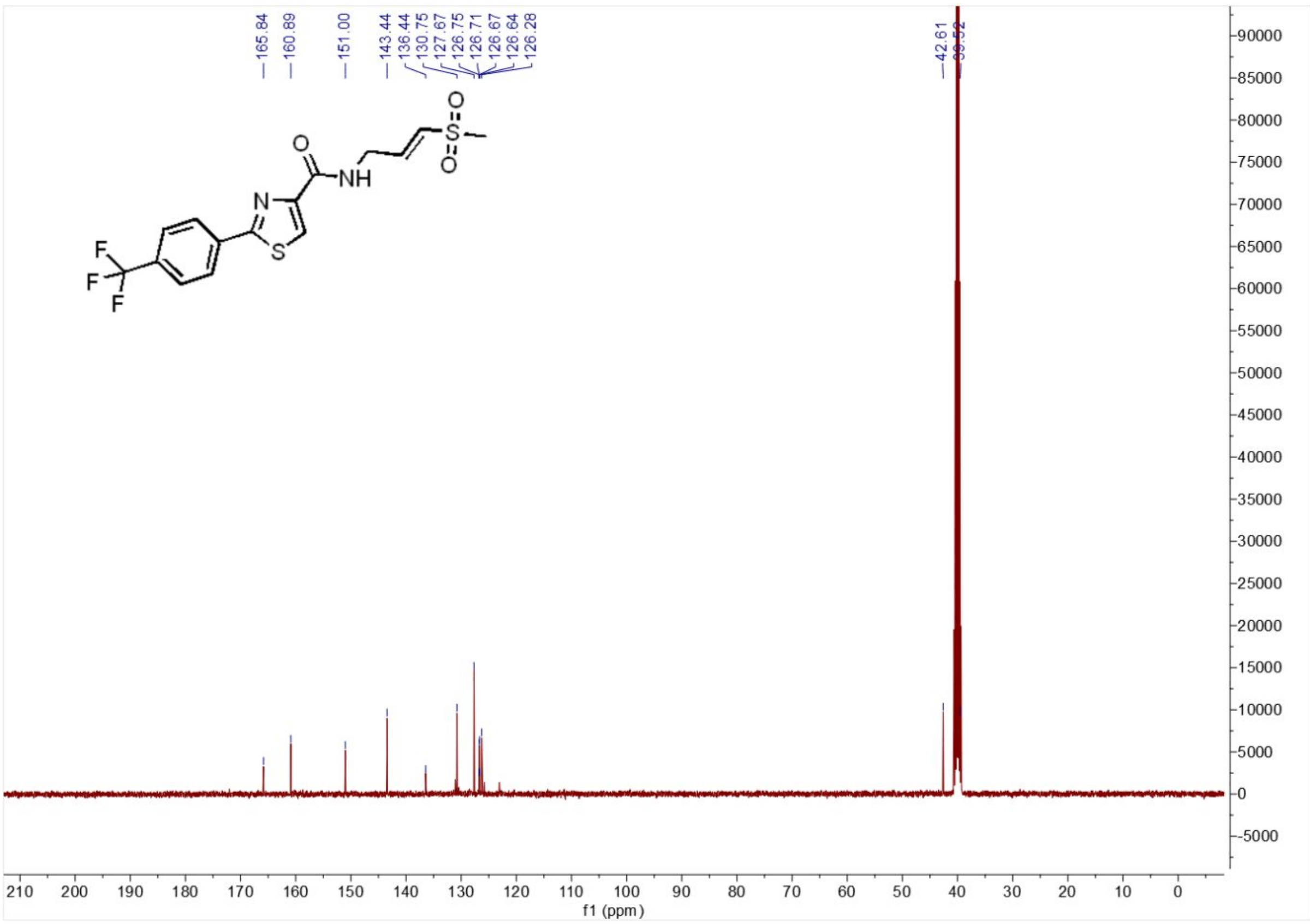

^{13}C NMR spectra of **Jun16227**

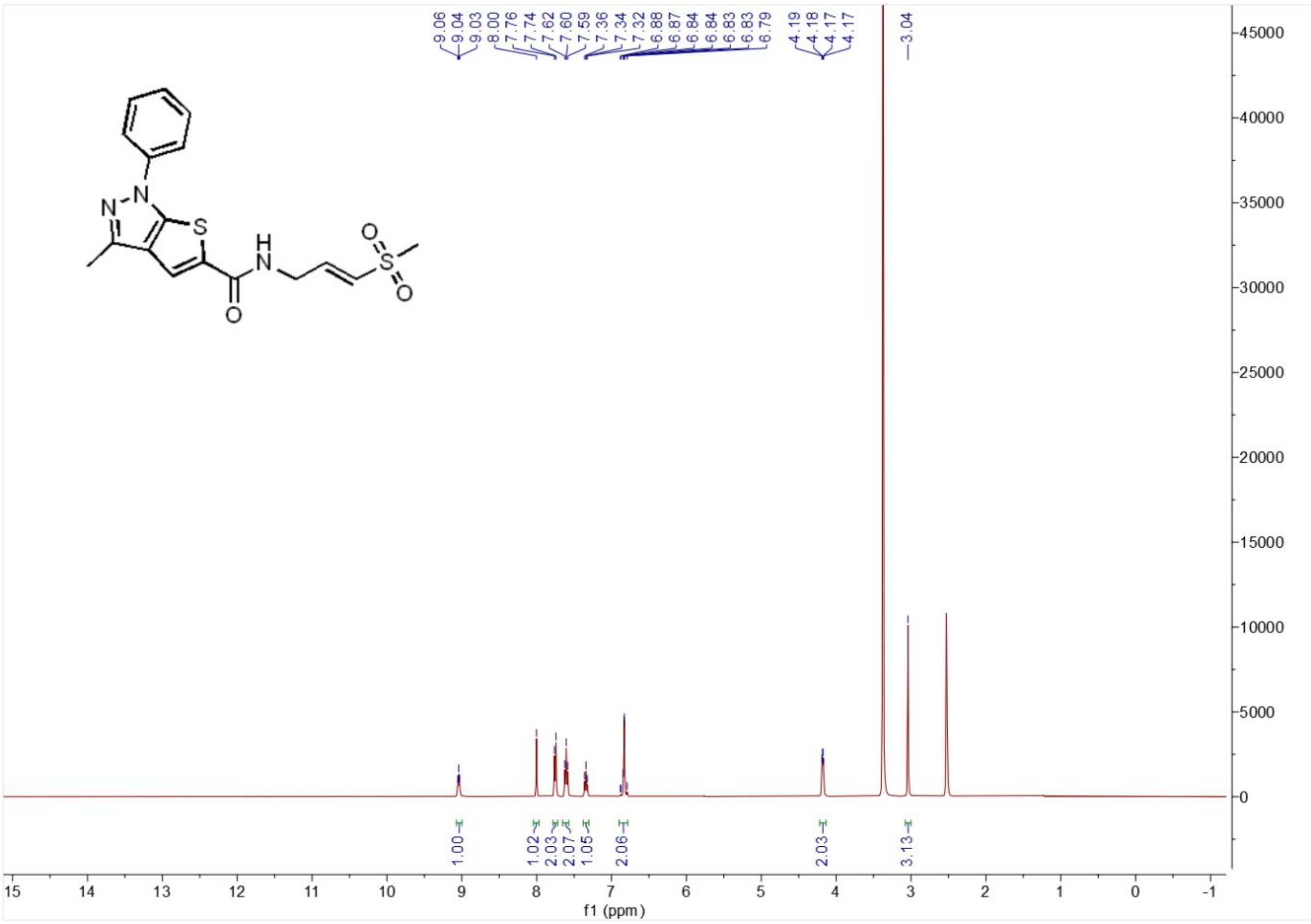

^1H NMR spectra of **Jun16228**

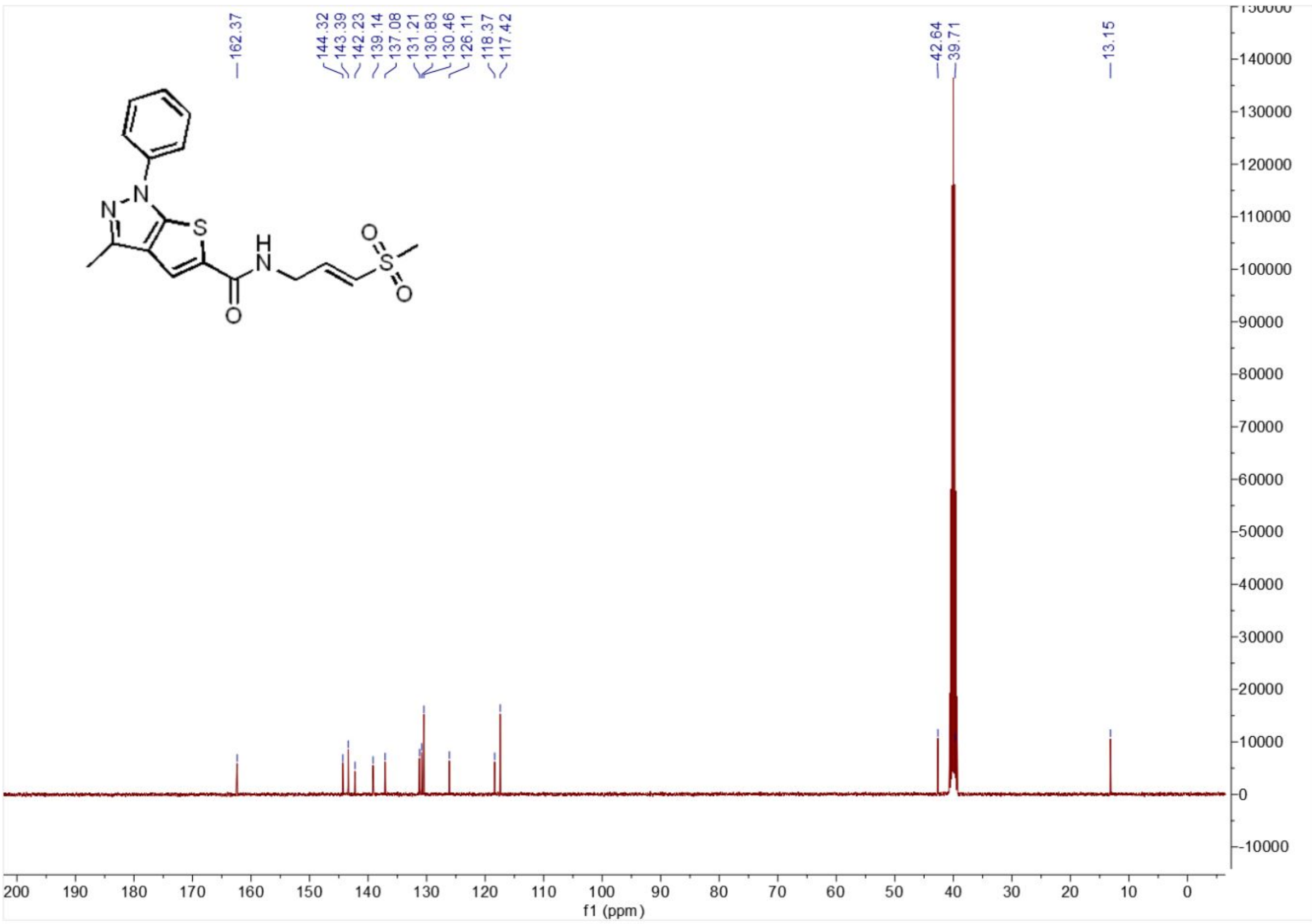

^{13}C NMR spectra of **Jun16228**

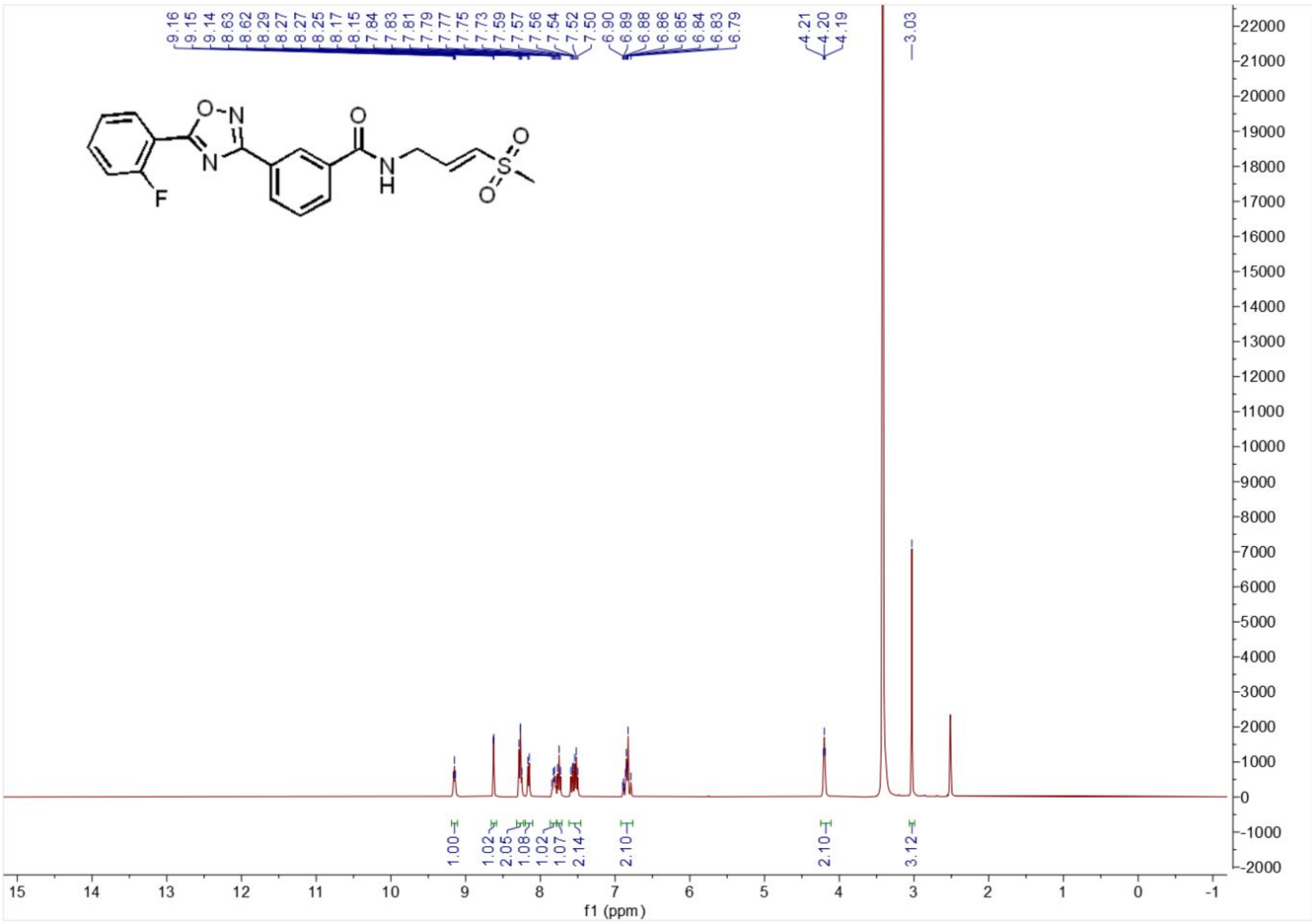

^{1}H NMR spectra of **Jun16229**

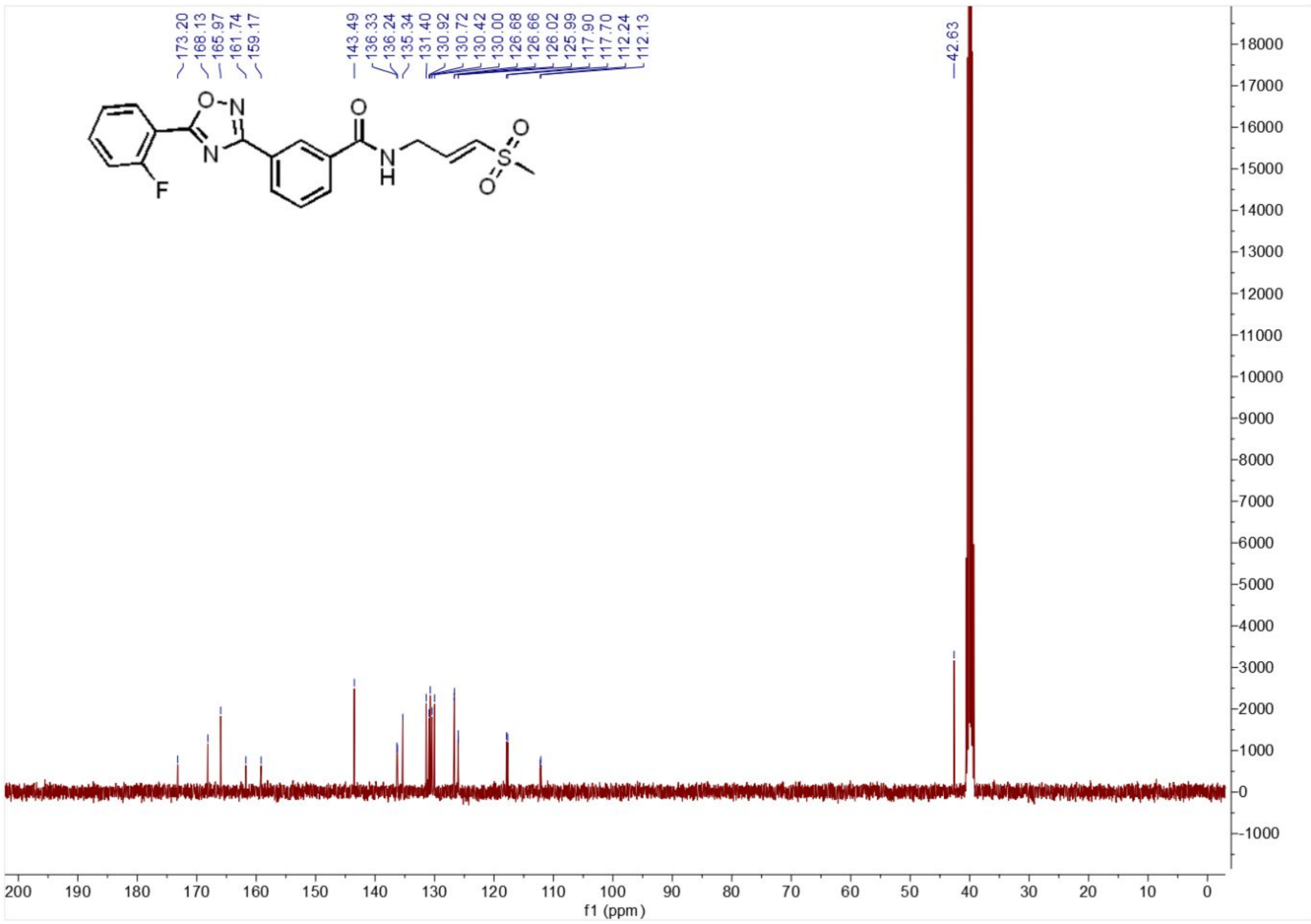

^{13}C NMR spectra of **Jun16229**

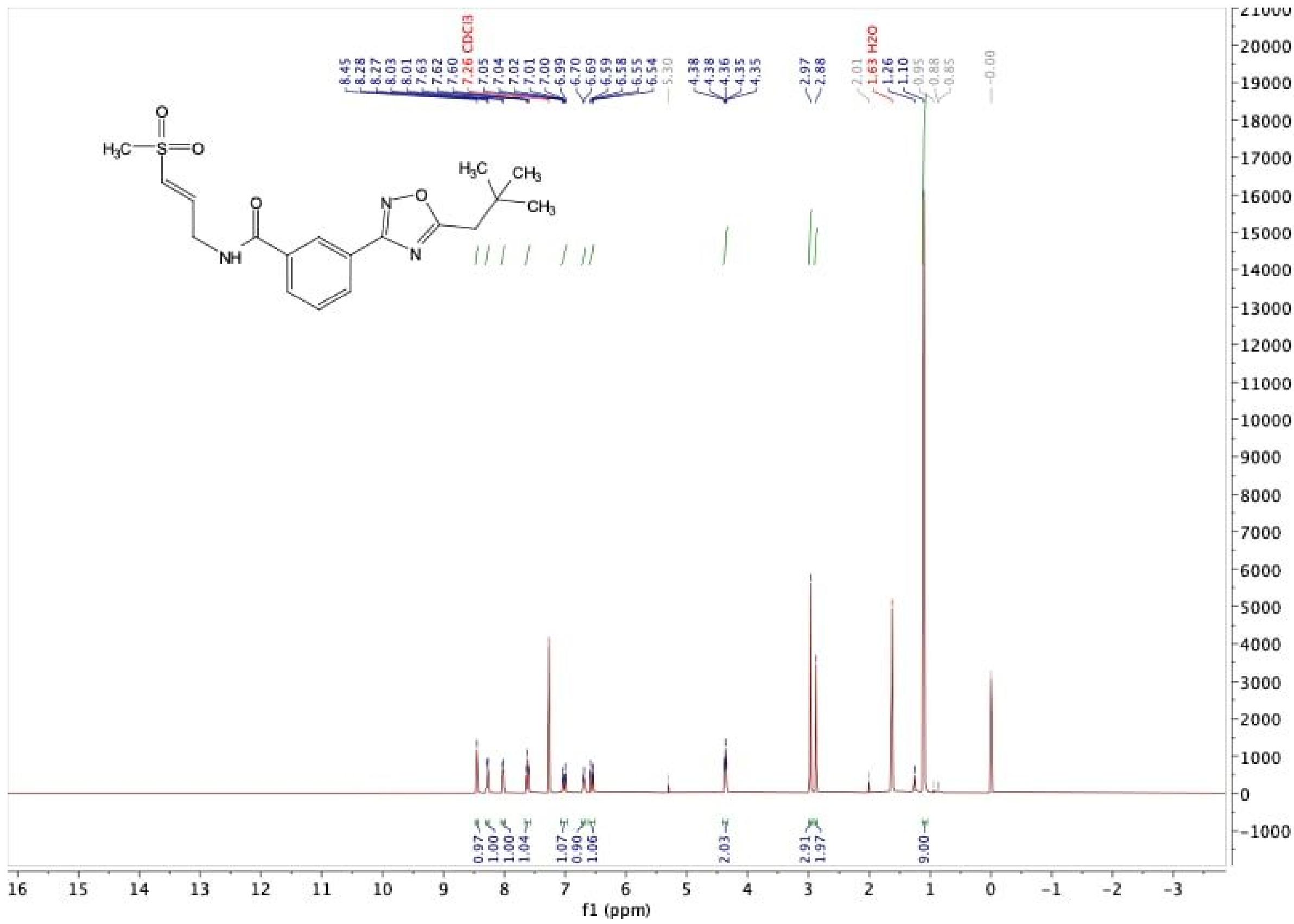

^{1}H NMR spectra of **Jun16387**

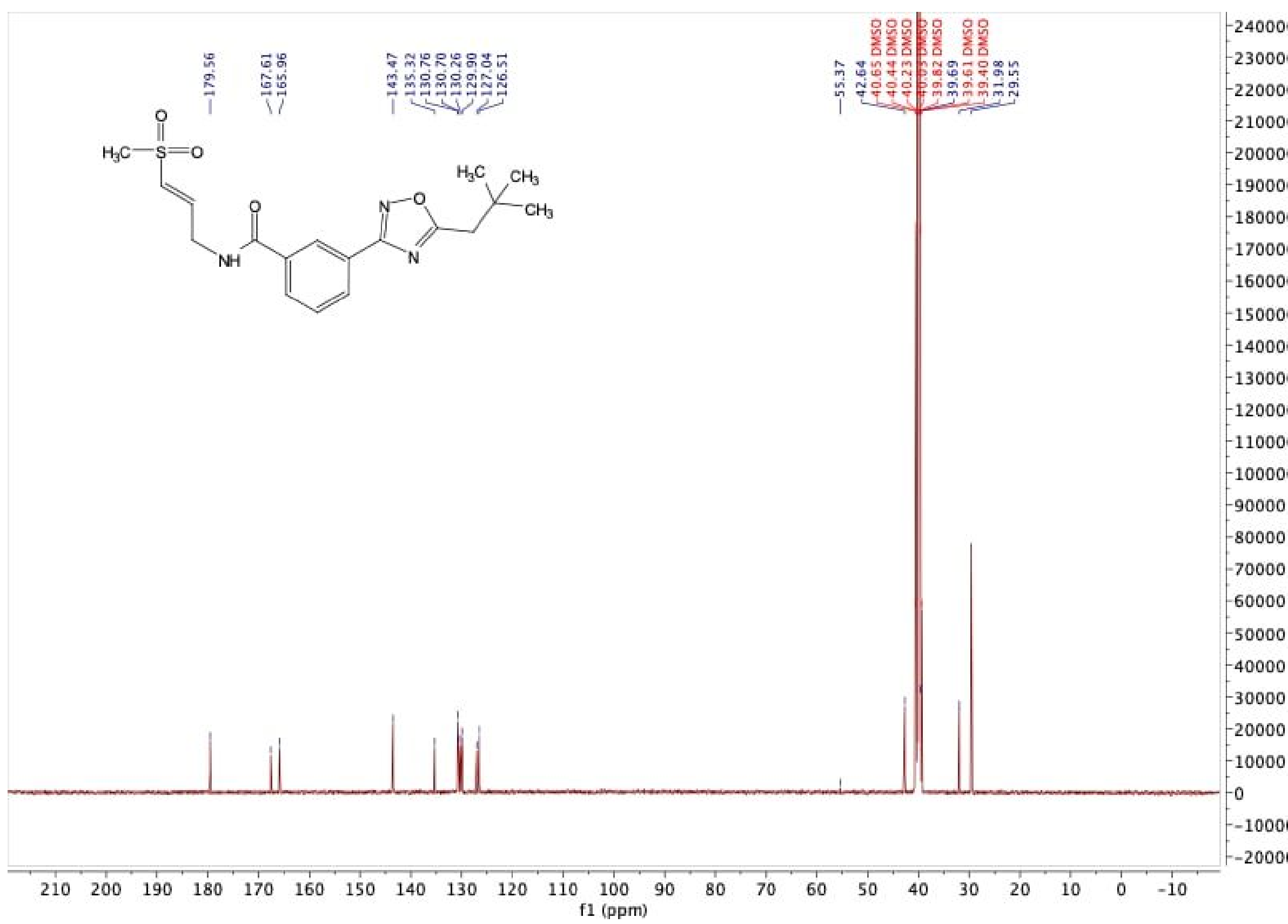

^{13}C NMR spectra of **Jun16387**

HPLC trace of Jun14502

Data File C:\CHEM32\1\DATA\BT_SEQN_ _DATE_ _TIME_ 82\031-0101.D
Sample Name: Jun14502

```
=====================================================================
Acq. Operator   : BT                            Seq. Line :   1
Acq. Instrument : Instrument 1                   Location : Vial 31
Injection Date  : 4/27/2025 3:17:35 AM                Inj :   1
                                               Inj Volume : 4 µl
Acq. Method     : C:\Chem32\1\DATA\BT\_SeqN_ _Date_ _Time_ 82\COLUMNWASHING.M
Last changed    : 4/27/2025 3:14:32 AM by BT
                  (modified after loading)
Analysis Method : C:\CHEM32\1\DATA\BT\_SEQN_ _DATE_ _TIME_ 82\031-0101.D\DA.M (COLUMNWASHING.M)
Last changed    : 5/12/2025 6:52:28 AM by BT
```

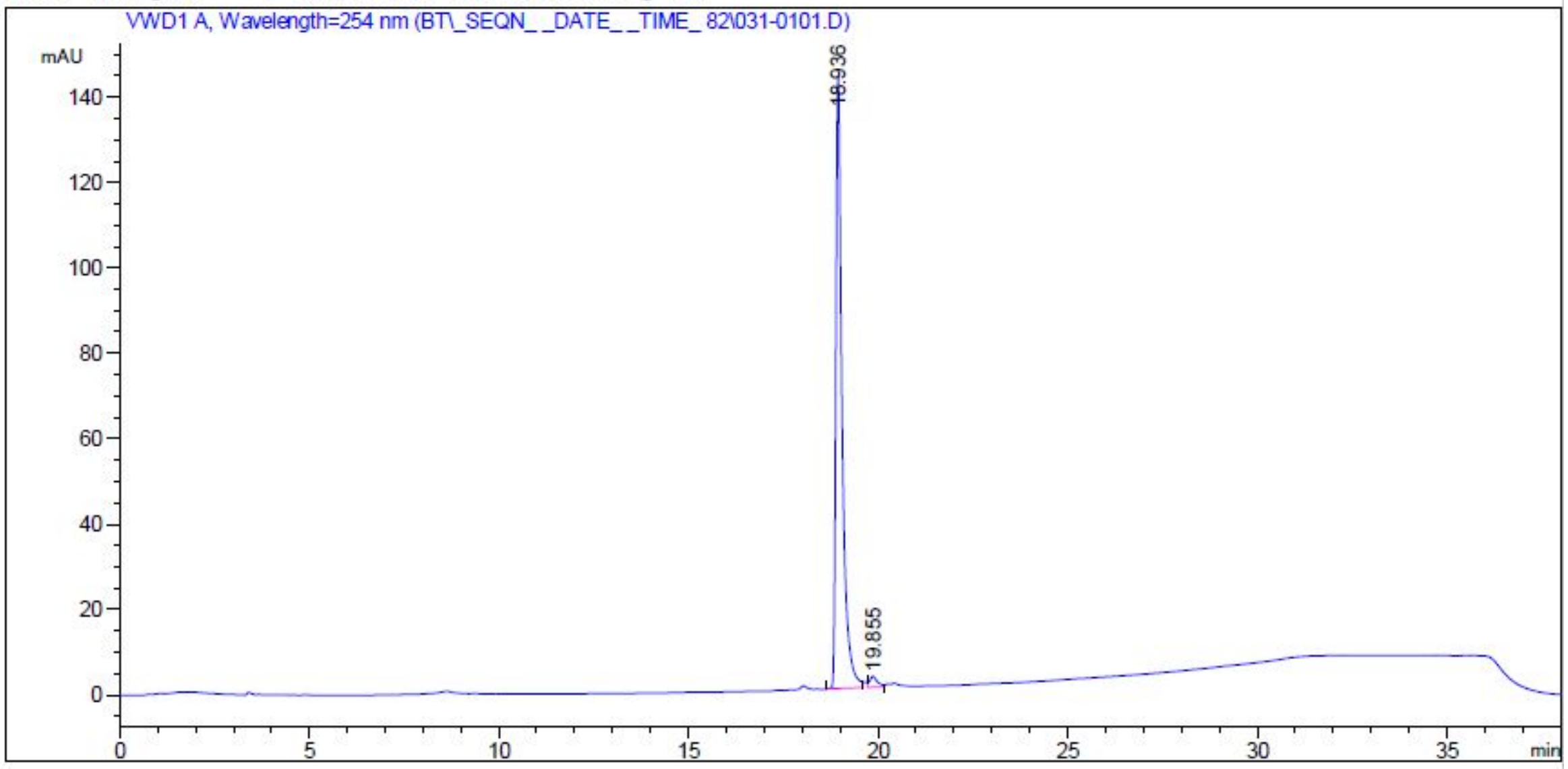


```
=====================================================================
                        Area Percent Report
=====================================================================

Sorted By             :      Signal
Multiplier            :      1.0000
Dilution              :      1.0000
Use Multiplier & Dilution Factor with ISTDs


Signal 1: VWD1 A, Wavelength=254 nm

Peak RetTime Type  Width     Area      Height     Area
  #   [min]        [min]  mAU   *s   [mAU   ]      %
----|-------|----|-------|----------|----------|--------|
   1  18.936 BB    0.1650 1637.95691  143.68178  97.7232
   2  19.855 BV    0.2071   38.16180    2.63114   2.2768

Totals :                  1676.11871  146.31292
```

HPLC trace of Jun14528

```
Data File C:\CHEM32\1\DATA\BT\_SEQN_ _DATE_ _TIME_ 82\032-0201.D
Sample Name: Jun14528

    =====================================================================
    Acq. Operator   : BT                            Seq. Line :   2
    Acq. Instrument : Instrument 1                   Location : Vial 32
    Injection Date  : 4/27/2025 3:56:19 AM                Inj :   1
                                                   Inj Volume : 4 µl
    Acq. Method     : C:\Chem32\1\DATA\BT\_SeqN_ _Date_ _Time_ 82\COLUMNWASHING.M
    Last changed    : 4/27/2025 3:55:33 AM by BT
                      (modified after loading)
    Analysis Method : C:\CHEM32\1\DATA\BT\_SEQN_ _DATE_ _TIME_ 82\032-0201.D\DA.M (COLUMNWASHING.M)
    Last changed    : 5/12/2025 6:54:18 AM by BT
```

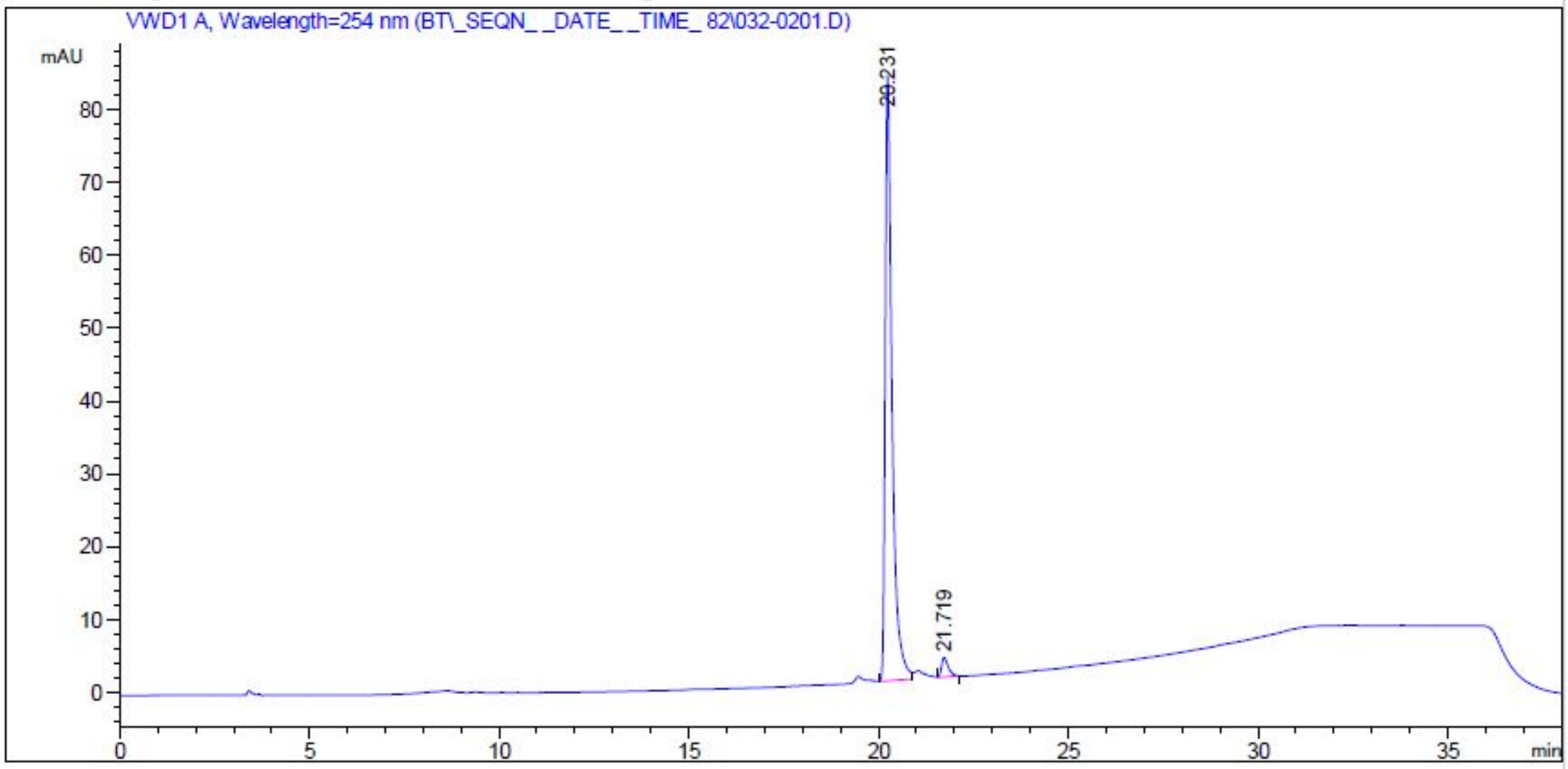


```
    =====================================================================
                          Area Percent Report
    =====================================================================

    Sorted By             :      Signal
    Multiplier            :      1.0000
    Dilution              :      1.0000
    Use Multiplier & Dilution Factor with ISTDs


    Signal 1: VWD1 A, Wavelength=254 nm

    Peak RetTime Type  Width     Area      Height     Area
      #   [min]        [min]  mAU   *s    [mAU   ]      %
    ----|-------|----|-------|----------|----------|--------|
       1  20.231 BB    0.1829 1044.33643   83.14513  96.7959
       2  21.719 BB    0.1864   34.56888    2.71478   3.2041

    Totals :                  1078.90530   85.85991
```

HPLC trace of Jun14529

```
Data File C:\CHEM32\1\DATA\BT\_SEQN_ _DATE_ _TIME_ 82\033-0301.D
Sample Name: Jun14529

    =====================================================================
    Acq. Operator   : BT                            Seq. Line :   3
    Acq. Instrument : Instrument 1                   Location : Vial 33
    Injection Date  : 4/27/2025 4:35:26 AM                Inj :   1
                                                   Inj Volume : 4 µl
    Acq. Method     : C:\Chem32\1\DATA\BT\_SeqN_ _Date_ _Time_ 82\COLUMNWASHING.M
    Last changed    : 4/27/2025 4:34:36 AM by BT
                      (modified after loading)
    Analysis Method : C:\CHEM32\1\DATA\BT\_SEQN_ _DATE_ _TIME_ 82\033-0301.D\DA.M (COLUMNWASHING.M)
    Last changed    : 5/12/2025 6:55:22 AM by BT
```

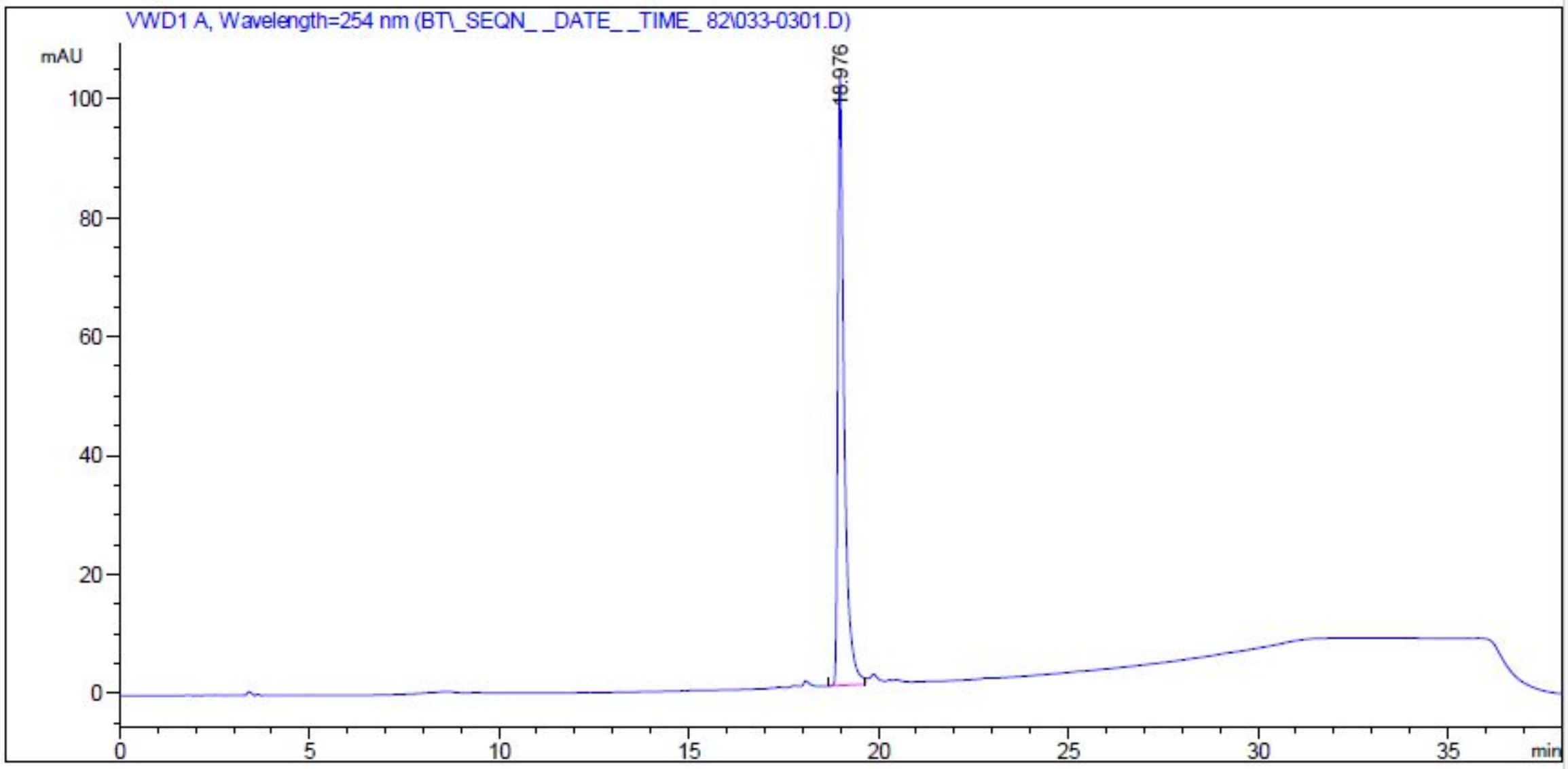


```
    =====================================================================
                            Area Percent Report
    =====================================================================

    Sorted By            :      Signal
    Multiplier           :      1.0000
    Dilution             :      1.0000
    Use Multiplier & Dilution Factor with ISTDs


    Signal 1: VWD1 A, Wavelength=254 nm

    Peak RetTime Type  Width     Area      Height     Area
      #   [min]        [min]  mAU   *s   [mAU   ]       %
    ----|-------|----|-------|----------|----------|--------|
       1  18.976 BB    0.1682 1198.22083  102.64517 100.0000

    Totals :                  1198.22083  102.64517
```

HPLC trace of Jun14693

Data File C:\CHEM32\1\DATA\BT_SEQN_ _DATE_ _TIME_ 82\035-0501.D
Sample Name: Jun14693

```
=====================================================================
Acq. Operator   : BT                              Seq. Line :   5
Acq. Instrument : Instrument 1                     Location : Vial 35
Injection Date  : 4/27/2025 5:53:40 AM                  Inj :   1
                                                 Inj Volume : 4 µl
Acq. Method     : C:\Chem32\1\DATA\BT\_SeqN_ _Date_ _Time_ 82\COLUMNWASHING.M
Last changed    : 4/27/2025 5:52:53 AM by BT
                  (modified after loading)
Analysis Method : C:\CHEM32\1\DATA\BT\_SEQN_ _DATE_ _TIME_ 82\035-0501.D\DA.M (COLUMNWASHING.M)
Last changed    : 5/12/2025 6:56:28 AM by BT
```

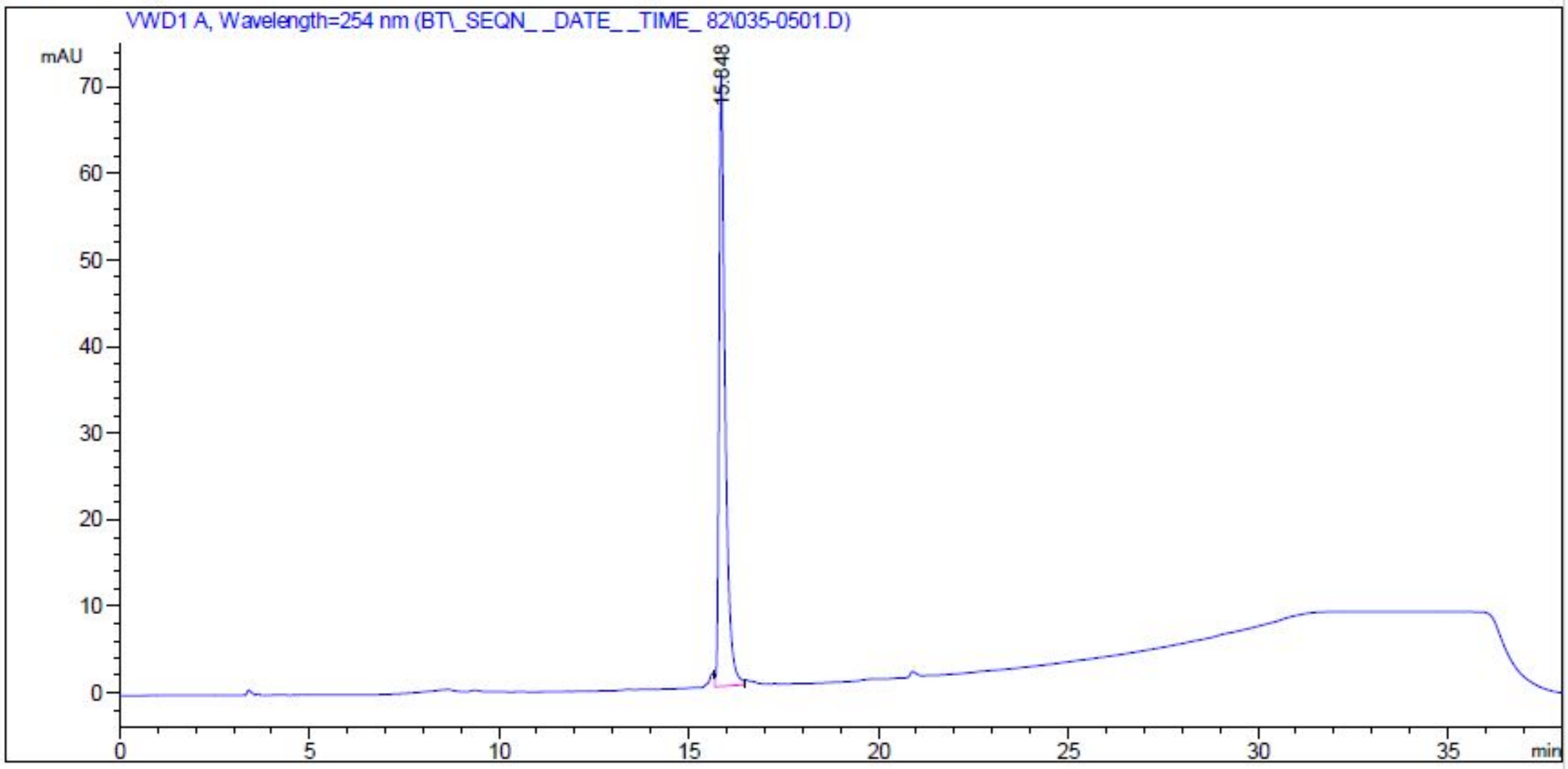


```
=====================================================================
                         Area Percent Report
=====================================================================

Sorted By             :      Signal
Multiplier            :      1.0000
Dilution              :      1.0000
Use Multiplier & Dilution Factor with ISTDs


Signal 1: VWD1 A, Wavelength=254 nm
```

Peak #	RetTime [min]	Type	Width [min]	Area mAU *s	Height [mAU]	Area %
1	15.848	VB	0.1629	792.52869	70.65610	100.0000

Totals : 792.52869 70.65610

HPLC trace of Jun14969

```
Data File C:\CHEM32\1\DATA\BT\_SEQN_ _DATE_ _TIME_ 84\050-1201.D
Sample Name: Jun14969

    =====================================================================
    Acq. Operator   : BT                          Seq. Line :  12
    Acq. Instrument : Instrument 1                 Location : Vial 50
    Injection Date  : 4/28/2025 5:12:45 PM              Inj :   1
                                                Inj Volume : 4 µl
    Acq. Method     : C:\Chem32\1\DATA\BT\_SeqN_ _Date_ _Time_ 84\COLUMNWASHING.M
    Last changed    : 4/28/2025 8:27:18 AM by BT
    Analysis Method : C:\CHEM32\1\DATA\BT\_SEQN_ _DATE_ _TIME_ 84\050-1201.D\DA.M (COLUMNWASHING.M)
    Last changed    : 5/12/2025 7:13:46 AM by BT
```

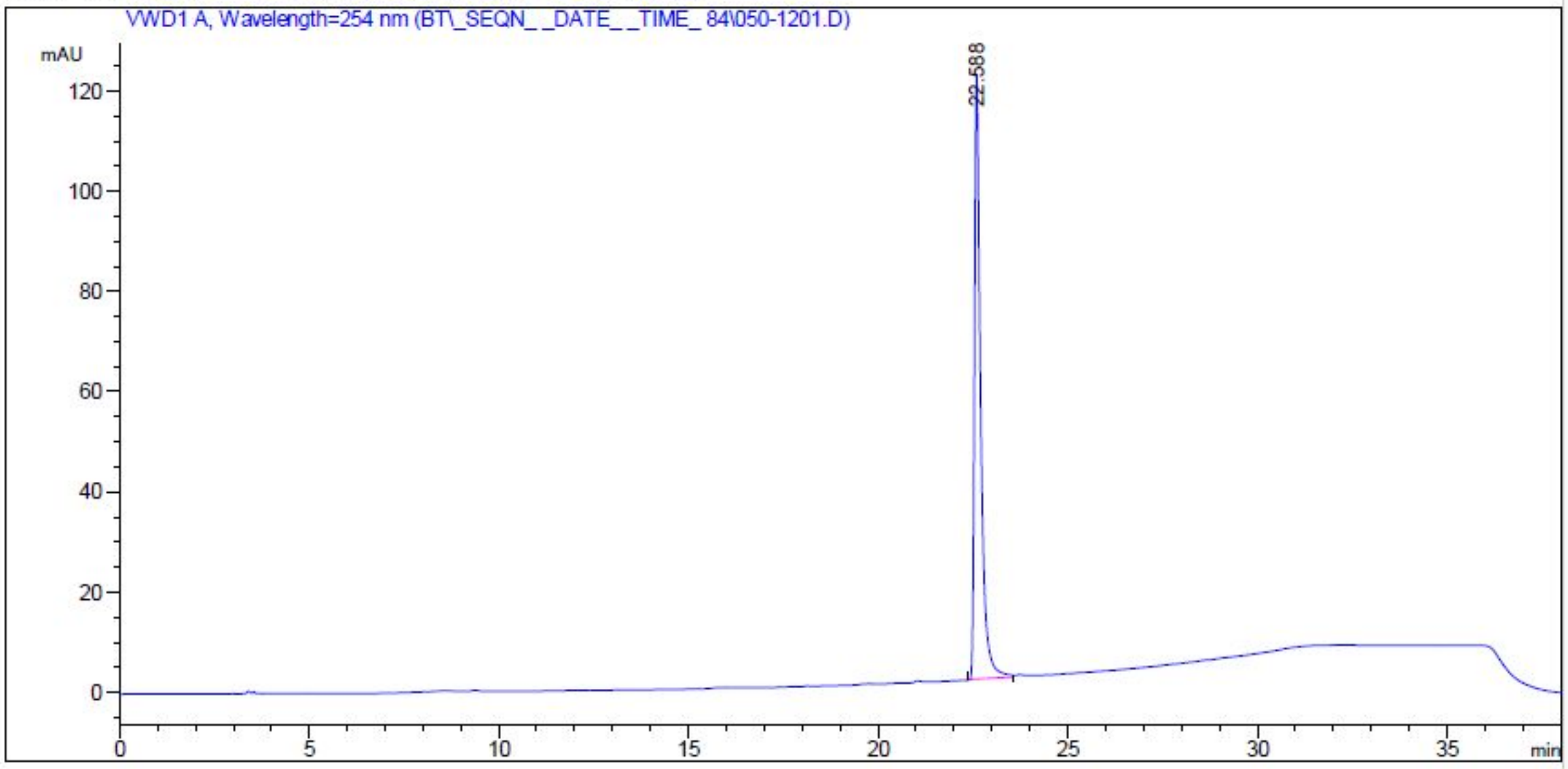


```
    =====================================================================
                         Area Percent Report
    =====================================================================

    Sorted By             :      Signal
    Multiplier            :      1.0000
    Dilution              :      1.0000
    Use Multiplier & Dilution Factor with ISTDs


    Signal 1: VWD1 A, Wavelength=254 nm

    Peak RetTime Type  Width     Area      Height     Area
      #   [min]        [min]  mAU    *s   [mAU   ]      %
    ----|-------|----|-------|----------|----------|--------|
       1  22.588 BB    0.1649 1374.50415  120.66559 100.0000

    Totals :                  1374.50415  120.66559
```

HPLC trace of Jun14454

```
Data File C:\CHEM32\1\DATA\BT\_SEQN_ _DATE_ _TIME_ 80\033-0401.D
Sample Name: Jun14454

    =====================================================================
    Acq. Operator   : BT                            Seq. Line :   4
    Acq. Instrument : Instrument 1                   Location : Vial 33
    Injection Date  : 4/25/2025 11:08:41 AM               Inj :   1
                                                  Inj Volume : 4 µl
    Acq. Method     : C:\Chem32\1\DATA\BT\_SeqN_ _Date_ _Time_ 80\COLUMNWASHING.M
    Last changed    : 4/25/2025 11:07:50 AM by BT
                      (modified after loading)
    Analysis Method : C:\CHEM32\1\DATA\BT\_SEQN_ _DATE_ _TIME_ 80\033-0401.D\DA.M (COLUMNWASHING.M)
    Last changed    : 5/12/2025 7:21:54 AM by BT
```

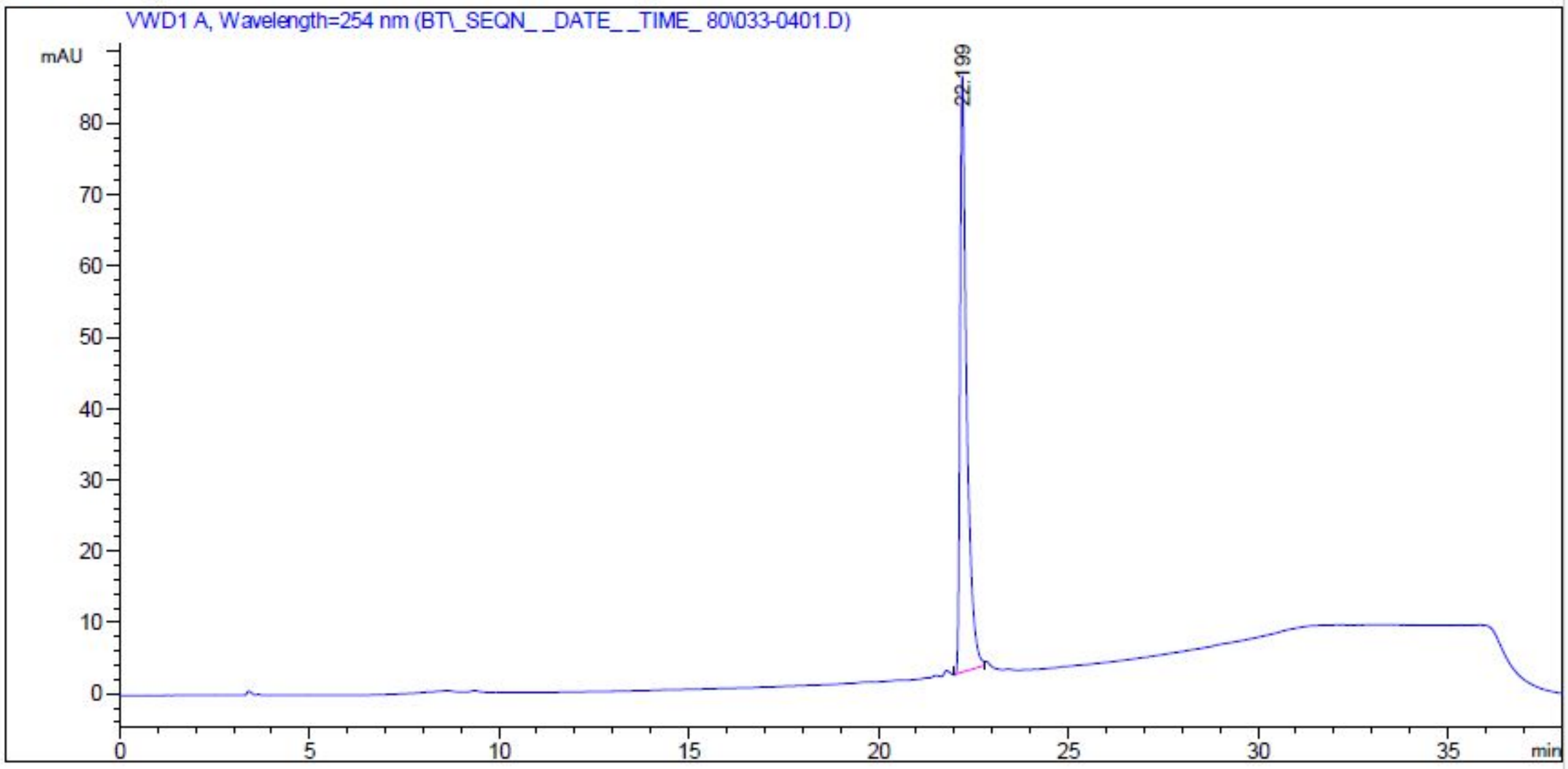


```
    =====================================================================
                         Area Percent Report
    =====================================================================

    Sorted By             :      Signal
    Multiplier            :      1.0000
    Dilution              :      1.0000
    Use Multiplier & Dilution Factor with ISTDs


    Signal 1: VWD1 A, Wavelength=254 nm

    Peak RetTime Type  Width     Area      Height     Area
      #   [min]        [min]  mAU   *s   [mAU   ]       %
    ----|-------|----|-------|----------|----------|--------|
       1  22.199 VB    0.1781 1027.73108   83.78138 100.0000

    Totals :                  1027.73108   83.78138
```

HPLC trace of Jun14855

```
Data File C:\CHEM32\1\DATA\BT\_SEQN_ _DATE_ _TIME_ 84\042-0201.D
Sample Name: Jun14855

    =====================================================================
    Acq. Operator    : BT                              Seq. Line :   2
    Acq. Instrument : Instrument 1                      Location : Vial 42
    Injection Date  : 4/28/2025 10:41:39 AM                  Inj :   1
                                                     Inj Volume : 4 µl
    Acq. Method     : C:\Chem32\1\DATA\BT\_SeqN_ _Date_ _Time_ 84\COLUMNWASHING.M
    Last changed    : 4/28/2025 8:27:18 AM by BT
    Analysis Method : C:\CHEM32\1\DATA\BT\_SEQN_ _DATE_ _TIME_ 84\042-0201.D\DA.M (COLUMNWASHING.M)
    Last changed    : 5/12/2025 7:05:29 AM by BT
```

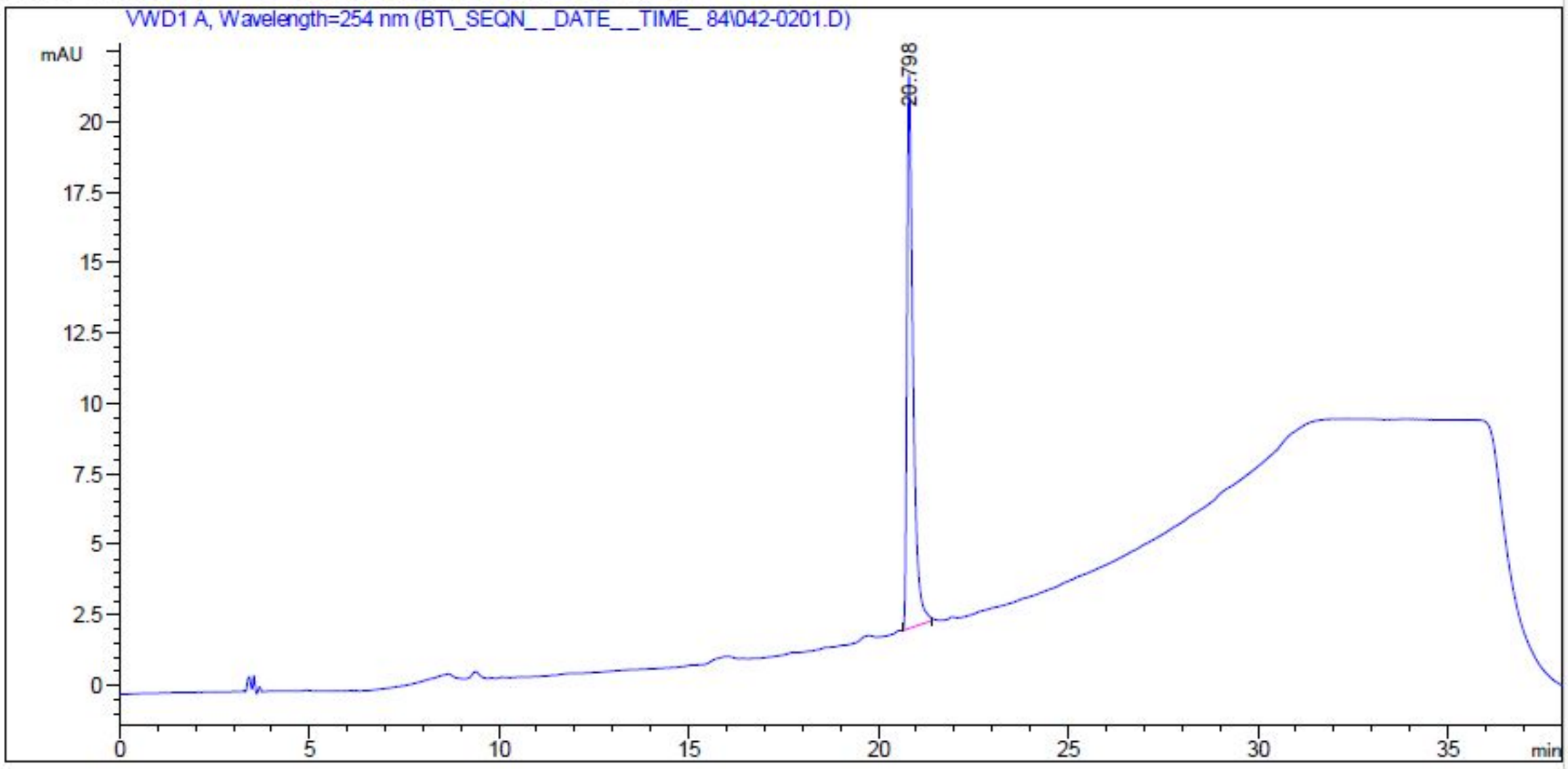


```
    =====================================================================
                             Area Percent Report
    =====================================================================

    Sorted By             :      Signal
    Multiplier            :      1.0000
    Dilution              :      1.0000
    Use Multiplier & Dilution Factor with ISTDs


    Signal 1: VWD1 A, Wavelength=254 nm

    Peak RetTime Type  Width     Area      Height     Area
      #   [min]        [min]  mAU   *s    [mAU   ]      %
    ----|-------|----|-------|----------|----------|--------|
       1  20.798 BB    0.1606  219.44821   19.68630 100.0000

    Totals :                   219.44821   19.68630
```

HPLC trace of Jun14458

```
Data File C:\CHEM32\1\DATA\BT\_SEQN_ _DATE_ _TIME_ 80\034-0501.D
Sample Name: Jun14458

=====================================================================
Acq. Operator   : BT                            Seq. Line :   5
Acq. Instrument : Instrument 1                   Location : Vial 34
Injection Date  : 4/25/2025 11:47:50 AM               Inj :   1
                                               Inj Volume : 4 µl
Acq. Method     : C:\Chem32\1\DATA\BT\_SeqN_ _Date_ _Time_ 80\COLUMNWASHING.M
Last changed    : 4/25/2025 11:46:59 AM by BT
                  (modified after loading)
Analysis Method : C:\CHEM32\1\DATA\BT\_SEQN_ _DATE_ _TIME_ 80\034-0501.D\DA.M (COLUMNWASHING.M)
Last changed    : 5/12/2025 7:22:23 AM by BT
```

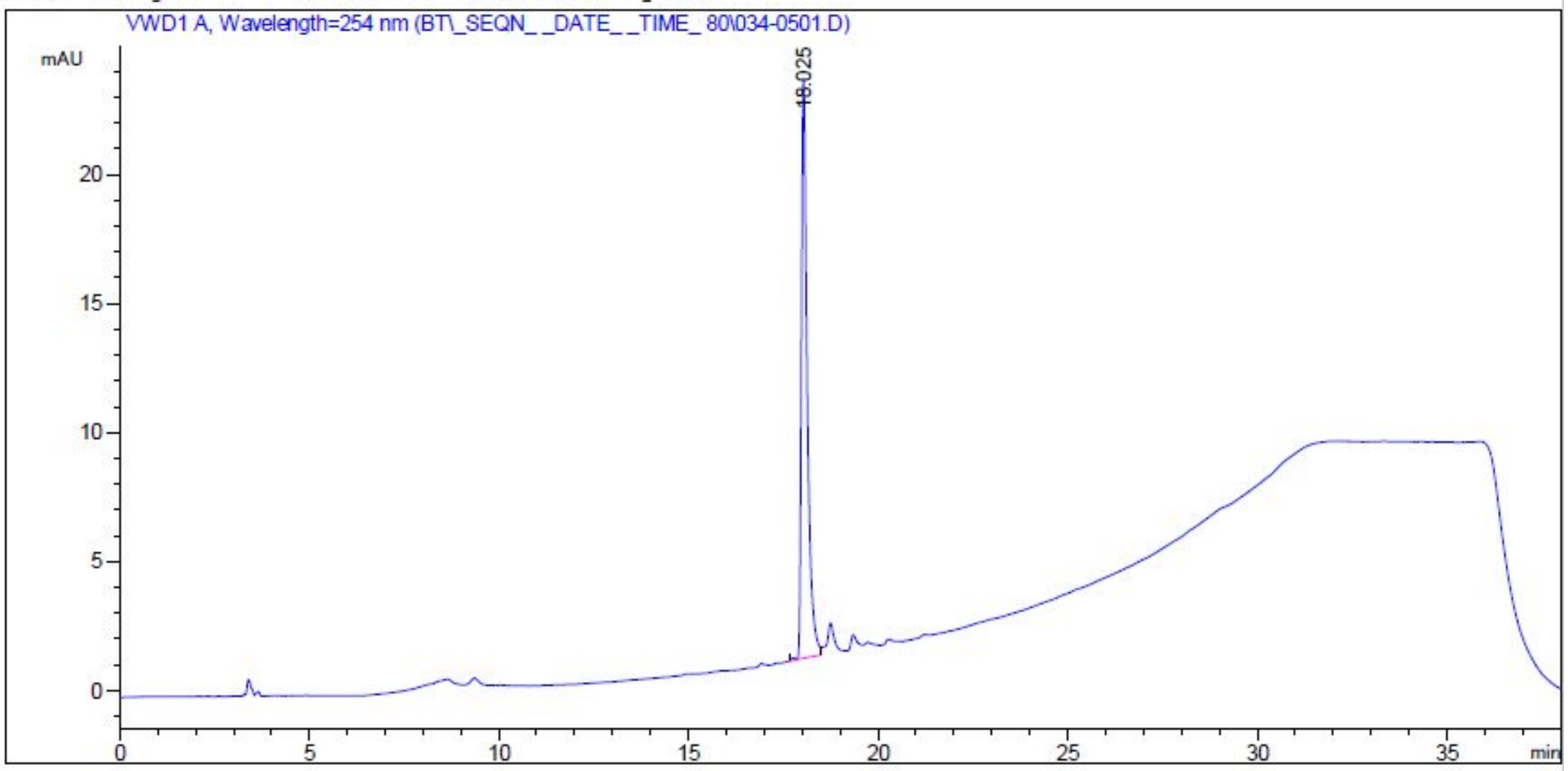


```
=====================================================================
                        Area Percent Report
=====================================================================

Sorted By             :      Signal
Multiplier            :      1.0000
Dilution              :      1.0000
Use Multiplier & Dilution Factor with ISTDs


Signal 1: VWD1 A, Wavelength=254 nm

Peak RetTime Type  Width     Area      Height     Area
  #   [min]        [min]  mAU   *s   [mAU   ]       %
----|-------|----|-------|----------|----------|--------|
   1  18.025 BB    0.1565  239.98911   22.50075 100.0000

Totals :                   239.98911   22.50075
```

HPLC trace of Jun14856

```
Data File C:\CHEM32\1\DATA\BT\_SEQN_ _DATE_ _TIME_ 84\043-0301.D
Sample Name: Jun14856

    =====================================================================
    Acq. Operator   : BT                             Seq. Line :    3
    Acq. Instrument : Instrument 1                    Location : Vial 43
    Injection Date  : 4/28/2025 11:20:44 AM                Inj :    1
                                                    Inj Volume : 4 µl
    Acq. Method     : C:\Chem32\1\DATA\BT\_SeqN_ _Date_ _Time_ 84\COLUMNWASHING.M
    Last changed    : 4/28/2025 8:27:18 AM by BT
    Analysis Method : C:\CHEM32\1\DATA\BT\_SEQN_ _DATE_ _TIME_ 84\043-0301.D\DA.M (COLUMNWASHING.M)
    Last changed    : 5/12/2025 7:05:54 AM by BT
```

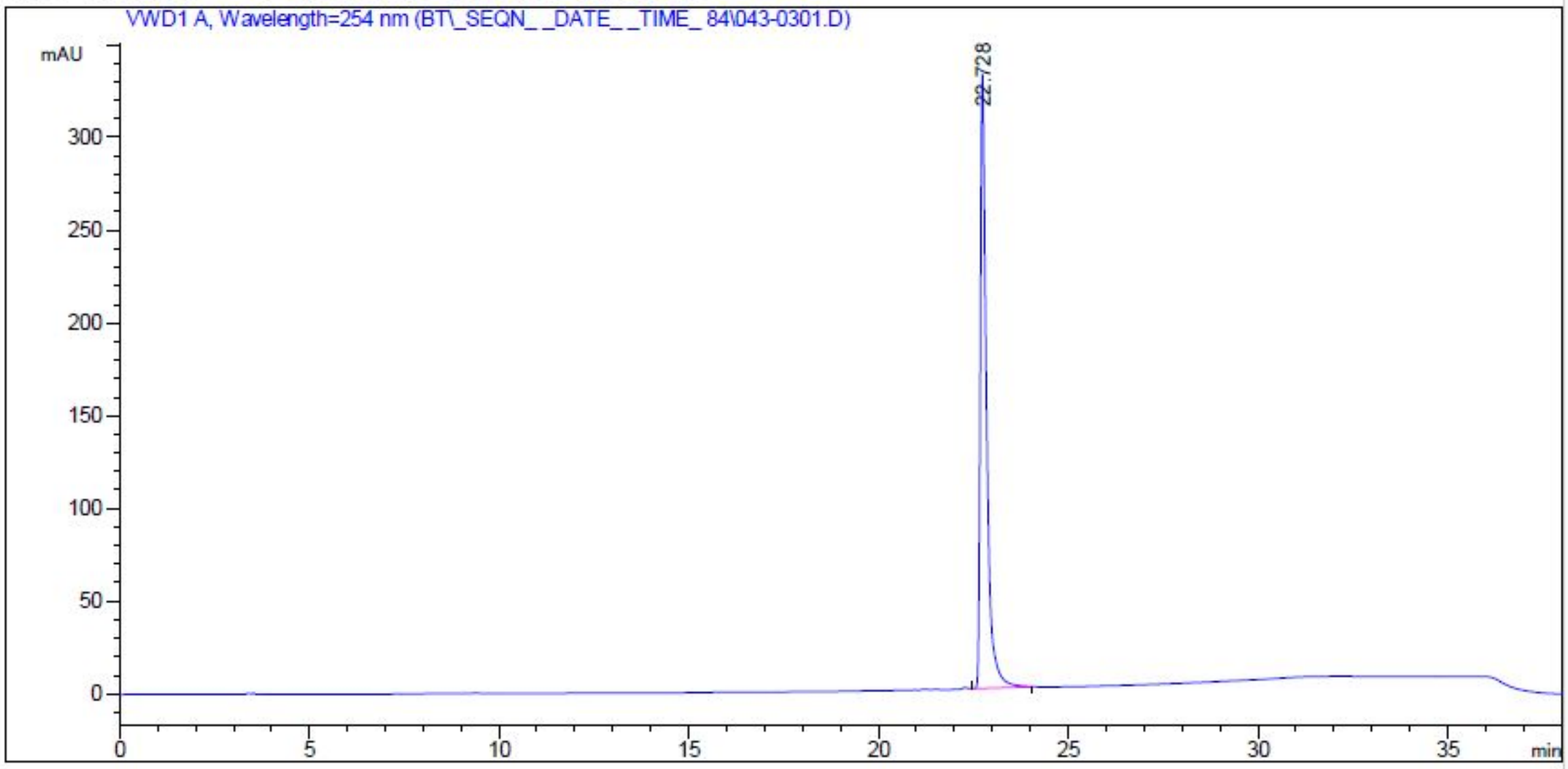


```
    =====================================================================
                             Area Percent Report
    =====================================================================

    Sorted By             :      Signal
    Multiplier            :      1.0000
    Dilution              :      1.0000
    Use Multiplier & Dilution Factor with ISTDs


    Signal 1: VWD1 A, Wavelength=254 nm

    Peak RetTime Type  Width     Area      Height     Area
      #   [min]        [min]  mAU   *s   [mAU   ]      %
    ----|-------|----|-------|----------|----------|--------|
       1  22.728 VB    0.1625 3789.97437  331.41028 100.0000

    Totals :                  3789.97437  331.41028
```

HPLC trace of Jun14924

```
Data File C:\CHEM32\1\DATA\BT\_SEQN_ _DATE_ _TIME_ 84\046-0701.D
Sample Name: Jun14924

    =====================================================================
    Acq. Operator   : BT                             Seq. Line :    7
    Acq. Instrument : Instrument 1                    Location : Vial 46
    Injection Date  : 4/28/2025 1:57:17 PM                 Inj :    1
                                                    Inj Volume : 4 µl
    Acq. Method     : C:\Chem32\1\DATA\BT\_SeqN_ _Date_ _Time_ 84\COLUMNWASHING.M
    Last changed    : 4/28/2025 8:27:18 AM by BT
    Analysis Method : C:\CHEM32\1\DATA\BT\_SEQN_ _DATE_ _TIME_ 84\046-0701.D\DA.M (COLUMNWASHING.M)
    Last changed    : 5/12/2025 7:07:41 AM by BT
```

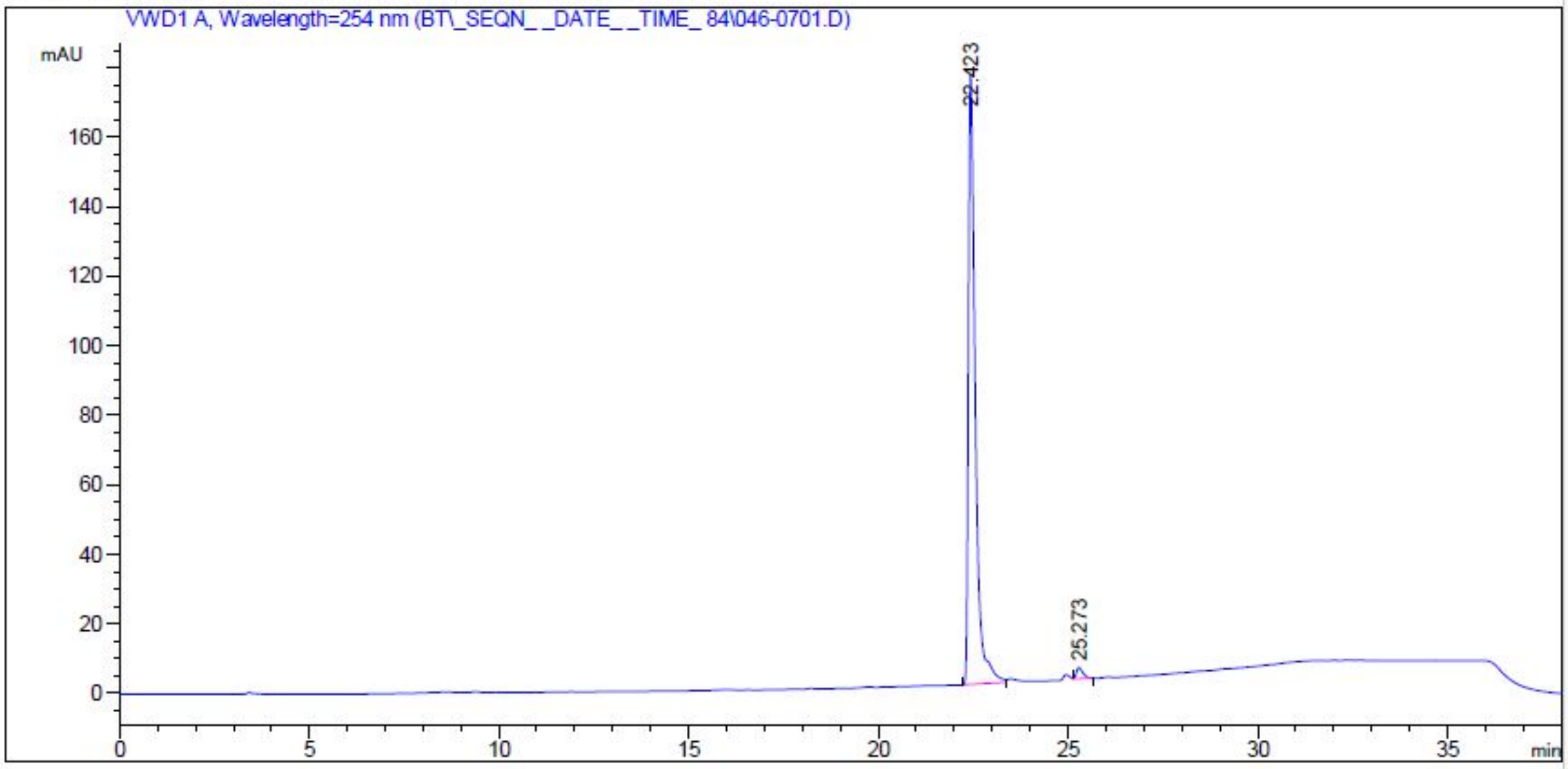


```
    =====================================================================
                             Area Percent Report
    =====================================================================

    Sorted By             :      Signal
    Multiplier            :      1.0000
    Dilution              :      1.0000
    Use Multiplier & Dilution Factor with ISTDs


    Signal 1: VWD1 A, Wavelength=254 nm

    Peak RetTime Type  Width     Area      Height     Area
      #   [min]        [min]  mAU   *s   [mAU   ]      %
    ----|-------|----|-------|----------|----------|--------|
       1  22.423 BV    0.1911 2217.88354  175.56058  98.1835
       2  25.273 VB    0.1802   41.03257    3.32893   1.8165

    Totals :                  2258.91611  178.88951
```

HPLC trace of Jun14782

```
Data File C:\CHEM32\1\DATA\BT\_SEQN_ _DATE_ _TIME_ 82\042-1401.D
Sample Name: Jun14782

    =====================================================================
    Acq. Operator   : BT                              Seq. Line :  14
    Acq. Instrument : Instrument 1                     Location : Vial 42
    Injection Date  : 4/27/2025 11:45:44 AM                 Inj :   1
                                                    Inj Volume : 4 µl
    Acq. Method     : C:\Chem32\1\DATA\BT\_SeqN_ _Date_ _Time_ 82\COLUMNWASHING.M
    Last changed    : 4/27/2025 11:44:57 AM by BT
                      (modified after loading)
    Analysis Method : C:\CHEM32\1\DATA\BT\_SEQN_ _DATE_ _TIME_ 82\042-1401.D\DA.M (COLUMNWASHING.M)
    Last changed    : 4/27/2025 1:07:18 AM by BT
```

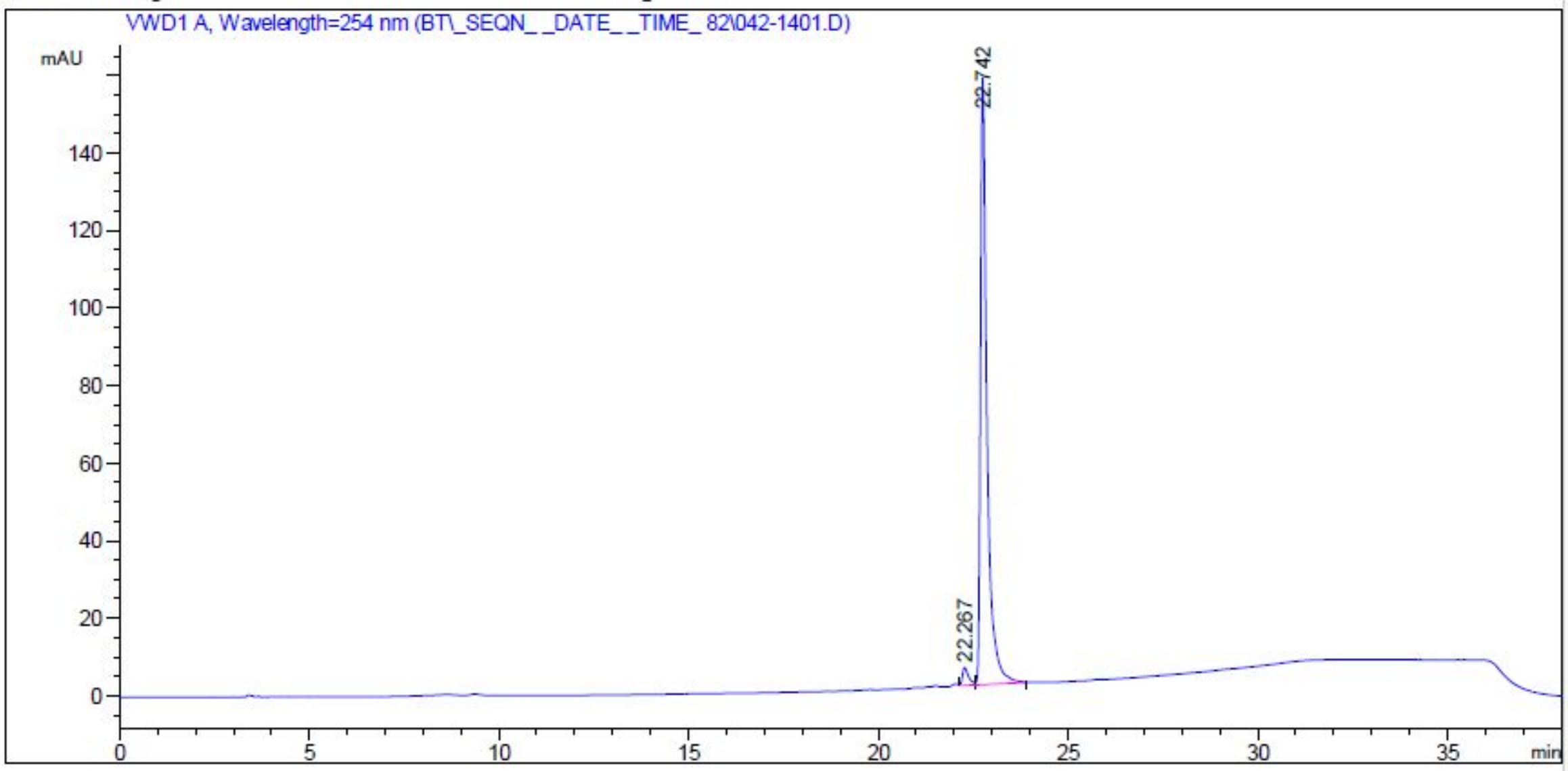


```
    =====================================================================
                             Area Percent Report
    =====================================================================

    Sorted By             :      Signal
    Multiplier            :      1.0000
    Dilution              :      1.0000
    Use Multiplier & Dilution Factor with ISTDs


    Signal 1: VWD1 A, Wavelength=254 nm

    Peak RetTime Type  Width     Area      Height     Area
      #   [min]        [min]  mAU   *s    [mAU   ]      %
    ----|-------|----|-------|----------|----------|--------|
       1  22.267 VV    0.1725   55.70543    4.72344   2.8761
       2  22.742 VB    0.1750 1881.11584  156.71054  97.1239

    Totals :                  1936.82127  161.43399
```

HPLC trace of Jun14966

```
Data File C:\CHEM32\1\DATA\BT\_SEQN_ _DATE_ _TIME_ 84\049-1001.D
Sample Name: Jun14966

    =====================================================================
    Acq. Operator   : BT                              Seq. Line :  10
    Acq. Instrument : Instrument 1                     Location : Vial 49
    Injection Date  : 4/28/2025 3:54:33 PM                  Inj :   1
                                                     Inj Volume : 4 µl
    Acq. Method     : C:\Chem32\1\DATA\BT\_SeqN_ _Date_ _Time_ 84\COLUMNWASHING.M
    Last changed    : 4/28/2025 8:27:18 AM by BT
    Analysis Method : C:\CHEM32\1\DATA\BT\_SEQN_ _DATE_ _TIME_ 84\049-1001.D\DA.M (COLUMNWASHING.M)
    Last changed    : 4/28/2025 8:27:18 AM by BT
```

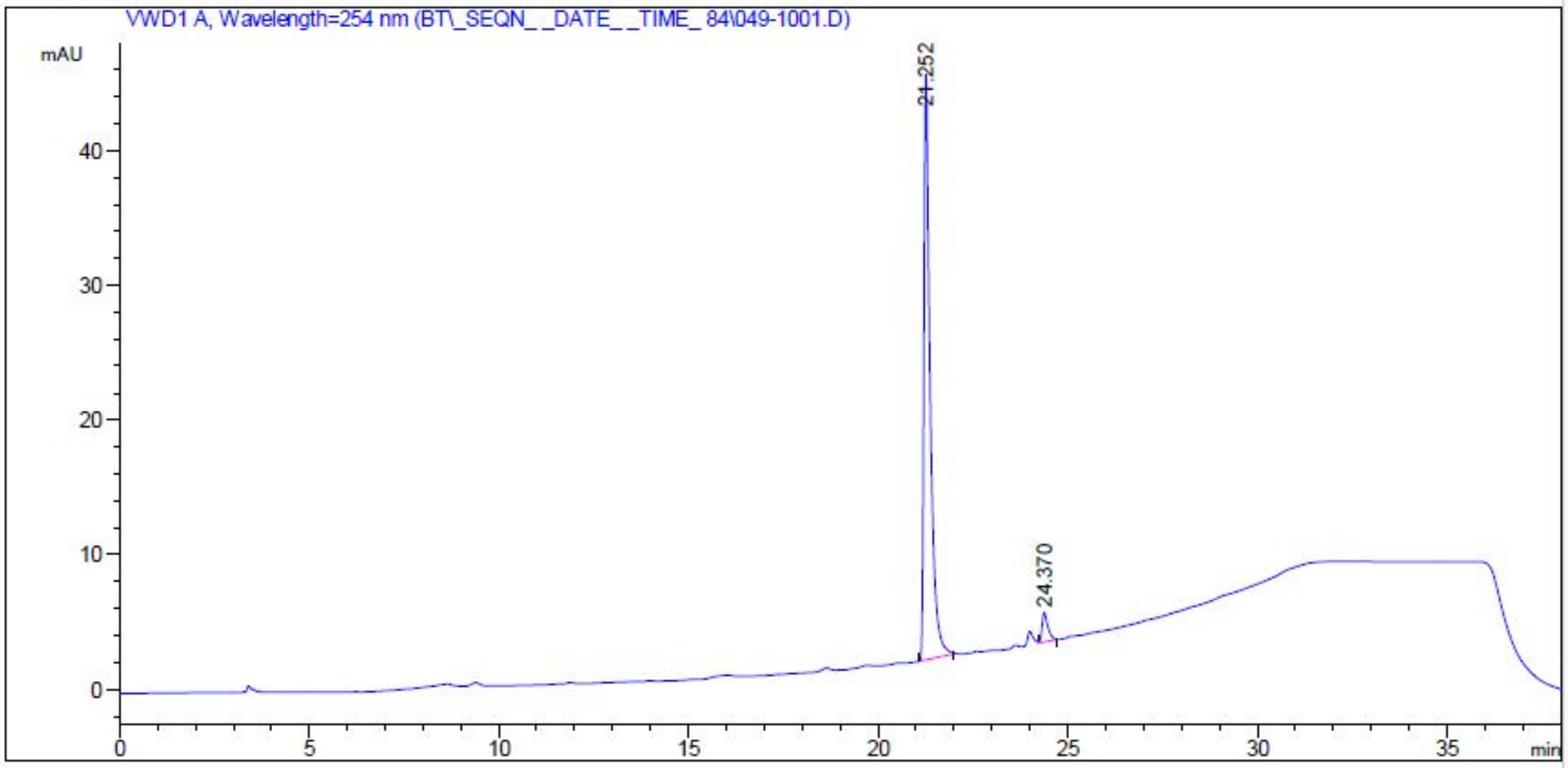


```
    =====================================================================
                             Area Percent Report
    =====================================================================

    Sorted By             :      Signal
    Multiplier            :      1.0000
    Dilution              :      1.0000
    Use Multiplier & Dilution Factor with ISTDs


    Signal 1: VWD1 A, Wavelength=254 nm

    Peak RetTime Type  Width     Area      Height     Area
      #   [min]        [min]  mAU   *s   [mAU   ]      %
    ----|-------|----|-------|----------|----------|--------|
       1  21.252 BB    0.1601  493.43753   43.47648  95.2900
       2  24.370 VB    0.1584   24.38959    2.22684   4.7100

    Totals :                   517.82712   45.70332
```

HPLC trace of Jun14926

```
Data File C:\CHEM32\1\DATA\BT\_SEQN_ _DATE_ _TIME_ 84\047-0801.D
Sample Name: Jun14926

    =====================================================================
    Acq. Operator   : BT                            Seq. Line :   8
    Acq. Instrument : Instrument 1                   Location : Vial 47
    Injection Date  : 4/28/2025 2:36:20 PM                Inj :   1
                                                Inj Volume : 4 µl
    Acq. Method     : C:\Chem32\1\DATA\BT\_SeqN_ _Date_ _Time_ 84\COLUMNWASHING.M
    Last changed    : 4/28/2025 8:27:18 AM by BT
    Analysis Method : C:\CHEM32\1\DATA\BT\_SEQN_ _DATE_ _TIME_ 84\047-0801.D\DA.M (COLUMNWASHING.M)
    Last changed    : 5/12/2025 7:08:12 AM by BT
```

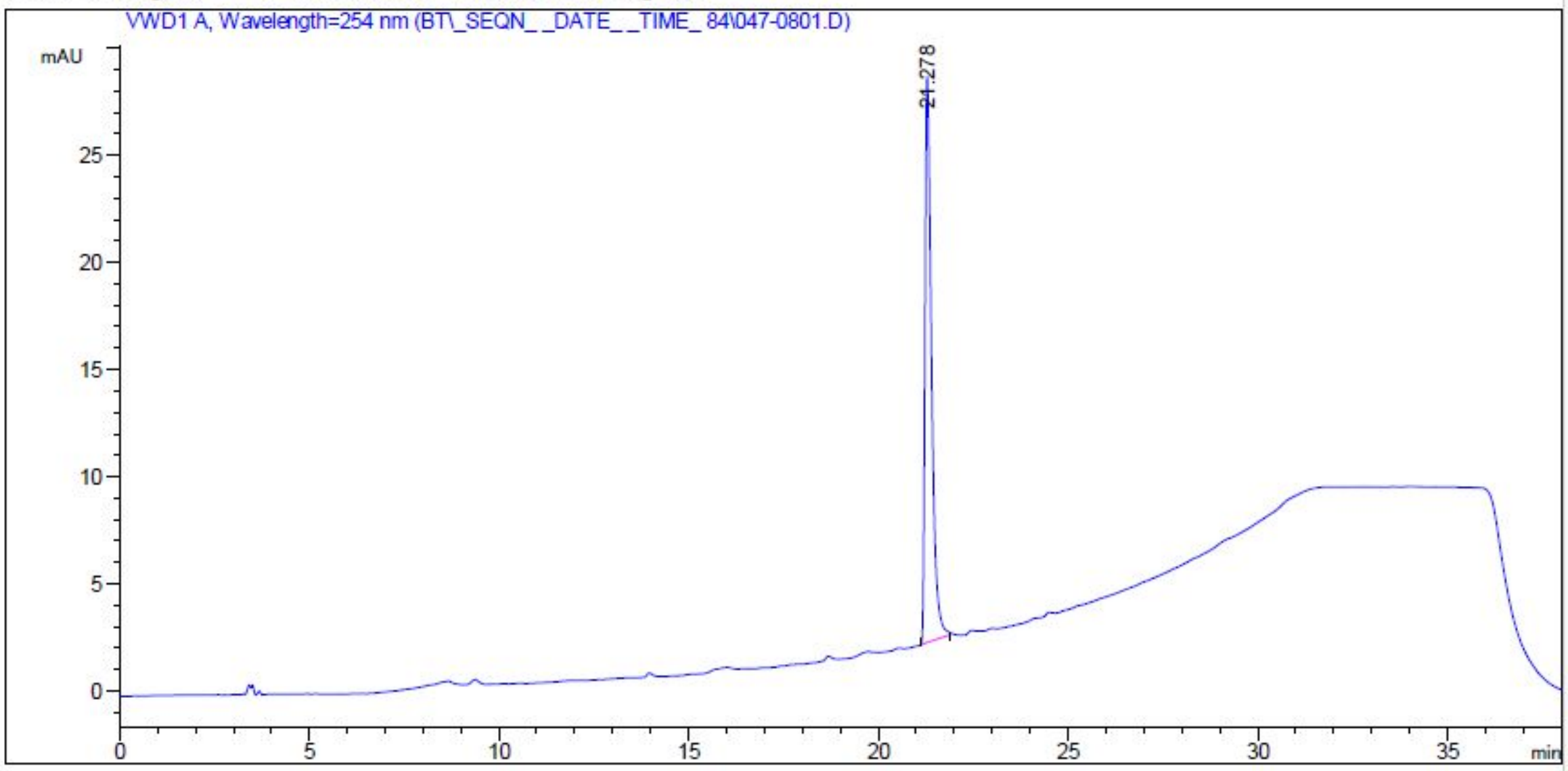


```
    =====================================================================
                         Area Percent Report
    =====================================================================

    Sorted By             :      Signal
    Multiplier            :      1.0000
    Dilution              :      1.0000
    Use Multiplier & Dilution Factor with ISTDs


    Signal 1: VWD1 A, Wavelength=254 nm

    Peak RetTime Type  Width     Area      Height     Area
      #   [min]        [min]  mAU   *s    [mAU   ]      %
    ----|-------|----|-------|----------|----------|--------|
       1  21.278 BB    0.1816  316.17398   26.45972 100.0000

    Totals :                   316.17398   26.45972
```

HPLC trace of Jun15111

```
Data File C:\CHEM32\1\DATA\BT\_SEQN_ _DATE_ _TIME_ 84\052-1401.D
Sample Name: Jun15111

    =====================================================================
    Acq. Operator   : BT                            Seq. Line :  14
    Acq. Instrument : Instrument 1                   Location : Vial 52
    Injection Date  : 4/28/2025 6:30:58 PM                Inj :   1
                                                   Inj Volume : 4 µl
    Acq. Method     : C:\Chem32\1\DATA\BT\_SeqN_ _Date_ _Time_ 84\COLUMNWASHING.M
    Last changed    : 4/28/2025 8:27:18 AM by BT
    Analysis Method : C:\CHEM32\1\DATA\BT\_SEQN_ _DATE_ _TIME_ 84\052-1401.D\DA.M (COLUMNWASHING.M)
    Last changed    : 4/28/2025 8:27:18 AM by BT
```

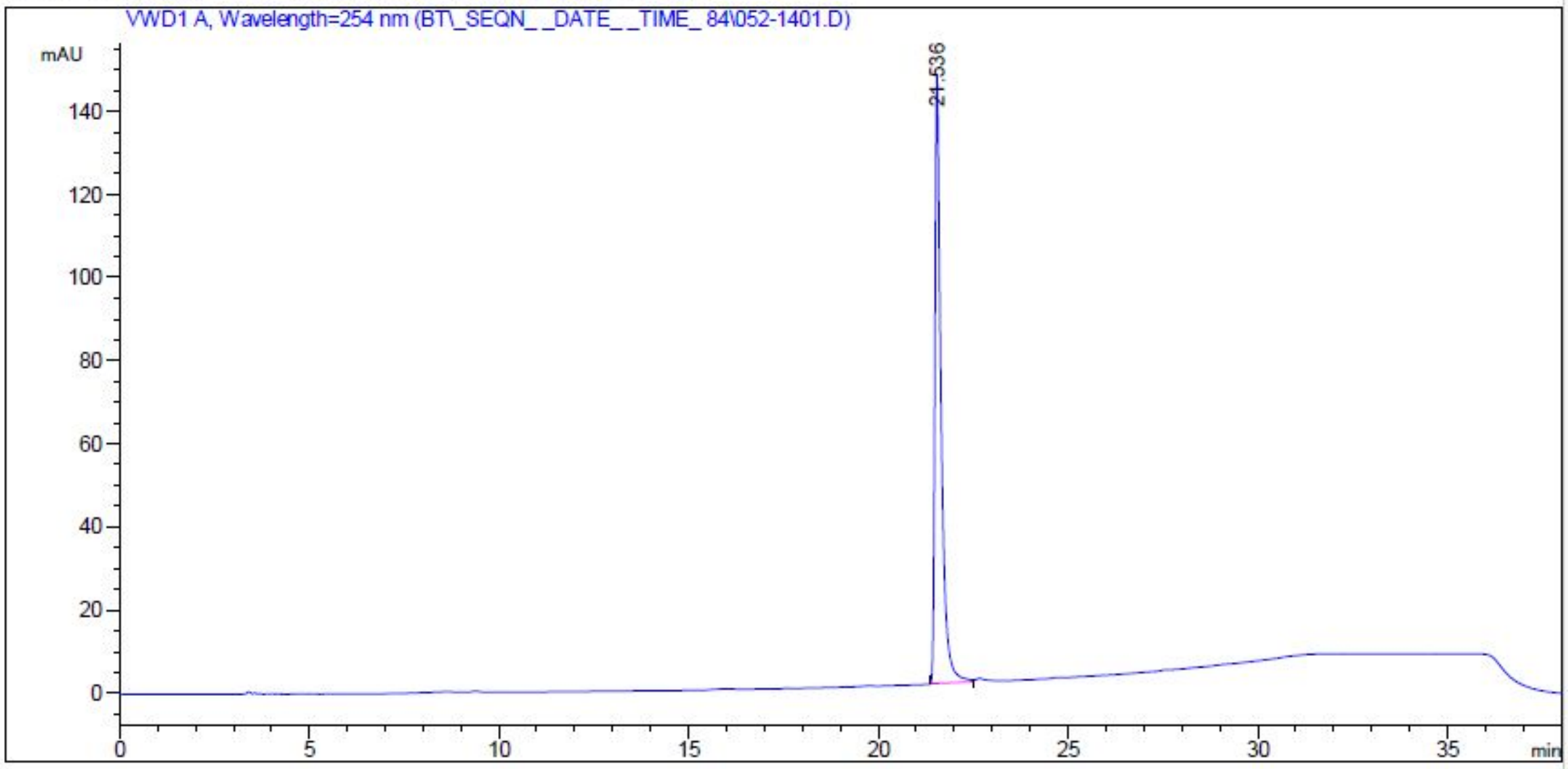


```
    =====================================================================
                            Area Percent Report
    =====================================================================

    Sorted By             :      Signal
    Multiplier            :      1.0000
    Dilution              :      1.0000
    Use Multiplier & Dilution Factor with ISTDs


    Signal 1: VWD1 A, Wavelength=254 nm

    Peak RetTime Type  Width     Area      Height     Area
      #   [min]        [min]  mAU   *s    [mAU   ]      %
    ----|-------|----|-------|----------|----------|--------|
       1  21.536 BB    0.1612 1660.95374  146.73872 100.0000

    Totals :                  1660.95374  146.73872
```

HPLC trace of Jun1551

```
Data File C:\CHEM32\1\DATA\BT\_SEQN_ _DATE_ _TIME_ 84\051-1301.D
Sample Name: Jun1551

=====================================================================
Acq. Operator   : BT                              Seq. Line :  13
Acq. Instrument : Instrument 1                     Location : Vial 51
Injection Date  : 4/28/2025 5:51:51 PM                  Inj :   1
                                                 Inj Volume : 4 µl
Acq. Method     : C:\Chem32\1\DATA\BT\_SeqN_ _Date_ _Time_ 84\COLUMNWASHING.M
Last changed    : 4/28/2025 8:27:18 AM by BT
Analysis Method : C:\CHEM32\1\DATA\BT\_SEQN_ _DATE_ _TIME_ 84\051-1301.D\DA.M (COLUMNWASHING.M)
Last changed    : 5/12/2025 7:14:25 AM by BT
```

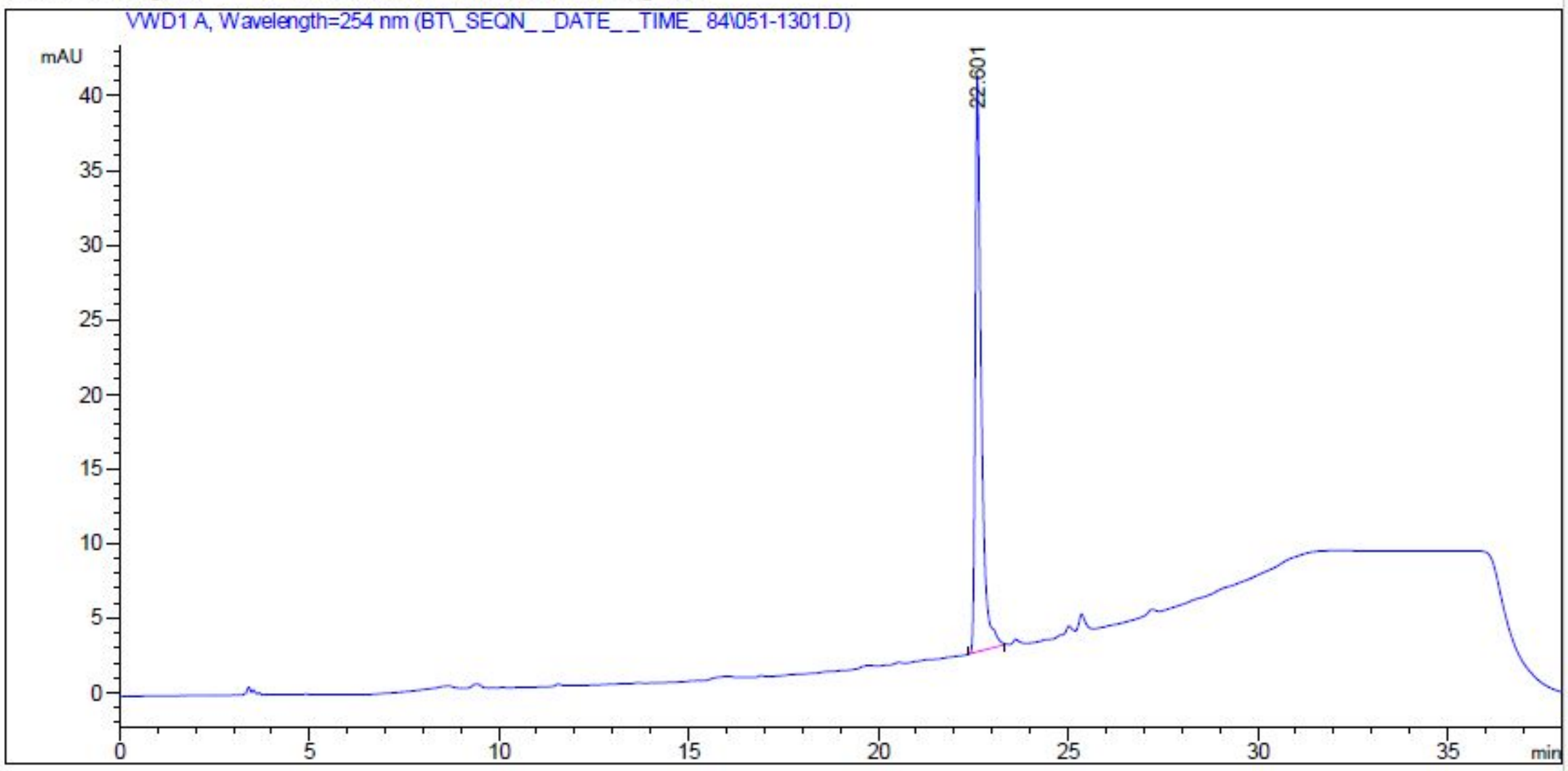


```
=====================================================================
                          Area Percent Report
=====================================================================

Sorted By             :      Signal
Multiplier            :      1.0000
Dilution              :      1.0000
Use Multiplier & Dilution Factor with ISTDs


Signal 1: VWD1 A, Wavelength=254 nm

Peak RetTime Type  Width     Area      Height     Area
  #   [min]        [min]  mAU   *s    [mAU   ]      %
----|-------|----|-------|----------|----------|--------|
   1  22.601 BB    0.1572  428.60748   38.60276 100.0000

Totals :                   428.60748   38.60276
```

HPLC Trace for starting materials (carboxylic acids) and reaction mixtures of nsP2 D2Bs

HPLC trace for Jun14529

8-p077-1s

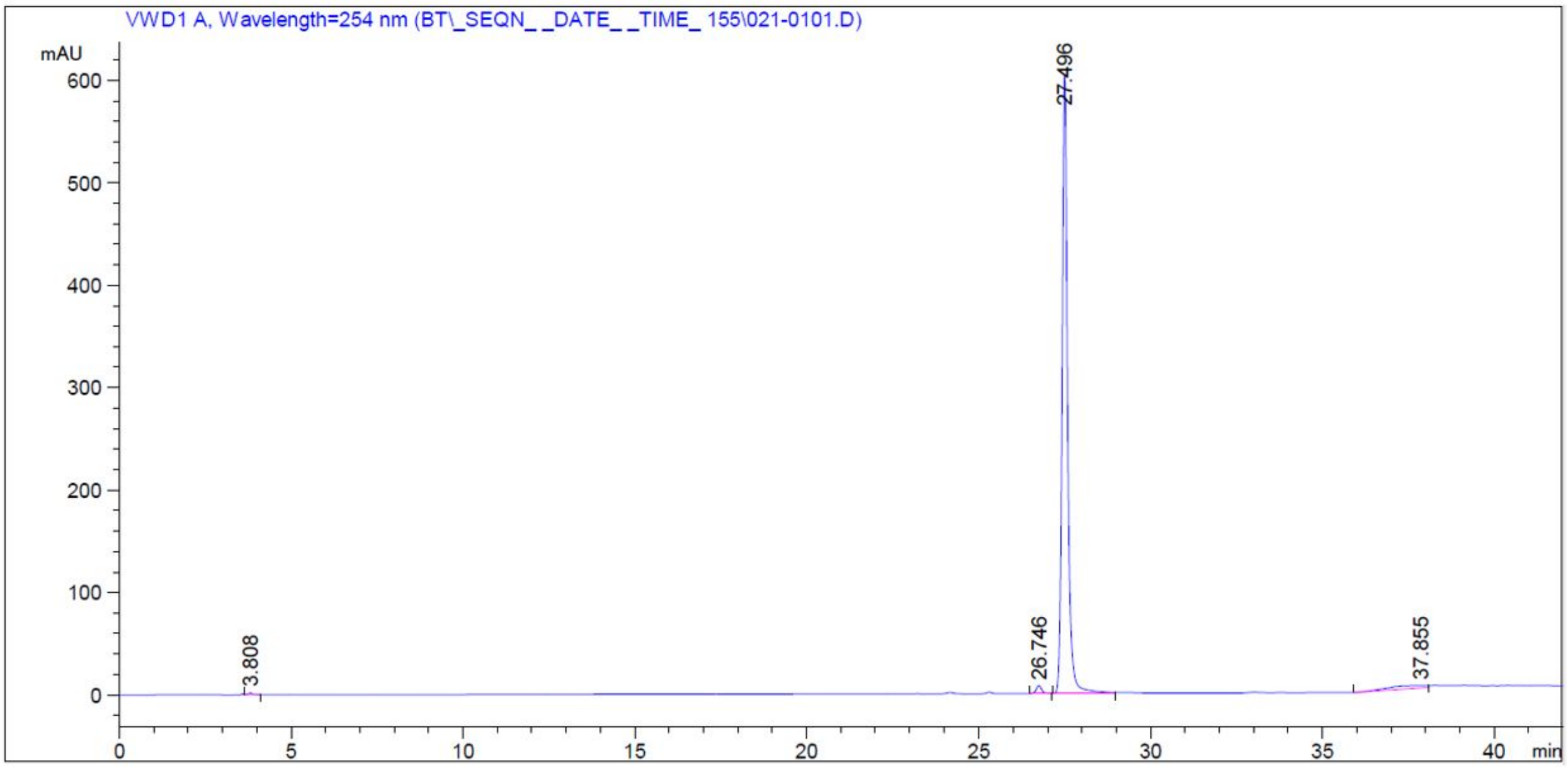

Jun14529 (zc8-p077-1)

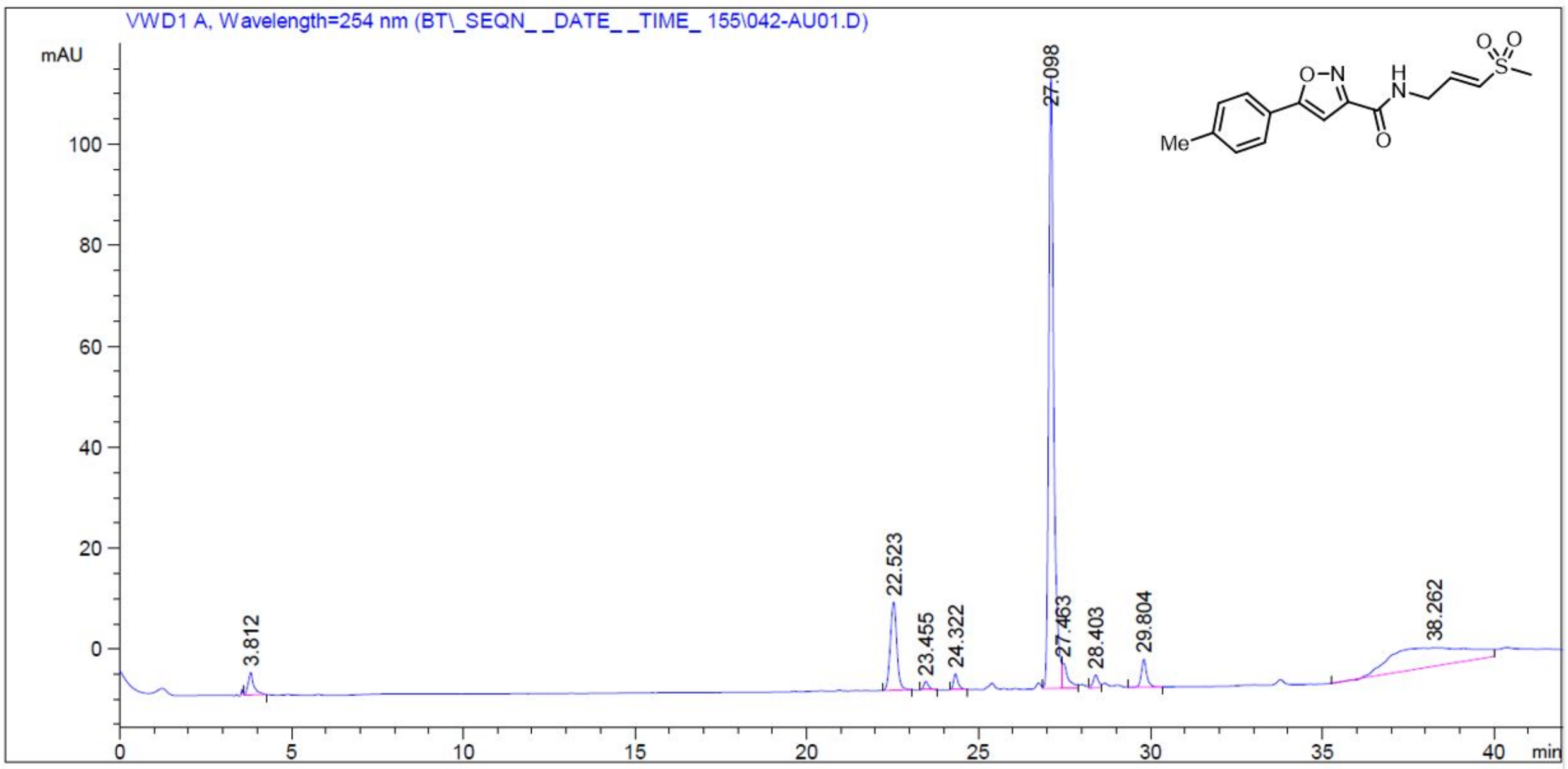

HPLC trace for Jun14502

8-p077-2s

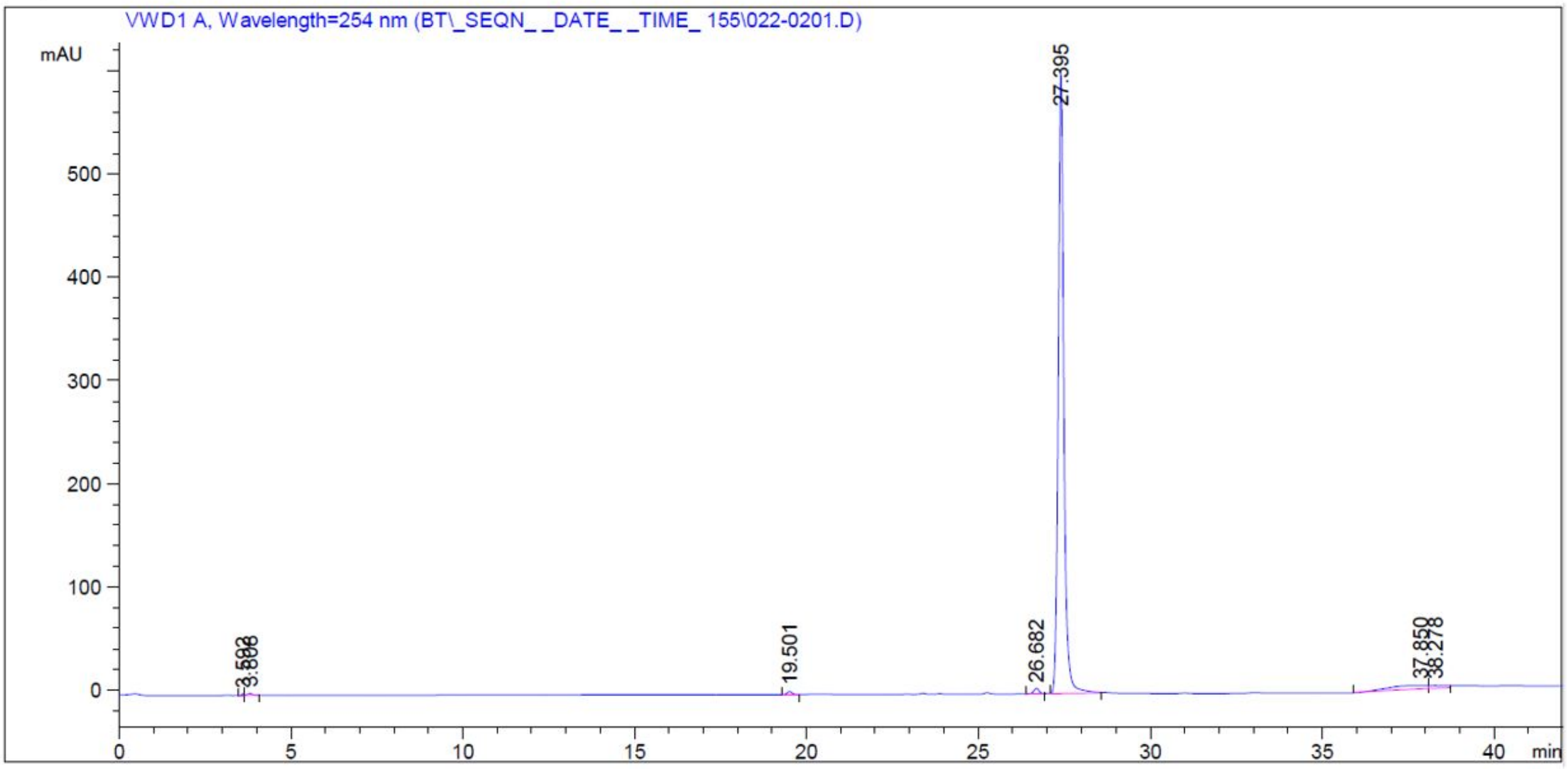

Jun14502 (zc8-p077-2)

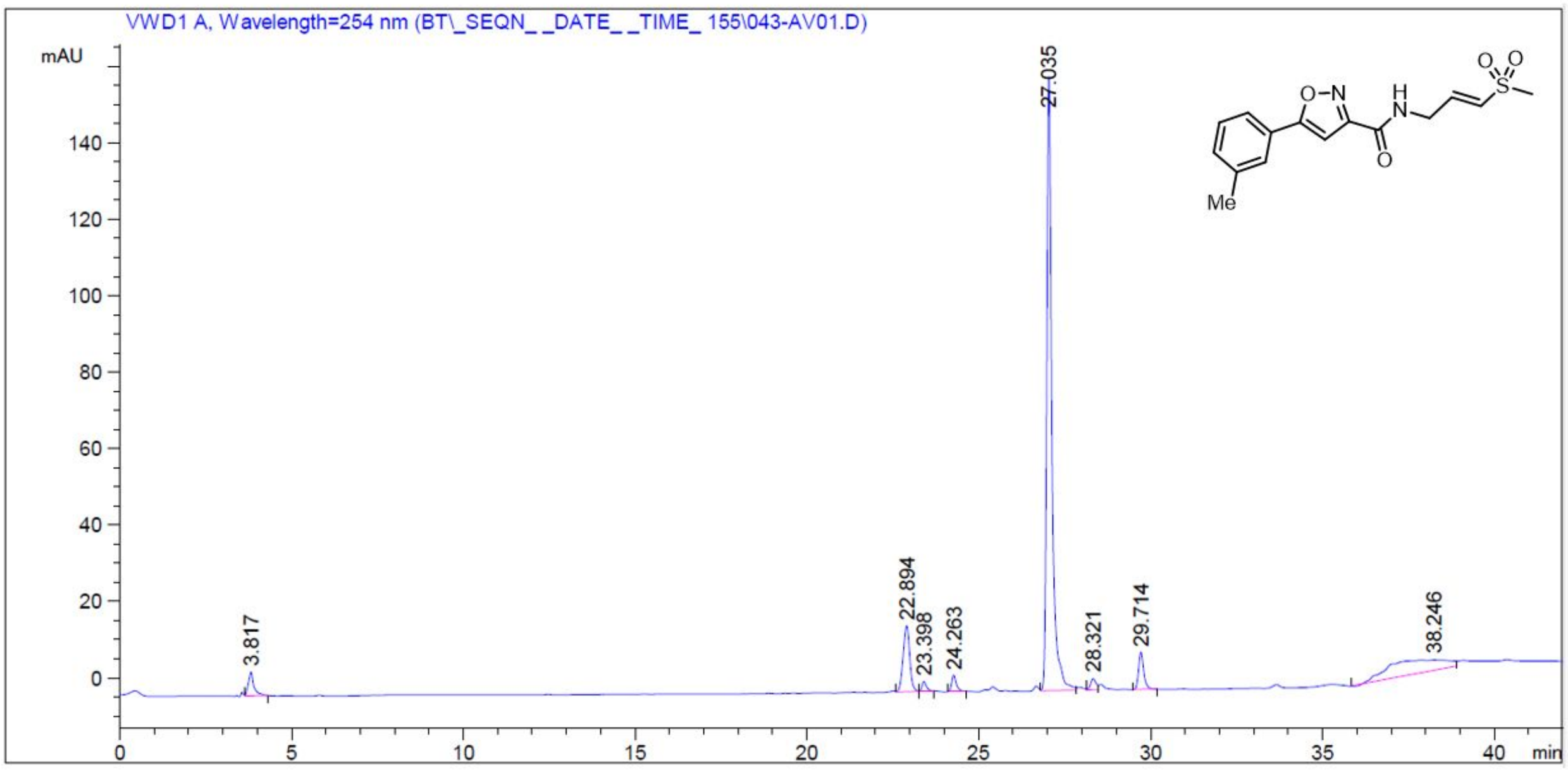

HPLC trace for Jun14528

8-p077-3s

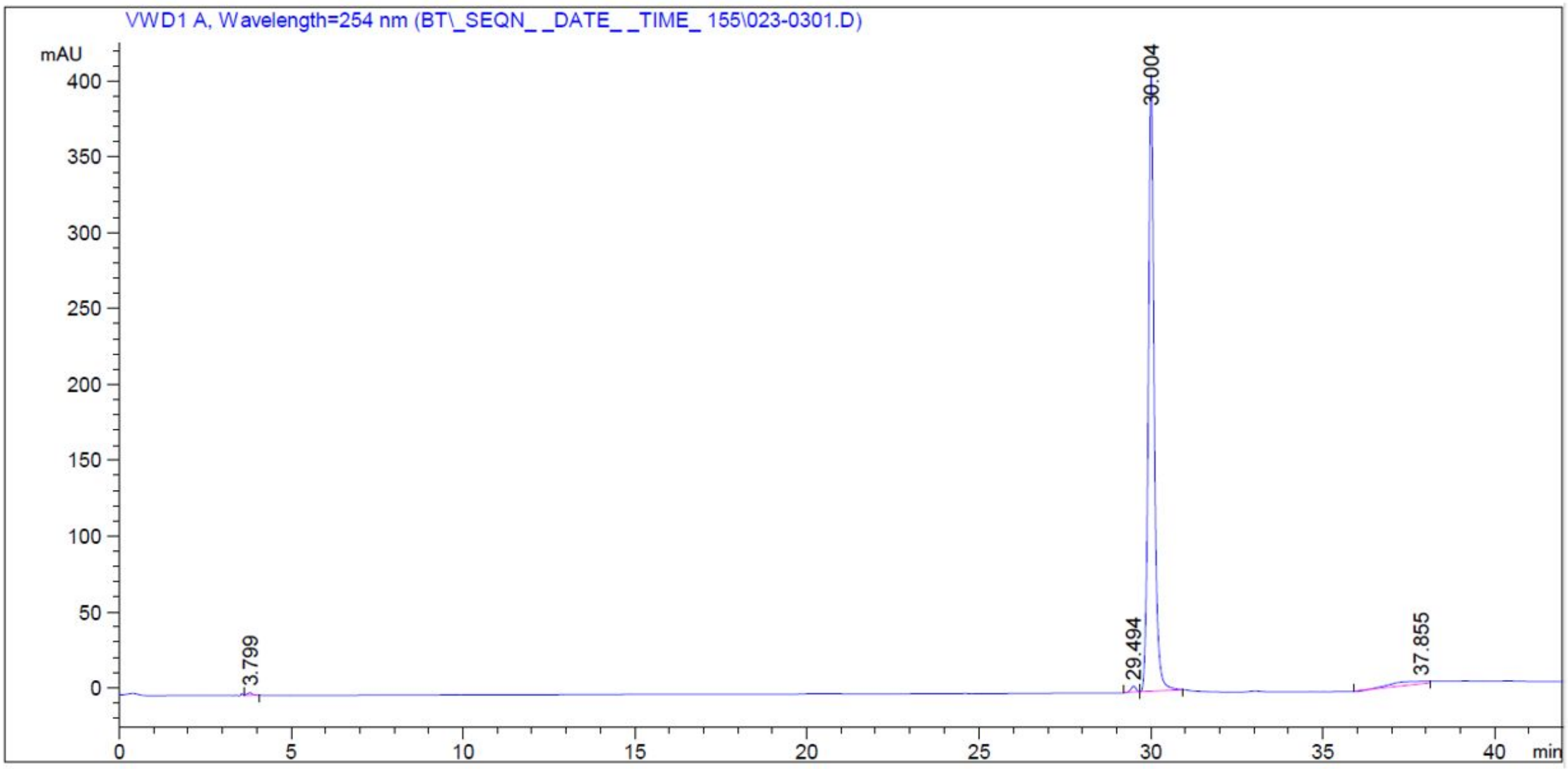

Jun14528 (zc8-p077-3)

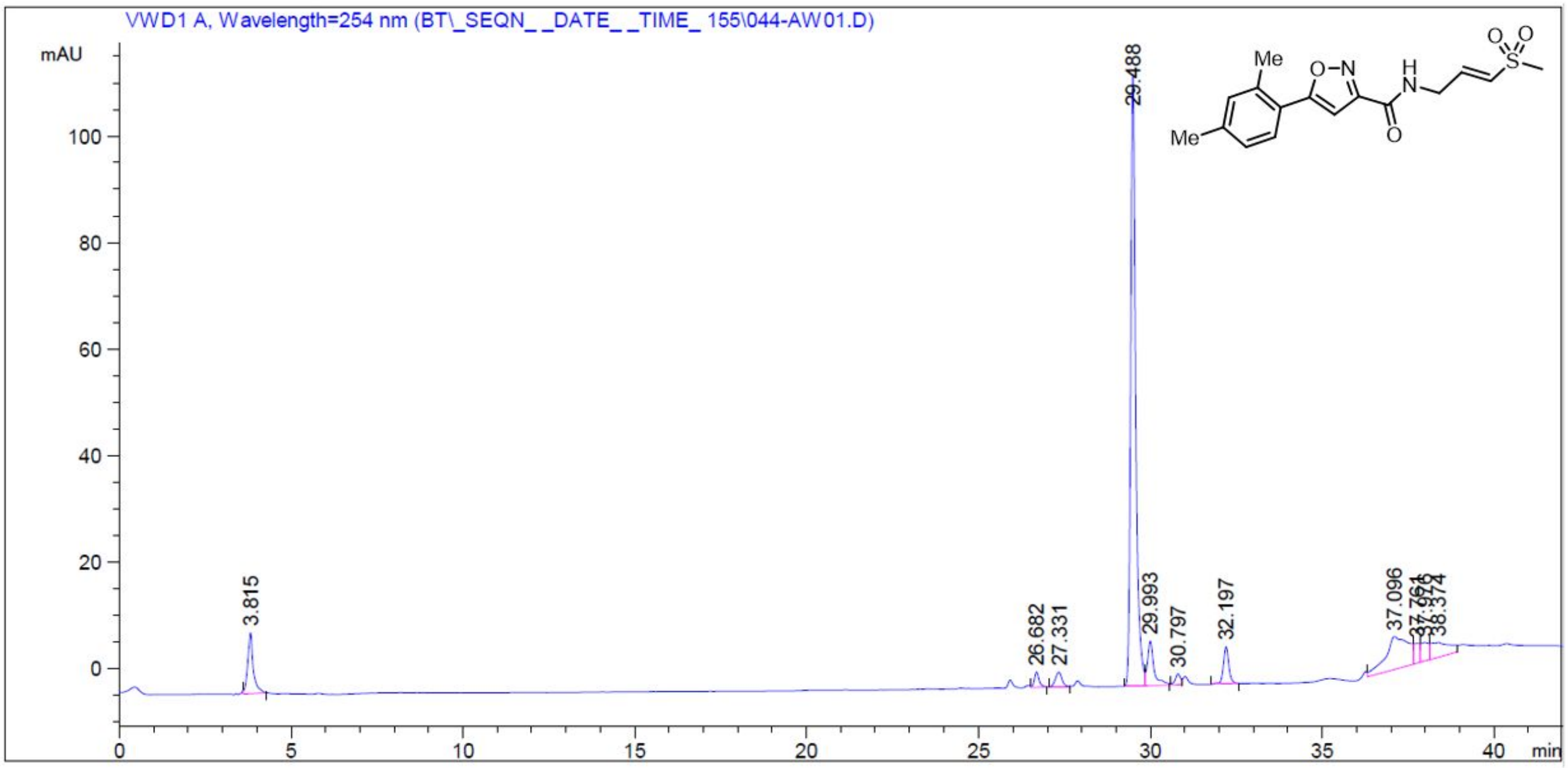

HPLC trace for Jun14617

8-p077-4s

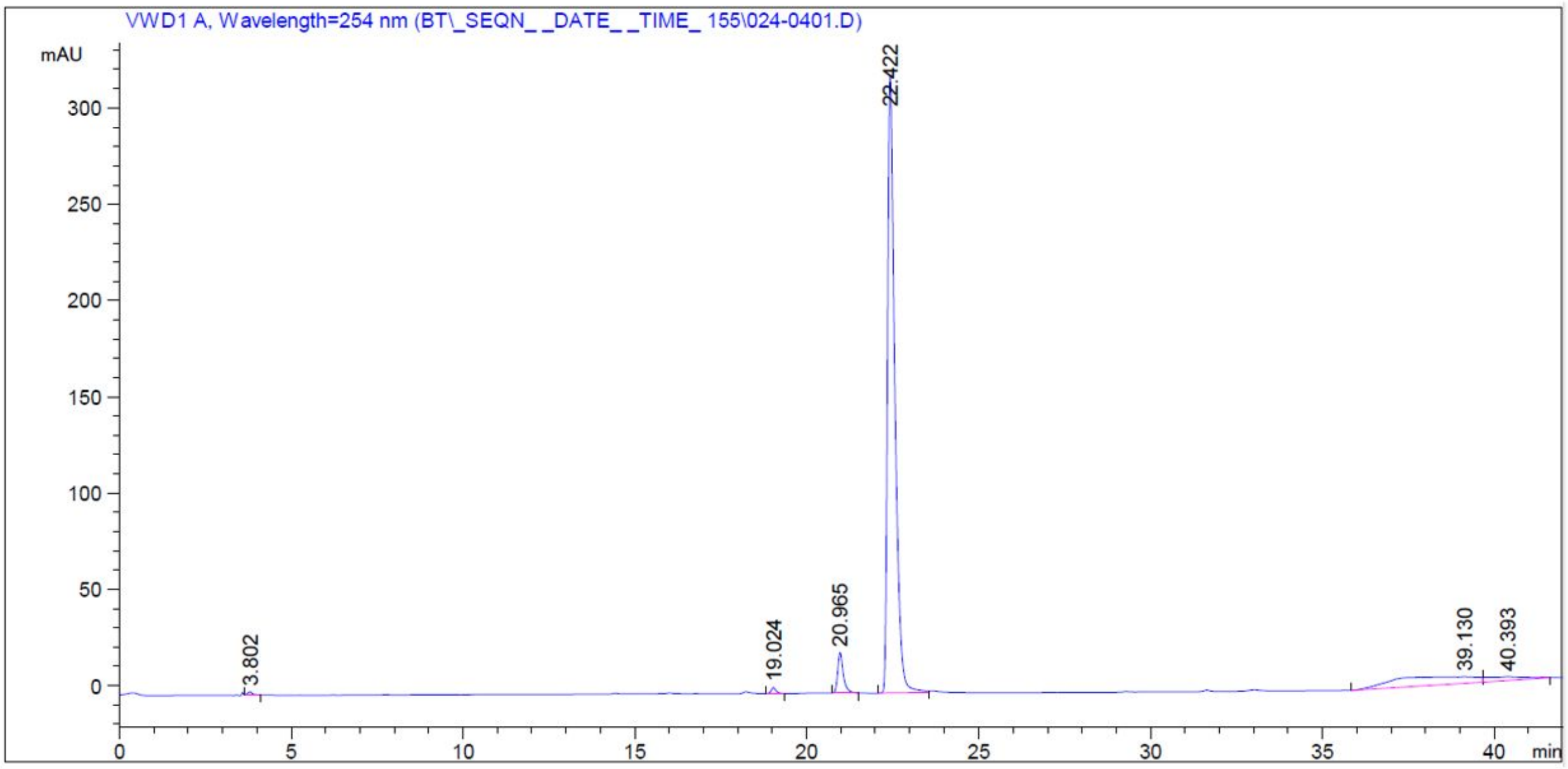

Jun14617 (zc8-p077-4)

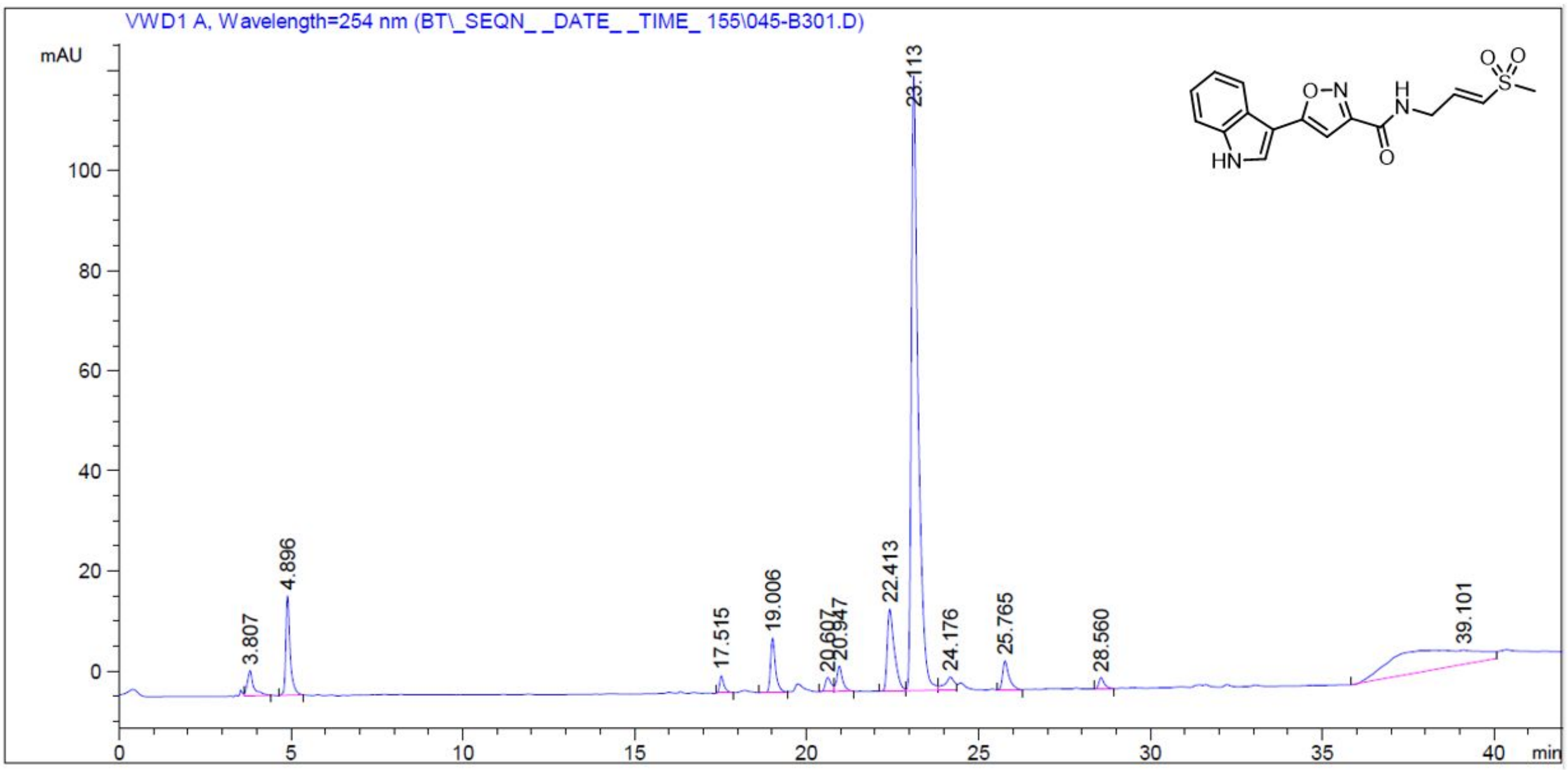

HPLC trace for Jun14736

8-p077-5s

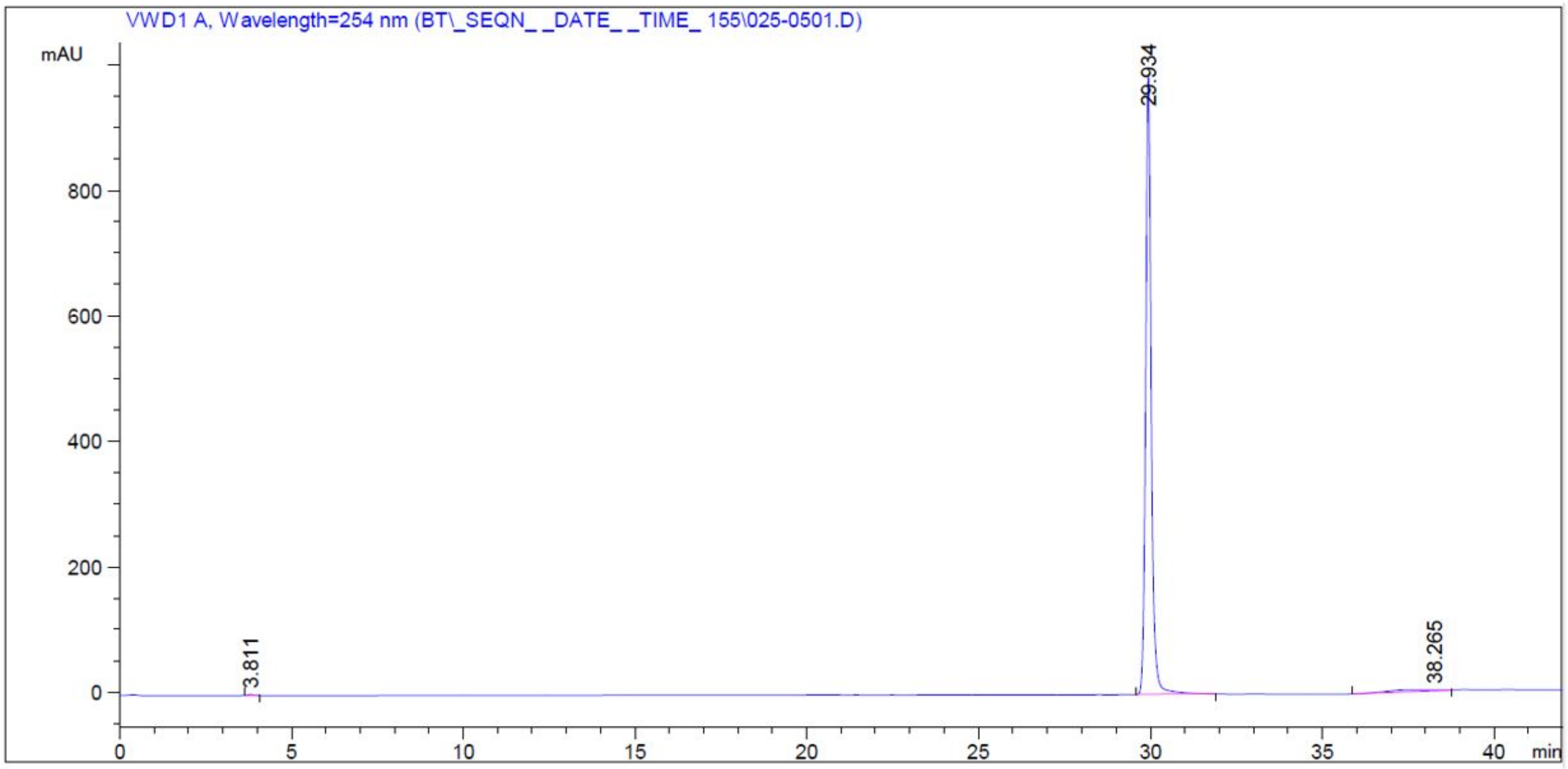

Jun14736 (zc8-p077-5)

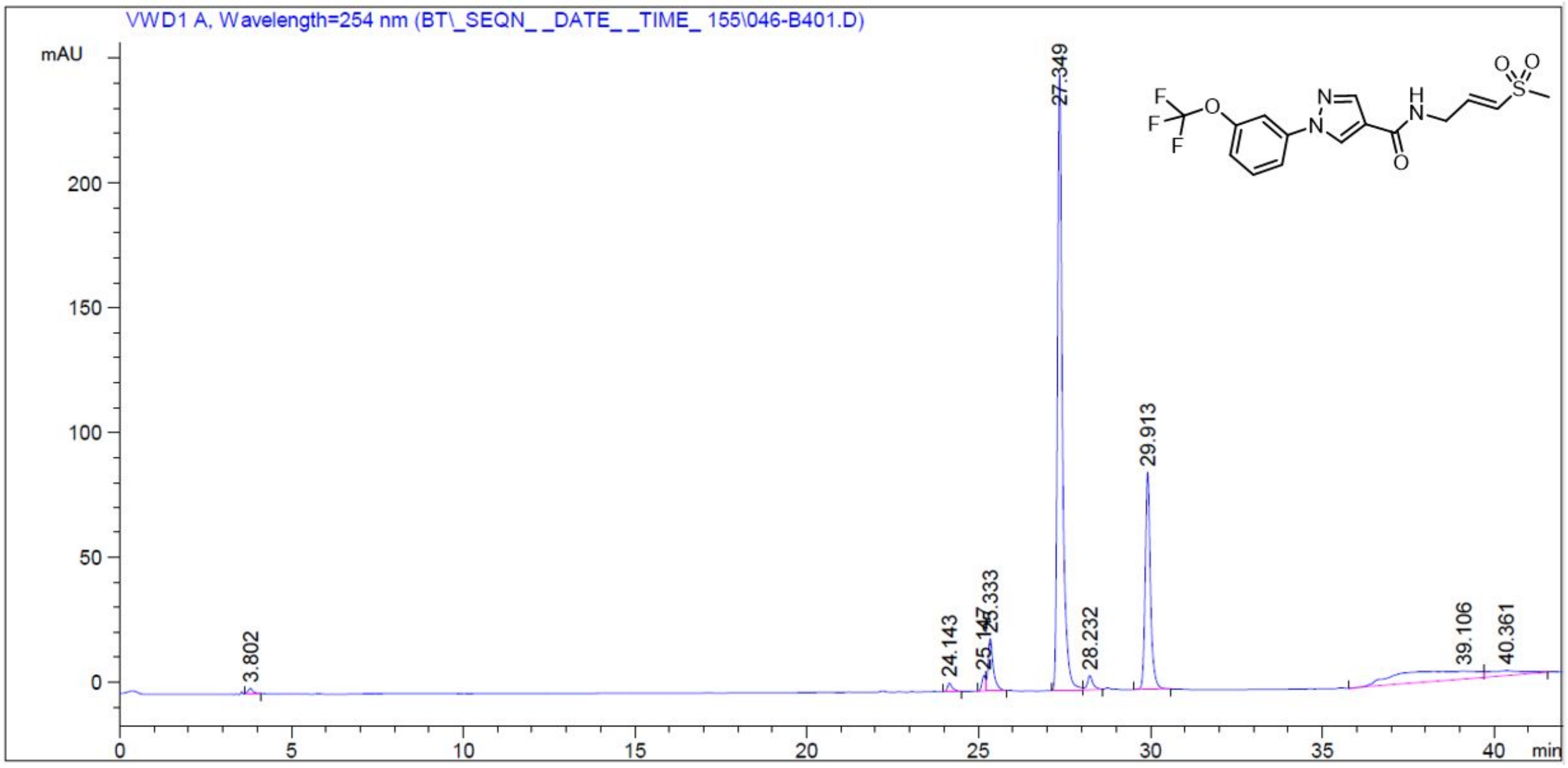

HPLC trace for Jun14741

8-p077-6s

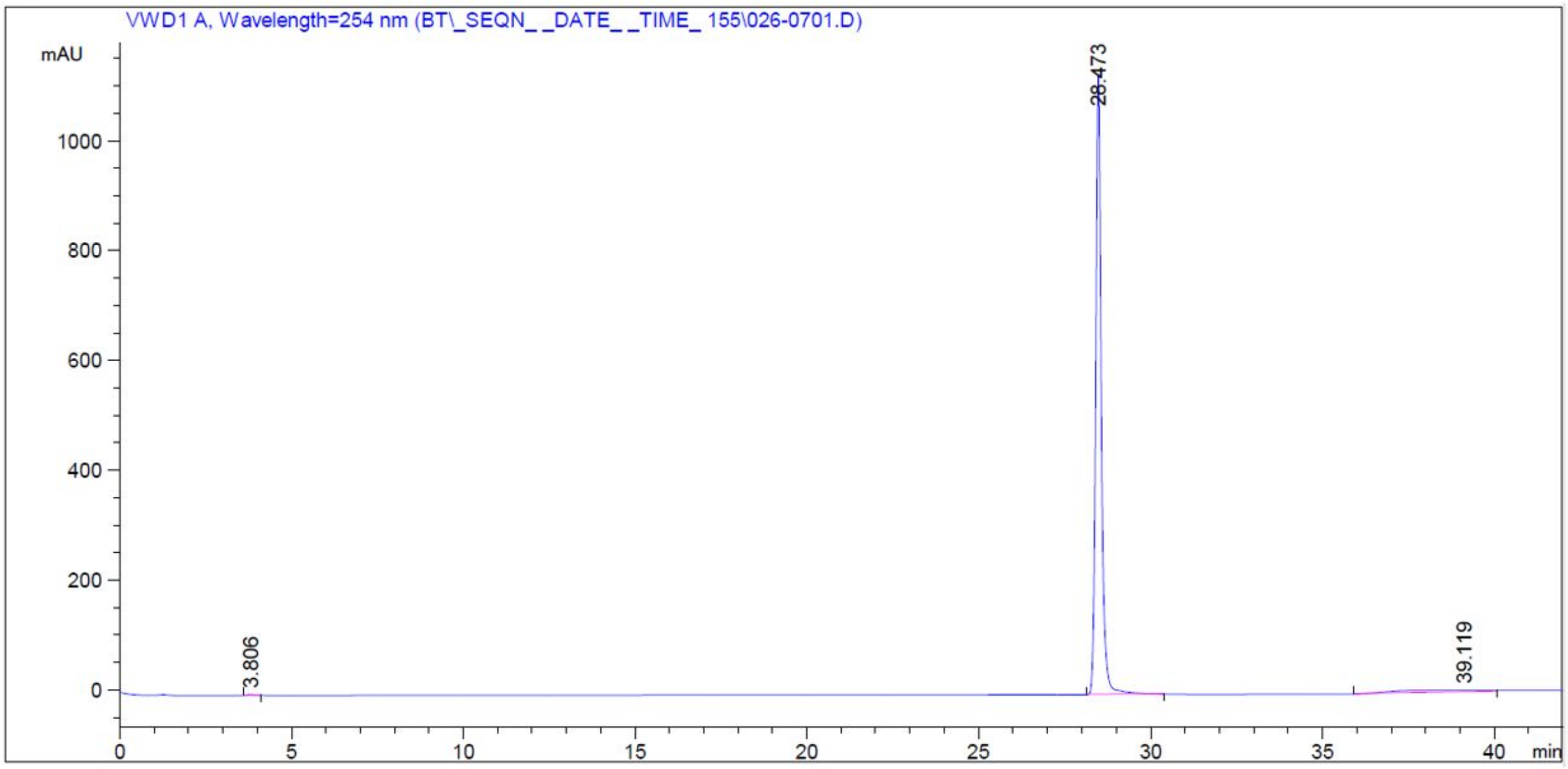

Jun14741 (zc8-p077-6)

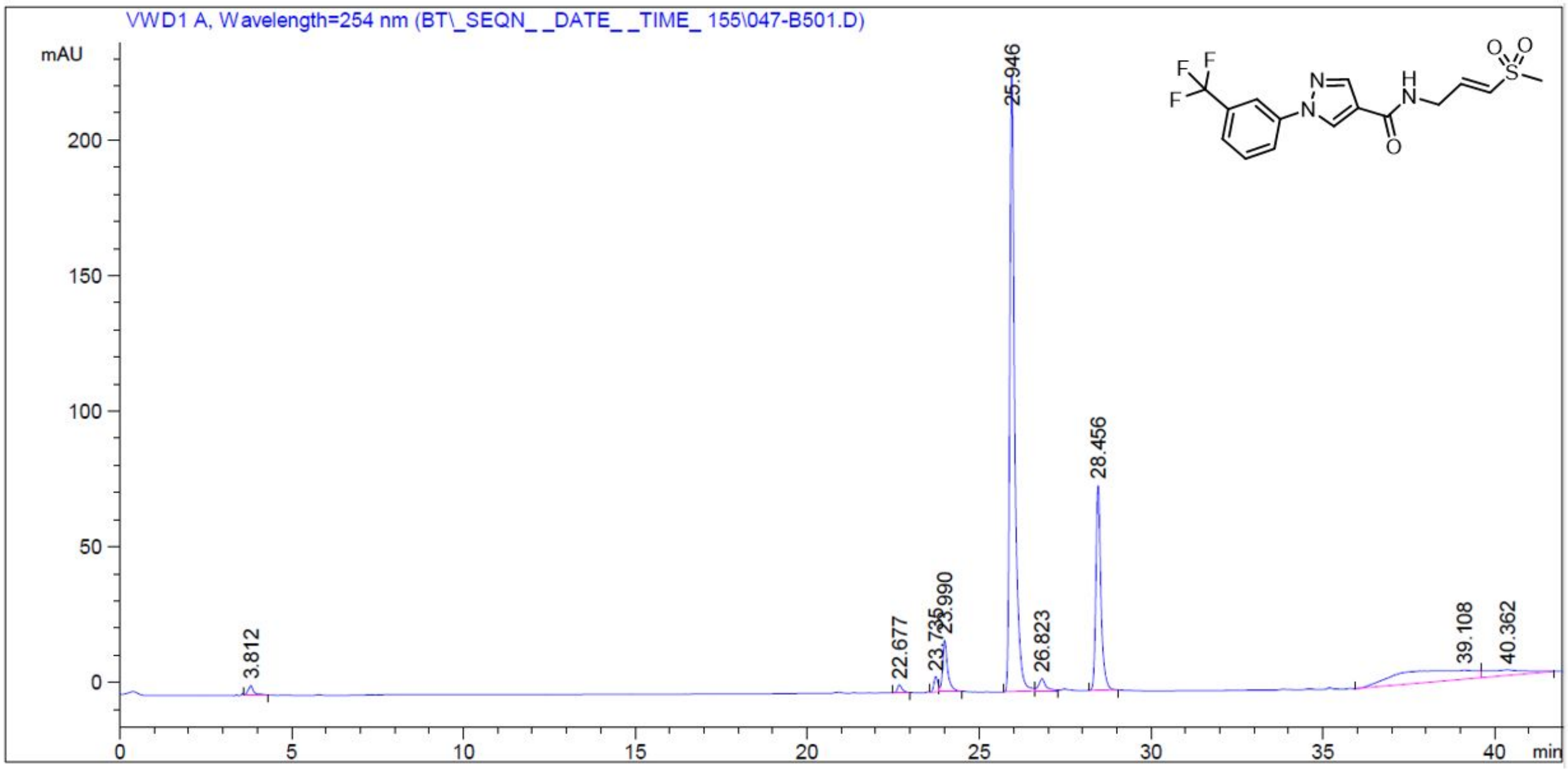

HPLC trace for Jun14363

8-p077-7s

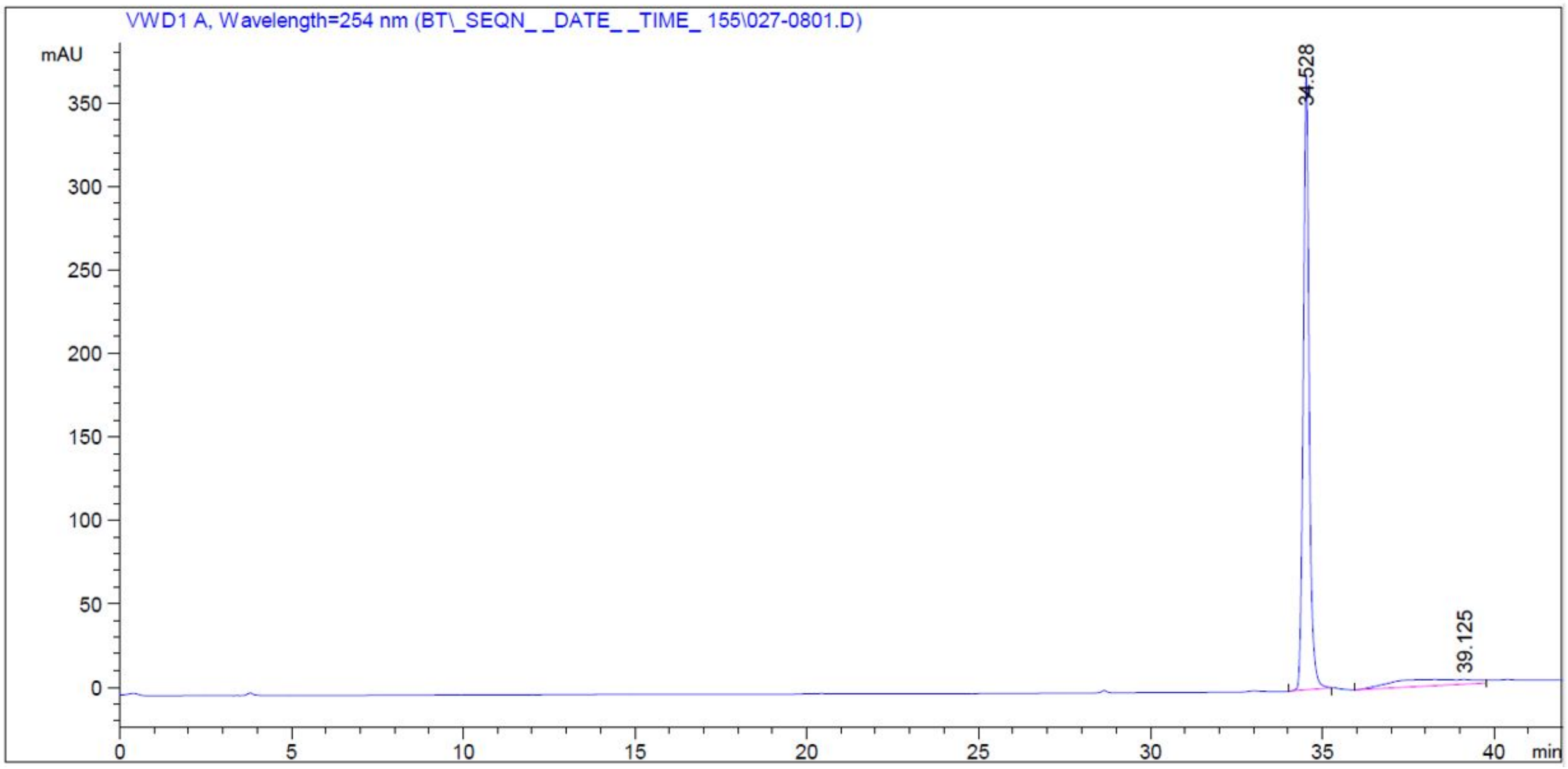

Jun14363 (zc8-p077-7)

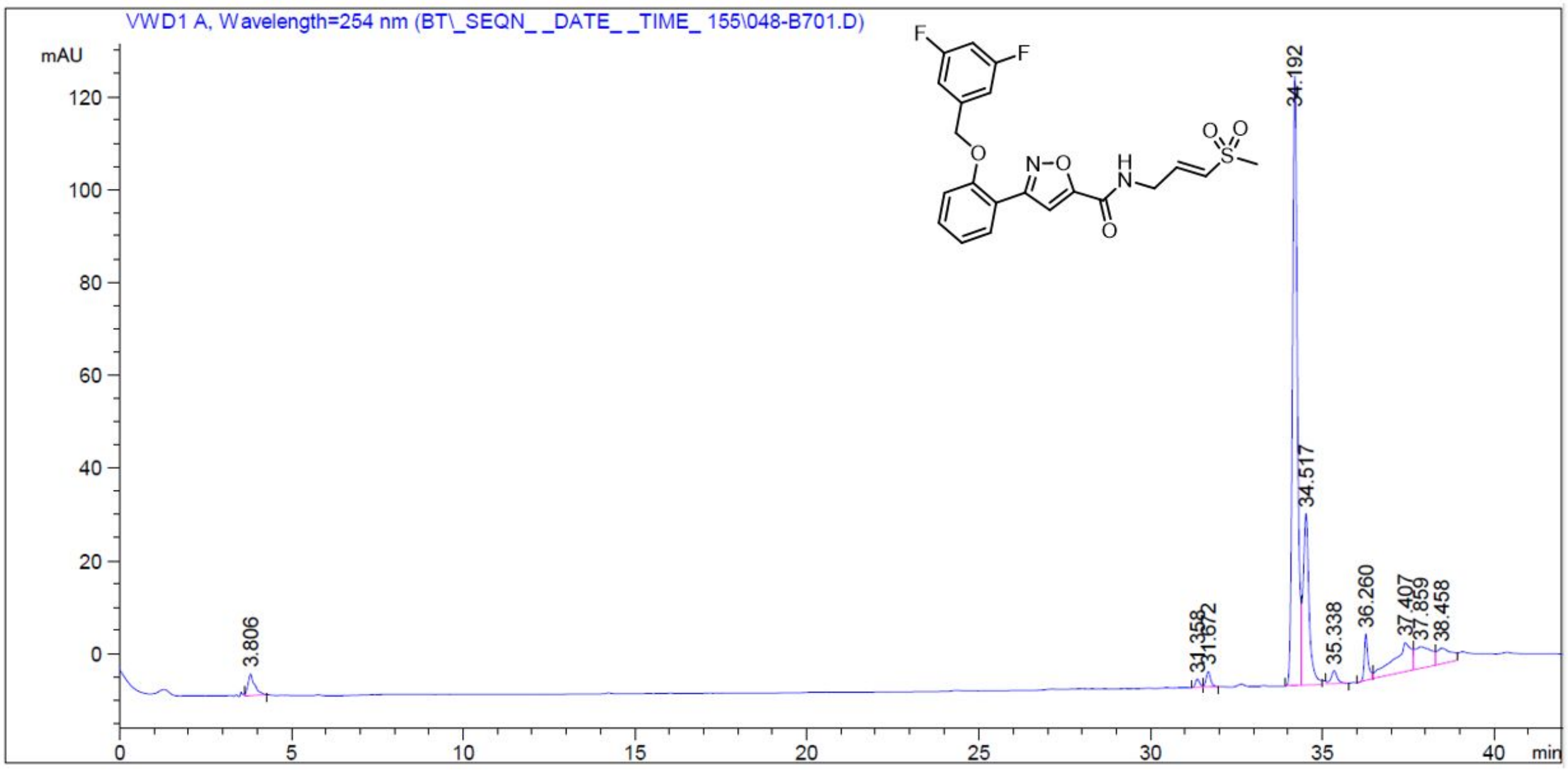

HPLC trace for Jun14364

8-p077-8s

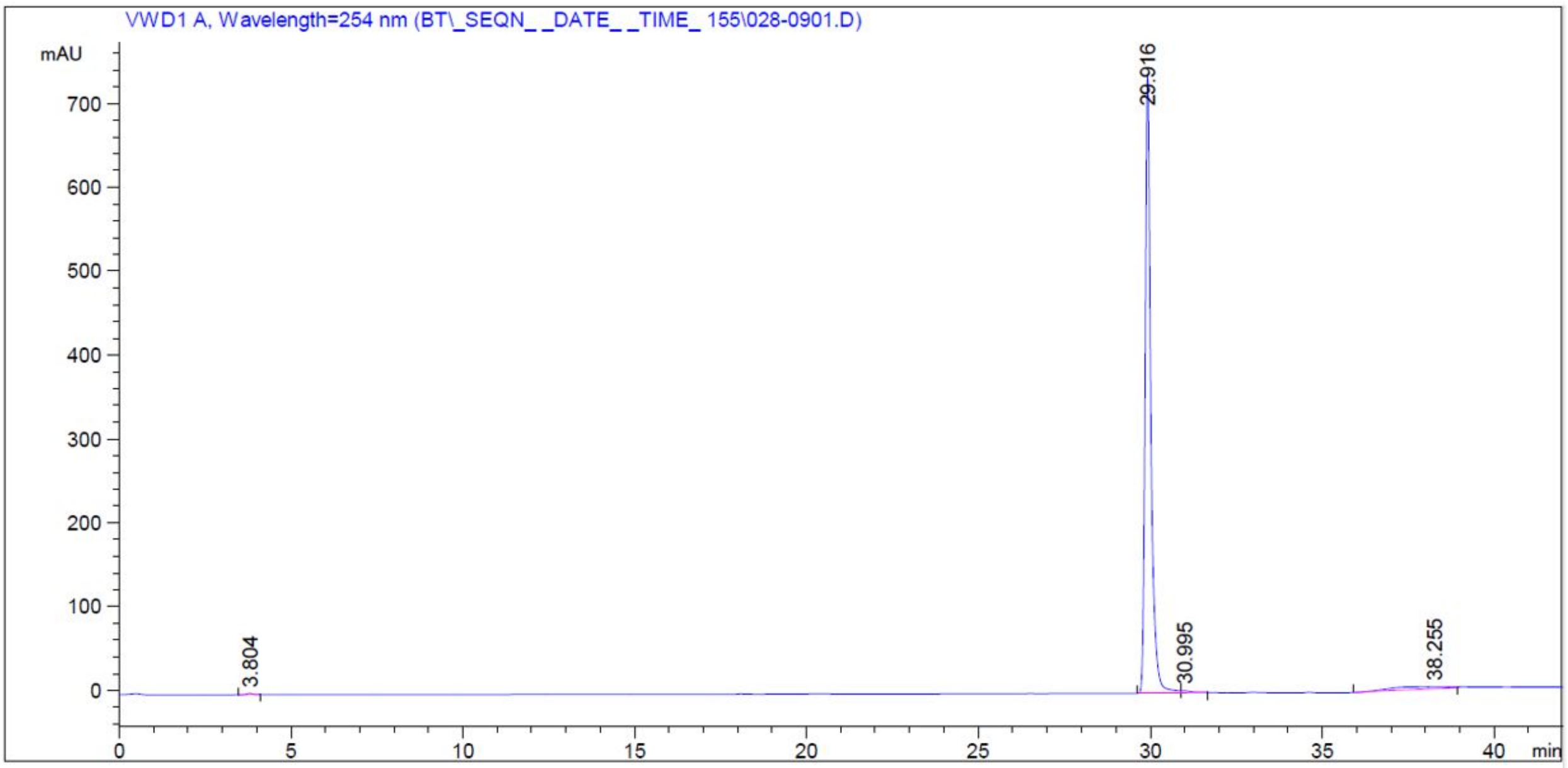

Jun14364 (zc8-p077-8a)#

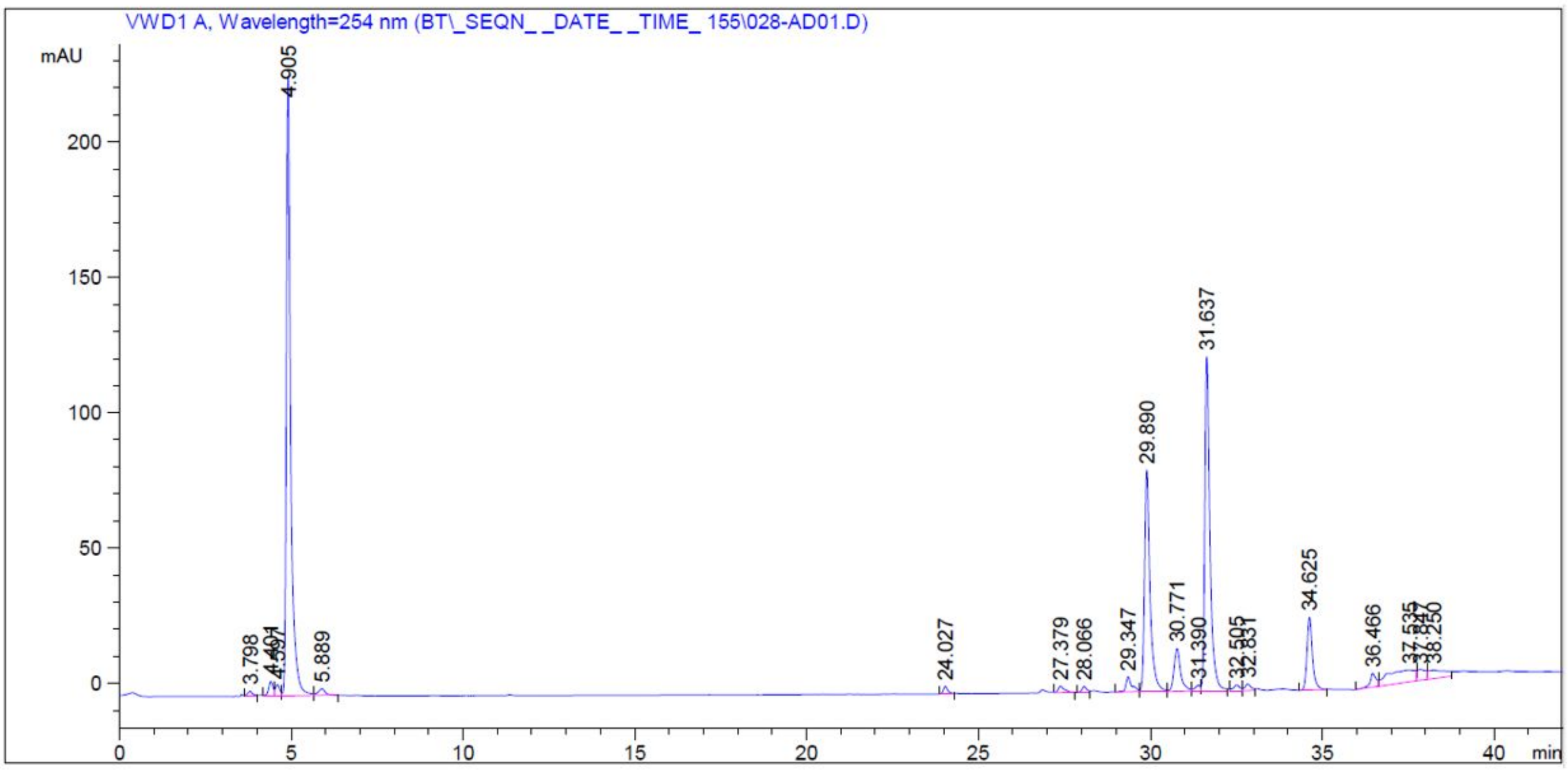

HPLC trace for Jun14435

8-p077-9s

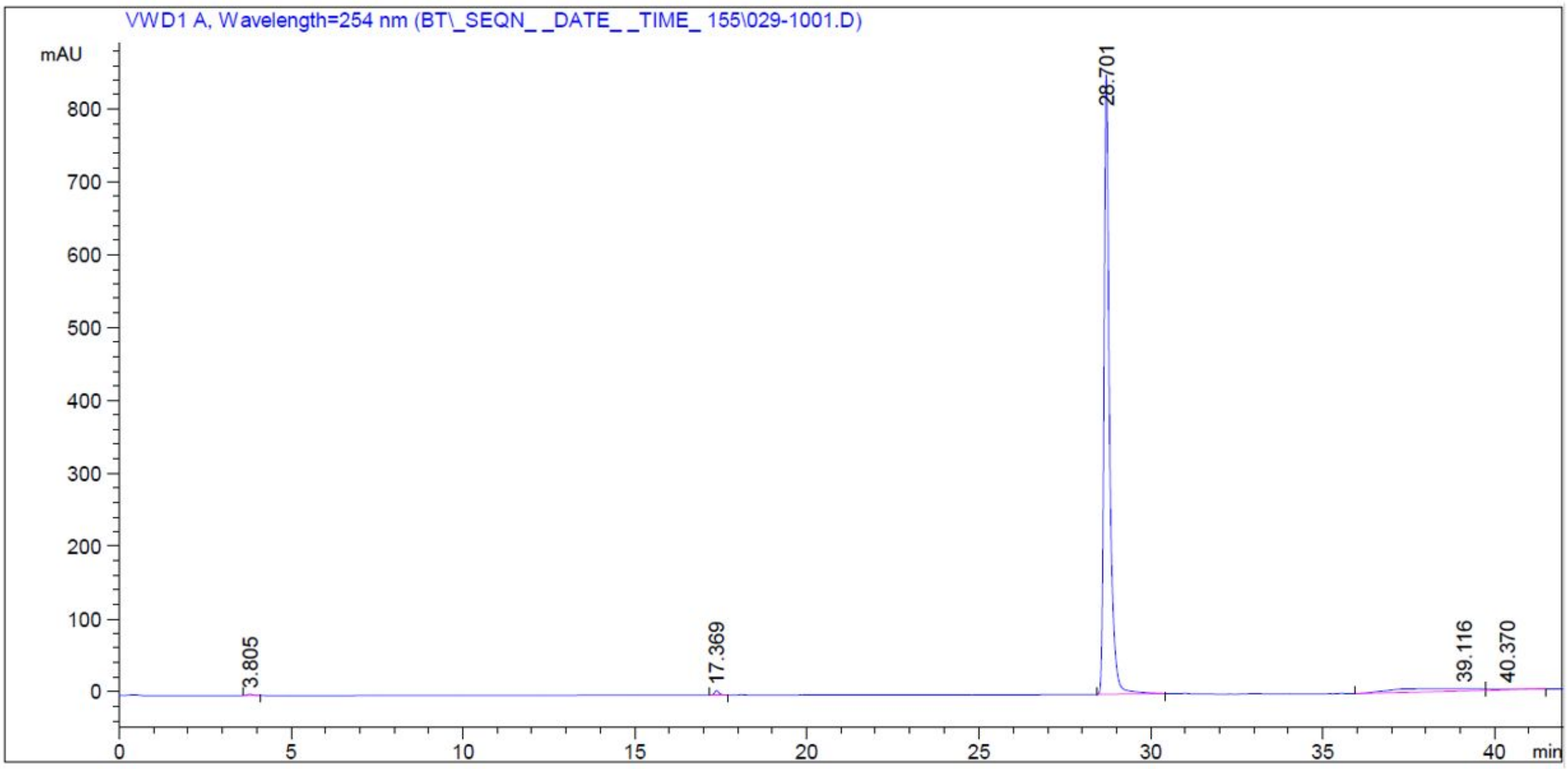

Jun14435 (zc8-p077-9)

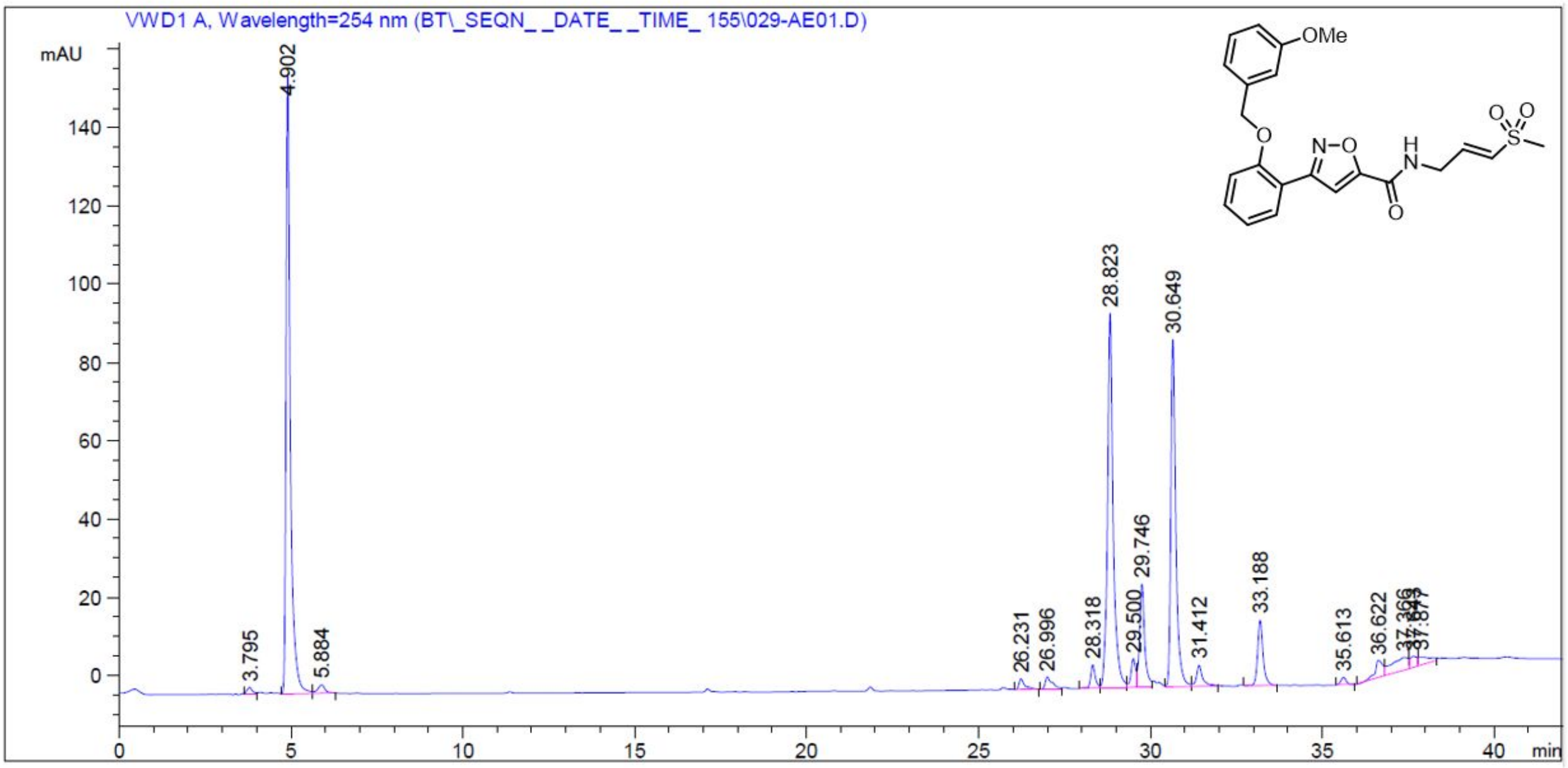

HPLC trace for Jun14454

8-p077-10s

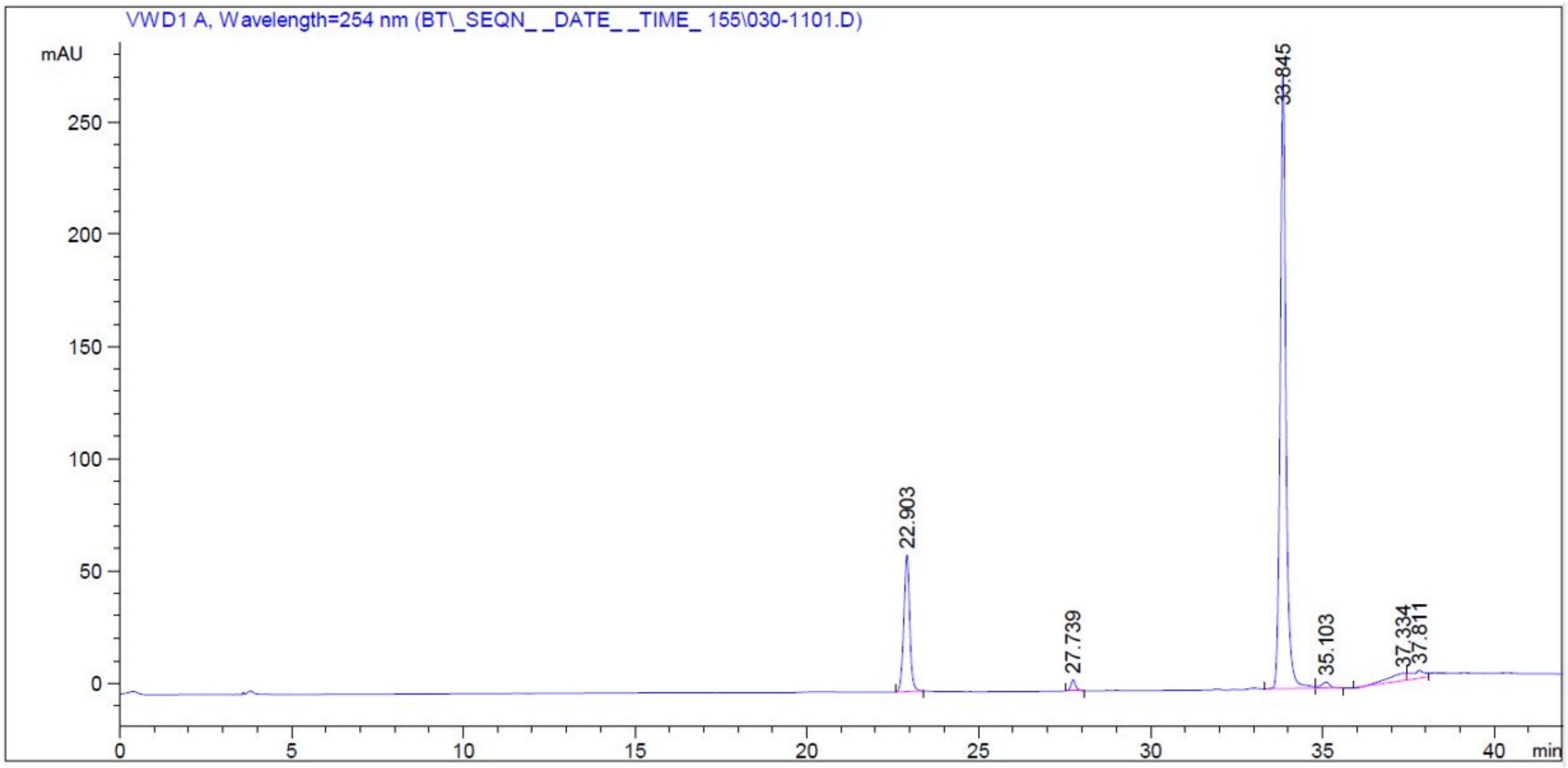

Jun14454 (zc8-p077-10)

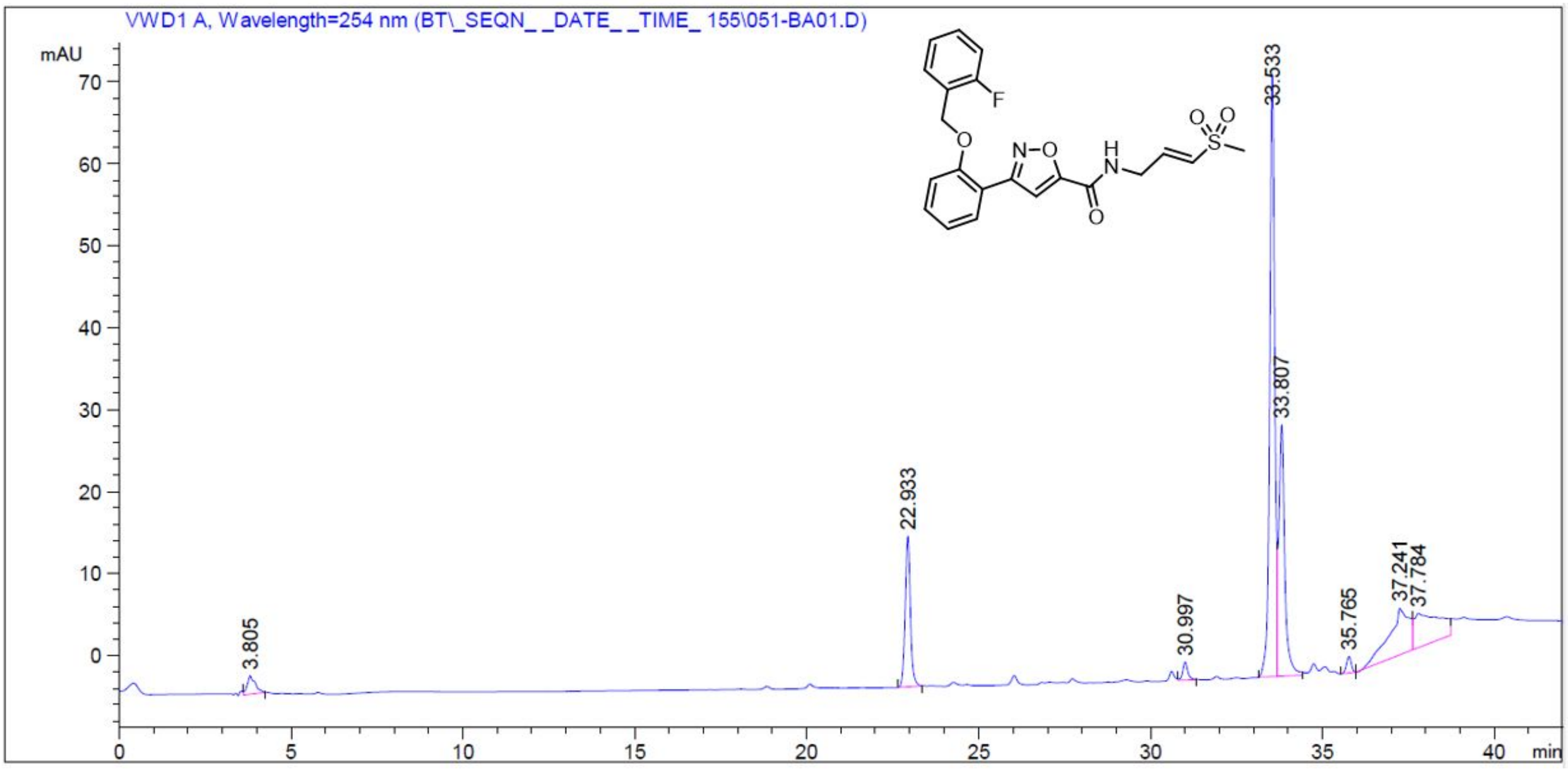

HPLC trace for Jun14482

8-p077-11s

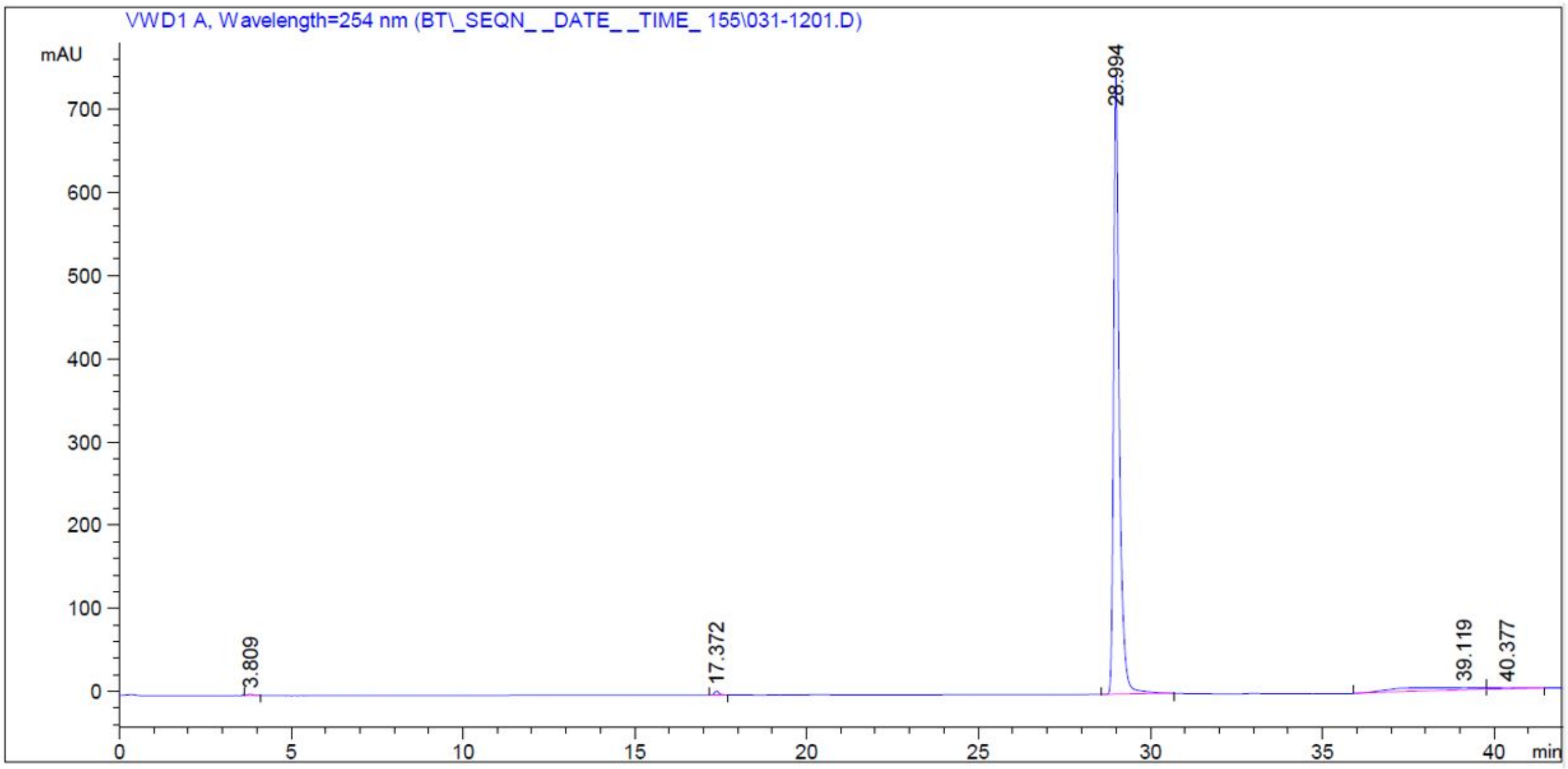

Jun4482 (zc8-p077-11)#

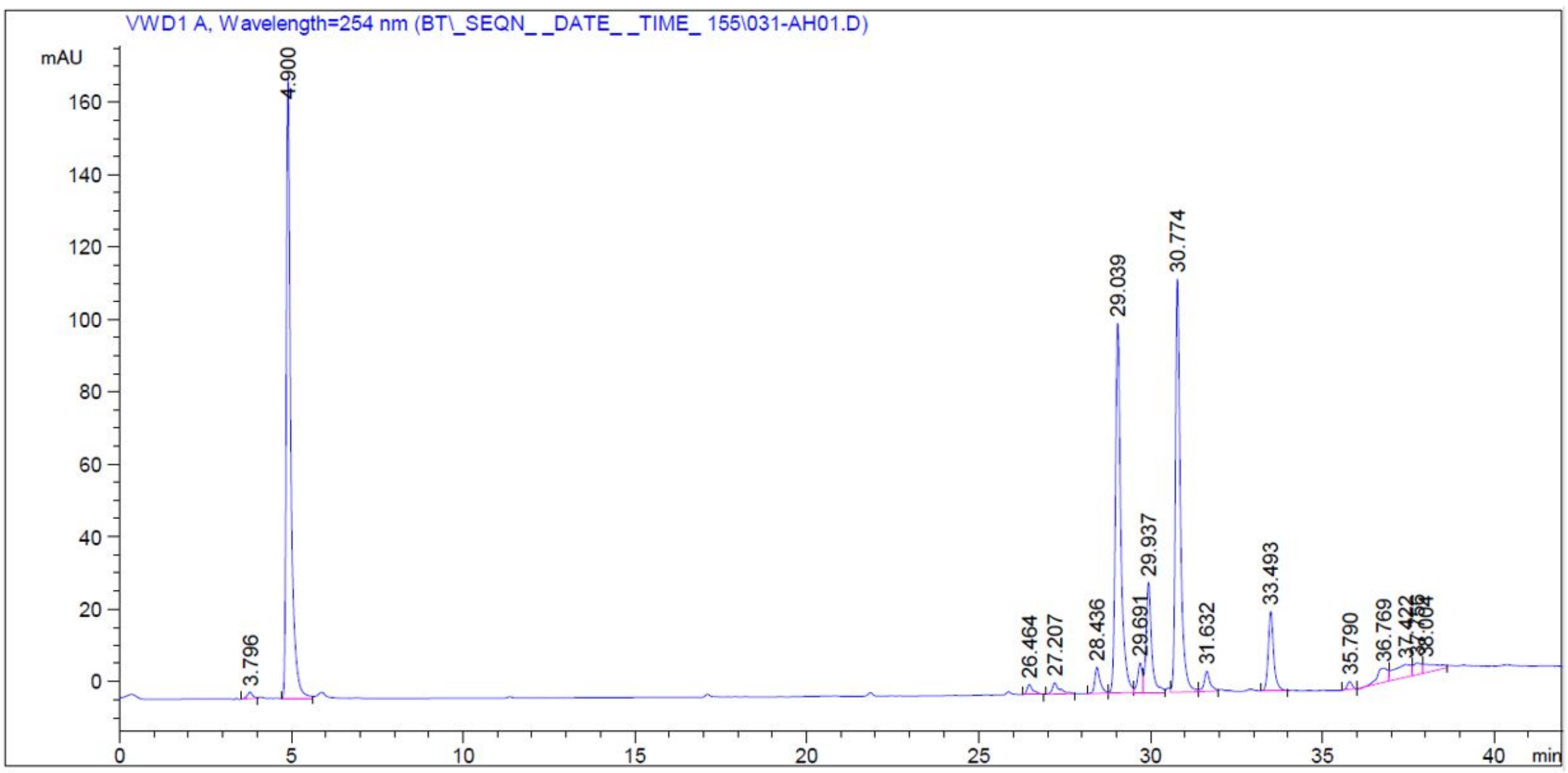

HPLC trace for Jun14693

8-p077-12s

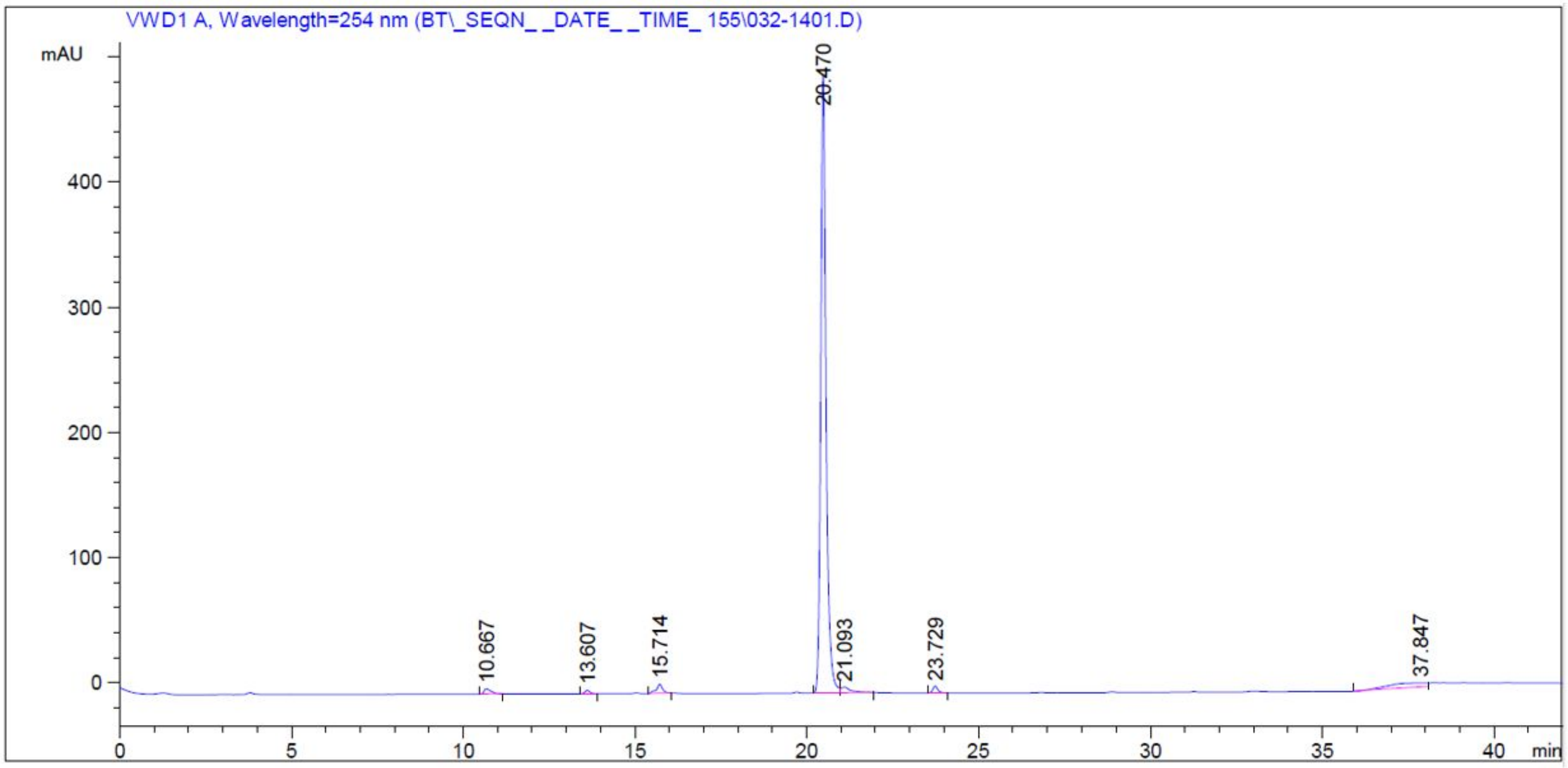

Jun14693 (zc8-p077-12)

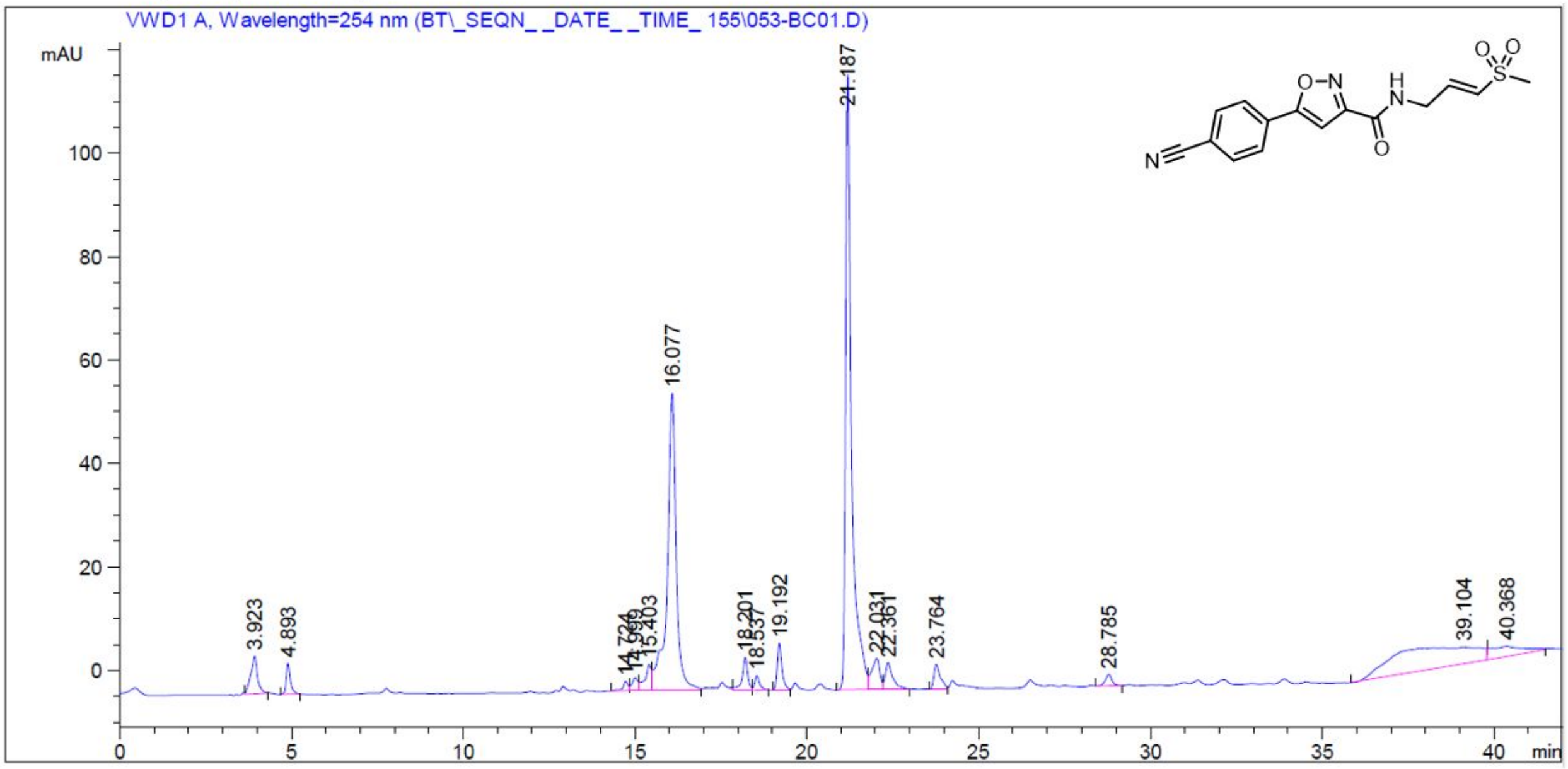

HPLC trace for Jun14694

8-p077-13s

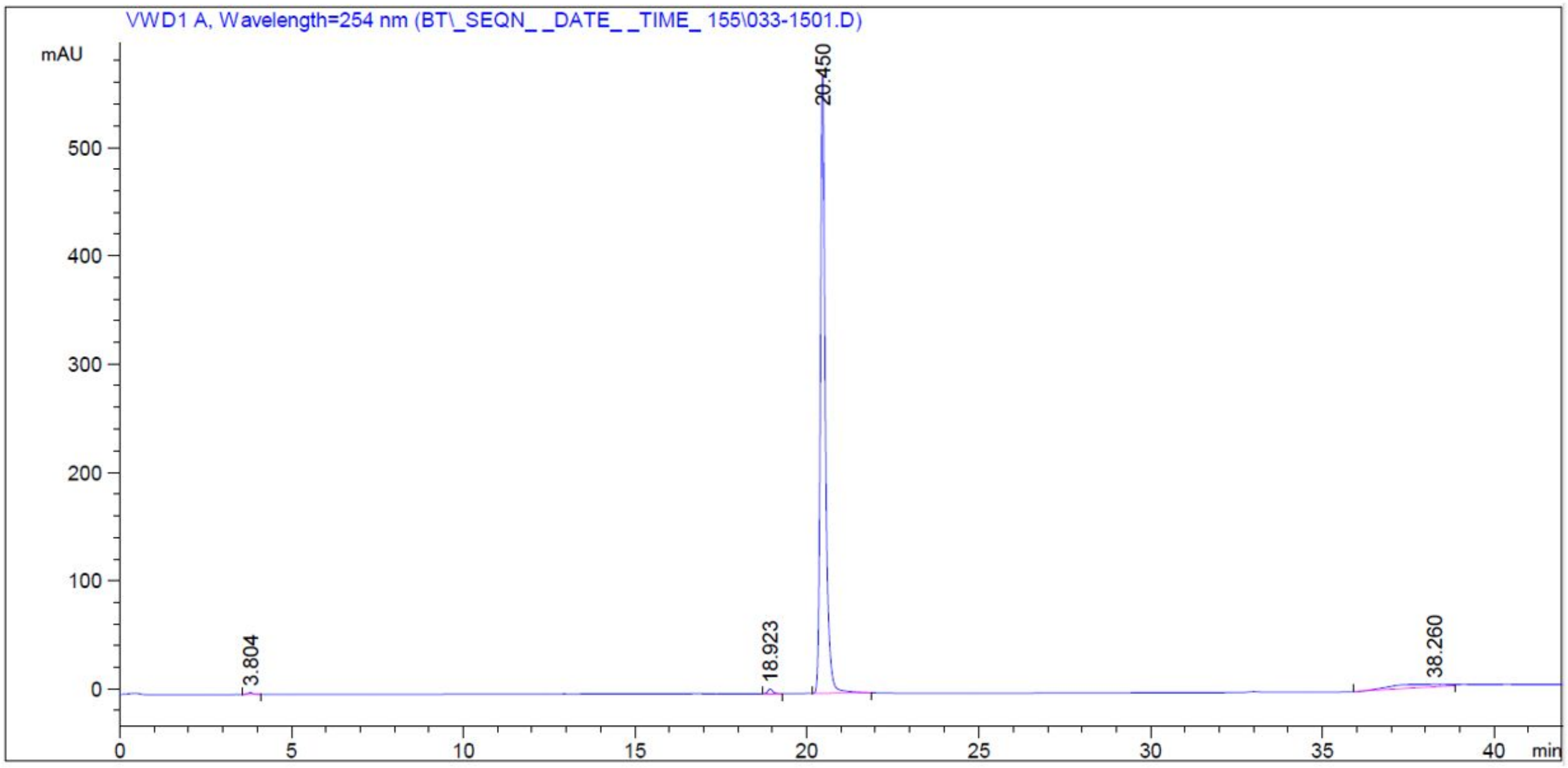

Jun14694 (zc8-p077-13)

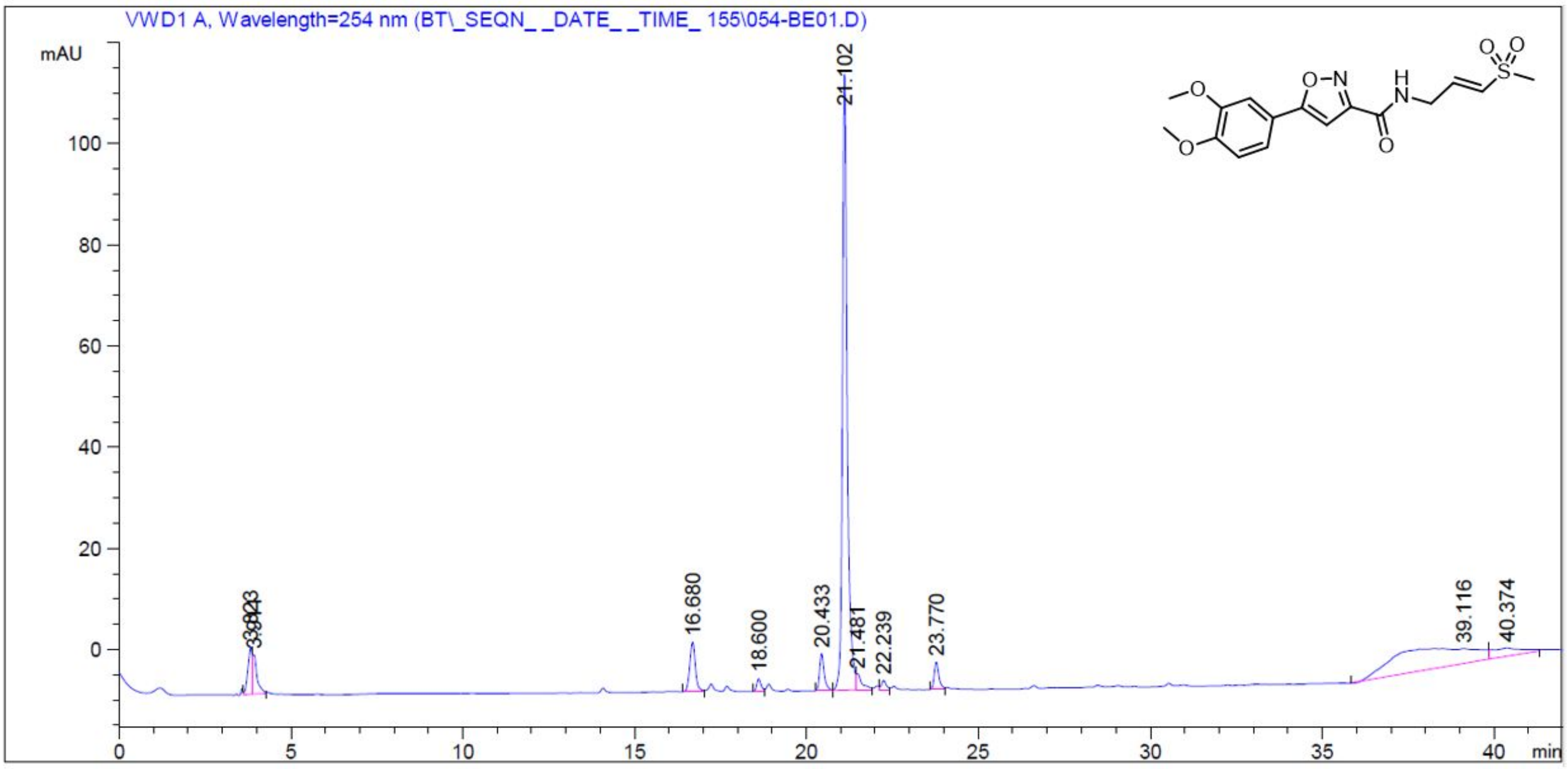

HPLC trace for Jun14778

8-p077-14s

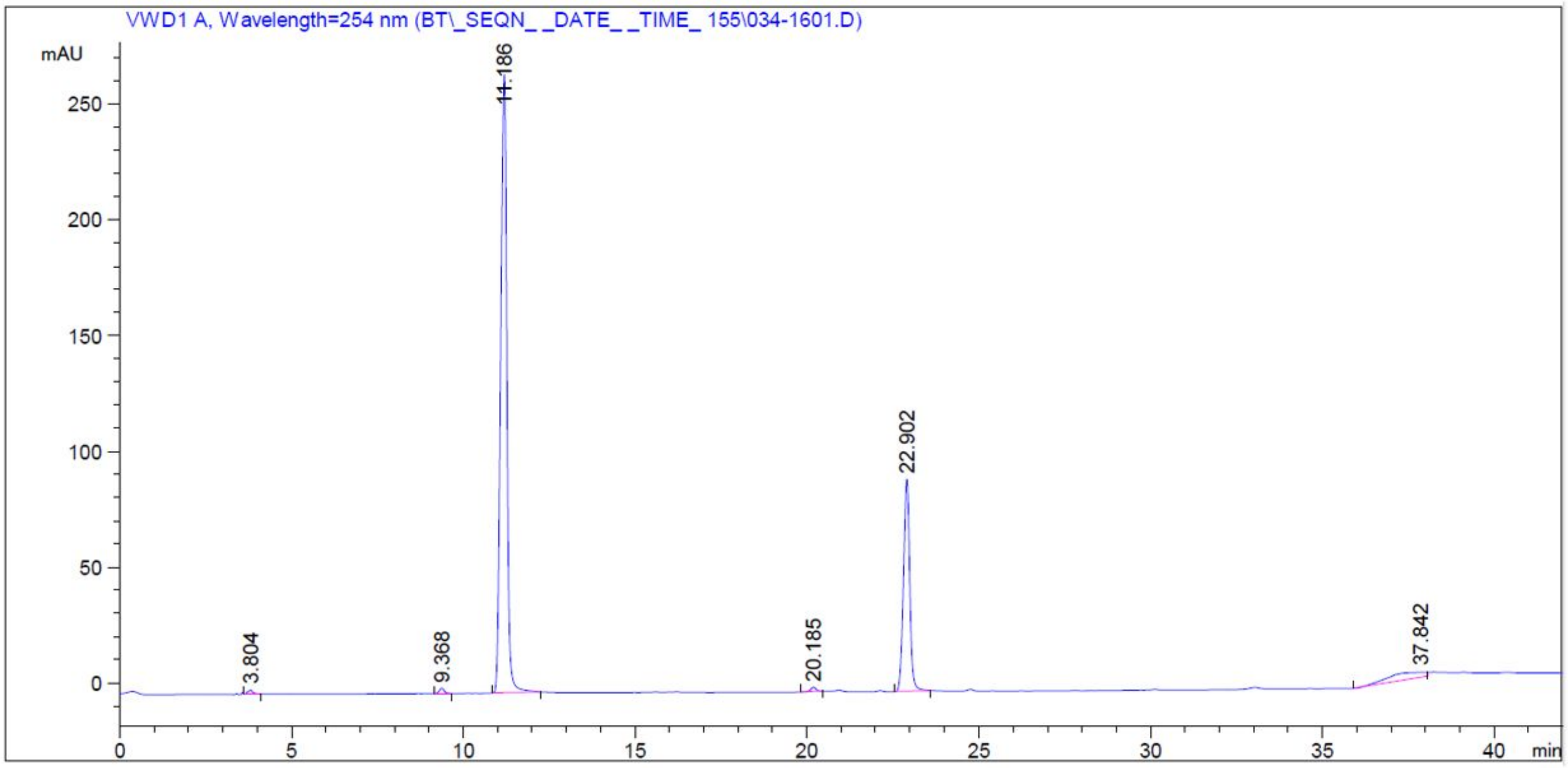

Jun14778 (zc8-p077-14)

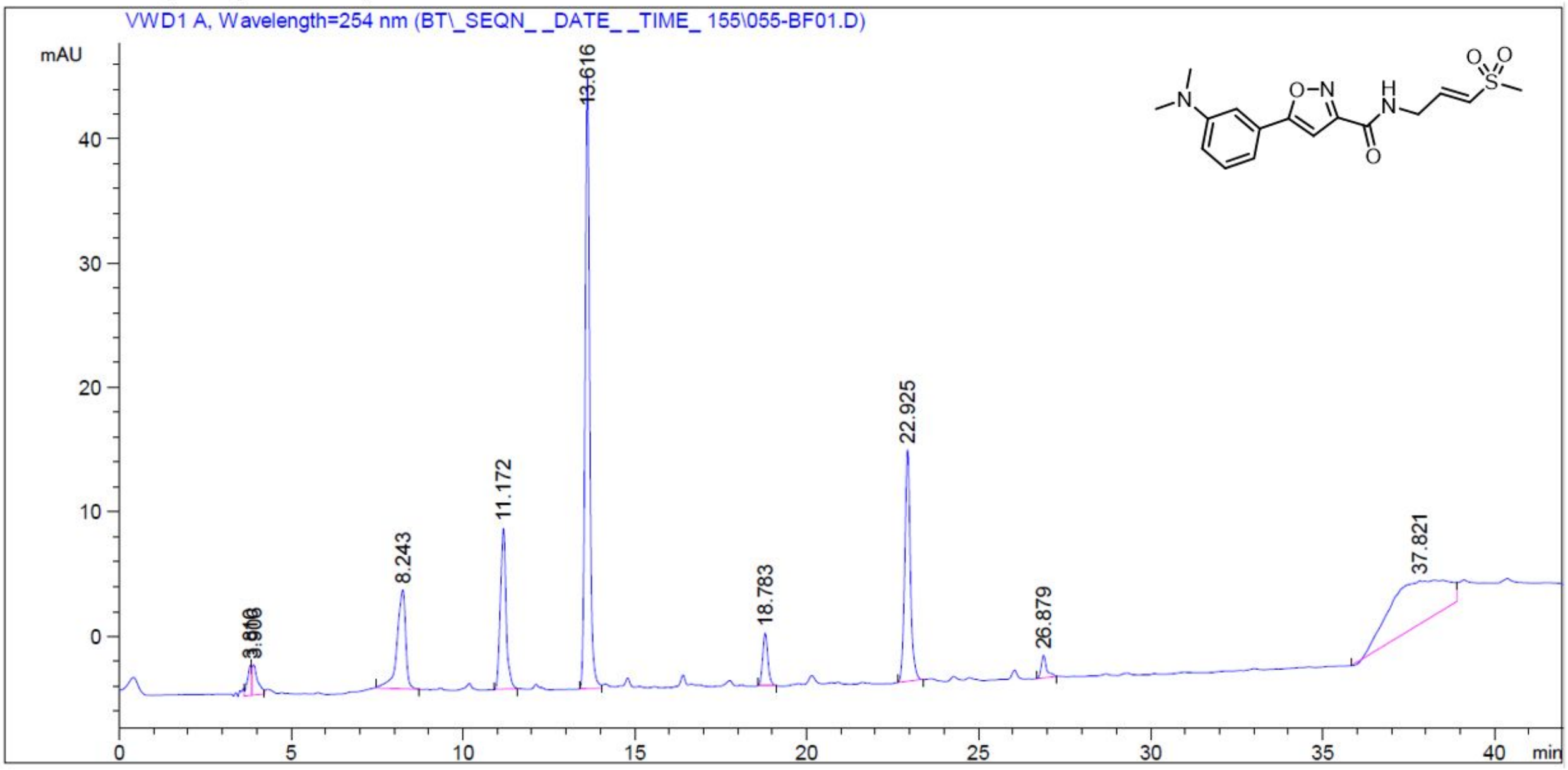

HPLC trace for Jun14782

8-p077-15s

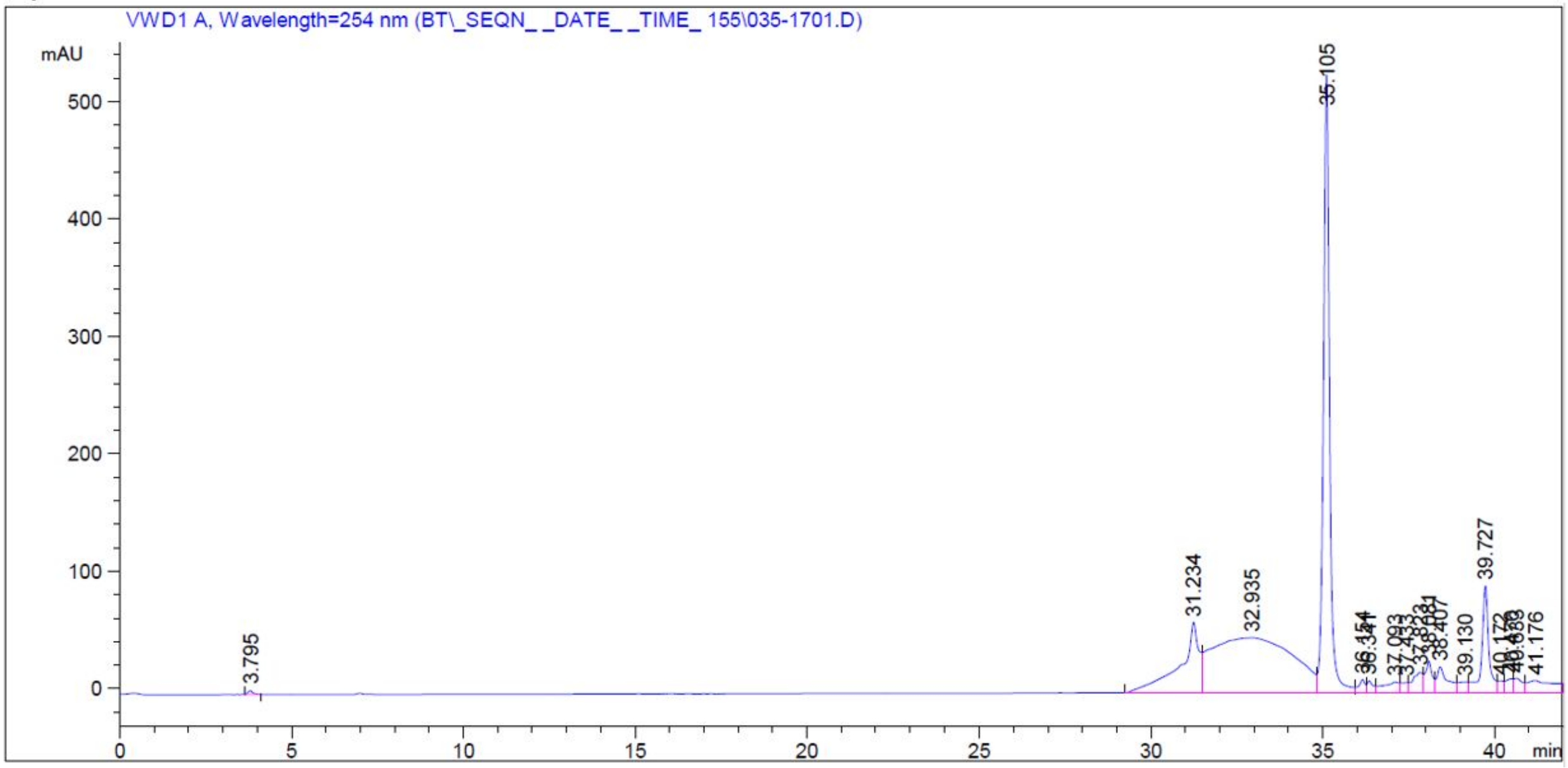

Jun14782 (zc8-p077-15)

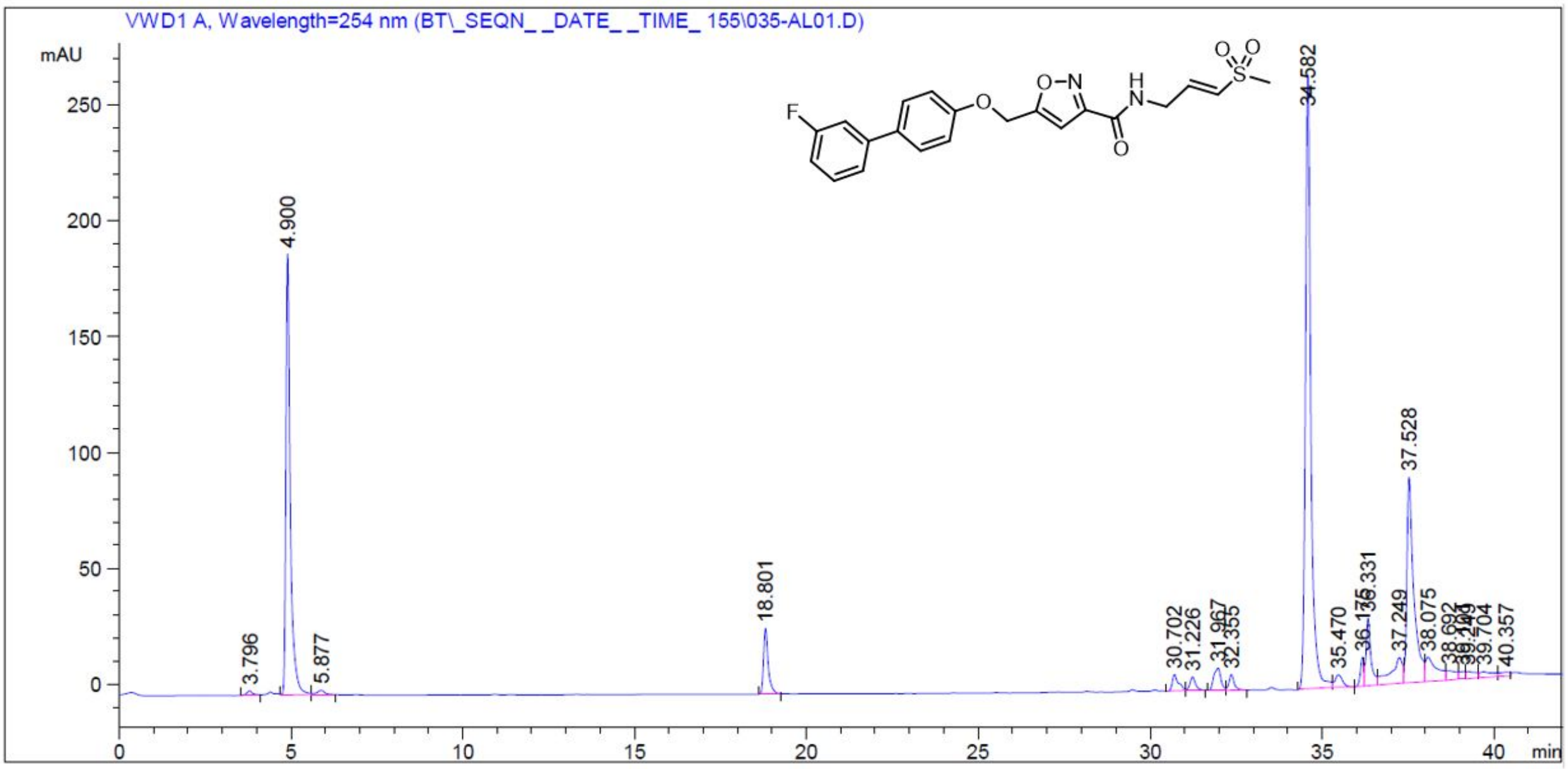

HPLC trace for Jun14852

8-p077-16s

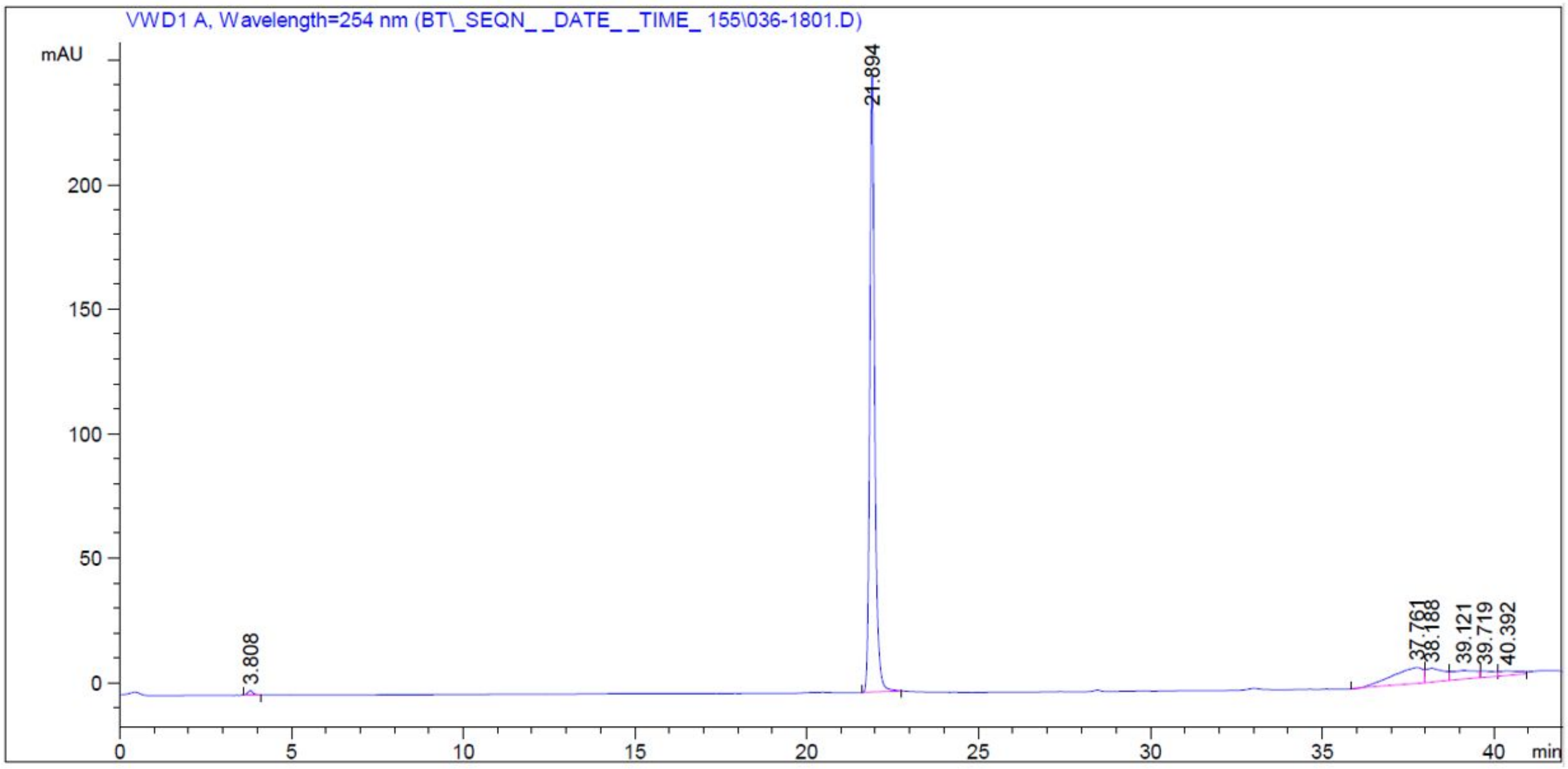

Jun14852 (zc8-p077-16)

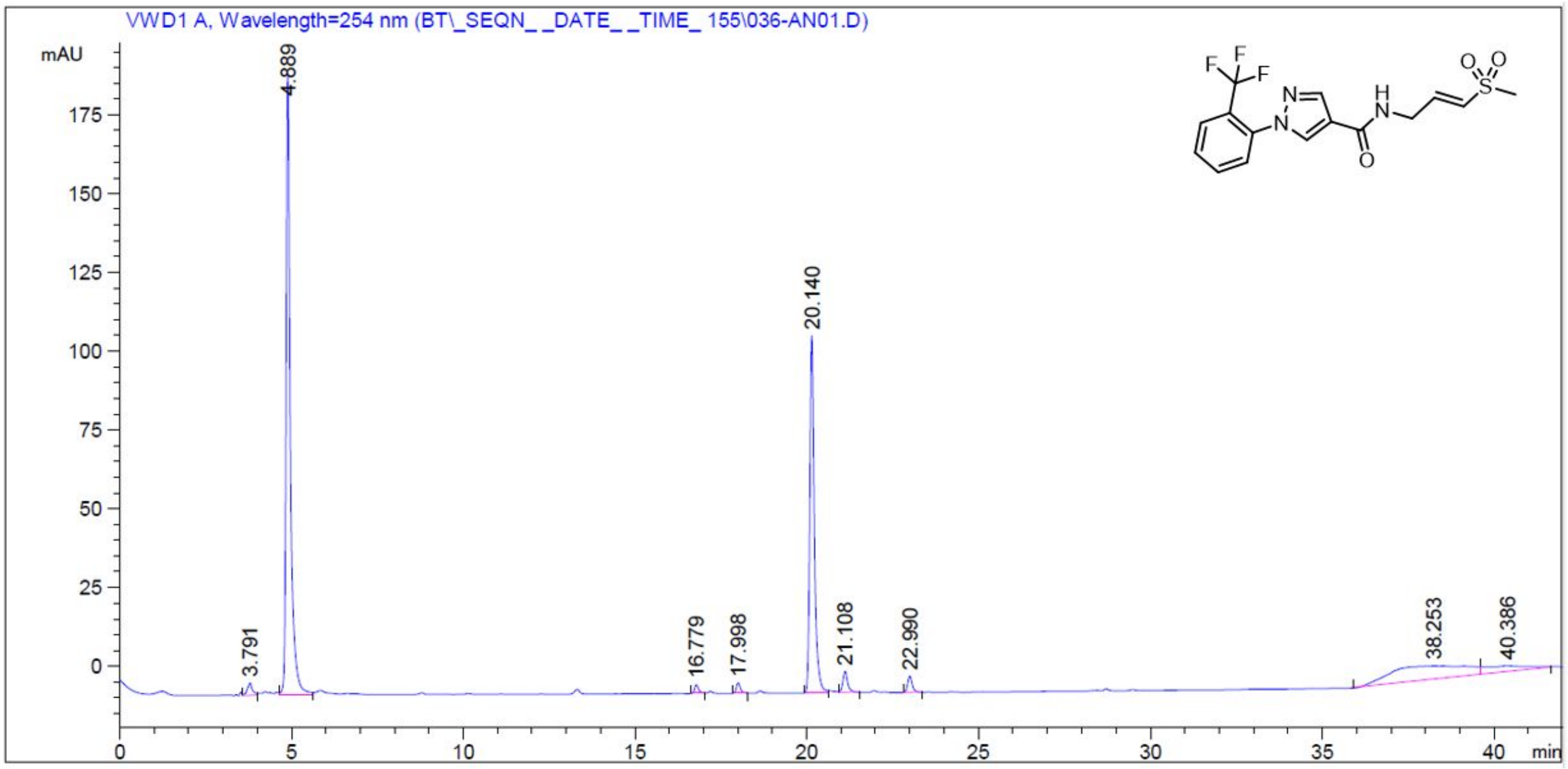

HPLC trace for Jun14742

8-p077-17s

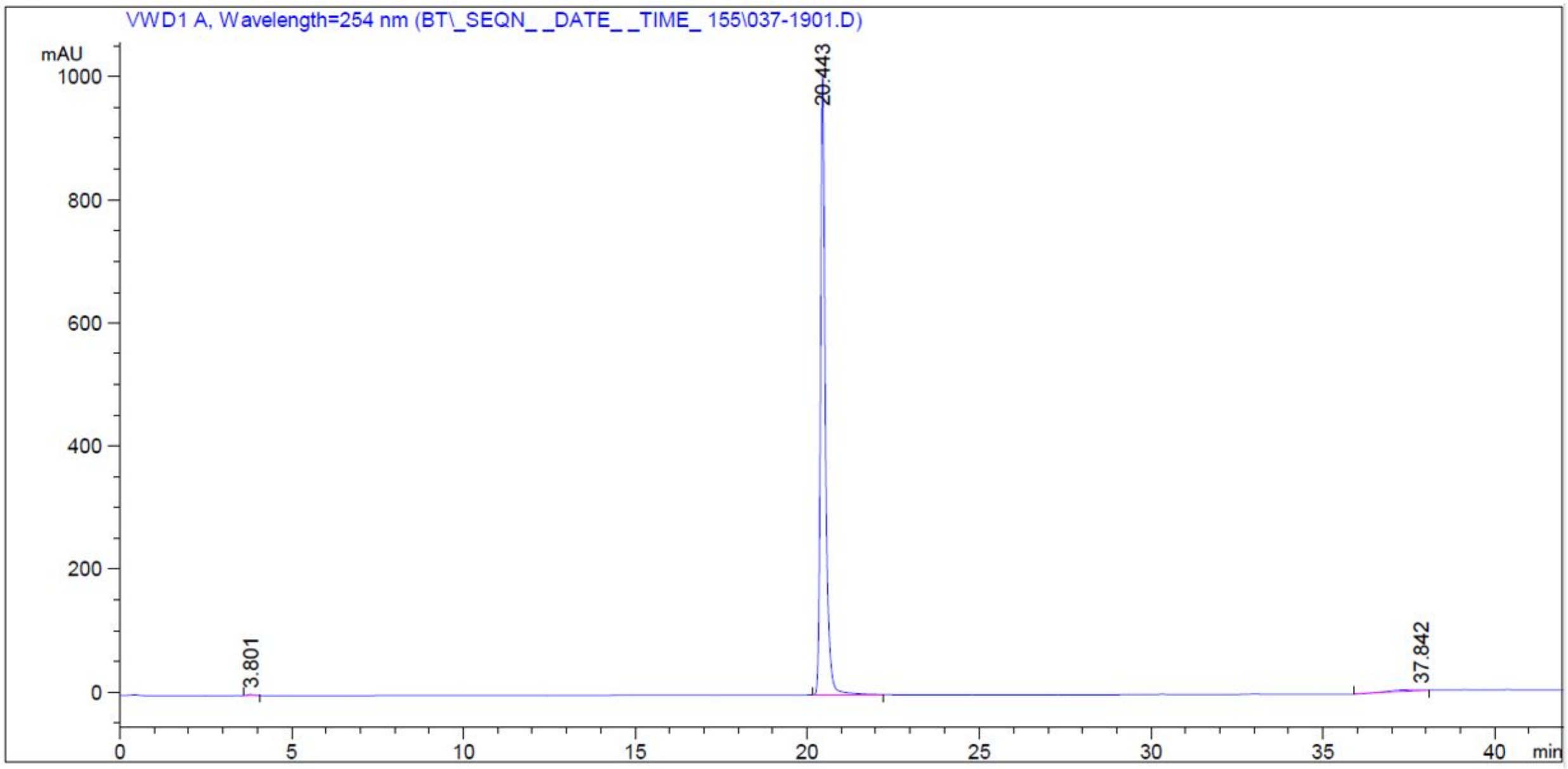

Jun14742 (zc8-p077-17)

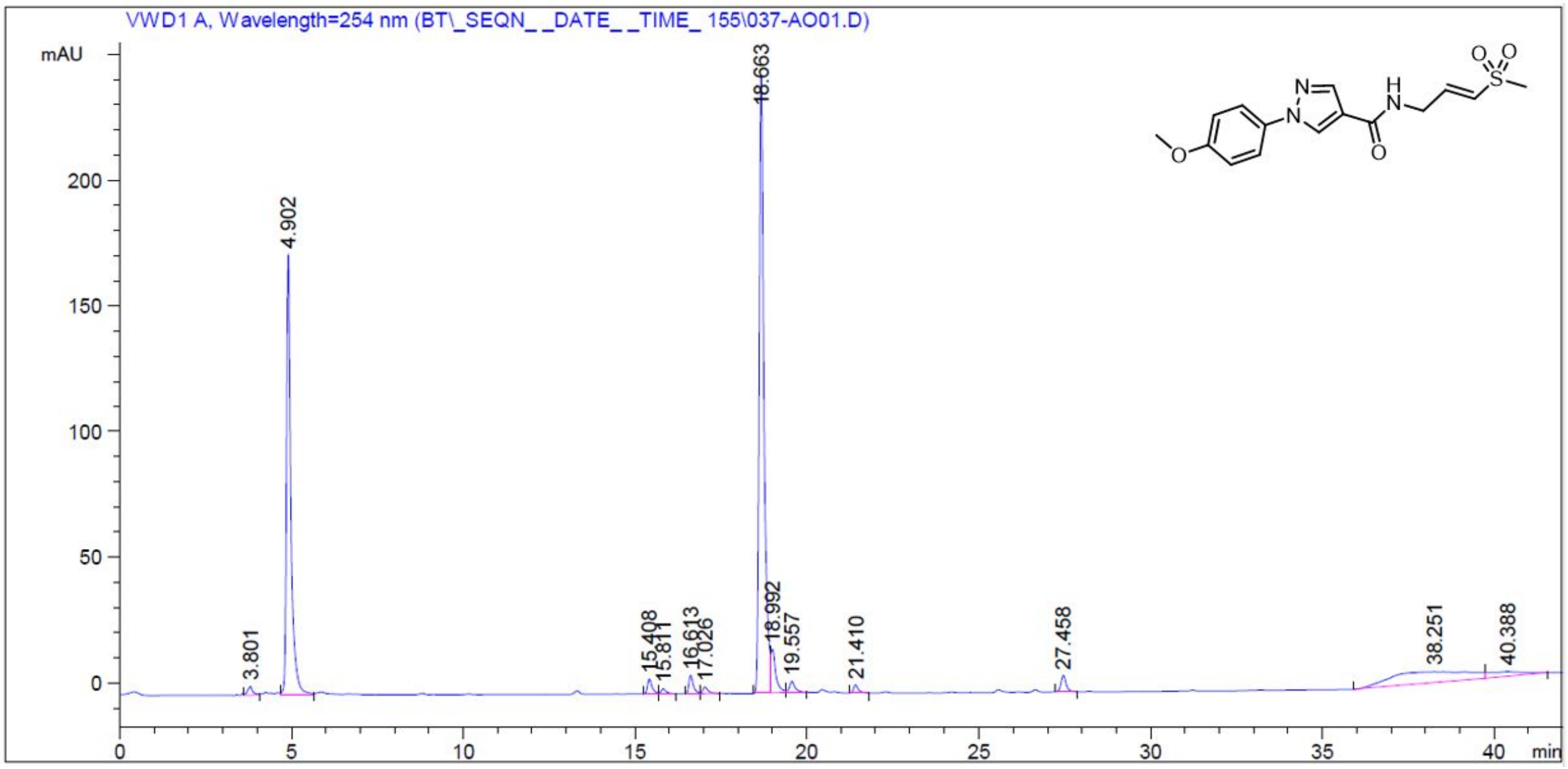

HPLC trace for Jun14855

8-p077-18s

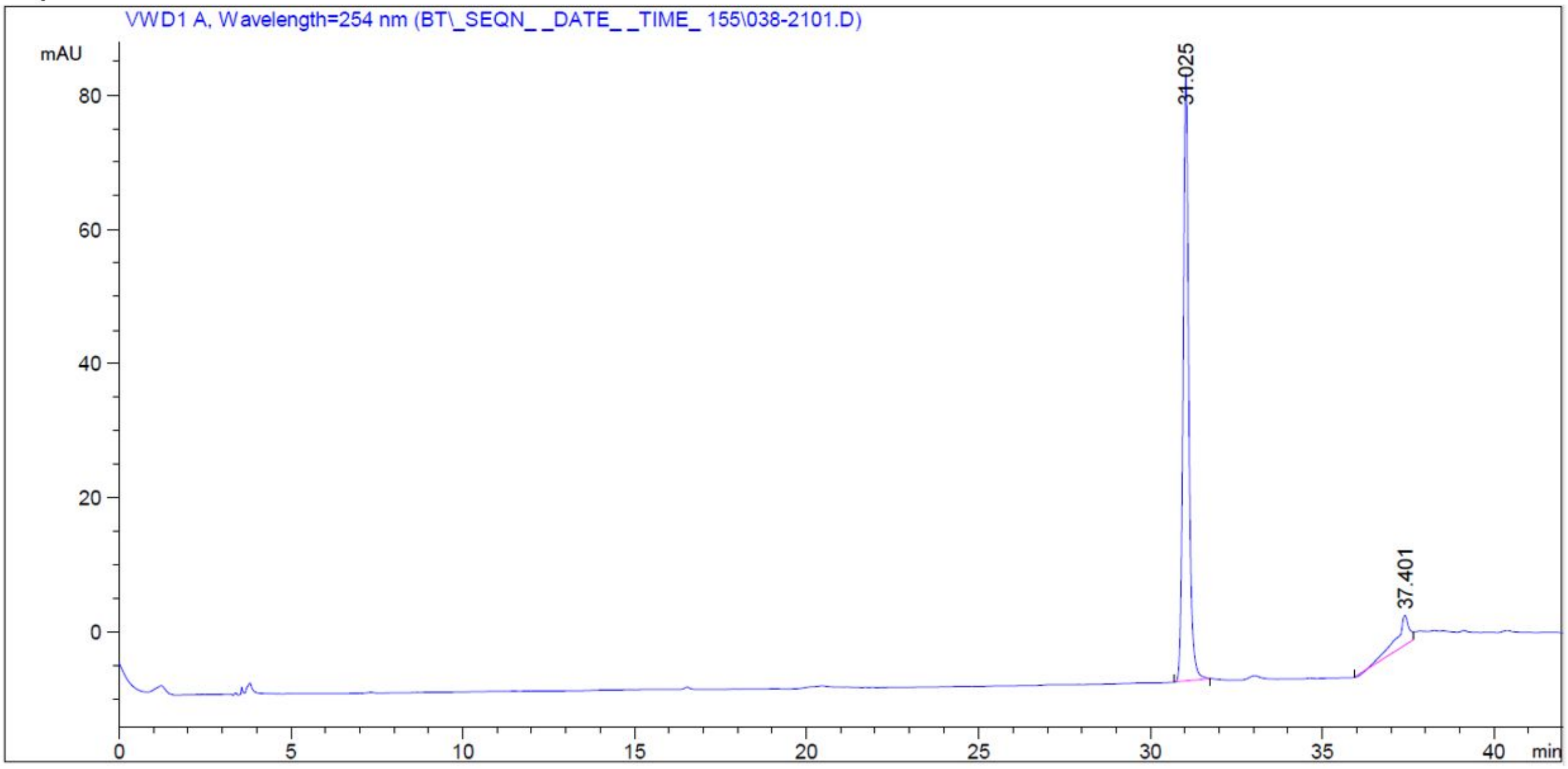

Jun14855 (zc8-p077-18)

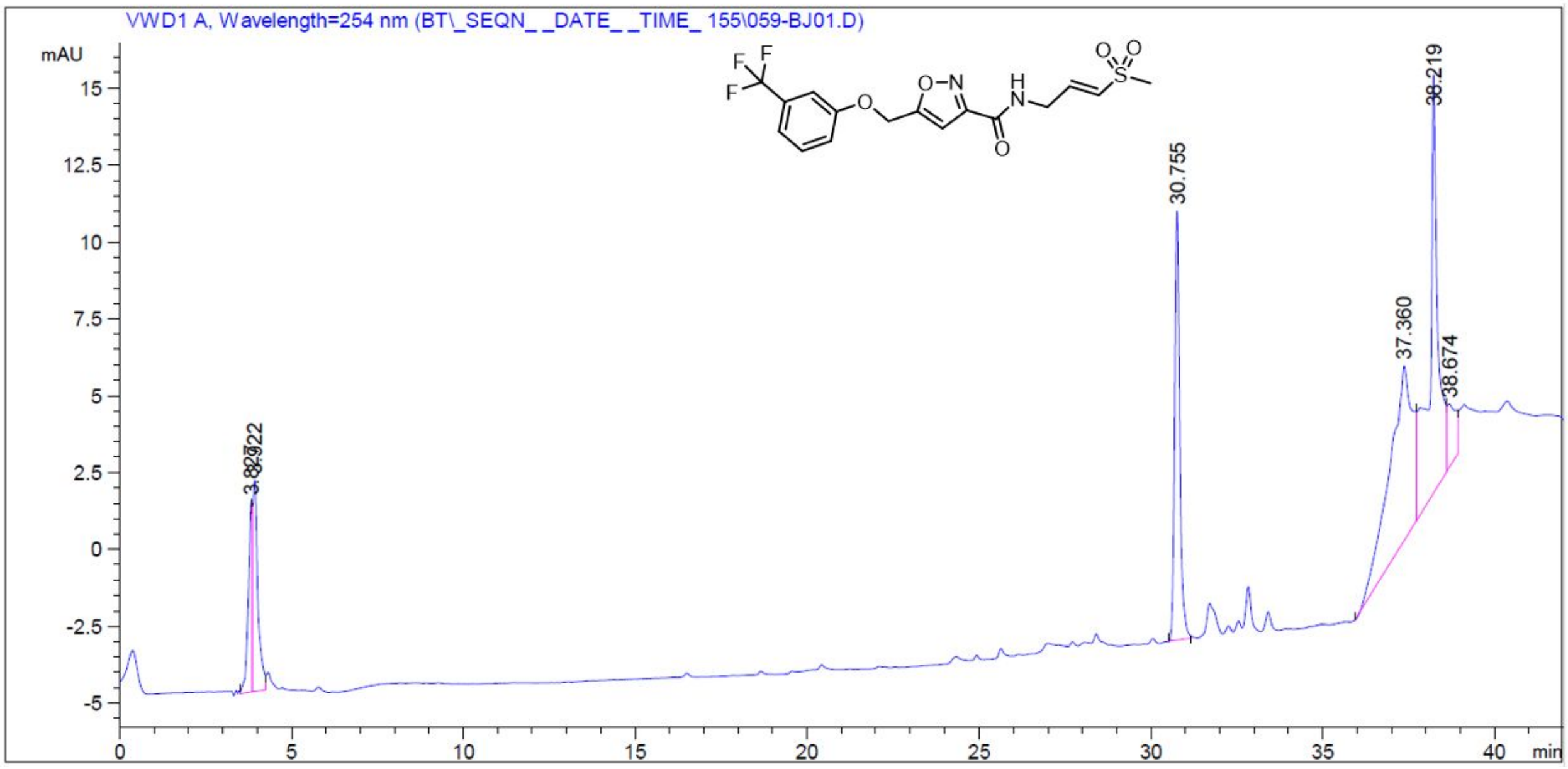

HPLC trace for Jun14861

8-p077-19s

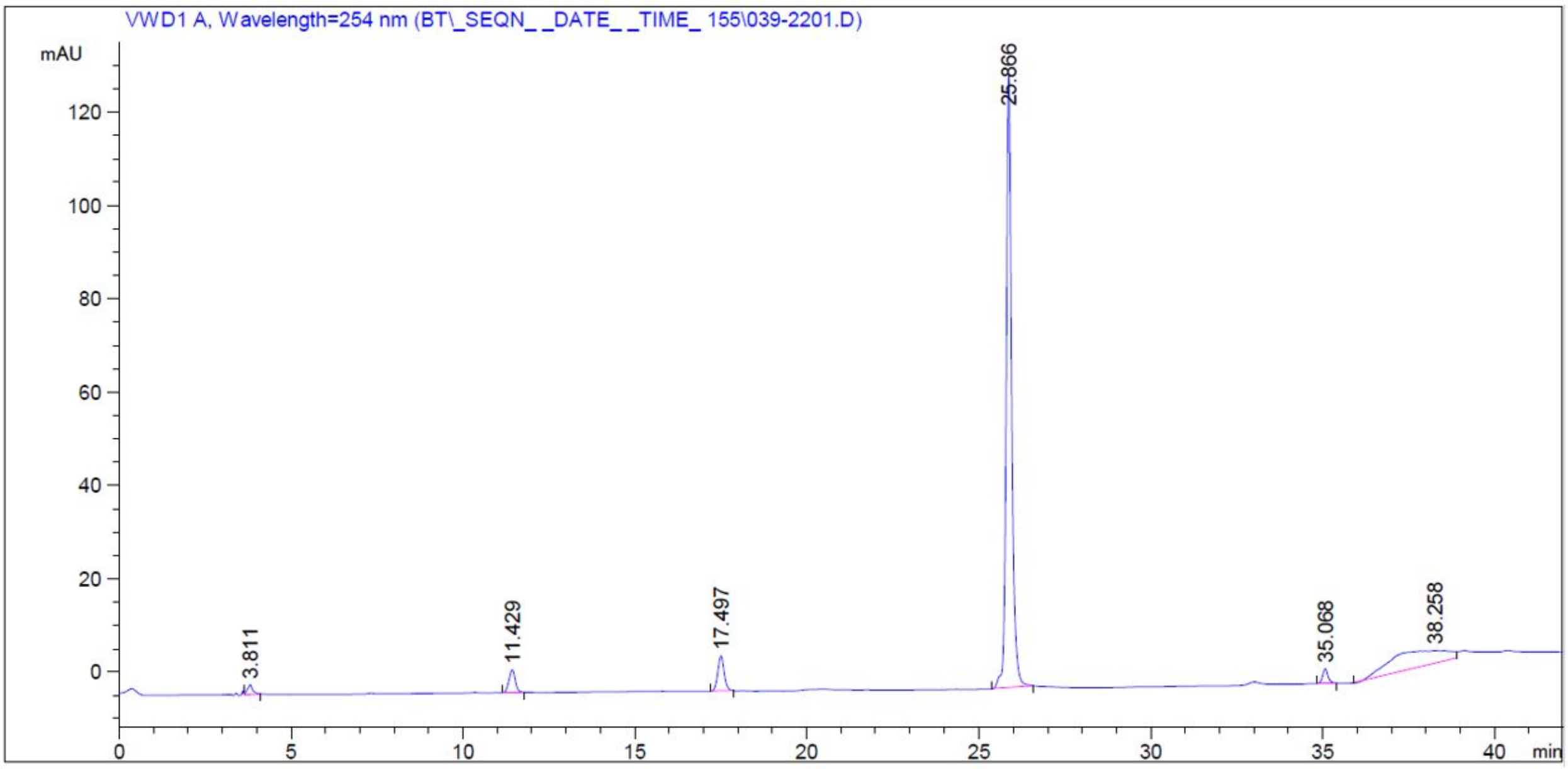

Jun14861 (zc8-p077-19)

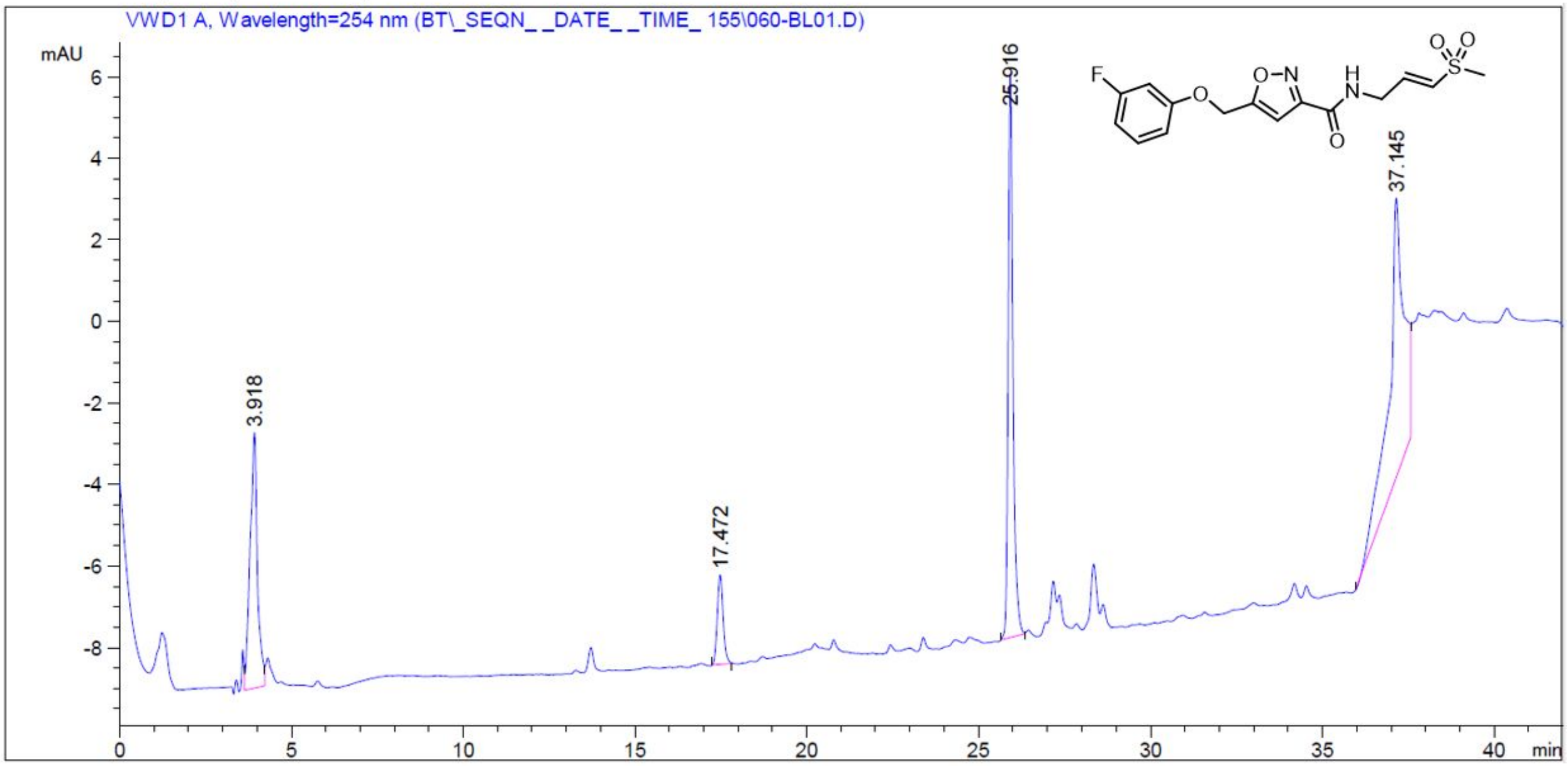

HPLC trace for Jun14856

8-p077-20s

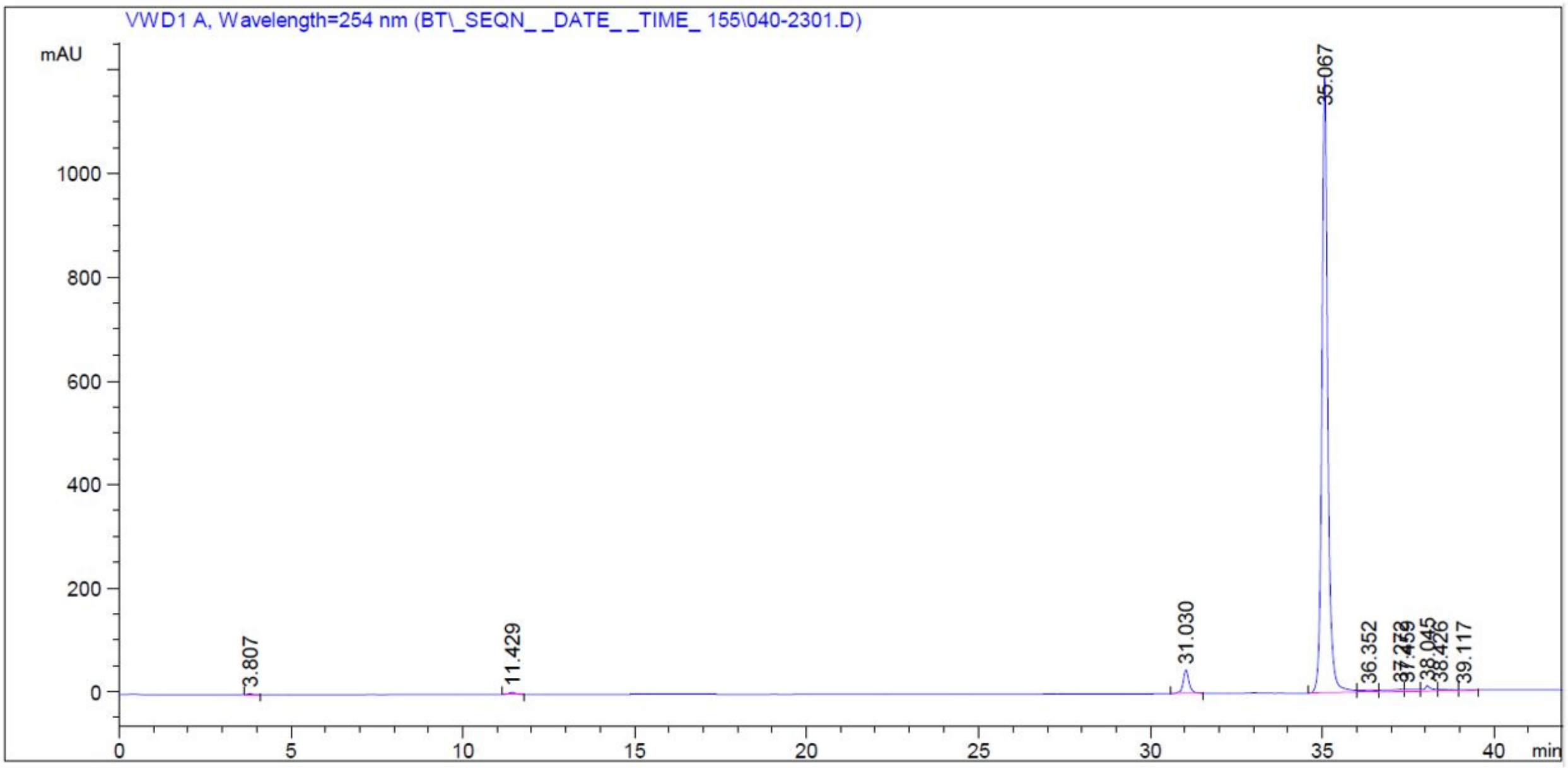

Jun14856 (zc8-p077-20)

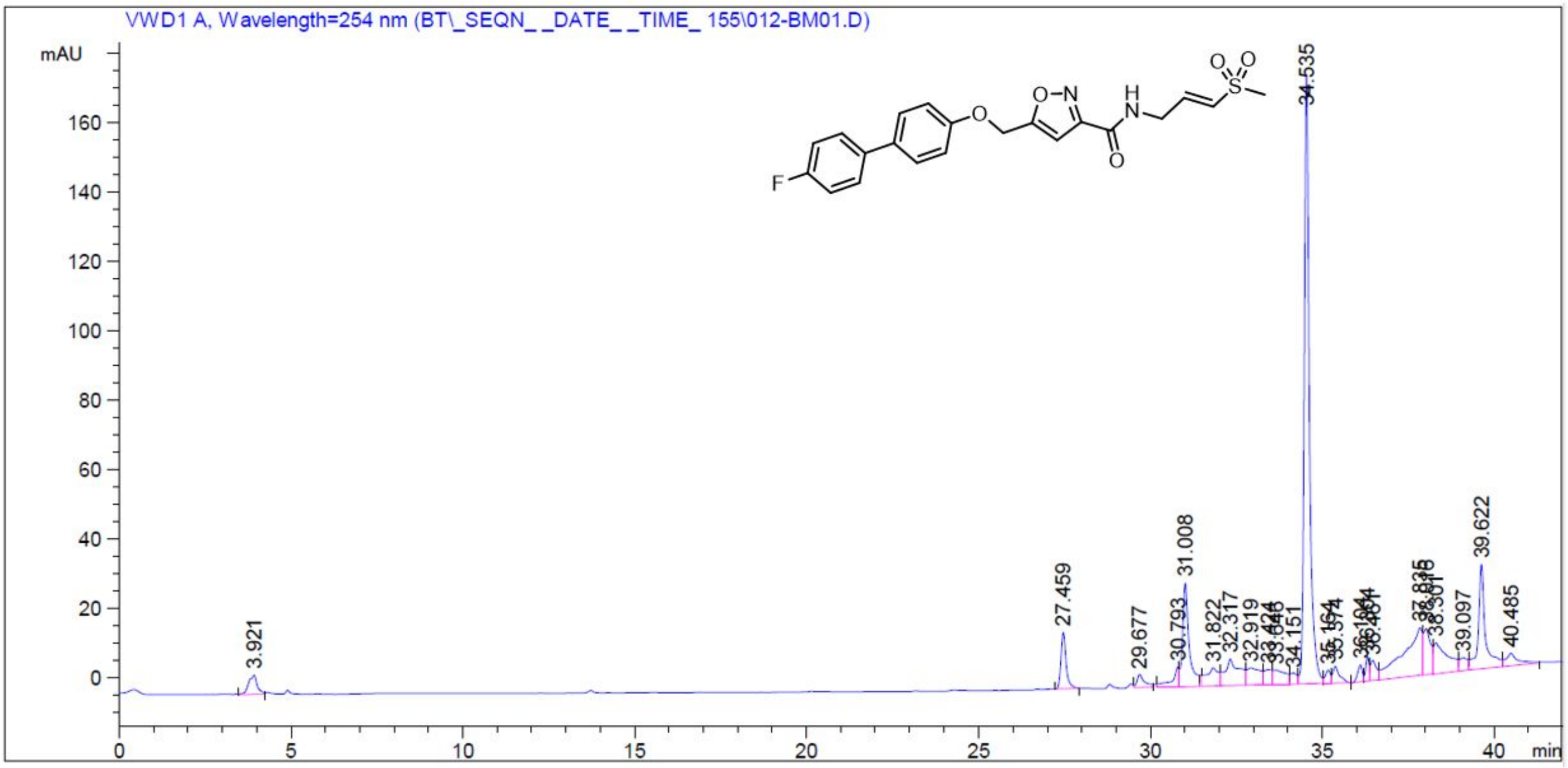

HPLC trace for Jun14458

8-p077-21s

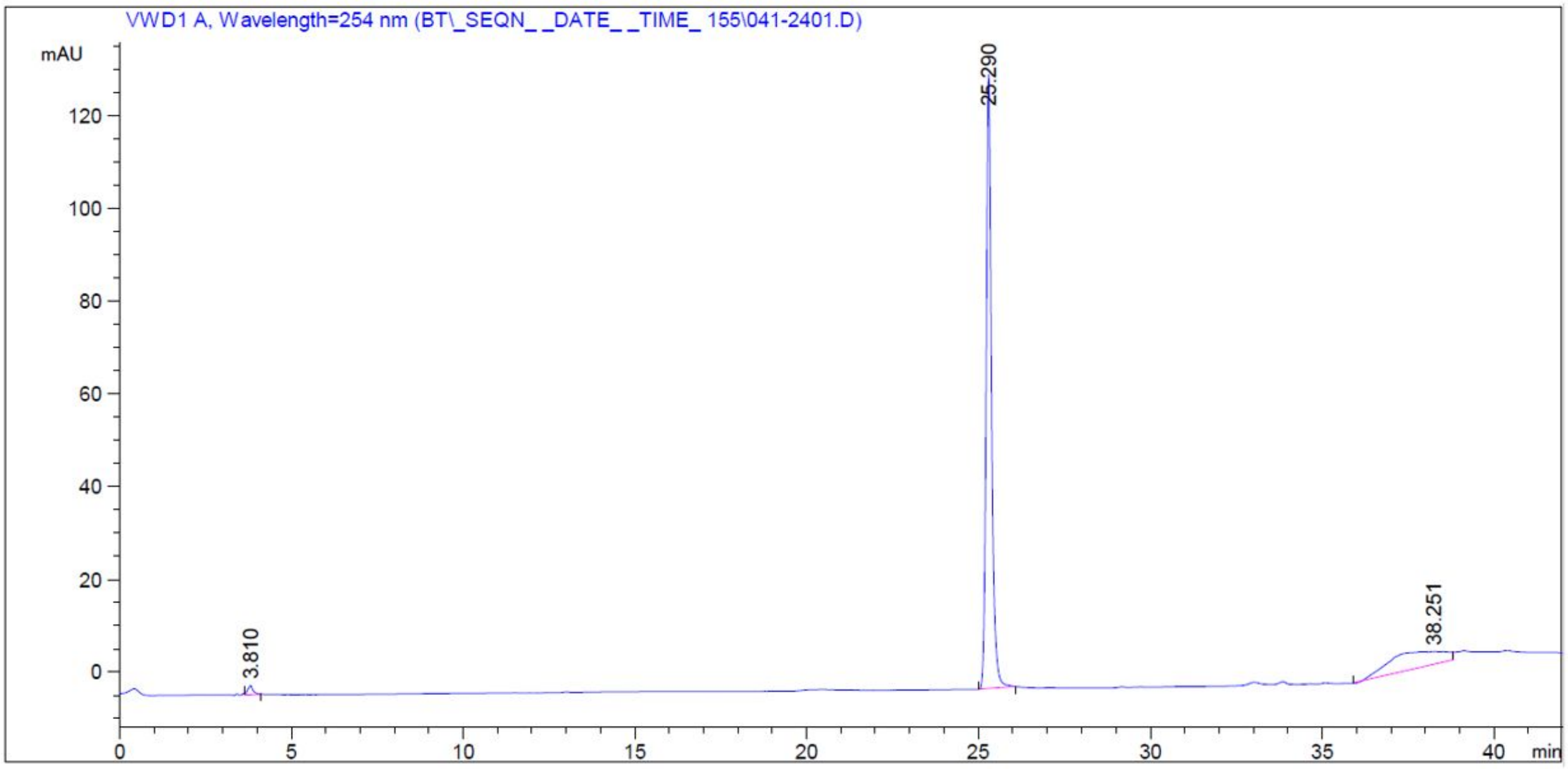

Jun14458 (zc8-p077-21)

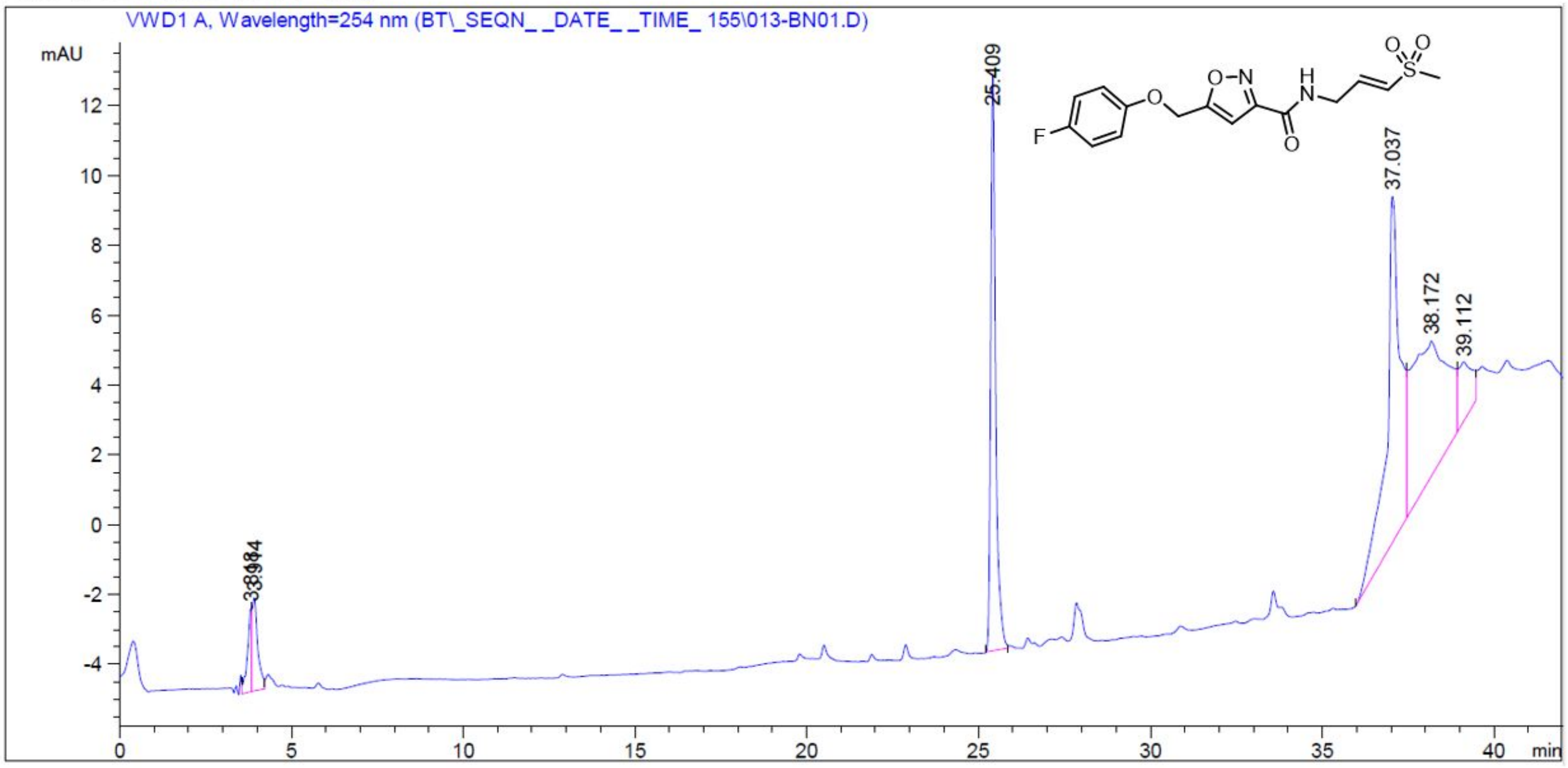

HPLC trace for Jun14744

8-p077-22s

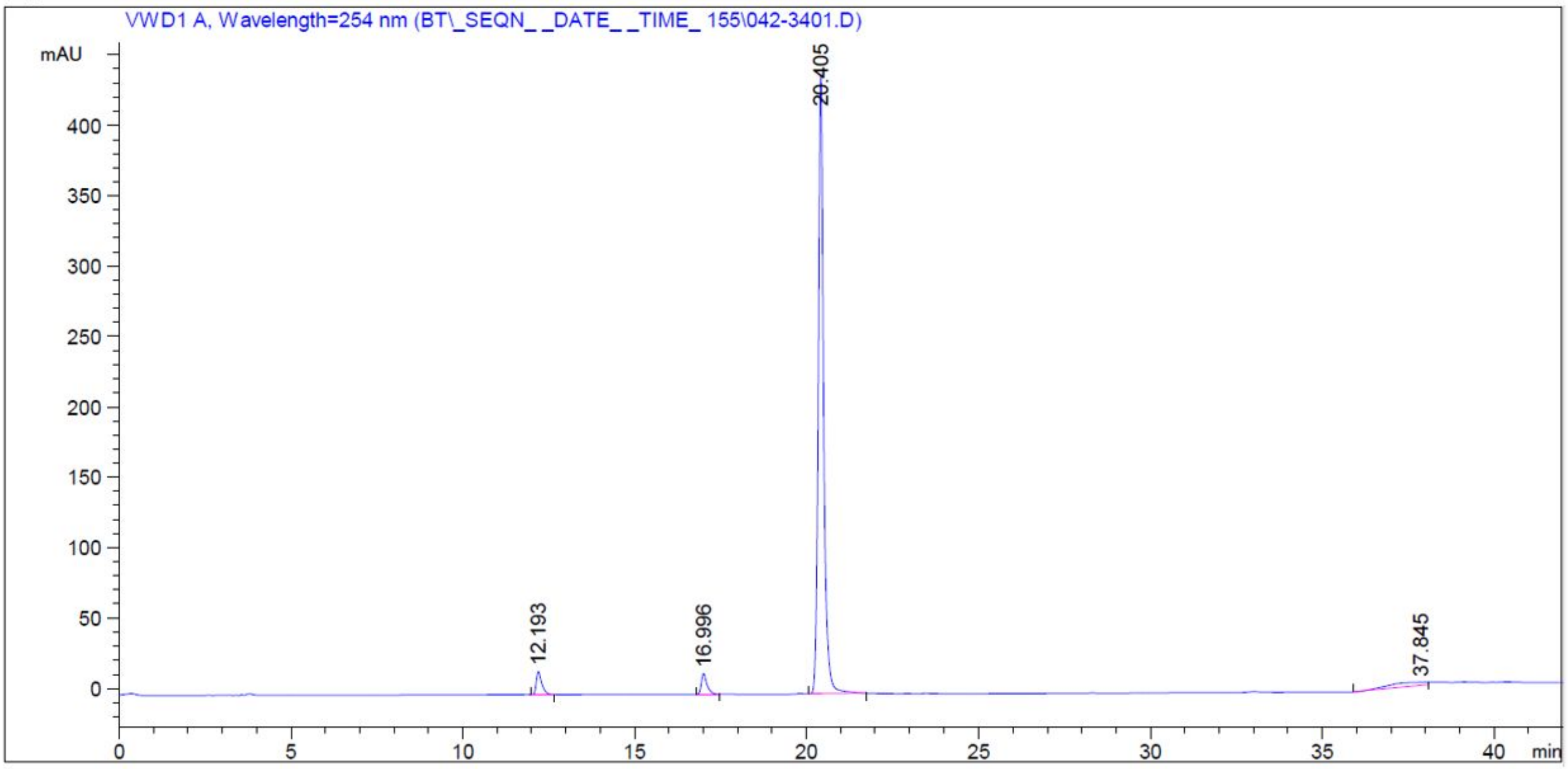

Jun14744 (zc8-p077-22)

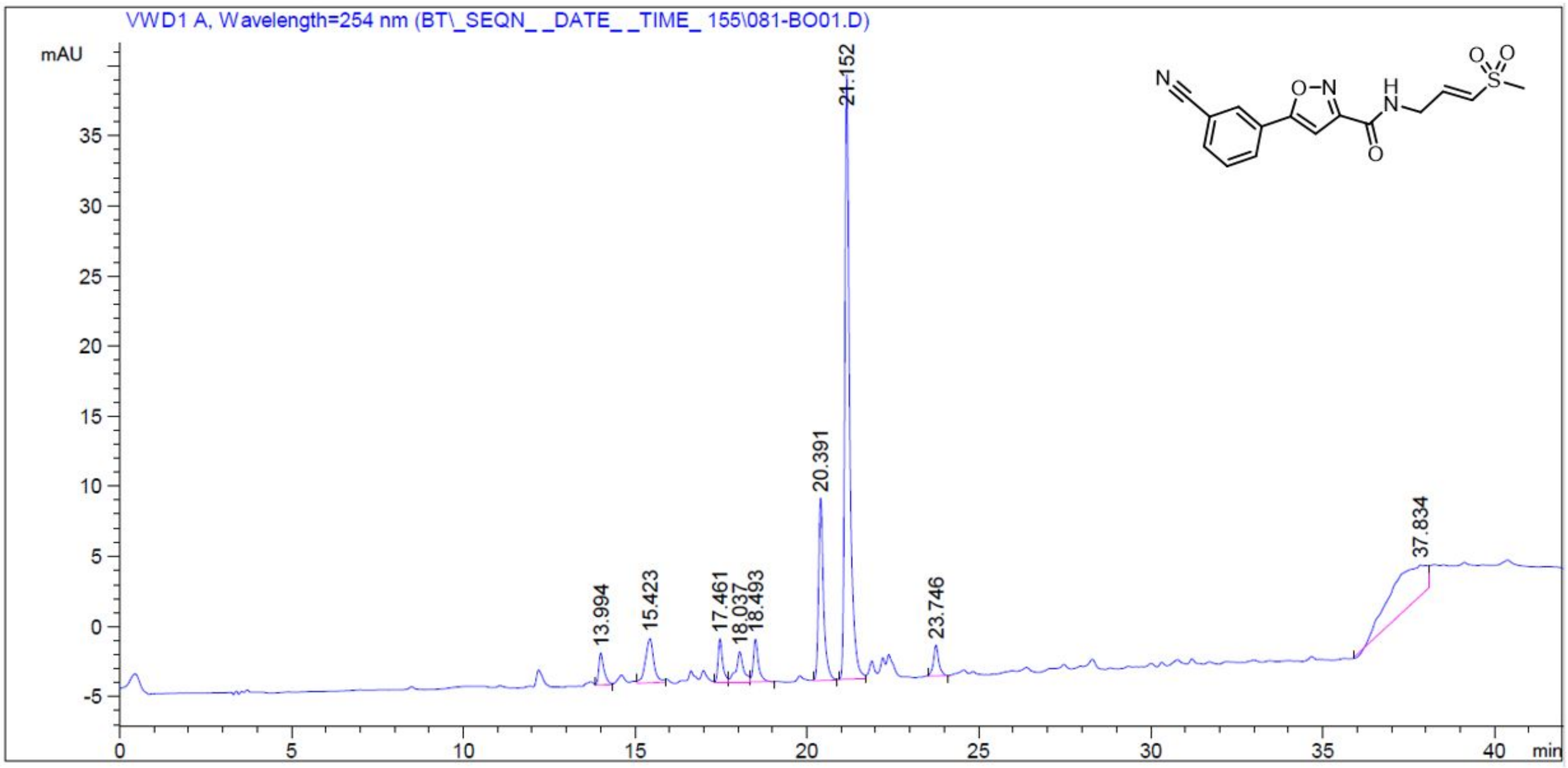

HPLC trace for Jun14853

8-p077-23s

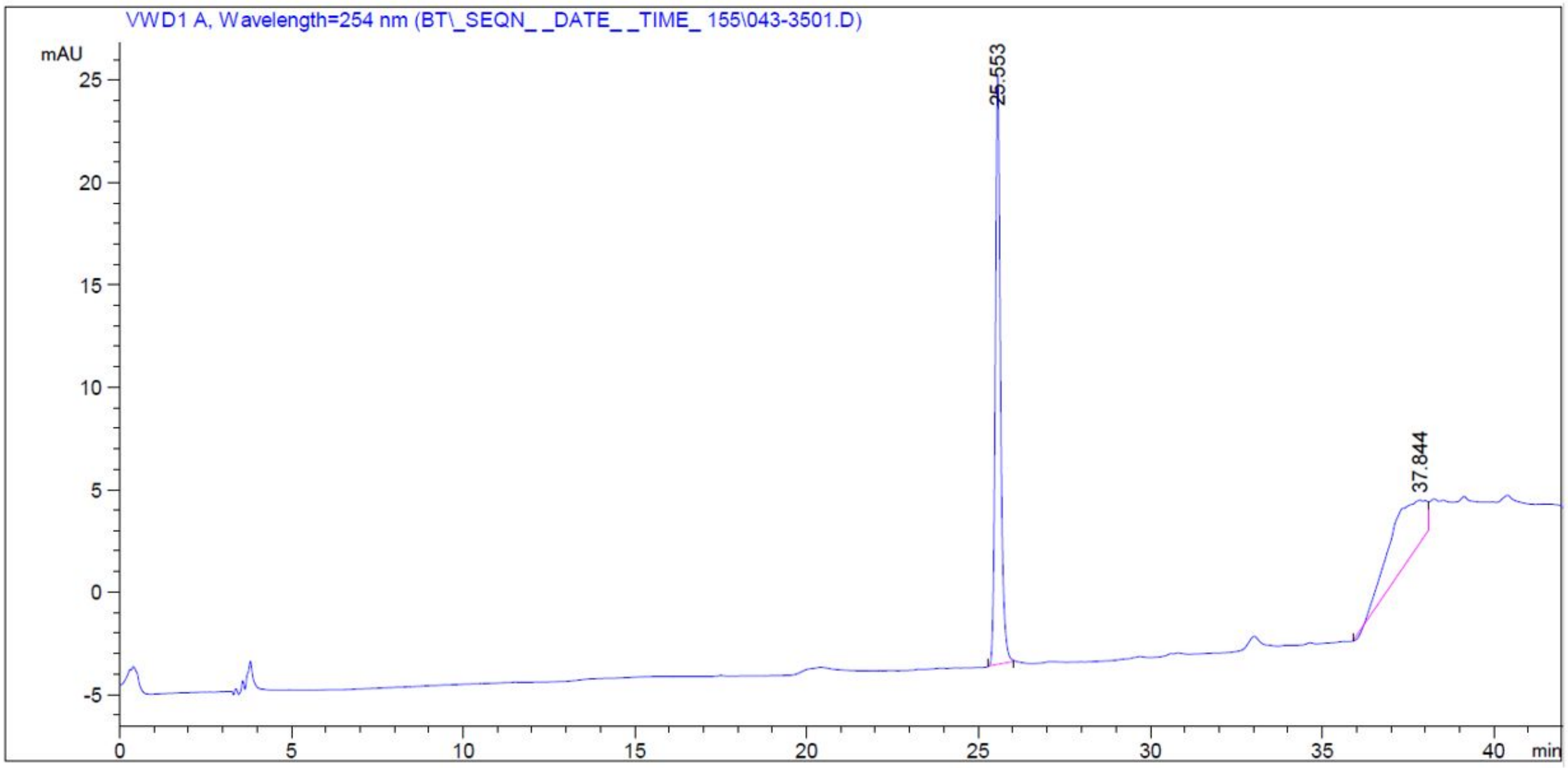

Jun14853 (zc8-p077-23)

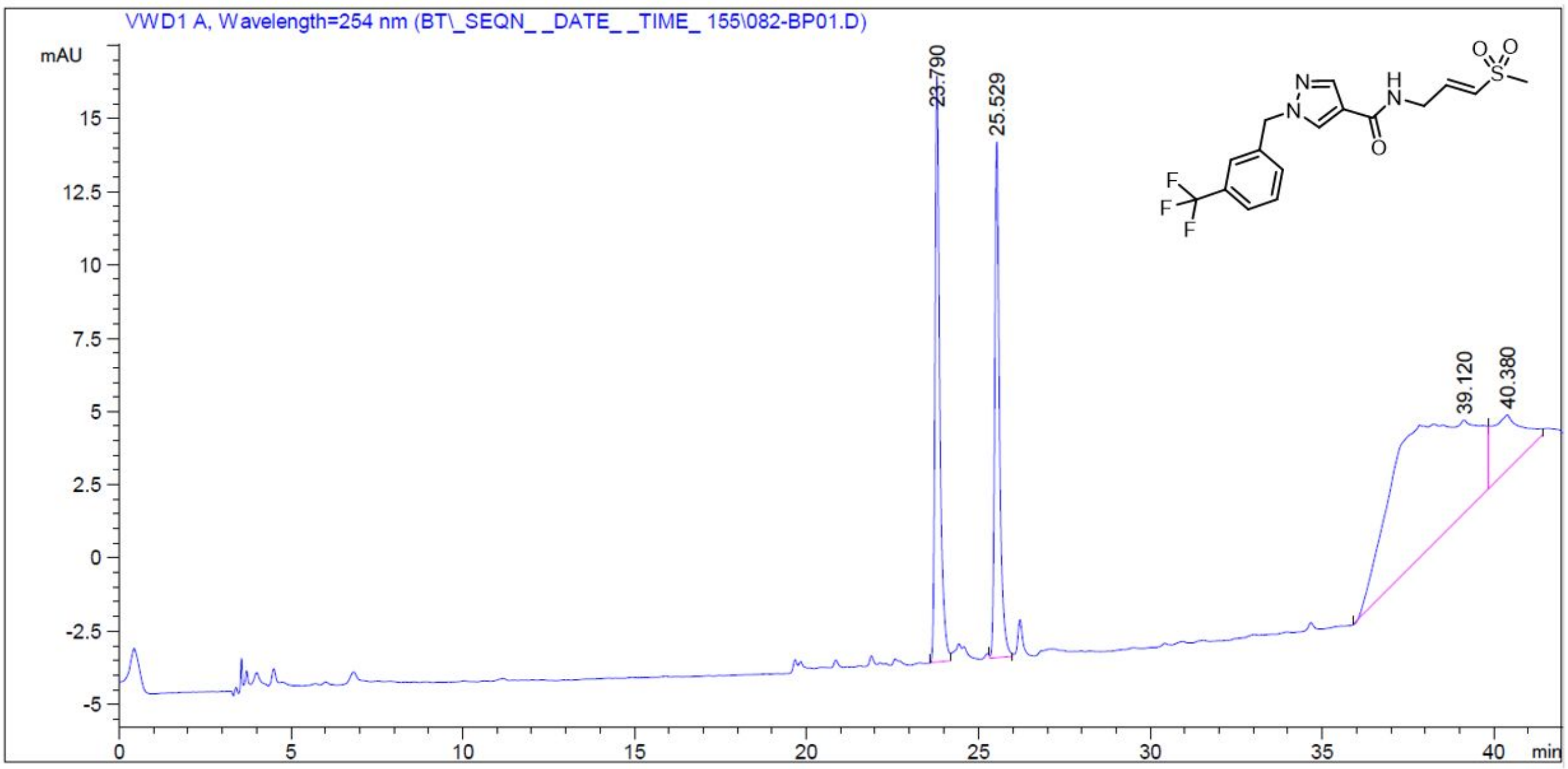

HPLC trace for Jun14897

8-p077-24s

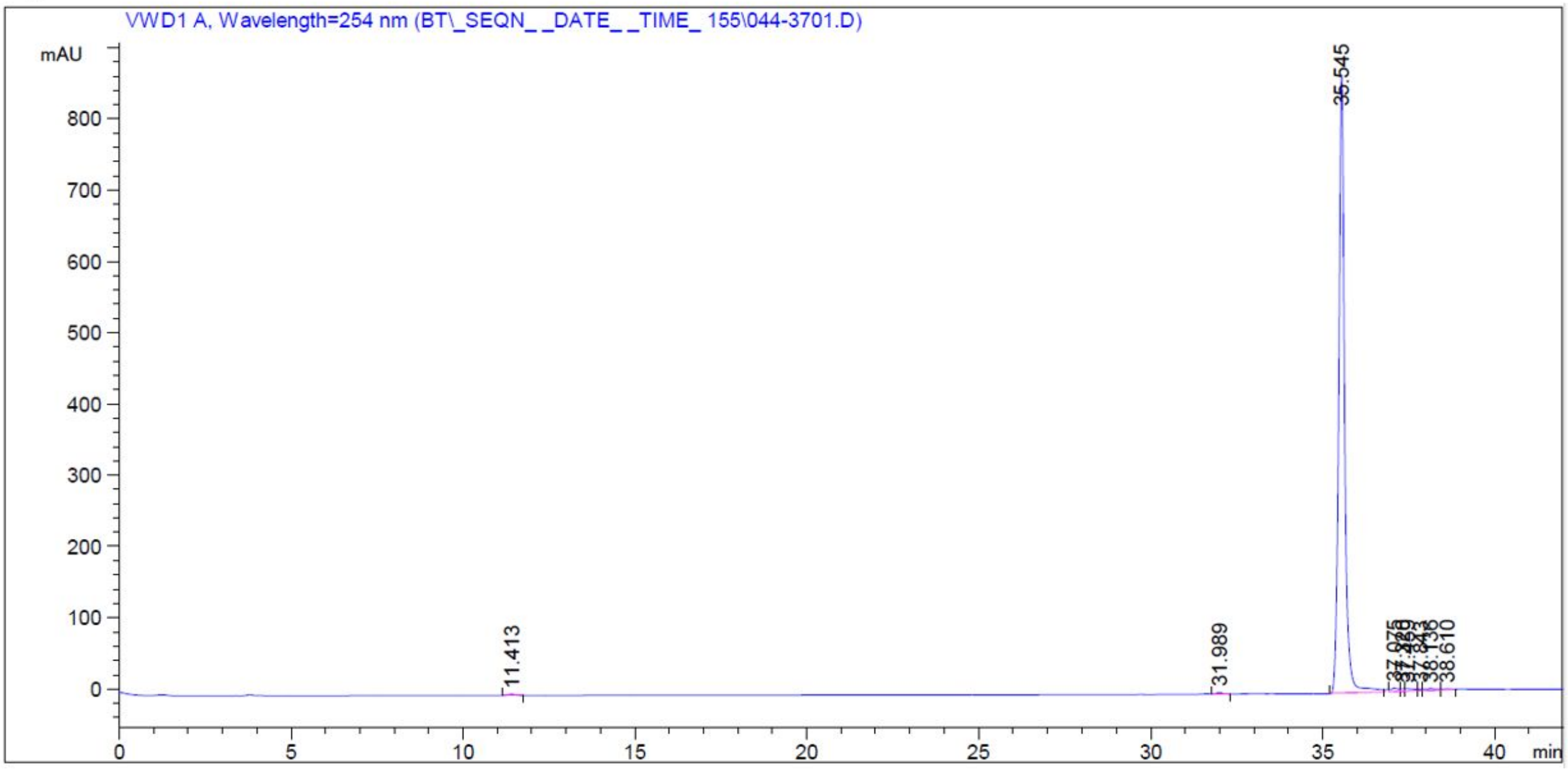

Jun14897 (zc8-p077-24)

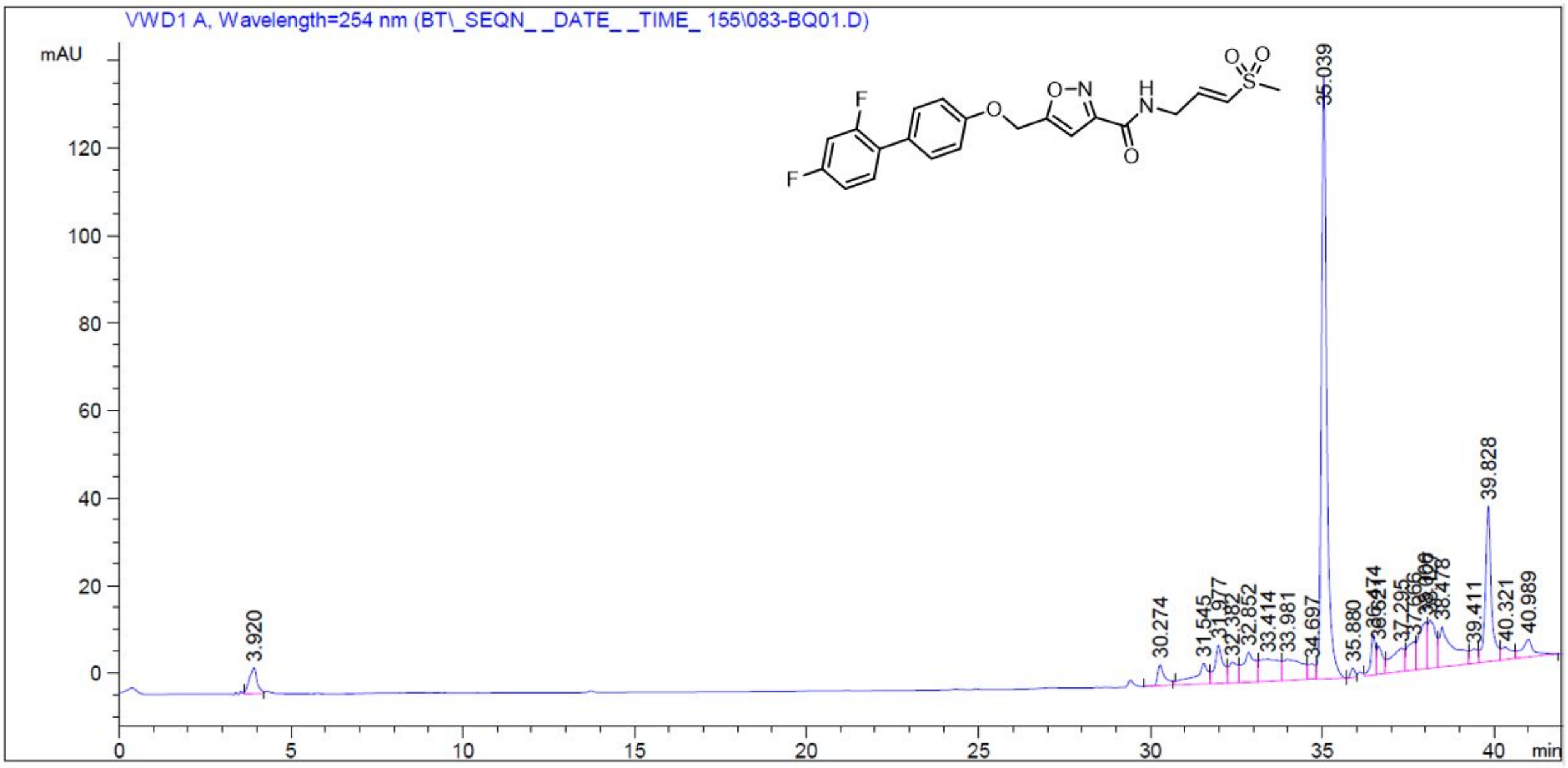

HPLC trace for Jun14924

8-p077-25s

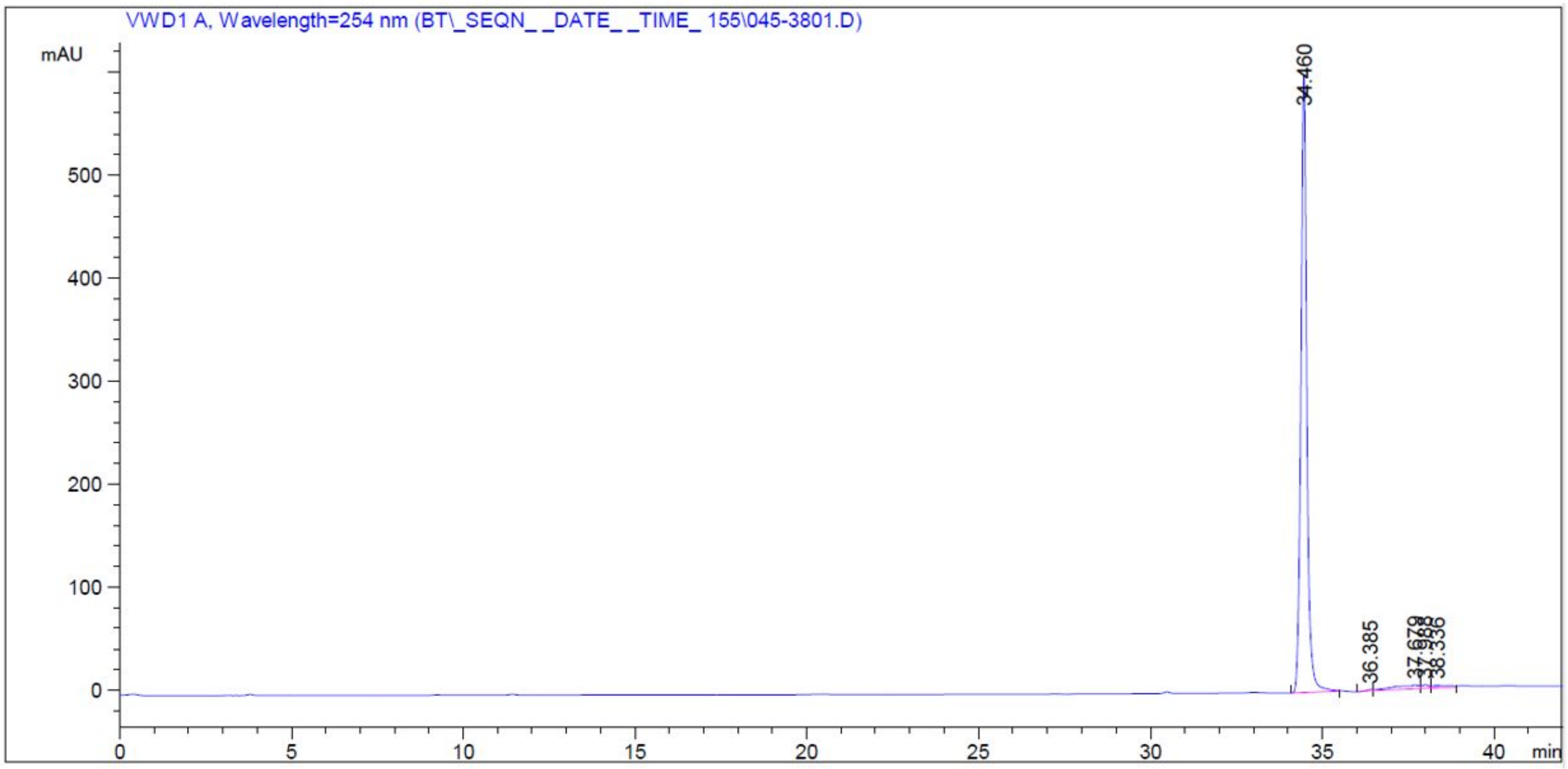

Jun14924 (8-p077-25)

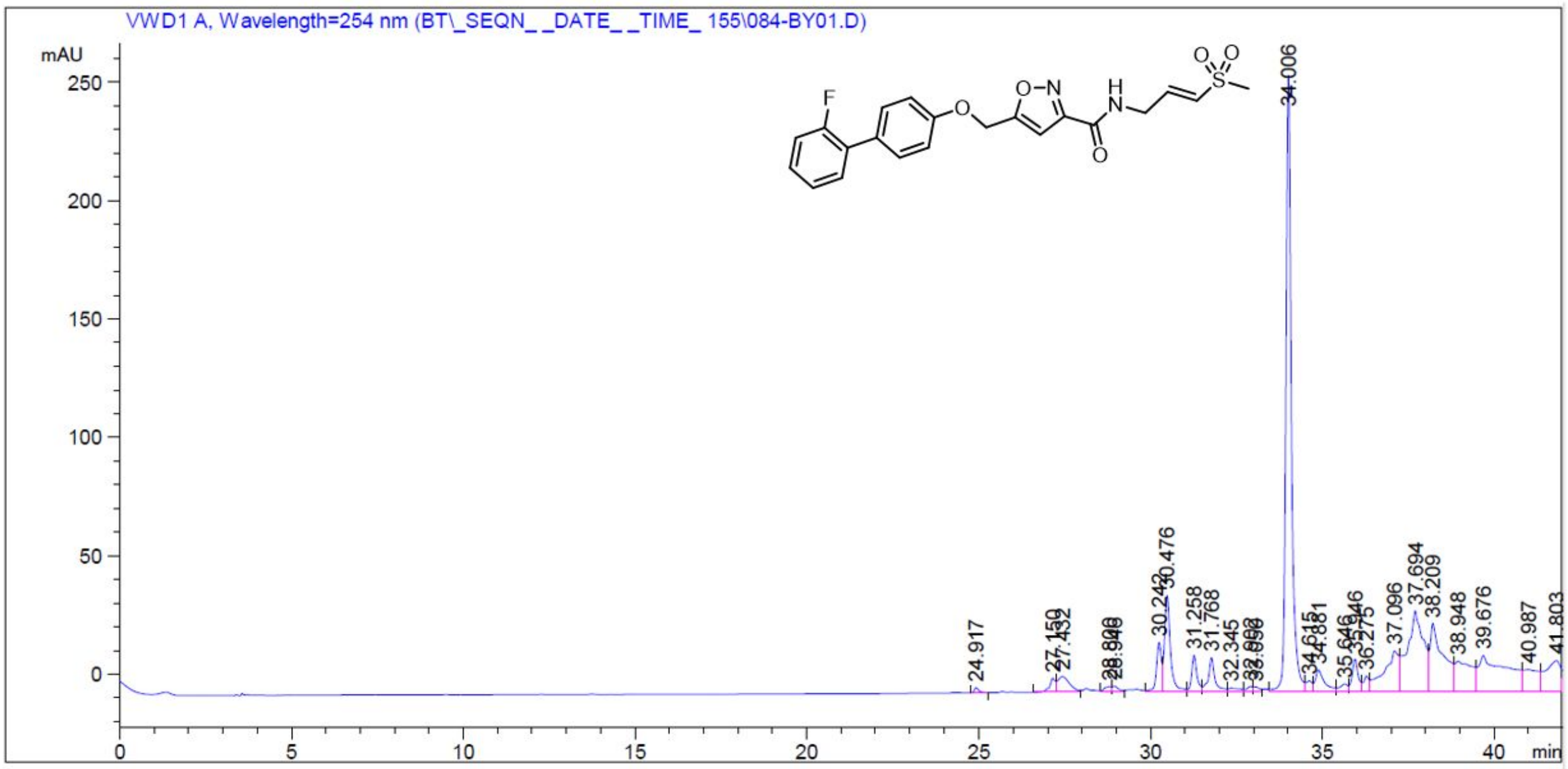

HPLC trace for Jun14926

8-p077-26s

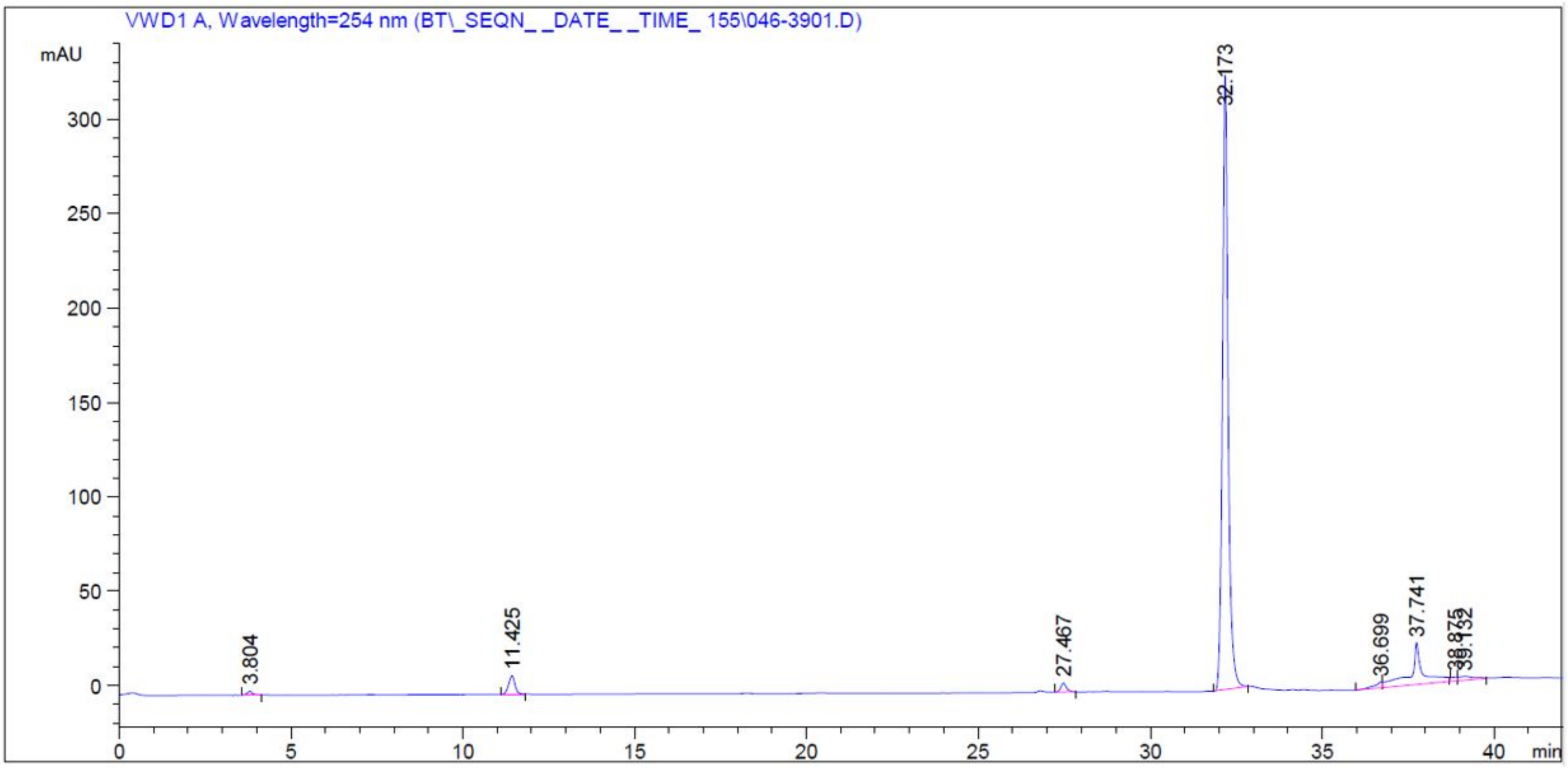

Jun14926 (zc8-p077-26)

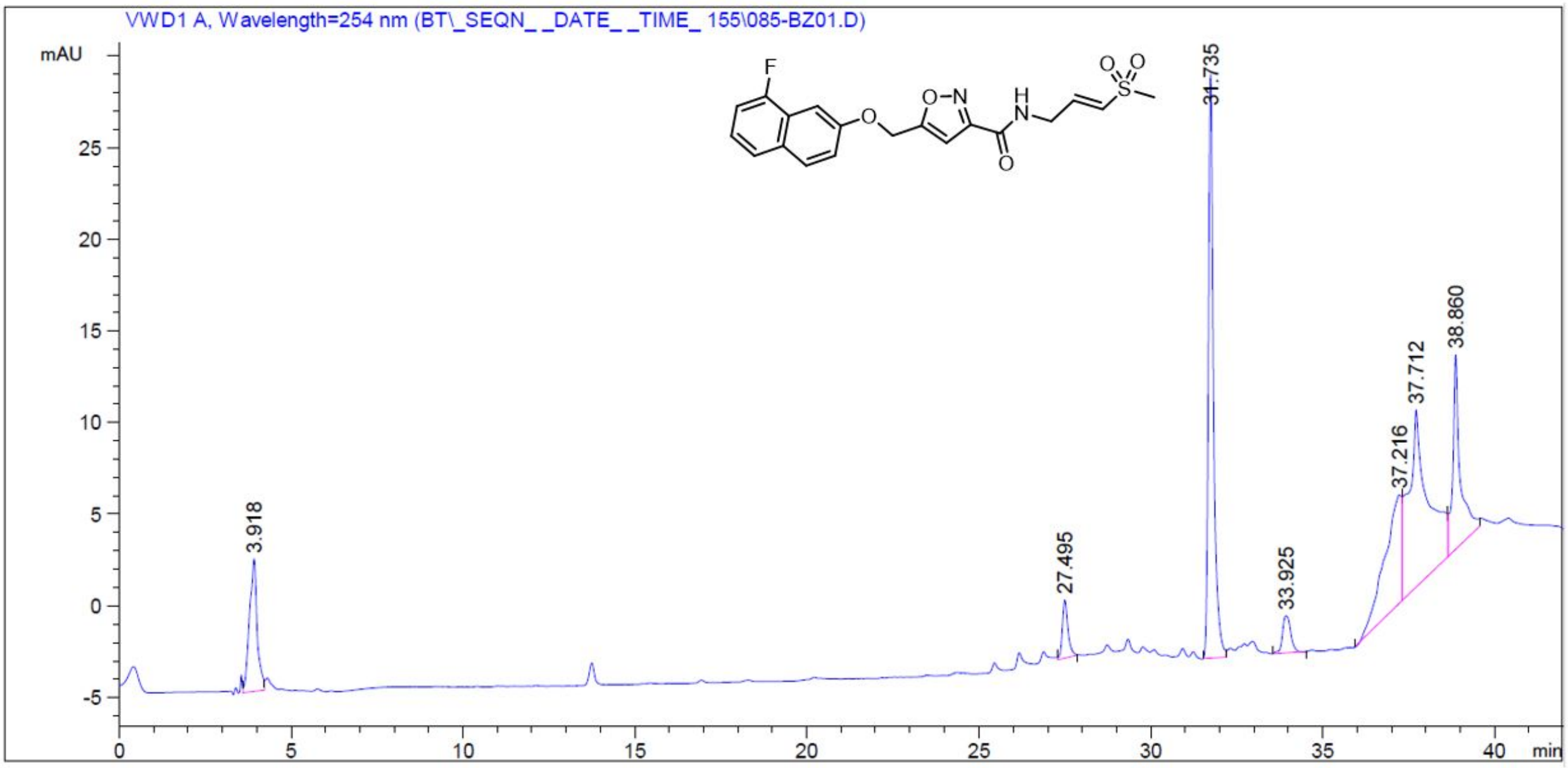

HPLC trace for Jun14927

8-p077-27s

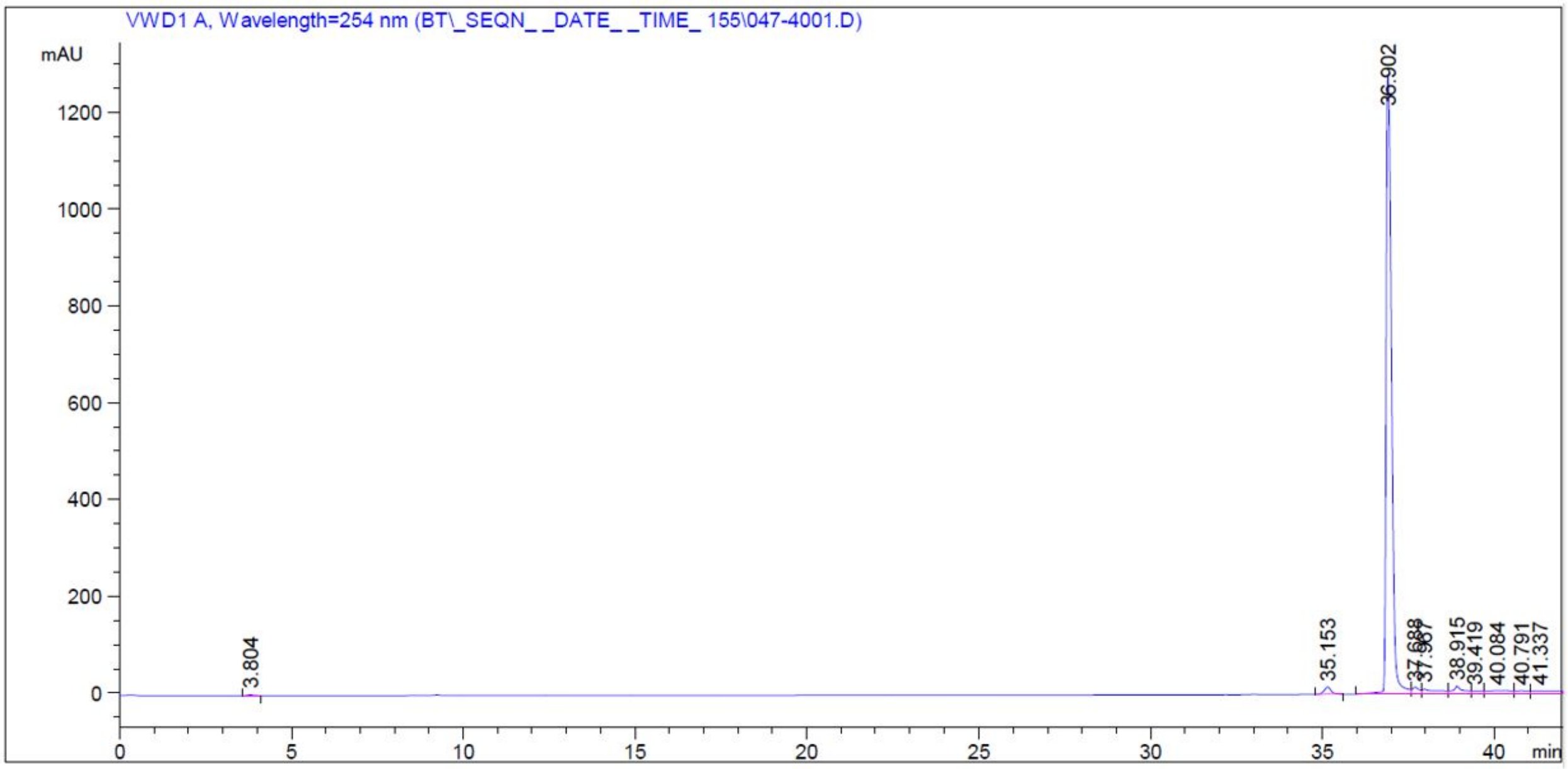

Jun14927 (zc8-p077-27)

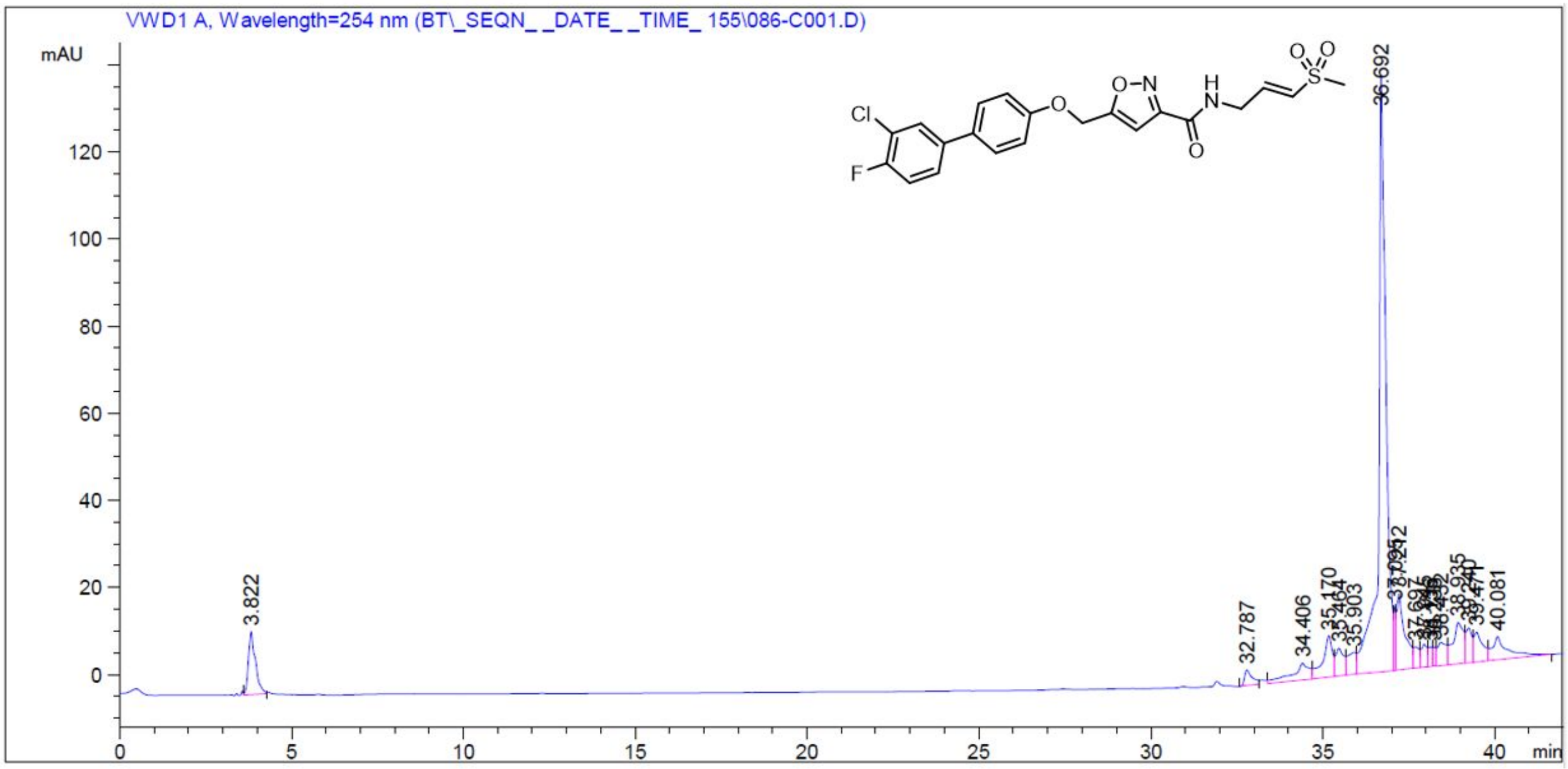

HPLC trace for Jun14966

8-p077-28s

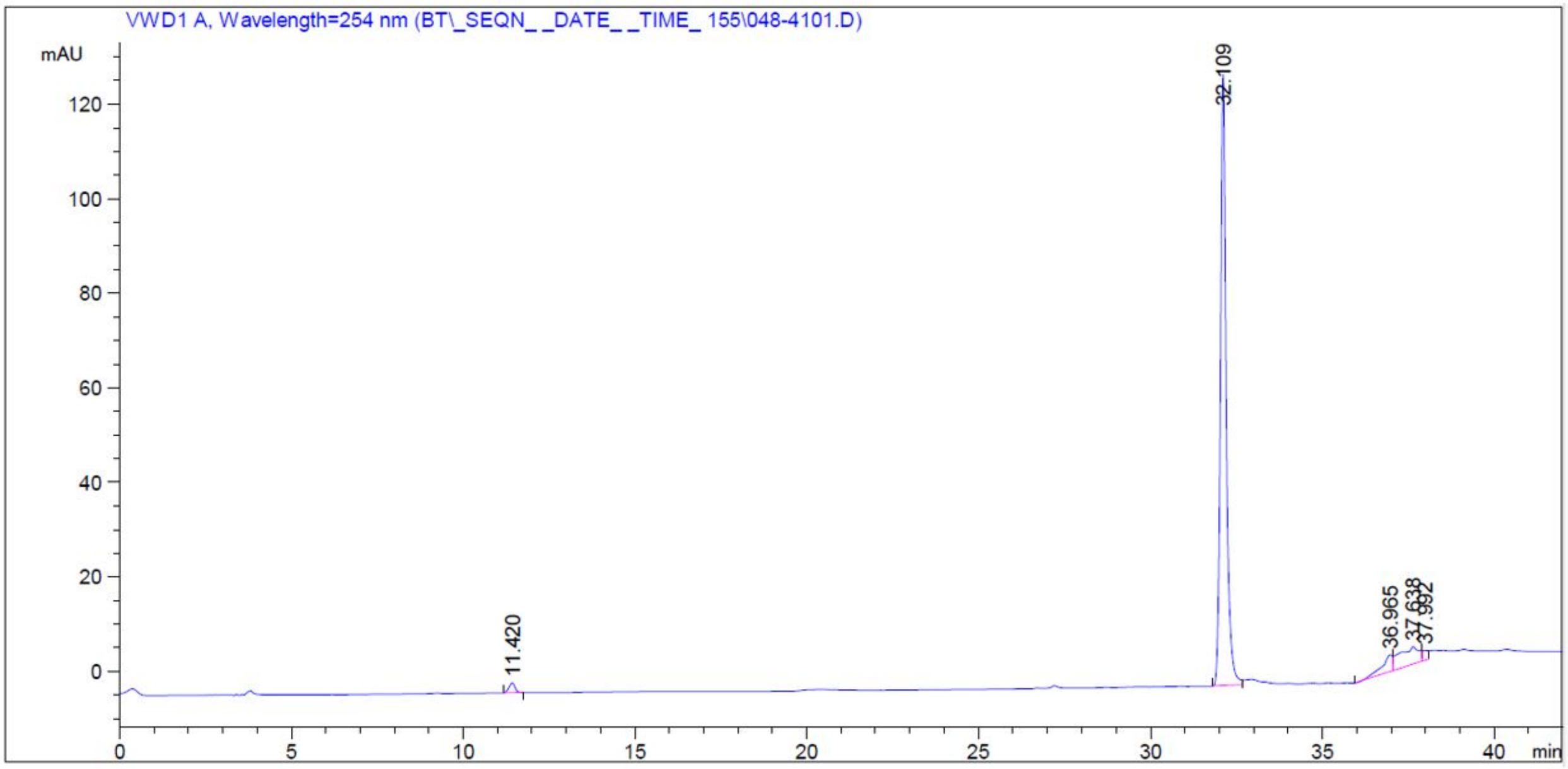

Jun14966 (zc8-p077-28)

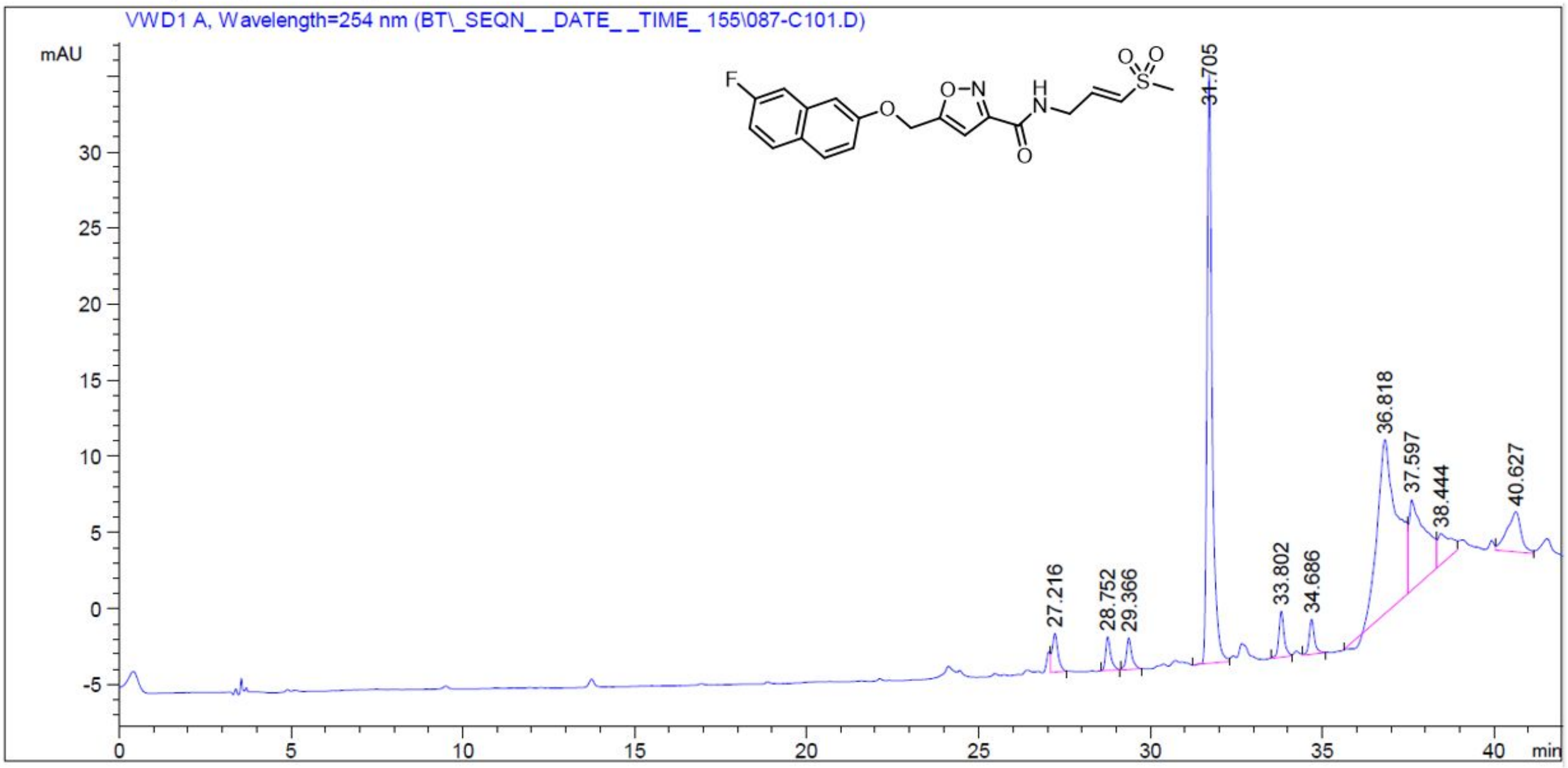

HPLC trace for Jun14969

8-p077-29s

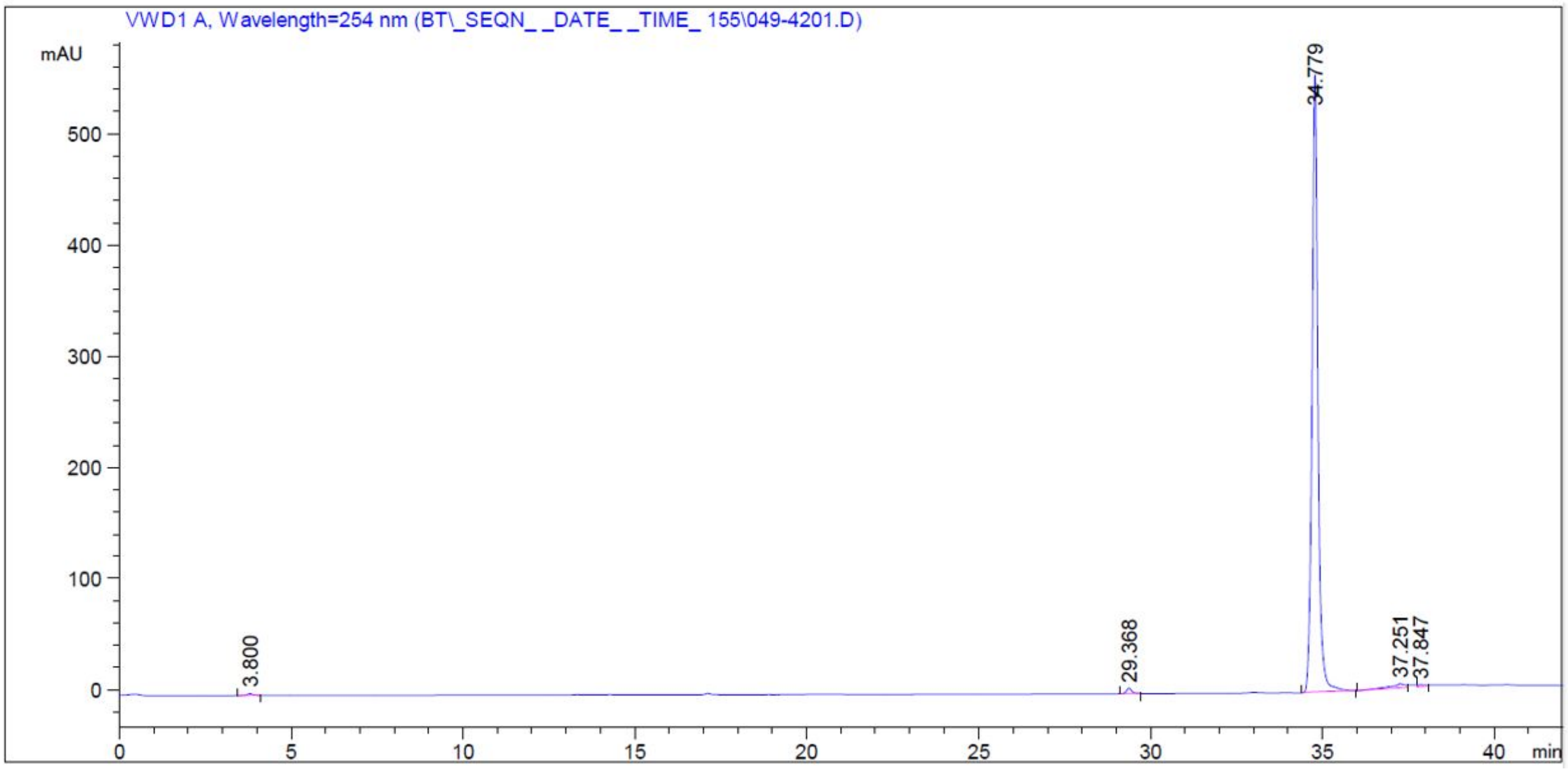

Jun14969 (zc8-p077-29)

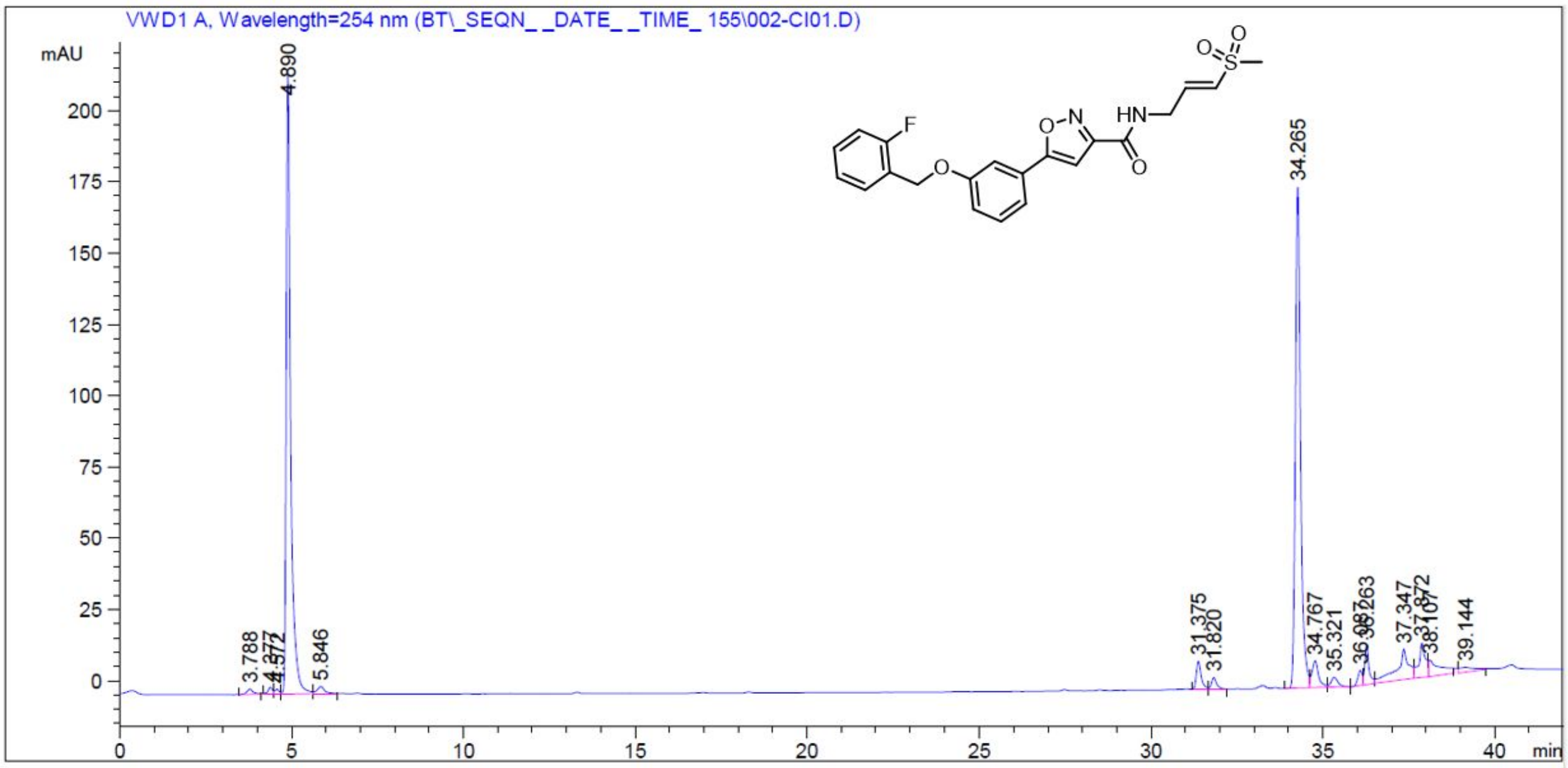

HPLC trace for Jun15111

8-p077-30s

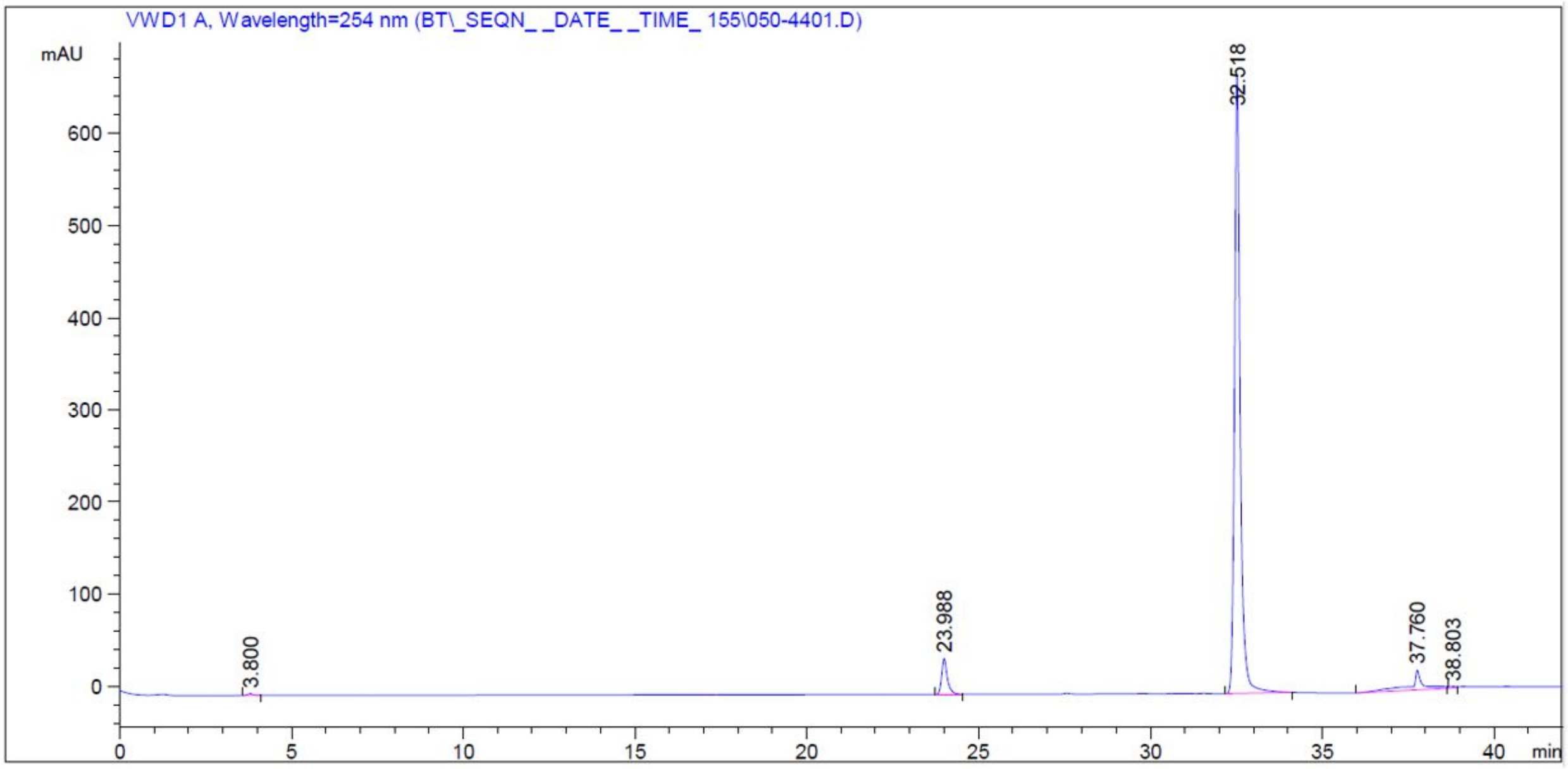

Jun15111 (zc8-p077-30)

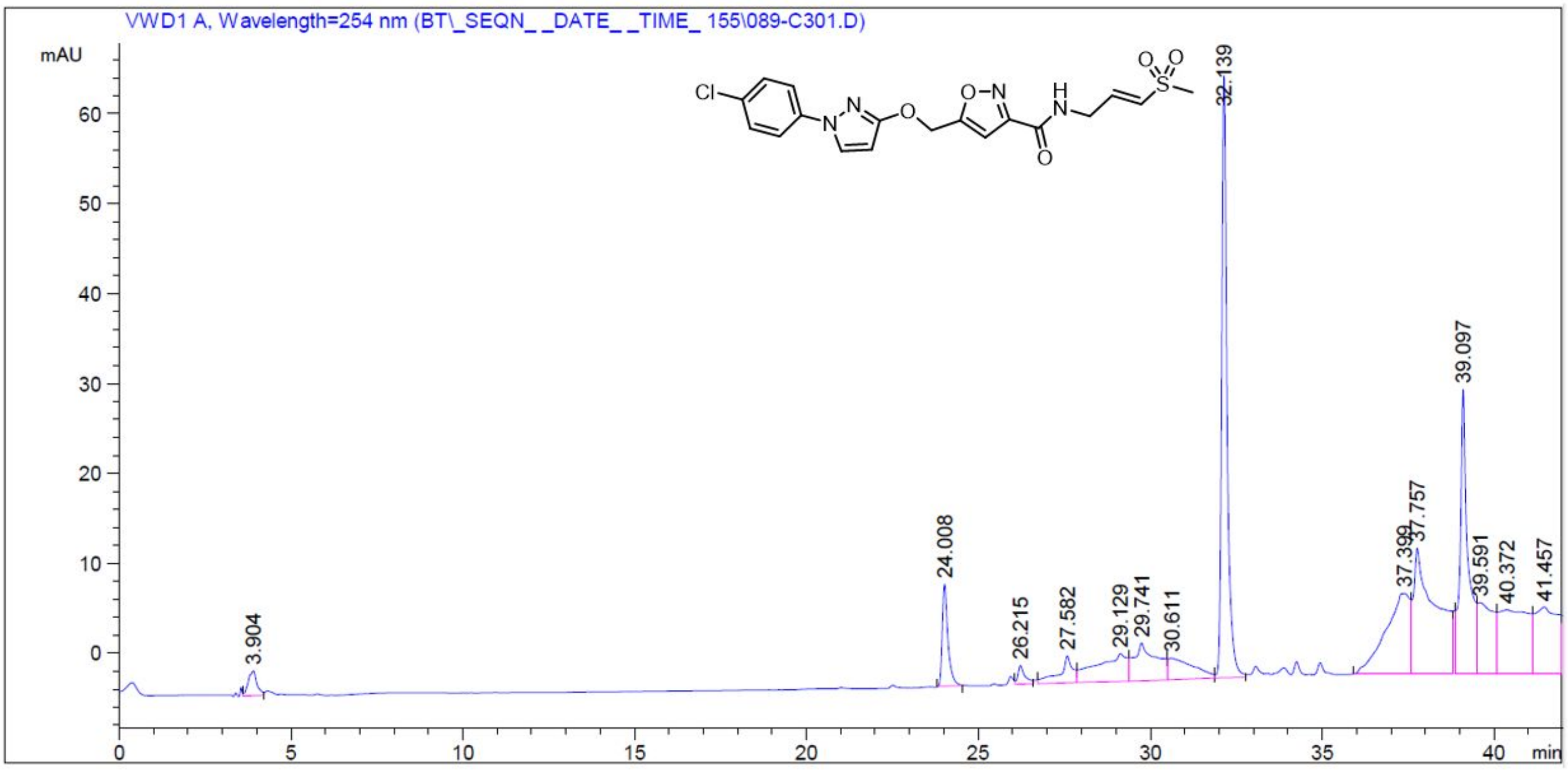

HPLC trace for Jun1551

8-p077-31s

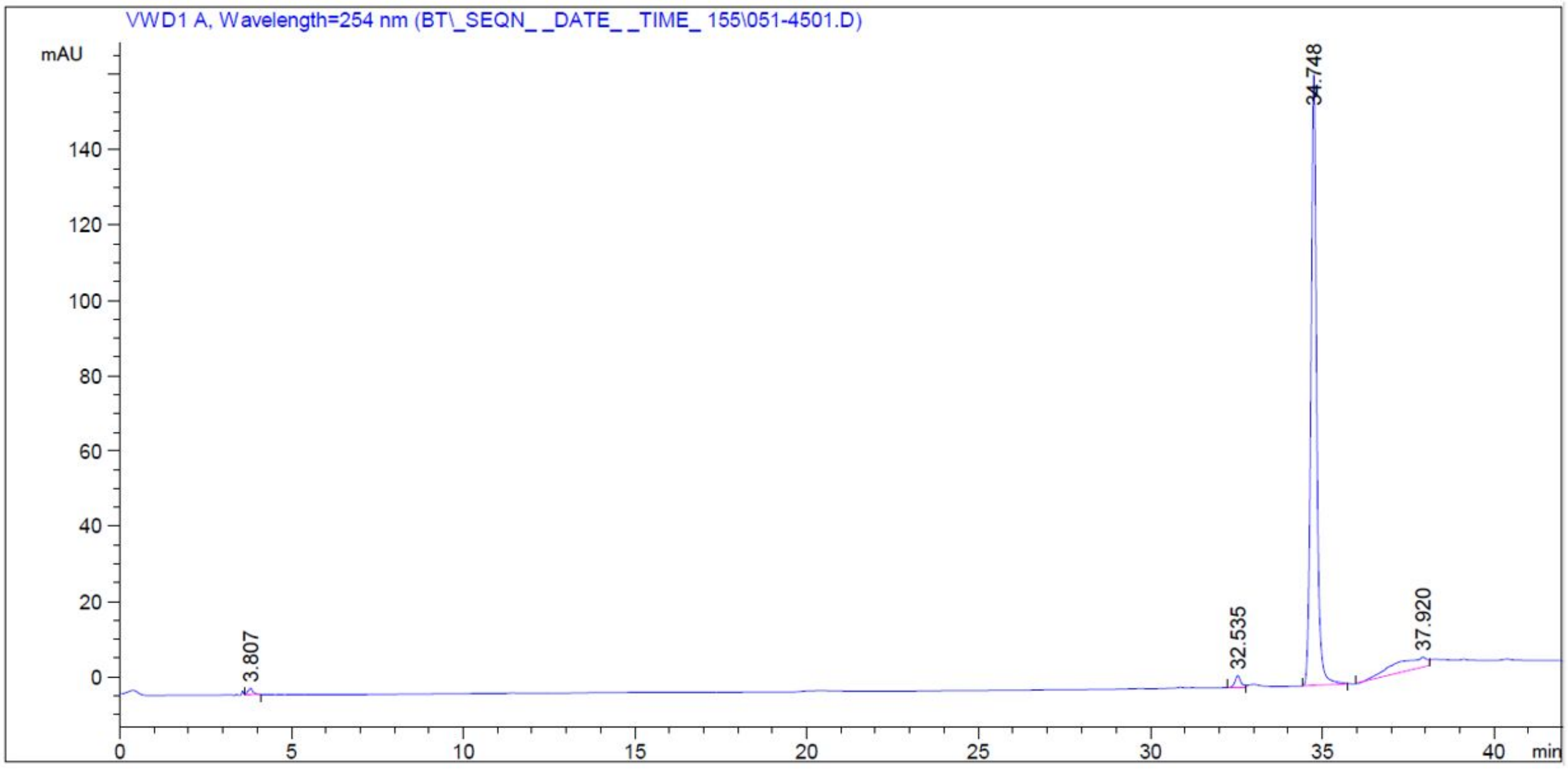

Jun1551 (zc8-p077-31)

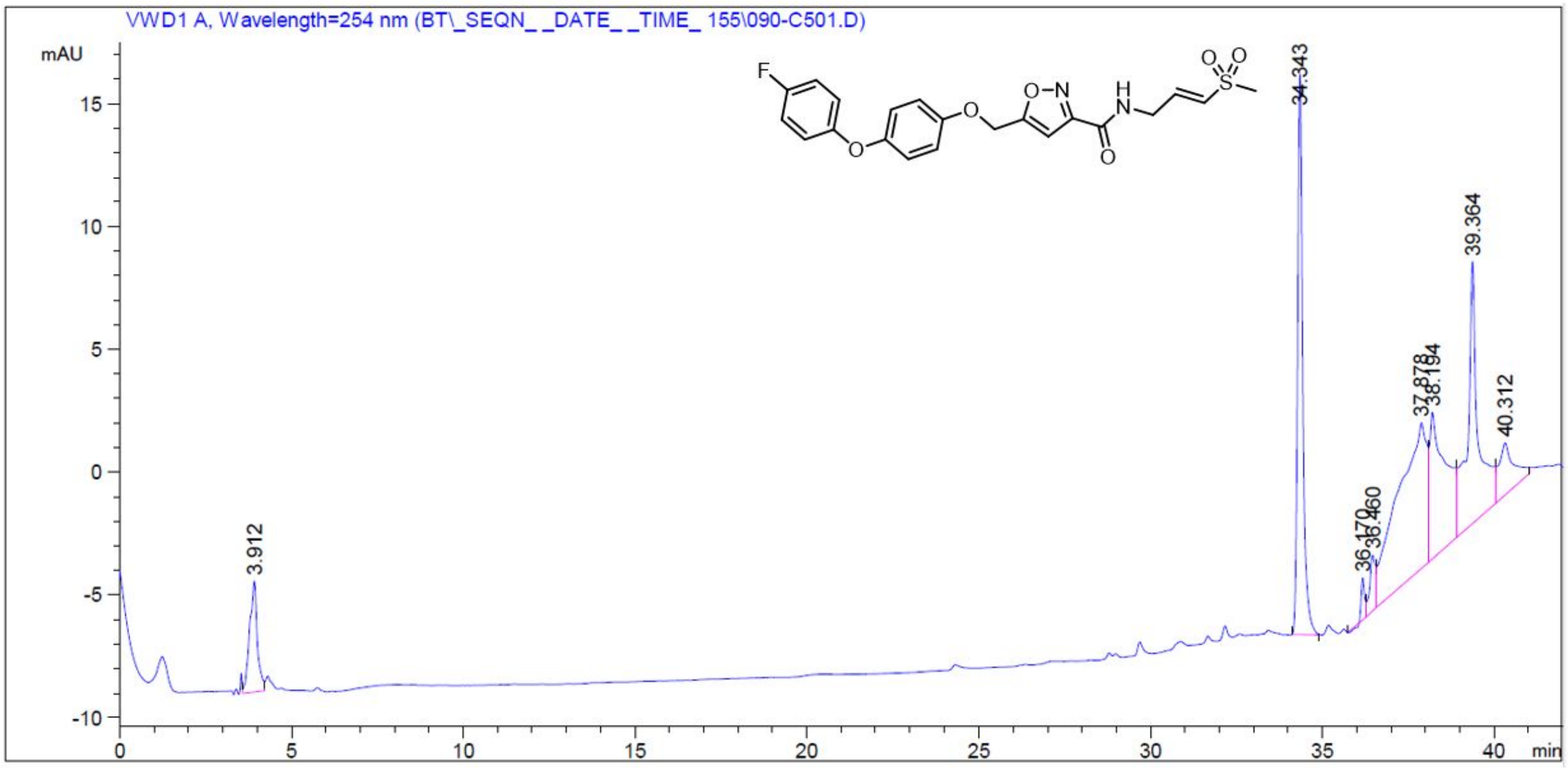

HPLC trace for Jun15172

8-p077-32s

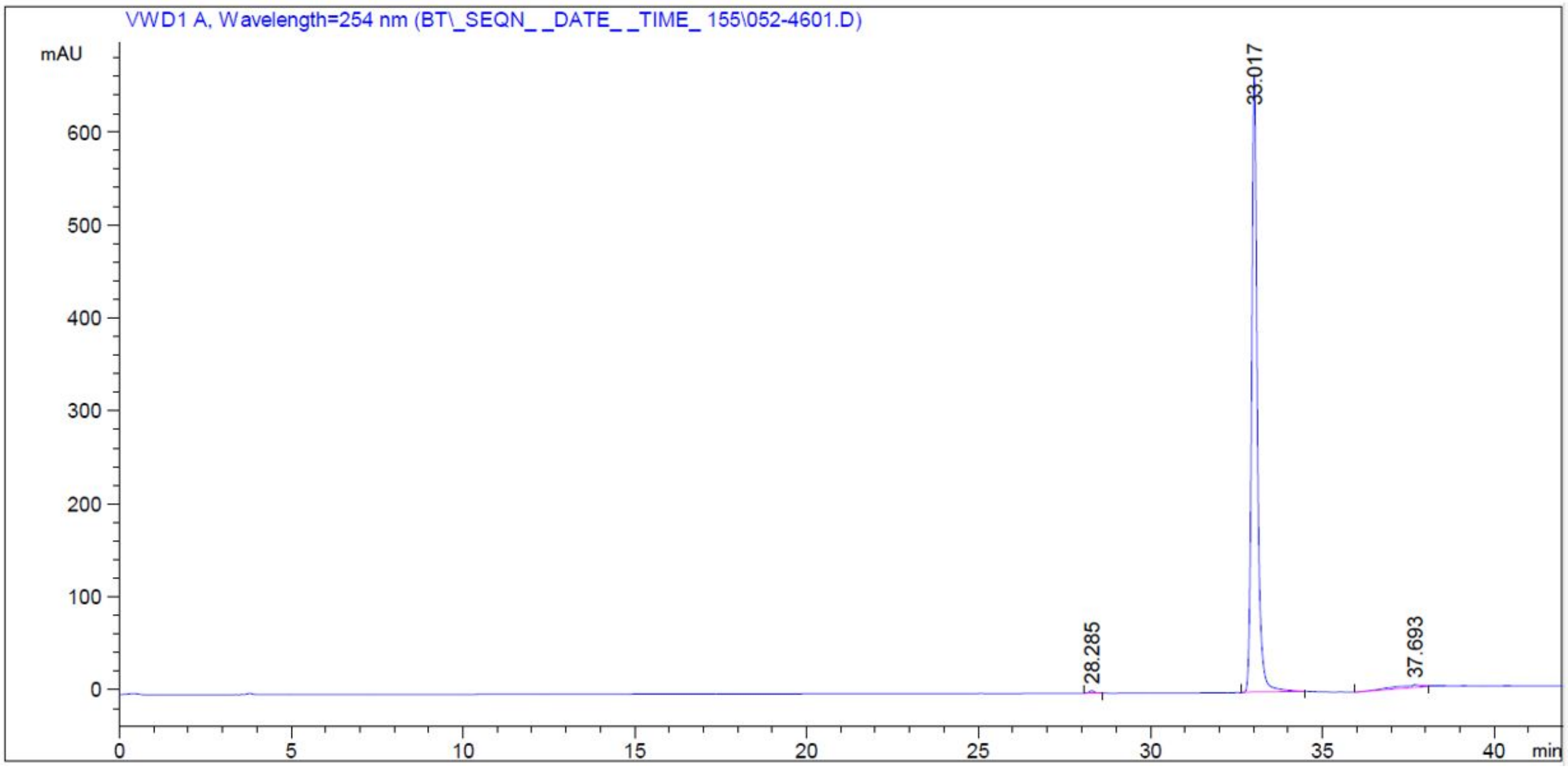

Jun15172 (zc8-p077-32)

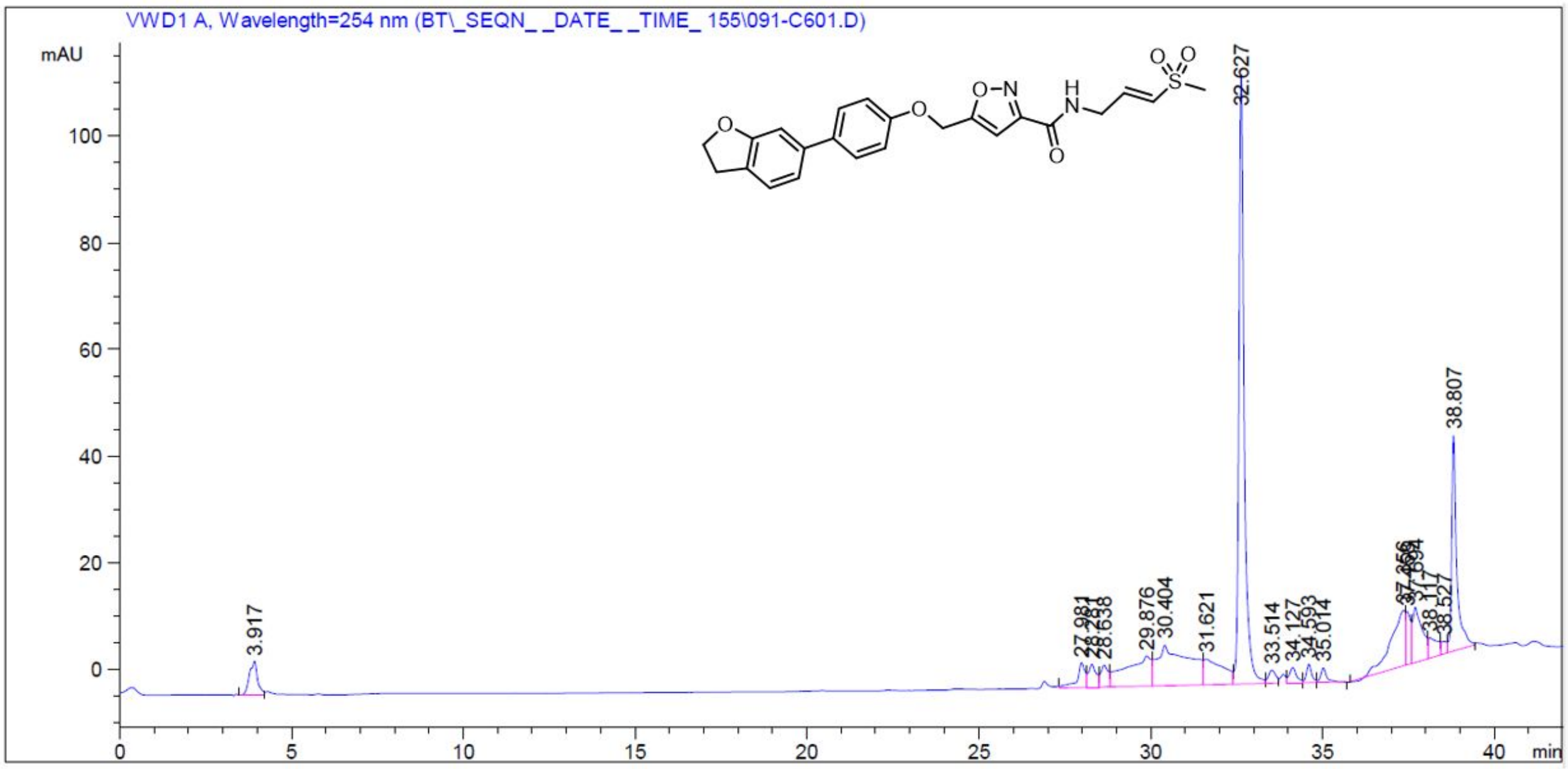

HPLC trace for Jun15175

8-p077-33s

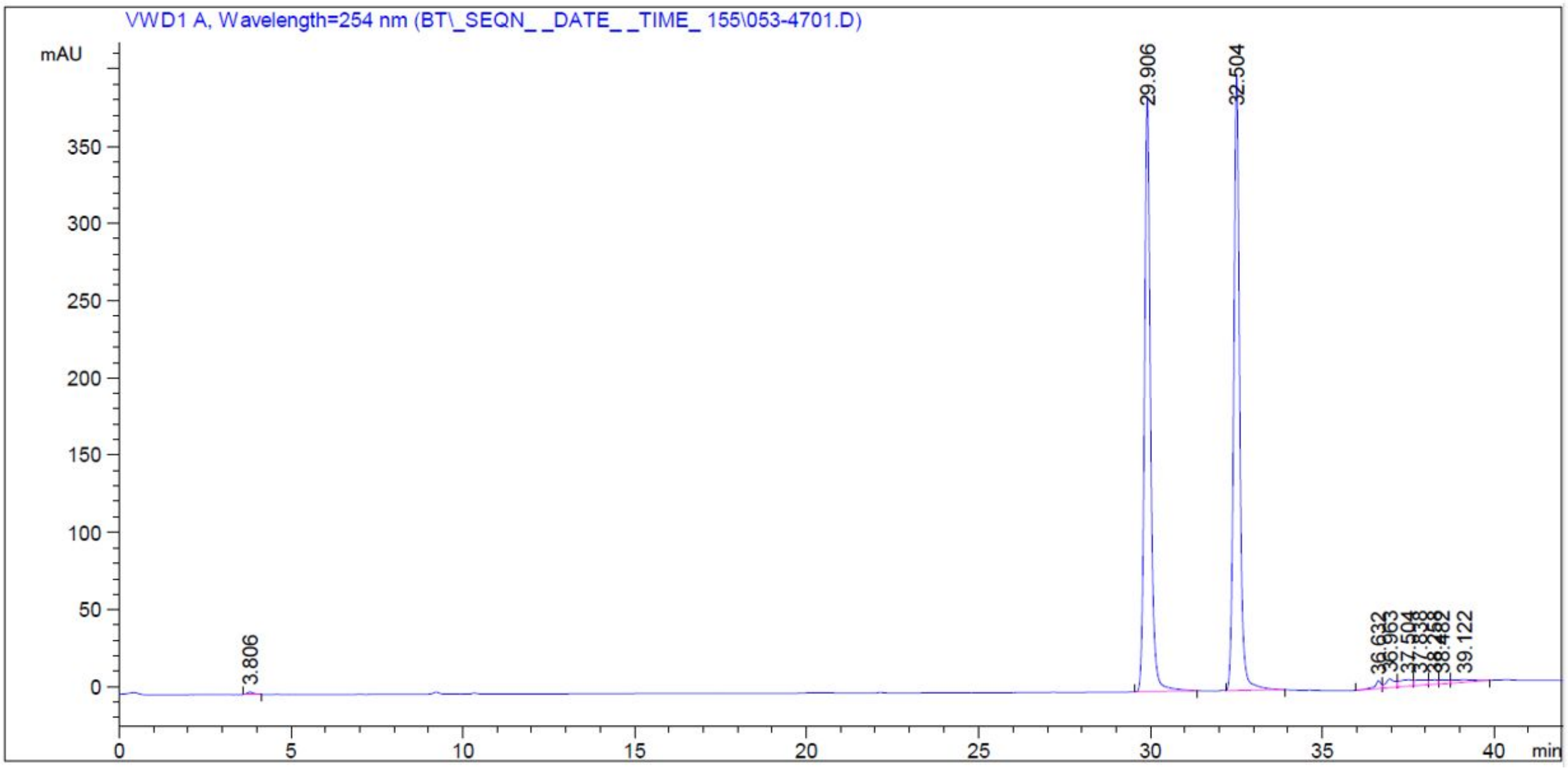

Jun15175 (zc8-p077-33)

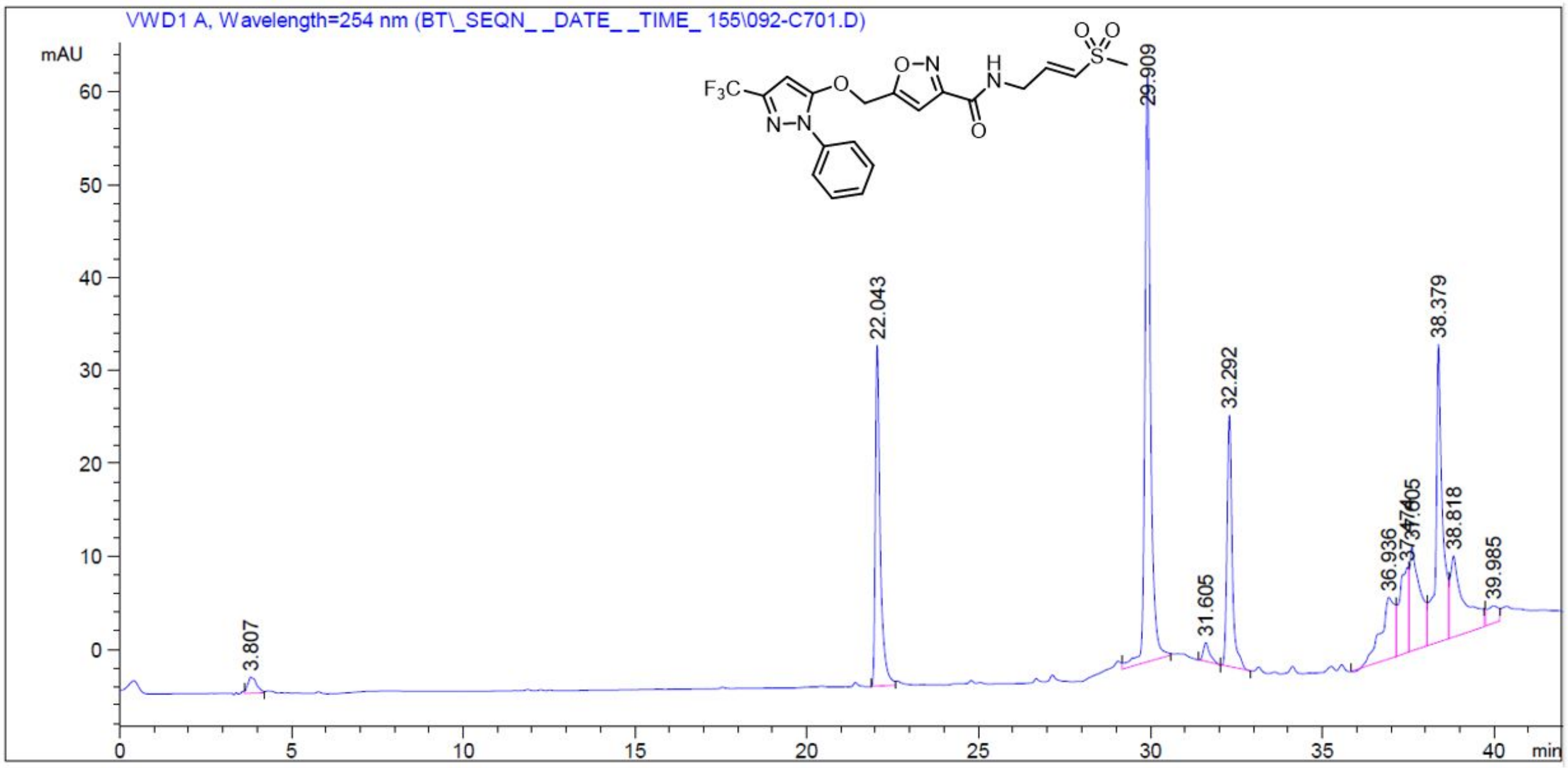

HPLC trace for Jun15176

8-p077-34s

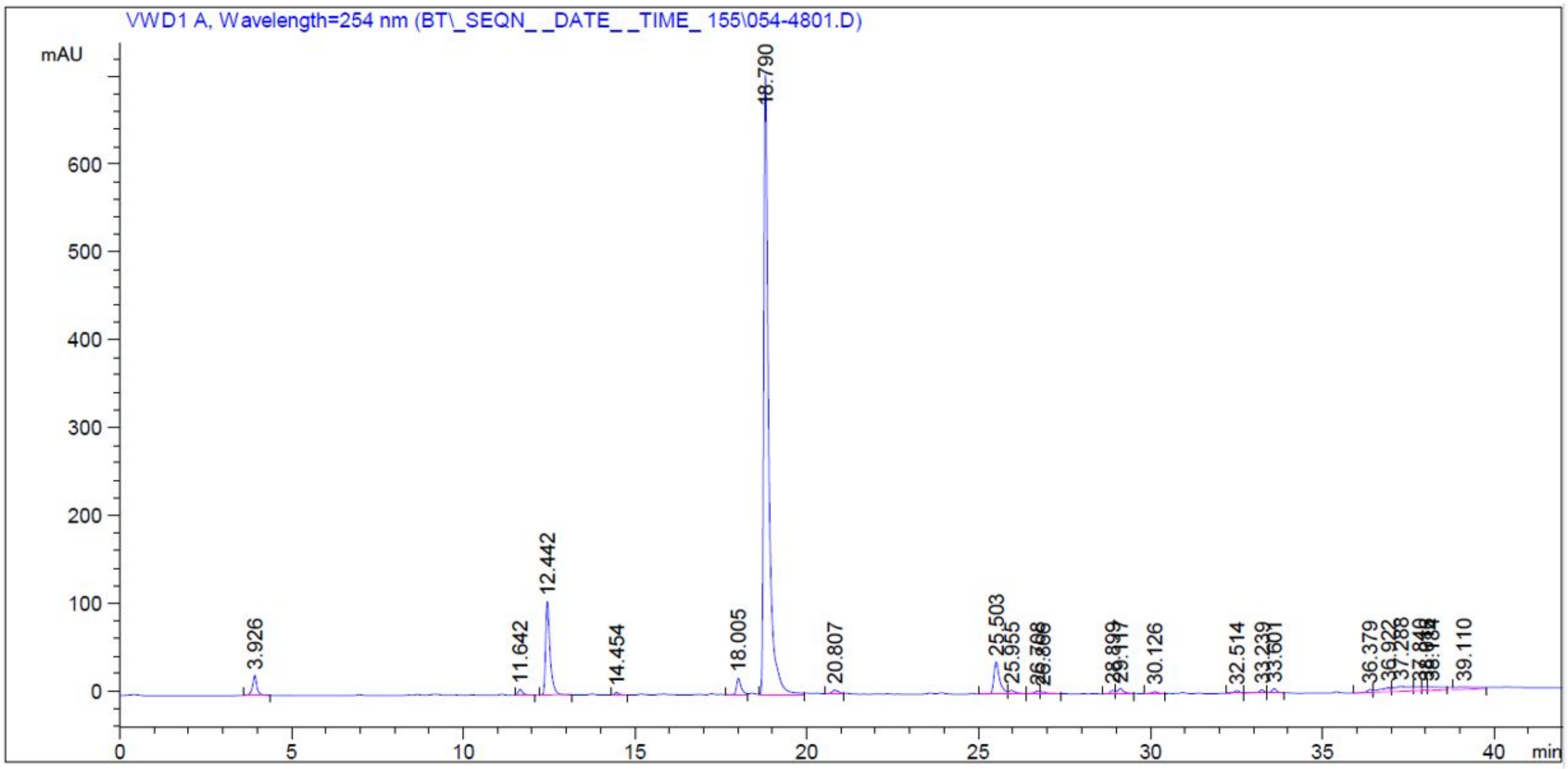

Jun15176 (zc8-p077-34)

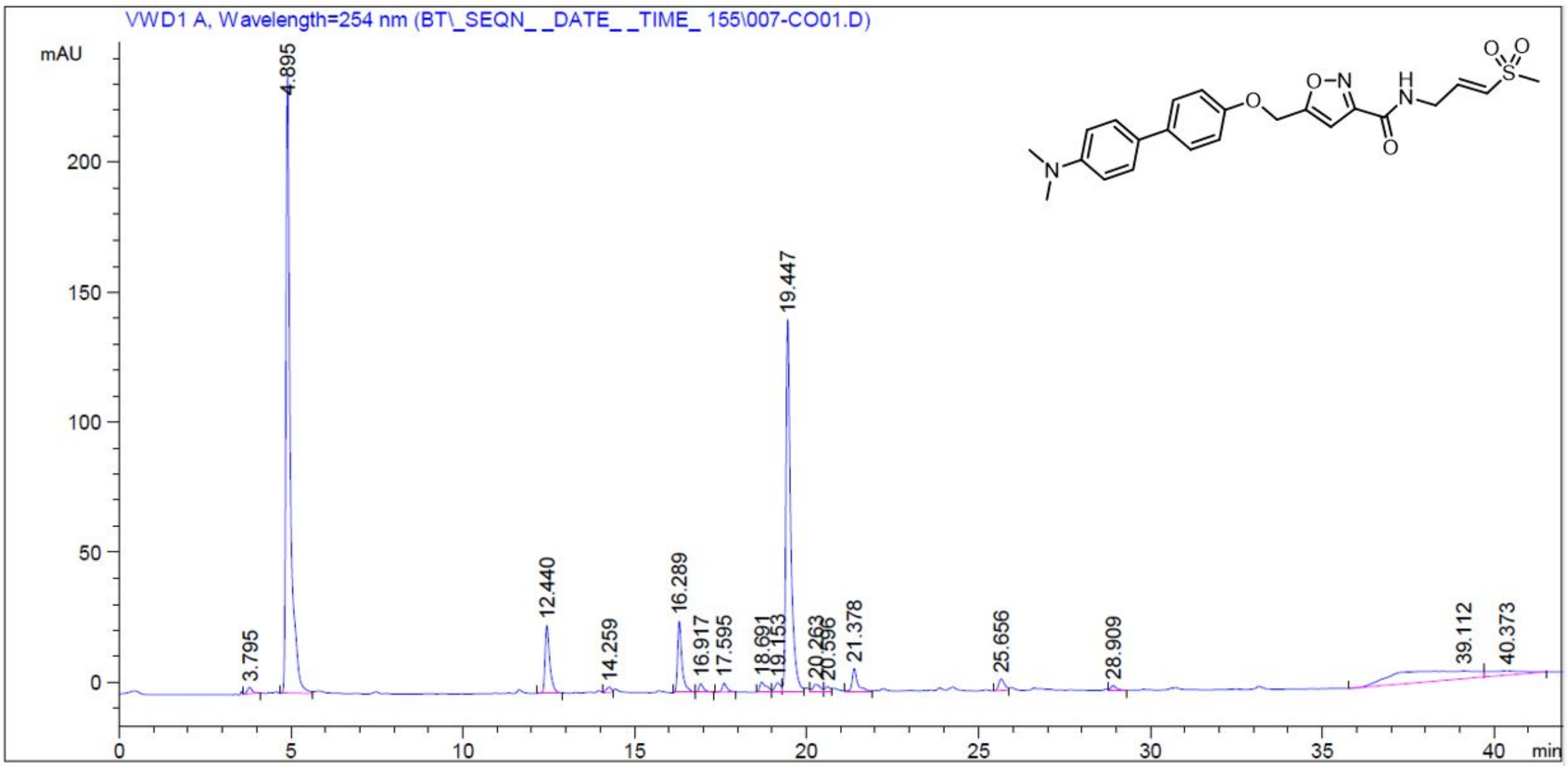

Supplement: Supplementary file 1 [file jm5c03672_si_001.pdf]
